# Supplementary material for: Comprehensive Quantitative Proteome Analysis of Aedes aegypti Identifies Proteins and Pathways Involved in Wolbachia pipientis and Zika Virus Interference Phenomenon
Source: Front Physiol. 2021 Feb 25;12:642237. doi: 10.3389/fphys.2021.642237 (PMC7947915; doi:10.3389/fphys.2021.642237)
Supplement: Supplementary file 2 [file Data_Sheet_2.PDF]

Proteins Ur	Checked	Protein FDI	Master	Accession	Descriptior	Exp. q-valu	Coverage [	Contamina	# Peptides
-5,56E+18	FALSO	High	IsMasterPr	AAEL02621	2:2938153	0	66	FALSO	238
8,79E+18	FALSO	High	IsMasterPr	AAEL00565	2:3820640	0	49	FALSO	160
-3,88E+18	FALSO	High	IsMasterPr	AAEL00282	3:2352398	0	89	FALSO	44
-7,90E+18	FALSO	High	IsMasterPr	AAEL01097	2:1511241	0	81	FALSO	119
3,26E+17	FALSO	High	IsMasterPr	AAEL00167	2:8708034	0	85	FALSO	44
8,13E+18	FALSO	High	IsMasterPr	AAEL01217	2:2043408	0	67	FALSO	64
9,01E+18	FALSO	High	IsMasterPr	AAEL01506	2:1918218	0	60	FALSO	161
5,66E+18	FALSO	High	IsMasterPr	AAEL01726	2:2884763	0	58	FALSO	19
6,36E+18	FALSO	High	IsMasterPr	AAEL00463	1:3062451	0	72	FALSO	40
-8,14E+18	FALSO	High	IsMasterPr	AAEL01698	2:2968944	0	90	FALSO	31
-7,30E+18	FALSO	High	IsMasterPr	AAEL00584	3:2188073	0	61	FALSO	130
-7,71E+18	FALSO	High	IsMasterPr	AAEL00995	3:7768756	0	50	FALSO	139
-3,74E+18	FALSO	High	IsMasterPr	AAEL00192	3:2275272	0	75	FALSO	39
2,08E+18	FALSO	High	IsMasterPr	AAEL01206	1:2705567	0	46	FALSO	56
-7,37E+17	FALSO	High	IsMasterPr	AAEL01206	1:2705567	0	46	FALSO	56
5,44E+18	FALSO	High	IsMasterPr	AAEL00256	1:2274336	0	30	FALSO	179
3,21E+18	FALSO	High	IsMasterPr	AAEL01097	2:1511747	0	57	FALSO	59
-1,14E+18	FALSO	High	IsMasterPr	AAEL00469	1:1589610	0	73	FALSO	67
5,15E+18	FALSO	High	IsMasterPr	AAEL01961	2:6350079	0	27	FALSO	150
-3,97E+18	FALSO	High	IsMasterPr	AAEL02230	2:2744089	0	86	FALSO	30
4,34E+17	FALSO	High	IsMasterPr	AAEL02518	NIGP01001	0	38	FALSO	132
9,18E+18	FALSO	High	IsMasterPr	AAEL00461	1:3062257	0	43	FALSO	17
-6,64E+18	FALSO	High	IsMasterPr	AAEL01289	2:3659501	0	52	FALSO	49
-4,06E+18	FALSO	High	IsMasterPr	AAEL00918	2:9746568	0	34	FALSO	43
-5,76E+18	FALSO	High	IsMasterPr	AAEL00257	1:2290812	0	90	FALSO	21
-2,45E+17	FALSO	High	IsMasterPr	AAEL00918	2:9759648	0	60	FALSO	43
1,31E+18	FALSO	High	IsMasterPr	AAEL00984	3:1836671	0	36	FALSO	118
-4,15E+18	FALSO	High	IsMasterPr	AAEL00285	3:2334208	0	58	FALSO	26
-4,25E+18	FALSO	High	IsMasterPr	AAEL00918	2:9759733	0	58	FALSO	40
3,20E+18	FALSO	High	IsMasterPr	AAEL00542	1:2460189	0	72	FALSO	49
4,74E+18	FALSO	High	IsMasterPr	AAEL00613	3:6820580	0	44	FALSO	78
-1,33E+18	FALSO	High	IsMasterPr	AAEL01351	2:1897770	0	76	FALSO	14
5,07E+18	FALSO	High	IsMasterPr	AAEL00596	3:2276465	0	47	FALSO	27
-9,21E+18	FALSO	High	IsMasterPr	AAEL01940	3:9374927	0	65	FALSO	42
1,62E+18	FALSO	High	IsMasterPr	AAEL00526	2:6914971	0	62	FALSO	25
-4,34E+17	FALSO	High	IsMasterPr	AAEL00195	3:2275868	0	66	FALSO	29
-2,62E+18	FALSO	High	IsMasterPr	AAEL00275	3:1451843	0	89	FALSO	41
-9,33E+17	FALSO	High	IsMasterPr	AAEL00828	2:6725721	0	64	FALSO	8
5,54E+18	FALSO	High	IsMasterPr	AAEL00816	3:1007637	0	68	FALSO	32
5,30E+18	FALSO	High	IsMasterPr	AAEL00576	3:1147384	0	63	FALSO	27
-3,58E+17	FALSO	High	IsMasterPr	AAEL00276	3:1450204	0	74	FALSO	33
-1,04E+17	FALSO	High	IsMasterPr	AAEL00878	3:1326520	0	76	FALSO	39
-3,45E+18	FALSO	High	IsMasterPr	AAEL01491	1:2852695	0	54	FALSO	32
-5,99E+17	FALSO	High	IsMasterPr	AAEL00688	2:2564515	0	63	FALSO	18
2,77E+18	FALSO	High	IsMasterPr	AAEL01711	3:8891851	0	63	FALSO	38
1,33E+17	FALSO	High	IsMasterPr	AAEL00969	3:2098202	0	59	FALSO	59
-8,97E+18	FALSO	High	IsMasterPr	AAEL00276	3:1450204	0	81	FALSO	29
4,36E+18	FALSO	High	IsMasterPr	AAEL00692	3:3613157	0	48	FALSO	24
4,61E+18	FALSO	High	IsMasterPr	AAEL01150	2:8179530	0	70	FALSO	11

8,27E+18	FALSO	High	IsMasterPr	AAEL01711	3:8891851	0	63	FALSO	37
2,19E+18	FALSO	High	IsMasterPr	AAEL01014	2:2647456	0	65	FALSO	49
-6,37E+18	FALSO	High	IsMasterPr	AAEL00658	2:1326763	0	37	FALSO	51
3,53E+18	FALSO	High	IsMasterPr	AAEL00276	3:1450204	0	88	FALSO	30
-8,14E+18	FALSO	High	IsMasterPr	AAEL00612	3:6808466	0	47	FALSO	76
6,53E+18	FALSO	High	IsMasterPr	AAEL01815	2:2346918	0	44	FALSO	77
-5,12E+17	FALSO	High	IsMasterPr	AAEL00241	1:1770320	0	52	FALSO	37
-1,79E+18	FALSO	High	IsMasterPr	AAEL00672	2:8507242	0	41	FALSO	42
-8,53E+18	FALSO	High	IsMasterPr	AAEL00728	3:1955906	0	61	FALSO	50
4,52E+18	FALSO	High	IsMasterPr	AAEL00672	2:8507242	0	41	FALSO	40
-3,79E+17	FALSO	High	IsMasterPr	AAEL00728	3:1955920	0	67	FALSO	51
6,21E+18	FALSO	High	IsMasterPr	AAEL00816	3:9298266	0	59	FALSO	29
-5,33E+18	FALSO	High	IsMasterPr	AAEL00119	2:3075452	0	39	FALSO	67
-8,64E+18	FALSO	High	IsMasterPr	AAEL01476	2:8528965	0	40	FALSO	70
-2,91E+18	FALSO	High	IsMasterPr	AAEL02281	3:1953027	0	40	FALSO	57
-1,52E+18	FALSO	High	IsMasterPr	AAEL02281	3:1953027	0	40	FALSO	57
-2,86E+17	FALSO	High	IsMasterPr	AAEL00141	2:4582085	0	44	FALSO	71
-2,04E+17	FALSO	High	IsMasterPr	AAEL01043	3:6748994	0	35	FALSO	54
6,82E+17	FALSO	High	IsMasterPr	AAEL00576	3:1147384	0	59	FALSO	23
-8,59E+18	FALSO	High	IsMasterPr	AAEL02422	2:2993156	0	34	FALSO	16
-2,44E+18	FALSO	High	IsMasterPr	AAEL01158	3:9484602	0	63	FALSO	42
8,05E+18	FALSO	High	IsMasterPr	AAEL00161	1:1374243	0	55	FALSO	42
7,19E+18	FALSO	High	IsMasterPr	AAEL00730	3:1968064	0	56	FALSO	47
3,98E+18	FALSO	High	IsMasterPr	AAEL00429	2:2935027	0	47	FALSO	40
4,27E+18	FALSO	High	IsMasterPr	AAEL01170	2:1048302	0	49	FALSO	38
-9,02E+18	FALSO	High	IsMasterPr	AAEL00730	3:1968064	0	56	FALSO	47
2,84E+18	FALSO	High	IsMasterPr	AAEL01484	2:1047935	0	49	FALSO	38
9,07E+18	FALSO	High	IsMasterPr	AAEL01255	1:2941531	0	56	FALSO	32
8,20E+18	FALSO	High	IsMasterPr	AAEL01058	2:1119774	0	65	FALSO	46
5,43E+18	FALSO	High	IsMasterPr	AAEL00109	1:2992015	0	31	FALSO	21
7,53E+18	FALSO	High	IsMasterPr	AAEL02226	2:1773214	0	76	FALSO	9
-8,56E+18	FALSO	High	IsMasterPr	AAEL00688	2:2564515	0	62	FALSO	18
3,38E+18	FALSO	High	IsMasterPr	AAEL00341	3:3486673	0	73	FALSO	46
-6,15E+18	FALSO	High	IsMasterPr	AAEL00498	2:2818069	0	79	FALSO	29
-1,88E+18	FALSO	High	IsMasterPr	AAEL01345	1:2064241	0	43	FALSO	17
-6,10E+17	FALSO	High	IsMasterPr	AAEL00664	3:1484736	0	59	FALSO	23
8,70E+18	FALSO	High	IsMasterPr	AAEL00683	3:4367011	0	57	FALSO	34
-1,57E+18	FALSO	High	IsMasterPr	AAEL00607	1:1840121	0	84	FALSO	25
-2,02E+18	FALSO	High	IsMasterPr	AAEL01373	1:2376230	0	79	FALSO	23
-6,29E+18	FALSO	High	IsMasterPr	AAEL00374	1:2485031	0	65	FALSO	29
-6,27E+18	FALSO	High	IsMasterPr	AAEL01488	2:1218254	0	63	FALSO	31
6,83E+18	FALSO	High	IsMasterPr	AAEL01060	3:2916433	0	51	FALSO	26
-1,29E+18	FALSO	High	IsMasterPr	AAEL01828	3:2340417	0	16	FALSO	82
-6,59E+18	FALSO	High	IsMasterPr	AAEL01111	3:3791653	0	66	FALSO	23
-7,31E+18	FALSO	High	IsMasterPr	AAEL01224	1:1466210	0	64	FALSO	40
5,43E+16	FALSO	High	IsMasterPr	AAEL01046	1:2041436	0	40	FALSO	28
-8,21E+18	FALSO	High	IsMasterPr	AAEL01828	3:2340417	0	15	FALSO	81
-5,98E+18	FALSO	High	IsMasterPr	AAEL02113	NIGP01001	0	52	FALSO	32
8,95E+18	FALSO	High	IsMasterPr	AAEL00559	3:4043619	0	97	FALSO	29
4,60E+17	FALSO	High	IsMasterPr	AAEL01734	3:6861486	0	50	FALSO	31

8,65E+18	FALSO	High	IsMasterPr	AAEL00276	3:1450506	0	37	FALSO	24
8,39E+18	FALSO	High	IsMasterPr	AAEL01046	1:2041436	0	42	FALSO	29
5,15E+18	FALSO	High	IsMasterPr	AAEL01174	3:2911010	0	56	FALSO	25
5,41E+18	FALSO	High	IsMasterPr	AAEL00938	3:8353529	0	34	FALSO	39
5,56E+18	FALSO	High	IsMasterPr	AAEL00579	1:2002786	0	57	FALSO	21
-8,82E+18	FALSO	High	IsMasterPr	AAEL01174	3:2911010	0	56	FALSO	26
2,51E+18	FALSO	High	IsMasterPr	AAEL01353	2:2463703	0	57	FALSO	23
2,87E+18	FALSO	High	IsMasterPr	AAEL01178	3:2887793	0	56	FALSO	33
2,64E+18	FALSO	High	IsMasterPr	AAEL01531	2:1123064	0	71	FALSO	28
-5,98E+17	FALSO	High	IsMasterPr	AAEL01210	1:219922:2	0	47	FALSO	20
5,58E+18	FALSO	High	IsMasterPr	AAEL00070	2:4588232	0	51	FALSO	40
-4,15E+18	FALSO	High	IsMasterPr	AAEL02797	2:1789064	0	78	FALSO	37
5,18E+18	FALSO	High	IsMasterPr	AAEL02423	2:4880913	0	36	FALSO	56
-8,17E+18	FALSO	High	IsMasterPr	AAEL02569	3:2953881	0	50	FALSO	31
-5,24E+18	FALSO	High	IsMasterPr	AAEL02519	1:1831919	0	47	FALSO	41
2,76E+18	FALSO	High	IsMasterPr	AAEL00945	3:3212726	0	51	FALSO	26
6,12E+18	FALSO	High	IsMasterPr	AAEL02080	2:1187272	0	59	FALSO	20
4,95E+18	FALSO	High	IsMasterPr	AAEL00276	3:1450250	0	18	FALSO	20
-5,69E+18	FALSO	High	IsMasterPr	AAEL01000	1:1941878	0	55	FALSO	42
-3,16E+18	FALSO	High	IsMasterPr	AAEL00445	2:4733150	0	69	FALSO	15
5,13E+17	FALSO	High	IsMasterPr	AAEL00276	3:1450250	0	18	FALSO	20
1,77E+18	FALSO	High	IsMasterPr	AAEL00860	2:3630579	0	40	FALSO	51
-4,52E+18	FALSO	High	IsMasterPr	AAEL01351	2:3755340	0	71	FALSO	9
6,57E+18	FALSO	High	IsMasterPr	AAEL02322	1:9879340	0	54	FALSO	24
6,95E+18	FALSO	High	IsMasterPr	AAEL00699	3:1704967	0	56	FALSO	17
4,33E+18	FALSO	High	IsMasterPr	AAEL00860	2:3630579	0	41	FALSO	52
-5,99E+18	FALSO	High	IsMasterPr	AAEL01156	3:1070394	0	43	FALSO	41
1,26E+18	FALSO	High	IsMasterPr	AAEL02519	1:1831919	0	55	FALSO	37
-2,72E+16	FALSO	High	IsMasterPr	AAEL00860	2:3630579	0	41	FALSO	51
6,21E+18	FALSO	High	IsMasterPr	AAEL00902	3:1933122	0	71	FALSO	28
-6,81E+18	FALSO	High	IsMasterPr	AAEL02655	3:1072422	0	35	FALSO	52
3,89E+18	FALSO	High	IsMasterPr	AAEL00728	3:1959573	0	52	FALSO	32
-3,22E+18	FALSO	High	IsMasterPr	AAEL01081	3:5216822	0	51	FALSO	19
-2,30E+18	FALSO	High	IsMasterPr	AAEL00254	1:2285393	0	81	FALSO	20
6,13E+18	FALSO	High	IsMasterPr	AAEL00699	3:1705140	0	60	FALSO	18
-1,51E+18	FALSO	High	IsMasterPr	AAEL00064	3:3955989	0	66	FALSO	33
3,30E+18	FALSO	High	IsMasterPr	AAEL01973	1:5549868	0	50	FALSO	32
7,57E+18	FALSO	High	IsMasterPr	AAEL00683	3:5655071	0	45	FALSO	21
-2,03E+18	FALSO	High	IsMasterPr	AAEL02297	3:3820218	0	36	FALSO	41
5,86E+18	FALSO	High	IsMasterPr	AAEL02551	2:2907302	0	15	FALSO	55
-8,92E+18	FALSO	High	IsMasterPr	AAEL00311	2:1921683	0	62	FALSO	25
8,52E+18	FALSO	High	IsMasterPr	AAEL01156	3:1070394	0	42	FALSO	38
1,26E+18	FALSO	High	IsMasterPr	AAEL01445	2:1189369	0	53	FALSO	26
-1,73E+18	FALSO	High	IsMasterPr	AAEL00442	3:2067301	0	88	FALSO	15
-1,82E+18	FALSO	High	IsMasterPr	AAEL01952	1:3050714	0	35	FALSO	28
-2,63E+18	FALSO	High	IsMasterPr	AAEL02644	2:1550943	0	41	FALSO	22
2,82E+17	FALSO	High	IsMasterPr	AAEL01110	2:2320617	0	56	FALSO	26
6,57E+18	FALSO	High	IsMasterPr	AAEL02784	NIGP0100C	0	62	FALSO	9
-1,96E+18	FALSO	High	IsMasterPr	AAEL00209	3:1775356	0	69	FALSO	9
-7,56E+18	FALSO	High	IsMasterPr	AAEL02459	2:1919094	0	73	FALSO	28

5,46E+18	FALSO	High	IsMasterPr	AAEL02458	2:12725947	0	28	FALSO	29
-4,00E+18	FALSO	High	IsMasterPr	AAEL01945	2:63716732	0	19	FALSO	44
6,16E+18	FALSO	High	IsMasterPr	AAEL01748	1:24196281	0	49	FALSO	10
3,37E+18	FALSO	High	IsMasterPr	AAEL01340	2:15925407	0	67	FALSO	28
-1,22E+18	FALSO	High	IsMasterPr	AAEL01346	2:35114326	0	28	FALSO	48
7,22E+18	FALSO	High	IsMasterPr	AAEL01945	2:63716732	0	18	FALSO	42
3,06E+17	FALSO	High	IsMasterPr	AAEL01730	3:25270405	0	52	FALSO	23
-2,42E+18	FALSO	High	IsMasterPr	AAEL00239	2:30315371	0	50	FALSO	19
-7,17E+18	FALSO	High	IsMasterPr	AAEL00689	2:25671958	0	42	FALSO	28
-1,58E+18	FALSO	High	IsMasterPr	AAEL01343	3:15305111	0	44	FALSO	24
5,51E+18	FALSO	High	IsMasterPr	AAEL00276	1:23157740	0	37	FALSO	17
1,45E+17	FALSO	High	IsMasterPr	AAEL00454	1:14427509	0	46	FALSO	21
7,96E+18	FALSO	High	IsMasterPr	AAEL00075	3:24498991	0	34	FALSO	30
-2,68E+18	FALSO	High	IsMasterPr	AAEL00045	3:18623715	0	39	FALSO	18
-1,53E+18	FALSO	High	IsMasterPr	AAEL02297	3:38202189	0	35	FALSO	37
6,99E+18	FALSO	High	IsMasterPr	AAEL01299	3:29724215	0	63	FALSO	28
6,74E+18	FALSO	High	IsMasterPr	AAEL01232	3:31646898	0	91	FALSO	10
-3,24E+18	FALSO	High	IsMasterPr	AAEL00443	3:20435324	0	53	FALSO	27
-3,68E+18	FALSO	High	IsMasterPr	AAEL01082	3:51727775	0	48	FALSO	18
-8,83E+18	FALSO	High	IsMasterPr	AAEL01952	1:30507147	0	34	FALSO	27
7,75E+18	FALSO	High	IsMasterPr	AAEL00287	1:25531059	0	48	FALSO	24
-2,19E+18	FALSO	High	IsMasterPr	AAEL00854	1:63841030	0	44	FALSO	35
-5,30E+18	FALSO	High	IsMasterPr	AAEL01709	2:45576971	0	53	FALSO	22
3,93E+18	FALSO	High	IsMasterPr	AAEL02459	2:19190948	0	69	FALSO	24
1,45E+18	FALSO	High	IsMasterPr	AAEL00113	3:1644049[illegible]	0	41	FALSO	20
-7,81E+17	FALSO	High	IsMasterPr	AAEL01973	1:5666173[illegible]	0	43	FALSO	27
-5,16E+18	FALSO	High	IsMasterPr	AAEL00143	2:45686319	0	61	FALSO	28
5,79E+18	FALSO	High	IsMasterPr	AAEL02751	3:34203791	0	50	FALSO	7
-8,87E+18	FALSO	High	IsMasterPr	AAEL01978	2:69084521	0	65	FALSO	23
4,77E+18	FALSO	High	IsMasterPr	AAEL01729	3:15539585	0	60	FALSO	4
2,34E+18	FALSO	High	IsMasterPr	AAEL00877	2:19877010	0	18	FALSO	42
-5,95E+18	FALSO	High	IsMasterPr	AAEL00674	2:84905797	0	42	FALSO	15
8,30E+18	FALSO	High	IsMasterPr	AAEL00987	1:92328868	0	35	FALSO	23
-8,09E+18	FALSO	High	IsMasterPr	AAEL01187	2:18727898	0	41	FALSO	8
-7,63E+18	FALSO	High	IsMasterPr	AAEL01031	3:40968851	0	57	FALSO	27
-8,39E+18	FALSO	High	IsMasterPr	AAEL00159	2:21107127	0	49	FALSO	25
-4,90E+18	FALSO	High	IsMasterPr	AAEL01545	1:22490674	0	42	FALSO	27
-1,80E+18	FALSO	High	IsMasterPr	AAEL02445	NIGP0100C	0	62	FALSO	17
-1,14E+18	FALSO	High	IsMasterPr	AAEL00505	1:29463225	0	43	FALSO	18
-6,63E+18	FALSO	High	IsMasterPr	AAEL00059	2:40753841	0	39	FALSO	41
-2,94E+18	FALSO	High	IsMasterPr	AAEL00450	2:45315289	0	39	FALSO	27
4,07E+18	FALSO	High	IsMasterPr	AAEL02606	2:14107031	0	26	FALSO	43
5,05E+18	FALSO	High	IsMasterPr	AAEL00934	2:40485150	0	96	FALSO	24
8,35E+18	FALSO	High	IsMasterPr	AAEL01476	2:85289651	0	40	FALSO	31
7,91E+18	FALSO	High	IsMasterPr	AAEL00476	2:28839384	0	74	FALSO	11
-4,75E+18	FALSO	High	IsMasterPr	AAEL01327	2:94717898	0	71	FALSO	17
-5,16E+18	FALSO	High	IsMasterPr	AAEL00281	1:30448860	0	69	FALSO	14
-6,96E+17	FALSO	High	IsMasterPr	AAEL01511	2:19933901	0	66	FALSO	29
-3,84E+18	FALSO	High	IsMasterPr	AAEL01198	2:20903020	0	31	FALSO	22
3,97E+18	FALSO	High	IsMasterPr	AAEL00468	1:14585409	0	60	FALSO	29

-6,65E+18	FALSO	High	IsMasterPr	AAEL02532	1:2954940	0	66	FALSO	21
-1,84E+18	FALSO	High	IsMasterPr	AAEL0102C	1:4365207	0	80	FALSO	12
-4,22E+18	FALSO	High	IsMasterPr	AAEL0226C	2:1312176	0	25	FALSO	20
8,63E+18	FALSO	High	IsMasterPr	AAEL0139C	2:9650907	0	73	FALSO	19
3,62E+18	FALSO	High	IsMasterPr	AAEL00188	2:3893455	0	34	FALSO	14
5,32E+17	FALSO	High	IsMasterPr	AAEL00433	3:3656749	0	46	FALSO	14
3,47E+18	FALSO	High	IsMasterPr	AAEL01295	3:2067609	0	52	FALSO	21
-3,40E+18	FALSO	High	IsMasterPr	AAEL0083C	1:9617334	0	67	FALSO	12
7,08E+18	FALSO	High	IsMasterPr	AAEL0129C	2:2518472	0	63	FALSO	24
7,65E+18	FALSO	High	IsMasterPr	AAEL00587	2:6633799	0	48	FALSO	26
5,47E+18	FALSO	High	IsMasterPr	AAEL00517	2:3218675	0	48	FALSO	13
-4,96E+18	FALSO	High	IsMasterPr	AAEL01395	2:3320070	0	71	FALSO	22
-7,97E+18	FALSO	High	IsMasterPr	AAEL01395	2:3320064	0	67	FALSO	22
-7,36E+18	FALSO	High	IsMasterPr	AAEL00704	3:2998072	0	36	FALSO	21
5,66E+18	FALSO	High	IsMasterPr	AAEL02045	2:6346905	0	13	FALSO	41
5,98E+18	FALSO	High	IsMasterPr	AAEL0173C	3:3472765	0	76	FALSO	16
3,61E+17	FALSO	High	IsMasterPr	AAEL0226C	2:1312176	0	14	FALSO	20
-1,40E+18	FALSO	High	IsMasterPr	AAEL00288	1:1082019	0	52	FALSO	24
8,46E+18	FALSO	High	IsMasterPr	AAEL01014	2:2647915	0	38	FALSO	17
4,59E+18	FALSO	High	IsMasterPr	AAEL00765	1:1349660	0	63	FALSO	21
6,59E+18	FALSO	High	IsMasterPr	AAEL00599	3:1547320	0	79	FALSO	17
-7,99E+18	FALSO	High	IsMasterPr	AAEL01989	2:3263366	0	23	FALSO	9
5,38E+18	FALSO	High	IsMasterPr	AAEL0122C	1:1609095	0	58	FALSO	10
6,58E+18	FALSO	High	IsMasterPr	AAEL0290C	3:2352010	0	70	FALSO	23
4,07E+18	FALSO	High	IsMasterPr	AAEL00246	3:3383191	0	56	FALSO	22
-2,71E+18	FALSO	High	IsMasterPr	AAEL02538	NIGP01001	0	59	FALSO	16
-5,13E+17	FALSO	High	IsMasterPr	AAEL00702	2:2419996	0	40	FALSO	26
-1,00E+18	FALSO	High	IsMasterPr	AAEL00167	2:8596618	0	77	FALSO	14
-7,33E+18	FALSO	High	IsMasterPr	AAEL00109	1:2816203	0	62	FALSO	16
8,64E+18	FALSO	High	IsMasterPr	AAEL0104C	1:2681453	0	63	FALSO	32
3,86E+18	FALSO	High	IsMasterPr	AAEL01176	2:1686855	0	60	FALSO	26
7,88E+18	FALSO	High	IsMasterPr	AAEL00617	3:1407321	0	49	FALSO	17
-3,62E+17	FALSO	High	IsMasterPr	AAEL01174	3:2910590	0	66	FALSO	17
5,67E+18	FALSO	High	IsMasterPr	AAEL00688	2:2447691	0	51	FALSO	23
-1,50E+18	FALSO	High	IsMasterPr	AAEL0208C	2:2414696	0	45	FALSO	38
-6,00E+18	FALSO	High	IsMasterPr	AAEL00387	3:8526269	0	41	FALSO	28
5,56E+18	FALSO	High	IsMasterPr	AAEL00884	2:2413287	0	49	FALSO	20
8,22E+18	FALSO	High	IsMasterPr	AAEL0077C	2:4703851	0	50	FALSO	17
6,80E+18	FALSO	High	IsMasterPr	AAEL0105C	2:9073890	0	61	FALSO	16
-4,97E+18	FALSO	High	IsMasterPr	AAEL0290C	3:3325031	0	73	FALSO	14
7,44E+18	FALSO	High	IsMasterPr	AAEL0105C	2:9073890	0	37	FALSO	16
-8,84E+17	FALSO	High	IsMasterPr	AAEL00884	2:2411917	0	61	FALSO	13
3,39E+16	FALSO	High	IsMasterPr	AAEL01458	1:3043970	0	85	FALSO	15
-5,55E+18	FALSO	High	IsMasterPr	AAEL00408	3:4016718	0	67	FALSO	13
-9,21E+18	FALSO	High	IsMasterPr	AAEL00755	2:3269330	0	42	FALSO	12
3,61E+18	FALSO	High	IsMasterPr	AAEL0290C	3:3325031	0	80	FALSO	16
1,68E+17	FALSO	High	IsMasterPr	AAEL02793	1:2614251	0	32	FALSO	18
9,26E+16	FALSO	High	IsMasterPr	AAEL01138	1:1221047	0	39	FALSO	13
-8,17E+18	FALSO	High	IsMasterPr	AAEL01361	3:1950298	0	44	FALSO	21
3,76E+18	FALSO	High	IsMasterPr	AAEL00382	3:3427534	0	31	FALSO	26

-4,18E+18	FALSO	High	IsMasterPr	AAEL0104	1:2681453	0	62	FALSO	31
4,22E+18	FALSO	High	IsMasterPr	AAEL01175	1:1475106	0	49	FALSO	7
-4,03E+18	FALSO	High	IsMasterPr	AAEL02727	1:2367907	0	43	FALSO	27
8,75E+18	FALSO	High	IsMasterPr	AAEL00894	1:8064222	0	33	FALSO	30
6,46E+18	FALSO	High	IsMasterPr	AAEL02484	3:3417219	0	27	FALSO	15
-1,21E+18	FALSO	High	IsMasterPr	AAEL00158	2:2283430	0	68	FALSO	23
-2,43E+18	FALSO	High	IsMasterPr	AAEL01972	2:2128669	0	20	FALSO	35
-6,88E+18	FALSO	High	IsMasterPr	AAEL00617	3:1407321	0	49	FALSO	17
-8,92E+18	FALSO	High	IsMasterPr	AAEL00942	2:1518331	0	37	FALSO	21
8,85E+18	FALSO	High	IsMasterPr	AAEL01241	2:1330599	0	34	FALSO	14
7,88E+18	FALSO	High	IsMasterPr	AAEL0146	3:3139193	0	60	FALSO	17
8,83E+18	FALSO	High	IsMasterPr	AAEL00657	2:1332631	0	32	FALSO	6
-6,47E+18	FALSO	High	IsMasterPr	AAEL00108	1:2994202	0	78	FALSO	12
2,09E+18	FALSO	High	IsMasterPr	AAEL00717	2:1499707	0	82	FALSO	19
3,68E+18	FALSO	High	IsMasterPr	AAEL00455	1:1452103	0	50	FALSO	8
-1,94E+18	FALSO	High	IsMasterPr	AAEL00508	1:2947351	0	50	FALSO	19
-9,05E+18	FALSO	High	IsMasterPr	AAEL02818	1:1461129	0	44	FALSO	16
8,42E+17	FALSO	High	IsMasterPr	AAEL0113	3:1402708	0	54	FALSO	16
-8,93E+18	FALSO	High	IsMasterPr	AAEL00367	2:3165969	0	47	FALSO	10
9,20E+18	FALSO	High	IsMasterPr	AAEL00408	3:4016722	0	67	FALSO	13
-6,05E+18	FALSO	High	IsMasterPr	AAEL01821	3:1924564	0	12	FALSO	34
-3,58E+18	FALSO	High	IsMasterPr	AAEL02179	1:3481120	0	77	FALSO	14
-2,09E+18	FALSO	High	IsMasterPr	AAEL00455	1:1452103	0	50	FALSO	8
6,80E+18	FALSO	High	IsMasterPr	AAEL00687	3:3693605	0	49	FALSO	11
2,40E+16	FALSO	High	IsMasterPr	AAEL00754	2:3272812	0	64	FALSO	12
2,53E+18	FALSO	High	IsMasterPr	AAEL00485	2:1152844	0	41	FALSO	18
4,00E+18	FALSO	High	IsMasterPr	AAEL01177	3:3768645	0	29	FALSO	19
-6,11E+18	FALSO	High	IsMasterPr	AAEL00716	3:1915318	0	62	FALSO	17
-5,02E+18	FALSO	High	IsMasterPr	AAEL0012	2:4133383	0	40	FALSO	22
-4,78E+18	FALSO	High	IsMasterPr	AAEL02727	1:2367907	0	33	FALSO	22
-1,25E+18	FALSO	High	IsMasterPr	AAEL00663	3:2497212	0	55	FALSO	15
-7,93E+18	FALSO	High	IsMasterPr	AAEL00032	2:3688444	0	47	FALSO	14
2,98E+18	FALSO	High	IsMasterPr	AAEL00816	3:9231475	0	59	FALSO	23
5,50E+18	FALSO	High	IsMasterPr	AAEL01069	2:1763169	0	36	FALSO	13
-8,38E+18	FALSO	High	IsMasterPr	AAEL00572	2:4373066	0	38	FALSO	20
-2,52E+18	FALSO	High	IsMasterPr	AAEL00365	3:6534692	0	18	FALSO	20
8,38E+18	FALSO	High	IsMasterPr	AAEL02541	2:4741588	0	10	FALSO	33
2,34E+18	FALSO	High	IsMasterPr	AAEL00387	3:8514752	0	69	FALSO	16
4,79E+18	FALSO	High	IsMasterPr	AAEL00229	3:3740958	0	47	FALSO	16
5,29E+18	FALSO	High	IsMasterPr	AAEL01052	2:2618307	0	26	FALSO	15
1,32E+18	FALSO	High	IsMasterPr	AAEL01815	2:2298711	0	32	FALSO	22
-2,42E+18	FALSO	High	IsMasterPr	AAEL0105	2:9098924	0	53	FALSO	18
8,43E+17	FALSO	High	IsMasterPr	AAEL00533	2:2712106	0	54	FALSO	19
-6,45E+18	FALSO	High	IsMasterPr	AAEL02174	3:1249204	0	27	FALSO	19
-6,15E+18	FALSO	High	IsMasterPr	AAEL02136	1:9580840	0	41	FALSO	12
9,00E+18	FALSO	High	IsMasterPr	AAEL01176	2:1686631	0	45	FALSO	19
8,00E+17	FALSO	High	IsMasterPr	AAEL0113	3:1402774	0	56	FALSO	14
-8,58E+18	FALSO	High	IsMasterPr	AAEL00223	2:4291708	0	60	FALSO	3
6,34E+18	FALSO	High	IsMasterPr	AAEL00697	2:3987869	0	36	FALSO	16
3,02E+18	FALSO	High	IsMasterPr	AAEL0092	3:3909777	0	42	FALSO	5

-1,51E+18	FALSO	High	IsMasterPr	AAEL00717	2:1499707	0	44	FALSO	18
4,90E+17	FALSO	High	IsMasterPr	AAEL0002	2:7536525	0	35	FALSO	23
-2,56E+18	FALSO	High	IsMasterPr	AAEL00813	3:1080446	0	44	FALSO	18
-2,54E+18	FALSO	High	IsMasterPr	AAEL01282	1:2425762	0	42	FALSO	28
3,10E+18	FALSO	High	IsMasterPr	AAEL0086	2:3214998	0	40	FALSO	7
9,50E+17	FALSO	High	IsMasterPr	AAEL0194	3:4017524	0	61	FALSO	13
4,31E+18	FALSO	High	IsMasterPr	AAEL01384	1:1712103	0	19	FALSO	15
6,13E+18	FALSO	High	IsMasterPr	AAEL00551	1:8733148	0	58	FALSO	15
-5,26E+17	FALSO	High	IsMasterPr	AAEL0138	3:2019089	0	21	FALSO	11
-4,08E+16	FALSO	High	IsMasterPr	AAEL0196	2:1362433	0	43	FALSO	17
-8,03E+18	FALSO	High	IsMasterPr	AAEL00851	2:9941906	0	57	FALSO	20
-1,35E+18	FALSO	High	IsMasterPr	AAEL00751	1:1980647	0	53	FALSO	10
-4,14E+18	FALSO	High	IsMasterPr	AAEL01493	2:1451045	0	83	FALSO	10
-2,77E+18	FALSO	High	IsMasterPr	AAEL0248	NIGP0100	0	23	FALSO	14
-5,96E+18	FALSO	High	IsMasterPr	AAEL0099	3:2868385	0	16	FALSO	32
-3,47E+18	FALSO	High	IsMasterPr	AAEL02492	2:9929198	0	36	FALSO	10
-6,00E+18	FALSO	High	IsMasterPr	AAEL0105	2:9098924	0	49	FALSO	15
-4,33E+18	FALSO	High	IsMasterPr	AAEL01954	1:8181610	0	24	FALSO	17
-2,08E+17	FALSO	High	IsMasterPr	AAEL00941	3:3380257	0	48	FALSO	17
5,68E+18	FALSO	High	IsMasterPr	AAEL01993	3:2653606	0	53	FALSO	7
-1,47E+18	FALSO	High	IsMasterPr	AAEL0042	2:2930486	0	39	FALSO	17
1,88E+18	FALSO	High	IsMasterPr	AAEL00516	1:1407650	0	39	FALSO	15
-3,89E+18	FALSO	High	IsMasterPr	AAEL00168	2:8600542	0	73	FALSO	10
-6,75E+18	FALSO	High	IsMasterPr	AAEL01003	2:1107974	0	38	FALSO	17
-6,75E+18	FALSO	High	IsMasterPr	AAEL00147	2:2616936	0	21	FALSO	23
6,86E+18	FALSO	High	IsMasterPr	AAEL00576	1:1219608	0	17	FALSO	1
-3,10E+18	FALSO	High	IsMasterPr	AAEL00561	3:1601248	0	46	FALSO	20
1,01E+18	FALSO	High	IsMasterPr	AAEL01374	2:9573912	0	41	FALSO	9
1,86E+18	FALSO	High	IsMasterPr	AAEL0081	3:1488193	0	43	FALSO	12
9,17E+18	FALSO	High	IsMasterPr	AAEL00703	1:1279445	0	69	FALSO	16
-6,25E+18	FALSO	High	IsMasterPr	AAEL00503	2:4443094	0	33	FALSO	14
-4,47E+18	FALSO	High	IsMasterPr	AAEL00867	2:2448850	0	31	FALSO	12
-1,53E+18	FALSO	High	IsMasterPr	AAEL0042	2:2930486	0	38	FALSO	16
-6,91E+16	FALSO	High	IsMasterPr	AAEL0134	2:1592540	0	64	FALSO	23
5,93E+18	FALSO	High	IsMasterPr	AAEL00518	2:313244:3	0	19	FALSO	21
8,02E+18	FALSO	High	IsMasterPr	AAEL00142	2:4632062	0	24	FALSO	22
5,72E+18	FALSO	High	IsMasterPr	AAEL01972	2:2129025	0	17	FALSO	27
6,71E+18	FALSO	High	IsMasterPr	AAEL01307	2:2535511	0	18	FALSO	13
-2,02E+18	FALSO	High	IsMasterPr	AAEL00763	3:1367666	0	36	FALSO	17
6,48E+17	FALSO	High	IsMasterPr	AAEL0232	NIGP01002	0	15	FALSO	19
7,69E+17	FALSO	High	IsMasterPr	AAEL00121	2:4114805	0	30	FALSO	23
1,13E+18	FALSO	High	IsMasterPr	AAEL01732	3:3472953	0	57	FALSO	12
8,17E+18	FALSO	High	IsMasterPr	AAEL01977	2:2321006	0	31	FALSO	18
-2,41E+18	FALSO	High	IsMasterPr	AAEL0140	1:2386156	0	43	FALSO	18
-2,61E+18	FALSO	High	IsMasterPr	AAEL00594	1:2574585	0	32	FALSO	11
6,68E+18	FALSO	High	IsMasterPr	AAEL0039	3:2540176	0	41	FALSO	15
-1,81E+18	FALSO	High	IsMasterPr	AAEL0073	1:2598036	0	53	FALSO	16
7,48E+18	FALSO	High	IsMasterPr	AAEL01382	1:2634911	0	35	FALSO	14
-2,37E+18	FALSO	High	IsMasterPr	AAEL0224	2:688567:7	0	48	FALSO	17
-9,40E+17	FALSO	High	IsMasterPr	AAEL0054	1:2471224	0	49	FALSO	12

3,65E+18	FALSO	High	IsMasterPr	AAEL01247	2:2189522	0	29	FALSO	12
4,69E+18	FALSO	High	IsMasterPr	AAEL0023C	3:3741608	0	57	FALSO	14
-3,71E+18	FALSO	High	IsMasterPr	AAEL00878	3:1325538	0	76	FALSO	18
-5,07E+18	FALSO	High	IsMasterPr	AAEL01128	1:1501232	0	46	FALSO	20
4,81E+18	FALSO	High	IsMasterPr	AAEL00367	3:6572711	0	34	FALSO	17
8,16E+18	FALSO	High	IsMasterPr	AAEL01511	NIGP0100C	0	70	FALSO	12
-6,59E+18	FALSO	High	IsMasterPr	AAEL00694	3:2795768	0	42	FALSO	15
-4,83E+18	FALSO	High	IsMasterPr	AAEL01335	2:2404308	0	65	FALSO	7
-6,34E+18	FALSO	High	IsMasterPr	AAEL00864	2:2149399	0	43	FALSO	16
5,57E+18	FALSO	High	IsMasterPr	AAEL0061C	3:3074169	0	57	FALSO	19
8,76E+18	FALSO	High	IsMasterPr	AAEL01731	3:3468090	0	32	FALSO	20
8,78E+18	FALSO	High	IsMasterPr	AAEL02818	1:3073216	0	14	FALSO	19
-7,78E+18	FALSO	High	IsMasterPr	AAEL01282	1:2425501	0	39	FALSO	21
-4,56E+18	FALSO	High	IsMasterPr	AAEL01257	1:2852437	0	40	FALSO	12
3,35E+18	FALSO	High	IsMasterPr	AAEL00402	3:1663721	0	47	FALSO	15
8,87E+18	FALSO	High	IsMasterPr	AAEL0110S	2:2321488	0	46	FALSO	14
-7,59E+18	FALSO	High	IsMasterPr	AAEL00718	2:1499497	0	65	FALSO	13
4,50E+18	FALSO	High	IsMasterPr	AAEL00196	2:4255327	0	39	FALSO	16
4,96E+18	FALSO	High	IsMasterPr	AAEL00087	3:3324840	0	53	FALSO	11
4,66E+18	FALSO	High	IsMasterPr	AAEL0030C	3:4007360	0	40	FALSO	8
-5,20E+17	FALSO	High	IsMasterPr	AAEL01082	3:5204930	0	45	FALSO	12
3,79E+18	FALSO	High	IsMasterPr	AAEL01274	3:3221304	0	37	FALSO	21
-8,09E+17	FALSO	High	IsMasterPr	AAEL00893	3:3562736	0	44	FALSO	6
-6,50E+18	FALSO	High	IsMasterPr	AAEL00484	2:1154986	0	22	FALSO	15
5,79E+18	FALSO	High	IsMasterPr	AAEL0031S	1:1921331	0	44	FALSO	13
3,52E+18	FALSO	High	IsMasterPr	AAEL00583	1:2258578	0	44	FALSO	11
4,51E+17	FALSO	High	IsMasterPr	AAEL00868	2:3214258	0	51	FALSO	13
6,37E+18	FALSO	High	IsMasterPr	AAEL0112C	1:3447105	0	35	FALSO	14
-8,88E+18	FALSO	High	IsMasterPr	AAEL00042	3:1852085	0	43	FALSO	16
6,97E+18	FALSO	High	IsMasterPr	AAEL00938	3:3809134	0	42	FALSO	11
4,60E+18	FALSO	High	IsMasterPr	AAEL0010C	1:2100612	0	36	FALSO	10
-2,17E+18	FALSO	High	IsMasterPr	AAEL00106	1:2998129	0	39	FALSO	8
-4,79E+18	FALSO	High	IsMasterPr	AAEL00702	1:1281471	0	46	FALSO	15
-3,78E+18	FALSO	High	IsMasterPr	AAEL01203	3:1472806	0	57	FALSO	13
5,84E+18	FALSO	High	IsMasterPr	AAEL02777	3:6977538	0	35	FALSO	5
7,12E+18	FALSO	High	IsMasterPr	AAEL00268	2:4649129	0	42	FALSO	15
-5,16E+18	FALSO	High	IsMasterPr	AAEL01971	2:3515088	0	16	FALSO	16
9,01E+18	FALSO	High	IsMasterPr	AAEL01945	3:4046191	0	18	FALSO	16
4,49E+18	FALSO	High	IsMasterPr	AAEL0057S	1:1218728	0	29	FALSO	14
7,62E+18	FALSO	High	IsMasterPr	AAEL01713	2:2298950	0	51	FALSO	8
-7,01E+18	FALSO	High	IsMasterPr	AAEL00208	2:1199720	0	39	FALSO	17
-7,91E+18	FALSO	High	IsMasterPr	AAEL00186	2:2768899	0	38	FALSO	17
-3,95E+18	FALSO	High	IsMasterPr	AAEL0007S	3:2428852	0	74	FALSO	4
7,95E+18	FALSO	High	IsMasterPr	AAEL0056S	2:3361713	0	19	FALSO	17
-5,42E+18	FALSO	High	IsMasterPr	AAEL01165	3:2168830	0	25	FALSO	18
-8,57E+17	FALSO	High	IsMasterPr	AAEL0234S	1:1113622	0	64	FALSO	13
-3,68E+18	FALSO	High	IsMasterPr	AAEL00284	3:2331842	0	23	FALSO	10
-3,23E+18	FALSO	High	IsMasterPr	AAEL00474	2:2885506	0	30	FALSO	6
-1,42E+18	FALSO	High	IsMasterPr	AAEL01366	1:1550732	0	53	FALSO	12
-1,53E+18	FALSO	High	IsMasterPr	AAEL00085	3:3620276	0	50	FALSO	13

-3,73E+18	FALSO	High	IsMasterPr	AAEL00517	2:1307660	0	68	FALSO	7
6,62E+18	FALSO	High	IsMasterPr	AAEL02211	1:2183687	0	18	FALSO	15
-1,71E+18	FALSO	High	IsMasterPr	AAEL00044	3:1834737	0	16	FALSO	12
-6,86E+18	FALSO	High	IsMasterPr	AAEL01995	3:4059264	0	25	FALSO	16
-6,29E+18	FALSO	High	IsMasterPr	AAEL00668	2:3262764	0	52	FALSO	17
8,43E+18	FALSO	High	IsMasterPr	AAEL01948	3:5064409	0	34	FALSO	18
-8,17E+18	FALSO	High	IsMasterPr	AAEL02293	3:3411401	0	38	FALSO	10
1,38E+18	FALSO	High	IsMasterPr	AAEL02727	1:2367907	0	29	FALSO	18
-2,29E+18	FALSO	High	IsMasterPr	AAEL0031	1:1921331	0	40	FALSO	13
-8,60E+18	FALSO	High	IsMasterPr	AAEL01085	1:5468388	0	47	FALSO	12
4,69E+18	FALSO	High	IsMasterPr	AAEL00124	2:1540889	0	33	FALSO	7
-7,99E+18	FALSO	High	IsMasterPr	AAEL00106	1:2998129	0	68	FALSO	10
-8,27E+18	FALSO	High	IsMasterPr	AAEL00008	2:1844662	0	29	FALSO	24
-9,13E+18	FALSO	High	IsMasterPr	AAEL01235	3:2011423	0	49	FALSO	9
-7,31E+18	FALSO	High	IsMasterPr	AAEL0175	2:8610365	0	57	FALSO	4
8,73E+18	FALSO	High	IsMasterPr	AAEL0150	2:3377253	0	64	FALSO	16
-9,12E+18	FALSO	High	IsMasterPr	AAEL01085	1:5468388	0	42	FALSO	15
-7,15E+17	FALSO	High	IsMasterPr	AAEL01127	2:2263137	0	38	FALSO	19
-9,34E+17	FALSO	High	IsMasterPr	AAEL00713	3:6986880	0	11	FALSO	29
-8,45E+18	FALSO	High	IsMasterPr	AAEL00318	1:1902518	0	42	FALSO	8
-6,71E+18	FALSO	High	IsMasterPr	AAEL00023	1:6644118	0	20	FALSO	14
8,79E+18	FALSO	High	IsMasterPr	AAEL00093	3:3770811	0	64	FALSO	8
6,70E+18	FALSO	High	IsMasterPr	AAEL0134	2:1992592	0	36	FALSO	16
6,32E+18	FALSO	High	IsMasterPr	AAEL01078	2:3048274	0	24	FALSO	8
-4,48E+17	FALSO	High	IsMasterPr	AAEL01953	2:2457884	0	27	FALSO	12
-8,95E+18	FALSO	High	IsMasterPr	A0A060PY	ATP syntha	0	15	FALSO	6
8,85E+18	FALSO	High	IsMasterPr	AAEL00404	3:1662530	0	46	FALSO	14
-7,18E+18	FALSO	High	IsMasterPr	AAEL01206	3:1608024	0	58	FALSO	13
8,99E+18	FALSO	High	IsMasterPr	AAEL00996	2:9310567	0	59	FALSO	7
-1,49E+18	FALSO	High	IsMasterPr	AAEL00093	3:3770811	0	75	FALSO	10
-3,68E+18	FALSO	High	IsMasterPr	AAEL00417	2:1473460	0	60	FALSO	11
-1,52E+18	FALSO	High	IsMasterPr	AAEL00093	3:3770811	0	72	FALSO	10
-7,80E+18	FALSO	High	IsMasterPr	AAEL02011	3:3152334	0	56	FALSO	16
8,15E+18	FALSO	High	IsMasterPr	AAEL01033	2:2876369	0	45	FALSO	15
-2,78E+18	FALSO	High	IsMasterPr	AAEL01335	2:2404861	0	38	FALSO	13
-2,34E+18	FALSO	High	IsMasterPr	AAEL00674	2:8477531	0	41	FALSO	12
-5,79E+16	FALSO	High	IsMasterPr	AAEL01333	2:1356903	0	65	FALSO	9
3,66E+18	FALSO	High	IsMasterPr	AAEL0013	2:2058123	0	75	FALSO	7
4,67E+18	FALSO	High	IsMasterPr	AAEL00424	2:1186696	0	60	FALSO	10
4,68E+18	FALSO	High	IsMasterPr	AAEL00453	2:3101784	0	61	FALSO	14
-5,47E+18	FALSO	High	IsMasterPr	AAEL01155	3:3128470	0	24	FALSO	8
-9,16E+18	FALSO	High	IsMasterPr	AAEL01084	2:1276760	0	58	FALSO	15
-9,04E+18	FALSO	High	IsMasterPr	AAEL00768	2:2508046	0	26	FALSO	11
2,83E+17	FALSO	High	IsMasterPr	AAEL00848	2:1898152	0	32	FALSO	11
-7,66E+18	FALSO	High	IsMasterPr	AAEL0099	1:1932537	0	39	FALSO	14
3,85E+18	FALSO	High	IsMasterPr	AAEL00473	2:2903508	0	34	FALSO	15
-1,64E+17	FALSO	High	IsMasterPr	AAEL0195	3:2879628	0	14	FALSO	15
1,56E+18	FALSO	High	IsMasterPr	AAEL00746	1:4593603	0	7	FALSO	17
-7,12E+18	FALSO	High	IsMasterPr	AAEL0044	2:4531393	0	50	FALSO	15
-1,76E+18	FALSO	High	IsMasterPr	AAEL00561	3:1601886	0	40	FALSO	15

6,23E+18	FALSO	High	IsMasterPr	AAEL00111	3:4065087	0	30	FALSO	15
2,87E+18	FALSO	High	IsMasterPr	AAEL00788	3:3821928	0	64	FALSO	6
1,20E+18	FALSO	High	IsMasterPr	AAEL0085E	2:1572191	0	53	FALSO	11
-4,42E+17	FALSO	High	IsMasterPr	AAEL01013	2:1620885	0	81	FALSO	7
-4,41E+18	FALSO	High	IsMasterPr	AAEL02458	2:2885141	0	32	FALSO	6
1,73E+17	FALSO	High	IsMasterPr	AAEL0040E	1:1354815	0	56	FALSO	13
-2,01E+18	FALSO	High	IsMasterPr	AAEL01213	3:2980809	0	37	FALSO	7
-8,83E+18	FALSO	High	IsMasterPr	AAEL0053C	3:3093724	0	26	FALSO	14
7,65E+18	FALSO	High	IsMasterPr	AAEL00975	3:2844844	0	53	FALSO	17
-3,76E+18	FALSO	High	IsMasterPr	AAEL00105	3:5521880	0	79	FALSO	8
7,78E+18	FALSO	High	IsMasterPr	AAEL0102S	2:3411953	0	35	FALSO	14
3,82E+18	FALSO	High	IsMasterPr	AAEL00983	3:1899038	0	20	FALSO	20
8,38E+18	FALSO	High	IsMasterPr	AAEL00675	2:8476751	0	39	FALSO	16
-2,30E+18	FALSO	High	IsMasterPr	AAEL00185	2:2745426	0	38	FALSO	18
8,65E+18	FALSO	High	IsMasterPr	AAEL0007C	2:4589742	0	30	FALSO	14
3,02E+18	FALSO	High	IsMasterPr	AAEL01208	3:1607094	0	40	FALSO	12
-4,65E+18	FALSO	High	IsMasterPr	AAEL01088	1:2180242	0	38	FALSO	15
1,09E+18	FALSO	High	IsMasterPr	AAEL02635	3:3371849	0	14	FALSO	15
3,69E+18	FALSO	High	IsMasterPr	AAEL01273	2:1623442	0	37	FALSO	11
5,37E+18	FALSO	High	IsMasterPr	AAEL0194S	3:1072856	0	10	FALSO	14
-1,28E+18	FALSO	High	IsMasterPr	AAEL01953	2:2457884	0	28	FALSO	11
5,41E+18	FALSO	High	IsMasterPr	AAEL01978	3:8471900	0	11	FALSO	21
-4,03E+18	FALSO	High	IsMasterPr	AAEL00493	2:2597265	0	49	FALSO	11
6,82E+17	FALSO	High	IsMasterPr	AAEL01211	1:198880:2	0	24	FALSO	14
5,19E+18	FALSO	High	IsMasterPr	AAEL0232E	1:1474214	0	17	FALSO	11
4,78E+17	FALSO	High	IsMasterPr	AAEL0255S	3:1966815	0	35	FALSO	11
1,52E+18	FALSO	High	IsMasterPr	AAEL00417	2:1473460	0	41	FALSO	9
9,00E+18	FALSO	High	IsMasterPr	AAEL0195C	3:2882979	0	13	FALSO	14
-6,20E+18	FALSO	High	IsMasterPr	AAEL0139S	2:1018258	0	17	FALSO	6
-3,67E+18	FALSO	High	IsMasterPr	AAEL0230E	1:2357370	0	54	FALSO	15
5,00E+18	FALSO	High	IsMasterPr	AAEL01393	2:3058310	0	31	FALSO	14
-5,07E+18	FALSO	High	IsMasterPr	AAEL00112	3:1707980	0	42	FALSO	13
6,58E+18	FALSO	High	IsMasterPr	AAEL02705	2:1561905	0	75	FALSO	10
-5,02E+18	FALSO	High	IsMasterPr	AAEL00374	1:2478678	0	23	FALSO	13
-3,26E+18	FALSO	High	IsMasterPr	AAEL01407	2:1323197	0	50	FALSO	14
5,17E+18	FALSO	High	IsMasterPr	AAEL02038	2:1246459	0	31	FALSO	14
8,92E+18	FALSO	High	IsMasterPr	AAEL00743	2:2913837	0	69	FALSO	11
-2,00E+17	FALSO	High	IsMasterPr	AAEL00301	3:3999212	0	48	FALSO	7
8,11E+18	FALSO	High	IsMasterPr	AAEL02284	2:1775343	0	30	FALSO	4
-6,39E+18	FALSO	High	IsMasterPr	AAEL0148E	2:1557703	0	15	FALSO	9
8,71E+18	FALSO	High	IsMasterPr	AAEL0236C	1:4291674	0	62	FALSO	8
1,27E+18	FALSO	High	IsMasterPr	AAEL01065	2:1357879	0	45	FALSO	6
-6,54E+18	FALSO	High	IsMasterPr	AAEL0094S	3:3448718	0	46	FALSO	7
-5,62E+18	FALSO	High	IsMasterPr	AAEL0105C	2:4462487	0	41	FALSO	7
-5,00E+18	FALSO	High	IsMasterPr	AAEL0017S	2:3747072	0	20	FALSO	18
-3,05E+18	FALSO	High	IsMasterPr	AAEL02555	3:1776203	0	55	FALSO	8
-7,45E+18	FALSO	High	IsMasterPr	AAEL01985	2:3526920	0	18	FALSO	21
6,80E+18	FALSO	High	IsMasterPr	AAEL02318	2:2594551	0	37	FALSO	14
3,43E+18	FALSO	High	IsMasterPr	AAEL0017C	2:4432688	0	78	FALSO	6
-7,23E+18	FALSO	High	IsMasterPr	AAEL01177	3:3670918	0	33	FALSO	9

1,71E+18	FALSO	High	IsMasterPr	AAEL00171	2:44308040	0	31	FALSO	12
-8,31E+18	FALSO	High	IsMasterPr	AAEL00395	3:2678894:	0	21	FALSO	11
-5,81E+18	FALSO	High	IsMasterPr	AAEL01977	2:2321161	0	58	FALSO	10
3,28E+18	FALSO	High	IsMasterPr	AAEL01217	2:2042630	0	55	FALSO	13
1,59E+18	FALSO	High	IsMasterPr	AAEL00796	2:3516239	0	52	FALSO	8
-1,23E+18	FALSO	High	IsMasterPr	AAEL00810	3:1457207	0	45	FALSO	11
-3,82E+18	FALSO	High	IsMasterPr	AAEL02314	1:2140118	0	12	FALSO	23
-5,29E+18	FALSO	High	IsMasterPr	AAEL00250	1:9047130	0	48	FALSO	7
-8,75E+18	FALSO	High	IsMasterPr	AAEL00395	3:2682858	0	47	FALSO	10
7,10E+18	FALSO	High	IsMasterPr	AAEL00041	3:1865250	0	59	FALSO	9
-5,92E+17	FALSO	High	IsMasterPr	AAEL02082	3:6514896	0	36	FALSO	14
7,26E+18	FALSO	High	IsMasterPr	AAEL02084	2:1328593	0	38	FALSO	16
-2,60E+18	FALSO	High	IsMasterPr	AAEL00756	2:2489402	0	23	FALSO	19
5,33E+17	FALSO	High	IsMasterPr	AAEL01952	2:4371144	0	25	FALSO	15
-5,85E+18	FALSO	High	IsMasterPr	AAEL01056	1:5069677	0	56	FALSO	13
8,96E+18	FALSO	High	IsMasterPr	AAEL01334	2:1356557	0	57	FALSO	9
8,56E+18	FALSO	High	IsMasterPr	AAEL00738	1:2597951	0	56	FALSO	9
4,28E+18	FALSO	High	IsMasterPr	AAEL02235	1:1314510	0	42	FALSO	10
8,45E+18	FALSO	High	IsMasterPr	AAEL02440	2:1806008	0	23	FALSO	12
-8,20E+18	FALSO	High	IsMasterPr	AAEL00363	1:1893358	0	49	FALSO	9
-8,92E+18	FALSO	High	IsMasterPr	AAEL00729	3:1955801	0	27	FALSO	11
-1,12E+18	FALSO	High	IsMasterPr	AAEL02680	2:1500186	0	19	FALSO	19
2,86E+18	FALSO	High	IsMasterPr	AAEL00428	2:2769752	0	60	FALSO	7
-7,34E+18	FALSO	High	IsMasterPr	AAEL01967	2:1176719	0	12	FALSO	12
2,10E+18	FALSO	High	IsMasterPr	AAEL00634	3:3969348	0	29	FALSO	14
-3,62E+18	FALSO	High	IsMasterPr	AAEL00650	2:4686900	0	38	FALSO	5
5,09E+18	FALSO	High	IsMasterPr	AAEL00374	1:2478678	0	21	FALSO	13
-5,81E+18	FALSO	High	IsMasterPr	AAEL00411	3:4017303	0	46	FALSO	14
3,48E+15	FALSO	High	IsMasterPr	AAEL01231	2:1030437	0	39	FALSO	14
-3,12E+17	FALSO	High	IsMasterPr	AAEL00884	2:2413748	0	35	FALSO	12
-7,52E+18	FALSO	High	IsMasterPr	AAEL02630	2:4287994	0	27	FALSO	1
4,28E+18	FALSO	High	IsMasterPr	AAEL00102	3:5518706	0	41	FALSO	9
-4,42E+18	FALSO	High	IsMasterPr	AAEL00070	2:4589742	0	29	FALSO	11
3,96E+18	FALSO	High	IsMasterPr	AAEL00644	2:4394882	0	27	FALSO	13
8,64E+18	FALSO	High	IsMasterPr	AAEL00327	2:3663550	0	55	FALSO	6
-5,29E+18	FALSO	High	IsMasterPr	AAEL00131	1:3493314	0	42	FALSO	4
-8,74E+18	FALSO	High	IsMasterPr	AAEL00204	1:5823199	0	67	FALSO	13
3,96E+18	FALSO	High	IsMasterPr	AAEL00482	2:1157129	0	39	FALSO	10
3,27E+18	FALSO	High	IsMasterPr	AAEL00927	3:2002462	0	64	FALSO	3
-2,92E+18	FALSO	High	IsMasterPr	AAEL01516	1:2760114	0	77	FALSO	9
-3,51E+18	FALSO	High	IsMasterPr	AAEL01338	2:1772812	0	25	FALSO	3
5,21E+18	FALSO	High	IsMasterPr	AAEL01227	2:1450205	0	24	FALSO	17
-4,02E+18	FALSO	High	IsMasterPr	AAEL01054	2:3672065	0	68	FALSO	7
-8,18E+18	FALSO	High	IsMasterPr	AAEL02501	2:1682771	0	35	FALSO	14
-7,57E+18	FALSO	High	IsMasterPr	AAEL00241	1:1767190	0	35	FALSO	15
-5,84E+18	FALSO	High	IsMasterPr	AAEL01211	2:4056084	0	66	FALSO	10
4,52E+18	FALSO	High	IsMasterPr	AAEL00315	1:1929357	0	44	FALSO	14
2,81E+18	FALSO	High	IsMasterPr	AAEL00931	1:1998214	0	29	FALSO	11
1,55E+18	FALSO	High	IsMasterPr	AAEL00593	3:1385811	0	23	FALSO	8
-2,25E+18	FALSO	High	IsMasterPr	AAEL00976	1:4146382	0	37	FALSO	13

-8,27E+18	FALSO	High	IsMasterPr	AAEL01014	2:2650951	0	43	FALSO	10
1,87E+18	FALSO	High	IsMasterPr	AAEL01955	2:4264048	0	36	FALSO	10
-8,40E+18	FALSO	High	IsMasterPr	AAEL00683	3:5641171	0	47	FALSO	7
-2,35E+18	FALSO	High	IsMasterPr	AAEL00975	3:2844864	0	49	FALSO	14
-9,17E+18	FALSO	High	IsMasterPr	AAEL00408	3:4017353	0	38	FALSO	9
3,07E+18	FALSO	High	IsMasterPr	AAEL00807	2:2307360	0	35	FALSO	10
5,97E+18	FALSO	High	IsMasterPr	AAEL00965	3:2691023	0	43	FALSO	15
7,30E+18	FALSO	High	IsMasterPr	AAEL00928	2:1808271	0	51	FALSO	11
-8,75E+18	FALSO	High	IsMasterPr	AAEL00682	1:8339241	0	14	FALSO	8
6,30E+18	FALSO	High	IsMasterPr	AAEL00037	2:1246285	0	35	FALSO	11
7,57E+18	FALSO	High	IsMasterPr	AAEL02663	NIGP0100C	0	28	FALSO	11
9,12E+17	FALSO	High	IsMasterPr	AAEL01956	3:1433566	0	14	FALSO	14
-9,00E+18	FALSO	High	IsMasterPr	AAEL00088	3:3322325	0	54	FALSO	11
-2,57E+18	FALSO	High	IsMasterPr	AAEL00194	2:1933518	0	16	FALSO	3
1,22E+17	FALSO	High	IsMasterPr	AAEL02394	3:2910857	0	64	FALSO	5
-5,15E+18	FALSO	High	IsMasterPr	AAEL01014	2:2650951	0	35	FALSO	8
7,78E+18	FALSO	High	IsMasterPr	AAEL01495	2:2227809	0	31	FALSO	10
8,95E+17	FALSO	High	IsMasterPr	AAEL01291	3:2791475	0	30	FALSO	5
8,88E+18	FALSO	High	IsMasterPr	AAEL01726	3:7807941	0	20	FALSO	15
-6,92E+18	FALSO	High	IsMasterPr	AAEL01545	2:1978995	0	48	FALSO	6
6,87E+18	FALSO	High	IsMasterPr	AAEL00825	3:6573829	0	31	FALSO	16
-6,07E+18	FALSO	High	IsMasterPr	AAEL00509	3:8282040	0	36	FALSO	9
-3,95E+17	FALSO	High	IsMasterPr	AAEL02314	1:2140118	0	24	FALSO	16
9,17E+18	FALSO	High	IsMasterPr	AAEL00349	1:2291562	0	41	FALSO	8
-9,11E+18	FALSO	High	IsMasterPr	AAEL0105C	2:9106127	0	9	FALSO	18
-3,57E+18	FALSO	High	IsMasterPr	AAEL00791	1:6361462	0	42	FALSO	13
-2,40E+18	FALSO	High	IsMasterPr	AAEL01962	3:1954469	0	14	FALSO	9
-3,08E+18	FALSO	High	IsMasterPr	AAEL00074	2:5210709	0	44	FALSO	17
5,22E+18	FALSO	High	IsMasterPr	AAEL01965	1:3991058	0	18	FALSO	12
-6,44E+18	FALSO	High	IsMasterPr	AAEL00475	2:2886390	0	56	FALSO	9
-2,48E+18	FALSO	High	IsMasterPr	AAEL00456	1:1448605	0	39	FALSO	12
2,90E+18	FALSO	High	IsMasterPr	AAEL01194	1:2715741	0	23	FALSO	13
8,17E+18	FALSO	High	IsMasterPr	AAEL00048	3:1628550	0	20	FALSO	15
6,37E+18	FALSO	High	IsMasterPr	AAEL02484	3:3146644	0	21	FALSO	10
6,42E+18	FALSO	High	IsMasterPr	AAEL00283	3:2349633	0	17	FALSO	3
-3,31E+17	FALSO	High	IsMasterPr	AAEL0259C	1:5730183	0	50	FALSO	12
-1,15E+18	FALSO	High	IsMasterPr	AAEL0025C	1:9036245	0	38	FALSO	10
5,55E+18	FALSO	High	IsMasterPr	AAEL00589	3:1748752	0	38	FALSO	8
5,68E+18	FALSO	High	IsMasterPr	AAEL00704	3:2998507	0	16	FALSO	8
-5,55E+18	FALSO	High	IsMasterPr	AAEL00834	1:8199652	0	37	FALSO	8
-4,18E+18	FALSO	High	IsMasterPr	AAEL01393	2:3058310	0	20	FALSO	8
-1,21E+18	FALSO	High	IsMasterPr	AAEL02266	2:4191528	0	12	FALSO	10
6,71E+18	FALSO	High	IsMasterPr	AAEL00395	3:2674585	0	23	FALSO	11
6,91E+18	FALSO	High	IsMasterPr	AAEL01235	2:1115432	0	61	FALSO	7
3,14E+18	FALSO	High	IsMasterPr	AAEL0055C	1:1508864	0	41	FALSO	9
-6,54E+18	FALSO	High	IsMasterPr	AAEL0096C	2:1698840	0	19	FALSO	11
1,04E+18	FALSO	High	IsMasterPr	AAEL01982	2:3675683	0	18	FALSO	14
6,94E+18	FALSO	High	IsMasterPr	AAEL02171	NIGP01001	0	58	FALSO	6
5,49E+18	FALSO	High	IsMasterPr	AAEL00395	3:2674585	0	23	FALSO	11
-5,00E+18	FALSO	High	IsMasterPr	AAEL00542	1:2473354	0	14	FALSO	9

2,28E+18	FALSO	High	IsMasterPr	AAEL00875	3:32695960	0	28	FALSO	7
7,21E+18	FALSO	High	IsMasterPr	AAEL01974	3:1678285	0	17	FALSO	15
9,05E+18	FALSO	High	IsMasterPr	AAEL00627	2:2213095	0	31	FALSO	5
-1,83E+18	FALSO	High	IsMasterPr	AAEL01106	3:2944803	0	71	FALSO	5
-1,86E+18	FALSO	High	IsMasterPr	AAEL01823	2:2093545	0	14	FALSO	7
6,05E+18	FALSO	High	IsMasterPr	AAEL02266	2:4191528	0	13	FALSO	10
-6,94E+18	FALSO	High	IsMasterPr	AAEL00395	3:2679928	0	17	FALSO	12
-8,09E+18	FALSO	High	IsMasterPr	AAEL00202	2:4255586	0	21	FALSO	9
1,59E+18	FALSO	High	IsMasterPr	AAEL01486	2:1557703	0	16	FALSO	10
4,29E+18	FALSO	High	IsMasterPr	AAEL00908	2:3663606	0	28	FALSO	10
-3,71E+18	FALSO	High	IsMasterPr	AAEL00555	1:2418903	0	46	FALSO	11
-7,34E+18	FALSO	High	IsMasterPr	AAEL00907	2:4114737	0	25	FALSO	4
7,86E+18	FALSO	High	IsMasterPr	AAEL00288	1:2559085	0	71	FALSO	10
-4,45E+18	FALSO	High	IsMasterPr	AAEL00669	3:3532294	0	52	FALSO	11
8,25E+16	FALSO	High	IsMasterPr	AAEL01294	3:2062069	0	47	FALSO	11
-7,79E+18	FALSO	High	IsMasterPr	AAEL00184	2:2766819	0	35	FALSO	7
-5,92E+18	FALSO	High	IsMasterPr	AAEL02504	2:1817834	0	18	FALSO	6
7,81E+17	FALSO	High	IsMasterPr	AAEL02536	1:1776297	0	27	FALSO	14
4,38E+18	FALSO	High	IsMasterPr	AAEL02126	3:2635109	0	15	FALSO	10
4,70E+18	FALSO	High	IsMasterPr	AAEL00873	3:2810905	0	25	FALSO	12
-6,96E+18	FALSO	High	IsMasterPr	AAEL01942	1:1162581	0	9	FALSO	11
4,61E+18	FALSO	High	IsMasterPr	AAEL02543	2:2000209	0	33	FALSO	12
6,42E+18	FALSO	High	IsMasterPr	AAEL00963	1:2227034	0	21	FALSO	7
-1,35E+18	FALSO	High	IsMasterPr	AAEL01971	2:3963811	0	13	FALSO	13
8,82E+18	FALSO	High	IsMasterPr	AAEL01869	2:6259966	0	8	FALSO	17
-5,26E+18	FALSO	High	IsMasterPr	AAEL00704	3:2998278	0	27	FALSO	4
3,87E+18	FALSO	High	IsMasterPr	AAEL01323	3:2800408	0	34	FALSO	11
-5,40E+18	FALSO	High	IsMasterPr	AAEL00962	3:3664935	0	21	FALSO	9
8,33E+18	FALSO	High	IsMasterPr	AAEL00988	3:1219452	0	33	FALSO	15
7,35E+18	FALSO	High	IsMasterPr	AAEL01046	1:2045319	0	28	FALSO	7
1,67E+18	FALSO	High	IsMasterPr	AAEL02731	1:3488502	0	51	FALSO	3
1,09E+18	FALSO	High	IsMasterPr	AAEL01452	1:2243096	0	38	FALSO	7
9,19E+18	FALSO	High	IsMasterPr	AAEL00395	3:2679928	0	17	FALSO	11
2,94E+18	FALSO	High	IsMasterPr	AAEL01052	2:2599025	0	16	FALSO	13
-5,54E+18	FALSO	High	IsMasterPr	AAEL00869	2:2457462	0	51	FALSO	5
1,40E+18	FALSO	High	IsMasterPr	AAEL00218	2:4287836	0	21	FALSO	1
-6,49E+18	FALSO	High	IsMasterPr	AAEL01306	2:2534727	0	45	FALSO	10
8,61E+17	FALSO	High	IsMasterPr	AAEL00207	1:5839417	0	34	FALSO	8
-7,59E+18	FALSO	High	IsMasterPr	AAEL01118	3:5157220	0	20	FALSO	9
-7,94E+18	FALSO	High	IsMasterPr	A0A5R9ME	Chaperonir	0	35	FALSO	16
4,63E+18	FALSO	High	IsMasterPr	AAEL00953	3:2237470	0	41	FALSO	7
-8,17E+18	FALSO	High	IsMasterPr	AAEL02497	1:5959519	0	44	FALSO	12
-7,59E+18	FALSO	High	IsMasterPr	AAEL01211	2:4055830	0	67	FALSO	8
-6,32E+18	FALSO	High	IsMasterPr	AAEL00770	2:4702684	0	43	FALSO	14
-2,20E+18	FALSO	High	IsMasterPr	AAEL02303	2:1472294	0	30	FALSO	10
-6,78E+18	FALSO	High	IsMasterPr	AAEL01241	3:1114086	0	40	FALSO	11
-2,28E+18	FALSO	High	IsMasterPr	AAEL00647	3:2162887	0	23	FALSO	15
-6,50E+18	FALSO	High	IsMasterPr	AAEL01470	2:4645965	0	17	FALSO	11
-6,32E+18	FALSO	High	IsMasterPr	AAEL00519	2:1137947	0	50	FALSO	5
8,89E+18	FALSO	High	IsMasterPr	AAEL00909	3:1348774	0	42	FALSO	11

2,61E+18	FALSO	High	IsMasterPr	AAEL00594	1:2577471	0	50	FALSO	6
-2,74E+18	FALSO	High	IsMasterPr	AAEL00583	1:1164321	0	36	FALSO	9
6,76E+18	FALSO	High	IsMasterPr	AAEL02568	NIGP01001	0	11	FALSO	11
-4,60E+18	FALSO	High	IsMasterPr	AAEL00297	1:2615145	0	44	FALSO	14
3,66E+18	FALSO	High	IsMasterPr	AAEL00817	3:1025218	0	19	FALSO	12
-1,01E+18	FALSO	High	IsMasterPr	AAEL02491	1:6114194	0	16	FALSO	7
2,74E+17	FALSO	High	IsMasterPr	AAEL00927	3:2001246	0	31	FALSO	8
5,53E+18	FALSO	High	IsMasterPr	AAEL01047	1:2046985	0	18	FALSO	7
-8,52E+18	FALSO	High	IsMasterPr	AAEL00506	1:2957745	0	50	FALSO	9
7,23E+18	FALSO	High	IsMasterPr	AAEL01496	1:2860398	0	29	FALSO	11
8,06E+18	FALSO	High	IsMasterPr	AAEL00176	2:1668519	0	31	FALSO	14
-3,82E+16	FALSO	High	IsMasterPr	AAEL00694	1:1572137	0	17	FALSO	13
-3,79E+18	FALSO	High	IsMasterPr	AAEL00144	1:1115484	0	10	FALSO	12
-6,14E+18	FALSO	High	IsMasterPr	AAEL01373	3:2542312	0	22	FALSO	10
2,44E+18	FALSO	High	IsMasterPr	AAEL0084C	1:9355659	0	29	FALSO	8
3,35E+18	FALSO	High	IsMasterPr	AAEL00025	1:6641690	0	24	FALSO	10
8,52E+18	FALSO	High	IsMasterPr	AAEL0199C	3:1094851	0	38	FALSO	5
8,51E+18	FALSO	High	IsMasterPr	AAEL01074	2:4065445	0	30	FALSO	8
-8,54E+18	FALSO	High	IsMasterPr	AAEL0080C	2:3647230	0	45	FALSO	10
2,39E+18	FALSO	High	IsMasterPr	B7TWA8	ATP syntha	0	20	FALSO	5
-8,76E+18	FALSO	High	IsMasterPr	AAEL00251	1:9039438	0	26	FALSO	9
7,07E+18	FALSO	High	IsMasterPr	AAEL00098	3:3158760	0	36	FALSO	7
8,15E+18	FALSO	High	IsMasterPr	AAEL00641	2:4150460	0	26	FALSO	9
-7,46E+18	FALSO	High	IsMasterPr	AAEL00577	1:1223858	0	25	FALSO	4
8,33E+18	FALSO	High	IsMasterPr	AAEL02398	2:1789887	0	32	FALSO	7
2,43E+17	FALSO	High	IsMasterPr	AAEL00424	1:1054010	0	38	FALSO	15
2,57E+18	FALSO	High	IsMasterPr	AAEL00752	2:2175457	0	29	FALSO	10
-3,76E+18	FALSO	High	IsMasterPr	AAEL02052	3:1746698	0	11	FALSO	15
5,14E+18	FALSO	High	IsMasterPr	AAEL00989	1:2072551	0	32	FALSO	11
-4,56E+18	FALSO	High	IsMasterPr	AAEL01393	2:3522867	0	29	FALSO	7
-6,04E+18	FALSO	High	IsMasterPr	AAEL01496	1:2860398	0	21	FALSO	11
-8,51E+18	FALSO	High	IsMasterPr	AAEL00484	2:1154502	0	60	FALSO	8
5,97E+18	FALSO	High	IsMasterPr	AAEL00048	3:1612878	0	28	FALSO	11
-6,76E+18	FALSO	High	IsMasterPr	AAEL0074C	2:1232220	0	5	FALSO	9
-2,71E+17	FALSO	High	IsMasterPr	AAEL01085	1:5468388	0	44	FALSO	11
-3,82E+18	FALSO	High	IsMasterPr	AAEL0011C	3:1679251	0	40	FALSO	4
4,56E+17	FALSO	High	IsMasterPr	AAEL00246	3:3381906	0	54	FALSO	6
-1,22E+18	FALSO	High	IsMasterPr	AAEL00602	3:3151158	0	26	FALSO	6
-4,08E+18	FALSO	High	IsMasterPr	AAEL01966	2:2055348	0	47	FALSO	9
-4,01E+18	FALSO	High	IsMasterPr	AAEL00541	1:2472503	0	32	FALSO	8
2,95E+18	FALSO	High	IsMasterPr	AAEL01832	2:3878376	0	8	FALSO	11
4,89E+17	FALSO	High	IsMasterPr	AAEL00041	3:1871206	0	37	FALSO	12
-1,41E+18	FALSO	High	IsMasterPr	AAEL00916	3:1688966	0	18	FALSO	12
-5,97E+17	FALSO	High	IsMasterPr	AAEL0032C	1:1902012	0	48	FALSO	6
1,92E+18	FALSO	High	IsMasterPr	AAEL00706	3:2984905	0	22	FALSO	13
4,46E+18	FALSO	High	IsMasterPr	AAEL00588	2:6617037	0	30	FALSO	4
5,91E+18	FALSO	High	IsMasterPr	AAEL01209	1:104374:1	0	44	FALSO	11
8,90E+18	FALSO	High	IsMasterPr	AAEL00526	1:3212065	0	55	FALSO	9
-5,38E+18	FALSO	High	IsMasterPr	AAEL00839	1:9350561	0	56	FALSO	10
-5,49E+18	FALSO	High	IsMasterPr	AAEL01128	1:1499035	0	18	FALSO	15

-5,14E+18	FALSO	High	IsMasterPr	AAEL01031	3:4089805	0	15	FALSO	8
-7,97E+18	FALSO	High	IsMasterPr	AAEL00638	3:2310673	0	19	FALSO	8
7,09E+18	FALSO	High	IsMasterPr	AAEL01125	1:2232008	0	14	FALSO	15
-7,46E+18	FALSO	High	IsMasterPr	AAEL00979	1:2760699	0	70	FALSO	8
4,59E+18	FALSO	High	IsMasterPr	AAEL01217	2:2042111	0	26	FALSO	13
-2,84E+18	FALSO	High	IsMasterPr	AAEL01291	2:1602407	0	20	FALSO	13
-9,09E+17	FALSO	High	IsMasterPr	AAEL00647	3:2163875	0	6	FALSO	13
-5,54E+18	FALSO	High	IsMasterPr	AAEL00282	3:2349452	0	59	FALSO	4
4,70E+18	FALSO	High	IsMasterPr	AAEL01175	1:1473482	0	33	FALSO	11
-4,62E+18	FALSO	High	IsMasterPr	AAEL01064	2:3267967	0	19	FALSO	15
-9,03E+18	FALSO	High	IsMasterPr	AAEL01971	3:5159227	0	23	FALSO	9
-4,75E+18	FALSO	High	IsMasterPr	AAEL00041	3:1865250	0	55	FALSO	8
-8,92E+18	FALSO	High	IsMasterPr	AAEL00446	2:4733231	0	15	FALSO	10
-8,04E+18	FALSO	High	IsMasterPr	AAEL01220	1:1609261	0	35	FALSO	12
2,98E+18	FALSO	High	IsMasterPr	AAEL00865	3:1724437	0	34	FALSO	7
7,06E+18	FALSO	High	IsMasterPr	AAEL01708	2:2134744	0	51	FALSO	10
-7,40E+18	FALSO	High	IsMasterPr	AAEL01496	1:2860398	0	29	FALSO	11
-3,27E+18	FALSO	High	IsMasterPr	A0A141GD	Surface prc	0	44	FALSO	9
-9,01E+18	FALSO	High	IsMasterPr	AAEL01367	3:2355373	0	27	FALSO	8
3,47E+18	FALSO	High	IsMasterPr	AAEL01257	1:2855066	0	29	FALSO	7
9,12E+18	FALSO	High	IsMasterPr	AAEL00283	3:2350284	0	20	FALSO	8
1,90E+18	FALSO	High	IsMasterPr	AAEL00076	3:2464346	0	24	FALSO	6
6,36E+18	FALSO	High	IsMasterPr	AAEL02193	2:4288961	0	21	FALSO	2
5,97E+18	FALSO	High	IsMasterPr	AAEL02610	1:1620032	0	63	FALSO	7
-9,17E+18	FALSO	High	IsMasterPr	AAEL00789	2:4743162	0	35	FALSO	6
-4,60E+18	FALSO	High	IsMasterPr	AAEL01756	2:2058275	0	45	FALSO	4
-5,89E+18	FALSO	High	IsMasterPr	AAEL02759	NIGP0100C	0	44	FALSO	7
-5,91E+18	FALSO	High	IsMasterPr	AAEL00002	1:2430099	0	37	FALSO	7
4,04E+18	FALSO	High	IsMasterPr	AAEL00135	1:8458369	0	28	FALSO	5
1,56E+18	FALSO	High	IsMasterPr	AAEL02564	1:5519421	0	19	FALSO	8
3,50E+18	FALSO	High	IsMasterPr	AAEL00186	2:2751547	0	53	FALSO	6
6,02E+18	FALSO	High	IsMasterPr	AAEL02761	3:2509907	0	6	FALSO	17
9,04E+18	FALSO	High	IsMasterPr	AAEL00493	2:2608686	0	26	FALSO	14
-7,65E+18	FALSO	High	IsMasterPr	AAEL01220	1:1609261	0	16	FALSO	13
-6,65E+18	FALSO	High	IsMasterPr	AAEL00413	3:1539812	0	18	FALSO	7
-3,80E+18	FALSO	High	IsMasterPr	AAEL01995	2:3090591	0	27	FALSO	9
2,10E+18	FALSO	High	IsMasterPr	AAEL00169	2:8675428	0	19	FALSO	5
-5,02E+18	FALSO	High	IsMasterPr	AAEL01979	3:3694848	0	13	FALSO	2
1,68E+18	FALSO	High	IsMasterPr	AAEL01313	3:3713198	0	54	FALSO	11
-2,34E+18	FALSO	High	IsMasterPr	AAEL01307	2:2535148	0	31	FALSO	8
-1,09E+18	FALSO	High	IsMasterPr	AAEL00604	3:7402103	0	23	FALSO	7
8,94E+18	FALSO	High	IsMasterPr	AAEL01296	1:2304134	0	23	FALSO	8
-7,37E+17	FALSO	High	IsMasterPr	AAEL00521	3:8961683	0	36	FALSO	9
1,73E+18	FALSO	High	IsMasterPr	AAEL00447	3:1100000	0	20	FALSO	9
-7,79E+18	FALSO	High	IsMasterPr	AAEL02698	1:1942507	0	42	FALSO	10
-6,25E+18	FALSO	High	IsMasterPr	AAEL00523	3:8872371	0	14	FALSO	7
8,14E+18	FALSO	High	IsMasterPr	AAEL00766	3:3473169	0	36	FALSO	6
-6,08E+18	FALSO	High	IsMasterPr	AAEL00056	2:1475936	0	34	FALSO	5
-7,62E+18	FALSO	High	IsMasterPr	AAEL00311	2:1920492	0	49	FALSO	5
-8,76E+18	FALSO	High	IsMasterPr	AAEL00771	3:2554316	0	26	FALSO	11

5,36E+17	FALSO	High	IsMasterPr	AAEL01109	2:2322452	0	29	FALSO	6
6,95E+18	FALSO	High	IsMasterPr	AAEL01171	2:1057896	0	57	FALSO	7
7,29E+17	FALSO	High	IsMasterPr	AAEL00636	1:1013779	0	28	FALSO	10
7,67E+18	FALSO	High	IsMasterPr	AAEL00758	1:3022084	0	10	FALSO	11
-4,12E+18	FALSO	High	IsMasterPr	AAEL00304	1:1309211	0	50	FALSO	7
-3,76E+17	FALSO	High	IsMasterPr	AAEL02715	3:1493974	0	39	FALSO	10
7,99E+18	FALSO	High	IsMasterPr	AAEL01006	3:1069842	0	26	FALSO	9
5,09E+18	FALSO	High	IsMasterPr	AAEL01059	1:3013564	0	39	FALSO	8
-2,99E+18	FALSO	High	IsMasterPr	AAEL00828	2:6742083	0	15	FALSO	2
5,38E+18	FALSO	High	IsMasterPr	AAEL01494	3:2042548	0	52	FALSO	8
-2,65E+18	FALSO	High	IsMasterPr	AAEL01112	3:3785545	0	18	FALSO	7
1,97E+18	FALSO	High	IsMasterPr	AAEL00719	2:3187794	0	79	FALSO	4
1,32E+18	FALSO	High	IsMasterPr	AAEL00406	1:1370144	0	53	FALSO	3
-6,68E+18	FALSO	High	IsMasterPr	AAEL01307	1:3170725	0	31	FALSO	9
-5,77E+17	FALSO	High	IsMasterPr	AAEL01029	2:3412762	0	26	FALSO	10
3,72E+18	FALSO	High	IsMasterPr	AAEL02624	2:1742218	0	40	FALSO	6
-6,27E+18	FALSO	High	IsMasterPr	AAEL00217	1:1016034	0	26	FALSO	11
6,01E+18	FALSO	High	IsMasterPr	AAEL00906	2:2703395	0	28	FALSO	11
-6,51E+18	FALSO	High	IsMasterPr	AAEL01393	2:3058310	0	14	FALSO	6
-6,02E+18	FALSO	High	IsMasterPr	AAEL01012	2:1619357	0	37	FALSO	3
5,87E+18	FALSO	High	IsMasterPr	AAEL00977	1:6758739	0	51	FALSO	4
2,44E+18	FALSO	High	IsMasterPr	AAEL00668	2:3256064	0	74	FALSO	6
-8,18E+18	FALSO	High	IsMasterPr	AAEL00606	3:7404276	0	57	FALSO	9
6,41E+18	FALSO	High	IsMasterPr	AAEL00856	2:1571782	0	27	FALSO	12
-8,36E+17	FALSO	High	IsMasterPr	AAEL00323	2:3675195	0	43	FALSO	4
9,01E+18	FALSO	High	IsMasterPr	AAEL00414	3:1537202	0	39	FALSO	11
8,45E+18	FALSO	High	IsMasterPr	AAEL00383	3:3430417	0	5	FALSO	16
2,54E+18	FALSO	High	IsMasterPr	AAEL00932	1:1998390	0	20	FALSO	8
-3,68E+17	FALSO	High	IsMasterPr	AAEL00980	1:2760655	0	57	FALSO	8
-8,54E+18	FALSO	High	IsMasterPr	AAEL00805	2:2306247	0	26	FALSO	16
2,58E+18	FALSO	High	IsMasterPr	AAEL00441	3:2710682	0	22	FALSO	10
-8,53E+18	FALSO	High	IsMasterPr	AAEL00388	3:8465654	0	50	FALSO	7
3,05E+17	FALSO	High	IsMasterPr	AAEL00338	3:8151374	0	38	FALSO	8
1,76E+18	FALSO	High	IsMasterPr	AAEL00700	3:1711350	0	39	FALSO	6
1,73E+18	FALSO	High	IsMasterPr	AAEL01396	3:1061903	0	25	FALSO	9
-5,95E+18	FALSO	High	IsMasterPr	AAEL00240	2:3022646	0	58	FALSO	9
7,19E+18	FALSO	High	IsMasterPr	AAEL00210	3:1778295	0	17	FALSO	5
-2,70E+18	FALSO	High	IsMasterPr	AAEL00642	2:4150230	0	22	FALSO	9
6,38E+18	FALSO	High	IsMasterPr	AAEL01441	2:3662016	0	33	FALSO	5
5,89E+18	FALSO	High	IsMasterPr	AAEL01740	2:4288661	0	21	FALSO	1
-2,86E+18	FALSO	High	IsMasterPr	AAEL00804	3:1320948	0	17	FALSO	7
9,16E+18	FALSO	High	IsMasterPr	AAEL00301	1:1306985	0	35	FALSO	7
8,15E+18	FALSO	High	IsMasterPr	AAEL02705	3:2440301	0	28	FALSO	10
-1,36E+18	FALSO	High	IsMasterPr	AAEL00116	2:3090813	0	61	FALSO	9
-5,48E+18	FALSO	High	IsMasterPr	AAEL00821	2:5912503	0	20	FALSO	11
6,90E+18	FALSO	High	IsMasterPr	AAEL00645	3:2165744	0	21	FALSO	8
3,12E+18	FALSO	High	IsMasterPr	AAEL00723	2:4214849	0	14	FALSO	4
-3,75E+18	FALSO	High	IsMasterPr	AAEL00414	3:1537202	0	45	FALSO	11
3,00E+18	FALSO	High	IsMasterPr	AAEL01351	2:3759393	0	43	FALSO	3
6,81E+18	FALSO	High	IsMasterPr	AAEL01703	2:3911243	0	21	FALSO	5

-1,26E+18	FALSO	High	IsMasterPr	AAEL0205٩	3:1218892	0	9	FALSO	13
-4,00E+18	FALSO	High	IsMasterPr	AAEL00004	3:1020172	0	18	FALSO	7
-6,67E+17	FALSO	High	IsMasterPr	AAEL0259٩	NIGP01001	0	62	FALSO	7
4,07E+18	FALSO	High	IsMasterPr	AAEL00845	NIGP01001	0	34	FALSO	8
2,98E+18	FALSO	High	IsMasterPr	AAEL0102C	1:3581839	0	29	FALSO	10
5,43E+18	FALSO	High	IsMasterPr	AAEL01054	2:3146336	0	6	FALSO	2
8,09E+18	FALSO	High	IsMasterPr	AAEL00952	3:9765315	0	20	FALSO	9
6,89E+18	FALSO	High	IsMasterPr	AAEL01231	2:1029159	0	29	FALSO	10
-7,94E+18	FALSO	High	IsMasterPr	AAEL0088€	3:2366628	0	22	FALSO	16
-1,50E+18	FALSO	High	IsMasterPr	AAEL00215	1:2523374	0	38	FALSO	10
8,64E+18	FALSO	High	IsMasterPr	AAEL01215	2:2745243	0	22	FALSO	10
7,96E+18	FALSO	High	IsMasterPr	AAEL00375	1:2484834	0	36	FALSO	5
-5,91E+18	FALSO	High	IsMasterPr	AAEL00547	3:3832920	0	25	FALSO	10
3,11E+17	FALSO	High	IsMasterPr	AAEL0151C	2:2330264	0	11	FALSO	7
-7,80E+18	FALSO	High	IsMasterPr	AAEL01075	3:2942249	0	48	FALSO	9
7,86E+18	FALSO	High	IsMasterPr	AAEL0097C	3:1556082	0	18	FALSO	7
-4,09E+18	FALSO	High	IsMasterPr	AAEL0080C	2:3655393	0	38	FALSO	5
1,65E+18	FALSO	High	IsMasterPr	AAEL0130٩	2:3377590	0	14	FALSO	10
8,84E+18	FALSO	High	IsMasterPr	AAEL00218	2:4284055	0	20	FALSO	4
4,93E+18	FALSO	High	IsMasterPr	AAEL00667	2:3257899	0	35	FALSO	12
7,95E+18	FALSO	High	IsMasterPr	AAEL01174	3:2910590	0	33	FALSO	6
9,19E+18	FALSO	High	IsMasterPr	AAEL00925	2:2684722	0	27	FALSO	4
8,71E+18	FALSO	High	IsMasterPr	AAEL01197	2:2091196	0	29	FALSO	9
6,37E+18	FALSO	High	IsMasterPr	AAEL0100€	3:1068406	0	27	FALSO	13
-1,52E+18	FALSO	High	IsMasterPr	AAEL01141	3:3288475	0	54	FALSO	7
1,91E+18	FALSO	High	IsMasterPr	AAEL01398	3:2305839	0	43	FALSO	10
5,42E+18	FALSO	High	IsMasterPr	AAEL02547	3:1225773	0	45	FALSO	8
6,58E+18	FALSO	High	IsMasterPr	AAEL01231	2:1028683	0	29	FALSO	10
5,11E+18	FALSO	High	IsMasterPr	AAEL00353	2:2562391	0	54	FALSO	6
3,46E+18	FALSO	High	IsMasterPr	A0A098AT\	Uncharacte	0	55	FALSO	3
-3,60E+18	FALSO	High	IsMasterPr	AAEL00692	3:1435881	0	45	FALSO	8
7,61E+18	FALSO	High	IsMasterPr	AAEL01377	2:2775700	0	44	FALSO	7
2,99E+17	FALSO	High	IsMasterPr	AAEL00115	2:3072040	0	21	FALSO	6
-7,15E+17	FALSO	High	IsMasterPr	AAEL01963	3:3704019	0	17	FALSO	8
9,58E+17	FALSO	High	IsMasterPr	AAEL0104٩	2:4464224	0	21	FALSO	6
-6,99E+18	FALSO	High	IsMasterPr	AAEL0082٩	2:6724025	0	49	FALSO	7
6,89E+18	FALSO	High	IsMasterPr	AAEL00657	3:4473793	0	20	FALSO	6
-3,26E+18	FALSO	High	IsMasterPr	AAEL01824	2:3279911	0	12	FALSO	9
-6,76E+18	FALSO	High	IsMasterPr	AAEL00895	1:8117864	0	24	FALSO	7
5,75E+18	FALSO	High	IsMasterPr	AAEL01038	2:1829785	0	15	FALSO	13
5,21E+18	FALSO	High	IsMasterPr	AAEL0019€	2:4262867	0	43	FALSO	9
-2,35E+18	FALSO	High	IsMasterPr	AAEL00418	1:1587690	0	26	FALSO	8
9,01E+18	FALSO	High	IsMasterPr	AAEL01353	2:2462414	0	35	FALSO	6
-1,63E+18	FALSO	High	IsMasterPr	AAEL0081٩	3:5358906	0	38	FALSO	10
-8,60E+18	FALSO	High	IsMasterPr	AAEL00914	2:3424820	0	18	FALSO	7
-3,37E+18	FALSO	High	IsMasterPr	AAEL00982	3:2024652	0	18	FALSO	10
9,19E+18	FALSO	High	IsMasterPr	AAEL00485	2:1159138	0	15	FALSO	10
-3,45E+18	FALSO	High	IsMasterPr	AAEL0110C	3:2877846	0	37	FALSO	7
-8,75E+17	FALSO	High	IsMasterPr	AAEL0120٩	1:242496:2	0	26	FALSO	8
4,10E+17	FALSO	High	IsMasterPr	AAEL0079٩	2:3657804	0	20	FALSO	7

8,63E+17	FALSO	High	IsMasterPr	AAEL01982	2:3676094	0	10	FALSO	5
-8,30E+18	FALSO	High	IsMasterPr	AAEL00378	1:2037303	0	16	FALSO	9
7,61E+18	FALSO	High	IsMasterPr	AAEL02666	3:3620140	0	26	FALSO	10
-3,75E+18	FALSO	High	IsMasterPr	AAEL00375	1:2483851	0	24	FALSO	11
-6,66E+18	FALSO	High	IsMasterPr	AAEL01080	3:1565805	0	14	FALSO	7
-3,53E+18	FALSO	High	IsMasterPr	AAEL00631	3:2828219	0	15	FALSO	7
-3,94E+18	FALSO	High	IsMasterPr	AAEL01085	1:5468388	0	36	FALSO	9
5,04E+18	FALSO	High	IsMasterPr	AAEL01978	2:1704224	0	13	FALSO	8
4,95E+18	FALSO	High	IsMasterPr	AAEL00932	1:5512877	0	48	FALSO	8
-1,80E+17	FALSO	High	IsMasterPr	AAEL01377	2:1225729	0	35	FALSO	9
7,73E+18	FALSO	High	IsMasterPr	AAEL00164	2:3003215	0	21	FALSO	9
2,60E+18	FALSO	High	IsMasterPr	AAEL02100	1:1374741	0	16	FALSO	8
5,30E+18	FALSO	High	IsMasterPr	AAEL00994	2:3796957	0	32	FALSO	11
-5,86E+17	FALSO	High	IsMasterPr	AAEL01276	3:1117485	0	36	FALSO	8
-6,39E+18	FALSO	High	IsMasterPr	AAEL00432	2:3866529	0	22	FALSO	6
1,36E+18	FALSO	High	IsMasterPr	AAEL01978	3:3040985	0	36	FALSO	7
-3,63E+18	FALSO	High	IsMasterPr	AAEL01258	1:2852190	0	23	FALSO	8
3,33E+18	FALSO	High	IsMasterPr	AAEL00342	3:3483911	0	65	FALSO	2
-8,92E+18	FALSO	High	IsMasterPr	AAEL01756	2:1257133	0	29	FALSO	13
6,30E+18	FALSO	High	IsMasterPr	AAEL00071	2:4588626	0	24	FALSO	8
-3,22E+18	FALSO	High	IsMasterPr	AAEL00651	2:4686282	0	20	FALSO	8
-1,90E+18	FALSO	High	IsMasterPr	AAEL00981	3:2031187	0	15	FALSO	4
5,64E+17	FALSO	High	IsMasterPr	AAEL01832	3:1099647	0	22	FALSO	10
8,77E+18	FALSO	High	IsMasterPr	AAEL02493	2:1795841	0	7	FALSO	10
2,01E+18	FALSO	High	IsMasterPr	AAEL01146	2:1436620	0	54	FALSO	5
-4,09E+18	FALSO	High	IsMasterPr	AAEL00817	3:1010383	0	48	FALSO	7
7,91E+18	FALSO	High	IsMasterPr	AAEL01239	1:2844125	0	34	FALSO	6
-8,93E+18	FALSO	High	IsMasterPr	AAEL00265	2:4333091	0	29	FALSO	11
-9,61E+17	FALSO	High	IsMasterPr	AAEL01243	1:2129188	0	19	FALSO	8
-4,92E+18	FALSO	High	IsMasterPr	AAEL01023	2:4029585	0	49	FALSO	7
5,29E+18	FALSO	High	IsMasterPr	AAEL00003	3:1028996	0	27	FALSO	7
-1,01E+18	FALSO	High	IsMasterPr	AAEL00495	3:2182259	0	36	FALSO	6
-3,24E+18	FALSO	High	IsMasterPr	AAEL01023	2:4028419	0	20	FALSO	5
5,05E+18	FALSO	High	IsMasterPr	AAEL00810	3:1472969	0	32	FALSO	4
3,45E+18	FALSO	High	IsMasterPr	AAEL00862	2:8056602	0	21	FALSO	8
6,75E+18	FALSO	High	IsMasterPr	AAEL01721	2:4743243	0	21	FALSO	3
3,43E+17	FALSO	High	IsMasterPr	AAEL02811	2:6506680	0	11	FALSO	9
1,76E+18	FALSO	High	IsMasterPr	AAEL00005	3:1041477	0	70	FALSO	7
4,55E+18	FALSO	High	IsMasterPr	AAEL00250	1:9049004	0	37	FALSO	12
4,55E+18	FALSO	High	IsMasterPr	AAEL01442	1:1590271	0	14	FALSO	12
4,69E+18	FALSO	High	IsMasterPr	AAEL02324	2:4084493	0	7	FALSO	5
6,22E+17	FALSO	High	IsMasterPr	AAEL00540	1:2471549	0	24	FALSO	5
8,50E+18	FALSO	High	IsMasterPr	AAEL00272	2:1443340	0	17	FALSO	6
2,46E+17	FALSO	High	IsMasterPr	AAEL00647	3:2165303	0	32	FALSO	7
-6,89E+17	FALSO	High	IsMasterPr	AAEL01060	3:2915991	0	24	FALSO	10
4,76E+18	FALSO	High	IsMasterPr	AAEL02785	NIGP0100	0	41	FALSO	5
-3,56E+18	FALSO	High	IsMasterPr	AAEL00366	2:3166103	0	24	FALSO	9
3,97E+17	FALSO	High	IsMasterPr	AAEL00565	2:3820374	0	26	FALSO	10
-1,71E+18	FALSO	High	IsMasterPr	AAEL01206	1:2704659	0	37	FALSO	3
5,48E+18	FALSO	High	IsMasterPr	AAEL02480	1:8613343	0	19	FALSO	8

-6,38E+18	FALSO	High	IsMasterPr	AAEL00312	2:1918542	0	28	FALSO	8
4,40E+18	FALSO	High	IsMasterPr	AAEL00527	2:6912329	0	22	FALSO	7
-6,90E+18	FALSO	High	IsMasterPr	AAEL01144	2:3715764	0	40	FALSO	10
-7,76E+18	FALSO	High	IsMasterPr	AAEL01022	2:4028431	0	21	FALSO	3
3,73E+18	FALSO	High	IsMasterPr	AAEL02678	2:1830449	0	10	FALSO	9
-7,25E+18	FALSO	High	IsMasterPr	AAEL0228C	1:1020689	0	26	FALSO	6
-4,87E+18	FALSO	High	IsMasterPr	AAEL0258C	3:1531784	0	22	FALSO	8
6,18E+18	FALSO	High	IsMasterPr	AAEL0035€	3:1800152	0	31	FALSO	9
-9,61E+17	FALSO	High	IsMasterPr	AAEL00777	2:2193461	0	32	FALSO	5
3,95E+18	FALSO	High	IsMasterPr	AAEL0145€	2:2352182	0	21	FALSO	3
5,05E+18	FALSO	High	IsMasterPr	AAEL0061€	1:1260304	0	24	FALSO	8
8,19E+18	FALSO	High	IsMasterPr	AAEL01313	3:3709437	0	27	FALSO	9
-7,87E+17	FALSO	High	IsMasterPr	Q5GRU7	ATP syntha	0	10	FALSO	4
2,44E+17	FALSO	High	IsMasterPr	AAEL0112€	1:2153346	0	32	FALSO	5
-2,39E+18	FALSO	High	IsMasterPr	AAEL01051	3:3100862	0	31	FALSO	9
-3,06E+18	FALSO	High	IsMasterPr	AAEL01243	1:2132974	0	33	FALSO	8
2,58E+18	FALSO	High	IsMasterPr	AAEL00135	3:4775475	0	15	FALSO	9
-7,90E+18	FALSO	High	IsMasterPr	AAEL01542	2:1784832	0	46	FALSO	7
-8,05E+18	FALSO	High	IsMasterPr	AAEL00422	1:1050603	0	15	FALSO	12
4,75E+18	FALSO	High	IsMasterPr	AAEL01144	2:2251318	0	18	FALSO	5
-2,40E+18	FALSO	High	IsMasterPr	AAEL00611	3:2597690	0	45	FALSO	7
7,62E+18	FALSO	High	IsMasterPr	AAEL01175	1:1473482	0	31	FALSO	9
-2,46E+17	FALSO	High	IsMasterPr	AAEL00399	3:2539916	0	30	FALSO	8
4,75E+18	FALSO	High	IsMasterPr	AAEL01372	1:2193364	0	22	FALSO	6
8,66E+18	FALSO	High	IsMasterPr	AAEL02811	2:6506680	0	11	FALSO	9
-5,18E+17	FALSO	High	IsMasterPr	AAEL00287	1:2544717	0	18	FALSO	9
-6,24E+18	FALSO	High	IsMasterPr	AAEL00392	3:2616320	0	19	FALSO	5
7,12E+18	FALSO	High	IsMasterPr	AAEL02803	NIGP01001	0	13	FALSO	5
-2,41E+17	FALSO	High	IsMasterPr	AAEL0109€	2:1512242	0	38	FALSO	6
-8,07E+18	FALSO	High	IsMasterPr	AAEL01482	1:1492265	0	32	FALSO	5
-3,00E+18	FALSO	High	IsMasterPr	AAEL0204C	1:1615667	0	41	FALSO	8
6,22E+18	FALSO	High	IsMasterPr	AAEL02385	1:2012533	0	13	FALSO	13
-7,31E+18	FALSO	High	IsMasterPr	AAEL01565	1:1082306	0	45	FALSO	7
1,58E+18	FALSO	High	IsMasterPr	AAEL0096C	2:1691899	0	32	FALSO	6
7,85E+18	FALSO	High	IsMasterPr	AAEL01058	2:1119430	0	21	FALSO	6
-4,07E+18	FALSO	High	IsMasterPr	AAEL01999	1:2324437	0	19	FALSO	8
4,69E+18	FALSO	High	IsMasterPr	AAEL02074	3:5637233	0	21	FALSO	9
4,70E+18	FALSO	High	IsMasterPr	AAEL02172	1:2922660	0	41	FALSO	7
8,12E+18	FALSO	High	IsMasterPr	AAEL02001	1:2760754	0	66	FALSO	9
-2,66E+18	FALSO	High	IsMasterPr	AAEL0011C	3:4071000	0	15	FALSO	5
-5,97E+18	FALSO	High	IsMasterPr	AAEL00773	2:1008980	0	46	FALSO	8
3,60E+18	FALSO	High	IsMasterPr	AAEL01313	3:3709432	0	25	FALSO	9
6,65E+18	FALSO	High	IsMasterPr	AAEL0041C	3:4036706	0	55	FALSO	6
-5,64E+18	FALSO	High	IsMasterPr	AAEL0019€	2:4259773	0	44	FALSO	8
4,71E+18	FALSO	High	IsMasterPr	AAEL00713	3:6978110	0	27	FALSO	5
-5,15E+18	FALSO	High	IsMasterPr	AAEL00557	3:4047170	0	13	FALSO	8
-4,61E+18	FALSO	High	IsMasterPr	AAEL00478	2:2884844	0	55	FALSO	5
-2,77E+18	FALSO	High	IsMasterPr	AAEL0021€	1:1022716	0	32	FALSO	11
-2,62E+18	FALSO	High	IsMasterPr	AAEL0083€	3:4131022	0	36	FALSO	9
1,24E+18	FALSO	High	IsMasterPr	AAEL0043C	2:3866642	0	42	FALSO	8

-1,54E+18	FALSO	High	IsMasterPr	AAEL0096	2:1699450	0	26	FALSO	6
2,70E+18	FALSO	High	IsMasterPr	AAEL0229	1:1344227	0	17	FALSO	9
-2,72E+18	FALSO	High	IsMasterPr	AAEL0195	2:2116446	0	17	FALSO	11
1,41E+18	FALSO	High	IsMasterPr	AAEL0290	2:3665030	0	41	FALSO	8
-8,57E+18	FALSO	High	IsMasterPr	AAEL0039	1:2505839	0	39	FALSO	7
4,13E+18	FALSO	High	IsMasterPr	AAEL0109	3:9696429	0	16	FALSO	10
2,60E+18	FALSO	High	IsMasterPr	AAEL0110	2:2355533	0	34	FALSO	5
3,52E+16	FALSO	High	IsMasterPr	AAEL0090	2:2695416	0	8	FALSO	7
-7,38E+17	FALSO	High	IsMasterPr	AAEL0040	1:1373641	0	29	FALSO	7
-2,80E+18	FALSO	High	IsMasterPr	AAEL0131	2:1884253	0	47	FALSO	8
-8,70E+17	FALSO	High	IsMasterPr	AAEL0079	2:3676957	0	36	FALSO	5
2,40E+18	FALSO	High	IsMasterPr	AAEL0241	3:3139528	0	10	FALSO	7
3,70E+18	FALSO	High	IsMasterPr	AAEL0059	3:1739150	0	54	FALSO	5
-6,18E+18	FALSO	High	IsMasterPr	AAEL0078	2:4605951	0	45	FALSO	6
-1,35E+18	FALSO	High	IsMasterPr	AAEL0044	3:2123412	0	39	FALSO	5
8,19E+18	FALSO	High	IsMasterPr	AAEL0245	2:4524646	0	9	FALSO	10
1,68E+18	FALSO	High	IsMasterPr	AAEL0197	3:6442786	0	46	FALSO	8
-4,35E+18	FALSO	High	IsMasterPr	AAEL0146	2:1574918	0	48	FALSO	4
5,26E+17	FALSO	High	IsMasterPr	AAEL0221	3:1832170	0	14	FALSO	7
7,88E+18	FALSO	High	IsMasterPr	AAEL0051	2:1729399	0	30	FALSO	2
-3,01E+18	FALSO	High	IsMasterPr	AAEL0118	2:3962271	0	19	FALSO	6
-2,43E+18	FALSO	High	IsMasterPr	AAEL0076	2:4703206	0	37	FALSO	4
-8,57E+18	FALSO	High	IsMasterPr	AAEL0065	2:1331804	0	22	FALSO	6
-5,66E+18	FALSO	High	IsMasterPr	AAEL0081	3:1074168	0	16	FALSO	7
9,18E+17	FALSO	High	IsMasterPr	AAEL0233	1:2066643	0	17	FALSO	7
-2,26E+17	FALSO	High	IsMasterPr	AAEL0211	1:1467177	0	17	FALSO	8
-5,47E+18	FALSO	High	IsMasterPr	AAEL0108	3:5178930	0	21	FALSO	7
-1,83E+18	FALSO	High	IsMasterPr	AAEL0077	2:2194024	0	27	FALSO	9
4,47E+18	FALSO	High	IsMasterPr	AAEL0005	3:1614874	0	17	FALSO	8
-7,64E+18	FALSO	High	IsMasterPr	AAEL0126	2:1879592	0	24	FALSO	4
-1,38E+18	FALSO	High	IsMasterPr	AAEL0098	3:2028345	0	27	FALSO	6
-8,84E+18	FALSO	High	IsMasterPr	AAEL0149	NIGP0100	0	10	FALSO	8
4,73E+18	FALSO	High	IsMasterPr	AAEL0038	3:8462529	0	34	FALSO	8
-4,40E+18	FALSO	High	IsMasterPr	AAEL0062	2:2203646	0	35	FALSO	8
-3,87E+18	FALSO	High	IsMasterPr	AAEL0014	2:2626985	0	23	FALSO	11
-8,65E+16	FALSO	High	IsMasterPr	AAEL0100	2:2004565	0	28	FALSO	5
4,08E+18	FALSO	High	IsMasterPr	AAEL0037	1:3039075	0	21	FALSO	6
-3,24E+18	FALSO	High	IsMasterPr	AAEL0144	2:1007177	0	36	FALSO	6
1,19E+18	FALSO	High	IsMasterPr	AAEL0195	3:6555998	0	9	FALSO	8
8,33E+18	FALSO	High	IsMasterPr	AAEL0040	1:1357692	0	29	FALSO	8
3,88E+18	FALSO	High	IsMasterPr	AAEL0001	3:2123904	0	16	FALSO	9
-3,89E+18	FALSO	High	IsMasterPr	AAEL0046	1:1590013	0	31	FALSO	8
9,08E+18	FALSO	High	IsMasterPr	AAEL0105	1:3013658	0	24	FALSO	9
9,30E+16	FALSO	High	IsMasterPr	AAEL0261	2:1319948	0	25	FALSO	8
1,06E+18	FALSO	High	IsMasterPr	AAEL0009	3:3769879	0	48	FALSO	7
6,82E+18	FALSO	High	IsMasterPr	AAEL0084	NIGP0100	0	25	FALSO	8
-3,48E+18	FALSO	High	IsMasterPr	AAEL0085	3:2225673	0	21	FALSO	8
-6,56E+18	FALSO	High	IsMasterPr	AAEL0093	3:2760958	0	29	FALSO	8
5,68E+18	FALSO	High	IsMasterPr	AAEL0183	3:1099647	0	22	FALSO	10
8,99E+18	FALSO	High	IsMasterPr	AAEL0182	2:2315109	0	7	FALSO	6

6,06E+18	FALSO	High	IsMasterPr	AAEL0017C	2:8603048	0	36	FALSO	3
4,75E+18	FALSO	High	IsMasterPr	AAEL00431	3:5084020	0	59	FALSO	5
-2,48E+18	FALSO	High	IsMasterPr	AAEL00186	2:2758957	0	12	FALSO	4
-6,30E+18	FALSO	High	IsMasterPr	AAEL00015	3:1096613	0	26	FALSO	6
2,62E+18	FALSO	High	IsMasterPr	AAEL0265C	NIGP01001	0	53	FALSO	5
-7,47E+18	FALSO	High	IsMasterPr	AAEL00497	2:2817508	0	16	FALSO	8
6,42E+16	FALSO	High	IsMasterPr	AAEL01979	3:3595628	0	31	FALSO	8
6,54E+18	FALSO	High	IsMasterPr	AAEL00641	2:4143702	0	14	FALSO	9
-4,08E+18	FALSO	High	IsMasterPr	AAEL01026	2:3600115	0	13	FALSO	2
-4,60E+18	FALSO	High	IsMasterPr	AAEL00186	2:1844319	0	39	FALSO	10
-5,38E+18	FALSO	High	IsMasterPr	AAEL00707	1:2117067	0	7	FALSO	6
3,13E+18	FALSO	High	IsMasterPr	AAEL01362	2:3343539	0	30	FALSO	4
-7,43E+18	FALSO	High	IsMasterPr	AAEL00739	3:4074518	0	26	FALSO	4
7,27E+18	FALSO	High	IsMasterPr	AAEL00637	1:1004653	0	22	FALSO	4
2,44E+18	FALSO	High	IsMasterPr	AAEL01421	2:3228447	0	16	FALSO	8
-2,62E+18	FALSO	High	IsMasterPr	AAEL00576	2:4360936	0	11	FALSO	8
2,79E+18	FALSO	High	IsMasterPr	AAEL01976	2:1700444	0	40	FALSO	4
4,33E+18	FALSO	High	IsMasterPr	AAEL00388	3:8541259	0	21	FALSO	4
-8,19E+18	FALSO	High	IsMasterPr	AAEL00691	3:1431022	0	23	FALSO	4
2,83E+18	FALSO	High	IsMasterPr	AAEL00794	2:3517082	0	48	FALSO	7
6,51E+18	FALSO	High	IsMasterPr	AAEL01352	1:1440013	0	27	FALSO	7
7,99E+18	FALSO	High	IsMasterPr	AAEL01187	2:1872359	0	17	FALSO	5
-3,77E+18	FALSO	High	IsMasterPr	AAEL01384	1:1035290	0	20	FALSO	6
-8,93E+18	FALSO	High	IsMasterPr	AAEL00401	3:1662088	0	29	FALSO	7
-2,10E+18	FALSO	High	IsMasterPr	AAEL01124	2:1797610	0	12	FALSO	7
6,80E+18	FALSO	High	IsMasterPr	AAEL00656	2:5948983	0	26	FALSO	6
4,54E+18	FALSO	High	IsMasterPr	AAEL00595	1:2577203	0	38	FALSO	11
-1,19E+17	FALSO	High	IsMasterPr	AAEL00974	3:2844604	0	37	FALSO	6
-8,46E+18	FALSO	High	IsMasterPr	AAEL00434	3:3680691	0	17	FALSO	6
7,57E+18	FALSO	High	IsMasterPr	AAEL00409	3:4016820	0	30	FALSO	7
-6,06E+18	FALSO	High	IsMasterPr	AAEL02649	2:1551299	0	33	FALSO	7
-3,98E+18	FALSO	High	IsMasterPr	AAEL0059C	3:1740515	0	15	FALSO	5
7,28E+18	FALSO	High	IsMasterPr	AAEL02376	3:1775750	0	68	FALSO	5
-2,65E+18	FALSO	High	IsMasterPr	AAEL01231	2:1027485	0	45	FALSO	5
6,43E+18	FALSO	High	IsMasterPr	AAEL0056C	3:1600495	0	17	FALSO	7
-8,13E+17	FALSO	High	IsMasterPr	AAEL02001	1:2760830	0	52	FALSO	8
-6,26E+18	FALSO	High	IsMasterPr	AAEL00075	3:2461894	0	24	FALSO	7
-5,07E+18	FALSO	High	IsMasterPr	AAEL01115	3:1936602	0	61	FALSO	7
-8,74E+18	FALSO	High	IsMasterPr	AAEL02442	1:2208246	0	35	FALSO	3
8,60E+18	FALSO	High	IsMasterPr	AAEL00086	3:3359356	0	17	FALSO	6
-1,06E+18	FALSO	High	IsMasterPr	AAEL00931	1:1997465	0	29	FALSO	7
9,17E+18	FALSO	High	IsMasterPr	AAEL00545	2:2956151	0	39	FALSO	4
8,92E+18	FALSO	High	IsMasterPr	AAEL00408	1:1374176	0	37	FALSO	7
8,10E+17	FALSO	High	IsMasterPr	AAEL00814	3:3020751	0	13	FALSO	6
-8,05E+17	FALSO	High	IsMasterPr	AAEL0049C	2:7239377	0	16	FALSO	4
3,65E+17	FALSO	High	IsMasterPr	AAEL02384	2:1744003	0	18	FALSO	9
-2,06E+18	FALSO	High	IsMasterPr	AAEL0125C	2:1859710	0	51	FALSO	6
-7,09E+18	FALSO	High	IsMasterPr	AAEL02048	2:3366628	0	9	FALSO	3
-8,65E+18	FALSO	High	IsMasterPr	AAEL00769	2:4707850	0	17	FALSO	8
-8,32E+18	FALSO	High	IsMasterPr	AAEL00432	3:3657233	0	11	FALSO	7

3,05E+18	FALSO	High	IsMasterPr	AAEL00191	2:1934320	0	28	FALSO	5
-4,09E+18	FALSO	High	IsMasterPr	AAEL00466	3:2466320	0	14	FALSO	5
-3,32E+18	FALSO	High	IsMasterPr	AAEL01067	2:1364558	0	22	FALSO	1
5,08E+18	FALSO	High	IsMasterPr	AAEL00576	3:1140918	0	18	FALSO	7
-4,11E+18	FALSO	High	IsMasterPr	AAEL00877	2:1988106	0	23	FALSO	3
5,15E+18	FALSO	High	IsMasterPr	AAEL0035C	3:2635733	0	32	FALSO	7
-4,04E+18	FALSO	High	IsMasterPr	AAEL0003C	3:2664866	0	14	FALSO	7
-4,85E+18	FALSO	High	IsMasterPr	AAEL01016	3:1493528	0	28	FALSO	8
5,42E+18	FALSO	High	IsMasterPr	AAEL01261	1:1460499	0	9	FALSO	7
-7,07E+18	FALSO	High	IsMasterPr	AAEL00538	3:2357113	0	13	FALSO	9
-1,12E+18	FALSO	High	IsMasterPr	AAEL00542	1:2471687	0	36	FALSO	8
3,13E+18	FALSO	High	IsMasterPr	AAEL02031	2:4708266	0	14	FALSO	3
6,54E+18	FALSO	High	IsMasterPr	AAEL00498	2:2818827	0	7	FALSO	5
6,49E+18	FALSO	High	IsMasterPr	AAEL01356	2:8279808	0	15	FALSO	7
5,72E+18	FALSO	High	IsMasterPr	AAEL00916	1:1123449	0	42	FALSO	4
7,37E+18	FALSO	High	IsMasterPr	AAEL01481	1:2786021	0	25	FALSO	7
1,94E+18	FALSO	High	IsMasterPr	AAEL0016C	2:9069654	0	17	FALSO	4
4,09E+18	FALSO	High	IsMasterPr	AAEL02495	1:1920472	0	30	FALSO	5
5,27E+18	FALSO	High	IsMasterPr	AAEL00268	2:4648062	0	12	FALSO	10
8,74E+18	FALSO	High	IsMasterPr	AAEL0181C	3:3666283	0	11	FALSO	2
-8,67E+18	FALSO	High	IsMasterPr	AAEL0027C	2:1447471	0	16	FALSO	4
1,27E+18	FALSO	High	IsMasterPr	AAEL01717	3:3508298	0	18	FALSO	3
-1,86E+18	FALSO	High	IsMasterPr	AAEL00907	1:2923567	0	11	FALSO	7
4,90E+18	FALSO	High	IsMasterPr	AAEL0119C	3:3376580	0	25	FALSO	5
6,29E+18	FALSO	High	IsMasterPr	AAEL01299	3:2972884	0	50	FALSO	7
-8,93E+18	FALSO	High	IsMasterPr	AAEL00288	1:1082884	0	13	FALSO	7
-6,71E+18	FALSO	High	IsMasterPr	AAEL01503	1:2898152	0	31	FALSO	6
-4,37E+18	FALSO	High	IsMasterPr	AAEL01069	2:1761987	0	13	FALSO	9
-3,73E+17	FALSO	High	IsMasterPr	AAEL01424	2:3627197	0	28	FALSO	10
-3,30E+18	FALSO	High	IsMasterPr	AAEL00786	3:3819391	0	31	FALSO	5
-8,06E+18	FALSO	High	IsMasterPr	AAEL02443	3:2032140	0	21	FALSO	6
1,12E+18	FALSO	High	IsMasterPr	AAEL02746	2:1873562	0	8	FALSO	6
-6,92E+18	FALSO	High	IsMasterPr	AAEL00422	3:1174348	0	14	FALSO	8
8,13E+18	FALSO	High	IsMasterPr	AAEL01815	2:3624356	0	17	FALSO	6
3,97E+18	FALSO	High	IsMasterPr	AAEL00543	1:8017214	0	13	FALSO	9
-6,84E+18	FALSO	High	IsMasterPr	AAEL02382	1:2721745	0	13	FALSO	8
-7,92E+18	FALSO	High	IsMasterPr	AAEL0054C	1:2468369	0	14	FALSO	5
2,21E+18	FALSO	High	IsMasterPr	AAEL00682	1:5302241	0	28	FALSO	5
-4,35E+17	FALSO	High	IsMasterPr	AAEL00261	3:2572846	0	12	FALSO	4
2,42E+18	FALSO	High	IsMasterPr	AAEL01305	3:3104925	0	21	FALSO	5
9,95E+17	FALSO	High	IsMasterPr	AAEL02143	1:1421646	0	20	FALSO	6
-1,86E+18	FALSO	High	IsMasterPr	AAEL02435	1:2052282	0	9	FALSO	4
1,53E+18	FALSO	High	IsMasterPr	AAEL00095	3:3801583	0	29	FALSO	6
6,13E+18	FALSO	High	IsMasterPr	AAEL0036C	1:6993330	0	21	FALSO	4
-8,78E+18	FALSO	High	IsMasterPr	AAEL01047	1:2041186	0	24	FALSO	6
1,41E+18	FALSO	High	IsMasterPr	AAEL00104	2:1017835	0	17	FALSO	5
-5,30E+18	FALSO	High	IsMasterPr	AAEL00454	1:1449439	0	14	FALSO	5
6,08E+18	FALSO	High	IsMasterPr	AAEL00151	3:2634096	0	12	FALSO	8
7,49E+18	FALSO	High	IsMasterPr	AAEL00179	2:3744619	0	34	FALSO	6
8,65E+17	FALSO	High	IsMasterPr	AAEL00923	3:3567345	0	16	FALSO	6

-4,55E+18	FALSO	High	IsMasterPr	AAEL01050	2:9105251	0	38	FALSO	5
-7,78E+18	FALSO	High	IsMasterPr	AAEL00753	2:3272199	0	21	FALSO	9
-1,09E+18	FALSO	High	IsMasterPr	AAEL00430	2:2930951	0	16	FALSO	4
-2,59E+18	FALSO	High	IsMasterPr	AAEL00434	3:3680979	0	11	FALSO	6
-2,53E+18	FALSO	High	IsMasterPr	AAEL01353	2:2461611	0	12	FALSO	10
3,39E+18	FALSO	High	IsMasterPr	AAEL02023	1:6237353	0	28	FALSO	6
4,27E+18	FALSO	High	IsMasterPr	AAEL01139	3:2016603	0	14	FALSO	7
-6,58E+17	FALSO	High	IsMasterPr	AAEL01286	1:2220368	0	9	FALSO	3
-2,29E+17	FALSO	High	IsMasterPr	AAEL00075	3:2456763	0	30	FALSO	5
-1,02E+17	FALSO	High	IsMasterPr	AAEL01297	2:1247398	0	41	FALSO	4
-2,62E+18	FALSO	High	IsMasterPr	AAEL02786	2:1261415	0	9	FALSO	5
8,33E+18	FALSO	High	IsMasterPr	AAEL00045	3:1868085	0	35	FALSO	4
-3,78E+18	FALSO	High	IsMasterPr	AAEL02809	1:2074775	0	20	FALSO	6
1,93E+18	FALSO	High	IsMasterPr	AAEL00737	2:2338529	0	23	FALSO	6
8,65E+18	FALSO	High	IsMasterPr	AAEL01953	1:2676329	0	12	FALSO	7
-7,35E+17	FALSO	High	IsMasterPr	AAEL01180	3:3608705	0	21	FALSO	5
5,48E+18	FALSO	High	IsMasterPr	AAEL00290	1:1083993	0	12	FALSO	8
-6,19E+17	FALSO	High	IsMasterPr	AAEL01231	2:1029061	0	56	FALSO	5
3,88E+18	FALSO	High	IsMasterPr	AAEL00691	3:1439041	0	30	FALSO	7
3,04E+18	FALSO	High	IsMasterPr	AAEL02905	3:2868594	0	6	FALSO	11
8,41E+18	FALSO	High	IsMasterPr	AAEL00915	1:1122417	0	31	FALSO	3
6,38E+18	FALSO	High	IsMasterPr	AAEL00824	3:5988385	0	33	FALSO	6
-6,99E+18	FALSO	High	IsMasterPr	AAEL02763	2:1991044	0	34	FALSO	8
1,80E+18	FALSO	High	IsMasterPr	AAEL00124	2:1541354	0	21	FALSO	6
-4,93E+17	FALSO	High	IsMasterPr	AAEL00207	2:3564894	0	15	FALSO	4
6,64E+18	FALSO	High	IsMasterPr	AAEL00018	3:2159916	0	28	FALSO	7
5,49E+18	FALSO	High	IsMasterPr	AAEL02095	3:6360344	0	11	FALSO	6
-2,35E+18	FALSO	High	IsMasterPr	AAEL00318	1:1901810	0	28	FALSO	4
-2,84E+18	FALSO	High	IsMasterPr	AAEL00081	3:1203791	0	16	FALSO	7
2,17E+18	FALSO	High	IsMasterPr	AAEL00325	2:3665624	0	41	FALSO	5
3,75E+16	FALSO	High	IsMasterPr	AAEL02152	1:2102719	0	9	FALSO	6
8,16E+18	FALSO	High	IsMasterPr	AAEL00260	3:2571644	0	31	FALSO	4
-8,89E+15	FALSO	High	IsMasterPr	AAEL00614	3:6856936	0	12	FALSO	9
2,78E+18	FALSO	High	IsMasterPr	AAEL02681	3:2162475	0	13	FALSO	6
4,09E+18	FALSO	High	IsMasterPr	AAEL01993	2:5762370	0	11	FALSO	7
-3,21E+18	FALSO	High	IsMasterPr	AAEL00461	1:3066738	0	20	FALSO	9
4,82E+17	FALSO	High	IsMasterPr	AAEL00072	2:5319852	0	26	FALSO	5
1,30E+18	FALSO	High	IsMasterPr	AAEL00360	1:6918967	0	15	FALSO	4
-4,72E+18	FALSO	High	IsMasterPr	AAEL01697	3:2968040	0	21	FALSO	5
4,91E+18	FALSO	High	IsMasterPr	AAEL01192	3:3511361	0	10	FALSO	6
-6,34E+18	FALSO	High	IsMasterPr	AAEL00244	3:1275857	0	38	FALSO	5
6,84E+18	FALSO	High	IsMasterPr	AAEL01237	2:1579580	0	30	FALSO	5
-5,20E+18	FALSO	High	IsMasterPr	AAEL00447	3:1097074	0	13	FALSO	5
2,90E+18	FALSO	High	IsMasterPr	AAEL02099	2:2646331	0	37	FALSO	4
-4,20E+18	FALSO	High	IsMasterPr	AAEL00098	2:1048709	0	20	FALSO	4
-7,83E+18	FALSO	High	IsMasterPr	AAEL00706	3:2985559	0	30	FALSO	3
-5,90E+17	FALSO	High	IsMasterPr	AAEL02098	NIGP01001	0	43	FALSO	4
-6,78E+18	FALSO	High	IsMasterPr	AAEL01242	2:1226813	0	12	FALSO	10
-8,94E+18	FALSO	High	IsMasterPr	AAEL01830	3:8096985	0	22	FALSO	6
-8,87E+18	FALSO	High	IsMasterPr	AAEL00059	2:4080194	0	7	FALSO	7

8,39E+18	FALSO	High	IsMasterPr	AAEL01752	2:1789247	0	7	FALSO	4
-5,15E+18	FALSO	High	IsMasterPr	AAEL00077	3:2426837	0	22	FALSO	6
1,89E+17	FALSO	High	IsMasterPr	AAEL01165	3:2183559	0	28	FALSO	2
5,31E+18	FALSO	High	IsMasterPr	AAEL01165	3:2184120	0	9	FALSO	3
-2,76E+18	FALSO	High	IsMasterPr	AAEL00074	2:5535843	0	37	FALSO	5
-6,70E+18	FALSO	High	IsMasterPr	AAEL01946	1:2761748	0	4	FALSO	7
-3,82E+18	FALSO	High	IsMasterPr	AAEL00652	2:4691313	0	31	FALSO	6
4,17E+18	FALSO	High	IsMasterPr	AAEL00410	3:4017160	0	39	FALSO	7
4,74E+18	FALSO	High	IsMasterPr	AAEL02128	1:2131192	0	7	FALSO	5
-8,30E+18	FALSO	High	IsMasterPr	AAEL00018	3:2130293	0	39	FALSO	2
-4,70E+18	FALSO	High	IsMasterPr	AAEL01993	3:1730368	0	12	FALSO	7
8,47E+18	FALSO	High	IsMasterPr	AAEL00318	1:1924036	0	33	FALSO	7
-4,79E+18	FALSO	High	IsMasterPr	AAEL00533	2:2711055	0	18	FALSO	9
-8,90E+18	FALSO	High	IsMasterPr	AAEL02482	2:4288440	0	20	FALSO	1
5,52E+18	FALSO	High	IsMasterPr	AAEL01286	1:2220740	0	32	FALSO	6
-8,61E+18	FALSO	High	IsMasterPr	AAEL00414	2:2559079	0	37	FALSO	8
7,14E+18	FALSO	High	IsMasterPr	AAEL01382	1:1582445	0	30	FALSO	6
3,29E+18	FALSO	High	IsMasterPr	AAEL02161	1:1284844	0	23	FALSO	8
1,48E+18	FALSO	High	IsMasterPr	AAEL00164	2:2990378	0	16	FALSO	5
-3,85E+18	FALSO	High	IsMasterPr	AAEL00643	2:4395531	0	40	FALSO	6
8,23E+18	FALSO	High	IsMasterPr	AAEL00577	3:1143701	0	31	FALSO	5
7,29E+18	FALSO	High	IsMasterPr	AAEL00240	1:1773680	0	8	FALSO	6
3,47E+18	FALSO	High	IsMasterPr	AAEL01294	3:2061692	0	15	FALSO	3
6,32E+18	FALSO	High	IsMasterPr	AAEL02307	3:1098804	0	46	FALSO	4
7,25E+18	FALSO	High	IsMasterPr	AAEL00193	3:2269149	0	20	FALSO	7
-2,96E+18	FALSO	High	IsMasterPr	AAEL00253	1:2262604	0	19	FALSO	9
-4,12E+18	FALSO	High	IsMasterPr	AAEL00213	3:1776718	0	24	FALSO	4
3,42E+18	FALSO	High	IsMasterPr	AAEL02471	2:1913798	0	38	FALSO	6
6,90E+18	FALSO	High	IsMasterPr	AAEL01423	2:4064517	0	18	FALSO	5
1,58E+17	FALSO	High	IsMasterPr	AAEL01428	2:3998820	0	25	FALSO	4
-7,73E+17	FALSO	High	IsMasterPr	AAEL01377	2:2776473	0	21	FALSO	4
4,26E+18	FALSO	High	IsMasterPr	AAEL00653	2:5836177	0	10	FALSO	6
4,44E+18	FALSO	High	IsMasterPr	AAEL00932	1:5537203	0	11	FALSO	4
-7,58E+18	FALSO	High	IsMasterPr	AAEL01525	2:4024027	0	13	FALSO	5
8,82E+18	FALSO	High	IsMasterPr	AAEL01332	3:7476961	0	27	FALSO	3
-5,60E+18	FALSO	High	IsMasterPr	AAEL01360	1:2677050	0	6	FALSO	5
-6,33E+18	FALSO	High	IsMasterPr	AAEL02055	1:3242143	0	15	FALSO	8
1,81E+18	FALSO	High	IsMasterPr	AAEL00703	2:2244461	0	23	FALSO	4
7,18E+18	FALSO	High	IsMasterPr	AAEL00298	3:4000557	0	11	FALSO	7
-9,09E+18	FALSO	High	IsMasterPr	AAEL01048	1:2783969	0	7	FALSO	4
-1,44E+18	FALSO	High	IsMasterPr	AAEL00104	3:5520839	0	22	FALSO	7
-7,54E+18	FALSO	High	IsMasterPr	AAEL00360	1:6993033	0	22	FALSO	6
-6,77E+18	FALSO	High	IsMasterPr	AAEL00521	2:1138063	0	31	FALSO	3
-6,19E+18	FALSO	High	IsMasterPr	AAEL00566	2:1322979	0	23	FALSO	6
-5,40E+18	FALSO	High	IsMasterPr	AAEL00696	2:3987197	0	24	FALSO	5
8,67E+18	FALSO	High	IsMasterPr	AAEL02719	1:3078205	0	17	FALSO	7
-8,34E+18	FALSO	High	IsMasterPr	AAEL01969	2:2382689	0	28	FALSO	3
-8,06E+18	FALSO	High	IsMasterPr	AAEL00246	2:7773188	0	5	FALSO	3
6,02E+18	FALSO	High	IsMasterPr	AAEL01376	2:3449607	0	42	FALSO	5
7,01E+18	FALSO	High	IsMasterPr	AAEL02459	1:1406566	0	18	FALSO	5

5,54E+18	FALSO	High	IsMasterPr	AAEL00993	1:30755790	0	14	FALSO	10
5,78E+18	FALSO	High	IsMasterPr	AAEL02125	3:1034957	0	19	FALSO	7
3,22E+18	FALSO	High	IsMasterPr	AAEL00634	3:3967776	0	26	FALSO	3
-4,40E+18	FALSO	High	IsMasterPr	AAEL01749	3:1529507	0	6	FALSO	7
-5,80E+18	FALSO	High	IsMasterPr	AAEL01709	2:3930683	0	27	FALSO	7
5,48E+18	FALSO	High	IsMasterPr	AAEL00121	2:4133035	0	26	FALSO	7
-8,47E+18	FALSO	High	IsMasterPr	AAEL01750	1:2793231	0	25	FALSO	5
1,34E+18	FALSO	High	IsMasterPr	AAEL01015	3:1492604	0	11	FALSO	5
1,65E+17	FALSO	High	IsMasterPr	AAEL01165	3:2145925	0	21	FALSO	4
-7,08E+18	FALSO	High	IsMasterPr	AAEL01470	1:4569774	0	14	FALSO	7
-7,63E+18	FALSO	High	IsMasterPr	AAEL01371	3:3078708	0	15	FALSO	4
-9,19E+18	FALSO	High	IsMasterPr	AAEL02377	NIGP0100C	0	31	FALSO	6
-7,25E+18	FALSO	High	IsMasterPr	AAEL00750	1:1986762	0	33	FALSO	5
-8,30E+18	FALSO	High	IsMasterPr	AAEL00540	1:2462197	0	23	FALSO	5
3,80E+18	FALSO	High	IsMasterPr	AAEL00350	1:2302385	0	41	FALSO	7
-7,23E+18	FALSO	High	IsMasterPr	AAEL02513	1:7168887	0	27	FALSO	3
-5,20E+18	FALSO	High	IsMasterPr	AAEL01829	3:3402473	0	5	FALSO	4
4,50E+18	FALSO	High	IsMasterPr	AAEL02328	2:2254043	0	13	FALSO	8
4,23E+18	FALSO	High	IsMasterPr	AAEL00979	1:2760541	0	47	FALSO	5
-8,99E+18	FALSO	High	IsMasterPr	AAEL00274	2:9624842	0	12	FALSO	4
-1,86E+18	FALSO	High	IsMasterPr	AAEL00260	3:2565751	0	18	FALSO	5
6,59E+18	FALSO	High	IsMasterPr	AAEL01272	3:4236470	0	16	FALSO	5
1,26E+18	FALSO	High	IsMasterPr	AAEL00430	2:3873657	0	20	FALSO	7
8,98E+18	FALSO	High	IsMasterPr	AAEL01479	2:3243437	0	23	FALSO	5
3,76E+17	FALSO	High	IsMasterPr	AAEL00152	3:2611395	0	9	FALSO	5
5,20E+18	FALSO	High	IsMasterPr	AAEL01001	1:1942732	0	18	FALSO	2
6,47E+18	FALSO	High	IsMasterPr	AAEL00602	3:3151158	0	25	FALSO	6
-8,30E+18	FALSO	High	IsMasterPr	AAEL00130	2:4480165	0	18	FALSO	4
2,42E+16	FALSO	High	IsMasterPr	AAEL00013	1:1945664	0	48	FALSO	4
1,56E+18	FALSO	High	IsMasterPr	AAEL00462	1:3018390	0	35	FALSO	5
2,61E+18	FALSO	High	IsMasterPr	AAEL00940	3:3807288	0	21	FALSO	6
-5,85E+18	FALSO	High	IsMasterPr	AAEL02554	1:3058685	0	15	FALSO	7
-6,56E+18	FALSO	High	IsMasterPr	AAEL02597	2:1663989	0	18	FALSO	6
7,63E+17	FALSO	High	IsMasterPr	AAEL00810	3:1467442	0	12	FALSO	6
6,17E+18	FALSO	High	IsMasterPr	AAEL00648	3:1598552	0	26	FALSO	8
-8,10E+18	FALSO	High	IsMasterPr	AAEL00473	2:3793104	0	26	FALSO	4
2,62E+18	FALSO	High	IsMasterPr	AAEL01982	3:3012098	0	9	FALSO	3
-4,92E+18	FALSO	High	IsMasterPr	AAEL01120	1:3487499	0	36	FALSO	5
-2,01E+17	FALSO	High	IsMasterPr	AAEL01047	1:2040653	0	46	FALSO	3
-7,12E+18	FALSO	High	IsMasterPr	AAEL00937	3:8609242	0	30	FALSO	6
6,49E+18	FALSO	High	IsMasterPr	AAEL01350	2:1992333	0	18	FALSO	7
5,96E+18	FALSO	High	IsMasterPr	AAEL00233	2:1760699	0	19	FALSO	5
-7,25E+18	FALSO	High	IsMasterPr	AAEL00252	1:9132079	0	15	FALSO	7
-7,27E+18	FALSO	High	IsMasterPr	AAEL01739	3:2351506	0	16	FALSO	4
-7,82E+18	FALSO	High	IsMasterPr	AAEL00023	1:6560805	0	14	FALSO	6
-7,23E+18	FALSO	High	IsMasterPr	AAEL01455	2:3323390	0	35	FALSO	4
7,00E+16	FALSO	High	IsMasterPr	AAEL00456	1:1446629	0	13	FALSO	5
8,04E+18	FALSO	High	IsMasterPr	AAEL00009	3:1044732	0	29	FALSO	5
-2,74E+18	FALSO	High	IsMasterPr	AAEL00859	2:3628831	0	6	FALSO	5
5,23E+18	FALSO	High	IsMasterPr	AAEL00136	2:2057146	0	14	FALSO	5

6,83E+18	FALSO	High	IsMasterPr	AAEL00127	2:1530303	0	15	FALSO	5
6,38E+18	FALSO	High	IsMasterPr	AAEL00299	3:3890559	0	16	FALSO	2
-4,28E+18	FALSO	High	IsMasterPr	AAEL00566	2:3818831	0	49	FALSO	4
5,95E+18	FALSO	High	IsMasterPr	AAEL01986	2:1215871	0	18	FALSO	4
4,62E+18	FALSO	High	IsMasterPr	AAEL00954	3:2108109	0	9	FALSO	5
3,13E+18	FALSO	High	IsMasterPr	AAEL02450	2:1326538	0	21	FALSO	5
-7,43E+18	FALSO	High	IsMasterPr	AAEL02690	2:2324186	0	29	FALSO	5
8,68E+18	FALSO	High	IsMasterPr	AAEL02050	1:2730376	0	3	FALSO	7
-8,56E+18	FALSO	High	IsMasterPr	AAEL00797	2:4309347	0	9	FALSO	5
-8,89E+18	FALSO	High	IsMasterPr	AAEL01543	2:1331627	0	19	FALSO	4
-6,71E+18	FALSO	High	IsMasterPr	AAEL01427	1:8383369	0	30	FALSO	6
1,85E+18	FALSO	High	IsMasterPr	AAEL02600	2:6484847	0	16	FALSO	2
-5,52E+17	FALSO	High	IsMasterPr	AAEL00493	2:2596216	0	24	FALSO	8
2,49E+17	FALSO	High	IsMasterPr	AAEL02023	1:6772095	0	6	FALSO	4
4,73E+18	FALSO	High	IsMasterPr	C0R550	50S ribosor	0	44	FALSO	5
-8,87E+18	FALSO	High	IsMasterPr	AAEL01212	2:4059528	0	19	FALSO	6
5,59E+18	FALSO	High	IsMasterPr	AAEL00333	2:4003415	0	16	FALSO	5
-4,90E+18	FALSO	High	IsMasterPr	AAEL01004	1:1593634	0	21	FALSO	8
6,42E+18	FALSO	High	IsMasterPr	AAEL01387	2:1228396	0	36	FALSO	4
6,02E+18	FALSO	High	IsMasterPr	AAEL01834	2:3821244	0	5	FALSO	3
4,37E+18	FALSO	High	IsMasterPr	AAEL01216	2:2038623	0	8	FALSO	4
-1,24E+18	FALSO	High	IsMasterPr	AAEL00369	3:6510858	0	12	FALSO	4
9,14E+18	FALSO	High	IsMasterPr	AAEL01064	2:2792670	0	16	FALSO	6
-4,19E+18	FALSO	High	IsMasterPr	AAEL01736	2:4041682	0	10	FALSO	4
-1,09E+18	FALSO	High	IsMasterPr	AAEL00047	3:1856366	0	8	FALSO	2
7,61E+18	FALSO	High	IsMasterPr	AAEL01073	2:4065870	0	11	FALSO	6
-8,33E+18	FALSO	High	IsMasterPr	AAEL02791	1:4555113	0	34	FALSO	5
2,27E+18	FALSO	High	IsMasterPr	AAEL00565	2:3821186	0	17	FALSO	3
-7,48E+17	FALSO	High	IsMasterPr	AAEL00195	3:2270133	0	21	FALSO	2
-3,68E+18	FALSO	High	IsMasterPr	AAEL01439	3:2891621	0	33	FALSO	4
-1,65E+18	FALSO	High	IsMasterPr	AAEL00084	3:3627015	0	9	FALSO	4
3,42E+17	FALSO	High	IsMasterPr	AAEL02139	1:1430474	0	12	FALSO	7
7,38E+18	FALSO	High	IsMasterPr	AAEL00737	2:2333631	0	20	FALSO	6
8,84E+18	FALSO	High	IsMasterPr	AAEL00609	3:3075610	0	39	FALSO	6
1,63E+18	FALSO	High	IsMasterPr	AAEL01174	3:2907273	0	11	FALSO	2
3,64E+18	FALSO	High	IsMasterPr	AAEL00442	3:2065090	0	29	FALSO	4
-3,14E+18	FALSO	High	IsMasterPr	AAEL01349	2:1993004	0	8	FALSO	4
1,62E+18	FALSO	High	IsMasterPr	AAEL00157	2:2282099	0	38	FALSO	5
8,76E+18	FALSO	High	IsMasterPr	AAEL01231	2:1028033	0	25	FALSO	7
9,22E+18	FALSO	High	IsMasterPr	AAEL02614	1:2923644	0	18	FALSO	5
9,21E+18	FALSO	High	IsMasterPr	AAEL00846	1:5329372	0	5	FALSO	3
-1,58E+18	FALSO	High	IsMasterPr	AAEL00402	3:1659686	0	17	FALSO	5
-4,76E+18	FALSO	High	IsMasterPr	AAEL01382	2:3158128	0	18	FALSO	2
9,15E+18	FALSO	High	IsMasterPr	AAEL00386	3:3428581	0	22	FALSO	5
-5,59E+18	FALSO	High	IsMasterPr	AAEL00281	3:1293550	0	12	FALSO	3
6,55E+18	FALSO	High	IsMasterPr	A0A1S6QV	Outer surfa	0	25	FALSO	5
7,02E+18	FALSO	High	IsMasterPr	AAEL01967	2:1743591	0	20	FALSO	6
8,39E+18	FALSO	High	IsMasterPr	AAEL00017	3:2128309	0	27	FALSO	2
-6,51E+18	FALSO	High	IsMasterPr	AAEL01175	1:1476104	0	22	FALSO	9
4,13E+18	FALSO	High	IsMasterPr	AAEL00870	3:9516513	0	26	FALSO	4

-4,69E+18	FALSO	High	IsMasterPr	AAEL02172	1:1779930!	0	28	FALSO	3
5,41E+18	FALSO	High	IsMasterPr	AAEL00455	1:1449014!	0	32	FALSO	4
8,37E+18	FALSO	High	IsMasterPr	AAEL0113C	3:1402168(	0	18	FALSO	6
-6,50E+17	FALSO	High	IsMasterPr	AAEL01398	3:2305979	0	29	FALSO	3
7,91E+18	FALSO	High	IsMasterPr	AAEL01175	1:1480415	0	18	FALSO	2
3,89E+17	FALSO	High	IsMasterPr	AAEL01046	1:2047767	0	10	FALSO	4
5,37E+18	FALSO	High	IsMasterPr	AAEL00443	3:2051826	0	38	FALSO	5
6,06E+18	FALSO	High	IsMasterPr	AAEL01322	3:3408717	0	25	FALSO	6
-7,88E+18	FALSO	High	IsMasterPr	AAEL01205	2:1634003	0	15	FALSO	6
4,55E+18	FALSO	High	IsMasterPr	AAEL02253	1:454097:4	0	22	FALSO	7
-6,35E+18	FALSO	High	IsMasterPr	AAEL0028S	1:1086620	0	29	FALSO	5
3,31E+18	FALSO	High	IsMasterPr	AAEL0014S	2:2645730	0	20	FALSO	5
9,16E+18	FALSO	High	IsMasterPr	AAEL00736	2:2332322	0	31	FALSO	3
7,02E+18	FALSO	High	IsMasterPr	AAEL00906	2:2694477	0	25	FALSO	3
1,80E+18	FALSO	High	IsMasterPr	AAEL00263	2:2455155	0	21	FALSO	7
4,98E+18	FALSO	High	IsMasterPr	AAEL00142	2:4826942	0	21	FALSO	6
-3,05E+17	FALSO	High	IsMasterPr	AAEL00351	3:2637613	0	11	FALSO	6
1,77E+18	FALSO	High	IsMasterPr	AAEL00377	1:4412373(	0	30	FALSO	5
-6,63E+18	FALSO	High	IsMasterPr	AAEL00398	3:2541348	0	19	FALSO	6
7,33E+17	FALSO	High	IsMasterPr	AAEL0060S	3:3074494	0	11	FALSO	6
-7,62E+18	FALSO	High	IsMasterPr	AAEL02318	1:2086159	0	10	FALSO	5
-6,14E+18	FALSO	High	IsMasterPr	AAEL01972	2:3476308	0	10	FALSO	3
-3,92E+18	FALSO	High	IsMasterPr	AAEL01738	3:4118428	0	19	FALSO	6
7,57E+18	FALSO	High	IsMasterPr	AAEL00702	3:1713741	0	16	FALSO	5
3,50E+18	FALSO	High	IsMasterPr	AAEL00185	2:2752742	0	16	FALSO	6
5,20E+18	FALSO	High	IsMasterPr	AAEL01488	2:1045591	0	13	FALSO	2
2,32E+18	FALSO	High	IsMasterPr	AAEL01183	1:1534476	0	29	FALSO	4
-4,08E+17	FALSO	High	IsMasterPr	AAEL00815	3:9358797	0	17	FALSO	4
8,12E+18	FALSO	High	IsMasterPr	AAEL00014	1:1973117	0	19	FALSO	5
-2,43E+18	FALSO	High	IsMasterPr	AAEL00547	3:3828824	0	6	FALSO	1
-7,03E+18	FALSO	High	IsMasterPr	AAEL0146C	3:3684811	0	22	FALSO	8
8,40E+18	FALSO	High	IsMasterPr	AAEL00514	3:3467898(	0	57	FALSO	5
6,70E+17	FALSO	High	IsMasterPr	AAEL01357	2:6186352	0	13	FALSO	2
5,14E+18	FALSO	High	IsMasterPr	AAEL00991	1:2066259	0	30	FALSO	3
-2,50E+18	FALSO	High	IsMasterPr	AAEL01034	2:4037797	0	18	FALSO	7
1,02E+18	FALSO	High	IsMasterPr	AAEL01175	NIGP01001	0	22	FALSO	5
3,76E+18	FALSO	High	IsMasterPr	AAEL00206	2:3566675	0	33	FALSO	5
-8,21E+18	FALSO	High	IsMasterPr	AAEL01812	3:2784781	0	5	FALSO	4
-5,95E+17	FALSO	High	IsMasterPr	AAEL00754	2:3272465	0	20	FALSO	5
-6,27E+18	FALSO	High	IsMasterPr	AAEL01145	2:3721928(	0	22	FALSO	5
5,35E+18	FALSO	High	IsMasterPr	AAEL01208	2:1442730	0	31	FALSO	7
-7,56E+18	FALSO	High	IsMasterPr	AAEL0253S	2:4741077(	0	2	FALSO	10
5,74E+18	FALSO	High	IsMasterPr	AAEL00741	2:1263647(	0	38	FALSO	5
-2,48E+17	FALSO	High	IsMasterPr	AAEL01076	2:3049387	0	14	FALSO	7
7,24E+18	FALSO	High	IsMasterPr	AAEL02175	1:3075089(	0	21	FALSO	5
6,65E+18	FALSO	High	IsMasterPr	AAEL01006	3:1121507	0	20	FALSO	7
4,48E+18	FALSO	High	IsMasterPr	AAEL00278	3:1299782	0	20	FALSO	6
4,11E+18	FALSO	High	IsMasterPr	AAEL01976	1:1888187	0	10	FALSO	3
-3,68E+18	FALSO	High	IsMasterPr	AAEL0019C	2:3899066	0	29	FALSO	5
-4,94E+17	FALSO	High	IsMasterPr	AAEL0246S	NIGP0100C	0	6	FALSO	2

-3,13E+18	FALSO	High	IsMasterPr	AAEL00831	2:2741048	0	13	FALSO	4
5,45E+18	FALSO	High	IsMasterPr	AAEL00841	1:1736119	0	27	FALSO	5
-5,22E+18	FALSO	High	IsMasterPr	AAEL00724	2:4223836	0	37	FALSO	6
2,41E+18	FALSO	High	IsMasterPr	AAEL00571	3:2040663	0	6	FALSO	7
-4,97E+18	FALSO	High	IsMasterPr	AAEL00127	2:1530549	0	6	FALSO	2
-4,17E+18	FALSO	High	IsMasterPr	AAEL00366	2:3171156	0	13	FALSO	7
-7,20E+18	FALSO	High	IsMasterPr	AAEL02640	2:2964306	0	16	FALSO	6
6,76E+18	FALSO	High	IsMasterPr	AAEL01979	2:4297834	0	3	FALSO	8
-3,06E+18	FALSO	High	IsMasterPr	AAEL01500	2:3377866	0	29	FALSO	3
-3,22E+18	FALSO	High	IsMasterPr	AAEL01716	2:1658006	0	8	FALSO	3
8,45E+17	FALSO	High	IsMasterPr	AAEL01101	1:7383542	0	16	FALSO	5
-3,90E+18	FALSO	High	IsMasterPr	AAEL02810	3:1620626	0	37	FALSO	5
7,82E+18	FALSO	High	IsMasterPr	AAEL00528	3:3096263	0	12	FALSO	4
-1,08E+18	FALSO	High	IsMasterPr	AAEL01084	2:1276875	0	26	FALSO	3
6,26E+18	FALSO	High	IsMasterPr	AAEL01251	2:3227519	0	13	FALSO	4
-8,61E+18	FALSO	High	IsMasterPr	AAEL00336	3:8078870	0	36	FALSO	4
8,88E+18	FALSO	High	IsMasterPr	AAEL00357	3:8744008	0	35	FALSO	3
-2,96E+18	FALSO	High	IsMasterPr	AAEL00740	3:4082487	0	4	FALSO	4
-6,37E+18	FALSO	High	IsMasterPr	AAEL00440	3:2723064	0	25	FALSO	8
1,96E+18	FALSO	High	IsMasterPr	AAEL01198	1:2855315	0	12	FALSO	5
2,22E+18	FALSO	High	IsMasterPr	AAEL00252	1:8931662	0	31	FALSO	2
2,82E+18	FALSO	High	IsMasterPr	AAEL00964	1:6063536	0	12	FALSO	5
-8,30E+18	FALSO	High	IsMasterPr	AAEL01101	1:7342259	0	19	FALSO	6
-5,27E+18	FALSO	High	IsMasterPr	AAEL00002	3:1043600	0	11	FALSO	4
6,69E+18	FALSO	High	IsMasterPr	AAEL02348	NIGP0100C	0	39	FALSO	3
-1,12E+18	FALSO	High	IsMasterPr	AAEL00889	3:3372331	0	16	FALSO	6
-6,89E+18	FALSO	High	IsMasterPr	AAEL00102	3:5516219	0	21	FALSO	6
-1,02E+18	FALSO	High	IsMasterPr	AAEL00850	2:3951151	0	29	FALSO	7
9,15E+18	FALSO	High	IsMasterPr	AAEL00118	2:3077450	0	18	FALSO	4
-7,88E+18	FALSO	High	IsMasterPr	AAEL01310	3:2946430	0	7	FALSO	8
-1,58E+17	FALSO	High	IsMasterPr	AAEL01242	3:1258449	0	17	FALSO	3
-5,36E+18	FALSO	High	IsMasterPr	AAEL00237	2:3022736	0	22	FALSO	3
-8,18E+18	FALSO	High	IsMasterPr	AAEL01984	3:7407126	0	19	FALSO	5
-9,07E+18	FALSO	High	IsMasterPr	AAEL01708	2:2629205	0	19	FALSO	2
6,26E+17	FALSO	High	IsMasterPr	AAEL01418	2:3553945	0	16	FALSO	4
-6,73E+18	FALSO	High	IsMasterPr	AAEL00135	2:5698756	0	21	FALSO	3
1,33E+18	FALSO	High	IsMasterPr	AAEL00497	2:2819201	0	5	FALSO	6
-1,09E+18	FALSO	High	IsMasterPr	AAEL00419	1:1567390	0	38	FALSO	5
2,86E+18	FALSO	High	IsMasterPr	AAEL00201	2:4265690	0	16	FALSO	6
2,34E+18	FALSO	High	IsMasterPr	AAEL02626	2:4045964	0	9	FALSO	5
-2,11E+18	FALSO	High	IsMasterPr	AAEL00565	2:3817872	0	10	FALSO	2
-9,53E+17	FALSO	High	IsMasterPr	AAEL01115	3:1936108	0	35	FALSO	5
-8,70E+18	FALSO	High	IsMasterPr	AAEL01287	3:2758776	0	25	FALSO	4
-1,53E+18	FALSO	High	IsMasterPr	AAEL02900	2:3695867	0	27	FALSO	6
5,69E+18	FALSO	High	IsMasterPr	AAEL01001	2:1108821	0	22	FALSO	5
8,76E+18	FALSO	High	IsMasterPr	AAEL01417	1:2725605	0	14	FALSO	4
2,80E+18	FALSO	High	IsMasterPr	AAEL01489	2:2574242	0	30	FALSO	6
-7,28E+18	FALSO	High	IsMasterPr	AAEL01202	3:1473241	0	19	FALSO	5
9,08E+18	FALSO	High	IsMasterPr	AAEL01127	2:2260009	0	14	FALSO	4
2,75E+17	FALSO	High	IsMasterPr	AAEL00053	3:3862233	0	25	FALSO	4

8,76E+17	FALSO	High	IsMasterPr	AAEL00186	2:27519620	0	67	FALSO	3
8,29E+18	FALSO	High	IsMasterPr	AAEL02022	NIGP01000	0	41	FALSO	4
-5,71E+17	FALSO	High	IsMasterPr	AAEL01473	2:3267561	0	5	FALSO	4
-2,77E+18	FALSO	High	IsMasterPr	AAEL00583	1:2239786	0	9	FALSO	3
2,64E+18	FALSO	High	IsMasterPr	AAEL00423	1:1052244	0	6	FALSO	3
2,16E+18	FALSO	High	IsMasterPr	AAEL01394	2:3522083	0	44	FALSO	4
-6,24E+18	FALSO	High	IsMasterPr	AAEL01201	3:3080021	0	29	FALSO	4
-6,12E+18	FALSO	High	IsMasterPr	AAEL00748	1:2164002	0	13	FALSO	6
-9,86E+17	FALSO	High	IsMasterPr	AAEL01951	1:2709918	0	6	FALSO	4
-7,95E+18	FALSO	High	IsMasterPr	AAEL00589	2:6615417	0	34	FALSO	3
-5,71E+18	FALSO	High	IsMasterPr	AAEL01306	2:2533906	0	16	FALSO	3
9,21E+18	FALSO	High	IsMasterPr	AAEL00286	1:2560523	0	21	FALSO	5
-5,18E+18	FALSO	High	IsMasterPr	AAEL02485	2:7617198	0	33	FALSO	4
4,24E+18	FALSO	High	IsMasterPr	AAEL02134	1:7350428	0	15	FALSO	4
-2,88E+18	FALSO	High	IsMasterPr	AAEL00332	2:4001934	0	12	FALSO	3
-8,84E+18	FALSO	High	IsMasterPr	AAEL01365	1:3846547	0	15	FALSO	5
-1,02E+18	FALSO	High	IsMasterPr	AAEL02907	1:8348377	0	22	FALSO	5
4,26E+18	FALSO	High	IsMasterPr	AAEL02436	3:2800592	0	26	FALSO	2
2,14E+18	FALSO	High	IsMasterPr	AAEL00335	2:4613917	0	28	FALSO	3
1,32E+18	FALSO	High	IsMasterPr	AAEL02057	2:1391234	0	14	FALSO	7
1,59E+18	FALSO	High	IsMasterPr	AAEL02275	2:1080646	0	17	FALSO	5
6,78E+18	FALSO	High	IsMasterPr	AAEL00498	2:2817814	0	20	FALSO	3
6,16E+18	FALSO	High	IsMasterPr	AAEL00908	2:3866911	0	22	FALSO	6
6,30E+18	FALSO	High	IsMasterPr	AAEL00562	3:2115327	0	15	FALSO	5
-3,13E+18	FALSO	High	IsMasterPr	AAEL01323	3:2800605	0	12	FALSO	4
6,89E+18	FALSO	High	IsMasterPr	AAEL00316	1:1930318	0	11	FALSO	4
2,86E+17	FALSO	High	IsMasterPr	AAEL00644	2:4386746	0	29	FALSO	4
-3,82E+18	FALSO	High	IsMasterPr	AAEL00901	1:5879894	0	7	FALSO	3
-1,90E+18	FALSO	High	IsMasterPr	AAEL01251	2:7614577	0	13	FALSO	3
6,02E+18	FALSO	High	IsMasterPr	AAEL00080	3:3638327	0	15	FALSO	7
8,11E+18	FALSO	High	IsMasterPr	AAEL00062	2:2851334	0	38	FALSO	5
5,97E+18	FALSO	High	IsMasterPr	AAEL01403	1:1429580	0	13	FALSO	3
-5,45E+18	FALSO	High	IsMasterPr	AAEL00224	2:4307367	0	22	FALSO	6
-8,51E+18	FALSO	High	IsMasterPr	AAEL00432	3:3657202	0	13	FALSO	4
-2,85E+18	FALSO	High	IsMasterPr	AAEL00922	2:1126400	0	27	FALSO	5
6,65E+18	FALSO	High	IsMasterPr	AAEL00505	1:2956279	0	43	FALSO	7
5,21E+17	FALSO	High	IsMasterPr	AAEL01989	2:1113980	0	7	FALSO	4
-2,28E+18	FALSO	High	IsMasterPr	AAEL01971	2:4723372	0	28	FALSO	3
6,34E+18	FALSO	High	IsMasterPr	AAEL00075	2:2661638	0	33	FALSO	7
-6,33E+18	FALSO	High	IsMasterPr	AAEL02526	3:2683534	0	14	FALSO	5
2,83E+18	FALSO	High	IsMasterPr	AAEL00128	2:1551926	0	6	FALSO	4
5,51E+18	FALSO	High	IsMasterPr	AAEL00510	2:1726582	0	25	FALSO	5
8,20E+18	FALSO	High	IsMasterPr	AAEL00446	3:2122800	0	27	FALSO	3
-5,89E+18	FALSO	High	IsMasterPr	AAEL00003	3:1050574	0	12	FALSO	2
-9,09E+18	FALSO	High	IsMasterPr	AAEL00487	1:2881966	0	20	FALSO	5
-2,60E+18	FALSO	High	IsMasterPr	AAEL01066	2:1359346	0	13	FALSO	5
2,37E+18	FALSO	High	IsMasterPr	AAEL00233	1:1194936	0	22	FALSO	5
2,69E+18	FALSO	High	IsMasterPr	AAEL01425	1:7249067	0	25	FALSO	2
-8,60E+18	FALSO	High	IsMasterPr	AAEL02138	1:1217908	0	26	FALSO	4
1,23E+18	FALSO	High	IsMasterPr	AAEL00002	3:1050981	0	19	FALSO	5

-4,64E+18	FALSO	High	IsMasterPr	AAEL01494	3:2042183	0	21	FALSO	4
4,80E+18	FALSO	High	IsMasterPr	AAEL00892	3:5028114	0	6	FALSO	1
-1,06E+17	FALSO	High	IsMasterPr	AAEL02907	1:8351650	0	16	FALSO	3
-1,56E+17	FALSO	High	IsMasterPr	gb:QCQ29(	ncbiId:QCC	0	1	FALSO	3
-8,98E+18	FALSO	High	IsMasterPr	AAEL00846	1:5320940	0	13	FALSO	4
-3,63E+18	FALSO	High	IsMasterPr	AAEL00701	NIGP01001	0	12	FALSO	3
7,95E+18	FALSO	High	IsMasterPr	AAEL01396	3:1042915	0	14	FALSO	3
-5,64E+18	FALSO	High	IsMasterPr	AAEL01396	3:1058267	0	23	FALSO	4
8,35E+18	FALSO	High	IsMasterPr	AAEL01048	1:2786948	0	28	FALSO	8
-2,40E+18	FALSO	High	IsMasterPr	AAEL0108C	3:5177199	0	18	FALSO	3
-4,00E+18	FALSO	High	IsMasterPr	AAEL00041	3:1836356	0	19	FALSO	4
5,33E+18	FALSO	High	IsMasterPr	AAEL00794	2:3521517	0	19	FALSO	5
-5,16E+18	FALSO	High	IsMasterPr	AAEL00636	1:1008268	0	20	FALSO	5
-3,22E+18	FALSO	High	IsMasterPr	AAEL0138C	1:1404715	0	19	FALSO	6
7,13E+18	FALSO	High	IsMasterPr	AAEL02517	2:3516563	0	25	FALSO	3
3,72E+18	FALSO	High	IsMasterPr	AAEL02105	1:7220665	0	15	FALSO	7
-5,48E+18	FALSO	High	IsMasterPr	AAEL02608	2:3666880	0	17	FALSO	6
-6,72E+18	FALSO	High	IsMasterPr	AAEL02419	2:1238117	0	26	FALSO	3
-1,98E+18	FALSO	High	IsMasterPr	AAEL00266	2:4332928	0	15	FALSO	5
-2,29E+17	FALSO	High	IsMasterPr	AAEL00278	3:1293249	0	31	FALSO	3
-4,97E+17	FALSO	High	IsMasterPr	AAEL01506	1:1532384	0	22	FALSO	2
7,13E+18	FALSO	High	IsMasterPr	AAEL00769	2:4703669	0	10	FALSO	7
4,09E+18	FALSO	High	IsMasterPr	AAEL00211	3:1769092	0	17	FALSO	5
3,69E+18	FALSO	High	IsMasterPr	AAEL01745	1:1307957	0	15	FALSO	2
5,21E+18	FALSO	High	IsMasterPr	AAEL00277	3:1448855	0	6	FALSO	4
7,21E+18	FALSO	High	IsMasterPr	AAEL02804	2:4189853	0	25	FALSO	5
-7,87E+18	FALSO	High	IsMasterPr	AAEL01721	1:1940253	0	7	FALSO	4
-1,98E+18	FALSO	High	IsMasterPr	AAEL01013	2:2652433	0	34	FALSO	5
8,57E+18	FALSO	High	IsMasterPr	AAEL00892	1:6523096	0	31	FALSO	4
8,24E+18	FALSO	High	IsMasterPr	AAEL0064C	2:4150788	0	17	FALSO	3
-3,11E+18	FALSO	High	IsMasterPr	AAEL00771	3:2555810	0	37	FALSO	5
8,31E+18	FALSO	High	IsMasterPr	AAEL00131	3:3271089	0	17	FALSO	7
-4,04E+18	FALSO	High	IsMasterPr	AAEL00219	2:4284528	0	18	FALSO	3
-8,94E+18	FALSO	High	IsMasterPr	AAEL00908	2:3962912	0	6	FALSO	4
-2,95E+18	FALSO	High	IsMasterPr	AAEL01511	2:1277668	0	3	FALSO	2
7,26E+18	FALSO	High	IsMasterPr	AAEL00963	1:2225943	0	11	FALSO	2
-6,33E+18	FALSO	High	IsMasterPr	AAEL01223	1:1465364	0	36	FALSO	3
7,06E+18	FALSO	High	IsMasterPr	AAEL01034	2:4041195	0	15	FALSO	2
9,20E+18	FALSO	High	IsMasterPr	AAEL01484	3:1976960	0	14	FALSO	4
9,02E+18	FALSO	High	IsMasterPr	AAEL01353	2:2460564	0	22	FALSO	5
-1,43E+18	FALSO	High	IsMasterPr	AAEL01827	2:1366353	0	9	FALSO	4
9,06E+18	FALSO	High	IsMasterPr	AAEL00302	1:1295291	0	7	FALSO	4
-2,74E+18	FALSO	High	IsMasterPr	AAEL01816	2:1606639	0	6	FALSO	4
-1,43E+17	FALSO	High	IsMasterPr	AAEL00546	2:2965041	0	14	FALSO	5
-2,93E+18	FALSO	High	IsMasterPr	AAEL00905	2:2703306	0	35	FALSO	3
-8,94E+18	FALSO	High	IsMasterPr	AAEL02451	NIGP0100C	0	12	FALSO	3
7,74E+18	FALSO	High	IsMasterPr	Q73H52	Uncharacte	0	42	FALSO	4
4,64E+18	FALSO	High	IsMasterPr	AAEL00248	3:3381924	0	14	FALSO	5
-6,50E+18	FALSO	High	IsMasterPr	AAEL00541	1:2471897	0	10	FALSO	2
7,05E+18	FALSO	High	IsMasterPr	AAEL00552	1:9027989	0	14	FALSO	5

7,69E+18	FALSO	High	IsMasterPr	AAEL00987	3:1222116	0	23	FALSO	6
5,36E+18	FALSO	High	IsMasterPr	AAEL02142	NIGP01001	0	21	FALSO	3
-5,99E+18	FALSO	High	IsMasterPr	AAEL01384	1:1788627	0	59	FALSO	6
-3,02E+17	FALSO	High	IsMasterPr	AAEL00674	2:8511088	0	32	FALSO	4
9,09E+18	FALSO	High	IsMasterPr	AAEL01196	3:4554235	0	25	FALSO	3
-6,40E+18	FALSO	High	IsMasterPr	AAEL0196	2:1042499	0	11	FALSO	5
-5,24E+18	FALSO	High	IsMasterPr	AAEL01433	2:2278572	0	29	FALSO	4
-4,17E+18	FALSO	High	IsMasterPr	AAEL00467	3:2467152	0	53	FALSO	3
-7,77E+18	FALSO	High	IsMasterPr	AAEL01953	1:8094731	0	3	FALSO	4
-7,93E+17	FALSO	High	IsMasterPr	AAEL00172	2:4432244	0	8	FALSO	4
9,33E+16	FALSO	High	IsMasterPr	AAEL0244	1:2216351	0	14	FALSO	4
-2,15E+18	FALSO	High	IsMasterPr	AAEL0093	3:3379981	0	12	FALSO	1
2,98E+18	FALSO	High	IsMasterPr	AAEL00926	2:2682862	0	7	FALSO	6
-5,00E+18	FALSO	High	IsMasterPr	AAEL00632	3:2724893	0	9	FALSO	4
2,15E+18	FALSO	High	IsMasterPr	AAEL00292	1:8594666	0	18	FALSO	4
4,96E+18	FALSO	High	IsMasterPr	AAEL01077	2:3053096	0	33	FALSO	3
6,95E+18	FALSO	High	IsMasterPr	AAEL01985	2:3339207	0	13	FALSO	5
5,97E+18	FALSO	High	IsMasterPr	AAEL0031	2:4409717	0	27	FALSO	9
-2,38E+18	FALSO	High	IsMasterPr	AAEL02108	3:1044065	0	32	FALSO	4
3,07E+18	FALSO	High	IsMasterPr	AAEL00875	3:3260236	0	10	FALSO	4
2,77E+18	FALSO	High	IsMasterPr	AAEL01145	2:1870289	0	23	FALSO	4
2,73E+18	FALSO	High	IsMasterPr	AAEL00562	1:4732659	0	14	FALSO	2
6,18E+18	FALSO	High	IsMasterPr	M4GQ98	Outer surfa	0	21	FALSO	2
-3,89E+18	FALSO	High	IsMasterPr	AAEL0250	3:2938364	0	8	FALSO	4
-5,95E+18	FALSO	High	IsMasterPr	AAEL01226	2:3842105	0	9	FALSO	4
5,33E+18	FALSO	High	IsMasterPr	AAEL01368	1:6464666	0	18	FALSO	4
-4,60E+18	FALSO	High	IsMasterPr	AAEL00427	2:2936800	0	5	FALSO	1
3,31E+18	FALSO	High	IsMasterPr	AAEL00807	1:1542445	0	19	FALSO	4
2,10E+18	FALSO	High	IsMasterPr	AAEL00064	3:3952184	0	14	FALSO	5
3,04E+18	FALSO	High	IsMasterPr	AAEL02228	1:2844436	0	19	FALSO	7
-6,94E+18	FALSO	High	IsMasterPr	AAEL01068	2:4181344	0	23	FALSO	5
6,34E+18	FALSO	High	IsMasterPr	AAEL01866	MT:2903:3	0	16	FALSO	2
-2,38E+18	FALSO	High	IsMasterPr	AAEL01387	2:4277087	0	5	FALSO	2
-2,61E+18	FALSO	High	IsMasterPr	AAEL00452	2:3101923	0	18	FALSO	4
3,57E+18	FALSO	High	IsMasterPr	AAEL0096	2:1691993	0	8	FALSO	3
-1,73E+18	FALSO	High	IsMasterPr	AAEL01332	3:3414276	0	15	FALSO	3
-3,32E+18	FALSO	High	IsMasterPr	AAEL00125	2:1519715	0	11	FALSO	5
6,91E+18	FALSO	High	IsMasterPr	AAEL02531	NIGP01001	0	12	FALSO	3
-6,84E+18	FALSO	High	IsMasterPr	AAEL00534	2:2713861	0	14	FALSO	6
-8,91E+18	FALSO	High	IsMasterPr	AAEL0104	1:2684569	0	8	FALSO	5
9,13E+18	FALSO	High	IsMasterPr	AAEL00852	1:6429449	0	9	FALSO	4
3,30E+18	FALSO	High	IsMasterPr	AAEL00074	NIGP01002	0	13	FALSO	3
7,27E+18	FALSO	High	IsMasterPr	AAEL00927	3:1994534	0	8	FALSO	3
1,24E+18	FALSO	High	IsMasterPr	AAEL0261	2:2766256	0	4	FALSO	5
5,27E+18	FALSO	High	IsMasterPr	AAEL00875	3:3269801	0	30	FALSO	4
2,82E+18	FALSO	High	IsMasterPr	AAEL0182	3:3599001	0	6	FALSO	7
-4,85E+18	FALSO	High	IsMasterPr	AAEL0274	2:2185243	0	14	FALSO	3
-2,25E+18	FALSO	High	IsMasterPr	AAEL00137	2:2049796	0	14	FALSO	3
6,93E+18	FALSO	High	IsMasterPr	AAEL02602	1:8137015	0	9	FALSO	4
4,44E+18	FALSO	High	IsMasterPr	AAEL00092	3:3355152	0	7	FALSO	4

-8,26E+18	FALSO	High	IsMasterPr	AAEL01956	2:4533703	0	15	FALSO	6
-9,12E+17	FALSO	High	IsMasterPr	AAEL00212	3:1770140	0	14	FALSO	5
-6,29E+18	FALSO	High	IsMasterPr	AAEL01146	3:1425982	0	2	FALSO	3
5,92E+18	FALSO	High	IsMasterPr	AAEL01871	3:3136377	0	11	FALSO	4
-6,03E+17	FALSO	High	IsMasterPr	AAEL01349	2:2378816	0	21	FALSO	4
-4,21E+18	FALSO	High	IsMasterPr	AAEL01495	2:2228411	0	16	FALSO	5
-7,98E+18	FALSO	High	IsMasterPr	AAEL02138	1:1217908	0	27	FALSO	4
-2,03E+17	FALSO	High	IsMasterPr	AAEL00376	1:2480636	0	16	FALSO	4
6,97E+18	FALSO	High	IsMasterPr	AAEL01405	1:1483330	0	8	FALSO	4
1,69E+18	FALSO	High	IsMasterPr	AAEL00259	3:2565089	0	17	FALSO	5
-1,10E+18	FALSO	High	IsMasterPr	AAEL00982	3:2030308	0	3	FALSO	5
4,04E+18	FALSO	High	IsMasterPr	AAEL00935	2:4516405	0	34	FALSO	5
-2,99E+18	FALSO	High	IsMasterPr	AAEL00009	3:1028585	0	10	FALSO	2
-4,15E+18	FALSO	High	IsMasterPr	AAEL01116	2:1015272	0	14	FALSO	4
-3,42E+18	FALSO	High	IsMasterPr	AAEL02449	1:2222133	0	11	FALSO	4
2,46E+17	FALSO	High	IsMasterPr	AAEL00337	3:8080376	0	10	FALSO	5
-6,23E+18	FALSO	High	IsMasterPr	AAEL00328	3:3303860	0	6	FALSO	3
-1,93E+18	FALSO	High	IsMasterPr	AAEL00776	1:7125835	0	36	FALSO	4
-1,45E+18	FALSO	High	IsMasterPr	AAEL01420	3:3015541	0	24	FALSO	4
-9,16E+17	FALSO	High	IsMasterPr	AAEL02756	3:1385633	0	6	FALSO	1
-7,39E+18	FALSO	High	IsMasterPr	AAEL00139	2:4773641	0	12	FALSO	4
5,00E+18	FALSO	High	IsMasterPr	AAEL00904	3:4610325	0	17	FALSO	3
-5,82E+18	FALSO	High	IsMasterPr	AAEL01007	3:2092066	0	20	FALSO	5
-8,93E+18	FALSO	High	IsMasterPr	AAEL00135	3:4773766	0	20	FALSO	3
8,63E+18	FALSO	High	IsMasterPr	AAEL00267	2:4338866	0	18	FALSO	4
1,48E+18	FALSO	High	IsMasterPr	AAEL00304	1:1313728	0	12	FALSO	2
-4,77E+18	FALSO	High	IsMasterPr	AAEL01942	3:2759393	0	4	FALSO	4
-2,15E+18	FALSO	High	IsMasterPr	AAEL01108	2:2352554	0	41	FALSO	4
-7,49E+18	FALSO	High	IsMasterPr	AAEL02495	1:8867247	0	4	FALSO	2
7,84E+18	FALSO	High	IsMasterPr	AAEL00785	2:4607315	0	12	FALSO	4
5,82E+18	FALSO	High	IsMasterPr	AAEL00810	3:1497056	0	1	FALSO	5
6,57E+18	FALSO	High	IsMasterPr	AAEL00097	3:3802144	0	10	FALSO	2
-6,16E+18	FALSO	High	IsMasterPr	AAEL02708	1:3036684	0	6	FALSO	2
-1,55E+18	FALSO	High	IsMasterPr	AAEL00046	3:1841947	0	18	FALSO	5
3,84E+18	FALSO	High	IsMasterPr	AAEL02218	2:1342303	0	8	FALSO	3
7,91E+18	FALSO	High	IsMasterPr	AAEL00133	3:4896339	0	18	FALSO	2
-5,88E+18	FALSO	High	IsMasterPr	AAEL00027	3:2650039	0	14	FALSO	2
-2,01E+18	FALSO	High	IsMasterPr	AAEL00608	1:1840723	0	5	FALSO	4
-1,80E+18	FALSO	High	IsMasterPr	AAEL00109	1:2991773	0	20	FALSO	2
4,24E+17	FALSO	High	IsMasterPr	AAEL01017	3:1488294	0	29	FALSO	4
-6,29E+18	FALSO	High	IsMasterPr	AAEL00722	2:4213965	0	6	FALSO	5
3,75E+18	FALSO	High	IsMasterPr	AAEL01977	2:3118226	0	5	FALSO	5
5,88E+17	FALSO	High	IsMasterPr	AAEL00932	1:5528992	0	21	FALSO	4
-1,91E+18	FALSO	High	IsMasterPr	AAEL02643	1:1220013	0	5	FALSO	1
8,38E+18	FALSO	High	IsMasterPr	AAEL01027	3:6179702	0	19	FALSO	7
-8,19E+17	FALSO	High	IsMasterPr	AAEL00391	1:2522382	0	16	FALSO	4
-1,53E+18	FALSO	High	IsMasterPr	AAEL01251	2:7633039	0	28	FALSO	3
8,99E+17	FALSO	High	IsMasterPr	AAEL00187	2:2743833	0	15	FALSO	5
-5,76E+18	FALSO	High	IsMasterPr	AAEL00100	1:2101067	0	10	FALSO	4
-6,71E+18	FALSO	High	IsMasterPr	AAEL01441	1:2868845	0	14	FALSO	4

-3,22E+18	FALSO	High	IsMasterPr	gb:APH116	ncbiId:APH	0	2	FALSO	2
-5,23E+18	FALSO	High	IsMasterPr	AAEL00267	2:4336273	0	24	FALSO	3
-5,79E+18	FALSO	High	IsMasterPr	AAEL00443	3:2043150	0	16	FALSO	6
-2,42E+17	FALSO	High	IsMasterPr	AAEL00301	3:3992727	0	28	FALSO	6
6,88E+18	FALSO	High	IsMasterPr	AAEL00735	3:1231500	0	11	FALSO	2
6,94E+18	FALSO	High	IsMasterPr	AAEL0066	3:3532427	0	47	FALSO	5
-4,77E+18	FALSO	High	IsMasterPr	AAEL00617	3:1417077	0	27	FALSO	3
3,90E+18	FALSO	High	IsMasterPr	AAEL0270	2:3516496	0	11	FALSO	2
3,99E+18	FALSO	High	IsMasterPr	AAEL00402	3:1663721	0	15	FALSO	4
-3,46E+18	FALSO	High	IsMasterPr	AAEL0001	1:1973440	0	16	FALSO	3
-3,02E+18	FALSO	High	IsMasterPr	AAEL00615	3:6869541	0	19	FALSO	6
-3,09E+18	FALSO	High	IsMasterPr	AAEL0066	2:4628433	0	18	FALSO	5
4,14E+18	FALSO	High	IsMasterPr	AAEL01364	2:3242696	0	16	FALSO	3
1,64E+18	FALSO	High	IsMasterPr	AAEL02724	2:4224544	0	11	FALSO	5
3,71E+18	FALSO	High	IsMasterPr	AAEL01334	2:1356744	0	24	FALSO	3
-4,35E+18	FALSO	High	IsMasterPr	AAEL01067	2:1364047	0	11	FALSO	4
-5,50E+18	FALSO	High	IsMasterPr	AAEL01264	3:2840688	0	9	FALSO	3
3,96E+18	FALSO	High	IsMasterPr	AAEL01702	2:3090202	0	7	FALSO	5
4,46E+18	FALSO	High	IsMasterPr	AAEL00062	2:2850670	0	6	FALSO	3
-4,12E+18	FALSO	High	IsMasterPr	AAEL00321	1:2916248	0	11	FALSO	5
7,53E+18	FALSO	High	IsMasterPr	AAEL01364	2:3242872	0	15	FALSO	3
1,34E+18	FALSO	High	IsMasterPr	AAEL0102	2:3594273	0	17	FALSO	1
3,07E+18	FALSO	High	IsMasterPr	AAEL00671	2:8490229	0	19	FALSO	6
-1,63E+18	FALSO	High	IsMasterPr	AAEL01247	2:2191471	0	8	FALSO	4
-1,13E+18	FALSO	High	IsMasterPr	AAEL02814	1:9228964	0	9	FALSO	3
8,51E+18	FALSO	High	IsMasterPr	AAEL0087	2:1988356	0	28	FALSO	3
-7,65E+18	FALSO	High	IsMasterPr	AAEL0194	3:1157153	0	5	FALSO	4
-7,29E+18	FALSO	High	IsMasterPr	AAEL0181	3:1727362	0	7	FALSO	4
-7,68E+18	FALSO	High	IsMasterPr	AAEL00432	3:5088519	0	6	FALSO	6
5,33E+18	FALSO	High	IsMasterPr	AAEL00622	2:4907414	0	35	FALSO	4
8,82E+18	FALSO	High	IsMasterPr	AAEL00573	2:4372624	0	11	FALSO	4
-8,24E+16	FALSO	High	IsMasterPr	AAEL00988	3:1222344	0	17	FALSO	3
8,17E+18	FALSO	High	IsMasterPr	AAEL02301	NIGP01002	0	25	FALSO	4
6,89E+18	FALSO	High	IsMasterPr	AAEL01181	3:1132770	0	10	FALSO	6
1,29E+17	FALSO	High	IsMasterPr	AAEL00642	2:4144307	0	19	FALSO	3
-2,60E+18	FALSO	High	IsMasterPr	AAEL0040	1:1359737	0	10	FALSO	6
6,71E+18	FALSO	High	IsMasterPr	AAEL02901	3:1195419	0	13	FALSO	2
-3,89E+18	FALSO	High	IsMasterPr	AAEL0250	2:4712388	0	14	FALSO	4
-4,62E+18	FALSO	High	IsMasterPr	AAEL0083	3:4137969	0	24	FALSO	5
-3,61E+18	FALSO	High	IsMasterPr	AAEL00667	2:3263181	0	9	FALSO	4
7,34E+18	FALSO	High	IsMasterPr	AAEL00395	3:2677145	0	13	FALSO	4
-2,87E+18	FALSO	High	IsMasterPr	AAEL00772	3:2553340	0	28	FALSO	4
-4,32E+18	FALSO	High	IsMasterPr	AAEL01271	1:9304065	0	9	FALSO	2
2,61E+17	FALSO	High	IsMasterPr	AAEL0070	3:2996781	0	17	FALSO	5
2,24E+18	FALSO	High	IsMasterPr	AAEL00118	2:3076468	0	9	FALSO	2
3,68E+18	FALSO	High	IsMasterPr	AAEL00973	2:6858894	0	7	FALSO	4
1,51E+18	FALSO	High	IsMasterPr	AAEL0059	3:1546684	0	17	FALSO	4
-7,66E+18	FALSO	High	IsMasterPr	AAEL01187	2:1873031	0	19	FALSO	3
7,72E+18	FALSO	High	IsMasterPr	AAEL00303	1:1306580	0	10	FALSO	4
6,79E+18	FALSO	High	IsMasterPr	AAEL00037	2:1244358	0	13	FALSO	5

8,36E+18	FALSO	High	IsMasterPr	AAEL01743	1:1508395	0	13	FALSO	5
-2,94E+18	FALSO	High	IsMasterPr	AAEL00651	2:4686771	0	43	FALSO	4
4,48E+18	FALSO	High	IsMasterPr	AAEL00041	3:1853511	0	6	FALSO	3
3,27E+18	FALSO	High	IsMasterPr	AAEL01954	3:3179510	0	14	FALSO	4
7,07E+18	FALSO	High	IsMasterPr	AAEL01493	2:1451270	0	7	FALSO	5
9,19E+18	FALSO	High	IsMasterPr	AAEL00806	2:2311016	0	5	FALSO	4
-2,47E+18	FALSO	High	IsMasterPr	AAEL00686	1:1939539	0	48	FALSO	5
9,39E+17	FALSO	High	IsMasterPr	AAEL00632	3:3273097	0	4	FALSO	4
-3,54E+18	FALSO	High	IsMasterPr	AAEL01741	3:8478896	0	17	FALSO	3
7,75E+18	FALSO	High	IsMasterPr	AAEL00021	3:1096017	0	7	FALSO	4
8,57E+18	FALSO	High	IsMasterPr	AAEL00549	3:5988676	0	19	FALSO	3
-4,91E+18	FALSO	High	IsMasterPr	AAEL00881	3:1333638	0	31	FALSO	3
-6,20E+18	FALSO	High	IsMasterPr	AAEL01504	2:1049533	0	8	FALSO	2
4,31E+18	FALSO	High	IsMasterPr	AAEL00812	3:1073724	0	39	FALSO	2
5,55E+18	FALSO	High	IsMasterPr	AAEL00465	3:2466094	0	29	FALSO	2
-4,69E+18	FALSO	High	IsMasterPr	AAEL01288	3:4218252	0	10	FALSO	3
-1,00E+18	FALSO	High	IsMasterPr	AAEL00058	3:1202484	0	6	FALSO	5
-1,46E+18	FALSO	High	IsMasterPr	AAEL01352	1:1440295	0	15	FALSO	4
6,62E+18	FALSO	High	IsMasterPr	AAEL01756	1:9892065	0	28	FALSO	4
5,94E+17	FALSO	High	IsMasterPr	AAEL01448	2:3492465	0	9	FALSO	3
5,31E+18	FALSO	High	IsMasterPr	AAEL01026	2:3600852	0	13	FALSO	3
-3,73E+18	FALSO	High	IsMasterPr	AAEL01283	2:1054406	0	12	FALSO	6
3,54E+18	FALSO	High	IsMasterPr	AAEL01399	2:1019219	0	12	FALSO	2
-8,62E+18	FALSO	High	IsMasterPr	AAEL00737	2:2344670	0	24	FALSO	2
6,96E+18	FALSO	High	IsMasterPr	AAEL00738	2:2332654	0	11	FALSO	2
-7,53E+18	FALSO	High	IsMasterPr	AAEL00944	1:1631055	0	22	FALSO	3
-7,40E+18	FALSO	High	IsMasterPr	AAEL00058	2:2834205	0	6	FALSO	3
1,62E+18	FALSO	High	IsMasterPr	AAEL01286	1:2221310	0	24	FALSO	3
-7,49E+18	FALSO	High	IsMasterPr	AAEL02320	2:2278245	0	12	FALSO	2
-7,02E+18	FALSO	High	IsMasterPr	AAEL00739	3:4083092	0	24	FALSO	5
1,22E+18	FALSO	High	IsMasterPr	AAEL00584	3:2200989	0	6	FALSO	6
-6,26E+18	FALSO	High	IsMasterPr	AAEL02335	3:3654806	0	25	FALSO	2
-1,46E+18	FALSO	High	IsMasterPr	AAEL02061	1:6638823	0	9	FALSO	6
7,11E+18	FALSO	High	IsMasterPr	AAEL00450	NIGP0100C	0	46	FALSO	3
1,57E+18	FALSO	High	IsMasterPr	AAEL01441	2:3661047	0	41	FALSO	2
-6,57E+18	FALSO	High	IsMasterPr	AAEL00140	2:4829065	0	7	FALSO	3
2,06E+18	FALSO	High	IsMasterPr	AAEL02389	1:3065201	0	2	FALSO	4
-3,00E+18	FALSO	High	IsMasterPr	AAEL01059	1:3013179	0	23	FALSO	4
-4,00E+18	FALSO	High	IsMasterPr	AAEL00206	2:3566375	0	7	FALSO	2
-7,65E+18	FALSO	High	IsMasterPr	AAEL00476	2:2885017	0	43	FALSO	6
8,90E+18	FALSO	High	IsMasterPr	AAEL00765	1:1336793	0	7	FALSO	4
-4,89E+18	FALSO	High	IsMasterPr	AAEL00349	2:2163886	0	9	FALSO	5
2,13E+18	FALSO	High	IsMasterPr	AAEL00929	2:1743559	0	11	FALSO	4
-2,66E+18	FALSO	High	IsMasterPr	AAEL01702	2:6799199	0	20	FALSO	4
-6,50E+18	FALSO	High	IsMasterPr	AAEL02414	2:4646654	0	4	FALSO	4
8,73E+17	FALSO	High	IsMasterPr	AAEL02356	1:2651363	0	5	FALSO	4
-5,34E+18	FALSO	High	IsMasterPr	AAEL00581	1:2002319	0	22	FALSO	3
-7,28E+18	FALSO	High	IsMasterPr	AAEL00723	2:4223877	0	1	FALSO	1
-5,08E+18	FALSO	High	IsMasterPr	AAEL01119	2:3797038	0	29	FALSO	4
2,21E+18	FALSO	High	IsMasterPr	AAEL02411	3:2207786	0	5	FALSO	2

-6,15E+18	FALSO	High	IsMasterPr	AAEL0004€	3:1858581ε	0	17	FALSO	4
-5,30E+17	FALSO	High	IsMasterPr	AAEL00343	3:3501522ε	0	10	FALSO	2
4,65E+18	FALSO	High	IsMasterPr	AAEL01425	3:2951101	0	14	FALSO	4
5,75E+18	FALSO	High	IsMasterPr	AAEL0058€	3:2188419	0	2	FALSO	3
-5,83E+18	FALSO	High	IsMasterPr	AAEL00968	3:2096568	0	23	FALSO	3
4,80E+18	FALSO	High	IsMasterPr	AAEL00963	3:3691593	0	3	FALSO	3
2,38E+18	FALSO	High	IsMasterPr	AAEL01973	2:8332201	0	4	FALSO	4
2,55E+18	FALSO	High	IsMasterPr	AAEL00517	2:406344:4	0	6	FALSO	3
-1,26E+18	FALSO	High	IsMasterPr	AAEL01823	2:4057241	0	6	FALSO	4
5,07E+17	FALSO	High	IsMasterPr	AAEL00942	2:2465985	0	8	FALSO	5
9,22E+18	FALSO	High	IsMasterPr	AAEL01403	2:4461552	0	9	FALSO	2
-2,71E+18	FALSO	High	IsMasterPr	AAEL01835	3:2186446	0	6	FALSO	4
1,72E+18	FALSO	High	IsMasterPr	AAEL01025	3:2790462	0	15	FALSO	4
-7,10E+18	FALSO	High	IsMasterPr	AAEL01812	2:5574013	0	16	FALSO	5
-2,63E+18	FALSO	High	IsMasterPr	AAEL00924	2:1709347	0	8	FALSO	3
-8,22E+18	FALSO	High	IsMasterPr	AAEL0057S	1:2011484	0	7	FALSO	3
-9,19E+16	FALSO	High	IsMasterPr	AAEL00405	1:1357091	0	35	FALSO	3
2,21E+18	FALSO	High	IsMasterPr	AAEL00888	2:1262644	0	51	FALSO	4
6,40E+18	FALSO	High	IsMasterPr	AAEL0217S	2:6482231	0	8	FALSO	4
-8,68E+18	FALSO	High	IsMasterPr	AAEL0226C	3:9296451	0	6	FALSO	4
7,27E+18	FALSO	High	IsMasterPr	AAEL00521	3:8886498	0	12	FALSO	5
-4,32E+18	FALSO	High	IsMasterPr	AAEL01067	2:1360153	0	25	FALSO	5
3,59E+18	FALSO	High	IsMasterPr	AAEL00258	3:2571506	0	34	FALSO	3
9,00E+18	FALSO	High	IsMasterPr	AAEL0010S	1:2996259	0	17	FALSO	4
5,21E+18	FALSO	High	IsMasterPr	AAEL0033S	3:8136133	0	36	FALSO	3
2,03E+18	FALSO	High	IsMasterPr	Q5MX76	Outer surfa	0	36	FALSO	5
2,22E+18	FALSO	High	IsMasterPr	AAEL02393	NIGP01001	0	10	FALSO	3
1,30E+18	FALSO	High	IsMasterPr	AAEL00848	2:1904524	0	9	FALSO	2
1,83E+18	FALSO	High	IsMasterPr	AAEL02454	2:1686586	0	12	FALSO	3
7,26E+18	FALSO	High	IsMasterPr	AAEL0039€	3:2670567	0	4	FALSO	3
1,58E+18	FALSO	High	IsMasterPr	AAEL0025S	3:2567470	0	13	FALSO	5
-6,50E+18	FALSO	High	IsMasterPr	AAEL01441	1:2867937	0	19	FALSO	4
1,82E+17	FALSO	High	IsMasterPr	AAEL00807	2:2307415	0	17	FALSO	1
-4,89E+18	FALSO	High	IsMasterPr	AAEL02371	3:2800734	0	5	FALSO	4
8,97E+18	FALSO	High	IsMasterPr	AAEL00917	3:1760806	0	8	FALSO	5
-3,89E+18	FALSO	High	IsMasterPr	AAEL00254	1:2289992	0	10	FALSO	4
1,27E+18	FALSO	High	IsMasterPr	AAEL0023C	3:3756357	0	14	FALSO	2
2,89E+18	FALSO	High	IsMasterPr	AAEL00393	2:3548041	0	8	FALSO	1
-2,02E+18	FALSO	High	IsMasterPr	AAEL0042S	2:9954230	0	20	FALSO	3
-5,06E+18	FALSO	High	IsMasterPr	AAEL00181	2:3745025	0	20	FALSO	5
7,85E+18	FALSO	High	IsMasterPr	AAEL01834	2:2586813	0	9	FALSO	4
-7,54E+18	FALSO	High	IsMasterPr	AAEL00585	3:2202520	0	13	FALSO	3
-4,30E+18	FALSO	High	IsMasterPr	AAEL00093	3:3801158	0	7	FALSO	1
-4,50E+18	FALSO	High	IsMasterPr	AAEL02362	1:2103450	0	6	FALSO	3
8,06E+18	FALSO	High	IsMasterPr	AAEL0070S	2:1294837	0	22	FALSO	3
-4,76E+18	FALSO	High	IsMasterPr	AAEL01754	3:6649165	0	48	FALSO	2
7,25E+18	FALSO	High	IsMasterPr	AAEL0095C	3:3449521	0	23	FALSO	4
-1,87E+18	FALSO	High	IsMasterPr	AAEL00262	3:2572629	0	12	FALSO	3
-5,29E+18	FALSO	High	IsMasterPr	AAEL00468	3:2486598	0	9	FALSO	3
-4,99E+18	FALSO	High	IsMasterPr	AAEL00818	3:5361534	0	15	FALSO	5

-1,75E+18	FALSO	High	IsMasterPr	AAEL0115C	2:8118887	0	6	FALSO	6
-1,67E+18	FALSO	High	IsMasterPr	AAEL00147	2:2631840	0	13	FALSO	4
1,41E+18	FALSO	High	IsMasterPr	AAEL00029	3:2665310	0	11	FALSO	1
8,00E+16	FALSO	High	IsMasterPr	AAEL00227	2:3085980	0	8	FALSO	2
3,93E+18	FALSO	High	IsMasterPr	AAEL00903	2:1017312	0	10	FALSO	2
8,62E+18	FALSO	High	IsMasterPr	AAEL00381	1:4492396	0	12	FALSO	5
-1,51E+18	FALSO	High	IsMasterPr	AAEL02363	2:3409639	0	8	FALSO	4
8,82E+17	FALSO	High	IsMasterPr	AAEL01361	3:1951959	0	3	FALSO	2
7,15E+18	FALSO	High	IsMasterPr	AAEL02299	2:1917553	0	11	FALSO	5
1,10E+18	FALSO	High	IsMasterPr	AAEL02648	1:2880808	0	29	FALSO	2
-9,18E+18	FALSO	High	IsMasterPr	AAEL01117	2:1823683	0	6	FALSO	2
-8,79E+17	FALSO	High	IsMasterPr	AAEL02524	3:2953726	0	20	FALSO	3
3,41E+18	FALSO	High	IsMasterPr	AAEL00679	3:3683370	0	5	FALSO	6
1,53E+18	FALSO	High	IsMasterPr	AAEL00217	1:2517911	0	16	FALSO	3
3,74E+18	FALSO	High	IsMasterPr	AAEL00545	2:2969199	0	5	FALSO	3
-4,67E+18	FALSO	High	IsMasterPr	AAEL01221	3:1702680	0	37	FALSO	2
-8,81E+18	FALSO	High	IsMasterPr	AAEL00358	1:6937231	0	30	FALSO	4
6,72E+18	FALSO	High	IsMasterPr	AAEL01353	2:2460956	0	7	FALSO	5
3,77E+18	FALSO	High	IsMasterPr	AAEL02243	2:4212641	0	7	FALSO	5
-3,26E+18	FALSO	High	IsMasterPr	AAEL00965	1:9287422	0	9	FALSO	2
-3,74E+18	FALSO	High	IsMasterPr	AAEL00435	3:9369339	0	24	FALSO	4
1,76E+18	FALSO	High	IsMasterPr	AAEL02468	1:1898180	0	15	FALSO	2
3,80E+18	FALSO	High	IsMasterPr	AAEL0011C	3:4068164	0	18	FALSO	4
-3,57E+18	FALSO	High	IsMasterPr	AAEL02275	2:1793692	0	9	FALSO	5
5,07E+18	FALSO	High	IsMasterPr	AAEL01519	2:1231651	0	14	FALSO	5
-3,55E+18	FALSO	High	IsMasterPr	A0A1P8W2	Outer surfa	0	20	FALSO	3
5,80E+18	FALSO	High	IsMasterPr	AAEL0153C	2:1743363	0	12	FALSO	3
-3,46E+18	FALSO	High	IsMasterPr	AAEL01491	1:3036954	0	13	FALSO	5
8,64E+18	FALSO	High	IsMasterPr	AAEL02195	1:2151685	0	8	FALSO	5
-8,26E+18	FALSO	High	IsMasterPr	AAEL0197C	1:2202703	0	5	FALSO	3
4,62E+18	FALSO	High	IsMasterPr	AAEL02068	2:1348307	0	3	FALSO	3
-3,85E+18	FALSO	High	IsMasterPr	AAEL00802	1:7425611	0	4	FALSO	3
8,08E+18	FALSO	High	IsMasterPr	AAEL01144	2:3743146	0	43	FALSO	2
8,59E+18	FALSO	High	IsMasterPr	AAEL02025	3:2888422	0	10	FALSO	4
-5,33E+18	FALSO	High	IsMasterPr	AAEL0027C	3:1245065	0	15	FALSO	4
-2,25E+17	FALSO	High	IsMasterPr	AAEL00837	3:2245378	0	24	FALSO	4
-4,19E+18	FALSO	High	IsMasterPr	AAEL00447	3:1101138	0	8	FALSO	3
5,29E+18	FALSO	High	IsMasterPr	AAEL00842	1:2053879	0	11	FALSO	2
8,59E+18	FALSO	High	IsMasterPr	AAEL01404	1:2204065	0	13	FALSO	4
3,54E+18	FALSO	High	IsMasterPr	AAEL0134C	1:1549066	0	9	FALSO	3
-9,21E+18	FALSO	High	IsMasterPr	AAEL00641	2:4138224	0	8	FALSO	4
-1,93E+18	FALSO	High	IsMasterPr	AAEL00089	3:3324257	0	29	FALSO	4
-6,22E+18	FALSO	High	IsMasterPr	AAEL00779	3:1517207	0	7	FALSO	3
-4,88E+18	FALSO	High	IsMasterPr	AAEL02802	1:2124619	0	9	FALSO	4
-2,16E+18	FALSO	High	IsMasterPr	AAEL01222	2:3350168	0	34	FALSO	4
-3,31E+18	FALSO	High	IsMasterPr	AAEL01306	2:3358748	0	31	FALSO	3
3,36E+18	FALSO	High	IsMasterPr	AAEL00034	2:1242334	0	29	FALSO	3
4,45E+18	FALSO	High	IsMasterPr	AAEL00162	1:1382962	0	11	FALSO	4
8,69E+18	FALSO	High	IsMasterPr	AAEL00219	2:4290751	0	30	FALSO	2
7,39E+18	FALSO	High	IsMasterPr	AAEL01972	2:3158800	0	6	FALSO	4

-2,04E+17	FALSO	High	IsMasterPr	AAEL0001	3:2146129	0	2	FALSO	4
5,88E+17	FALSO	High	IsMasterPr	AAEL01071	1:2774834	0	2	FALSO	3
-3,40E+17	FALSO	High	IsMasterPr	AAEL01307	2:2533642	0	8	FALSO	2
2,22E+18	FALSO	High	IsMasterPr	AAEL0280	1:2614669	0	16	FALSO	3
-7,38E+18	FALSO	High	IsMasterPr	AAEL0025	3:2568599	0	15	FALSO	3
3,87E+18	FALSO	High	IsMasterPr	AAEL00334	2:4007673	0	15	FALSO	4
-3,08E+18	FALSO	High	IsMasterPr	AAEL0115	3:3152465	0	37	FALSO	4
-4,77E+18	FALSO	High	IsMasterPr	AAEL0007	3:2462212	0	14	FALSO	4
-4,64E+18	FALSO	High	IsMasterPr	AAEL0195	2:3073901	0	5	FALSO	4
-9,13E+18	FALSO	High	IsMasterPr	AAEL0043	3:9438980	0	10	FALSO	2
-4,19E+18	FALSO	High	IsMasterPr	AAEL0013	2:2062895	0	14	FALSO	3
3,15E+18	FALSO	High	IsMasterPr	AAEL0107	1:1786671	0	13	FALSO	5
-8,26E+18	FALSO	High	IsMasterPr	AAEL0036	3:6531189	0	16	FALSO	2
6,50E+18	FALSO	High	IsMasterPr	AAEL0119	3:4526017	0	18	FALSO	3
-5,68E+16	FALSO	High	IsMasterPr	AAEL00304	1:1291102	0	3	FALSO	2
-3,17E+18	FALSO	High	IsMasterPr	AAEL00931	NIGP01001	0	4	FALSO	1
-4,54E+18	FALSO	High	IsMasterPr	AAEL0057	3:1154662	0	9	FALSO	3
-7,26E+18	FALSO	High	IsMasterPr	AAEL0027	3:1257651	0	22	FALSO	3
5,75E+18	FALSO	High	IsMasterPr	AAEL02204	3:9460966	0	2	FALSO	3
8,34E+18	FALSO	High	IsMasterPr	AAEL0124	1:7250179	0	5	FALSO	3
-4,58E+18	FALSO	High	IsMasterPr	AAEL00224	2:4291034	0	34	FALSO	3
1,82E+18	FALSO	High	IsMasterPr	AAEL00727	2:3124122	0	11	FALSO	4
3,47E+18	FALSO	High	IsMasterPr	AAEL00633	3:3978385	0	10	FALSO	3
-8,40E+18	FALSO	High	IsMasterPr	AAEL00667	2:3250420	0	12	FALSO	3
2,57E+18	FALSO	High	IsMasterPr	AAEL0028	1:2561138	0	4	FALSO	6
-5,61E+18	FALSO	High	IsMasterPr	AAEL00607	1:1827473	0	4	FALSO	4
-4,79E+18	FALSO	High	IsMasterPr	AAEL0267	2:2616581	0	11	FALSO	5
-2,48E+18	FALSO	High	IsMasterPr	AAEL0199	3:1682568	0	4	FALSO	3
-4,63E+18	FALSO	High	IsMasterPr	AAEL02682	1:2788017	0	3	FALSO	1
8,09E+18	FALSO	High	IsMasterPr	AAEL0083	1:9700937	0	12	FALSO	2
-6,79E+18	FALSO	High	IsMasterPr	AAEL01131	2:3314910	0	21	FALSO	5
-4,69E+18	FALSO	High	IsMasterPr	AAEL0056	2:3817778	0	10	FALSO	4
-8,56E+18	FALSO	High	IsMasterPr	AAEL0100	3:1120281	0	13	FALSO	3
6,65E+18	FALSO	High	IsMasterPr	AAEL00517	2:226546:2	0	34	FALSO	4
7,31E+18	FALSO	High	IsMasterPr	AAEL0169	1:1079429	0	8	FALSO	4
4,43E+18	FALSO	High	IsMasterPr	AAEL0105	2:9105039	0	33	FALSO	3
-1,53E+18	FALSO	High	IsMasterPr	AAEL0255	2:3183343	0	10	FALSO	3
-1,11E+18	FALSO	High	IsMasterPr	AAEL0058	1:2012318	0	6	FALSO	5
-5,64E+18	FALSO	High	IsMasterPr	AAEL02121	3:2812527	0	10	FALSO	3
-8,63E+18	FALSO	High	IsMasterPr	AAEL0098	2:1646297	0	16	FALSO	1
-7,14E+18	FALSO	High	IsMasterPr	AAEL0068	2:2511345	0	14	FALSO	4
-8,05E+18	FALSO	High	IsMasterPr	AAEL02744	2:1258752	0	3	FALSO	4
7,04E+18	FALSO	High	IsMasterPr	AAEL0042	2:2931609	0	13	FALSO	3
2,75E+18	FALSO	High	IsMasterPr	AAEL0063	3:2310909	0	17	FALSO	3
3,02E+17	FALSO	High	IsMasterPr	AAEL0118	2:3961052	0	30	FALSO	2
-2,39E+18	FALSO	High	IsMasterPr	AAEL00642	2:4146832	0	18	FALSO	3
2,73E+18	FALSO	High	IsMasterPr	AAEL0042	3:2926632	0	14	FALSO	5
-6,62E+18	FALSO	High	IsMasterPr	AAEL0017	2:1657934	0	15	FALSO	4
2,49E+18	FALSO	High	IsMasterPr	AAEL0139	3:2304349	0	17	FALSO	3
-2,71E+18	FALSO	High	IsMasterPr	AAEL02087	2:1284070	0	6	FALSO	4

6,03E+18	FALSO	High	IsMasterPr	AAEL00261	3:2571427	0	20	FALSO	2
4,33E+18	FALSO	High	IsMasterPr	AAEL01832	2:2887474	0	5	FALSO	2
4,33E+18	FALSO	High	IsMasterPr	AAEL00967	2:2037995	0	4	FALSO	3
3,94E+18	FALSO	High	IsMasterPr	AAEL00855	2:1574036	0	9	FALSO	4
-9,48E+17	FALSO	High	IsMasterPr	AAEL00499	2:2822545	0	18	FALSO	3
-1,76E+18	FALSO	High	IsMasterPr	AAEL01821	3:4879735	0	4	FALSO	3
1,17E+18	FALSO	High	IsMasterPr	AAEL00507	1:2953670	0	17	FALSO	4
-3,01E+17	FALSO	High	IsMasterPr	AAEL00902	3:1931117	0	11	FALSO	2
-1,05E+18	FALSO	High	IsMasterPr	AAEL00545	2:2957399	0	5	FALSO	3
-2,85E+18	FALSO	High	IsMasterPr	AAEL00572	1:1237560	0	5	FALSO	2
-6,63E+18	FALSO	High	IsMasterPr	AAEL00771	3:2555613	0	28	FALSO	4
8,36E+18	FALSO	High	IsMasterPr	AAEL0095C	3:3447899	0	17	FALSO	6
5,63E+18	FALSO	High	IsMasterPr	AAEL0099C	1:2072134	0	15	FALSO	4
3,45E+18	FALSO	High	IsMasterPr	AAEL01823	1:1113777	0	5	FALSO	3
-4,44E+18	FALSO	High	IsMasterPr	AAEL00107	1:2996093	0	11	FALSO	4
6,32E+17	FALSO	High	IsMasterPr	AAEL01047	1:2786412	0	17	FALSO	4
-2,73E+18	FALSO	High	IsMasterPr	AAEL00868	2:3211447	0	9	FALSO	3
-5,14E+18	FALSO	High	IsMasterPr	A0A2A4IJU	Elongation	0	12	FALSO	4
-3,89E+18	FALSO	High	IsMasterPr	AAEL01132	3:3221601	0	43	FALSO	3
4,86E+18	FALSO	High	IsMasterPr	AAEL01323	3:2799441	0	5	FALSO	3
-3,39E+18	FALSO	High	IsMasterPr	AAEL00656	2:1331185	0	8	FALSO	2
4,21E+18	FALSO	High	IsMasterPr	AAEL01024	2:4028538	0	49	FALSO	5
-5,10E+18	FALSO	High	IsMasterPr	AAEL01736	2:3003454	0	3	FALSO	4
7,92E+18	FALSO	High	IsMasterPr	AAEL00094	2:3018172	0	5	FALSO	2
-9,03E+18	FALSO	High	IsMasterPr	AAEL00389	3:8495187	0	6	FALSO	3
1,19E+18	FALSO	High	IsMasterPr	AAEL00859	2:3624269	0	14	FALSO	4
-5,94E+17	FALSO	High	IsMasterPr	AAEL01322	3:3410311	0	20	FALSO	3
-9,11E+18	FALSO	High	IsMasterPr	AAEL00056	3:3880458	0	45	FALSO	3
-3,43E+18	FALSO	High	IsMasterPr	AAEL00144	1:1114609	0	6	FALSO	2
-4,93E+18	FALSO	High	IsMasterPr	AAEL0068C	3:3682731	0	11	FALSO	3
-7,03E+18	FALSO	High	IsMasterPr	AAEL0227C	NIGP01001	0	32	FALSO	2
-1,19E+18	FALSO	High	IsMasterPr	AAEL00596	2:3609196	0	20	FALSO	3
-9,53E+16	FALSO	High	IsMasterPr	AAEL00636	1:1014496	0	31	FALSO	4
3,58E+18	FALSO	High	IsMasterPr	AAEL00618	3:1417642	0	3	FALSO	5
6,87E+18	FALSO	High	IsMasterPr	AAEL0039C	1:2506182	0	7	FALSO	3
4,63E+17	FALSO	High	IsMasterPr	AAEL00017	3:2124428	0	14	FALSO	3
-8,04E+18	FALSO	High	IsMasterPr	AAEL00896	2:1784918	0	25	FALSO	2
-3,95E+18	FALSO	High	IsMasterPr	AAEL01075	3:2940886	0	22	FALSO	3
7,05E+18	FALSO	High	IsMasterPr	AAEL0077C	2:4708250	0	10	FALSO	3
-4,80E+18	FALSO	High	IsMasterPr	AAEL00343	3:3492998	0	12	FALSO	4
-7,37E+18	FALSO	High	IsMasterPr	AAEL01945	2:1486292	0	13	FALSO	2
-6,40E+18	FALSO	High	IsMasterPr	AAEL00183	2:2742950	0	37	FALSO	5
-3,66E+18	FALSO	High	IsMasterPr	AAEL01165	3:2148205	0	9	FALSO	4
-6,72E+17	FALSO	High	IsMasterPr	AAEL00949	3:3449532	0	26	FALSO	5
-3,96E+18	FALSO	High	IsMasterPr	AAEL00942	2:2470825	0	21	FALSO	4
3,43E+18	FALSO	High	IsMasterPr	AAEL02686	1:2100558	0	15	FALSO	2
5,58E+18	FALSO	High	IsMasterPr	AAEL01407	2:4060053	0	3	FALSO	2
4,56E+18	FALSO	High	IsMasterPr	AAEL01002	2:1103820	0	17	FALSO	4
3,31E+18	FALSO	High	IsMasterPr	AAEL02529	1:2918465	0	3	FALSO	3
-3,53E+18	FALSO	High	IsMasterPr	AAEL00414	3:1536378	0	11	FALSO	3

-4,00E+17	FALSO	High	IsMasterPr	AAEL00893	1:80929419	0	6	FALSO	2
1,67E+18	FALSO	High	IsMasterPr	AAEL00573	2:2940662	0	10	FALSO	3
2,97E+18	FALSO	High	IsMasterPr	AAEL0053C	3:3084185	0	25	FALSO	4
7,48E+18	FALSO	High	IsMasterPr	AAEL0138C	1:6138879	0	13	FALSO	2
-2,47E+16	FALSO	High	IsMasterPr	AAEL00847	2:1933770	0	23	FALSO	3
2,78E+18	FALSO	High	IsMasterPr	AAEL00741	2:1346223	0	32	FALSO	3
-8,37E+18	FALSO	High	IsMasterPr	AAEL01226	2:3844316	0	12	FALSO	3
5,67E+18	FALSO	High	IsMasterPr	AAEL00402	3:1662270	0	9	FALSO	3
2,89E+18	FALSO	High	IsMasterPr	AAEL00027	3:2649198	0	4	FALSO	4
3,52E+18	FALSO	High	IsMasterPr	AAEL0095C	NIGP01002	0	7	FALSO	2
-3,39E+18	FALSO	High	IsMasterPr	AAEL00769	2:4707653	0	9	FALSO	4
8,24E+18	FALSO	High	IsMasterPr	AAEL01311	1:1428979	0	28	FALSO	3
7,16E+18	FALSO	High	IsMasterPr	AAEL00473	2:2887015	0	7	FALSO	2
6,21E+18	FALSO	High	IsMasterPr	AAEL00554	3:9059453	0	6	FALSO	3
-3,69E+18	FALSO	High	IsMasterPr	AAEL02453	2:1626019	0	13	FALSO	2
4,30E+18	FALSO	High	IsMasterPr	AAEL0290C	2:4291022	0	34	FALSO	3
6,19E+18	FALSO	High	IsMasterPr	AAEL00763	2:1072082	0	5	FALSO	2
6,42E+18	FALSO	High	IsMasterPr	AAEL00946	1:5733659	0	29	FALSO	3
9,25E+16	FALSO	High	IsMasterPr	AAEL02433	2:1500942	0	10	FALSO	2
3,04E+18	FALSO	High	IsMasterPr	AAEL00408	3:4033527	0	6	FALSO	3
8,95E+18	FALSO	High	IsMasterPr	AAEL01951	3:1878274	0	5	FALSO	3
-1,88E+16	FALSO	High	IsMasterPr	AAEL01292	2:3865188	0	7	FALSO	3
6,78E+18	FALSO	High	IsMasterPr	AAEL01003	2:1107649	0	11	FALSO	2
8,39E+18	FALSO	High	IsMasterPr	AAEL01123	3:2394857	0	47	FALSO	3
1,78E+18	FALSO	High	IsMasterPr	AAEL00687	3:3698961	0	10	FALSO	3
5,91E+18	FALSO	High	IsMasterPr	AAEL0031C	2:4315393	0	5	FALSO	2
2,74E+18	FALSO	High	IsMasterPr	AAEL00848	2:1921843	0	19	FALSO	2
9,06E+17	FALSO	High	IsMasterPr	AAEL00612	3:6937154	0	5	FALSO	1
1,79E+18	FALSO	High	IsMasterPr	AAEL01135	1:1647258	0	8	FALSO	4
-7,83E+18	FALSO	High	IsMasterPr	AAEL01452	1:2243403	0	8	FALSO	3
2,08E+18	FALSO	High	IsMasterPr	AAEL00441	3:2051944	0	8	FALSO	4
7,41E+18	FALSO	High	IsMasterPr	AAEL01241	3:2902907	0	5	FALSO	4
4,79E+18	FALSO	High	IsMasterPr	AAEL00467	3:2484642	0	20	FALSO	4
-6,81E+18	FALSO	High	IsMasterPr	AAEL01007	3:2092973	0	10	FALSO	2
-6,79E+18	FALSO	High	IsMasterPr	AAEL01487	3:2939460	0	12	FALSO	4
-2,28E+18	FALSO	High	IsMasterPr	AAEL00228	3:3750986	0	21	FALSO	4
2,02E+18	FALSO	High	IsMasterPr	AAEL01701	2:3059521	0	7	FALSO	4
6,27E+18	FALSO	High	IsMasterPr	AAEL02286	2:1595988	0	10	FALSO	3
7,45E+17	FALSO	High	IsMasterPr	AAEL00618	3:1421515	0	5	FALSO	4
-2,42E+18	FALSO	High	IsMasterPr	AAEL02555	1:3824475	0	15	FALSO	1
5,06E+18	FALSO	High	IsMasterPr	AAEL00541	1:2472985	0	22	FALSO	4
-8,54E+18	FALSO	High	IsMasterPr	AAEL0007C	2:4587788	0	10	FALSO	2
4,88E+18	FALSO	High	IsMasterPr	AAEL02409	3:2373187	0	6	FALSO	2
-5,82E+18	FALSO	High	IsMasterPr	AAEL01425	3:2953457	0	6	FALSO	2
3,18E+18	FALSO	High	IsMasterPr	AAEL01947	1:1463574	0	5	FALSO	5
-2,51E+18	FALSO	High	IsMasterPr	AAEL00446	3:2123139	0	7	FALSO	2
-2,80E+18	FALSO	High	IsMasterPr	AAEL00325	2:3666107	0	41	FALSO	2
8,50E+18	FALSO	High	IsMasterPr	AAEL00521	2:27322:58	0	5	FALSO	3
3,58E+18	FALSO	High	IsMasterPr	AAEL00539	2:2575077	0	24	FALSO	5
-9,21E+18	FALSO	High	IsMasterPr	A0A1V2N4	Chaperone	0	5	FALSO	2

3,86E+17	FALSO	High	IsMasterPr	AAEL00232	2:1752834	0	18	FALSO	3
-4,27E+18	FALSO	High	IsMasterPr	AAEL00341	3:3492638	0	12	FALSO	1
4,71E+18	FALSO	High	IsMasterPr	AAEL00444	3:2065470	0	6	FALSO	1
7,77E+18	FALSO	High	IsMasterPr	AAEL01268	3:2393713	0	27	FALSO	3
-3,24E+18	FALSO	High	IsMasterPr	AAEL01999	2:2883033	0	15	FALSO	2
3,45E+18	FALSO	High	IsMasterPr	AAEL00194	2:1936292	0	10	FALSO	3
8,70E+18	FALSO	High	IsMasterPr	AAEL00815	3:1002847	0	17	FALSO	3
9,00E+17	FALSO	High	IsMasterPr	AAEL01262	1:2793376	0	6	FALSO	2
7,57E+18	FALSO	High	IsMasterPr	AAEL00841	NIGP01001	0	30	FALSO	4
4,86E+17	FALSO	High	IsMasterPr	AAEL00031	2:3679107	0	5	FALSO	2
7,32E+18	FALSO	High	IsMasterPr	AAEL01118	3:5132752	0	3	FALSO	3
8,98E+18	FALSO	High	IsMasterPr	AAEL00646	3:2163308	0	19	FALSO	1
6,53E+18	FALSO	High	IsMasterPr	AAEL02707	2:8637251	0	34	FALSO	1
6,26E+18	FALSO	High	IsMasterPr	AAEL00480	3:2734280	0	6	FALSO	3
-3,17E+18	FALSO	High	IsMasterPr	AAEL01802	2:2851439	0	8	FALSO	2
-5,87E+18	FALSO	High	IsMasterPr	AAEL01063	2:2791553	0	15	FALSO	2
3,20E+18	FALSO	High	IsMasterPr	AAEL01327	2:3498312	0	4	FALSO	1
-7,37E+18	FALSO	High	IsMasterPr	AAEL00520	2:638065:6	0	11	FALSO	3
2,65E+18	FALSO	High	IsMasterPr	AAEL01247	2:2186098	0	24	FALSO	5
4,05E+18	FALSO	High	IsMasterPr	AAEL00314	3:2696047	0	10	FALSO	3
-7,38E+18	FALSO	High	IsMasterPr	AAEL02002	3:3174960	0	17	FALSO	2
5,18E+18	FALSO	High	IsMasterPr	AAEL00412	3:1539580	0	6	FALSO	2
-8,44E+18	FALSO	High	IsMasterPr	AAEL00857	3:1980281	0	3	FALSO	1
-4,69E+18	FALSO	High	IsMasterPr	AAEL01191	3:3376951	0	6	FALSO	2
-5,19E+18	FALSO	High	IsMasterPr	AAEL01223	1:1468329	0	10	FALSO	4
5,56E+18	FALSO	High	IsMasterPr	AAEL02026	2:1223307	0	9	FALSO	3
-8,25E+17	FALSO	High	IsMasterPr	AAEL01158	1:1203305	0	10	FALSO	1
8,44E+18	FALSO	High	IsMasterPr	AAEL00263	2:2455083	0	7	FALSO	3
-3,71E+18	FALSO	High	IsMasterPr	AAEL00696	2:3987476	0	17	FALSO	4
4,35E+18	FALSO	High	IsMasterPr	AAEL00693	1:1567085	0	4	FALSO	4
4,97E+18	FALSO	High	IsMasterPr	AAEL00180	2:3752563	0	20	FALSO	3
4,02E+18	FALSO	High	IsMasterPr	AAEL00795	2:3516093	0	8	FALSO	1
-8,03E+18	FALSO	High	IsMasterPr	AAEL00240	1:1773063	0	3	FALSO	2
-2,31E+18	FALSO	High	IsMasterPr	AAEL00652	2:4687153	0	9	FALSO	2
8,43E+17	FALSO	High	IsMasterPr	AAEL01037	2:1829098	0	21	FALSO	3
1,03E+17	FALSO	High	IsMasterPr	AAEL00334	2:4614333	0	11	FALSO	2
6,55E+17	FALSO	High	IsMasterPr	AAEL00964	1:2227408	0	16	FALSO	3
7,10E+18	FALSO	High	IsMasterPr	AAEL00324	2:3667056	0	11	FALSO	3
-4,57E+18	FALSO	High	IsMasterPr	AAEL00466	3:2470013	0	25	FALSO	3
-8,31E+18	FALSO	High	IsMasterPr	AAEL01057	2:1231305	0	23	FALSO	2
9,02E+18	FALSO	High	IsMasterPr	AAEL00291	1:1082425	0	9	FALSO	3
-8,13E+18	FALSO	High	IsMasterPr	AAEL01311	2:1791542	0	5	FALSO	1
-4,09E+18	FALSO	High	IsMasterPr	AAEL00602	3:3361718	0	2	FALSO	2
6,96E+17	FALSO	High	IsMasterPr	AAEL00548	3:6013088	0	8	FALSO	1
7,22E+18	FALSO	High	IsMasterPr	AAEL00592	3:1386469	0	14	FALSO	5
-5,22E+18	FALSO	High	IsMasterPr	AAEL01317	2:3891354	0	26	FALSO	4
2,33E+18	FALSO	High	IsMasterPr	AAEL00147	2:2645339	0	3	FALSO	2
-8,28E+18	FALSO	High	IsMasterPr	AAEL00217	1:1022335	0	15	FALSO	3
-8,87E+17	FALSO	High	IsMasterPr	AAEL00873	3:2811796	0	11	FALSO	1
3,08E+18	FALSO	High	IsMasterPr	AAEL00353	3:2640909	0	6	FALSO	3

7,61E+18	FALSO	High	IsMasterPr	AAEL01461	3:3685024	0	9	FALSO	4
2,64E+18	FALSO	High	IsMasterPr	AAEL00354	3:2640591	0	16	FALSO	3
-5,21E+16	FALSO	High	IsMasterPr	AAEL0273	2:8635902	0	53	FALSO	2
8,98E+18	FALSO	High	IsMasterPr	AAEL00802	1:5184317	0	7	FALSO	1
-5,21E+18	FALSO	High	IsMasterPr	AAEL0006	3:3950882	0	15	FALSO	1
7,36E+18	FALSO	High	IsMasterPr	AAEL02507	1:1466018	0	13	FALSO	3
9,05E+18	FALSO	High	IsMasterPr	AAEL01301	1:1630242	0	22	FALSO	2
5,39E+18	FALSO	High	IsMasterPr	AAEL0255	3:4888927	0	1	FALSO	3
7,41E+17	FALSO	High	IsMasterPr	AAEL00014	1:1949475	0	23	FALSO	3
-6,59E+18	FALSO	High	IsMasterPr	AAEL0267	2:5921429	0	13	FALSO	4
-6,08E+17	FALSO	High	IsMasterPr	AAEL0097	3:1557164	0	6	FALSO	2
6,74E+18	FALSO	High	IsMasterPr	AAEL00637	1:1015431	0	8	FALSO	2
-3,31E+17	FALSO	High	IsMasterPr	AAEL0104	3:3135775	0	21	FALSO	2
1,63E+18	FALSO	High	IsMasterPr	AAEL02352	3:1217004	0	15	FALSO	2
-7,73E+18	FALSO	High	IsMasterPr	AAEL01437	3:8673321	0	11	FALSO	3
-3,88E+18	FALSO	High	IsMasterPr	AAEL02024	1:2673438	0	11	FALSO	3
-4,70E+18	FALSO	High	IsMasterPr	AAEL02528	NIGP0100	0	4	FALSO	1
-6,67E+18	FALSO	High	IsMasterPr	AAEL01993	2:4420121	0	1	FALSO	3
7,62E+18	FALSO	High	IsMasterPr	AAEL0001	3:2130088	0	6	FALSO	3
5,88E+18	FALSO	High	IsMasterPr	AAEL00573	2:4373018	0	3	FALSO	1
-5,66E+18	FALSO	High	IsMasterPr	AAEL01353	2:2460133	0	5	FALSO	2
2,01E+18	FALSO	High	IsMasterPr	AAEL00391	1:2506384	0	9	FALSO	3
4,37E+18	FALSO	High	IsMasterPr	AAEL0041	2:1463725	0	4	FALSO	3
7,34E+18	FALSO	High	IsMasterPr	AAEL0018	2:3894362	0	15	FALSO	4
-6,16E+18	FALSO	High	IsMasterPr	AAEL01162	3:2815872	0	7	FALSO	1
7,67E+18	FALSO	High	IsMasterPr	AAEL00194	2:1933883	0	13	FALSO	2
-3,53E+18	FALSO	High	IsMasterPr	AAEL00581	1:2012065	0	8	FALSO	3
8,01E+18	FALSO	High	IsMasterPr	AAEL01258	2:3279707	0	21	FALSO	4
4,77E+18	FALSO	High	IsMasterPr	AAEL02434	1:5515361	0	9	FALSO	3
-5,59E+18	FALSO	High	IsMasterPr	AAEL00082	3:3620409	0	4	FALSO	3
-4,85E+18	FALSO	High	IsMasterPr	AAEL01338	3:1731485	0	8	FALSO	4
8,98E+18	FALSO	High	IsMasterPr	AAEL00021	1:6592495	0	21	FALSO	4
-8,44E+18	FALSO	High	IsMasterPr	AAEL0069	2:3406146	0	8	FALSO	4
3,73E+18	FALSO	High	IsMasterPr	AAEL0030	2:2135721	0	6	FALSO	3
8,46E+18	FALSO	High	IsMasterPr	AAEL00832	2:2741843	0	9	FALSO	2
1,63E+17	FALSO	High	IsMasterPr	AAEL00947	1:9770190	0	4	FALSO	6
-7,18E+18	FALSO	High	IsMasterPr	AAEL0069	1:1573803	0	9	FALSO	4
-5,22E+18	FALSO	High	IsMasterPr	AAEL0229	1:2846364	0	13	FALSO	1
-1,30E+17	FALSO	High	IsMasterPr	AAEL00441	3:2711021	0	48	FALSO	4
-3,95E+18	FALSO	High	IsMasterPr	AAEL02817	3:3374006	0	9	FALSO	2
-2,53E+18	FALSO	High	IsMasterPr	AAEL00198	2:4259454	0	16	FALSO	1
-4,00E+18	FALSO	High	IsMasterPr	AAEL00771	2:4703068	0	20	FALSO	3
-6,08E+18	FALSO	High	IsMasterPr	AAEL0011	3:4071395	0	32	FALSO	3
8,79E+18	FALSO	High	IsMasterPr	AAEL0030	2:2128482	0	8	FALSO	1
-8,40E+18	FALSO	High	IsMasterPr	AAEL01454	1:2176046	0	4	FALSO	2
3,32E+17	FALSO	High	IsMasterPr	AAEL00732	2:3451264	0	9	FALSO	2
-2,75E+18	FALSO	High	IsMasterPr	AAEL0089	1:8152441	0	23	FALSO	4
6,47E+18	FALSO	High	IsMasterPr	AAEL00342	3:3493164	0	16	FALSO	3
-2,71E+18	FALSO	High	IsMasterPr	AAEL0122	2:3843670	0	3	FALSO	2
-8,67E+18	FALSO	High	IsMasterPr	AAEL0071	3:1909085	0	3	FALSO	3

-2,19E+18	FALSO	High	IsMasterPr	AAEL00278	3:12931070	0	7	FALSO	3
-4,63E+17	FALSO	High	IsMasterPr	AAEL02038	1:20667485	0	4	FALSO	2
-8,96E+17	FALSO	High	IsMasterPr	AAEL01246	1:12329755	0	15	FALSO	4
-3,84E+18	FALSO	High	IsMasterPr	AAEL02419	2:11583051	0	6	FALSO	3
1,34E+18	FALSO	High	IsMasterPr	AAEL00509	3:82780743	0	10	FALSO	3
2,24E+18	FALSO	High	IsMasterPr	AAEL02205	NIGP01002	0	8	FALSO	3
7,41E+18	FALSO	High	IsMasterPr	AAEL01250	2:76225751	0	7	FALSO	2
-3,23E+18	FALSO	High	IsMasterPr	AAEL00697	2:39910010	0	8	FALSO	3
3,46E+18	FALSO	High	IsMasterPr	AAEL01389	3:17043448	0	10	FALSO	4
3,72E+18	FALSO	High	IsMasterPr	AAEL00773	2:10086588	0	7	FALSO	2
-6,18E+18	FALSO	High	IsMasterPr	AAEL01818	3:22124364	0	5	FALSO	3
3,47E+17	FALSO	High	IsMasterPr	AAEL02045	3:87526005	0	22	FALSO	2
-6,50E+18	FALSO	High	IsMasterPr	AAEL00655	2:59363790	0	35	FALSO	4
8,80E+18	FALSO	High	IsMasterPr	AAEL01821	1:29460134	0	6	FALSO	2
-8,75E+18	FALSO	High	IsMasterPr	AAEL01958	3:26827757	0	14	FALSO	4
-8,40E+18	FALSO	High	IsMasterPr	AAEL00833	1:23542843	0	1	FALSO	2
5,31E+18	FALSO	High	IsMasterPr	AAEL00041	3:18597029	0	33	FALSO	3
8,86E+18	FALSO	High	IsMasterPr	AAEL01017	3:14867630	0	7	FALSO	4
7,98E+18	FALSO	High	IsMasterPr	AAEL00876	3:32650347	0	19	FALSO	1
2,55E+18	FALSO	High	IsMasterPr	AAEL01029	3:40927920	1	12	FALSO	2
4,36E+18	FALSO	High	IsMasterPr	AAEL01005	1:15956915	1	12	FALSO	3
-6,25E+18	FALSO	High	IsMasterPr	AAEL00517	2:757171:9	1	4	FALSO	5
4,84E+17	FALSO	High	IsMasterPr	AAEL01372	1:22003744	1	3	FALSO	2
7,78E+17	FALSO	High	IsMasterPr	AAEL02545	3:14764611	1	10	FALSO	2
-9,12E+18	FALSO	High	IsMasterPr	AAEL00474	2:28921389	1	5	FALSO	4
-7,39E+18	FALSO	High	IsMasterPr	AAEL01209	2:13973038	1	3	FALSO	2
-6,82E+18	FALSO	High	IsMasterPr	M9WUN3	Putative ou	1	8	FALSO	3
8,44E+18	FALSO	High	IsMasterPr	AAEL00854	3:29346321	1	18	FALSO	2
-9,14E+18	FALSO	High	IsMasterPr	AAEL02483	2:86366324	1	34	FALSO	1
-5,94E+18	FALSO	High	IsMasterPr	AAEL01821	1:22352091	1	1	FALSO	2
-1,76E+18	FALSO	High	IsMasterPr	AAEL00673	2:83711661	1	12	FALSO	4
4,53E+18	FALSO	High	IsMasterPr	AAEL00535	3:34493440	1	57	FALSO	4
4,19E+16	FALSO	High	IsMasterPr	AAEL00236	2:30312461	1	3	FALSO	2
-3,82E+18	FALSO	High	IsMasterPr	AAEL01192	3:35087210	1	16	FALSO	3
6,47E+18	FALSO	High	IsMasterPr	AAEL00200	2:42604320	1	5	FALSO	1
1,58E+18	FALSO	High	IsMasterPr	AAEL02246	1:24184505	1	2	FALSO	2
-1,09E+17	FALSO	High	IsMasterPr	AAEL00083	3:36378331	1	13	FALSO	4
-2,90E+18	FALSO	High	IsMasterPr	AAEL01479	2:32429548	1	13	FALSO	1
-7,66E+18	FALSO	High	IsMasterPr	AAEL01449	2:46471471	1	12	FALSO	1
8,86E+18	FALSO	High	IsMasterPr	AAEL01210	1:185220:1	1	4	FALSO	3
-7,24E+18	FALSO	High	IsMasterPr	AAEL00757	NIGP01000	1	7	FALSO	3
4,18E+18	FALSO	High	IsMasterPr	AAEL00863	2:45488258	1	12	FALSO	3
-6,10E+18	FALSO	High	IsMasterPr	AAEL00319	1:19208631	1	8	FALSO	4
5,90E+18	FALSO	High	IsMasterPr	AAEL02457	2:78264820	1	1	FALSO	2
-2,14E+18	FALSO	High	IsMasterPr	AAEL00503	2:44604618	1	7	FALSO	1
-4,11E+18	FALSO	High	IsMasterPr	AAEL00293	1:86045397	1	14	FALSO	4
-2,43E+18	FALSO	High	IsMasterPr	AAEL02002	2:12193694	1	3	FALSO	1
4,14E+18	FALSO	High	IsMasterPr	AAEL00522	3:90581368	1	41	FALSO	4
4,91E+18	FALSO	High	IsMasterPr	AAEL01079	1:17852287	1	8	FALSO	3
-8,73E+18	FALSO	High	IsMasterPr	AAEL01013	2:26467631	1	26	FALSO	2

-5,59E+18	FALSO	High	IsMasterPr	AAEL01307	1:3173777	1	14	FALSO	3
-5,73E+18	FALSO	High	IsMasterPr	AAEL00744	2:2913552	1	6	FALSO	1
-1,73E+18	FALSO	High	IsMasterPr	AAEL01496	2:1152422	1	18	FALSO	4
-8,71E+18	FALSO	High	IsMasterPr	AAEL00986	2:1645615	1	2	FALSO	1
-1,86E+18	FALSO	High	IsMasterPr	AAEL00837	3:4075311	1	6	FALSO	2
7,04E+18	FALSO	High	IsMasterPr	AAEL02522	2:3341331	1	5	FALSO	3
-3,51E+18	FALSO	High	IsMasterPr	AAEL00585	3:2187321	1	6	FALSO	2
-1,79E+18	FALSO	High	IsMasterPr	AAEL01313	2:1884760	1	12	FALSO	3
1,36E+18	FALSO	High	IsMasterPr	AAEL01464	2:3544351	1	10	FALSO	2
-3,39E+18	FALSO	High	IsMasterPr	AAEL01506	2:1918168	1	5	FALSO	1
4,86E+18	FALSO	High	IsMasterPr	AAEL00777	2:2197547	1	13	FALSO	2
3,66E+18	FALSO	High	IsMasterPr	AAEL00556	3:43502:44	1	4	FALSO	2
-4,82E+18	FALSO	High	IsMasterPr	AAEL00605	3:7370900	1	8	FALSO	3
3,65E+18	FALSO	High	IsMasterPr	AAEL00491	2:7241999	1	3	FALSO	2
-1,10E+18	FALSO	High	IsMasterPr	AAEL01990	3:1094787	1	2	FALSO	2
-6,88E+17	FALSO	High	IsMasterPr	AAEL01445	2:3220016	3	4	FALSO	2
-5,55E+18	FALSO	High	IsMasterPr	AAEL00270	3:1255636	3	18	FALSO	2
2,82E+18	FALSO	High	IsMasterPr	AAEL01147	3:1426797	3	15	FALSO	3
5,98E+18	FALSO	High	IsMasterPr	AAEL02778	3:3711085	3	4	FALSO	4
-3,64E+18	FALSO	High	IsMasterPr	AAEL01032	3:4090620	3	13	FALSO	3
4,42E+18	FALSO	High	IsMasterPr	AAEL00606	1:1841657	3	6	FALSO	1
-3,02E+18	FALSO	High	IsMasterPr	AAEL00073	2:5567807	3	5	FALSO	3
5,52E+18	FALSO	High	IsMasterPr	AAEL00211	3:1775942	3	20	FALSO	2
-4,26E+18	FALSO	High	IsMasterPr	AAEL00655	2:5848746	3	8	FALSO	3
-6,57E+18	FALSO	High	IsMasterPr	AAEL00981	3:2026648	3	8	FALSO	2
7,30E+18	FALSO	High	IsMasterPr	AAEL01156	3:1066335	3	7	FALSO	2
-7,19E+17	FALSO	High	IsMasterPr	AAEL00388	3:8509119	3	3	FALSO	2
8,02E+18	FALSO	High	IsMasterPr	AAEL01832	3:1626316	3	3	FALSO	3
2,19E+18	FALSO	High	IsMasterPr	AAEL00544	2:2958239	3	10	FALSO	3
-3,69E+18	FALSO	High	IsMasterPr	AAEL00272	2:1381470	3	9	FALSO	2
4,30E+18	FALSO	High	IsMasterPr	AAEL00346	2:1532166	3	3	FALSO	1
6,99E+18	FALSO	High	IsMasterPr	AAEL00227	2:3141217	3	5	FALSO	2
9,01E+18	FALSO	High	IsMasterPr	AAEL00780	3:1526509	3	4	FALSO	1
8,66E+18	FALSO	High	IsMasterPr	AAEL00177	2:1657035	3	10	FALSO	1
-3,70E+18	FALSO	High	IsMasterPr	AAEL00136	2:2044890	3	20	FALSO	1
5,86E+18	FALSO	High	IsMasterPr	AAEL01179	3:2887496	3	16	FALSO	2
6,78E+18	FALSO	High	IsMasterPr	AAEL02250	1:2832129	3	7	FALSO	2
4,56E+18	FALSO	High	IsMasterPr	AAEL01413	1:468596:4	3	10	FALSO	3
-8,88E+18	FALSO	High	IsMasterPr	AAEL00605	3:7432810	3	10	FALSO	3
9,17E+18	FALSO	High	IsMasterPr	AAEL00094	3:3771175	3	19	FALSO	2
-3,03E+18	FALSO	High	IsMasterPr	AAEL01988	2:5786664	3	6	FALSO	3
2,72E+18	FALSO	High	IsMasterPr	AAEL00055	3:3862349	3	21	FALSO	3
7,42E+17	FALSO	High	IsMasterPr	AAEL02125	2:2281335	3	9	FALSO	5
-6,78E+18	FALSO	High	IsMasterPr	AAEL01363	2:3243727	3	8	FALSO	3
9,11E+18	FALSO	High	IsMasterPr	AAEL00196	2:4239736	3	6	FALSO	2
6,28E+18	FALSO	High	IsMasterPr	AAEL01030	3:4093330	3	5	FALSO	1
2,77E+18	FALSO	High	IsMasterPr	AAEL01281	3:7558578	3	28	FALSO	2
-7,74E+18	FALSO	High	IsMasterPr	AAEL00407	1:1353858	3	10	FALSO	2
-2,73E+18	FALSO	High	IsMasterPr	AAEL00103	3:5569880	3	13	FALSO	2
-3,45E+18	FALSO	High	IsMasterPr	AAEL02122	3:2936743	3	6	FALSO	3

-8,47E+18	FALSO	High	IsMasterPr	AAEL0139€	2:2412875(	3	8	FALSO	2
6,21E+18	FALSO	High	IsMasterPr	AAEL0022C	2:4307878:	3	24	FALSO	3
-2,00E+18	FALSO	High	IsMasterPr	AAEL01973	3:2007455!	3	2	FALSO	4
8,67E+18	FALSO	High	IsMasterPr	AAEL01344	2:1554229:	3	4	FALSO	2
-9,01E+18	FALSO	High	IsMasterPr	AAEL0098€	2:1640537!	3	9	FALSO	2
4,78E+18	FALSO	High	IsMasterPr	AAEL0198!	1:6432634!	3	4	FALSO	2
-9,31E+17	FALSO	High	IsMasterPr	AAEL01427	1:8378452:	3	9	FALSO	1
-3,12E+18	FALSO	High	IsMasterPr	AAEL01541	1:1907989!	3	7	FALSO	3
-4,49E+18	FALSO	High	IsMasterPr	A0A4S2QR|	Malate deh	3	27	FALSO	3
-7,31E+18	FALSO	High	IsMasterPr	AAEL00984	3:1892548:	3	11	FALSO	1
2,82E+18	FALSO	High	IsMasterPr	AAEL0052C	2:229031:2	3	10	FALSO	2
1,74E+18	FALSO	High	IsMasterPr	AAEL02272	2:1472645:	3	6	FALSO	2
3,52E+18	FALSO	High	IsMasterPr	AAEL0097ٍ8	1:2761093!	3	18	FALSO	3
8,76E+18	FALSO	High	IsMasterPr	AAEL01834	3:3833563!	3	4	FALSO	3
-2,78E+18	FALSO	High	IsMasterPr	AAEL01067	2:1360175!	3	9	FALSO	2
7,67E+18	FALSO	High	IsMasterPr	Q9L6V2	Cell divisioı	3	13	FALSO	1
8,05E+18	FALSO	High	IsMasterPr	AAEL0078!	2:4739446:	3	2	FALSO	1
-8,42E+18	FALSO	High	IsMasterPr	AAEL00402	3:1663721!	3	8	FALSO	2
5,93E+18	FALSO	High	IsMasterPr	AAEL0150€	2:2103900!	3	11	FALSO	2
5,42E+18	FALSO	High	IsMasterPr	AAEL0235!	NIGP01001	3	17	FALSO	2
-4,24E+18	FALSO	High	IsMasterPr	AAEL00078	3:2451418:	3	45	FALSO	2
-1,84E+18	FALSO	High	IsMasterPr	AAEL00754	2:3271176:	3	8	FALSO	2
7,62E+18	FALSO	High	IsMasterPr	AAEL0213C	2:4027848(	3	5	FALSO	2
6,46E+18	FALSO	High	IsMasterPr	AAEL0123!	3:3656062(	3	11	FALSO	1
-5,06E+17	FALSO	High	IsMasterPr	AAEL0227€	NIGP0100C	3	5	FALSO	2
8,47E+18	FALSO	High	IsMasterPr	AAEL00194	2:1944269:	3	20	FALSO	3
-2,10E+18	FALSO	High	IsMasterPr	AAEL0205!	2:1762998:	3	6	FALSO	2
2,17E+18	FALSO	High	IsMasterPr	AAEL00991	1:2067016!	3	6	FALSO	2
-4,50E+17	FALSO	High	IsMasterPr	AAEL00278	1:3052250(	3	13	FALSO	3
-6,51E+18	FALSO	High	IsMasterPr	AAEL00574	2:4361914:	3	8	FALSO	4
8,80E+18	FALSO	High	IsMasterPr	AAEL0107€	2:3046871(	3	9	FALSO	2
1,95E+18	FALSO	High	IsMasterPr	AAEL02302	1:7318510!	3	20	FALSO	1
-7,71E+18	FALSO	High	IsMasterPr	AAEL0104!	2:4463787	3	6	FALSO	2
-1,74E+18	FALSO	High	IsMasterPr	AAEL00067	2:4596819:	3	24	FALSO	1
-2,34E+18	FALSO	High	IsMasterPr	AAEL02294	2:7873901:	3	9	FALSO	3
4,47E+17	FALSO	High	IsMasterPr	AAEL0098!	3:1864921(	3	13	FALSO	1
-9,31E+16	FALSO	High	IsMasterPr	AAEL0083ٍ8	3:2248863:	3	7	FALSO	4
-2,34E+18	FALSO	High	IsMasterPr	AAEL00873	3:2813274:	3	7	FALSO	2
-3,39E+18	FALSO	High	IsMasterPr	AAEL0031ٍ8	NIGP01001	3	3	FALSO	2
1,56E+18	FALSO	High	IsMasterPr	AAEL00392	1:2505740(	3	23	FALSO	2
3,77E+18	FALSO	High	IsMasterPr	AAEL01952	2:1617198:	3	11	FALSO	2
-3,87E+18	FALSO	High	IsMasterPr	AAEL00883	2:2415124(	3	5	FALSO	1
1,84E+18	FALSO	High	IsMasterPr	AAEL0007C	2:4587253	3	19	FALSO	2
5,37E+18	FALSO	High	IsMasterPr	AAEL01173	3:2908918:	3	4	FALSO	3
5,12E+18	FALSO	High	IsMasterPr	AAEL0076€	1:4911236:	3	10	FALSO	2
3,50E+18	FALSO	High	IsMasterPr	AAEL00137	2:2062325:	3	10	FALSO	3
8,35E+18	FALSO	High	IsMasterPr	AAEL0000€	1:2430393	3	13	FALSO	3
7,33E+18	FALSO	High	IsMasterPr	AAEL00012	2:1100675!	3	6	FALSO	2
-8,04E+18	FALSO	High	IsMasterPr	AAEL0099!	3:2869677(	3	4	FALSO	3
4,65E+18	FALSO	High	IsMasterPr	AAEL00017	3:2158361:	3	18	FALSO	2

-7,87E+18	FALSO	High	IsMasterPr	AAEL00067	3:3951049	3	13	FALSO	2
4,62E+18	FALSO	High	IsMasterPr	AAEL01471	1:2354474	3	15	FALSO	2
1,10E+18	FALSO	High	IsMasterPr	AAEL01331	3:1001903	3	13	FALSO	2
-5,63E+18	FALSO	High	IsMasterPr	AAEL00286	1:2560241	3	3	FALSO	1
1,42E+18	FALSO	High	IsMasterPr	AAEL00952	3:2238477	3	28	FALSO	2
-7,62E+17	FALSO	High	IsMasterPr	AAEL01105	3:2945825	3	4	FALSO	1
5,54E+18	FALSO	High	IsMasterPr	AAEL02710	1:1086415	3	5	FALSO	2
-6,61E+18	FALSO	High	IsMasterPr	AAEL00600	2:4722436	3	7	FALSO	1
9,23E+17	FALSO	High	IsMasterPr	AAEL00915	1:1126399	3	12	FALSO	3
5,21E+18	FALSO	High	IsMasterPr	AAEL00651	2:4690473	3	5	FALSO	1
-1,52E+17	FALSO	High	IsMasterPr	AAEL00728	3:1955393	3	14	FALSO	3
-6,68E+18	FALSO	High	IsMasterPr	AAEL00063	2:4075606	3	29	FALSO	2
5,28E+18	FALSO	High	IsMasterPr	AAEL00399	3:2539071	3	15	FALSO	2
-7,62E+18	FALSO	High	IsMasterPr	AAEL02108	1:1077452	3	2	FALSO	2
-3,91E+18	FALSO	High	IsMasterPr	AAEL01396	3:1064600	3	9	FALSO	4
5,26E+18	FALSO	High	IsMasterPr	AAEL00508	1:2982749	3	12	FALSO	4
5,67E+18	FALSO	High	IsMasterPr	AAEL00619	1:2247763	3	11	FALSO	4
5,07E+18	FALSO	High	IsMasterPr	AAEL02597	2:1664154	3	2	FALSO	2
4,23E+18	FALSO	High	IsMasterPr	AAEL00651	2:4689257	3	4	FALSO	2
8,56E+18	FALSO	High	IsMasterPr	AAEL00969	3:3586759	3	14	FALSO	3
6,08E+18	FALSO	High	IsMasterPr	AAEL00453	2:3098508	3	2	FALSO	1
2,60E+18	FALSO	High	IsMasterPr	AAEL01438	1:2387463	3	8	FALSO	2
-7,81E+18	FALSO	High	IsMasterPr	AAEL01988	3:3433510	3	3	FALSO	2
-1,62E+18	FALSO	High	IsMasterPr	AAEL00428	2:2931158	3	9	FALSO	3
1,91E+18	FALSO	High	IsMasterPr	AAEL01951	3:2025783	3	2	FALSO	3
6,23E+18	FALSO	High	IsMasterPr	AAEL00095	3:3800834	3	22	FALSO	1
6,01E+18	FALSO	High	IsMasterPr	AAEL01953	3:3614369	3	5	FALSO	2
-6,08E+18	FALSO	High	IsMasterPr	AAEL00245	1:9870791	3	3	FALSO	2
4,50E+17	FALSO	High	IsMasterPr	AAEL00601	3:3153264	3	7	FALSO	2
-5,94E+18	FALSO	High	IsMasterPr	AAEL00602	3:3147359	3	13	FALSO	2
-2,62E+18	FALSO	High	IsMasterPr	AAEL01134	2:9262543	3	13	FALSO	4
-3,17E+18	FALSO	High	IsMasterPr	AAEL01968	2:3548304	3	5	FALSO	3
2,15E+18	FALSO	High	IsMasterPr	AAEL00995	2:9334095	3	2	FALSO	4
-1,68E+18	FALSO	High	IsMasterPr	AAEL00833	2:2740873	3	10	FALSO	2
-9,22E+18	FALSO	High	IsMasterPr	AAEL00989	3:1219665	3	6	FALSO	2
-7,08E+18	FALSO	High	IsMasterPr	AAEL01751	2:5878038	3	7	FALSO	2
-8,28E+18	FALSO	High	IsMasterPr	AAEL00025	3:2656199	3	9	FALSO	2
-5,40E+18	FALSO	High	IsMasterPr	AAEL00164	2:3004566	3	29	FALSO	4
-6,02E+18	FALSO	High	IsMasterPr	AAEL00650	2:4677792	3	6	FALSO	1
6,62E+18	FALSO	High	IsMasterPr	AAEL01501	2:1219493	3	4	FALSO	2
-7,50E+18	FALSO	High	IsMasterPr	AAEL00915	1:1128155	3	3	FALSO	3
6,66E+18	FALSO	High	IsMasterPr	AAEL00392	2:3545905	3	4	FALSO	1
8,19E+18	FALSO	High	IsMasterPr	AAEL00491	2:2608288	3	4	FALSO	1
6,83E+18	FALSO	High	IsMasterPr	AAEL00140	2:4827705	3	6	FALSO	2
-2,99E+18	FALSO	High	IsMasterPr	AAEL00806	2:2309333	3	1	FALSO	2
-8,09E+18	FALSO	High	IsMasterPr	AAEL02074	3:3102186	3	10	FALSO	1
-7,54E+18	FALSO	High	IsMasterPr	AAEL00771	3:2553836	3	20	FALSO	3
-1,57E+18	FALSO	High	IsMasterPr	AAEL02702	2:4386338	3	22	FALSO	1
-2,23E+18	FALSO	High	IsMasterPr	AAEL00729	3:1957768	3	8	FALSO	2
2,07E+18	FALSO	High	IsMasterPr	AAEL00904	3:4609003	3	8	FALSO	1

-5,76E+18	FALSO	High	IsMasterPr	AAEL01953	1:2389682	3	3	FALSO	1
-4,84E+18	FALSO	High	IsMasterPr	AAEL02142	2:3602714	3	4	FALSO	3
9,18E+18	FALSO	High	IsMasterPr	AAEL01381	1:3582790	3	4	FALSO	2
-1,22E+18	FALSO	High	IsMasterPr	AAEL00910	3:1353436	3	6	FALSO	1
-5,63E+18	FALSO	High	IsMasterPr	AAEL01958	3:2452819	3	1	FALSO	1
1,53E+18	FALSO	High	IsMasterPr	AAEL00708	2:1281228	3	15	FALSO	3
-4,89E+18	FALSO	High	IsMasterPr	AAEL00954	3:2239264	3	18	FALSO	1
-5,06E+18	FALSO	High	IsMasterPr	AAEL01024	3:2790924	3	23	FALSO	2
-4,45E+18	FALSO	High	IsMasterPr	AAEL00676	3:2407870	3	22	FALSO	2
7,12E+18	FALSO	High	IsMasterPr	AAEL01094	3:1682064	3	21	FALSO	2
7,42E+18	FALSO	High	IsMasterPr	AAEL00679	3:3681259	2	5	FALSO	2
1,47E+18	FALSO	High	IsMasterPr	AAEL00948	1:9832949	2	4	FALSO	1
7,17E+17	FALSO	High	IsMasterPr	AAEL02763	NIGP01001	2	37	FALSO	1
3,96E+18	FALSO	High	IsMasterPr	AAEL01975	3:1272405	2	10	FALSO	2
-5,03E+18	FALSO	High	IsMasterPr	AAEL01303	3:2086260	2	4	FALSO	1
-5,13E+18	FALSO	High	IsMasterPr	AAEL01086	3:3191735	2	2	FALSO	3
-7,04E+18	FALSO	High	IsMasterPr	AAEL02466	3:1049496	2	3	FALSO	1
5,69E+17	FALSO	High	IsMasterPr	AAEL01142	3:3288796	2	7	FALSO	4
1,72E+18	FALSO	High	IsMasterPr	AAEL00593	3:1383104	2	24	FALSO	1
-2,37E+18	FALSO	High	IsMasterPr	AAEL00894	1:8150210	2	7	FALSO	2
7,89E+18	FALSO	High	IsMasterPr	AAEL00603	3:3335834	2	9	FALSO	2
-6,84E+18	FALSO	High	IsMasterPr	AAEL00775	1:7185744	2	27	FALSO	2
7,36E+18	FALSO	High	IsMasterPr	AAEL00385	3:3426596	2	3	FALSO	2
1,82E+18	FALSO	High	IsMasterPr	AAEL01115	3:1941055	2	8	FALSO	3
-9,98E+17	FALSO	High	IsMasterPr	AAEL00956	3:1195902	2	9	FALSO	1
2,26E+18	FALSO	High	IsMasterPr	AAEL00412	3:4035034	2	7	FALSO	1
1,73E+18	FALSO	High	IsMasterPr	AAEL01339	3:1731120	2	13	FALSO	2
3,66E+18	FALSO	High	IsMasterPr	AAEL01230	2:1029501	2	6	FALSO	2
5,76E+18	FALSO	High	IsMasterPr	AAEL00214	1:2535815	2	23	FALSO	2
-3,53E+18	FALSO	High	IsMasterPr	AAEL00222	2:4292286	2	1	FALSO	1
2,88E+18	FALSO	High	IsMasterPr	AAEL01352	1:1440050	2	12	FALSO	1
1,97E+18	FALSO	High	IsMasterPr	AAEL01406	2:4059387	2	22	FALSO	3
-7,07E+18	FALSO	High	IsMasterPr	AAEL01349	2:1991259	2	9	FALSO	1
2,76E+18	FALSO	High	IsMasterPr	AAEL00557	3:4051322	2	12	FALSO	3
2,07E+18	FALSO	High	IsMasterPr	AAEL00788	3:3811035	2	8	FALSO	1
6,76E+18	FALSO	High	IsMasterPr	AAEL01081	3:5209200	2	5	FALSO	3
1,18E+18	FALSO	High	IsMasterPr	AAEL00546	3:3837972	2	2	FALSO	1
-9,34E+16	FALSO	High	IsMasterPr	AAEL00692	3:1438982	2	14	FALSO	2
-5,86E+18	FALSO	High	IsMasterPr	AAEL02557	3:1406485	2	6	FALSO	1
3,65E+18	FALSO	High	IsMasterPr	AAEL02257	1:1889016	2	3	FALSO	2
3,04E+18	FALSO	High	IsMasterPr	AAEL01415	3:3022274	2	20	FALSO	3
3,11E+18	FALSO	High	IsMasterPr	AAEL00785	2:4609610	2	2	FALSO	1
-7,10E+17	FALSO	High	IsMasterPr	AAEL02736	1:1847186	2	14	FALSO	2
-2,52E+18	FALSO	High	IsMasterPr	AAEL01728	3:1368237	2	2	FALSO	3
2,86E+16	FALSO	High	IsMasterPr	AAEL00057	3:1247193	2	9	FALSO	2
6,75E+18	FALSO	High	IsMasterPr	AAEL00974	2:4436095	2	3	FALSO	2
-6,19E+18	FALSO	High	IsMasterPr	AAEL00042	3:1868580	2	10	FALSO	2
-3,69E+18	FALSO	High	IsMasterPr	AAEL00307	2:2135264	2	5	FALSO	3
6,75E+18	FALSO	High	IsMasterPr	AAEL00472	2:3792274	2	3	FALSO	2
-4,34E+18	FALSO	High	IsMasterPr	AAEL00235	1:1036232	2	2	FALSO	4

-8,47E+18	FALSO	High	IsMasterPr	AAEL00697	2:39873610	2	11	FALSO	2
-3,76E+18	FALSO	High	IsMasterPr	AAEL02901	2:12627307	2	16	FALSO	1
-2,07E+18	FALSO	High	IsMasterPr	AAEL00826	2:23479399	2	18	FALSO	1
-3,25E+18	FALSO	High	IsMasterPr	AAEL00710	2:16325397	2	13	FALSO	3
-5,68E+18	FALSO	High	IsMasterPr	AAEL02297	2:34245113	2	10	FALSO	2
8,38E+18	FALSO	High	IsMasterPr	AAEL00116	2:30723464	3	12	FALSO	3
-6,03E+18	FALSO	High	IsMasterPr	AAEL02309	3:31259643	3	5	FALSO	2
-4,96E+17	FALSO	High	IsMasterPr	AAEL00544	1:80269981	3	15	FALSO	2
5,53E+18	FALSO	High	IsMasterPr	AAEL01078	1:17816799	3	10	FALSO	1
5,13E+18	FALSO	High	IsMasterPr	AAEL01384	1:18145527	3	10	FALSO	3
-6,50E+18	FALSO	High	IsMasterPr	AAEL02099	3:13817611	3	2	FALSO	2
-6,56E+16	FALSO	High	IsMasterPr	AAEL01125	NIGP0100C	3	5	FALSO	1
7,44E+18	FALSO	High	IsMasterPr	AAEL00691	3:14371335	3	3	FALSO	2
7,04E+18	FALSO	High	IsMasterPr	AAEL00238	2:30220237	3	9	FALSO	2
3,63E+18	FALSO	High	IsMasterPr	AAEL00207	2:35670931	3	3	FALSO	5
-3,56E+18	FALSO	High	IsMasterPr	AAEL00973	2:68616159	3	13	FALSO	2
1,36E+18	FALSO	High	IsMasterPr	AAEL00290	1:10876870	3	12	FALSO	2
6,73E+18	FALSO	High	IsMasterPr	AAEL00874	3:28044560	3	4	FALSO	3
8,47E+18	FALSO	High	IsMasterPr	AAEL02599	1:43379189	3	3	FALSO	2
-6,33E+18	FALSO	High	IsMasterPr	AAEL02032	2:14983568	3	10	FALSO	2
8,56E+18	FALSO	High	IsMasterPr	AAEL00997	2:93061010	3	11	FALSO	2
1,32E+18	FALSO	High	IsMasterPr	AAEL00272	2:13816017	3	18	FALSO	2
6,08E+18	FALSO	High	IsMasterPr	AAEL00121	2:41326651	3	18	FALSO	2
-2,53E+18	FALSO	High	IsMasterPr	AAEL00620	3:15050170	3	1	FALSO	1
2,06E+18	FALSO	High	IsMasterPr	AAEL01327	2:34976051	3	7	FALSO	3
2,04E+18	FALSO	High	IsMasterPr	AAEL01458	2:27781351	3	22	FALSO	3
4,23E+18	FALSO	High	IsMasterPr	AAEL00705	3:29968834	3	11	FALSO	1
-1,64E+17	FALSO	High	IsMasterPr	AAEL00080	3:12049640	3	6	FALSO	2
-1,82E+18	FALSO	High	IsMasterPr	AAEL02556	2:65180790	3	16	FALSO	3
-5,74E+18	FALSO	High	IsMasterPr	AAEL01181	3:11323328	3	10	FALSO	3
5,07E+18	FALSO	High	IsMasterPr	AAEL01452	1:15014118	3	4	FALSO	2
1,17E+18	FALSO	High	IsMasterPr	AAEL01291	3:27940339	3	9	FALSO	2
-3,81E+18	FALSO	High	IsMasterPr	AAEL00573	2:29396081	3	4	FALSO	1
6,73E+18	FALSO	High	IsMasterPr	AAEL00921	2:22842778	3	2	FALSO	1
1,98E+18	FALSO	High	IsMasterPr	AAEL00310	1:21504397	3	7	FALSO	2
-2,95E+18	FALSO	High	IsMasterPr	AAEL00345	2:15291448	3	27	FALSO	3
-5,40E+18	FALSO	High	IsMasterPr	AAEL01957	3:17741521	3	1	FALSO	2
6,84E+18	FALSO	High	IsMasterPr	AAEL00817	3:97365481	3	7	FALSO	2
-8,28E+18	FALSO	High	IsMasterPr	AAEL01093	1:13966910	3	14	FALSO	3
4,50E+18	FALSO	High	IsMasterPr	AAEL01136	1:94997801	3	5	FALSO	2
1,24E+18	FALSO	High	IsMasterPr	AAEL00756	2:24892051	3	5	FALSO	1
5,74E+18	FALSO	High	IsMasterPr	AAEL01107	2:27791107	3	8	FALSO	1
7,43E+18	FALSO	High	IsMasterPr	AAEL00128	2:44809410	3	15	FALSO	2
1,07E+18	FALSO	High	IsMasterPr	AAEL00141	2:48256821	3	8	FALSO	2
-2,93E+18	FALSO	High	IsMasterPr	AAEL00791	2:47424721	3	4	FALSO	1
6,39E+18	FALSO	High	IsMasterPr	AAEL02512	3:28888019	3	9	FALSO	2
-4,55E+18	FALSO	High	IsMasterPr	AAEL01086	2:38358115	3	9	FALSO	1
-1,69E+18	FALSO	High	IsMasterPr	AAEL01436	3:11087421	3	4	FALSO	1
6,72E+18	FALSO	High	IsMasterPr	AAEL01152	2:12318951	3	6	FALSO	1
1,29E+18	FALSO	High	IsMasterPr	AAEL00341	3:34827740	3	6	FALSO	2

5,99E+17	FALSO	High	IsMasterPr	AAEL01835	1:1163628(	3	22	FALSO	1
-7,75E+18	FALSO	High	IsMasterPr	AAEL0030C	3:3978410:	3	3	FALSO	1
5,33E+18	FALSO	High	IsMasterPr	AAEL0132C	2:1860236!	3	3	FALSO	3
6,83E+18	FALSO	High	IsMasterPr	AAEL0039C	1:2502668(	3	9	FALSO	2
-9,37E+16	FALSO	High	IsMasterPr	AAEL00329	3:3302458(	3	20	FALSO	2
-1,32E+18	FALSO	High	IsMasterPr	AAEL02047	1:3074044:	3	11	FALSO	3
8,76E+18	FALSO	High	IsMasterPr	AAEL01984	2:2206546	3	2	FALSO	1
-7,91E+17	FALSO	High	IsMasterPr	AAEL00782	2:2718323(	3	2	FALSO	1
-6,09E+18	FALSO	High	IsMasterPr	AAEL01993	3:3254061:	3	6	FALSO	2
1,68E+18	FALSO	High	IsMasterPr	AAEL01736	3:3685886	3	6	FALSO	2
-8,42E+18	FALSO	High	IsMasterPr	AAEL00708	2:1286171	3	30	FALSO	2
-3,20E+18	FALSO	High	IsMasterPr	AAEL01325	2:3997755!	3	20	FALSO	3
-1,13E+18	FALSO	High	IsMasterPr	AAEL02173	2:2147995:	3	1	FALSO	1
-6,30E+17	FALSO	High	IsMasterPr	AAEL02636	1:1844470	3	2	FALSO	1
1,19E+18	FALSO	High	IsMasterPr	AAEL0030C	3:3931382:	3	6	FALSO	1
-7,47E+18	FALSO	High	IsMasterPr	AAEL00925	2:2684035!	3	2	FALSO	1
8,98E+18	FALSO	High	IsMasterPr	AAEL00813	3:1076280(	3	7	FALSO	1
8,36E+18	FALSO	High	IsMasterPr	AAEL02465	1:1355869!	3	2	FALSO	3
-9,75E+17	FALSO	High	IsMasterPr	AAEL01024	3:2790075:	3	20	FALSO	4
2,57E+17	FALSO	High	IsMasterPr	AAEL02347	2:1960108:	3	2	FALSO	2
-6,02E+18	FALSO	High	IsMasterPr	AAEL00516	1:1682412	3	11	FALSO	1
4,61E+18	FALSO	High	IsMasterPr	AAEL02361	2:8631792:	3	17	FALSO	1
4,30E+18	FALSO	High	IsMasterPr	AAEL00672	2:8480440:	3	4	FALSO	2
5,80E+18	FALSO	High	IsMasterPr	AAEL01248	1:2715983:	3	6	FALSO	3
-1,40E+18	FALSO	High	IsMasterPr	AAEL02417	1:3272447:	3	6	FALSO	1
8,28E+18	FALSO	High	IsMasterPr	AAEL00825	3:6329335:	3	13	FALSO	1
-9,98E+17	FALSO	High	IsMasterPr	AAEL0123C	2:1028599:	3	14	FALSO	3
-3,84E+18	FALSO	High	IsMasterPr	AAEL0145C	1:6021852:	3	8	FALSO	1
-4,27E+18	FALSO	High	IsMasterPr	AAEL01081	3:5171679:	3	11	FALSO	3
9,55E+17	FALSO	High	IsMasterPr	AAEL02351	2:2459669	3	6	FALSO	1
-8,44E+18	FALSO	High	IsMasterPr	AAEL00894	1:8113687:	3	3	FALSO	1
-2,58E+18	FALSO	High	IsMasterPr	AAEL00072	2:5208975	3	11	FALSO	1
-7,22E+18	FALSO	High	IsMasterPr	AAEL01155	3:3128266	3	16	FALSO	3
-2,90E+18	FALSO	High	IsMasterPr	AAEL01972	2:3476292	3	6	FALSO	2
-4,60E+18	FALSO	High	IsMasterPr	AAEL00552	1:9064698:	3	8	FALSO	3
-2,62E+18	FALSO	High	IsMasterPr	AAEL00463	1:3071491:	3	6	FALSO	2
2,10E+18	FALSO	High	IsMasterPr	AAEL01324	2:3994506(	3	4	FALSO	2
-1,59E+18	FALSO	High	IsMasterPr	AAEL02765	1:7538850:	3	5	FALSO	1
4,48E+18	FALSO	High	IsMasterPr	AAEL00493	2:2607795!	3	5	FALSO	2
1,30E+18	FALSO	High	IsMasterPr	AAEL0039C	1:2525244(	3	7	FALSO	3
3,40E+17	FALSO	High	IsMasterPr	AAEL02001	2:1964623	3	2	FALSO	1
3,69E+18	FALSO	High	IsMasterPr	AAEL00835	2:4163038!	3	2	FALSO	1
8,77E+18	FALSO	High	IsMasterPr	AAEL01707	2:1791179(	3	2	FALSO	2
1,58E+18	FALSO	High	IsMasterPr	AAEL0124C	3:2904852:	3	13	FALSO	2
3,03E+18	FALSO	High	IsMasterPr	AAEL01212	2:4059603:	3	5	FALSO	2
4,15E+18	FALSO	High	IsMasterPr	AAEL00477	2:2904119:	3	5	FALSO	1
-9,05E+18	FALSO	High	IsMasterPr	AAEL01978	2:4326139(	3	1	FALSO	1
3,20E+18	FALSO	High	IsMasterPr	AAEL01312	2:1793341:	3	13	FALSO	1
1,58E+18	FALSO	High	IsMasterPr	AAEL01052	2:2641430:	3	24	FALSO	1
4,12E+18	FALSO	High	IsMasterPr	AAEL00675	2:8398235:	3	10	FALSO	2

5,34E+18	FALSO	High	IsMasterPr	AAEL02484	1:6822317	3	4	FALSO	1
-1,76E+18	FALSO	High	IsMasterPr	AAEL00051	3:1640326	3	11	FALSO	3
6,87E+18	FALSO	High	IsMasterPr	AAEL02739	3:2742830	3	2	FALSO	3
-6,24E+17	FALSO	High	IsMasterPr	AAEL01997	3:3637500	3	13	FALSO	2
6,70E+18	FALSO	High	IsMasterPr	AAEL01297	2:1249483	3	6	FALSO	1
1,19E+18	FALSO	High	IsMasterPr	AAEL02333	3:3599460	3	2	FALSO	2
-5,58E+18	FALSO	High	IsMasterPr	AAEL01005	1:1594217	3	6	FALSO	2
6,14E+18	FALSO	High	IsMasterPr	AAEL01109	2:2321925	3	3	FALSO	3
-1,30E+18	FALSO	High	IsMasterPr	AAEL02588	3:2642736	3	6	FALSO	1
-5,21E+18	FALSO	High	IsMasterPr	AAEL01135	1:1653932	3	6	FALSO	2
9,11E+18	FALSO	High	IsMasterPr	AAEL00986	1:9242588	3	1	FALSO	2
-2,26E+18	FALSO	High	IsMasterPr	AAEL01727	2:1868915	3	13	FALSO	2
-2,28E+18	FALSO	High	IsMasterPr	AAEL00967	3:2218664	3	7	FALSO	2
-4,52E+18	FALSO	High	IsMasterPr	AAEL00992	1:3076797	3	3	FALSO	2
2,06E+18	FALSO	High	IsMasterPr	AAEL01001	2:1105776	3	6	FALSO	3
-4,41E+17	FALSO	High	IsMasterPr	AAEL00690	2:3407218	3	7	FALSO	2
4,08E+18	FALSO	High	IsMasterPr	AAEL00437	3:9304495	3	20	FALSO	3
-2,47E+18	FALSO	High	IsMasterPr	AAEL02520	2:3320753	3	2	FALSO	1
5,69E+18	FALSO	High	IsMasterPr	AAEL00855	3:2940274	3	29	FALSO	1
-1,11E+18	FALSO	High	IsMasterPr	AAEL01385	2:1001663	3	9	FALSO	1
-6,09E+18	FALSO	High	IsMasterPr	AAEL01012	2:1617375	3	7	FALSO	2
5,09E+18	FALSO	High	IsMasterPr	AAEL01126	1:2153784	3	10	FALSO	2
-7,77E+18	FALSO	High	IsMasterPr	AAEL00541	1:2462364	3	6	FALSO	1
-8,37E+18	FALSO	High	IsMasterPr	AAEL01095	3:1676589	3	9	FALSO	2
2,22E+18	FALSO	High	IsMasterPr	AAEL00871	3:9574653	3	4	FALSO	3
-2,20E+18	FALSO	High	IsMasterPr	AAEL01870	3:3398240	3	3	FALSO	2
4,41E+18	FALSO	High	IsMasterPr	AAEL00662	2:4627140	3	13	FALSO	2
-5,45E+18	FALSO	High	IsMasterPr	AAEL01383	1:1793966	3	10	FALSO	1
-6,10E+17	FALSO	High	IsMasterPr	AAEL00959	2:3578181	3	2	FALSO	1
-8,99E+18	FALSO	High	IsMasterPr	AAEL02633	2:3735502	3	12	FALSO	2
2,88E+18	FALSO	High	IsMasterPr	AAEL00143	2:4601096	3	6	FALSO	2
-3,90E+17	FALSO	High	IsMasterPr	AAEL02467	2:2567596	3	12	FALSO	1
6,03E+18	FALSO	High	IsMasterPr	AAEL01977	2:2425954	3	4	FALSO	2
-6,91E+18	FALSO	High	IsMasterPr	AAEL01993	2:5972040	3	3	FALSO	1
2,04E+16	FALSO	High	IsMasterPr	AAEL01698	1:2099998	3	3	FALSO	2
6,88E+18	FALSO	High	IsMasterPr	AAEL02591	2:3895898	3	3	FALSO	1
-6,29E+18	FALSO	High	IsMasterPr	AAEL00172	2:4417686	3	3	FALSO	1
-5,57E+17	FALSO	High	IsMasterPr	AAEL02694	3:4231539	3	1	FALSO	2
7,28E+18	FALSO	High	IsMasterPr	AAEL01701	1:7986955	3	15	FALSO	3
-4,00E+18	FALSO	High	IsMasterPr	AAEL00906	2:2694582	3	5	FALSO	2
3,74E+18	FALSO	High	IsMasterPr	AAEL01416	2:1157942	3	29	FALSO	1
4,14E+18	FALSO	High	IsMasterPr	AAEL00849	2:1909927	3	10	FALSO	1
-3,92E+18	FALSO	High	IsMasterPr	AAEL01322	3:2799085	3	4	FALSO	2
4,55E+18	FALSO	High	IsMasterPr	AAEL00643	2:4394229	3	3	FALSO	3
-3,65E+18	FALSO	High	IsMasterPr	AAEL00582	1:2003852	3	4	FALSO	1
8,10E+18	FALSO	High	IsMasterPr	AAEL00051	3:1640497	3	6	FALSO	2
-2,84E+18	FALSO	High	IsMasterPr	AAEL02354	1:3022951	3	2	FALSO	2
-7,64E+18	FALSO	High	IsMasterPr	AAEL00613	3:6970560	3	8	FALSO	2
-7,94E+18	FALSO	High	IsMasterPr	AAEL00117	2:3072930	3	10	FALSO	2
7,78E+18	FALSO	High	IsMasterPr	AAEL00684	3:4115273	3	13	FALSO	2

-7,17E+18	FALSO	High	IsMasterPr	AAEL00722	2:42233660	3	8	FALSO	1
1,67E+18	FALSO	High	IsMasterPr	AAEL01107	2:2778225	3	3	FALSO	3
9,12E+18	FALSO	High	IsMasterPr	AAEL02743	2:1797419	3	8	FALSO	2
6,02E+18	FALSO	High	IsMasterPr	AAEL02667	2:2135185	3	3	FALSO	1
-3,23E+18	FALSO	High	IsMasterPr	AAEL02627	3:1327976	3	9	FALSO	2
2,63E+17	FALSO	High	IsMasterPr	AAEL00421	3:1162874	3	5	FALSO	3
4,39E+18	FALSO	High	IsMasterPr	AAEL00243	1:9894328	3	4	FALSO	2
8,84E+18	FALSO	High	IsMasterPr	AAEL00348	2:1536721	3	5	FALSO	3
-5,86E+18	FALSO	High	IsMasterPr	AAEL01045	3:3138071	3	15	FALSO	1
-8,09E+18	FALSO	High	IsMasterPr	AAEL02693	3:3096866	3	2	FALSO	2
6,27E+18	FALSO	High	IsMasterPr	AAEL02011	2:1156909	3	1	FALSO	1
7,30E+18	FALSO	High	IsMasterPr	AAEL00149	2:2624209	3	14	FALSO	2
3,74E+18	FALSO	High	IsMasterPr	AAEL02823	1:2852449	3	7	FALSO	1
-1,76E+18	FALSO	High	IsMasterPr	AAEL00782	2:2725507	3	4	FALSO	2
-3,52E+18	FALSO	High	IsMasterPr	AAEL01275	2:7568211	3	2	FALSO	2
-5,66E+18	FALSO	High	IsMasterPr	AAEL02325	1:9610320	3	20	FALSO	3
-3,26E+18	FALSO	High	IsMasterPr	AAEL00683	3:5710624	3	2	FALSO	1
-3,30E+18	FALSO	High	IsMasterPr	AAEL01037	2:1830232	3	2	FALSO	2
4,72E+18	FALSO	High	IsMasterPr	AAEL00326	1:2913314	3	11	FALSO	2
5,54E+18	FALSO	High	IsMasterPr	AAEL00430	3:5094421	3	16	FALSO	2
-5,41E+18	FALSO	High	IsMasterPr	AAEL01553	1:2187256	3	5	FALSO	2
-6,89E+18	FALSO	High	IsMasterPr	AAEL01745	2:4325922	3	9	FALSO	4
-7,92E+18	FALSO	High	IsMasterPr	AAEL01147	3:1427175	3	10	FALSO	2
8,32E+17	FALSO	High	IsMasterPr	AAEL00217	1:2512258	3	8	FALSO	1
-7,88E+18	FALSO	High	IsMasterPr	AAEL01963	2:2907047	3	6	FALSO	3
-5,31E+18	FALSO	High	IsMasterPr	AAEL02711	2:3890221	3	5	FALSO	2
3,26E+18	FALSO	High	IsMasterPr	AAEL00193	2:1955328	3	18	FALSO	3
-7,16E+18	FALSO	High	IsMasterPr	AAEL01201	2:1863771	3	6	FALSO	2
1,78E+18	FALSO	High	IsMasterPr	AAEL01810	2:2820835	3	1	FALSO	2
-4,57E+18	FALSO	High	IsMasterPr	AAEL01014	2:2647118	3	10	FALSO	1
-8,54E+18	FALSO	High	IsMasterPr	AAEL00636	1:1014879	3	13	FALSO	2
1,69E+18	FALSO	High	IsMasterPr	AAEL00331	3:3302910	3	5	FALSO	1
-4,46E+18	FALSO	High	IsMasterPr	AAEL01426	1:2857990	3	4	FALSO	2
2,95E+18	FALSO	High	IsMasterPr	AAEL02327	2:3203539	3	6	FALSO	3
-7,23E+18	FALSO	High	IsMasterPr	AAEL01472	1:6999868	3	7	FALSO	2
5,45E+18	FALSO	High	IsMasterPr	AAEL00344	3:3497761	3	14	FALSO	2
-6,33E+18	FALSO	High	IsMasterPr	AAEL00213	1:2535702	3	6	FALSO	1
5,53E+18	FALSO	High	IsMasterPr	AAEL02782	1:5747368	3	5	FALSO	2
3,27E+18	FALSO	High	IsMasterPr	AAEL01835	2:4076068	3	6	FALSO	2
2,95E+18	FALSO	High	IsMasterPr	AAEL00203	2:4239928	3	7	FALSO	2
-9,19E+18	FALSO	High	IsMasterPr	Q8GBH0	10 kDa cha	3	29	FALSO	2
5,54E+18	FALSO	High	IsMasterPr	AAEL00735	3:1231945	3	5	FALSO	2
-8,59E+18	FALSO	High	IsMasterPr	AAEL00278	1:3046248	3	4	FALSO	1
5,17E+18	FALSO	High	IsMasterPr	AAEL01822	3:2233691	3	4	FALSO	1
-5,63E+17	FALSO	High	IsMasterPr	AAEL00235	2:1752408	3	11	FALSO	1
-5,66E+18	FALSO	High	IsMasterPr	AAEL02801	1:1233942	3	3	FALSO	1
-3,57E+18	FALSO	High	IsMasterPr	AAEL00725	3:3902147	3	3	FALSO	1
4,32E+17	FALSO	High	IsMasterPr	AAEL00415	1:1577534	3	21	FALSO	1
-6,12E+18	FALSO	High	IsMasterPr	AAEL00211	3:1769435	3	12	FALSO	2
8,00E+18	FALSO	High	IsMasterPr	AAEL00438	1:2375318	3	4	FALSO	2

-5,59E+18	FALSO	High	IsMasterPr	AAEL0123S	1:28455338	3	5	FALSO	2
-5,17E+18	FALSO	High	IsMasterPr	AAEL00917	3:17602628	3	9	FALSO	3
1,98E+18	FALSO	High	IsMasterPr	AAEL01388	1:7190185(	3	2	FALSO	3
-6,88E+18	FALSO	High	IsMasterPr	AAEL00404	1:1357480	3	15	FALSO	2
-8,13E+18	FALSO	High	IsMasterPr	AAEL01062	1:4235938	3	9	FALSO	2
6,18E+18	FALSO	High	IsMasterPr	AAEL02095	1:5933215	3	5	FALSO	2
-3,34E+18	FALSO	High	IsMasterPr	AAEL01111	3:3816722(	3	4	FALSO	1
7,28E+17	FALSO	High	IsMasterPr	AAEL00423	1:1049680	3	10	FALSO	1
8,24E+18	FALSO	High	IsMasterPr	AAEL00921	2:1126085	3	10	FALSO	1
6,76E+18	FALSO	High	IsMasterPr	AAEL00007	3:1031012	3	20	FALSO	2
6,95E+18	FALSO	High	IsMasterPr	AAEL00491	2:7139623	3	17	FALSO	1
-2,76E+18	FALSO	High	IsMasterPr	AAEL02752	1:9083840(	3	3	FALSO	2
3,39E+18	FALSO	High	IsMasterPr	AAEL0008S	3:3344226	3	11	FALSO	1
-7,06E+16	FALSO	High	IsMasterPr	AAEL01972	3:3396107	3	8	FALSO	2
-5,28E+18	FALSO	High	IsMasterPr	AAEL02162	1:3078364(	3	2	FALSO	2
2,52E+18	FALSO	High	IsMasterPr	AAEL00233	2:1761640	3	22	FALSO	2
-5,82E+18	FALSO	High	IsMasterPr	AAEL0014C	2:4578562	3	6	FALSO	1
8,61E+18	FALSO	High	IsMasterPr	AAEL00941	3:3808414	3	8	FALSO	3
5,10E+18	FALSO	High	IsMasterPr	AAEL00286	1:2553666(	3	14	FALSO	1
6,52E+18	FALSO	High	IsMasterPr	AAEL01958	1:7005992	3	1	FALSO	2
-8,76E+18	FALSO	High	IsMasterPr	AAEL01032	3:4093146	3	9	FALSO	3
-6,87E+18	FALSO	High	IsMasterPr	AAEL00546	3:3827102	3	11	FALSO	2
1,86E+17	FALSO	High	IsMasterPr	AAEL00437	3:9382993	3	8	FALSO	2
6,76E+18	FALSO	High	IsMasterPr	AAEL01138	1:1200923	3	6	FALSO	1
6,41E+18	FALSO	High	IsMasterPr	AAEL01232	3:3166285	3	3	FALSO	3
-4,57E+18	FALSO	High	IsMasterPr	M9WSV3	Putative m	3	9	FALSO	1
6,70E+18	FALSO	High	IsMasterPr	AAEL00553	1:8532581	3	6	FALSO	1
9,08E+18	FALSO	High	IsMasterPr	AAEL00923	2:1707975	3	35	FALSO	2
-8,95E+18	FALSO	High	IsMasterPr	AAEL01221	1:1517195	3	3	FALSO	2
-5,62E+18	FALSO	High	IsMasterPr	AAEL01195	2:2181006(	3	24	FALSO	2
6,90E+18	FALSO	High	IsMasterPr	AAEL00231	3:3732878	3	1	FALSO	1
-1,53E+18	FALSO	High	IsMasterPr	AAEL01957	2:1303563	3	2	FALSO	2
-4,56E+17	FALSO	High	IsMasterPr	A0A3D1L5/	Uncharacte	3	6	FALSO	1
5,15E+18	FALSO	High	IsMasterPr	AAEL00703	1:1279252	3	9	FALSO	3
-9,80E+17	FALSO	High	IsMasterPr	AAEL00613	3:6879981	3	21	FALSO	2
8,01E+18	FALSO	High	IsMasterPr	AAEL00857	3:2224865	3	23	FALSO	2
-6,72E+18	FALSO	High	IsMasterPr	AAEL0140S	2:1211307	3	8	FALSO	1
6,67E+18	FALSO	High	IsMasterPr	AAEL00248	2:7790519	3	9	FALSO	2
6,72E+17	FALSO	High	IsMasterPr	AAEL0004C	3:3999332(	3	2	FALSO	3
1,77E+18	FALSO	High	IsMasterPr	AAEL00792	3:3120596(	3	7	FALSO	2
-3,53E+18	FALSO	High	IsMasterPr	AAEL00445	3:1096834	3	6	FALSO	2
-4,38E+18	FALSO	High	IsMasterPr	AAEL0025S	3:2568184(	3	7	FALSO	2
-4,16E+18	FALSO	High	IsMasterPr	AAEL01365	1:4298969(	3	5	FALSO	1
-3,02E+18	FALSO	High	IsMasterPr	AAEL01944	3:2988609	3	14	FALSO	2
4,72E+18	FALSO	High	IsMasterPr	AAEL0213C	3:3199584	3	17	FALSO	2
-4,70E+18	FALSO	High	IsMasterPr	AAEL02661	3:1213440	3	21	FALSO	2
4,60E+18	FALSO	High	IsMasterPr	AAEL00181	2:3753544	3	5	FALSO	2
6,28E+17	FALSO	High	IsMasterPr	AAEL02744	3:3142533	3	19	FALSO	1
7,38E+18	FALSO	High	IsMasterPr	AAEL02018	2:4029900	3	2	FALSO	2
-7,16E+18	FALSO	High	IsMasterPr	AAEL00837	3:4120610	3	1	FALSO	2

-3,91E+18	FALSO	High	IsMasterPr	AAEL00291	1:1087482	3	6	FALSO	1
-4,52E+18	FALSO	High	IsMasterPr	AAEL00788	3:3822822	3	4	FALSO	2
-6,22E+18	FALSO	High	IsMasterPr	AAEL01005	3:1122584	3	2	FALSO	1
-8,02E+17	FALSO	High	IsMasterPr	AAEL01095	3:1682322	3	6	FALSO	1
-2,80E+18	FALSO	High	IsMasterPr	AAEL02425	3:3771247	3	14	FALSO	1
-8,84E+18	FALSO	High	IsMasterPr	AAEL01125	1:2238712	3	7	FALSO	2
6,81E+18	FALSO	High	IsMasterPr	AAEL00634	3:3965781	3	6	FALSO	1
3,31E+18	FALSO	High	IsMasterPr	AAEL01207	3:1604466	3	19	FALSO	2
5,55E+18	FALSO	High	IsMasterPr	AAEL00738	2:2344474	3	4	FALSO	1
-2,67E+18	FALSO	High	IsMasterPr	AAEL01064	2:2791893	3	4	FALSO	1
-6,85E+18	FALSO	High	IsMasterPr	AAEL00929	2:1719622	3	10	FALSO	2
-8,97E+18	FALSO	High	IsMasterPr	AAEL00498	2:2815362	3	3	FALSO	1
-7,92E+17	FALSO	High	IsMasterPr	AAEL00241	1:1768159	3	15	FALSO	2
8,11E+18	FALSO	High	IsMasterPr	AAEL00322	2:3663869	3	10	FALSO	1
-7,66E+18	FALSO	High	IsMasterPr	AAEL02288	3:3166608	3	7	FALSO	1
7,83E+18	FALSO	High	IsMasterPr	AAEL01338	2:2650564	3	9	FALSO	1
7,18E+18	FALSO	High	IsMasterPr	AAEL00306	2:2125590	3	16	FALSO	2
-2,85E+18	FALSO	High	IsMasterPr	AAEL01389	1:7190058	3	25	FALSO	2
-2,46E+16	FALSO	High	IsMasterPr	AAEL00253	1:8934012	3	9	FALSO	2
3,59E+18	FALSO	High	IsMasterPr	AAEL00215	1:1022490	3	11	FALSO	2
2,77E+18	FALSO	High	IsMasterPr	AAEL00970	3:1555825	3	15	FALSO	3
7,84E+18	FALSO	High	IsMasterPr	AAEL01816	2:2148967	3	5	FALSO	1
8,26E+18	FALSO	High	IsMasterPr	AAEL00592	3:1386107	3	20	FALSO	2
1,06E+18	FALSO	High	IsMasterPr	A0A3D1L39	ATP syntha	3	16	FALSO	2
-2,37E+18	FALSO	High	IsMasterPr	AAEL00221	2:4305345	3	8	FALSO	1
8,05E+18	FALSO	High	IsMasterPr	AAEL00449	2:4532793	3	7	FALSO	2
-2,56E+18	FALSO	High	IsMasterPr	AAEL00143	1:2842087	3	11	FALSO	2
1,26E+18	FALSO	High	IsMasterPr	AAEL01952	2:1387190	3	4	FALSO	3
4,26E+18	FALSO	High	IsMasterPr	AAEL02154	1:6016757	3	3	FALSO	3
-7,87E+18	FALSO	High	IsMasterPr	AAEL01978	1:2016401	3	2	FALSO	1
-8,43E+18	FALSO	High	IsMasterPr	AAEL01305	3:3106234	3	4	FALSO	2
2,94E+18	FALSO	High	IsMasterPr	AAEL01102	1:7407643	3	11	FALSO	2
-6,04E+18	FALSO	High	IsMasterPr	AAEL02505	2:2764426	5	4	FALSO	2
6,86E+18	FALSO	High	IsMasterPr	AAEL00928	2:1746415	5	10	FALSO	1
7,46E+18	FALSO	High	IsMasterPr	AAEL02181	3:1496523	5	5	FALSO	1
-4,78E+18	FALSO	High	IsMasterPr	AAEL00130	2:4479222	5	5	FALSO	1
-4,06E+17	FALSO	High	IsMasterPr	AAEL00746	1:4638989	5	9	FALSO	2
-5,32E+18	FALSO	High	IsMasterPr	AAEL01268	3:2400449	5	4	FALSO	2
-5,28E+17	FALSO	High	IsMasterPr	AAEL00801	1:1644984	5	19	FALSO	2
6,54E+18	FALSO	High	IsMasterPr	AAEL02063	1:2084590	5	3	FALSO	3
5,66E+17	FALSO	High	IsMasterPr	AAEL00685	3:3703510	6	6	FALSO	1
-7,20E+18	FALSO	High	IsMasterPr	AAEL00922	2:1129124	6	13	FALSO	2
4,69E+18	FALSO	High	IsMasterPr	A0A059IWI	HU family D	6	24	FALSO	2
-9,00E+18	FALSO	High	IsMasterPr	AAEL00352	2:2562718	6	2	FALSO	1
8,15E+18	FALSO	High	IsMasterPr	AAEL01173	3:2910959	6	16	FALSO	2
4,51E+18	FALSO	High	IsMasterPr	AAEL02776	3:3381583	6	22	FALSO	2
-3,00E+18	FALSO	High	IsMasterPr	AAEL01216	2:2042214	6	6	FALSO	2
-9,06E+18	FALSO	High	IsMasterPr	AAEL00996	2:9310392	6	15	FALSO	1
8,22E+17	FALSO	High	IsMasterPr	AAEL00810	3:1458460	6	6	FALSO	2
-3,31E+18	FALSO	High	IsMasterPr	AAEL00341	3:3498764	6	16	FALSO	1

5,01E+18	FALSO	High	IsMasterPr	AAEL0117S	3:3607264(	6	14	FALSO	2
-8,63E+18	FALSO	High	IsMasterPr	A0A225X5\	Translation	6	4	FALSO	1
8,18E+18	FALSO	High	IsMasterPr	AAEL01471	2:1698220(	6	6	FALSO	2
-4,62E+18	FALSO	High	IsMasterPr	AAEL01543	3:1050803	6	4	FALSO	1
6,14E+18	FALSO	High	IsMasterPr	AAEL00168	2:8540217	6	12	FALSO	2
2,81E+18	FALSO	High	IsMasterPr	AAEL0017E	2:1652739	6	3	FALSO	3
4,96E+18	FALSO	High	IsMasterPr	AAEL00943	2:2471087	6	5	FALSO	1
-7,37E+18	FALSO	High	IsMasterPr	AAEL00841	1:1728837(	6	3	FALSO	1
6,17E+18	FALSO	High	IsMasterPr	AAEL00313	3:2706558	6	4	FALSO	2
-5,02E+18	FALSO	High	IsMasterPr	AAEL00054	3:3849385	6	6	FALSO	2
-8,54E+18	FALSO	High	IsMasterPr	AAEL0022E	2:3111215	6	9	FALSO	2
5,23E+18	FALSO	High	IsMasterPr	AAEL02063	2:4592629	6	1	FALSO	2
-8,76E+18	FALSO	High	IsMasterPr	Q4W4A0	60 kDa cha	6	12	FALSO	2
-8,69E+18	FALSO	High	IsMasterPr	AAEL00235	1:1197546	6	7	FALSO	2
-7,34E+18	FALSO	High	IsMasterPr	A0A5F1B8k	ANK_REP_I	6	1	FALSO	2
-6,27E+18	FALSO	High	IsMasterPr	AAEL01975	1:2248315	6	2	FALSO	2
-6,00E+18	FALSO	High	IsMasterPr	AAEL00885	2:2408081(	6	6	FALSO	2
7,90E+18	FALSO	High	IsMasterPr	AAEL0051S	2:1463368	6	4	FALSO	1
-7,71E+18	FALSO	High	IsMasterPr	AAEL00123	2:6088955	6	5	FALSO	1
8,69E+18	FALSO	High	IsMasterPr	AAEL0115E	3:1068731	6	6	FALSO	1
-5,95E+18	FALSO	High	IsMasterPr	AAEL0153S	2:3422300	6	6	FALSO	2
9,04E+17	FALSO	High	IsMasterPr	AAEL00154	2:2080424	6	7	FALSO	2
2,47E+18	FALSO	High	IsMasterPr	A0A140INC	60 kDa cha	6	13	FALSO	2
5,80E+17	FALSO	High	IsMasterPr	AAEL0080C	2:3653849	6	3	FALSO	1
6,75E+18	FALSO	High	IsMasterPr	AAEL01997	2:2051912(	6	1	FALSO	2
6,15E+18	FALSO	High	IsMasterPr	AAEL00648	3:1555281	6	6	FALSO	2
8,02E+18	FALSO	High	IsMasterPr	AAEL0236E	2:2537204	6	5	FALSO	2
-2,52E+18	FALSO	High	IsMasterPr	AAEL01814	2:4739265	6	3	FALSO	1
-2,96E+18	FALSO	High	IsMasterPr	AAEL00938	3:3808639(	6	6	FALSO	2
3,54E+18	FALSO	High	IsMasterPr	AAEL0089S	2:1784591	6	35	FALSO	2
5,56E+18	FALSO	High	IsMasterPr	AAEL01465	1:2615621	6	11	FALSO	1
-6,24E+18	FALSO	High	IsMasterPr	AAEL0145C	2:1496052	6	5	FALSO	2
-6,33E+18	FALSO	High	IsMasterPr	AAEL0037C	2:1978249	6	7	FALSO	1
6,85E+18	FALSO	High	IsMasterPr	AAEL0072S	3:1959291	6	5	FALSO	1
1,99E+18	FALSO	High	IsMasterPr	AAEL0040S	3:4022658(	6	3	FALSO	2
-6,55E+18	FALSO	High	IsMasterPr	AAEL00978	1:2760996	6	11	FALSO	2
-2,45E+18	FALSO	High	IsMasterPr	AAEL00281	NIGP01001	6	4	FALSO	1
-2,55E+18	FALSO	High	IsMasterPr	AAEL0130C	2:3491566	6	8	FALSO	2
4,23E+18	FALSO	High	IsMasterPr	AAEL0276S	3:7524553	6	1	FALSO	1
-1,03E+17	FALSO	High	IsMasterPr	Q6KEZ5	60 kDa cha	6	6	FALSO	2
7,06E+18	FALSO	High	IsMasterPr	AAEL0219S	2:4321206	6	12	FALSO	2
3,27E+18	FALSO	High	IsMasterPr	AAEL00132	2:4481869	6	20	FALSO	2
2,99E+18	FALSO	High	IsMasterPr	AAEL02178	2:2007041	6	9	FALSO	3
4,54E+18	FALSO	High	IsMasterPr	AAEL0132E	1:3234495(	6	13	FALSO	1
-8,78E+18	FALSO	High	IsMasterPr	AAEL01295	1:2393913	6	15	FALSO	1
2,64E+18	FALSO	High	IsMasterPr	AAEL01148	3:1425546	6	2	FALSO	1
5,99E+18	FALSO	High	IsMasterPr	AAEL00045	3:1838174	6	14	FALSO	2
-3,74E+18	FALSO	High	IsMasterPr	AAEL01261	1:1461494(	6	4	FALSO	2
-2,72E+17	FALSO	High	IsMasterPr	AAEL0093C	2:1745047	6	4	FALSO	1
-2,11E+16	FALSO	High	IsMasterPr	AAEL00863	2:2159581	6	2	FALSO	1

-1,61E+18	FALSO	High	IsMasterPr	AAEL0113C	3:1402430	6	15	FALSO	2
-4,19E+18	FALSO	High	IsMasterPr	AAEL0049€	3:3231658	6	13	FALSO	2
7,00E+18	FALSO	High	IsMasterPr	AAEL00691	3:1437500	6	5	FALSO	1
7,27E+18	FALSO	High	IsMasterPr	AAEL02293	1:1480743	6	22	FALSO	2
-6,50E+18	FALSO	High	IsMasterPr	AAEL00133	3:4769415	6	6	FALSO	1
-6,45E+18	FALSO	High	IsMasterPr	A0A2A4IH4	Probable c	7	7	FALSO	2
-6,31E+18	FALSO	High	IsMasterPr	AAEL00224	2:4291439	7	19	FALSO	1
-1,77E+18	FALSO	High	IsMasterPr	AAEL02497	2:1676601	7	4	FALSO	2
-1,43E+18	FALSO	High	IsMasterPr	AAEL01345	2:1757510	7	3	FALSO	1
6,20E+18	FALSO	High	IsMasterPr	AAEL00527	2:6931898	7	5	FALSO	2
5,47E+18	FALSO	High	IsMasterPr	AAEL00622	2:4907826	7	18	FALSO	3
-1,89E+18	FALSO	High	IsMasterPr	AAEL01022	2:4029572	7	5	FALSO	1
8,93E+18	FALSO	High	IsMasterPr	AAEL01802	3:2309121	7	5	FALSO	2
5,01E+18	FALSO	High	IsMasterPr	AAEL0131€	2:1804616	7	10	FALSO	2
-2,92E+18	FALSO	High	IsMasterPr	AAEL0127C	1:2030072	7	25	FALSO	2
-5,44E+18	FALSO	High	IsMasterPr	AAEL00738	1:2598410	7	6	FALSO	3
-5,49E+18	FALSO	High	IsMasterPr	AAEL0029C	1:1082743	7	5	FALSO	2
-4,77E+18	FALSO	High	IsMasterPr	AAEL01338	2:2652398	7	8	FALSO	2
-5,75E+18	FALSO	High	IsMasterPr	AAEL0071S	2:3182633	7	3	FALSO	1
-5,42E+17	FALSO	High	IsMasterPr	AAEL00507	1:2950003	7	4	FALSO	1
-5,11E+18	FALSO	High	IsMasterPr	AAEL01333	1:1928043	7	2	FALSO	1
5,54E+18	FALSO	High	IsMasterPr	AAEL0107€	2:3055247	7	9	FALSO	2
-1,07E+15	FALSO	High	IsMasterPr	AAEL00118	2:3075315	7	6	FALSO	1
-9,79E+17	FALSO	High	IsMasterPr	AAEL02565	1:6011140	7	2	FALSO	1
-8,09E+18	FALSO	High	IsMasterPr	AAEL00514	3:3459812	7	1	FALSO	1
4,53E+18	FALSO	High	IsMasterPr	AAEL00063	3:3951912	7	12	FALSO	2
1,19E+18	FALSO	High	IsMasterPr	AAEL00204	1:5827643	7	4	FALSO	2
-2,48E+18	FALSO	High	IsMasterPr	AAEL00528	2:2861630	7	1	FALSO	2
-2,47E+18	FALSO	High	IsMasterPr	AAEL00027	3:2664784	7	17	FALSO	2
-8,82E+18	FALSO	High	IsMasterPr	AAEL0101C	2:3607742	6	4	FALSO	1
-5,94E+18	FALSO	High	IsMasterPr	AAEL02352	3:3023145	6	3	FALSO	1
1,12E+18	FALSO	High	IsMasterPr	AAEL00011	1:1973326	6	9	FALSO	3
3,40E+18	FALSO	High	IsMasterPr	AAEL01981	2:1577706	6	6	FALSO	2
5,21E+18	FALSO	High	IsMasterPr	AAEL00495	3:3232667	6	6	FALSO	1
8,56E+18	FALSO	High	IsMasterPr	AAEL00498	2:2812220	6	4	FALSO	2
1,38E+18	FALSO	High	IsMasterPr	AAEL0045€	1:2206002	6	11	FALSO	1
9,04E+18	FALSO	High	IsMasterPr	AAEL01143	2:2253137	6	7	FALSO	1
-1,44E+18	FALSO	High	IsMasterPr	AAEL0076S	2:2510523	6	1	FALSO	1
-6,88E+18	FALSO	High	IsMasterPr	AAEL01228	2:1449633	6	20	FALSO	2
8,55E+18	FALSO	High	IsMasterPr	AAEL00728	3:1963978	6	8	FALSO	2
-6,84E+18	FALSO	High	IsMasterPr	AAEL01497	2:1783142	6	14	FALSO	2
-3,72E+18	FALSO	High	IsMasterPr	AAEL01215	2:2748847	6	3	FALSO	1
6,13E+17	FALSO	High	IsMasterPr	AAEL00467	3:2470031	6	13	FALSO	2
2,01E+18	FALSO	High	IsMasterPr	AAEL01982	3:3762218	6	3	FALSO	1
5,03E+18	FALSO	High	IsMasterPr	AAEL02213	2:1923641	6	3	FALSO	1
-7,83E+18	FALSO	High	IsMasterPr	AAEL00372	2:1974769	6	9	FALSO	2
7,98E+18	FALSO	High	IsMasterPr	AAEL01965	3:3319524	6	2	FALSO	1
-3,07E+18	FALSO	High	IsMasterPr	AAEL00483	2:1157255	6	19	FALSO	1
-3,45E+18	FALSO	High	IsMasterPr	AAEL02485	2:1498824	6	1	FALSO	1
1,51E+18	FALSO	High	IsMasterPr	AAEL00137	2:1181081	6	18	FALSO	2

6,59E+18	FALSO	High	IsMasterPr	AAEL00776	1:71881501	6	21	FALSO	2
-4,63E+18	FALSO	High	IsMasterPr	AAEL02439	3:26735431	6	2	FALSO	2
-6,35E+18	FALSO	High	IsMasterPr	AAEL00367	2:31721680	6	6	FALSO	1
6,09E+18	FALSO	High	IsMasterPr	AAEL01083	2:12804697	6	9	FALSO	2
8,91E+18	FALSO	High	IsMasterPr	AAEL01193	1:28632699	6	5	FALSO	1
8,17E+18	FALSO	High	IsMasterPr	AAEL00582	1:22551168	6	9	FALSO	1
2,43E+18	FALSO	High	IsMasterPr	AAEL00572	1:12356421	6	3	FALSO	2
4,91E+18	FALSO	High	IsMasterPr	AAEL01518	1:71478690	6	1	FALSO	1
3,32E+18	FALSO	High	IsMasterPr	AAEL00221	2:43063531	6	14	FALSO	2
9,18E+18	FALSO	High	IsMasterPr	AAEL00279	1:30518158	6	7	FALSO	3
4,86E+18	FALSO	High	IsMasterPr	AAEL01746	3:26328672	6	23	FALSO	1
1,01E+18	FALSO	High	IsMasterPr	AAEL00205	2:35674320	6	2	FALSO	1
3,63E+18	FALSO	High	IsMasterPr	AAEL00008	1:24277929	6	15	FALSO	1
1,45E+18	FALSO	High	IsMasterPr	AAEL00731	2:34293697	6	8	FALSO	1
-3,77E+17	FALSO	High	IsMasterPr	AAEL00305	2:21278257	6	3	FALSO	1
-6,68E+17	FALSO	High	IsMasterPr	A0A3T0GJ8	PmbA prot	6	6	FALSO	2
-5,63E+18	FALSO	High	IsMasterPr	AAEL00550	1:15047724	6	6	FALSO	1
2,23E+17	FALSO	High	IsMasterPr	AAEL01282	1:24252041	6	7	FALSO	2
-5,35E+17	FALSO	High	IsMasterPr	AAEL01061	3:29166857	6	12	FALSO	1
-3,74E+18	FALSO	High	IsMasterPr	AAEL01090	2:10853597	6	5	FALSO	2
-2,47E+17	FALSO	High	IsMasterPr	AAEL00028	2:75310399	6	11	FALSO	1
3,43E+18	FALSO	High	IsMasterPr	AAEL00112	3:16892321	6	2	FALSO	1
-1,48E+18	FALSO	High	IsMasterPr	AAEL00581	1:20157155	6	4	FALSO	1
-3,44E+17	FALSO	High	IsMasterPr	AAEL02285	NIGP0100C	6	5	FALSO	1
-1,85E+18	FALSO	High	IsMasterPr	AAEL02174	2:23319360	6	1	FALSO	1
-3,88E+18	FALSO	High	IsMasterPr	AAEL00358	1:69235014	6	4	FALSO	2
-6,69E+18	FALSO	High	IsMasterPr	AAEL01040	1:26837919	6	1	FALSO	1
1,99E+17	FALSO	High	IsMasterPr	A0A218KQ	Uncharacte	6	20	FALSO	1
-4,10E+18	FALSO	High	IsMasterPr	AAEL00602	3:33451101	6	3	FALSO	2
-7,37E+18	FALSO	High	IsMasterPr	AAEL00613	3:69657872	6	5	FALSO	1
1,52E+18	FALSO	High	IsMasterPr	AAEL01361	3:19511290	6	2	FALSO	1
-5,37E+18	FALSO	High	IsMasterPr	AAEL02114	2:19306821	6	6	FALSO	1
3,55E+18	FALSO	High	IsMasterPr	AAEL02565	1:60200081	6	12	FALSO	2
-4,81E+18	FALSO	High	IsMasterPr	AAEL01948	2:20428162	6	4	FALSO	2
6,47E+17	FALSO	High	IsMasterPr	AAEL00377	1:24809960	6	9	FALSO	2
-1,46E+18	FALSO	High	IsMasterPr	AAEL01208	3:16036535	6	7	FALSO	1
-2,05E+18	FALSO	High	IsMasterPr	AAEL00976	1:41535921	6	10	FALSO	3
3,77E+18	FALSO	High	IsMasterPr	AAEL00117	2:30725147	7	9	FALSO	1
-8,58E+18	FALSO	High	IsMasterPr	AAEL00150	3:26094441	7	4	FALSO	2
-5,21E+18	FALSO	High	IsMasterPr	AAEL02153	2:23173139	7	3	FALSO	1
8,55E+18	FALSO	High	IsMasterPr	AAEL00314	3:27089554	7	4	FALSO	2
5,68E+18	FALSO	High	IsMasterPr	AAEL01429	2:17184834	7	4	FALSO	1
-3,59E+18	FALSO	High	IsMasterPr	AAEL00704	3:29896091	7	0	FALSO	1
4,18E+18	FALSO	High	IsMasterPr	AAEL00645	2:43925468	7	12	FALSO	1
3,35E+18	FALSO	High	IsMasterPr	AAEL01225	3:53380387	7	10	FALSO	2
-3,40E+18	FALSO	High	IsMasterPr	AAEL01974	1:12328281	7	2	FALSO	1
7,02E+18	FALSO	High	IsMasterPr	AAEL00042	3:18525618	7	3	FALSO	1
3,89E+18	FALSO	High	IsMasterPr	AAEL00038	3:97604445	7	2	FALSO	1
7,33E+17	FALSO	High	IsMasterPr	AAEL00455	1:14444337	7	3	FALSO	2
1,68E+18	FALSO	High	IsMasterPr	AAEL02175	1:30326128	7	6	FALSO	2

-4,25E+18	FALSO	High	IsMasterPr	AAEL02223	1:2164891	7	4	FALSO	1
7,47E+18	FALSO	High	IsMasterPr	AAEL01078	2:3052422	7	3	FALSO	1
2,45E+18	FALSO	High	IsMasterPr	AAEL00118	2:1489239	7	4	FALSO	1
-9,20E+18	FALSO	High	IsMasterPr	AAEL01227	2:1449983	7	16	FALSO	2
-6,82E+18	FALSO	High	IsMasterPr	AAEL02302	1:2883230	7	4	FALSO	2
3,78E+18	FALSO	High	IsMasterPr	AAEL00545	2:2957876	7	6	FALSO	2
-9,02E+18	FALSO	High	IsMasterPr	A0A5R9ME	Phage tail t	7	9	FALSO	2
2,86E+18	FALSO	High	IsMasterPr	AAEL0036C	1:6924560	7	2	FALSO	1
3,27E+18	FALSO	High	IsMasterPr	AAEL01729	2:2488928	7	16	FALSO	1
-4,91E+17	FALSO	High	IsMasterPr	AAEL00525	1:3217838	7	3	FALSO	1
-6,02E+18	FALSO	High	IsMasterPr	AAEL01416	3:3022719	7	4	FALSO	1
1,36E+18	FALSO	High	IsMasterPr	AAEL00498	2:2813842	7	3	FALSO	1
9,69E+17	FALSO	High	IsMasterPr	AAEL00007	1:2426535	7	2	FALSO	2
-7,17E+18	FALSO	High	IsMasterPr	AAEL01432	2:1201272	7	4	FALSO	1
4,30E+18	FALSO	High	IsMasterPr	AAEL00866	2:2387407	7	7	FALSO	2
-5,34E+18	FALSO	High	IsMasterPr	AAEL00983	2:1917777	7	5	FALSO	1
4,16E+17	FALSO	High	IsMasterPr	AAEL00782	1:1491401	7	12	FALSO	1
6,94E+18	FALSO	High	IsMasterPr	AAEL00951	1:1408675	7	1	FALSO	1
6,94E+18	FALSO	High	IsMasterPr	AAEL00613	3:6874409	7	9	FALSO	1
5,96E+18	FALSO	High	IsMasterPr	AAEL0039C	1:2505456	7	3	FALSO	1
-7,72E+17	FALSO	High	IsMasterPr	AAEL00852	3:2974099	7	3	FALSO	2
-1,09E+18	FALSO	High	IsMasterPr	AAEL00171	2:4418604	7	5	FALSO	2
-7,17E+18	FALSO	High	IsMasterPr	AAEL0198C	3:3107478	7	0	FALSO	1
-3,10E+18	FALSO	High	IsMasterPr	AAEL01164	3:2816192	7	2	FALSO	1
2,12E+18	FALSO	High	IsMasterPr	AAEL0279C	3:3613491	7	5	FALSO	2
-6,52E+18	FALSO	High	IsMasterPr	AAEL00767	3:4072958	7	7	FALSO	1
-2,59E+17	FALSO	High	IsMasterPr	AAEL0211C	1:2625422	7	2	FALSO	1
3,47E+18	FALSO	High	IsMasterPr	AAEL02903	2:1003638	7	23	FALSO	1
-2,20E+18	FALSO	High	IsMasterPr	AAEL01123	3:7102298	7	2	FALSO	1
-7,83E+17	FALSO	High	IsMasterPr	AAEL01207	3:1602265	7	4	FALSO	2
6,48E+18	FALSO	High	IsMasterPr	AAEL00926	2:2681588	7	2	FALSO	2
1,04E+18	FALSO	High	IsMasterPr	AAEL01109	2:2320136	7	3	FALSO	1
-6,85E+18	FALSO	High	IsMasterPr	AAEL00535	2:3703435	7	12	FALSO	2
3,45E+18	FALSO	High	IsMasterPr	AAEL01068	2:4175553	7	2	FALSO	1
-5,17E+18	FALSO	High	IsMasterPr	AAEL00235	2:1760958	7	5	FALSO	2
1,54E+18	FALSO	High	IsMasterPr	AAEL00138	2:1181151	7	28	FALSO	2
-8,98E+18	FALSO	High	IsMasterPr	AAEL00837	3:4140651	7	2	FALSO	1
2,51E+18	FALSO	High	IsMasterPr	AAEL01173	3:2906569	7	7	FALSO	2
-5,33E+18	FALSO	High	IsMasterPr	AAEL00823	1:4402530	7	4	FALSO	1
-5,43E+18	FALSO	High	IsMasterPr	AAEL0009C	3:3344797	7	3	FALSO	1
-7,83E+18	FALSO	High	IsMasterPr	AAEL01744	3:2923104	7	4	FALSO	1
-2,22E+17	FALSO	High	IsMasterPr	AAEL01285	1:2222208	7	9	FALSO	1
-8,42E+18	FALSO	High	IsMasterPr	AAEL01112	3:3820712	7	8	FALSO	1
-1,09E+18	FALSO	High	IsMasterPr	AAEL01401	3:1878003	7	9	FALSO	2
-2,06E+18	FALSO	High	IsMasterPr	AAEL00845	1:5341893	7	16	FALSO	1
-3,86E+18	FALSO	High	IsMasterPr	AAEL01499	2:1720739	7	7	FALSO	2
7,43E+18	FALSO	High	IsMasterPr	AAEL00485	2:1156216	7	3	FALSO	1
-2,20E+18	FALSO	High	IsMasterPr	AAEL02787	3:2913770	7	1	FALSO	1
-7,35E+18	FALSO	High	IsMasterPr	AAEL0259C	1:3609070	7	3	FALSO	1
6,97E+18	FALSO	High	IsMasterPr	AAEL02056	2:1913126	7	9	FALSO	1

-6,27E+18	FALSO	High	IsMasterPr	A0A369RLT	Fumarate h	7	7	FALSO	1
2,89E+18	FALSO	High	IsMasterPr	AAEL02591	3:2183601	7	2	FALSO	1
-7,72E+18	FALSO	High	IsMasterPr	AAEL00433	3:3666964	7	12	FALSO	1
8,08E+18	FALSO	High	IsMasterPr	AAEL0078C	3:1518352	7	1	FALSO	1
5,47E+18	FALSO	High	IsMasterPr	AAEL00193	2:1944280	7	5	FALSO	1
-1,51E+18	FALSO	High	IsMasterPr	AAEL00724	2:4227126	7	1	FALSO	2
8,32E+18	FALSO	High	IsMasterPr	AAEL01105	2:6335711	7	5	FALSO	2
5,56E+18	FALSO	High	IsMasterPr	AAEL0015C	2:2149843	7	6	FALSO	1
-8,95E+18	FALSO	High	IsMasterPr	AAEL00793	3:2955620	7	10	FALSO	1
3,27E+18	FALSO	High	IsMasterPr	AAEL00877	2:1988892	7	1	FALSO	1
-8,86E+18	FALSO	High	IsMasterPr	AAEL0017S	2:3764335	7	18	FALSO	2
5,62E+18	FALSO	High	IsMasterPr	AAEL00903	3:1932977	7	3	FALSO	2
-8,96E+18	FALSO	High	IsMasterPr	AAEL00428	2:2934584	7	4	FALSO	1
1,60E+18	FALSO	High	IsMasterPr	AAEL00045	3:1853168	7	18	FALSO	1
2,70E+18	FALSO	High	IsMasterPr	AAEL0275C	3:2427924	7	3	FALSO	2
-5,52E+18	FALSO	High	IsMasterPr	AAEL01347	2:1210886	7	4	FALSO	2
-7,18E+18	FALSO	High	IsMasterPr	AAEL00162	1:1388096	7	4	FALSO	1
1,86E+18	FALSO	High	IsMasterPr	AAEL0073E	2:2334184	7	32	FALSO	1
-6,15E+18	FALSO	High	IsMasterPr	AAEL00754	2:3272644	7	7	FALSO	2
4,89E+18	FALSO	High	IsMasterPr	AAEL00815	3:9704029	7	6	FALSO	1
-3,16E+17	FALSO	High	IsMasterPr	AAEL0025S	3:2571213	7	7	FALSO	1
5,42E+18	FALSO	High	IsMasterPr	AAEL00937	3:2974829	7	3	FALSO	2
-6,69E+18	FALSO	High	IsMasterPr	AAEL00121	2:4134413	7	3	FALSO	1
-3,60E+17	FALSO	High	IsMasterPr	AAEL01957	3:1300912	7	1	FALSO	1
6,95E+18	FALSO	High	IsMasterPr	AAEL00971	3:1559399	7	4	FALSO	1
-4,50E+18	FALSO	High	IsMasterPr	AAEL01083	2:1281160	7	13	FALSO	2
-4,28E+18	FALSO	High	IsMasterPr	AAEL0155E	2:4423570	7	9	FALSO	1
6,49E+18	FALSO	High	IsMasterPr	AAEL00801	1:1634406	7	2	FALSO	1
-1,65E+18	FALSO	High	IsMasterPr	AAEL0107C	3:3057253	7	7	FALSO	2
3,65E+17	FALSO	High	IsMasterPr	AAEL02263	1:6992883	7	18	FALSO	2
-6,19E+18	FALSO	High	IsMasterPr	AAEL01194	2:2184569	7	5	FALSO	2
-4,91E+18	FALSO	High	IsMasterPr	AAEL00001	1:2428204	7	6	FALSO	1
-8,75E+18	FALSO	High	IsMasterPr	AAEL00782	2:2718299	7	6	FALSO	1
8,08E+18	FALSO	High	IsMasterPr	AAEL00742	2:1349933	7	3	FALSO	1
2,67E+18	FALSO	High	IsMasterPr	AAEL01422	3:1972756	7	4	FALSO	2
-5,96E+18	FALSO	High	IsMasterPr	AAEL00813	3:7911503	7	2	FALSO	1
-5,74E+18	FALSO	High	IsMasterPr	AAEL00521	2:353721:3	7	5	FALSO	1
3,66E+17	FALSO	High	IsMasterPr	AAEL0146E	2:3475971	7	7	FALSO	2
-3,63E+18	FALSO	High	IsMasterPr	AAEL01181	3:1132918	7	2	FALSO	1
2,55E+18	FALSO	High	IsMasterPr	AAEL01527	2:3458559	7	17	FALSO	2
5,32E+18	FALSO	High	IsMasterPr	AAEL0033C	3:3403743	7	5	FALSO	1
-7,44E+18	FALSO	High	IsMasterPr	AAEL00811	3:1490484	7	10	FALSO	1
6,08E+18	FALSO	High	IsMasterPr	AAEL01341	2:1592449	7	18	FALSO	2
7,63E+18	FALSO	High	IsMasterPr	AAEL0196S	2:3407448	7	5	FALSO	2
8,29E+18	FALSO	High	IsMasterPr	AAEL00102	3:5552273	7	7	FALSO	2
8,47E+18	FALSO	High	IsMasterPr	AAEL01406	2:4059580	8	8	FALSO	1
6,52E+18	FALSO	High	IsMasterPr	AAEL01984	3:3066135	8	1	FALSO	1
-2,83E+18	FALSO	High	IsMasterPr	AAEL01994	3:4530340	8	2	FALSO	1
3,86E+18	FALSO	High	IsMasterPr	AAEL0275E	2:3630715	8	4	FALSO	2
8,29E+18	FALSO	High	IsMasterPr	AAEL01238	3:9630366	8	3	FALSO	1

-7,08E+17	FALSO	High	IsMasterPr	AAEL0219٤	2:1523523	8	2	FALSO	2
-7,85E+18	FALSO	High	IsMasterPr	AAEL00327	1:2909795	8	3	FALSO	2
2,29E+18	FALSO	High	IsMasterPr	AAEL01237	2:1275100	8	4	FALSO	1
-7,28E+18	FALSO	High	IsMasterPr	AAEL01951	3:3739958	8	1	FALSO	1
-5,64E+18	FALSO	High	IsMasterPr	B6Y9Q4	Putative pł	8	15	FALSO	1
2,93E+18	FALSO	High	IsMasterPr	A0A059IW!	Uncharacte	8	6	FALSO	1
-5,85E+18	FALSO	High	IsMasterPr	AAEL00494	3:3231432	8	2	FALSO	2
1,05E+18	FALSO	High	IsMasterPr	AAEL0015€	2:2285575!	8	4	FALSO	1
-2,15E+18	FALSO	High	IsMasterPr	A0A218KPI	DNA ligase	8	4	FALSO	1
-8,05E+18	FALSO	High	IsMasterPr	AAEL00291	NIGP0100C	7	7	FALSO	1
3,86E+18	FALSO	High	IsMasterPr	AAEL00547	3:3829964	7	5	FALSO	1
-5,87E+18	FALSO	High	IsMasterPr	AAEL01403	1:1429868	7	13	FALSO	1
-2,22E+18	FALSO	High	IsMasterPr	AAEL0116€	3:2190105	7	9	FALSO	1
4,36E+18	FALSO	High	IsMasterPr	AAEL01943	2:3345947	7	3	FALSO	2
5,41E+18	FALSO	High	IsMasterPr	AAEL0072C	1:2673140	7	8	FALSO	1
1,55E+18	FALSO	High	IsMasterPr	AAEL00495	3:3231305	7	2	FALSO	1
-3,69E+18	FALSO	High	IsMasterPr	AAEL00506	1:2980890	7	8	FALSO	2
7,44E+18	FALSO	High	IsMasterPr	AAEL01253	2:8266277	7	4	FALSO	1
4,40E+18	FALSO	High	IsMasterPr	AAEL00958	2:3570160	7	5	FALSO	1
-8,92E+18	FALSO	High	IsMasterPr	AAEL00065	3:3935717	7	7	FALSO	1
2,78E+17	FALSO	High	IsMasterPr	AAEL0037€	1:2479473	7	3	FALSO	1
3,04E+18	FALSO	High	IsMasterPr	AAEL0099C	1:2067273	7	7	FALSO	2
1,09E+18	FALSO	High	IsMasterPr	AAEL00777	3:1335332	7	7	FALSO	2
2,93E+18	FALSO	High	IsMasterPr	AAEL00937	3:7553030	7	2	FALSO	1
3,89E+18	FALSO	High	IsMasterPr	AAEL0206!	3:2146813	7	2	FALSO	1
-6,89E+17	FALSO	High	IsMasterPr	AAEL01754	3:2243802	7	7	FALSO	1
-3,31E+18	FALSO	High	IsMasterPr	AAEL01043	3:6764465	7	12	FALSO	2
8,99E+18	FALSO	High	IsMasterPr	AAEL00255	1:2264942	7	1	FALSO	1
4,67E+18	FALSO	High	IsMasterPr	AAEL00617	3:1413087	8	3	FALSO	1
-7,09E+18	FALSO	High	IsMasterPr	AAEL01138	1:1239560	8	2	FALSO	1
4,40E+18	FALSO	High	IsMasterPr	AAEL01957	3:5703440	8	2	FALSO	1
-5,35E+18	FALSO	High	IsMasterPr	AAEL01313	3:3711667	8	2	FALSO	2
3,38E+18	FALSO	High	IsMasterPr	AAEL00664	3:1481537	8	15	FALSO	1
3,28E+18	FALSO	High	IsMasterPr	AAEL01325	2:3993471	8	1	FALSO	1
-5,56E+17	FALSO	High	IsMasterPr	AAEL00507	1:2952895	9	1	FALSO	1
3,98E+18	FALSO	High	IsMasterPr	AAEL00817	3:9455394	9	24	FALSO	1
4,92E+18	FALSO	High	IsMasterPr	AAEL00197	2:4253238	9	4	FALSO	1
4,50E+18	FALSO	High	IsMasterPr	AAEL0034C	3:3493497	9	15	FALSO	2
3,11E+18	FALSO	High	IsMasterPr	AAEL02683	2:6487293	9	4	FALSO	1
-7,33E+18	FALSO	High	IsMasterPr	A0A518HF\	Outer surfa	9	27	FALSO	2
8,36E+18	FALSO	High	IsMasterPr	AAEL00342	3:3501620	9	14	FALSO	2
-2,04E+18	FALSO	High	IsMasterPr	AAEL01277	1:2906234	9	4	FALSO	2
-3,05E+18	FALSO	High	IsMasterPr	AAEL00864	2:2152688	9	7	FALSO	2
7,08E+18	FALSO	High	IsMasterPr	AAEL00835	2:4203564	10	6	FALSO	2
4,94E+18	FALSO	High	IsMasterPr	AAEL00995	2:3796849	10	5	FALSO	1
-1,12E+17	FALSO	High	IsMasterPr	AAEL01171	1:2382611	10	19	FALSO	1
1,87E+17	FALSO	High	IsMasterPr	AAEL00272	2:1446346	10	15	FALSO	1
-7,22E+18	FALSO	High	IsMasterPr	AAEL0195!	1:2907845	10	2	FALSO	1
5,91E+18	FALSO	High	IsMasterPr	AAEL00272	2:1332653	10	2	FALSO	2
-2,44E+18	FALSO	High	IsMasterPr	Q9JMN9	Gp2 protei	10	30	FALSO	1

5,85E+18	FALSO	High	IsMasterPr	AAEL00027	2:7542148	10	10	FALSO	2
-5,82E+17	FALSO	High	IsMasterPr	AAEL01139	3:2016533	10	2	FALSO	1
-4,07E+18	FALSO	High	IsMasterPr	AAEL02293	3:1046469	10	1	FALSO	1
-7,21E+18	FALSO	High	IsMasterPr	A0A060PYI	Protein Hfl	10	7	FALSO	1
-8,26E+18	FALSO	High	IsMasterPr	AAEL02584	3:1482871	10	4	FALSO	2
-8,59E+17	FALSO	High	IsMasterPr	AAEL00241	1:1767546	10	2	FALSO	1
-2,93E+18	FALSO	High	IsMasterPr	Q5GRN3	Probable tr	10	18	FALSO	2
4,42E+18	FALSO	High	IsMasterPr	AAEL0122C	1:1606222	10	4	FALSO	1
-5,63E+18	FALSO	High	IsMasterPr	AAEL0072C	2:7722742	10	2	FALSO	1
8,13E+18	FALSO	High	IsMasterPr	AAEL01399	3:2306217	10	4	FALSO	2
3,94E+18	FALSO	High	IsMasterPr	AAEL01396	3:5781003	10	11	FALSO	1
5,11E+18	FALSO	High	IsMasterPr	AAEL0034C	3:3498595	10	5	FALSO	1
1,39E+18	FALSO	High	IsMasterPr	AAEL01464	2:1575217	10	5	FALSO	1
8,44E+18	FALSO	High	IsMasterPr	AAEL01818	1:2070828	10	4	FALSO	1
-6,82E+18	FALSO	High	IsMasterPr	AAEL02007	1:6818626	10	2	FALSO	1
-9,19E+18	FALSO	High	IsMasterPr	AAEL01283	2:1055334	10	5	FALSO	1
6,03E+18	FALSO	High	IsMasterPr	AAEL01451	1:2721453	10	4	FALSO	1
-3,53E+18	FALSO	High	IsMasterPr	AAEL01124	1:2237332	10	4	FALSO	1
6,69E+18	FALSO	High	IsMasterPr	AAEL00885	2:2413992	10	8	FALSO	1
-8,79E+18	FALSO	High	IsMasterPr	Q4ECQ2	Phenylalan	10	3	FALSO	1
-4,92E+17	FALSO	High	IsMasterPr	AAEL00662	2:4640101	10	3	FALSO	1
7,93E+18	FALSO	High	IsMasterPr	AAEL00926	2:2684446	10	12	FALSO	2
-1,60E+17	FALSO	High	IsMasterPr	AAEL01725	3:2641355	10	3	FALSO	2
-4,78E+18	FALSO	High	IsMasterPr	A0A060Q0	Uncharacte	10	10	FALSO	1
7,76E+18	FALSO	Medium	IsMasterPr	AAEL00057	2:1476223	10	4	FALSO	1
-5,48E+18	FALSO	Medium	IsMasterPr	AAEL00254	1:2285562	10	4	FALSO	1
-3,20E+18	FALSO	Medium	IsMasterPr	AAEL02339	2:2318953	10	6	FALSO	1
1,75E+18	FALSO	Medium	IsMasterPr	AAEL00586	NIGP01002	10	5	FALSO	1
5,94E+18	FALSO	Medium	IsMasterPr	AAEL00206	2:1199880	10	5	FALSO	1
5,62E+18	FALSO	Medium	IsMasterPr	AAEL02903	1:8454197	10	15	FALSO	1
-1,27E+18	FALSO	Medium	IsMasterPr	AAEL01227	2:1450848	10	7	FALSO	2
-2,11E+18	FALSO	Medium	IsMasterPr	AAEL0026C	3:2566680	10	2	FALSO	1
7,82E+18	FALSO	Medium	IsMasterPr	AAEL01954	3:3724931	10	27	FALSO	2
4,50E+18	FALSO	Medium	IsMasterPr	AAEL00525	2:6916497	10	8	FALSO	2
1,65E+18	FALSO	Medium	IsMasterPr	AAEL00542	1:2477750	10	7	FALSO	1
-8,07E+18	FALSO	Medium	IsMasterPr	A0A059IWI	Phosphatid	11	10	FALSO	1
-6,94E+18	FALSO	Medium	IsMasterPr	A0A1E7QJC	Serine hydi	11	6	FALSO	1
-1,58E+18	FALSO	Medium	IsMasterPr	AAEL00836	3:4137588	11	14	FALSO	1
3,08E+18	FALSO	Medium	IsMasterPr	AAEL0183C	1:2481166	11	1	FALSO	1
-2,16E+18	FALSO	Medium	IsMasterPr	AAEL0108C	3:1567466	11	3	FALSO	1
-5,04E+18	FALSO	Medium	IsMasterPr	AAEL01948	2:3549676	11	4	FALSO	2
7,47E+18	FALSO	Medium	IsMasterPr	AAEL00322	1:2916576	11	5	FALSO	2
-4,95E+18	FALSO	Medium	IsMasterPr	AAEL0182C	1:7402363	11	3	FALSO	1
8,70E+18	FALSO	Medium	IsMasterPr	AAEL02407	1:2133406	11	15	FALSO	1
5,63E+18	FALSO	Medium	IsMasterPr	AAEL00861	1:2413402	11	3	FALSO	1
8,43E+18	FALSO	Medium	IsMasterPr	AAEL00601	3:3152047	11	5	FALSO	2
4,51E+18	FALSO	Medium	IsMasterPr	AAEL00287	1:2559186	11	3	FALSO	1
-8,78E+18	FALSO	Medium	IsMasterPr	AAEL01946	1:7208132	11	7	FALSO	2
-8,96E+18	FALSO	Medium	IsMasterPr	AAEL00229	3:3741214	11	19	FALSO	1
-4,52E+18	FALSO	Medium	IsMasterPr	AAEL00458	2:3510079	11	10	FALSO	1

5,10E+18	FALSO	Medium	IsMasterPr	AAEL00884	2:2418199	11	4	FALSO	2
6,93E+16	FALSO	Medium	IsMasterPr	AAEL02107	3:3157502	11	6	FALSO	2
5,14E+18	FALSO	Medium	IsMasterPr	AAEL00174	2:3791319	11	1	FALSO	1
-2,89E+18	FALSO	Medium	IsMasterPr	AAEL01287	3:2760001	11	4	FALSO	1
-5,38E+18	FALSO	Medium	IsMasterPr	AAEL02627	1:1011982	11	3	FALSO	1
4,67E+17	FALSO	Medium	IsMasterPr	AAEL02472	1:2035781	11	1	FALSO	1
-5,76E+18	FALSO	Medium	IsMasterPr	AAEL01245	1:1234265	11	3	FALSO	1
-7,75E+17	FALSO	Medium	IsMasterPr	AAEL01376	2:3449502	11	4	FALSO	2
-2,59E+18	FALSO	Medium	IsMasterPr	AAEL00328	1:2920184	11	1	FALSO	2
-3,18E+18	FALSO	Medium	IsMasterPr	AAEL01115	3:1935532	11	9	FALSO	1
-8,51E+18	FALSO	Medium	IsMasterPr	A0A5R9MF	Uncharacte	11	8	FALSO	2
5,47E+18	FALSO	Medium	IsMasterPr	AAEL00093	3:3771832	11	8	FALSO	1
8,76E+18	FALSO	Medium	IsMasterPr	AAEL00809	3:1485053	11	2	FALSO	1
4,87E+18	FALSO	Medium	IsMasterPr	AAEL00392	3:2541834	11	11	FALSO	1
-3,62E+18	FALSO	Medium	IsMasterPr	AAEL00501	2:4444596	11	5	FALSO	1
6,35E+18	FALSO	Medium	IsMasterPr	AAEL02155	1:7917387	11	1	FALSO	2
-9,00E+18	FALSO	Medium	IsMasterPr	AAEL01437	3:4465360	11	10	FALSO	1
-4,89E+18	FALSO	Medium	IsMasterPr	AAEL00003	3:1049825	11	8	FALSO	2
2,18E+18	FALSO	Medium	IsMasterPr	AAEL00007	3:1049875	11	3	FALSO	1
3,76E+18	FALSO	Medium	IsMasterPr	AAEL00620	3:1496221	11	3	FALSO	2
8,41E+18	FALSO	Medium	IsMasterPr	AAEL00033	2:1243828	11	11	FALSO	1
-6,25E+17	FALSO	Medium	IsMasterPr	AAEL00144	1:1117505	11	7	FALSO	2
7,29E+18	FALSO	Medium	IsMasterPr	AAEL01317	2:3890512	11	2	FALSO	1
-3,26E+18	FALSO	Medium	IsMasterPr	AAEL00317	1:2757903	11	5	FALSO	2
6,51E+18	FALSO	Medium	IsMasterPr	AAEL00617	3:1404866	11	8	FALSO	2
1,80E+18	FALSO	Medium	IsMasterPr	AAEL00783	1:1032346	11	17	FALSO	2
-8,95E+18	FALSO	Medium	IsMasterPr	AAEL00924	1:3543953	11	2	FALSO	2
1,02E+18	FALSO	Medium	IsMasterPr	AAEL01489	1:2714033	11	1	FALSO	1
3,03E+18	FALSO	Medium	IsMasterPr	AAEL00794	2:3521803	11	16	FALSO	1
-5,31E+18	FALSO	Medium	IsMasterPr	AAEL00346	3:2213796	11	8	FALSO	1
6,45E+18	FALSO	Medium	IsMasterPr	AAEL00546	3:3824910	11	1	FALSO	1
5,90E+18	FALSO	Medium	IsMasterPr	AAEL02362	NIGP0100C	11	3	FALSO	1
-7,10E+17	FALSO	Medium	IsMasterPr	AAEL00937	3:8106185	11	6	FALSO	1
-8,62E+18	FALSO	Medium	IsMasterPr	AAEL02751	1:4709213	11	6	FALSO	1
4,61E+18	FALSO	Medium	IsMasterPr	AAEL01946	2:1762184	11	3	FALSO	2
-1,89E+17	FALSO	Medium	IsMasterPr	AAEL01515	1:2054936	11	6	FALSO	2
-7,52E+18	FALSO	Medium	IsMasterPr	AAEL00152	3:2608024	11	10	FALSO	1
2,68E+18	FALSO	Medium	IsMasterPr	AAEL01211	2:4059280	11	11	FALSO	1
3,57E+18	FALSO	Medium	IsMasterPr	AAEL01138	1:1259220	11	2	FALSO	2
-4,11E+18	FALSO	Medium	IsMasterPr	AAEL00084	3:3637962	11	14	FALSO	1
3,68E+18	FALSO	Medium	IsMasterPr	AAEL00030	2:3678704	11	7	FALSO	2
3,16E+18	FALSO	Medium	IsMasterPr	AAEL00221	1:3752727	11	22	FALSO	1
-3,73E+18	FALSO	Medium	IsMasterPr	AAEL00422	1:1046174	11	4	FALSO	1
1,48E+18	FALSO	Medium	IsMasterPr	AAEL02442	3:3657854	11	2	FALSO	1
5,23E+18	FALSO	Medium	IsMasterPr	AAEL01082	3:5195611	11	5	FALSO	2
7,12E+18	FALSO	Medium	IsMasterPr	A0A0B4U8	GroEL (Frag	11	20	FALSO	1
2,48E+18	FALSO	Medium	IsMasterPr	AAEL00594	1:2574475	11	7	FALSO	1
4,19E+18	FALSO	Medium	IsMasterPr	AAEL02374	NIGP01002	11	3	FALSO	1
8,57E+18	FALSO	Medium	IsMasterPr	A0A218KN	Conjugal tr	11	4	FALSO	2
-7,90E+18	FALSO	Medium	IsMasterPr	AAEL00226	2:3152461	11	2	FALSO	2

-1,81E+18	FALSO	Medium	IsMasterPr	AAEL00066	3:3953769	11	6	FALSO	2
-1,89E+18	FALSO	Medium	IsMasterPr	AAEL00435	3:9436546	11	2	FALSO	1
-4,48E+18	FALSO	Medium	IsMasterPr	AAEL00473	2:3787951	11	1	FALSO	2
2,21E+18	FALSO	Medium	IsMasterPr	AAEL01236	2:1116004	11	4	FALSO	2
3,42E+17	FALSO	Medium	IsMasterPr	AAEL02082	1:1563926	11	2	FALSO	2
-5,84E+18	FALSO	Medium	IsMasterPr	AAEL02077	NIGP01002	11	23	FALSO	2
8,80E+18	FALSO	Medium	IsMasterPr	AAEL01813	2:1282671	11	3	FALSO	1
-6,53E+18	FALSO	Medium	IsMasterPr	AAEL02147	2:6519509	11	3	FALSO	2
3,60E+17	FALSO	Medium	IsMasterPr	AAEL01167	2:3476147	11	7	FALSO	2
3,50E+18	FALSO	Medium	IsMasterPr	AAEL00818	3:9925829	11	6	FALSO	2
4,48E+18	FALSO	Medium	IsMasterPr	AAEL01239	1:2845661	11	4	FALSO	1
-4,56E+18	FALSO	Medium	IsMasterPr	AAEL00961	2:1697603	11	14	FALSO	2
-7,10E+18	FALSO	Medium	IsMasterPr	AAEL00214	1:2438716	11	24	FALSO	1
7,05E+18	FALSO	Medium	IsMasterPr	AAEL01818	2:4113514	11	3	FALSO	1
4,16E+18	FALSO	Medium	IsMasterPr	AAEL01963	2:1467589	11	0	FALSO	1
-1,27E+18	FALSO	Medium	IsMasterPr	AAEL01159	2:2663197	11	2	FALSO	1
-2,01E+18	FALSO	Medium	IsMasterPr	AAEL02289	2:1049863	11	2	FALSO	2
8,64E+18	FALSO	Medium	IsMasterPr	AAEL00183	2:3769669	11	9	FALSO	1
2,54E+18	FALSO	Medium	IsMasterPr	AAEL00765	1:1366592	11	7	FALSO	1
-8,93E+18	FALSO	Medium	IsMasterPr	AAEL02470	2:1550030	11	3	FALSO	1
3,59E+18	FALSO	Medium	IsMasterPr	AAEL00426	2:1185913	11	12	FALSO	2
-1,46E+18	FALSO	Medium	IsMasterPr	AAEL01958	2:5526266	11	3	FALSO	1
5,91E+18	FALSO	Medium	IsMasterPr	AAEL00047	3:1628210	11	16	FALSO	2
-5,70E+18	FALSO	Medium	IsMasterPr	AAEL00678	3:1689785	11	9	FALSO	1
6,56E+18	FALSO	Medium	IsMasterPr	A0A1E7QK	Uncharacte	11	31	FALSO	2
-8,80E+18	FALSO	Medium	IsMasterPr	A0A4Y6UQ	ANK_REP_I	11	7	FALSO	1
-6,03E+18	FALSO	Medium	IsMasterPr	AAEL00275	3:1449258	11	11	FALSO	1
-8,12E+18	FALSO	Medium	IsMasterPr	AAEL01943	3:1001637	11	5	FALSO	1
9,02E+18	FALSO	Medium	IsMasterPr	AAEL01436	3:7510695	11	7	FALSO	1
6,27E+18	FALSO	Medium	IsMasterPr	AAEL01975	2:4297032	11	9	FALSO	1
-5,31E+18	FALSO	Medium	IsMasterPr	AAEL00018	3:2162342	11	9	FALSO	2
-5,17E+18	FALSO	Medium	IsMasterPr	AAEL01483	3:3222102	11	45	FALSO	1
-8,20E+18	FALSO	Medium	IsMasterPr	AAEL01200	3:3079760	11	4	FALSO	1
2,64E+18	FALSO	Medium	IsMasterPr	AAEL01742	1:1594660	11	6	FALSO	1
2,26E+18	FALSO	Medium	IsMasterPr	A0A3B0J7N	tRNA-speci	12	9	FALSO	1
1,10E+18	FALSO	Medium	IsMasterPr	AAEL01973	1:2001760	12	2	FALSO	1
4,46E+18	FALSO	Medium	IsMasterPr	AAEL00242	1:1773334	12	1	FALSO	1
4,45E+18	FALSO	Medium	IsMasterPr	AAEL00394	3:2543471	12	11	FALSO	1
-1,03E+18	FALSO	Medium	IsMasterPr	AAEL01086	2:3835244	12	9	FALSO	1
-6,33E+18	FALSO	Medium	IsMasterPr	AAEL02208	1:8926628	12	2	FALSO	1
4,79E+18	FALSO	Medium	IsMasterPr	AAEL02289	2:1449817	12	2	FALSO	1
-6,58E+18	FALSO	Medium	IsMasterPr	AAEL00596	3:2277126	12	6	FALSO	2
-4,21E+18	FALSO	Medium	IsMasterPr	AAEL00452	2:3104412	12	2	FALSO	1
7,66E+18	FALSO	Medium	IsMasterPr	AAEL00988	3:1224483	12	1	FALSO	2
8,02E+18	FALSO	Medium	IsMasterPr	AAEL00396	3:2676679	12	2	FALSO	1
2,32E+18	FALSO	Medium	IsMasterPr	AAEL00643	2:4386324	12	3	FALSO	1
1,58E+18	FALSO	Medium	IsMasterPr	AAEL00399	3:2538045	12	2	FALSO	1
-4,43E+18	FALSO	Medium	IsMasterPr	AAEL00912	2:4188531	12	5	FALSO	2
-2,01E+18	FALSO	Medium	IsMasterPr	AAEL01474	1:3604814	12	7	FALSO	1
6,18E+18	FALSO	Medium	IsMasterPr	AAEL01484	2:1048722	12	3	FALSO	1

-6,29E+18	FALSO	Medium	IsMasterPr	AAEL0043€	3:9394194	12	5	FALSO	2
-1,95E+17	FALSO	Medium	IsMasterPr	AAEL00591	3:1739370	12	16	FALSO	1
-5,52E+18	FALSO	Medium	IsMasterPr	AAEL0201S	2:4077544	12	3	FALSO	1
-2,49E+18	FALSO	Medium	IsMasterPr	AAEL00544	1:8005246	12	3	FALSO	1
6,80E+18	FALSO	Medium	IsMasterPr	AAEL01716	2:8678552	12	14	FALSO	1
3,22E+18	FALSO	Medium	IsMasterPr	AAEL00697	2:3993191	12	11	FALSO	1
-4,77E+18	FALSO	Medium	IsMasterPr	AAEL01292	1:2059314	12	5	FALSO	1
-3,45E+18	FALSO	Medium	IsMasterPr	AAEL01117	2:1819803	12	3	FALSO	1
5,35E+18	FALSO	Medium	IsMasterPr	AAEL00491	2:7244746	12	6	FALSO	1
-4,89E+18	FALSO	Medium	IsMasterPr	AAEL00164	2:2993522	12	7	FALSO	1
-7,51E+18	FALSO	Medium	IsMasterPr	AAEL01384	1:1791486	13	5	FALSO	2
-1,91E+16	FALSO	Medium	IsMasterPr	AAEL01346	1:2063974	13	11	FALSO	1
3,18E+18	FALSO	Medium	IsMasterPr	AAEL0142S	2:1087693	13	12	FALSO	1
2,45E+18	FALSO	Medium	IsMasterPr	AAEL00535	3:3474398	13	6	FALSO	2
2,24E+18	FALSO	Medium	IsMasterPr	AAEL00353	2:2559152	13	3	FALSO	1
5,05E+18	FALSO	Medium	IsMasterPr	AAEL0029€	1:2613782	13	3	FALSO	1
-8,82E+18	FALSO	Medium	IsMasterPr	J7GTF9	Outer surfa	13	23	FALSO	1
-4,83E+18	FALSO	Medium	IsMasterPr	AAEL0003€	2:1243953	13	4	FALSO	1
-1,52E+18	FALSO	Medium	IsMasterPr	AAEL00588	3:2849234	13	2	FALSO	1
-2,13E+18	FALSO	Medium	IsMasterPr	AAEL02716	2:3378483	13	9	FALSO	2
-6,57E+18	FALSO	Medium	IsMasterPr	AAEL0040€	1:1354346	13	8	FALSO	1
7,70E+18	FALSO	Medium	IsMasterPr	AAEL00908	2:4023517	13	7	FALSO	1
-2,08E+18	FALSO	Medium	IsMasterPr	AAEL02071	1:2294074	13	6	FALSO	1
1,22E+18	FALSO	Medium	IsMasterPr	AAEL0080S	1:6156315	13	2	FALSO	1
-9,18E+18	FALSO	Medium	IsMasterPr	AAEL02123	1:9621705	13	4	FALSO	1
-2,79E+18	FALSO	Medium	IsMasterPr	A0A2A4IJD	Malate deh	13	14	FALSO	1
-6,31E+18	FALSO	Medium	IsMasterPr	AAEL00913	2:4188782	13	5	FALSO	2
1,17E+18	FALSO	Medium	IsMasterPr	AAEL00388	3:8464312	13	23	FALSO	1
7,00E+18	FALSO	Medium	IsMasterPr	AAEL00075	2:5212547	13	2	FALSO	1
2,56E+18	FALSO	Medium	IsMasterPr	AAEL00187	2:2767428	13	6	FALSO	1
2,82E+18	FALSO	Medium	IsMasterPr	AAEL00527	1:3043667	13	2	FALSO	1
2,92E+18	FALSO	Medium	IsMasterPr	AAEL01473	2:3371809	13	4	FALSO	1
3,26E+18	FALSO	Medium	IsMasterPr	AAEL01038	2:1825061	13	1	FALSO	1
-8,57E+18	FALSO	Medium	IsMasterPr	AAEL00625	3:6287029	13	41	FALSO	1
7,08E+18	FALSO	Medium	IsMasterPr	AAEL00902	2:1016834	13	5	FALSO	1
7,25E+18	FALSO	Medium	IsMasterPr	AAEL0043C	2:2934639	13	3	FALSO	1
7,81E+18	FALSO	Medium	IsMasterPr	AAEL00531	3:3100367	13	1	FALSO	1
-2,14E+18	FALSO	Medium	IsMasterPr	AAEL0121C	1:171612:1	13	5	FALSO	1
-2,42E+18	FALSO	Medium	IsMasterPr	AAEL02732	3:2610620	14	4	FALSO	1
-7,21E+18	FALSO	Medium	IsMasterPr	A0A218KN	4-diphosph	14	11	FALSO	1
1,21E+17	FALSO	Medium	IsMasterPr	AAEL00148	2:2623476	14	1	FALSO	1
-4,52E+18	FALSO	Medium	IsMasterPr	AAEL0061C	3:3074684	14	9	FALSO	1
-6,53E+18	FALSO	Medium	IsMasterPr	AAEL0224€	3:7548273	14	6	FALSO	1
-6,02E+18	FALSO	Medium	IsMasterPr	AAEL0234S	2:1113282	14	15	FALSO	1
-2,88E+18	FALSO	Medium	IsMasterPr	AAEL00114	3:4066004	14	3	FALSO	1
-5,05E+18	FALSO	Medium	IsMasterPr	AAEL0277S	NIGP0100C	15	17	FALSO	1
8,01E+18	FALSO	Medium	IsMasterPr	AAEL02277	1:4549107	15	7	FALSO	1
-5,20E+18	FALSO	Medium	IsMasterPr	A0A5F1B9(	DNA polym	15	19	FALSO	1
-2,88E+18	FALSO	Medium	IsMasterPr	AAEL01033	2:4041557	15	15	FALSO	1
4,45E+18	FALSO	Medium	IsMasterPr	A0A5F1B85	Uncharacte	15	4	FALSO	1

-1,92E+18	FALSO	Medium	IsMasterPr	AAEL02545	3:2062344	15	1	FALSO	1
-6,43E+18	FALSO	Medium	IsMasterPr	AAEL0000	3:1051596	15	4	FALSO	1
2,50E+17	FALSO	Medium	IsMasterPr	AAEL0186	MT:1298:2	15	7	FALSO	1
2,20E+18	FALSO	Medium	IsMasterPr	AAEL0066	3:2004869	15	11	FALSO	1
2,13E+18	FALSO	Medium	IsMasterPr	AAEL0123	2:1117056	15	5	FALSO	1
2,45E+18	FALSO	Medium	IsMasterPr	AAEL00195	3:2270872	15	3	FALSO	1
7,26E+17	FALSO	Medium	IsMasterPr	AAEL02732	1:2437405	15	25	FALSO	1
2,80E+17	FALSO	Medium	IsMasterPr	AAEL00365	3:6498128	16	7	FALSO	1
4,25E+18	FALSO	Medium	IsMasterPr	C0R3M1	Uncharacte	16	24	FALSO	1
8,26E+18	FALSO	Medium	IsMasterPr	AAEL01751	2:2701287	16	9	FALSO	1
-3,47E+18	FALSO	Medium	IsMasterPr	A0A2A4II0	Uncharacte	16	7	FALSO	1
3,45E+18	FALSO	Medium	IsMasterPr	AAEL00252	1:9012361	16	3	FALSO	1
-1,11E+18	FALSO	Medium	IsMasterPr	C0FAC2	Lon protea	16	5	FALSO	1
-7,19E+18	FALSO	Medium	IsMasterPr	AAEL01805	1:1144837	16	5	FALSO	1
-5,77E+18	FALSO	Medium	IsMasterPr	AAEL00513	NIGP0100	16	23	FALSO	1
-6,87E+18	FALSO	Medium	IsMasterPr	AAEL0060	2:4726458	16	9	FALSO	1
3,14E+18	FALSO	Medium	IsMasterPr	AAEL0078	3:3815640	16	3	FALSO	2
8,05E+18	FALSO	Medium	IsMasterPr	AAEL0037	2:1974063	16	9	FALSO	1
-9,34E+17	FALSO	Medium	IsMasterPr	AAEL0074	2:2916249	16	2	FALSO	2
5,74E+18	FALSO	Medium	IsMasterPr	AAEL0230	1:2153137	16	5	FALSO	1
-6,48E+18	FALSO	Medium	IsMasterPr	AAEL00731	2:2224933	16	7	FALSO	1
-4,50E+18	FALSO	Medium	IsMasterPr	AAEL0012	2:4133227	16	3	FALSO	1
5,74E+18	FALSO	Medium	IsMasterPr	AAEL00082	3:3638579	16	1	FALSO	1
-8,40E+18	FALSO	Medium	IsMasterPr	AAEL0152	2:1762903	16	6	FALSO	1
5,73E+18	FALSO	Medium	IsMasterPr	AAEL00692	3:1955160	16	3	FALSO	1
-6,56E+18	FALSO	Medium	IsMasterPr	A0A218KM	Uncharacte	16	12	FALSO	1
7,06E+18	FALSO	Medium	IsMasterPr	A0A2A3U1	Uncharacte	16	13	FALSO	1
3,11E+18	FALSO	Medium	IsMasterPr	AAEL0028	1:1081802	16	24	FALSO	1
-8,79E+18	FALSO	Medium	IsMasterPr	AAEL0015	2:2207966	16	7	FALSO	1
-5,86E+18	FALSO	Medium	IsMasterPr	AAEL01163	3:2815561	16	6	FALSO	1
5,25E+18	FALSO	Medium	IsMasterPr	A0A5R9M	Uncharacte	16	13	FALSO	1
-6,06E+18	FALSO	Medium	IsMasterPr	A0A060PV	Phosphate	16	12	FALSO	1
5,47E+17	FALSO	Medium	IsMasterPr	AAEL01398	3:2304829	16	10	FALSO	1
-3,86E+18	FALSO	Medium	IsMasterPr	A0A3T0GJ	Sodium:pro	16	11	FALSO	1
-7,39E+18	FALSO	Medium	IsMasterPr	A0A218KQ	Uncharacte	16	2	FALSO	1
-1,67E+18	FALSO	Medium	IsMasterPr	AAEL0140	3:1473852	16	1	FALSO	1
7,27E+18	FALSO	Medium	IsMasterPr	AAEL00547	3:3838460	16	4	FALSO	1
8,70E+18	FALSO	Medium	IsMasterPr	AAEL0111	3:1934928	16	5	FALSO	1
-9,19E+18	FALSO	Medium	IsMasterPr	AAEL0044	3:2712927	16	5	FALSO	1
7,85E+18	FALSO	Medium	IsMasterPr	AAEL00884	2:2408963	16	3	FALSO	1
-4,62E+18	FALSO	Medium	IsMasterPr	AAEL01035	3:3720400	16	2	FALSO	1
9,04E+18	FALSO	Medium	IsMasterPr	AAEL01171	2:1049229	16	3	FALSO	1
5,16E+18	FALSO	Medium	IsMasterPr	A0A1E7QK	ATP-depen	16	7	FALSO	2
5,35E+18	FALSO	Medium	IsMasterPr	AAEL0095	2:1344806	16	4	FALSO	1
-7,81E+17	FALSO	Medium	IsMasterPr	AAEL00643	2:4397552	16	2	FALSO	1
-8,48E+18	FALSO	Medium	IsMasterPr	A0A369RIU	Transposas	16	5	FALSO	1
-8,92E+18	FALSO	Medium	IsMasterPr	AAEL00983	3:1872939	16	1	FALSO	1
-6,10E+18	FALSO	Medium	IsMasterPr	AAEL00415	2:1463247	16	13	FALSO	1
9,01E+18	FALSO	Medium	IsMasterPr	AAEL01221	3:1703080	16	2	FALSO	1
-9,15E+18	FALSO	Medium	IsMasterPr	AAEL00464	1:3071790	16	5	FALSO	1

-7,84E+18	FALSO	Medium	IsMasterPr	AAEL01442	2:1366729	16	4	FALSO	2
8,74E+18	FALSO	Medium	IsMasterPr	AAEL00221	2:4283283	16	2	FALSO	1
9,91E+17	FALSO	Medium	IsMasterPr	A0A218KP(	Terminase	16	7	FALSO	1
-1,21E+18	FALSO	Medium	IsMasterPr	AAEL01054	2:3145514	16	6	FALSO	1
-7,21E+18	FALSO	Medium	IsMasterPr	A0A2A4IKI	Sodium:prc	16	11	FALSO	1
-7,68E+18	FALSO	Medium	IsMasterPr	B6Y850	Putative m	16	13	FALSO	1
7,34E+18	FALSO	Medium	IsMasterPr	AAEL00237	2:3030835	16	17	FALSO	1
9,56E+17	FALSO	Medium	IsMasterPr	AAEL01462	1:2420196	16	5	FALSO	1
6,46E+18	FALSO	Medium	IsMasterPr	AAEL00502	2:4445467	17	6	FALSO	1
7,04E+18	FALSO	Medium	IsMasterPr	AAEL00385	3:3432023	17	1	FALSO	1
7,08E+18	FALSO	Medium	IsMasterPr	AAEL00617	3:1404872	17	2	FALSO	1
-4,04E+18	FALSO	Medium	IsMasterPr	AAEL01284	2:3351786	17	3	FALSO	1
3,31E+18	FALSO	Medium	IsMasterPr	A0A3G5BS	Surface prc	17	69	FALSO	1
2,71E+18	FALSO	Medium	IsMasterPr	AAEL0211S	2:2702120	17	6	FALSO	1
4,32E+18	FALSO	Medium	IsMasterPr	AAEL00937	3:8516998	17	29	FALSO	1
-6,40E+18	FALSO	Medium	IsMasterPr	AAEL0076€	1:1345643	17	1	FALSO	1
-1,42E+18	FALSO	Medium	IsMasterPr	AAEL0085S	2:3631816	18	2	FALSO	1
3,09E+18	FALSO	Medium	IsMasterPr	AAEL01127	2:2266270	18	1	FALSO	1
8,17E+18	FALSO	Medium	IsMasterPr	AAEL01378	3:3201256	18	3	FALSO	1
1,29E+18	FALSO	Medium	IsMasterPr	Q5GTL8	2-polypren	18	10	FALSO	1
2,06E+18	FALSO	Medium	IsMasterPr	AAEL0083S	3:2244159	20	10	FALSO	1
3,78E+18	FALSO	Medium	IsMasterPr	AAEL01121	2:1945583	20	5	FALSO	1
7,47E+18	FALSO	Medium	IsMasterPr	AAEL00037	2:3677966	20	3	FALSO	1
-4,54E+18	FALSO	Medium	IsMasterPr	A0A3T0GI5	DNA polym	20	3	FALSO	1
1,92E+18	FALSO	Medium	IsMasterPr	A0A1V2N6	Uncharacte	21	3	FALSO	1
-1,82E+18	FALSO	Medium	IsMasterPr	AAEL0062€	2:2202697	21	8	FALSO	1
-1,37E+18	FALSO	Medium	IsMasterPr	AAEL0021C	3:1772474	21	2	FALSO	1
8,53E+18	FALSO	Medium	IsMasterPr	A0A225X5\	Uncharacte	21	4	FALSO	1
1,61E+18	FALSO	Medium	IsMasterPr	AAEL0023S	2:2144628	21	1	FALSO	2
4,23E+18	FALSO	Medium	IsMasterPr	AAEL0076C	3:1810956	21	6	FALSO	1
-4,61E+18	FALSO	Medium	IsMasterPr	AAEL0034€	NIGP01002	21	3	FALSO	1
7,33E+18	FALSO	Medium	IsMasterPr	AAEL01834	2:2854419	21	1	FALSO	1
-2,90E+17	FALSO	Medium	IsMasterPr	M9WSJ3	Ankyrin rep	21	3	FALSO	1
3,42E+18	FALSO	Medium	IsMasterPr	AAEL01153	2:1231676	21	5	FALSO	1
4,75E+18	FALSO	Medium	IsMasterPr	A0A4S2QT	Rod shape-	21	9	FALSO	1
8,42E+18	FALSO	Medium	IsMasterPr	AAEL01108	2:2356078	21	8	FALSO	1
-5,83E+18	FALSO	Medium	IsMasterPr	AAEL01705	2:4628964	21	6	FALSO	1
-3,78E+18	FALSO	Medium	IsMasterPr	AAEL0015C	2:2622742	22	13	FALSO	1
2,67E+17	FALSO	Medium	IsMasterPr	AAEL00613	3:6848750	23	3	FALSO	1
-2,02E+18	FALSO	Medium	IsMasterPr	AAEL01405	2:3429090	23	3	FALSO	1
2,94E+18	FALSO	Medium	IsMasterPr	A0A218KN	Insulinase f	23	8	FALSO	1
1,93E+18	FALSO	Medium	IsMasterPr	AAEL00332	3:3306627	23	3	FALSO	1
3,50E+17	FALSO	Medium	IsMasterPr	AAEL0088S	3:3371588	24	9	FALSO	1
4,30E+18	FALSO	Medium	IsMasterPr	AAEL0050€	1:2980604	24	4	FALSO	1
3,28E+18	FALSO	Medium	IsMasterPr	AAEL02687	NIGP0100C	24	3	FALSO	1
-3,96E+18	FALSO	Medium	IsMasterPr	AAEL00581	1:2002540	24	2	FALSO	1
-4,01E+18	FALSO	Medium	IsMasterPr	AAEL0034S	2:2163432	25	2	FALSO	1
-4,14E+17	FALSO	Medium	IsMasterPr	A0A218KQ	Uncharacte	25	41	FALSO	1
-7,60E+18	FALSO	Medium	IsMasterPr	AAEL0028S	1:1084764	25	5	FALSO	1
-5,52E+18	FALSO	Medium	IsMasterPr	AAEL00334	2:4001860	25	4	FALSO	1

8,64E+18	FALSO	Medium	IsMasterPr	AAEL0069€	2:3988379:	25	3	FALSO	1
-5,95E+18	FALSO	Medium	IsMasterPr	AAEL02543	1:3077150:	25	5	FALSO	1
-6,83E+17	FALSO	Medium	IsMasterPr	Q73G52	Uncharacte	25	11	FALSO	1
8,87E+18	FALSO	Medium	IsMasterPr	AAEL00916	1:1565118:	25	6	FALSO	1
-2,65E+18	FALSO	Medium	IsMasterPr	AAEL00764	1:1872611!	25	6	FALSO	1
3,91E+18	FALSO	Medium	IsMasterPr	AAEL00317	1:1931030!	25	4	FALSO	1
-4,98E+18	FALSO	Medium	IsMasterPr	A0A5R9MG	ATP-depen	25	24	FALSO	1
-8,82E+18	FALSO	Medium	IsMasterPr	AAEL00865	3:1725518!	25	10	FALSO	2
6,56E+18	FALSO	Medium	IsMasterPr	AAEL01042	1:2680104	26	7	FALSO	1
-7,57E+18	FALSO	Medium	IsMasterPr	AAEL0011C	3:4064605!	26	3	FALSO	2
-9,14E+18	FALSO	Medium	IsMasterPr	AAEL00255	1:2272738:	26	6	FALSO	1
5,60E+18	FALSO	Medium	IsMasterPr	A0A218KN	Single-strar	26	21	FALSO	1
-3,33E+17	FALSO	Medium	IsMasterPr	A0A369RN.	Dna-J like r	27	26	FALSO	1
-8,27E+18	FALSO	Medium	IsMasterPr	A0A2A4IJY:	Polyribonu	27	5	FALSO	1
7,40E+17	FALSO	Medium	IsMasterPr	AAEL00724	2:4218105(	27	1	FALSO	2
-5,89E+18	FALSO	Medium	IsMasterPr	AAEL00632	2:1408458(	27	1	FALSO	1
-2,70E+18	FALSO	Medium	IsMasterPr	A0A178GT,	ANK_REP_I	27	3	FALSO	1
-4,73E+18	FALSO	Medium	IsMasterPr	A0A059IWI	Uncharacte	28	2	FALSO	1
4,00E+18	FALSO	Medium	IsMasterPr	AAEL0270€	3:3413506	28	3	FALSO	1
-3,12E+17	FALSO	Medium	IsMasterPr	AAEL0092!	2:1791862:	28	2	FALSO	1
8,19E+18	FALSO	Medium	IsMasterPr	AAEL01811	2:4557227	28	2	FALSO	1
3,33E+18	FALSO	Medium	IsMasterPr	AAEL00974	3:2842682:	28	4	FALSO	1
-8,05E+18	FALSO	Medium	IsMasterPr	AAEL01944	3:1109490	29	2	FALSO	1
1,49E+17	FALSO	Medium	IsMasterPr	A0A098ASI	Surface prc	29	14	FALSO	1
3,71E+18	FALSO	Medium	IsMasterPr	A0A3D1L6I	Collagen-lil	29	10	FALSO	1
-6,22E+18	FALSO	Medium	IsMasterPr	AAEL01952	2:1350933(	29	3	FALSO	1
-4,94E+18	FALSO	Medium	IsMasterPr	AAEL02715	3:6017914	29	1	FALSO	1
-1,70E+18	FALSO	Medium	IsMasterPr	AAEL00285	1:2556716:	29	7	FALSO	1
1,98E+18	FALSO	Medium	IsMasterPr	A0A5R9MI	50S ribosor	29	26	FALSO	1
-6,55E+18	FALSO	Medium	IsMasterPr	AAEL00458	2:3374828	29	1	FALSO	1
-5,49E+18	FALSO	Medium	IsMasterPr	AAEL0028€	1:2550594	29	3	FALSO	1
-3,54E+18	FALSO	Medium	IsMasterPr	AAEL00003	3:1034950	29	14	FALSO	1
9,14E+18	FALSO	Medium	IsMasterPr	AAEL00964	1:6061010!	30	3	FALSO	1
2,08E+18	FALSO	Medium	IsMasterPr	AAEL02364	NIGP0100C	30	1	FALSO	1
7,20E+18	FALSO	Medium	IsMasterPr	AAEL0007!	3:2434803:	30	7	FALSO	1
-2,60E+18	FALSO	Medium	IsMasterPr	AAEL01115	3:1941190:	30	19	FALSO	1
-6,27E+17	FALSO	Medium	IsMasterPr	A0A059IVZ	Glutamine-	30	6	FALSO	1
8,66E+18	FALSO	Medium	IsMasterPr	AAEL0090!	3:1352224(	30	7	FALSO	1
4,05E+18	FALSO	Medium	IsMasterPr	AAEL0247C	2:2329323!	30	5	FALSO	1
3,50E+18	FALSO	Medium	IsMasterPr	AAEL02268	2:1973232	31	2	FALSO	1
3,05E+18	FALSO	Medium	IsMasterPr	AAEL00137	2:2055961!	31	5	FALSO	1
4,18E+18	FALSO	Medium	IsMasterPr	AAEL0107!	1:1785698(	31	7	FALSO	1
3,56E+18	FALSO	Medium	IsMasterPr	AAEL0213!	2:1735346	31	1	FALSO	2
-1,78E+17	FALSO	Medium	IsMasterPr	AAEL00612	3:6874520!	31	11	FALSO	1
1,98E+18	FALSO	Medium	IsMasterPr	AAEL00621	2:4915744:	33	3	FALSO	1
8,36E+18	FALSO	Medium	IsMasterPr	AAEL0112C	2:1946153	33	4	FALSO	1
-5,49E+18	FALSO	Medium	IsMasterPr	AAEL0044!	2:4523307(	33	12	FALSO	1
6,04E+18	FALSO	Medium	IsMasterPr	A0A369RH	Enolase OS	33	8	FALSO	1
-5,03E+18	FALSO	Medium	IsMasterPr	A0A178GY	Uncharacte	33	24	FALSO	1
-7,36E+18	FALSO	Medium	IsMasterPr	A0A178GS!	Ribosomal	34	11	FALSO	1

-6,35E+18	FALSO	Medium	IsMasterPr	AAEL00313	3:2709580	34	5	FALSO	2
-3,26E+18	FALSO	Medium	IsMasterPr	A0A218KPI	DUF4815 d	34	4	FALSO	1
5,34E+18	FALSO	Medium	IsMasterPr	AAEL01987	3:1795126	34	1	FALSO	1
4,05E+18	FALSO	Medium	IsMasterPr	AAEL01405	NIGP01002	34	3	FALSO	1
7,13E+18	FALSO	Medium	IsMasterPr	AAEL02195	1:5744671	34	1	FALSO	1
-4,00E+18	FALSO	Medium	IsMasterPr	A0A176Q8	Phenylalan	34	2	FALSO	1
-1,57E+18	FALSO	Medium	IsMasterPr	AAEL02481	3:1045690	34	8	FALSO	1
5,56E+18	FALSO	Medium	IsMasterPr	AAEL02739	2:3990227	34	16	FALSO	1
-3,36E+18	FALSO	Medium	IsMasterPr	AAEL01142	3:3290648	35	4	FALSO	1
6,86E+17	FALSO	Medium	IsMasterPr	AAEL00469	1:1188342	35	15	FALSO	1
-2,76E+18	FALSO	Medium	IsMasterPr	AAEL00279	3:1292725	37	10	FALSO	1
-8,46E+18	FALSO	Medium	IsMasterPr	AAEL00294	1:8600444	37	1	FALSO	1
-4,38E+18	FALSO	Medium	IsMasterPr	AAEL00377	1:2030688	37	1	FALSO	1
7,37E+18	FALSO	Medium	IsMasterPr	AAEL02506	2:5862583	37	1	FALSO	1
6,29E+18	FALSO	Medium	IsMasterPr	AAEL00683	1:8318108	37	4	FALSO	1
-5,28E+18	FALSO	Medium	IsMasterPr	AAEL01424	2:3621647	37	12	FALSO	1
-7,26E+18	FALSO	Medium	IsMasterPr	A0A098AT8	Ferredoxin	37	12	FALSO	1
-5,21E+18	FALSO	Medium	IsMasterPr	Q5GTQ4	Cysteine de	37	8	FALSO	1
9,57E+17	FALSO	Medium	IsMasterPr	AAEL00844	2:3294032	37	10	FALSO	1
7,06E+18	FALSO	Medium	IsMasterPr	B7TW69	NADH dehy	38	21	FALSO	1
1,89E+18	FALSO	Medium	IsMasterPr	AAEL02318	2:3517954	38	2	FALSO	1
7,34E+18	FALSO	Medium	IsMasterPr	AAEL01376	3:3360784	38	6	FALSO	1
-5,13E+18	FALSO	Medium	IsMasterPr	AAEL00943	2:2467336	39	1	FALSO	1
6,80E+18	FALSO	Medium	IsMasterPr	AAEL01079	1:1786188	39	3	FALSO	1
-9,22E+18	FALSO	Medium	IsMasterPr	AAEL02545	3:1200538	39	3	FALSO	1
-2,30E+17	FALSO	Medium	IsMasterPr	AAEL00199	2:4243729	39	1	FALSO	1
2,68E+18	FALSO	Medium	IsMasterPr	AAEL01449	2:4647699	39	2	FALSO	1
-2,65E+18	FALSO	Medium	IsMasterPr	AAEL00824	3:6283153	39	5	FALSO	1
9,05E+18	FALSO	Medium	IsMasterPr	AAEL00118	2:3090799	39	5	FALSO	1
7,62E+18	FALSO	Medium	IsMasterPr	AAEL00038	3:9737702	39	4	FALSO	1
-2,57E+18	FALSO	Medium	IsMasterPr	A0A178GZ	DNA misma	39	3	FALSO	1
-5,54E+18	FALSO	Medium	IsMasterPr	AAEL00962	3:3672211	39	7	FALSO	1
4,60E+18	FALSO	Medium	IsMasterPr	AAEL00072	2:4593255	39	6	FALSO	1
7,04E+18	FALSO	Medium	IsMasterPr	AAEL01156	3:1068147	39	3	FALSO	1
7,10E+18	FALSO	Medium	IsMasterPr	AAEL00447	NIGP01001	39	12	FALSO	1
7,30E+18	FALSO	Medium	IsMasterPr	AAEL00457	1:2207521	39	2	FALSO	1
7,30E+18	FALSO	Medium	IsMasterPr	AAEL01451	3:3133972	39	4	FALSO	1
5,97E+18	FALSO	Medium	IsMasterPr	AAEL01189	2:3961052	39	8	FALSO	1
-6,77E+18	FALSO	Medium	IsMasterPr	Q5GSI9	Ankyrin rep	40	3	FALSO	2
6,55E+18	FALSO	Medium	IsMasterPr	AAEL02170	2:1006284	40	50	FALSO	1
6,26E+18	FALSO	Medium	IsMasterPr	AAEL01234	1:3033415	40	5	FALSO	1
7,77E+18	FALSO	Medium	IsMasterPr	AAEL00776	2:2202094	40	5	FALSO	1
-1,50E+18	FALSO	Medium	IsMasterPr	AAEL00303	1:1290688	40	4	FALSO	1
5,28E+18	FALSO	Medium	IsMasterPr	AAEL00067	3:3951445	40	15	FALSO	1
-5,05E+18	FALSO	Medium	IsMasterPr	C0F8P0	Uncharacte	40	5	FALSO	1
-3,93E+18	FALSO	Medium	IsMasterPr	AAEL01197	1:2855500	40	13	FALSO	1
4,74E+18	FALSO	Medium	IsMasterPr	AAEL00568	3:1155256	40	3	FALSO	1
7,38E+18	FALSO	Medium	IsMasterPr	AAEL01947	2:4727713	41	3	FALSO	1
-8,67E+18	FALSO	Medium	IsMasterPr	AAEL00680	3:3684605	41	3	FALSO	1
8,88E+18	FALSO	Medium	IsMasterPr	AAEL02319	1:1461922	41	1	FALSO	1

-7,57E+18	FALSO	Medium	IsMasterPr	AAEL01244	1:2133261	41	2	FALSO	1
1,78E+18	FALSO	Medium	IsMasterPr	AAEL0207C	3:2863696	41	2	FALSO	1
3,51E+18	FALSO	Medium	IsMasterPr	AAEL00605	3:7435307	41	3	FALSO	1
1,54E+18	FALSO	Medium	IsMasterPr	AAEL00744	2:2913209	41	12	FALSO	1
4,53E+18	FALSO	Medium	IsMasterPr	AAEL00855	2:1573767	41	1	FALSO	1
6,82E+17	FALSO	Medium	IsMasterPr	AAEL01075	3:2941174	41	0	FALSO	1
5,41E+18	FALSO	Medium	IsMasterPr	AAEL00965	1:6059450	41	4	FALSO	1
-7,18E+18	FALSO	Medium	IsMasterPr	AAEL00201	2:4253449	43	8	FALSO	1
2,78E+18	FALSO	Medium	IsMasterPr	AAEL00211	3:1784418	43	3	FALSO	1
-3,45E+18	FALSO	Medium	IsMasterPr	AAEL00731	2:2226450	43	3	FALSO	1
-8,69E+18	FALSO	Medium	IsMasterPr	A0A218KPE	Uncharacte	43	1	FALSO	2
8,33E+18	FALSO	Medium	IsMasterPr	AAEL00201	2:4263908	43	4	FALSO	1
9,01E+18	FALSO	Medium	IsMasterPr	AAEL00443	3:2064484	43	8	FALSO	1
3,43E+18	FALSO	Medium	IsMasterPr	AAEL01376	1:1626448	43	8	FALSO	1
-5,23E+18	FALSO	Medium	IsMasterPr	AAEL0104C	1:2687848	43	5	FALSO	1
-1,71E+18	FALSO	Medium	IsMasterPr	AAEL01421	3:2931900	43	2	FALSO	1
3,22E+18	FALSO	Medium	IsMasterPr	AAEL02512	2:9327363	43	1	FALSO	1
4,61E+18	FALSO	Medium	IsMasterPr	AAEL01942	1:4573017	43	3	FALSO	1
6,65E+18	FALSO	Medium	IsMasterPr	AAEL01386	1:1192602	43	2	FALSO	1
-9,02E+18	FALSO	Medium	IsMasterPr	AAEL01197	1:2859432	43	1	FALSO	1
8,25E+18	FALSO	Medium	IsMasterPr	AAEL01949	2:4185263	43	1	FALSO	1
-8,86E+18	FALSO	Medium	IsMasterPr	A0A098ASC	Uncharacte	43	11	FALSO	1
3,68E+18	FALSO	Medium	IsMasterPr	AAEL02793	NIGP0100C	43	17	FALSO	1
5,17E+18	FALSO	Medium	IsMasterPr	AAEL00443	3:2050869	43	2	FALSO	1
-3,19E+18	FALSO	Medium	IsMasterPr	AAEL00876	3:3268164	43	24	FALSO	1
6,23E+18	FALSO	Medium	IsMasterPr	AAEL01949	NIGP01001	43	18	FALSO	1
-3,18E+18	FALSO	Medium	IsMasterPr	AAEL00972	1:7843683	43	8	FALSO	1
1,59E+18	FALSO	Medium	IsMasterPr	AAEL00702	3:1706532	43	4	FALSO	1
5,45E+18	FALSO	Medium	IsMasterPr	AAEL0040C	3:1668969	43	4	FALSO	1
5,64E+18	FALSO	Medium	IsMasterPr	AAEL00047	NIGP0100C	43	4	FALSO	1
-8,31E+18	FALSO	Medium	IsMasterPr	AAEL00047	3:1614342	43	5	FALSO	1
8,72E+18	FALSO	Medium	IsMasterPr	AAEL02765	1:7538850	43	5	FALSO	1
-3,31E+18	FALSO	Medium	IsMasterPr	AAEL02743	2:2575681	43	6	FALSO	1
6,79E+18	FALSO	Medium	IsMasterPr	AAEL01955	2:2958916	43	3	FALSO	1
-1,30E+18	FALSO	Medium	IsMasterPr	AAEL01985	3:1990606	43	2	FALSO	1
1,22E+18	FALSO	Medium	IsMasterPr	AAEL00981	3:2031489	43	7	FALSO	1
7,91E+18	FALSO	Medium	IsMasterPr	AAEL00756	2:2488932	43	13	FALSO	1
7,87E+18	FALSO	Medium	IsMasterPr	AAEL00846	1:5339746	43	8	FALSO	1
7,61E+18	FALSO	Medium	IsMasterPr	AAEL01225	1:7690233	43	12	FALSO	1
2,81E+18	FALSO	Medium	IsMasterPr	AAEL00924	2:1716842	43	8	FALSO	1
-1,51E+18	FALSO	Medium	IsMasterPr	AAEL01019	3:3078044	43	8	FALSO	1
-6,69E+18	FALSO	Medium	IsMasterPr	AAEL01359	2:1035054	43	1	FALSO	1
6,88E+17	FALSO	Medium	IsMasterPr	AAEL00291	1:1086846	43	3	FALSO	1
-9,03E+18	FALSO	Medium	IsMasterPr	AAEL01085	1:5444162	43	3	FALSO	1
-4,31E+18	FALSO	Medium	IsMasterPr	AAEL01826	2:1002849	43	1	FALSO	1
-4,52E+17	FALSO	Medium	IsMasterPr	AAEL00351	2:2568647	43	14	FALSO	1
-5,80E+17	FALSO	Medium	IsMasterPr	AAEL01489	3:2908675	43	3	FALSO	1
5,63E+18	FALSO	Medium	IsMasterPr	AAEL01175	1:1474078	43	7	FALSO	1
-8,60E+18	FALSO	Medium	IsMasterPr	AAEL01829	3:1822005	43	2	FALSO	1
-5,47E+17	FALSO	Medium	IsMasterPr	AAEL00768	2:2501331	43	2	FALSO	1

-4,57E+18	FALSO	Medium	IsMasterPr	AAEL0105C	2:4462915	43	1	FALSO	1
8,17E+18	FALSO	Medium	IsMasterPr	AAEL02627	3:7490313	43	13	FALSO	1
6,39E+18	FALSO	Medium	IsMasterPr	AAEL00071	2:4593917	43	2	FALSO	1
1,11E+18	FALSO	Medium	IsMasterPr	AAEL01043	3:6759665	43	3	FALSO	1
-2,42E+18	FALSO	Medium	IsMasterPr	AAEL0047C	1:1185638	43	6	FALSO	1
8,60E+18	FALSO	Medium	IsMasterPr	AAEL01227	2:1450579	43	4	FALSO	1
-5,98E+18	FALSO	Medium	IsMasterPr	AAEL02557	2:4689806	43	3	FALSO	1
-2,71E+18	FALSO	Medium	IsMasterPr	AAEL0199C	3:2255590	43	0	FALSO	1
6,13E+17	FALSO	Medium	IsMasterPr	AAEL0041€	1:1224714	43	4	FALSO	1
8,02E+18	FALSO	Medium	IsMasterPr	AAEL01244	1:2133745	43	5	FALSO	1
-3,45E+18	FALSO	Medium	IsMasterPr	AAEL0209€	1:4391880	43	3	FALSO	1
-5,60E+17	FALSO	Medium	IsMasterPr	AAEL01095	3:1677585	43	3	FALSO	1
3,88E+18	FALSO	Medium	IsMasterPr	AAEL00055	3:3880193	43	4	FALSO	1
1,26E+18	FALSO	Medium	IsMasterPr	AAEL00038	3:4006969	43	4	FALSO	1
2,18E+18	FALSO	Medium	IsMasterPr	AAEL00384	3:3427144	43	26	FALSO	1
5,73E+18	FALSO	Medium	IsMasterPr	AAEL01327	2:9476195	43	1	FALSO	1
8,78E+18	FALSO	Medium	IsMasterPr	AAEL02414	2:2553738	43	2	FALSO	1
8,56E+18	FALSO	Medium	IsMasterPr	AAEL00782	2:2726016	43	2	FALSO	1
5,93E+18	FALSO	Medium	IsMasterPr	AAEL02638	3:7401755	43	11	FALSO	1
2,89E+18	FALSO	Medium	IsMasterPr	AAEL02361	3:2222864	43	1	FALSO	1
-3,33E+18	FALSO	Medium	IsMasterPr	AAEL0083€	3:4139876	43	17	FALSO	1
-1,48E+18	FALSO	Medium	IsMasterPr	A0A178GT(	GDSL famil	43	12	FALSO	1
-3,52E+18	FALSO	Medium	IsMasterPr	AAEL00437	3:9311097	43	3	FALSO	1
-2,54E+18	FALSO	Medium	IsMasterPr	AAEL02713	1:1689366	43	5	FALSO	1
1,25E+18	FALSO	Medium	IsMasterPr	AAEL0093S	3:3807907	43	6	FALSO	1
6,26E+18	FALSO	Medium	IsMasterPr	AAEL01318	3:8431159	43	9	FALSO	1
1,26E+18	FALSO	Medium	IsMasterPr	AAEL00198	2:4266020	43	3	FALSO	1
2,01E+18	FALSO	Medium	IsMasterPr	AAEL00217	1:2530545	43	5	FALSO	1
-4,46E+18	FALSO	Medium	IsMasterPr	AAEL0047€	2:2899571	43	10	FALSO	1
8,22E+18	FALSO	Medium	IsMasterPr	AAEL01868	MT:11547:	43	6	FALSO	1
-7,63E+18	FALSO	Medium	IsMasterPr	AAEL00874	3:2804147	43	4	FALSO	1
-1,21E+18	FALSO	Medium	IsMasterPr	AAEL0088S	3:3375974	43	16	FALSO	1
-7,71E+18	FALSO	Medium	IsMasterPr	AAEL01554	3:2956137	43	3	FALSO	1
-1,44E+18	FALSO	Medium	IsMasterPr	AAEL00457	1:1445341	43	5	FALSO	1
-3,78E+18	FALSO	Medium	IsMasterPr	AAEL01557	1:1941760	43	18	FALSO	1
-7,79E+18	FALSO	Medium	IsMasterPr	AAEL01202	3:1471982	43	4	FALSO	1
-5,25E+18	FALSO	Medium	IsMasterPr	AAEL0147S	2:3243045	43	16	FALSO	1
-3,77E+18	FALSO	Medium	IsMasterPr	AAEL02091	NIGP01001	43	3	FALSO	1
6,60E+18	FALSO	Medium	IsMasterPr	AAEL00677	3:2403633	43	11	FALSO	1
7,90E+18	FALSO	Medium	IsMasterPr	A0A4S2QR'	Ribonuclea	43	16	FALSO	1
-4,70E+18	FALSO	Medium	IsMasterPr	AAEL00603	3:3158667	43	2	FALSO	1
8,58E+18	FALSO	Medium	IsMasterPr	AAEL0083€	3:4138979	43	15	FALSO	1
-3,59E+18	FALSO	Medium	IsMasterPr	AAEL0071€	3:1908056	44	11	FALSO	1
3,48E+18	FALSO	Medium	IsMasterPr	AAEL01988	2:1025726	44	2	FALSO	1
8,56E+18	FALSO	Medium	IsMasterPr	AAEL00732	2:2227335	44	13	FALSO	1
2,83E+18	FALSO	Medium	IsMasterPr	AAEL0133C	3:1001343	44	10	FALSO	1
6,40E+18	FALSO	Medium	IsMasterPr	AAEL01984	3:3726795	44	3	FALSO	1
-5,62E+18	FALSO	Medium	IsMasterPr	AAEL00324	1:2909081	44	4	FALSO	1
-2,40E+18	FALSO	Medium	IsMasterPr	AAEL00187	2:2742887	44	6	FALSO	1
4,61E+18	FALSO	Medium	IsMasterPr	AAEL01203	3:1473715	44	5	FALSO	1

-6,67E+18	FALSO	Medium	IsMasterPr	AAEL00904	2:1605954!	44	4	FALSO	1
9,83E+17	FALSO	Medium	IsMasterPr	AAEL01309	2:33780910	44	3	FALSO	1
5,75E+18	FALSO	Medium	IsMasterPr	AAEL00867	2:2646959	44	7	FALSO	1
2,16E+18	FALSO	Medium	IsMasterPr	AAEL00763	2:1068530	44	6	FALSO	1
-3,72E+18	FALSO	Medium	IsMasterPr	AAEL01346	1:2064891	44	2	FALSO	1
7,16E+18	FALSO	Medium	IsMasterPr	AAEL00338	3:8043196	44	3	FALSO	1
3,99E+18	FALSO	Medium	IsMasterPr	AAEL00254	1:2265531	45	2	FALSO	1
-7,21E+18	FALSO	Medium	IsMasterPr	AAEL00497	2:2812631	45	4	FALSO	1
3,68E+18	FALSO	Medium	IsMasterPr	AAEL00337	3:8082654	46	3	FALSO	1
3,96E+18	FALSO	Medium	IsMasterPr	AAEL02582	2:1280645	46	4	FALSO	1
7,91E+18	FALSO	Medium	IsMasterPr	AAEL01311	1:1428851	46	3	FALSO	1
-6,50E+18	FALSO	Medium	IsMasterPr	AAEL00422	3:11752420	47	4	FALSO	1
-8,50E+18	FALSO	Medium	IsMasterPr	A0A218KQ	4-hydroxy-	47	9	FALSO	1
4,65E+18	FALSO	Medium	IsMasterPr	A0A2A4IHK	Ketol-acid r	47	5	FALSO	1
-8,55E+18	FALSO	Medium	IsMasterPr	A0A5C4TL4	Elongation	47	4	FALSO	1
4,08E+18	FALSO	Medium	IsMasterPr	AAEL00462	1:3062693	47	2	FALSO	1
-7,72E+18	FALSO	Medium	IsMasterPr	A0A225X80	Tyrosine--t	47	6	FALSO	1
-3,22E+18	FALSO	Medium	IsMasterPr	AAEL00096	3:37706920	47	3	FALSO	1
8,47E+17	FALSO	Medium	IsMasterPr	AAEL01056	1:50774190	47	4	FALSO	1
-6,88E+18	FALSO	Medium	IsMasterPr	AAEL00486	1:2885351	47	4	FALSO	1
-2,26E+18	FALSO	Medium	IsMasterPr	AAEL02307	3:2696971	47	1	FALSO	1
6,26E+18	FALSO	Medium	IsMasterPr	AAEL01112	3:3786638	47	4	FALSO	1
-1,67E+17	FALSO	Medium	IsMasterPr	AAEL02146	1:3427760	47	2	FALSO	1
7,51E+18	FALSO	Medium	IsMasterPr	AAEL01226	2:3840542	47	4	FALSO	1
-7,31E+18	FALSO	Medium	IsMasterPr	AAEL02084	2:3697264	48	4	FALSO	1
-4,22E+18	FALSO	Medium	IsMasterPr	AAEL01014	2:26497390	48	9	FALSO	1
-3,12E+18	FALSO	Medium	IsMasterPr	AAEL01092	1:2312872	48	2	FALSO	1
-1,10E+16	FALSO	Medium	IsMasterPr	AAEL00000	3:1027995	48	3	FALSO	1
3,38E+18	FALSO	Medium	IsMasterPr	AAEL00160	1:1388216	48	4	FALSO	1
3,02E+18	FALSO	Medium	IsMasterPr	A0A369RLS	Holliday jur	48	21	FALSO	1
1,15E+18	FALSO	Medium	IsMasterPr	AAEL00883	3:3298583	49	1	FALSO	1
1,17E+18	FALSO	Medium	IsMasterPr	A0A3B0IV4	Chaperone	49	5	FALSO	1
4,25E+17	FALSO	Medium	IsMasterPr	AAEL02092	2:4924230	49	5	FALSO	1
-6,65E+18	FALSO	Medium	IsMasterPr	AAEL01394	2:65106300	49	5	FALSO	1
7,68E+18	FALSO	Medium	IsMasterPr	AAEL00046	3:18774150	49	10	FALSO	1
-8,08E+18	FALSO	Medium	IsMasterPr	AAEL00849	2:19186500	49	3	FALSO	1
1,22E+18	FALSO	Medium	IsMasterPr	AAEL00126	2:1529834	49	10	FALSO	1
2,00E+18	FALSO	Medium	IsMasterPr	I7ITT6	Glyceraldel	49	7	FALSO	1
8,76E+18	FALSO	Medium	IsMasterPr	AAEL00668	3:3527725	49	28	FALSO	1
8,29E+18	FALSO	Medium	IsMasterPr	AAEL00222	2:4278051	49	8	FALSO	1
-8,94E+18	FALSO	Medium	IsMasterPr	A0A1S5R26	Aspartyl/gl	49	8	FALSO	1
-6,88E+18	FALSO	Medium	IsMasterPr	AAEL00753	3:1001971	49	1	FALSO	1
-7,08E+18	FALSO	Medium	IsMasterPr	AAEL01143	2:2251665	49	1	FALSO	1
8,12E+18	FALSO	Medium	IsMasterPr	AAEL01113	2:1030056	50	6	FALSO	1
-4,43E+17	FALSO	Medium	IsMasterPr	A0A1E7QK	DNA-direct	50	2	FALSO	1
-6,02E+18	FALSO	Medium	IsMasterPr	AAEL01254	2:2383634	50	1	FALSO	1
1,49E+18	FALSO	Medium	IsMasterPr	A0A5F1B84	Peptidylpro	50	4	FALSO	1
3,02E+18	FALSO	Medium	IsMasterPr	A0A369RIV	DNA misma	50	4	FALSO	1
7,57E+18	FALSO	Medium	IsMasterPr	AAEL02021	1:1557897	50	9	FALSO	1
-3,36E+18	FALSO	Medium	IsMasterPr	AAEL01555	2:2507856	50	3	FALSO	1

8,11E+17	FALSO	Medium	IsMasterPr	D5LGC9	Probable se	50	5	FALSO	1
6,40E+18	FALSO	Medium	IsMasterPr	Q5GTF6	Peroxiredo	50	19	FALSO	1
8,86E+17	FALSO	Medium	IsMasterPr	AAEL00671	1:16761529	50	7	FALSO	1
5,95E+18	FALSO	Medium	IsMasterPr	AAEL00548	3:60227661	50	2	FALSO	1
2,35E+18	FALSO	Medium	IsMasterPr	AAEL00712	3:69814001	50	19	FALSO	1
6,68E+18	FALSO	Medium	IsMasterPr	AAEL0064C	2:41389831	50	3	FALSO	1
-3,75E+18	FALSO	Medium	IsMasterPr	A0A3B0J1D	Peptide cha	50	9	FALSO	1
-6,61E+18	FALSO	Medium	IsMasterPr	AAEL0231C	3:36590158	50	2	FALSO	1
5,23E+18	FALSO	Low	IsMasterPr	AAEL02365	2:57825820	50	4	FALSO	1
3,93E+17	FALSO	Low	IsMasterPr	AAEL00778	3:13348897	50	6	FALSO	1
2,79E+18	FALSO	Low	IsMasterPr	A0A2A4IK6	Uncharacte	50	14	FALSO	1
6,47E+18	FALSO	Low	IsMasterPr	AAEL0092C	3:39095828	51	2	FALSO	1
-3,83E+18	FALSO	Low	IsMasterPr	AAEL00436	3:94168230	51	2	FALSO	1
4,38E+18	FALSO	Low	IsMasterPr	AAEL00561	3:20944241	51	2	FALSO	1
2,34E+17	FALSO	Low	IsMasterPr	AAEL02135	3:31954378	51	2	FALSO	1
-4,46E+18	FALSO	Low	IsMasterPr	AAEL02755	2:31452029	51	3	FALSO	1
-6,59E+17	FALSO	Low	IsMasterPr	AAEL01997	3:17478788	51	1	FALSO	1
-1,13E+18	FALSO	Low	IsMasterPr	AAEL02601	1:66871400	51	1	FALSO	1
8,52E+18	FALSO	Low	IsMasterPr	AAEL0091C	1:23412631	51	18	FALSO	1
-3,04E+17	FALSO	Low	IsMasterPr	AAEL00903	2:10140490	51	6	FALSO	1
-7,24E+18	FALSO	Low	IsMasterPr	AAEL00452	1:64391728	51	3	FALSO	1
-9,02E+17	FALSO	Low	IsMasterPr	A0A3B0IV2	Bifunctiona	51	6	FALSO	1
4,32E+18	FALSO	Low	IsMasterPr	AAEL02678	2:40098040	51	4	FALSO	1
-4,16E+18	FALSO	Low	IsMasterPr	AAEL00541	1:24716151	51	5	FALSO	1
8,16E+18	FALSO	Low	IsMasterPr	AAEL02699	3:33401451	51	2	FALSO	1
-1,68E+18	FALSO	Low	IsMasterPr	AAEL00365	2:31676230	51	6	FALSO	1
8,10E+18	FALSO	Low	IsMasterPr	AAEL0029C	1:10802225	52	2	FALSO	1
1,65E+18	FALSO	Low	IsMasterPr	AAEL00418	2:14629478	52	3	FALSO	1
-9,21E+18	FALSO	Low	IsMasterPr	AAEL01822	3:38117051	52	2	FALSO	1
-1,82E+18	FALSO	Low	IsMasterPr	AAEL00795	2:35160740	52	7	FALSO	1
-4,86E+18	FALSO	Low	IsMasterPr	A0A2A4IIQ	Ketol-acid r	52	6	FALSO	1
-5,60E+18	FALSO	Low	IsMasterPr	A0A5C0YSC	ANK_REP_I	52	6	FALSO	1
-7,44E+18	FALSO	Low	IsMasterPr	A0A176Q6I	Pyruvate, p	52	4	FALSO	1
2,26E+18	FALSO	Low	IsMasterPr	A0A225X4C	Proton-trar	52	5	FALSO	1
-8,54E+18	FALSO	Low	IsMasterPr	AAEL01202	3:14729769	52	9	FALSO	1
-6,16E+17	FALSO	Low	IsMasterPr	AAEL00894	1:80905481	52	4	FALSO	1
-1,78E+17	FALSO	Low	IsMasterPr	A0A178GW	Uncharacte	52	5	FALSO	1
-3,77E+18	FALSO	Low	IsMasterPr	M9WS76	DNA polym	53	3	FALSO	1
4,68E+18	FALSO	Low	IsMasterPr	AAEL00527	1:31203321	53	3	FALSO	1
5,26E+18	FALSO	Low	IsMasterPr	AAEL01463	1:24214620	53	7	FALSO	1
2,24E+18	FALSO	Low	IsMasterPr	AAEL01174	3:29069084	53	5	FALSO	1
-8,49E+18	FALSO	Low	IsMasterPr	AAEL02076	2:34843641	53	2	FALSO	1
6,89E+17	FALSO	Low	IsMasterPr	AAEL00688	2:25615481	53	5	FALSO	1
-3,64E+18	FALSO	Low	IsMasterPr	A0A218KNI	Glutamate	53	3	FALSO	1
-6,81E+18	FALSO	Low	IsMasterPr	AAEL00719	2:31802195	53	2	FALSO	1
6,28E+18	FALSO	Low	IsMasterPr	AAEL01457	2:45225191	53	3	FALSO	1
3,16E+17	FALSO	Low	IsMasterPr	Q73HN6	Uncharacte	53	1	FALSO	1
5,06E+18	FALSO	Low	IsMasterPr	AAEL00432	2:38800344	53	7	FALSO	1
7,91E+17	FALSO	Low	IsMasterPr	AAEL00847	2:19118671	53	1	FALSO	1
-9,07E+18	FALSO	Low	IsMasterPr	A5H0C1	Uroporphy	53	10	FALSO	1

-7,49E+18	FALSO	Low	IsMasterPr	AAEL00587	NIGP0100C	53	4	FALSO	1
7,28E+18	FALSO	Low	IsMasterPr	AAEL01404	2:2399990	53	2	FALSO	1
-1,36E+18	FALSO	Low	IsMasterPr	AAEL00305	2:2126302	53	4	FALSO	1
-7,68E+18	FALSO	Low	IsMasterPr	AAEL00411	3:4017659	53	3	FALSO	1
8,03E+18	FALSO	Low	IsMasterPr	AAEL01353	2:2461753	53	9	FALSO	1
3,81E+18	FALSO	Low	IsMasterPr	I7IU10	Peptidylprc	53	5	FALSO	1
-5,15E+18	FALSO	Low	IsMasterPr	AAEL00515	2:1726898	53	2	FALSO	1
7,27E+18	FALSO	Low	IsMasterPr	Q5GS82	Trigger fact	53	6	FALSO	1
-6,96E+18	FALSO	Low	IsMasterPr	AAEL01203	1:2092127	53	2	FALSO	1
-6,46E+18	FALSO	Low	IsMasterPr	AAEL00998	1:1774304	53	5	FALSO	1
-3,65E+18	FALSO	Low	IsMasterPr	AAEL00867	2:2636383	53	3	FALSO	1
7,95E+18	FALSO	Low	IsMasterPr	A0A2A4IGF	UDP-N-ace	53	8	FALSO	1
1,28E+18	FALSO	Low	IsMasterPr	AAEL01557	1:1941760	54	22	FALSO	1
-5,30E+18	FALSO	Low	IsMasterPr	AAEL01133	3:3534960	54	3	FALSO	1
5,50E+18	FALSO	Low	IsMasterPr	AAEL0001S	3:2159551	54	6	FALSO	1
3,97E+18	FALSO	Low	IsMasterPr	AAEL02352	2:1495914	54	5	FALSO	1
-8,79E+18	FALSO	Low	IsMasterPr	AAEL02503	2:1688165	55	9	FALSO	1
7,61E+18	FALSO	Low	IsMasterPr	AAEL00824	3:6487872	55	2	FALSO	1
5,76E+18	FALSO	Low	IsMasterPr	AAEL01262	1:2794276	55	2	FALSO	1
-8,79E+18	FALSO	Low	IsMasterPr	AAEL01136	2:2662447	55	3	FALSO	1
-1,81E+18	FALSO	Low	IsMasterPr	AAEL0120S	2:1396989	55	3	FALSO	1
-1,13E+18	FALSO	Low	IsMasterPr	A0A218KNI	Uncharacte	55	14	FALSO	1
-6,97E+18	FALSO	Low	IsMasterPr	A0A059IW.	Cytochrom	55	5	FALSO	1
-3,37E+18	FALSO	Low	IsMasterPr	I7IAD5	ATP-depen	55	4	FALSO	1
4,91E+18	FALSO	Low	IsMasterPr	A0A4S2QSI	Ankyrin rep	55	8	FALSO	1
-8,42E+18	FALSO	Low	IsMasterPr	A0A075CK	DUF28 pro	55	28	FALSO	1
7,96E+18	FALSO	Low	IsMasterPr	AAEL02622	1:5750522	55	1	FALSO	1
-8,81E+18	FALSO	Low	IsMasterPr	A0A0U1D9	Ankyrin rep	55	3	FALSO	1
-6,18E+18	FALSO	Low	IsMasterPr	AAEL02766	2:8986979	55	2	FALSO	1
2,04E+18	FALSO	Low	IsMasterPr	A0A2A4IHV	Sugar ABC	55	10	FALSO	1
5,95E+18	FALSO	Low	IsMasterPr	A0A218KQ	Uncharacte	55	8	FALSO	1
-3,60E+18	FALSO	Low	IsMasterPr	AAEL02001	2:1964596	55	4	FALSO	1
-1,00E+18	FALSO	Low	IsMasterPr	AAEL02188	2:2479367	55	4	FALSO	1
-4,29E+18	FALSO	Low	IsMasterPr	B6Y911	Cytochrom	55	15	FALSO	1
6,21E+18	FALSO	Low	IsMasterPr	AAEL00021	3:2162212	56	7	FALSO	1
-8,80E+18	FALSO	Low	IsMasterPr	AAEL02041	2:1557739	56	4	FALSO	1
-5,86E+18	FALSO	Low	IsMasterPr	A0A060PXI	Ankyrin an	57	5	FALSO	1
1,88E+18	FALSO	Low	IsMasterPr	AAEL00625	3:6290717	57	2	FALSO	1
-5,27E+18	FALSO	Low	IsMasterPr	AAEL00458	2:3387502	57	2	FALSO	1
-5,44E+18	FALSO	Low	IsMasterPr	A0A2A4IIF1	Uncharacte	57	6	FALSO	1
-4,36E+18	FALSO	Low	IsMasterPr	AAEL0199S	1:6939343	57	2	FALSO	1
1,83E+18	FALSO	Low	IsMasterPr	AAEL00505	1:2954745	57	4	FALSO	1
2,42E+18	FALSO	Low	IsMasterPr	AAEL02711	3:2917179	58	2	FALSO	1
-4,22E+18	FALSO	Low	IsMasterPr	AAEL00326	1:2911495	58	2	FALSO	1
-1,09E+18	FALSO	Low	IsMasterPr	AAEL00154	2:2241004	57	17	FALSO	1
5,81E+18	FALSO	Low	IsMasterPr	AAEL0116C	3:1923925	57	2	FALSO	1
-8,59E+18	FALSO	Low	IsMasterPr	AAEL01988	2:4008099	57	1	FALSO	1
-2,95E+18	FALSO	Low	IsMasterPr	AAEL00141	2:4827102	58	4	FALSO	1
-3,20E+18	FALSO	Low	IsMasterPr	AAEL00737	2:2335643	58	2	FALSO	1
2,60E+17	FALSO	Low	IsMasterPr	AAEL01706	3:1406230	58	8	FALSO	1

-5,37E+18	FALSO	Low	IsMasterPr	AAEL00614	3:69391754	58	10	FALSO	1
2,27E+18	FALSO	Low	IsMasterPr	AAEL01013	2:26472690	59	4	FALSO	1
-2,54E+18	FALSO	Low	IsMasterPr	AAEL02317	2:29090784	59	2	FALSO	1
-2,12E+18	FALSO	Low	IsMasterPr	AAEL02735	2:18248541	59	5	FALSO	1
8,82E+18	FALSO	Low	IsMasterPr	AAEL02657	3:29236007	59	7	FALSO	1
-2,85E+18	FALSO	Low	IsMasterPr	AAEL00520	2:196410:2	59	2	FALSO	1
7,35E+18	FALSO	Low	IsMasterPr	AAEL00765	1:13781664	59	5	FALSO	1
-2,06E+18	FALSO	Low	IsMasterPr	AAEL01965	2:22580331	59	1	FALSO	1
2,87E+18	FALSO	Low	IsMasterPr	A0A369RM	Uncharacte	60	7	FALSO	1
9,03E+18	FALSO	Low	IsMasterPr	A0A2A4IHY	2-nitroprop	60	8	FALSO	1
4,59E+18	FALSO	Low	IsMasterPr	A0A218KM	Bifunctiona	60	7	FALSO	1
1,61E+18	FALSO	Low	IsMasterPr	AAEL00096	3:37997891	60	3	FALSO	1
5,24E+18	FALSO	Low	IsMasterPr	AAEL00792	3:29543884	60	3	FALSO	1
-8,04E+18	FALSO	Low	IsMasterPr	AAEL01111	3:37881294	60	6	FALSO	1
3,29E+18	FALSO	Low	IsMasterPr	AAEL01258	2:32803945	60	4	FALSO	1
9,10E+18	FALSO	Low	IsMasterPr	AAEL00784	2:46042699	60	3	FALSO	1
-1,23E+18	FALSO	Low	IsMasterPr	Q2TPL2	60 kDa cha	60	11	FALSO	1
-2,79E+18	FALSO	Low	IsMasterPr	A0A3D1L4Y	Periplasmic	60	5	FALSO	1
1,84E+18	FALSO	Low	IsMasterPr	AAEL00857	3:22249461	60	3	FALSO	1
8,45E+18	FALSO	Low	IsMasterPr	AAEL02015	2:36062400	60	14	FALSO	1
6,79E+18	FALSO	Low	IsMasterPr	AAEL02209	2:46483048	60	1	FALSO	1
7,95E+18	FALSO	Low	IsMasterPr	AAEL00879	3:13338131	60	3	FALSO	1
-5,85E+18	FALSO	Low	IsMasterPr	AAEL00082	3:36393471	60	4	FALSO	1
-6,71E+18	FALSO	Low	IsMasterPr	A0A225X8F	Aspartate--	60	5	FALSO	1
6,91E+18	FALSO	Low	IsMasterPr	A0A225X6T	RNA polym	60	4	FALSO	1
5,87E+18	FALSO	Low	IsMasterPr	AAEL01483	2:15275191	60	4	FALSO	1
3,76E+18	FALSO	Low	IsMasterPr	AAEL00207	1:58306255	60	5	FALSO	1
5,37E+18	FALSO	Low	IsMasterPr	B9A927	F protein O	60	21	FALSO	1
1,28E+18	FALSO	Low	IsMasterPr	AAEL01805	1:88487341	60	2	FALSO	1
-4,22E+18	FALSO	Low	IsMasterPr	AAEL02738	3:36582909	60	7	FALSO	1
5,66E+17	FALSO	Low	IsMasterPr	AAEL00012	1:17039414	60	3	FALSO	1
3,03E+18	FALSO	Low	IsMasterPr	AAEL02048	1:28697429	60	7	FALSO	1
-3,91E+18	FALSO	Low	IsMasterPr	AAEL00146	1:11185090	60	12	FALSO	1
4,18E+18	FALSO	Low	IsMasterPr	AAEL01962	3:34288150	60	2	FALSO	1
1,25E+18	FALSO	Low	IsMasterPr	AAEL00976	1:41999351	61	3	FALSO	1
-7,53E+18	FALSO	Low	IsMasterPr	AAEL00688	2:25581240	62	1	FALSO	1
-4,99E+18	FALSO	Low	IsMasterPr	A0A5C0YLC	MFS transp	63	7	FALSO	1
3,45E+18	FALSO	Low	IsMasterPr	AAEL00154	2:20663878	63	1	FALSO	1
5,24E+18	FALSO	Low	IsMasterPr	AAEL02797	3:29182738	63	1	FALSO	1
-5,71E+18	FALSO	Low	IsMasterPr	AAEL00498	2:28182247	63	4	FALSO	1
8,14E+18	FALSO	Low	IsMasterPr	AAEL00674	2:83746241	63	2	FALSO	1
-4,24E+17	FALSO	Low	IsMasterPr	AAEL00595	1:25825621	63	3	FALSO	1
7,27E+18	FALSO	Low	IsMasterPr	AAEL00641	2:11288990	63	6	FALSO	1
6,48E+18	FALSO	Low	IsMasterPr	AAEL02713	3:18021440	64	4	FALSO	1
7,22E+18	FALSO	Low	IsMasterPr	AAEL00286	1:25607095	65	4	FALSO	1
-4,03E+17	FALSO	Low	IsMasterPr	AAEL00133	3:47647180	65	4	FALSO	1
-6,57E+17	FALSO	Low	IsMasterPr	AAEL02183	3:32713185	65	1	FALSO	1
-2,86E+18	FALSO	Low	IsMasterPr	A0A369RJ3	Alanine--tR	65	3	FALSO	1
8,68E+18	FALSO	Low	IsMasterPr	A0A225X66	Uncharacte	66	8	FALSO	1
-6,07E+18	FALSO	Low	IsMasterPr	AAEL00353	2:25621519	66	3	FALSO	1

-2,71E+18	FALSO	Low	IsMasterPr	AAEL00611	3:2597770	66	6	FALSO	1
6,48E+18	FALSO	Low	IsMasterPr	AAEL0141C	2:3830496	66	7	FALSO	1
-4,67E+18	FALSO	Low	IsMasterPr	AAEL00601	2:4723743	66	1	FALSO	1
4,44E+18	FALSO	Low	IsMasterPr	AAEL0077E	2:2192615	66	4	FALSO	1
4,00E+18	FALSO	Low	IsMasterPr	AAEL00815	3:9631880	66	5	FALSO	1
-4,03E+17	FALSO	Low	IsMasterPr	A0A3D1L3I	UvrABC sys	67	5	FALSO	1
-2,96E+18	FALSO	Low	IsMasterPr	A0A369RLI	Type III eff	68	6	FALSO	1
2,43E+18	FALSO	Low	IsMasterPr	AAEL00695	1:1573519	69	1	FALSO	1
-3,63E+18	FALSO	Low	IsMasterPr	AAEL0078C	3:1519094	70	3	FALSO	1
3,56E+18	FALSO	Low	IsMasterPr	AAEL02082	NIGP01001	71	4	FALSO	1
8,35E+18	FALSO	Low	IsMasterPr	AAEL00551	1:1503738	71	3	FALSO	1
-5,49E+18	FALSO	Low	IsMasterPr	AAEL0238E	1:2763185	71	1	FALSO	1
5,29E+18	FALSO	Low	IsMasterPr	AAEL01237	2:1275400	71	2	FALSO	1
-5,63E+18	FALSO	Low	IsMasterPr	AAEL01043	3:6761196	71	3	FALSO	1
-3,10E+17	FALSO	Low	IsMasterPr	AAEL02653	3:1732013	71	1	FALSO	1
4,27E+18	FALSO	Low	IsMasterPr	AAEL00142	2:4767847	71	8	FALSO	1
-1,88E+18	FALSO	Low	IsMasterPr	AAEL0080C	2:3700721	71	4	FALSO	1
-8,01E+18	FALSO	Low	IsMasterPr	A0A3B0JH6	tRNA nucle	71	1	FALSO	1
-3,64E+18	FALSO	Low	IsMasterPr	A0A4S2QQ	Uncharacte	73	8	FALSO	1
6,09E+17	FALSO	Low	IsMasterPr	AAEL00277	3:1292672	73	5	FALSO	1
1,38E+18	FALSO	Low	IsMasterPr	A0A176Q2	GTP cycloh	73	8	FALSO	1
-1,03E+18	FALSO	Low	IsMasterPr	AAEL0257E	3:2307094	74	1	FALSO	1
-1,18E+18	FALSO	Low	IsMasterPr	A0A218KQ	Inositol mo	74	9	FALSO	1
-8,17E+17	FALSO	Low	IsMasterPr	I7ITN7	DNA segreg	74	4	FALSO	1
-4,09E+18	FALSO	Low	IsMasterPr	AAEL00071	2:4597459	74	1	FALSO	1
-2,61E+18	FALSO	Low	IsMasterPr	AAEL0019S	2:4246232	75	6	FALSO	1
-4,21E+18	FALSO	Low	IsMasterPr	AAEL0023C	3:3742131	75	0	FALSO	1
-2,08E+18	FALSO	Low	IsMasterPr	A0A4Y6UP	Uncharacte	75	6	FALSO	1
-7,83E+18	FALSO	Low	IsMasterPr	A0A218KM	Ribonucleo	75	5	FALSO	1
7,94E+18	FALSO	Low	IsMasterPr	AAEL0100E	2:2004948	75	11	FALSO	1
1,08E+18	FALSO	Low	IsMasterPr	AAEL00142	2:4558981	76	8	FALSO	1
1,58E+18	FALSO	Low	IsMasterPr	A0A5C0YM	Uncharacte	76	23	FALSO	1
1,07E+18	FALSO	Low	IsMasterPr	A0A369RKI	1-deoxy-D-	76	8	FALSO	1
-3,77E+18	FALSO	Low	IsMasterPr	AAEL01244	1:2134511	77	0	FALSO	1
5,21E+18	FALSO	Low	IsMasterPr	AAEL0038E	2:3159503	77	5	FALSO	1
4,07E+18	FALSO	Low	IsMasterPr	AAEL02903	2:3167678	77	1	FALSO	1
7,37E+18	FALSO	Low	IsMasterPr	AAEL00072	2:5328888	77	7	FALSO	1
1,10E+17	FALSO	Low	IsMasterPr	AAEL0085E	3:1981237	77	3	FALSO	1
4,63E+18	FALSO	Low	IsMasterPr	Q5GS81	Predicted li	77	15	FALSO	1
-2,09E+18	FALSO	Low	IsMasterPr	A0A1E7QKI	Signal reco	77	10	FALSO	1
6,26E+18	FALSO	Low	IsMasterPr	A0A2A4IIQ	Phage port	77	7	FALSO	1
-2,43E+18	FALSO	Low	IsMasterPr	AAEL01437	3:4458911	77	2	FALSO	1
-7,57E+18	FALSO	Low	IsMasterPr	A0A1V2N4	UvrABC sys	78	2	FALSO	1
2,24E+18	FALSO	Low	IsMasterPr	AAEL0038C	1:4517227	78	9	FALSO	1
-2,48E+18	FALSO	Low	IsMasterPr	AAEL01992	3:3422707	78	2	FALSO	1
-6,58E+18	FALSO	Low	IsMasterPr	AAEL0061E	1:2246681	78	6	FALSO	1
-6,80E+18	FALSO	Low	IsMasterPr	AAEL0214C	2:2729390	78	4	FALSO	1
-2,18E+18	FALSO	Low	IsMasterPr	AAEL01341	2:9014398	78	2	FALSO	1
-5,35E+18	FALSO	Low	IsMasterPr	AAEL0037E	1:4395290	79	5	FALSO	1
-4,04E+18	FALSO	Low	IsMasterPr	AAEL0057C	3:1154846	80	7	FALSO	1

2,59E+18	FALSO	Low	IsMasterPr	AAEL00812	3:1076334	80	10	FALSO	1
-7,16E+18	FALSO	Low	IsMasterPr	Q9MCW7	Capsid prot	80	21	FALSO	1
-7,31E+18	FALSO	Low	IsMasterPr	A0A5C0YR	3,4-dihydro	80	13	FALSO	1
-6,06E+18	FALSO	Low	IsMasterPr	A0A0U1D2	Cation ABC	79	11	FALSO	1
-7,85E+18	FALSO	Low	IsMasterPr	A0A218KPI	Uncharacte	79	11	FALSO	1
-6,27E+18	FALSO	Low	IsMasterPr	A0A225X78	30S riboson	79	24	FALSO	1
7,90E+18	FALSO	Low	IsMasterPr	A0A060PW	Phosphorib	79	10	FALSO	1
7,70E+18	FALSO	Low	IsMasterPr	AAEL01363	2:3344617	80	4	FALSO	1
-2,63E+18	FALSO	Low	IsMasterPr	AAEL01007	3:2091333	80	2	FALSO	1
-2,79E+17	FALSO	Low	IsMasterPr	AAEL00719	2:3185187	79	7	FALSO	1
-7,71E+18	FALSO	Low	IsMasterPr	AAEL02173	3:3658571	80	1	FALSO	1
-3,71E+18	FALSO	Low	IsMasterPr	AAEL00397	3:2682533	80	2	FALSO	1
-3,06E+18	FALSO	Low	IsMasterPr	A0A369RL3	Uncharacte	80	25	FALSO	1
6,49E+18	FALSO	Low	IsMasterPr	A0A2A4IJZ	Peptidylpro	80	8	FALSO	1
4,63E+18	FALSO	Low	IsMasterPr	A0A1E7QIY	Glycine--tR	80	3	FALSO	1
-4,47E+18	FALSO	Low	IsMasterPr	V9NZK1	Cell divisio	80	18	FALSO	1
-4,46E+18	FALSO	Low	IsMasterPr	AAEL00113	3:1367575	81	6	FALSO	1
-4,79E+18	FALSO	Low	IsMasterPr	AAEL01514	2:1113023	81	2	FALSO	1
8,30E+18	FALSO	Low	IsMasterPr	I7IAC6	Queuine tR	81	8	FALSO	1
6,78E+18	FALSO	Low	IsMasterPr	A0A1E7QK	Glutamate-	81	4	FALSO	1
-4,67E+18	FALSO	Low	IsMasterPr	B6Y741	Glutamyl-t	81	8	FALSO	1
4,71E+18	FALSO	Low	IsMasterPr	G9I5G0	Outer surfa	81	13	FALSO	1
6,30E+18	FALSO	Low	IsMasterPr	A0A4S2QTI	Phenylalan	81	3	FALSO	1
-6,49E+18	FALSO	Low	IsMasterPr	AAEL00573	2:4375857	81	2	FALSO	1
8,06E+18	FALSO	Low	IsMasterPr	A0A3D1L4	Uncharacte	81	9	FALSO	1
9,39E+17	FALSO	Low	IsMasterPr	AAEL00699	3:1714330	83	4	FALSO	1
-5,14E+18	FALSO	Low	IsMasterPr	AAEL0028C	1:3047257	83	4	FALSO	1
-6,33E+17	FALSO	Low	IsMasterPr	A0A218KN	Phosphorib	83	10	FALSO	1
1,01E+18	FALSO	Low	IsMasterPr	Q73HS8	Uncharacte	83	5	FALSO	1
-4,14E+18	FALSO	Low	IsMasterPr	AAEL02034	3:6882361	83	4	FALSO	1
-7,81E+18	FALSO	Low	IsMasterPr	AAEL00489	2:7110100	83	3	FALSO	1
-1,48E+18	FALSO	Low	IsMasterPr	AAEL01974	2:1155348	83	1	FALSO	1
-8,38E+18	FALSO	Low	IsMasterPr	AAEL0272C	1:2106299	84	4	FALSO	1
6,46E+17	FALSO	Low	IsMasterPr	AAEL02609	2:1265063	84	1	FALSO	1
-3,03E+18	FALSO	Low	IsMasterPr	A0A0U1D5	Ankyrin rep	84	2	FALSO	1
-2,72E+18	FALSO	Low	IsMasterPr	AAEL00048	3:1628063	84	3	FALSO	1
-5,66E+18	FALSO	Low	IsMasterPr	A0A3B0IW	Uncharacte	84	6	FALSO	1
7,33E+18	FALSO	Low	IsMasterPr	A0A5C4TN	Threonylca	84	15	FALSO	1
-6,03E+18	FALSO	Low	IsMasterPr	AAEL0030C	3:3881471	84	3	FALSO	1
-3,23E+18	FALSO	Low	IsMasterPr	A0A5C4TP	Uncharacte	84	2	FALSO	1
-7,26E+18	FALSO	Low	IsMasterPr	AAEL00162	1:1380031	84	9	FALSO	1
5,28E+17	FALSO	Low	IsMasterPr	AAEL02556	1:1620129	84	2	FALSO	1
8,43E+18	FALSO	Low	IsMasterPr	A0A178GS	Uncharacte	84	5	FALSO	1
4,32E+18	FALSO	Low	IsMasterPr	AAEL02494	1:2916479	84	6	FALSO	1
3,71E+18	FALSO	Low	IsMasterPr	AAEL00736	3:1229033	84	1	FALSO	1
-6,34E+18	FALSO	Low	IsMasterPr	AAEL00656	2:5932829	84	9	FALSO	1
-6,12E+18	FALSO	Low	IsMasterPr	AAEL00581	1:2012897	85	2	FALSO	1
-8,53E+18	FALSO	Low	IsMasterPr	A0A098AS	Penicillin-b	86	6	FALSO	1
4,17E+18	FALSO	Low	IsMasterPr	A0A2P1L4	Cell divisio	86	19	FALSO	1
-4,76E+18	FALSO	Low	IsMasterPr	A0A369RL	Putative tr	87	8	FALSO	1

7,38E+18	FALSO	Low	IsMasterPr	A0A2A4IIW	Ribosome r	87	17	FALSO	1
-2,54E+18	FALSO	Low	IsMasterPr	AAEL01711	3:2215805:	86	8	FALSO	1
-6,81E+17	FALSO	Low	IsMasterPr	AAEL0078S	1:6340274:	86	2	FALSO	1
-3,29E+18	FALSO	Low	IsMasterPr	AAEL00442	3:2057904	86	5	FALSO	1
5,17E+18	FALSO	Low	IsMasterPr	A0A218KM	Glutamate-	86	8	FALSO	1
-3,98E+18	FALSO	Low	IsMasterPr	A0A5B9JW	Outer mem	86	18	FALSO	1
4,28E+18	FALSO	Low	IsMasterPr	AAEL01185	2:2879114	86	4	FALSO	1
6,87E+18	FALSO	Low	IsMasterPr	AAEL01353	2:2463111	86	3	FALSO	1
7,80E+18	FALSO	Low	IsMasterPr	AAEL01997	3:3637727	86	2	FALSO	1
2,33E+18	FALSO	Low	IsMasterPr	AAEL0111C	2:2318677	86	3	FALSO	1
5,45E+17	FALSO	Low	IsMasterPr	AAEL02524	3:2367507	86	2	FALSO	1
1,90E+18	FALSO	Low	IsMasterPr	AAEL00237	2:3046424	86	2	FALSO	1
2,02E+18	FALSO	Low	IsMasterPr	AAEL01348	2:2368008	86	1	FALSO	1
1,86E+18	FALSO	Low	IsMasterPr	AAEL01075	3:2942153	86	5	FALSO	1
-4,67E+18	FALSO	Low	IsMasterPr	AAEL00103	3:5549072	86	6	FALSO	1
-8,99E+18	FALSO	Low	IsMasterPr	AAEL0135C	2:1896676	86	2	FALSO	1
6,13E+18	FALSO	Low	IsMasterPr	AAEL0098S	3:1221999	86	4	FALSO	1
-6,90E+18	FALSO	Low	IsMasterPr	AAEL00547	3:3830693	86	2	FALSO	1
8,87E+18	FALSO	Low	IsMasterPr	AAEL02141	2:1514064	86	0	FALSO	1
-6,29E+18	FALSO	Low	IsMasterPr	AAEL0083S	3:2244604	86	2	FALSO	1
-7,38E+18	FALSO	Low	IsMasterPr	AAEL00237	2:3039968	86	6	FALSO	1
-1,30E+18	FALSO	Low	IsMasterPr	AAEL00778	3:1336971	86	5	FALSO	1
-8,67E+18	FALSO	Low	IsMasterPr	AAEL00173	2:4418431	86	5	FALSO	1
2,74E+18	FALSO	Low	IsMasterPr	AAEL00295	1:2613847	86	3	FALSO	1
5,98E+17	FALSO	Low	IsMasterPr	AAEL02132	2:1218668	86	0	FALSO	1
-3,00E+18	FALSO	Low	IsMasterPr	AAEL00085	3:3322563	86	2	FALSO	1
6,95E+17	FALSO	Low	IsMasterPr	AAEL0013S	2:4571493	86	2	FALSO	1
-8,31E+18	FALSO	Low	IsMasterPr	AAEL01257	1:2852272	86	13	FALSO	1
-8,63E+18	FALSO	Low	IsMasterPr	AAEL00937	3:8537269	85	2	FALSO	1
2,12E+18	FALSO	Low	IsMasterPr	AAEL02012	3:3119397	85	12	FALSO	1
-3,97E+18	FALSO	Low	IsMasterPr	AAEL00353	2:2562031	85	2	FALSO	1
8,45E+18	FALSO	Low	IsMasterPr	AAEL01707	2:1829312	85	3	FALSO	1
-7,73E+18	FALSO	Low	IsMasterPr	AAEL00443	3:2044294	85	8	FALSO	1
3,76E+18	FALSO	Low	IsMasterPr	AAEL0029S	1:2089617	85	6	FALSO	1
-5,89E+18	FALSO	Low	IsMasterPr	AAEL02514	1:3060004	85	2	FALSO	1
-2,10E+18	FALSO	Low	IsMasterPr	AAEL01304	3:3109228	85	3	FALSO	1
5,02E+18	FALSO	Low	IsMasterPr	AAEL01983	3:1577157	85	1	FALSO	1
-7,49E+18	FALSO	Low	IsMasterPr	AAEL01827	2:1211415	85	1	FALSO	1
5,59E+18	FALSO	Low	IsMasterPr	AAEL00963	1:2227426	85	2	FALSO	1
3,44E+18	FALSO	Low	IsMasterPr	AAEL0048C	3:2734500	85	4	FALSO	1
2,42E+18	FALSO	Low	IsMasterPr	AAEL00664	3:2005573	85	3	FALSO	1
5,94E+18	FALSO	Low	IsMasterPr	AAEL0085C	2:9922133	85	2	FALSO	1
4,15E+18	FALSO	Low	IsMasterPr	AAEL01017	3:1490820	85	5	FALSO	1
4,95E+18	FALSO	Low	IsMasterPr	AAEL00373	2:1960419	86	2	FALSO	1
-7,95E+18	FALSO	Low	IsMasterPr	Q4ECC2	Ankyrin rep	86	1	FALSO	1
-3,54E+18	FALSO	Low	IsMasterPr	AAEL0087S	3:1327323	86	6	FALSO	1
3,45E+18	FALSO	Low	IsMasterPr	AAEL00404	3:1669425	86	4	FALSO	1
7,78E+18	FALSO	Low	IsMasterPr	AAEL02463	2:1182120	86	8	FALSO	1
-4,37E+18	FALSO	Low	IsMasterPr	AAEL0080E	2:2303789	85	6	FALSO	1
1,20E+18	FALSO	Low	IsMasterPr	Q73IA5	Uncharacte	85	12	FALSO	1

-2,71E+18	FALSO	Low	IsMasterPr	Q4EB50	Putative re	86	4	FALSO	1
1,23E+18	FALSO	Low	IsMasterPr	AAEL02546	3:1384517	86	1	FALSO	1
8,51E+18	FALSO	Low	IsMasterPr	AAEL00412	3:1542794	85	2	FALSO	1
1,03E+18	FALSO	Low	IsMasterPr	AAEL00174	2:3778328	85	2	FALSO	1
1,72E+18	FALSO	Low	IsMasterPr	AAEL02364	3:3484168	85	2	FALSO	1
-4,36E+18	FALSO	Low	IsMasterPr	AAEL00635	3:3968375	85	4	FALSO	1
-8,04E+18	FALSO	Low	IsMasterPr	AAEL02776	NIGP0100C	85	3	FALSO	1
-6,89E+18	FALSO	Low	IsMasterPr	AAEL00766	1:4929297	85	2	FALSO	1
4,32E+18	FALSO	Low	IsMasterPr	AAEL01109	2:2322754	85	2	FALSO	1
-5,62E+18	FALSO	Low	IsMasterPr	AAEL00635	3:3977607	85	5	FALSO	1
-5,21E+18	FALSO	Low	IsMasterPr	AAEL02684	2:7897845	85	1	FALSO	1
2,85E+18	FALSO	Low	IsMasterPr	A0A1E7QJN	Uncharacte	85	4	FALSO	1
-9,93E+17	FALSO	Low	IsMasterPr	AAEL00218	2:4295492	85	1	FALSO	1
5,52E+18	FALSO	Low	IsMasterPr	AAEL0085C	2:9938555	85	1	FALSO	1
4,51E+18	FALSO	Low	IsMasterPr	AAEL01461	3:3685155	85	2	FALSO	1
2,83E+18	FALSO	Low	IsMasterPr	A0A060PV	DNA repair	85	9	FALSO	1
7,64E+18	FALSO	Low	IsMasterPr	AAEL01226	2:3843805	85	5	FALSO	1
-1,04E+18	FALSO	Low	IsMasterPr	AAEL02515	NIGP01002	85	1	FALSO	1
6,53E+18	FALSO	Low	IsMasterPr	AAEL00393	2:3544893	85	3	FALSO	1
-1,42E+18	FALSO	Low	IsMasterPr	AAEL01352	1:1440557	85	3	FALSO	1
1,09E+18	FALSO	Low	IsMasterPr	AAEL00905	2:2693335	85	1	FALSO	1
9,07E+18	FALSO	Low	IsMasterPr	AAEL00489	2:7222711	85	2	FALSO	1
-8,34E+18	FALSO	Low	IsMasterPr	AAEL01139	3:2016812	85	5	FALSO	1
-9,04E+18	FALSO	Low	IsMasterPr	A0A225X6I	Protein tra	85	3	FALSO	1
-8,04E+18	FALSO	Low	IsMasterPr	AAEL00596	3:2288472	85	2	FALSO	1
4,15E+17	FALSO	Low	IsMasterPr	AAEL01347	2:1209260	85	1	FALSO	1
-2,30E+18	FALSO	Low	IsMasterPr	AAEL00159	2:2290589	85	2	FALSO	1
-3,94E+18	FALSO	Low	IsMasterPr	A0A060PZ7	Aspartate--	85	5	FALSO	1
6,65E+18	FALSO	Low	IsMasterPr	AAEL02198	2:1627347	85	1	FALSO	1
4,11E+18	FALSO	Low	IsMasterPr	AAEL00249	2:7770385	85	3	FALSO	1
-9,12E+16	FALSO	Low	IsMasterPr	AAEL01203	1:2090631	85	2	FALSO	1
-5,46E+18	FALSO	Low	IsMasterPr	AAEL00431	3:5111464	85	3	FALSO	1
7,35E+18	FALSO	Low	IsMasterPr	AAEL01195	2:2178242	85	1	FALSO	1
-7,11E+18	FALSO	Low	IsMasterPr	AAEL01128	2:2267802	85	7	FALSO	1
-2,66E+18	FALSO	Low	IsMasterPr	AAEL00646	3:2165166	85	4	FALSO	1
-4,22E+18	FALSO	Low	IsMasterPr	AAEL01034	2:4039562	85	6	FALSO	1
-1,67E+18	FALSO	Low	IsMasterPr	AAEL01372	3:2542870	85	0	FALSO	1
-1,04E+17	FALSO	Low	IsMasterPr	A0A060Q4	Protein-exp	85	5	FALSO	1
2,15E+18	FALSO	Low	IsMasterPr	AAEL00688	2:2530567	85	1	FALSO	1
-8,00E+18	FALSO	Low	IsMasterPr	AAEL00613	3:6874343	85	9	FALSO	1
-1,14E+18	FALSO	Low	IsMasterPr	AAEL02151	NIGP01001	85	3	FALSO	1
-3,17E+18	FALSO	Low	IsMasterPr	AAEL02573	2:4019978	85	2	FALSO	1
-6,13E+18	FALSO	Low	IsMasterPr	A0A225X5	ABC transp	86	8	FALSO	1
5,04E+18	FALSO	Low	IsMasterPr	AAEL01953	2:1374996	86	2	FALSO	1
3,64E+18	FALSO	Low	IsMasterPr	A0A2A4IH2	3-oxoacyl-[	86	6	FALSO	1
-1,16E+18	FALSO	Low	IsMasterPr	AAEL01563	3:1004446	86	2	FALSO	1
-7,17E+18	FALSO	Low	IsMasterPr	AAEL00338	3:8135293	87	4	FALSO	1
-5,50E+18	FALSO	Low	IsMasterPr	AAEL00471	2:3781200	87	1	FALSO	1
-4,90E+18	FALSO	Low	IsMasterPr	AAEL0059C	3:1739807	87	10	FALSO	1
-4,14E+18	FALSO	Low	IsMasterPr	AAEL02734	3:2497713	87	3	FALSO	1

2,50E+18	FALSO	Low	IsMasterPr	AAEL0245C	1:3070402	87	1	FALSO	1
-5,78E+18	FALSO	Low	IsMasterPr	AAEL01868	MT:9798:1	87	12	FALSO	1
-8,28E+18	FALSO	Low	IsMasterPr	AAEL00661	2:4641878	87	8	FALSO	1
1,35E+18	FALSO	Low	IsMasterPr	AAEL00603	3:3147549	87	1	FALSO	1
-1,61E+18	FALSO	Low	IsMasterPr	AAEL00185	2:2765933	87	2	FALSO	1
7,33E+18	FALSO	Low	IsMasterPr	I7IAK0	50S ribosor	87	21	FALSO	1
6,29E+18	FALSO	Low	IsMasterPr	A0A369RM	Fe(3+) ions	87	8	FALSO	1
-7,52E+18	FALSO	Low	IsMasterPr	AAEL0077C	2:4702826	88	3	FALSO	1
-8,30E+18	FALSO	Low	IsMasterPr	AAEL02592	1:2935822	88	4	FALSO	1
-3,96E+18	FALSO	Low	IsMasterPr	AAEL01014	2:2648140	88	0	FALSO	1
-9,11E+18	FALSO	Low	IsMasterPr	A0A178GSI	Bacteriofer	87	15	FALSO	1
2,92E+18	FALSO	Low	IsMasterPr	AAEL00605	3:7367543	87	2	FALSO	1
-6,48E+18	FALSO	Low	IsMasterPr	A0A5C4TM	Inositol mo	87	7	FALSO	1
2,23E+18	FALSO	Low	IsMasterPr	AAEL01712	2:3885584	88	2	FALSO	1
-6,19E+17	FALSO	Low	IsMasterPr	AAEL0048E	2:1156368	88	9	FALSO	1
-4,53E+18	FALSO	Low	IsMasterPr	AAEL01257	1:2853176	88	4	FALSO	1
-2,49E+18	FALSO	Low	IsMasterPr	A0A3B0JI4	Cytochrom	87	3	FALSO	1
7,10E+18	FALSO	Low	IsMasterPr	AAEL00111	3:4063323	88	4	FALSO	1
1,56E+18	FALSO	Low	IsMasterPr	I7JEM6	Uncharacte	88	8	FALSO	1
3,50E+18	FALSO	Low	IsMasterPr	A0A218KP\	Phage tail t	88	3	FALSO	1
-8,95E+18	FALSO	Low	IsMasterPr	AAEL01302	1:1993967	90	3	FALSO	1
1,74E+18	FALSO	Low	IsMasterPr	B5L360	Outer surfa	90	7	FALSO	1
4,02E+18	FALSO	Low	IsMasterPr	AAEL00747	2:5775187	90	2	FALSO	1
2,08E+18	FALSO	Low	IsMasterPr	AAEL02532	2:5176121	91	2	FALSO	1
-3,41E+18	FALSO	Low	IsMasterPr	AAEL01363	1:6178159	91	5	FALSO	1
1,87E+18	FALSO	Low	IsMasterPr	AAEL0009C	3:3355990	91	13	FALSO	1
-5,71E+18	FALSO	Low	IsMasterPr	AAEL02723	3:2251033	91	2	FALSO	1
7,71E+18	FALSO	Low	IsMasterPr	A0A225X4E	tRNA-dihyc	91	4	FALSO	1
-1,35E+18	FALSO	Low	IsMasterPr	A0A0H3VEI	UDP-glucos	91	4	FALSO	1
3,56E+18	FALSO	Low	IsMasterPr	AAEL0119C	3:3378203	91	1	FALSO	1
-7,02E+18	FALSO	Low	IsMasterPr	AAEL01559	2:6632866	91	1	FALSO	1
-6,54E+18	FALSO	Low	IsMasterPr	AAEL00743	2:1347488	91	20	FALSO	1
-8,96E+17	FALSO	Low	IsMasterPr	AAEL00623	3:2533969	91	1	FALSO	1
7,32E+18	FALSO	Low	IsMasterPr	A0A176Q2	ANK_REP_I	92	2	FALSO	1
6,30E+18	FALSO	Low	IsMasterPr	AAEL00073	2:5475752	92	2	FALSO	1
-2,83E+18	FALSO	Low	IsMasterPr	AAEL00263	3:2567854	92	2	FALSO	1
-2,52E+18	FALSO	Low	IsMasterPr	AAEL00671	2:8512147	92	1	FALSO	1
8,82E+17	FALSO	Low	IsMasterPr	AAEL00618	3:1409893	92	2	FALSO	1
2,53E+18	FALSO	Low	IsMasterPr	AAEL00117	2:3090930	92	3	FALSO	1
-7,96E+18	FALSO	Low	IsMasterPr	AAEL01321	2:1860747	92	2	FALSO	1
1,08E+18	FALSO	Low	IsMasterPr	AAEL00622	3:2463576	92	3	FALSO	1
7,47E+18	FALSO	Low	IsMasterPr	AAEL0198E	2:1232308	92	1	FALSO	1
-8,90E+18	FALSO	Low	IsMasterPr	A0A218KM	Uncharacte	92	2	FALSO	1
-7,71E+18	FALSO	Low	IsMasterPr	A0A3T0GH	6,7-dimeth	92	20	FALSO	1
5,14E+18	FALSO	Low	IsMasterPr	AAEL01527	1:1610854	92	5	FALSO	1
-4,00E+18	FALSO	Low	IsMasterPr	AAEL00713	3:6982161	92	1	FALSO	1
-5,05E+17	FALSO	Low	IsMasterPr	AAEL00423	1:1045995	92	2	FALSO	1
6,14E+18	FALSO	Low	IsMasterPr	AAEL01511	2:3351327	92	3	FALSO	1
4,11E+18	FALSO	Low	IsMasterPr	AAEL02563	3:6971850	92	1	FALSO	1
-3,33E+18	FALSO	Low	IsMasterPr	AAEL00335	2:1127464	92	5	FALSO	1

3,95E+18	FALSO	Low	IsMasterPr	AAEL02074	2:35430927	93	1	FALSO	1
-2,24E+18	FALSO	Low	IsMasterPr	A0A225X41	Glycerol-3-	93	6	FALSO	1
-7,09E+18	FALSO	Low	IsMasterPr	AAEL0006C	2:40879067	93	2	FALSO	1
5,26E+18	FALSO	Low	IsMasterPr	A0A059IVL	MgtE_N do	93	3	FALSO	1
-2,84E+18	FALSO	Low	IsMasterPr	AAEL02222	1:18709121	93	2	FALSO	1
-5,69E+18	FALSO	Low	IsMasterPr	gb:AEN752	ncbiId:AEN	93	0	FALSO	1
-5,44E+18	FALSO	Low	IsMasterPr	AAEL01131	2:33154857	93	2	FALSO	1
-6,73E+18	FALSO	Low	IsMasterPr	A0A1E7QLI	Uncharacte	94	1	FALSO	1
-8,71E+18	FALSO	Low	IsMasterPr	AAEL0075C	1:21635025	94	2	FALSO	1
3,65E+17	FALSO	Low	IsMasterPr	AAEL00443	3:20653370	94	9	FALSO	1
5,08E+18	FALSO	Low	IsMasterPr	AAEL00321	2:36662304	94	6	FALSO	1
8,76E+18	FALSO	Low	IsMasterPr	A0A369RLC	Uncharacte	94	2	FALSO	1
-3,11E+18	FALSO	Low	IsMasterPr	AAEL02043	2:17967884	94	3	FALSO	1
-7,65E+18	FALSO	Low	IsMasterPr	I7IAG5	3-oxoacyl-[	94	6	FALSO	1
5,95E+18	FALSO	Low	IsMasterPr	AAEL02441	1:17743181	95	3	FALSO	1
-3,53E+18	FALSO	Low	IsMasterPr	AAEL00091	3:33252938	95	3	FALSO	1
-8,07E+18	FALSO	Low	IsMasterPr	A0A218KNI	FAD_bindir	95	3	FALSO	1
-5,79E+18	FALSO	Low	IsMasterPr	AAEL00158	2:21613031	95	3	FALSO	1
8,78E+18	FALSO	Low	IsMasterPr	AAEL00553	2:33708160	95	1	FALSO	1
5,96E+18	FALSO	Low	IsMasterPr	AAEL00582	1:22522724	95	2	FALSO	1
-1,73E+18	FALSO	Low	IsMasterPr	A0A3D1L3V	Guanylate I	95	9	FALSO	1
-8,37E+17	FALSO	Low	IsMasterPr	AAEL02112	1:88610360	96	3	FALSO	1
-2,34E+18	FALSO	Low	IsMasterPr	Q5GS94	Uncharacte	96	10	FALSO	1
3,29E+18	FALSO	Low	IsMasterPr	AAEL02372	2:44742710	96	3	FALSO	1
2,18E+18	FALSO	Low	IsMasterPr	A0A2A4IL3	Collar dom	96	3	FALSO	1
6,47E+18	FALSO	Low	IsMasterPr	AAEL0026S	2:14407491	97	4	FALSO	1
-2,04E+18	FALSO	Low	IsMasterPr	AAEL0021C	3:17904451	97	3	FALSO	1
2,78E+18	FALSO	Low	IsMasterPr	AAEL0056S	2:33700980	97	1	FALSO	1
8,61E+18	FALSO	Low	IsMasterPr	AAEL0002C	3:21559710	97	8	FALSO	1
5,81E+18	FALSO	Low	IsMasterPr	AAEL01133	2:92774897	97	2	FALSO	1
-4,59E+18	FALSO	Low	IsMasterPr	AAEL00901	1:29356941	97	5	FALSO	1
7,69E+18	FALSO	Low	IsMasterPr	AAEL00827	3:25045341	97	3	FALSO	1
-4,05E+18	FALSO	Low	IsMasterPr	AAEL01382	1:15849321	98	1	FALSO	1
3,12E+18	FALSO	Low	IsMasterPr	AAEL00654	2:59479341	98	2	FALSO	1
-7,23E+17	FALSO	Low	IsMasterPr	AAEL0240C	2:14569238	98	5	FALSO	1
-8,43E+18	FALSO	Low	IsMasterPr	A0A5F1B7F	Acyl-CoA ca	98	2	FALSO	1
-6,96E+18	FALSO	Low	IsMasterPr	C0R5B0	Glycine--tR	98	2	FALSO	1
3,40E+18	FALSO	Low	IsMasterPr	AAEL01011	2:39626641	98	2	FALSO	1
-2,79E+17	FALSO	Low	IsMasterPr	A0A369RK2	Protein tra	98	2	FALSO	1
6,50E+18	FALSO	Low	IsMasterPr	A0A1V2N3	Oxoglutara	98	2	FALSO	1
2,92E+18	FALSO	Low	IsMasterPr	AAEL02772	3:24193551	99	1	FALSO	1
2,67E+18	FALSO	Low	IsMasterPr	A0A3B0J7D	Chromosor	99	2	FALSO	1
-1,67E+18	FALSO	Low	IsMasterPr	AAEL00614	3:68861890	99	2	FALSO	1
-8,93E+18	FALSO	Low	IsMasterPr	A0A2A4II73	Threonine-	100	3	FALSO	1
5,26E+18	FALSO	Low	IsMasterPr	C0FAC5	GTP-bindin	100	6	FALSO	1
-7,01E+18	FALSO	Low	IsMasterPr	AAEL01956	3:62870945	100	2	FALSO	1
8,99E+18	FALSO	Low	IsMasterPr	AAEL01965	3:16495990	100	3	FALSO	1
3,64E+18	FALSO	Low	IsMasterPr	AAEL0094C	3:38079300	100	4	FALSO	1
-2,91E+18	FALSO	Low	IsMasterPr	AAEL02023	2:14970794	100	5	FALSO	1
6,50E+18	FALSO	Low	IsMasterPr	AAEL00275	3:14522280	100	2	FALSO	1

-3,51E+18	FALSO	Low	IsMasterPr	AAEL0063C	3:2882263:	100	3	FALSO	1
-6,23E+18	FALSO	Low	IsMasterPr	AAEL0203C	2:2406420!	100	3	FALSO	1
-8,74E+18	FALSO	Low	IsMasterPr	A0A218KP6	Glutamate-	100	3	FALSO	1
-6,00E+18	FALSO	Low	IsMasterPr	AAEL01804	3:3226139:	101	1	FALSO	1
8,68E+18	FALSO	Low	IsMasterPr	AAEL00164	2:2979209	101	1	FALSO	1
-2,22E+18	FALSO	Low	IsMasterPr	AAEL00626	2:2212559	101	3	FALSO	1
-6,43E+18	FALSO	Low	IsMasterPr	AAEL00567	2:1319996:	101	2	FALSO	1
-5,48E+18	FALSO	Low	IsMasterPr	AAEL00755	2:2480307:	101	2	FALSO	1
-3,45E+16	FALSO	Low	IsMasterPr	AAEL01499	2:1285282(	101	2	FALSO	1
-3,69E+18	FALSO	Low	IsMasterPr	AAEL02692	1:2017273:	101	1	FALSO	1
-4,38E+18	FALSO	Low	IsMasterPr	AAEL01374	2:9561784(	101	2	FALSO	1
3,91E+18	FALSO	Low	IsMasterPr	AAEL00819	2:2476889:	101	7	FALSO	1
7,67E+18	FALSO	Low	IsMasterPr	AAEL02059	1:2455847:	101	3	FALSO	1
-2,82E+18	FALSO	Low	IsMasterPr	AAEL01379	3:2365437	101	7	FALSO	1
-5,05E+18	FALSO	Low	IsMasterPr	M9WN55	Orotidine 5	101	10	FALSO	1
2,99E+18	FALSO	Low	IsMasterPr	AAEL02618	3:3079746(	102	7	FALSO	1
-1,84E+18	FALSO	Low	IsMasterPr	AAEL00165	2:3000540!	102	1	FALSO	1
-2,82E+18	FALSO	Low	IsMasterPr	AAEL0017C	2:4431264(	102	1	FALSO	1
-6,17E+18	FALSO	Low	IsMasterPr	AAEL02155	1:9018893!	102	1	FALSO	1
-2,92E+18	FALSO	Low	IsMasterPr	AAEL00393	3:2629421!	102	6	FALSO	1
5,92E+18	FALSO	Low	IsMasterPr	A0A369RJC	50S riboson	102	9	FALSO	1
-9,25E+16	FALSO	Low	IsMasterPr	AAEL02378	1:1695331(	103	3	FALSO	1
8,04E+18	FALSO	Low	IsMasterPr	AAEL0270C	1:3555082	103	1	FALSO	1
-9,05E+18	FALSO	Low	IsMasterPr	A0A1E7QJ\	Ribonucleo	103	5	FALSO	1
5,70E+18	FALSO	Low	IsMasterPr	Q8L1V4	Surface prc	103	11	FALSO	1
-7,40E+18	FALSO	Low	IsMasterPr	A0A3D1L4U	Integrase C	103	5	FALSO	1
-3,40E+18	FALSO	Low	IsMasterPr	AAEL01118	2:1823518	103	4	FALSO	1
7,75E+18	FALSO	Low	IsMasterPr	AAEL00621	2:4910327(	103	1	FALSO	1
-1,38E+18	FALSO	Low	IsMasterPr	M4LIF7	Heat shock	103	13	FALSO	1
-5,58E+18	FALSO	Low	IsMasterPr	A0A3B0J6P	DNA repair	103	5	FALSO	1
-7,70E+18	FALSO	Low	IsMasterPr	A0A369RI3	Glycerol-1-	103	2	FALSO	1
-2,92E+18	FALSO	Low	IsMasterPr	AAEL02515	2:3867313:	103	3	FALSO	1
-4,98E+18	FALSO	Low	IsMasterPr	AAEL0100C	1:1942291:	103	2	FALSO	1
-2,73E+18	FALSO	Low	IsMasterPr	AAEL00523	3:8936158	103	1	FALSO	1
7,38E+18	FALSO	Low	IsMasterPr	A0A1V2N4	SURF1-like	103	8	FALSO	1
4,58E+18	FALSO	Low	IsMasterPr	AAEL02434	2:5211991	103	8	FALSO	1
4,52E+16	FALSO	Low	IsMasterPr	AAEL01397	2:1341747(	104	1	FALSO	1
-5,06E+18	FALSO	Low	IsMasterPr	AAEL00018	3:1090236:	104	1	FALSO	1
-2,58E+17	FALSO	Low	IsMasterPr	A0A098AS)	Transcripti	104	7	FALSO	1
8,28E+18	FALSO	Low	IsMasterPr	AAEL00634	3:3968064	104	2	FALSO	1
4,56E+17	FALSO	Low	IsMasterPr	A0A1E7QJF	Protein Tol	104	4	FALSO	1
-1,98E+18	FALSO	Low	IsMasterPr	A0A218KR(	TldD/PmbA	104	3	FALSO	1
3,91E+18	FALSO	Low	IsMasterPr	AAEL00487	1:2893865:	104	4	FALSO	1
-4,94E+18	FALSO	Low	IsMasterPr	Q5GT32	Uncharacte	104	8	FALSO	1
1,96E+18	FALSO	Low	IsMasterPr	A0A4Y6ULI	Uncharacte	104	0	FALSO	1
4,44E+18	FALSO	Low	IsMasterPr	AAEL00278	1:3047400:	105	1	FALSO	1
4,32E+18	FALSO	Low	IsMasterPr	M9QV41	Capsid prot	105	16	FALSO	1
-2,58E+18	FALSO	Low	IsMasterPr	AAEL02315	2:6889763:	105	3	FALSO	1
3,35E+18	FALSO	Low	IsMasterPr	AAEL00929	2:1810649:	105	7	FALSO	1
-3,30E+18	FALSO	Low	IsMasterPr	AAEL01218	2:1881058:	105	3	FALSO	1

-7,17E+18	FALSO	Low	IsMasterPr	AAEL02645	2:13279699	105	3	FALSO	1
7,53E+18	FALSO	Low	IsMasterPr	C0F9S3	SCO1/SenC	106	11	FALSO	1
-6,41E+18	FALSO	Low	IsMasterPr	AAEL00213	3:17732239	106	5	FALSO	1
-9,07E+16	FALSO	Low	IsMasterPr	A0A1E7QKI	Rod shape-	106	4	FALSO	1
-2,56E+18	FALSO	Low	IsMasterPr	A0A218KQ.	Helicase AT	106	1	FALSO	1
-4,52E+18	FALSO	Low	IsMasterPr	I7IU31	DNA polym	106	1	FALSO	1
3,31E+18	FALSO	Low	IsMasterPr	AAEL01362	1:61611710	107	3	FALSO	1
4,85E+18	FALSO	Low	IsMasterPr	AAEL00436	3:93880349	107	2	FALSO	1
-7,07E+18	FALSO	Low	IsMasterPr	A0A5F1B59	Uncharacte	107	9	FALSO	1
7,50E+18	FALSO	Low	IsMasterPr	AAEL01097	3:19043067	107	2	FALSO	1
2,47E+18	FALSO	Low	IsMasterPr	I7IAB8	UDP-N-ace	108	5	FALSO	1
-4,05E+18	FALSO	Low	IsMasterPr	AAEL01534	2:31378623	108	3	FALSO	1
1,83E+18	FALSO	Low	IsMasterPr	AAEL01400	2:35310443	108	1	FALSO	1
4,50E+18	FALSO	Low	IsMasterPr	AAEL00848	2:19352989	108	10	FALSO	1
8,81E+18	FALSO	Low	IsMasterPr	A0A176Q89	50S riboson	108	8	FALSO	1
-5,41E+18	FALSO	Low	IsMasterPr	A0A3B0IW.	Uncharacte	109	2	FALSO	1
8,71E+18	FALSO	Low	IsMasterPr	A0A3T0GIC	Uncharacte	109	2	FALSO	1
-6,75E+18	FALSO	Low	IsMasterPr	A0A2A4IIU.	FHA domai	109	6	FALSO	1
-6,41E+18	FALSO	Low	IsMasterPr	A0A060PVI	Short-chair	109	3	FALSO	1
-5,83E+18	FALSO	Low	IsMasterPr	AAEL00633	3:39702710	109	1	FALSO	1
1,54E+18	FALSO	Low	IsMasterPr	AAEL00115	2:30720070	109	3	FALSO	1
-3,33E+18	FALSO	Low	IsMasterPr	AAEL00643	2:43929560	109	1	FALSO	1
3,39E+18	FALSO	Low	IsMasterPr	AAEL01361	3:19529834	109	6	FALSO	1
6,14E+18	FALSO	Low	IsMasterPr	AAEL00312	3:27792394	110	2	FALSO	1
-6,58E+18	FALSO	Low	IsMasterPr	AAEL00124	2:15323980	110	13	FALSO	1
1,04E+18	FALSO	Low	IsMasterPr	AAEL01833	1:18117894	110	2	FALSO	1
3,56E+18	FALSO	Low	IsMasterPr	Q4E8V8	Pol protein	110	4	FALSO	1
-6,79E+18	FALSO	Low	IsMasterPr	AAEL02055	3:11151780	110	4	FALSO	1
-1,92E+18	FALSO	Low	IsMasterPr	A0A5C4TM	Malate deh	110	5	FALSO	1
-8,20E+18	FALSO	Low	IsMasterPr	AAEL00484	2:11493965	110	6	FALSO	1
2,61E+18	FALSO	Low	IsMasterPr	AAEL00526	2:69352217	110	12	FALSO	1
-5,36E+18	FALSO	Low	IsMasterPr	A0A178GZI	Uncharacte	110	5	FALSO	1
-7,98E+17	FALSO	Low	IsMasterPr	AAEL01041	1:26844729	110	7	FALSO	1
-1,16E+18	FALSO	Low	IsMasterPr	A0A5F1B5U	Ankyrin rep	110	2	FALSO	1
7,37E+18	FALSO	Low	IsMasterPr	AAEL00946	1:57117864	111	1	FALSO	1
-3,91E+18	FALSO	Low	IsMasterPr	AAEL01956	1:29502332	111	1	FALSO	1
-8,36E+18	FALSO	Low	IsMasterPr	AAEL02060	2:20862410	111	3	FALSO	1
-8,72E+18	FALSO	Low	IsMasterPr	AAEL02167	1:24130840	112	2	FALSO	1
6,94E+18	FALSO	Low	IsMasterPr	AAEL01361	1:68814374	112	3	FALSO	1
6,23E+18	FALSO	Low	IsMasterPr	AAEL00879	3:13267920	112	5	FALSO	1
-1,13E+18	FALSO	Low	IsMasterPr	AAEL01832	3:93850431	112	1	FALSO	1
1,73E+18	FALSO	Low	IsMasterPr	A0A060Q4.	Uncharacte	112	6	FALSO	1
1,23E+18	FALSO	Low	IsMasterPr	Q5GRR7	Ferrochelat	112	6	FALSO	1
-7,63E+18	FALSO	Low	IsMasterPr	AAEL00521	3:90514320	113	4	FALSO	1
-3,93E+18	FALSO	Low	IsMasterPr	A0A1E7QK.	Uncharacte	113	2	FALSO	1
2,74E+18	FALSO	Low	IsMasterPr	A0A218KM	Oxidoreduc	113	8	FALSO	1
-8,96E+18	FALSO	Low	IsMasterPr	A0A059IVR	Putative Ty	113	3	FALSO	1
-6,79E+17	FALSO	Low	IsMasterPr	I7ITV1	Uncharacte	113	5	FALSO	1
-5,51E+18	FALSO	Low	IsMasterPr	AAEL01811	2:22137514	113	3	FALSO	1
4,89E+18	FALSO	Low	IsMasterPr	AAEL01965	2:34767025	113	1	FALSO	1

-2,09E+18	FALSO	Low	IsMasterPr	A0A178GY	Uncharacte	113	3	FALSO	1
9,16E+18	FALSO	Low	IsMasterPr	AAEL01243	1:4587821	114	3	FALSO	1
3,30E+18	FALSO	Low	IsMasterPr	AAEL01434	2:2384394	114	2	FALSO	1
-6,76E+18	FALSO	Low	IsMasterPr	AAEL0138	1:1193419	114	3	FALSO	1
-5,08E+16	FALSO	Low	IsMasterPr	AAEL01254	2:2381230	115	3	FALSO	1
-5,52E+17	FALSO	Low	IsMasterPr	B6Y627	Protein Ap	115	15	FALSO	1
7,73E+18	FALSO	Low	IsMasterPr	A0A3G4YI9	Aminotrans	115	6	FALSO	1
-8,79E+18	FALSO	Low	IsMasterPr	A0A178GW	Recombina	115	2	FALSO	1
1,19E+18	FALSO	Low	IsMasterPr	AAEL0238	2:6575280	116	2	FALSO	1
-6,02E+18	FALSO	Low	IsMasterPr	A0A1V2N3	Uncharacte	116	6	FALSO	1
6,04E+18	FALSO	Low	IsMasterPr	A0A2A4IKN	Phosphorib	116	1	FALSO	1
-3,79E+18	FALSO	Low	IsMasterPr	A0A218KN	Peptidase	116	1	FALSO	1
-2,55E+18	FALSO	Low	IsMasterPr	AAEL01834	3:1599824	116	1	FALSO	1
1,74E+18	FALSO	Low	IsMasterPr	AAEL00051	3:1629209	116	1	FALSO	1
5,49E+18	FALSO	Low	IsMasterPr	AAEL00253	1:9035213	116	2	FALSO	1
-8,70E+18	FALSO	Low	IsMasterPr	C0F8E2	Uncharacte	116	12	FALSO	1
3,94E+18	FALSO	Low	IsMasterPr	A0A225X6	DNA mism	120	3	FALSO	1
-5,29E+18	FALSO	Low	IsMasterPr	AAEL0145	2:3251462	120	10	FALSO	1
-3,14E+18	FALSO	Low	IsMasterPr	AAEL0071	3:1908568	120	2	FALSO	1
-2,40E+17	FALSO	Low	IsMasterPr	AAEL00612	3:6871933	120	2	FALSO	1
-4,05E+18	FALSO	Low	IsMasterPr	A0A2Z4JV	N-6 DNA m	120	2	FALSO	1
-2,13E+18	FALSO	Low	IsMasterPr	A0A369RJI	50S ribosor	120	14	FALSO	1
3,79E+18	FALSO	Low	IsMasterPr	AAEL02434	2:2050993	120	3	FALSO	1
-8,37E+18	FALSO	Low	IsMasterPr	AAEL00024	2:2277807	121	6	FALSO	1
6,08E+18	FALSO	Low	IsMasterPr	AAEL01454	2:6516773	122	3	FALSO	1
5,43E+18	FALSO	Low	IsMasterPr	AAEL02751	3:2850325	122	7	FALSO	1
5,25E+17	FALSO	Low	IsMasterPr	A0A2A4IFJ	Uncharacte	122	3	FALSO	1
5,43E+18	FALSO	Low	IsMasterPr	A0A2A4IIL	ANK_REP_	122	2	FALSO	1
2,90E+18	FALSO	Low	IsMasterPr	A0A218KR	Uncharacte	122	4	FALSO	1
-4,66E+18	FALSO	Low	IsMasterPr	A0A218KM	Uncharacte	122	3	FALSO	1
-2,25E+18	FALSO	Low	IsMasterPr	AAEL00294	1:8591915	122	4	FALSO	1
-8,96E+18	FALSO	Low	IsMasterPr	A0A218KQ	DNA methy	122	3	FALSO	1
1,39E+18	FALSO	Low	IsMasterPr	AAEL0197	3:8786027	122	1	FALSO	1
-3,17E+18	FALSO	Low	IsMasterPr	AAEL0122	2:1435814	122	1	FALSO	1
6,89E+18	FALSO	Low	IsMasterPr	A0A060PX	Periplasmi	122	4	FALSO	1
5,49E+18	FALSO	Low	IsMasterPr	AAEL01702	2:4324661	122	3	FALSO	1
-6,03E+18	FALSO	Low	IsMasterPr	A0A369RH	Uncharacte	122	9	FALSO	1
4,22E+18	FALSO	Low	IsMasterPr	AAEL0062	1:2246390	122	2	FALSO	1
1,34E+18	FALSO	Low	IsMasterPr	AAEL0134	2:3511050	123	4	FALSO	1
7,03E+18	FALSO	Low	IsMasterPr	AAEL0111	2:2317991	123	0	FALSO	1
-6,55E+18	FALSO	Low	IsMasterPr	A0A3D1L3J	Uncharacte	123	5	FALSO	1
5,94E+18	FALSO	Low	IsMasterPr	AAEL01281	3:7554929	123	10	FALSO	1
-8,54E+18	FALSO	Low	IsMasterPr	AAEL0111	2:3458676	123	5	FALSO	1
-9,20E+18	FALSO	Low	IsMasterPr	A0A0U1D9	Ankyrin re	124	4	FALSO	1
-8,78E+18	FALSO	Low	IsMasterPr	A0A369RM	Single-stra	124	3	FALSO	1
1,51E+18	FALSO	Low	IsMasterPr	AAEL02353	3:3805031	124	2	FALSO	1
5,18E+18	FALSO	Low	IsMasterPr	AAEL0267	1:1316439	124	4	FALSO	1
5,69E+18	FALSO	Low	IsMasterPr	AAEL01334	2:1357362	124	14	FALSO	1
-5,60E+18	FALSO	Low	IsMasterPr	AAEL0084	1:1715113	124	2	FALSO	1
-1,97E+18	FALSO	Low	IsMasterPr	AAEL0006	3:3921393	125	2	FALSO	1

-8,82E+18	FALSO	Low	IsMasterPr	AAEL01295	3:20592868	125	4	FALSO	1
-7,04E+18	FALSO	Low	IsMasterPr	Q5GS88	DNA ligase	125	3	FALSO	1
-7,64E+17	FALSO	Low	IsMasterPr	A0A178GYI	Uncharacte	125	1	FALSO	1
-8,72E+18	FALSO	Low	IsMasterPr	A0A1E7QIL	50S ribosor	125	10	FALSO	1
2,59E+18	FALSO	Low	IsMasterPr	C0F9U3	30S ribosor	125	16	FALSO	1
8,46E+18	FALSO	Low	IsMasterPr	AAEL01975	3:5226505:	125	1	FALSO	1
1,90E+18	FALSO	Low	IsMasterPr	AAEL00403	3:16618889	125	10	FALSO	1
-6,96E+18	FALSO	Low	IsMasterPr	A0A176Q89	Uncharacte	126	13	FALSO	1
2,62E+18	FALSO	Low	IsMasterPr	M9WTC3	Dnaj doma	126	7	FALSO	1
-8,61E+17	FALSO	Low	IsMasterPr	Q5GTD3	ATP-depen	126	4	FALSO	1
7,14E+18	FALSO	Low	IsMasterPr	AAEL0258C	2:33549269	126	2	FALSO	1
8,68E+18	FALSO	Low	IsMasterPr	A0A178GX.	Uncharacte	126	25	FALSO	1
4,72E+18	FALSO	Low	IsMasterPr	AAEL0140S	2:1211147:	127	3	FALSO	1
8,30E+18	FALSO	Low	IsMasterPr	AAEL01065	2:13648234	127	1	FALSO	1
1,72E+18	FALSO	Low	IsMasterPr	A0A369RHI	Putative lip	128	4	FALSO	1
8,59E+18	FALSO	Low	IsMasterPr	AAEL00262	3:2566978	128	2	FALSO	1
8,09E+18	FALSO	Low	IsMasterPr	I7ITJ8	Major facili	128	4	FALSO	1
-8,14E+18	FALSO	Low	IsMasterPr	AAEL02732	3:2930916(	128	1	FALSO	1
-9,21E+18	FALSO	Low	IsMasterPr	A0A1V2N4.	2-nitroprop	129	8	FALSO	1
6,32E+18	FALSO	Low	IsMasterPr	AAEL0005€	3:12573893	129	3	FALSO	1
1,76E+18	FALSO	Low	IsMasterPr	AAEL0047S	2:1456339(	129	3	FALSO	1
-6,24E+18	FALSO	Low	IsMasterPr	A0A369RJ0	Uncharacte	130	7	FALSO	1
-6,18E+17	FALSO	Low	IsMasterPr	A0A0U1DA	Ankyrin rep	130	2	FALSO	1
5,83E+18	FALSO	Low	IsMasterPr	A0A369RIZ	Insulinase f	130	4	FALSO	1
-2,36E+18	FALSO	Low	IsMasterPr	AAEL0077C	2:4705650:	130	8	FALSO	1
3,64E+18	FALSO	Low	IsMasterPr	AAEL01094	3:1681853	130	5	FALSO	1
-3,44E+18	FALSO	Low	IsMasterPr	E9P605	Ankyrin rep	130	2	FALSO	1
-1,93E+18	FALSO	Low	IsMasterPr	AAEL0281C	2:2657413:	130	2	FALSO	1
8,87E+18	FALSO	Low	IsMasterPr	A0A3T0GJD	ANK_REP_I	130	2	FALSO	1
5,83E+18	FALSO	Low	IsMasterPr	AAEL00611	1:26598168	130	1	FALSO	1
-3,13E+18	FALSO	Low	IsMasterPr	AAEL0146C	3:3686561:	130	3	FALSO	1
-9,50E+17	FALSO	Low	IsMasterPr	AAEL0218€	3:29659835	130	4	FALSO	1
8,33E+18	FALSO	Low	IsMasterPr	A0A178GR:	Uncharacte	131	2	FALSO	1
9,17E+18	FALSO	Low	IsMasterPr	A0A218KN(	Glutaredox	131	17	FALSO	1
-6,73E+18	FALSO	Low	IsMasterPr	AAEL0275C	3:2923461(	132	4	FALSO	1
-2,21E+18	FALSO	Low	IsMasterPr	A0A369RH:	Uncharacte	132	5	FALSO	1
-4,92E+18	FALSO	Low	IsMasterPr	A0A5C4TLX	ANK_REP_I	132	5	FALSO	1
7,78E+18	FALSO	Low	IsMasterPr	AAEL01047	1:20402043	132	6	FALSO	1
-5,24E+18	FALSO	Low	IsMasterPr	AAEL00881	3:13325479	132	1	FALSO	1
2,66E+18	FALSO	Low	IsMasterPr	A0A369RJN	Sporulatior	132	8	FALSO	1
-1,31E+18	FALSO	Low	IsMasterPr	AAEL00058	2:2843042(	133	3	FALSO	1
5,34E+18	FALSO	Low	IsMasterPr	AAEL02632	1:57666799	133	2	FALSO	1
6,31E+18	FALSO	Low	IsMasterPr	AAEL02714	NIGP0100C	133	4	FALSO	1
-4,52E+18	FALSO	Low	IsMasterPr	AAEL02517	2:3890870	133	1	FALSO	1
-4,76E+18	FALSO	Low	IsMasterPr	AAEL02357	1:9160432(	133	2	FALSO	1
-1,89E+18	FALSO	Low	IsMasterPr	A0A3T0GKI	Uncharacte	133	5	FALSO	1
-5,47E+18	FALSO	Low	IsMasterPr	AAEL0234€	3:3031821:	133	1	FALSO	1
6,08E+18	FALSO	Low	IsMasterPr	A0A2A4IIS1	Valine--tRN	133	2	FALSO	1
1,33E+18	FALSO	Low	IsMasterPr	AAEL01284	2:3354375	133	2	FALSO	1
-2,69E+18	FALSO	Low	IsMasterPr	A0A059PC\	Uncharacte	133	9	FALSO	1

-8,03E+18	FALSO	Low	IsMasterPr	A0A369RJV	Ribosomal	134	10	FALSO	1
7,03E+18	FALSO	Low	IsMasterPr	AAEL01255	1:29504489	134	4	FALSO	1
1,87E+18	FALSO	Low	IsMasterPr	AAEL00113	3:4066143	134	2	FALSO	1
-6,68E+18	FALSO	Low	IsMasterPr	AAEL01254	2:2381354	134	3	FALSO	1
-3,68E+18	FALSO	Low	IsMasterPr	A0A5B9JXA	Uncharacte	134	2	FALSO	1
4,10E+18	FALSO	Low	IsMasterPr	AAEL00458	1:1448198	134	3	FALSO	1
-3,85E+18	FALSO	Low	IsMasterPr	M9WS94	Ankyrin rep	134	1	FALSO	1
-5,74E+18	FALSO	Low	IsMasterPr	AAEL0137C	2:3526783	134	15	FALSO	1
4,16E+18	FALSO	Low	IsMasterPr	AAEL02082	2:6296637	134	2	FALSO	1
2,88E+18	FALSO	Low	IsMasterPr	AAEL00896	2:4183418	134	4	FALSO	1
8,63E+18	FALSO	Low	IsMasterPr	AAEL0119	2:3013853	134	10	FALSO	1
-5,73E+18	FALSO	Low	IsMasterPr	A0A3D1L4	Uncharacte	134	7	FALSO	1
9,12E+18	FALSO	Low	IsMasterPr	AAEL01131	3:3221713	134	2	FALSO	1
-2,02E+18	FALSO	Low	IsMasterPr	AAEL02207	NIGP01001	134	1	FALSO	1
3,59E+17	FALSO	Low	IsMasterPr	AAEL02044	2:2994659	134	5	FALSO	1
-3,32E+18	FALSO	Low	IsMasterPr	A0A5C4TM	Bifunctiona	134	2	FALSO	1
-8,30E+18	FALSO	Low	IsMasterPr	AAEL02238	NIGP01002	134	13	FALSO	1

# PSMs	# Unique P	# AAs	MW [kDa]	calc. pI	Score Sequ	# Peptides	Abundance	Abundance	Abundance
2117	92	1963	2238	602	1290541	238	662243	695601	774278
1385	14	1940	2213	599	823494	160	30493	33021	60784
803	41	504	539	512	518387	44	515586	470818	497351
822	68	883	1032	558	498790	119	310587	312831	322288
734	7	376	418	539	446611	44	165079	136148	145680
667	64	551	594	894	395261	64	664945	649238	721465
612	161	2438	2806	526	384828	161	640473	594903	670506
457	19	255	297	615	359910	19	250221	197067	213098
564	5	376	418	548	333871	40	23149	18387	20881
452	28	332	354	818	333612	31	365548	332323	432240
516	130	2396	2763	550	329610	130	470049	460726	514348
513	139	3343	3696	766	302546	139	650480	613115	718136
498	7	376	416	548	296727	39	48746	76911	71426
463	1	1040	1152	560	293427	56	5849	5788	4195
461	1	1040	1152	564	292593	56			
480	179	8149	9160	665	281711	179	553706	515828	540373
440	8	645	748	878	269943	59	23081	27517	27927
397	67	1006	1120	952	259173	67	341714	310775	332716
414	19	7554	8569	552	248177	150	65186	64775	67940
347	30	282	307	854	240158	30	323664	316464	356902
378	1	4635	5217	590	226755	132			
366	1	376	417	548	226210	17	3676	3577	3917
352	49	788	857	844	224319	49	394615	393119	447540
358	2	612	671	677	216203	43	30353	26466	40453
305	21	210	228	477	216108	21	275469	249064	350557
351	2	395	437	600	215191	43	1548	1531	1471
347	118	4613	5034	502	212469	118	412227	339551	435761
354	11	447	501	486	209764	26	226919	223026	250072
342	1	355	398	640	206749	40			
336	49	570	632	876	205235	49	364221	372012	392463
355	49	2142	2493	718	199907	78	216575	194686	313595
238	14	178	202	637	194175	14	88438	98503	87537
335	2	376	415	548	192529	27	12981	12541	12402
317	29	651	711	547	189232	42	221430	214966	233836
234	25	441	455	802	182325	25	167565	167323	170960
297	1	376	416	548	171377	29	1464	1555	1766
295	40	284	324	486	160025	41	290826	292812	385924
193	8	136	145	463	158433	8	103401	112321	101352
261	32	337	353	904	156920	32	227493	225190	260068
250	5	363	391	787	156054	27	56847	53296	47322
264	1	284	324	481	152312	33	16037	11450	15676
255	39	614	682	539	151683	39	217462	217231	251644
243	32	529	574	739	148694	32	235831	214042	249482
240	3	248	282	488	145203	18	80193	83338	82434
221	3	601	680	897	143342	38	9509	9486	8123
235	59	1195	1321	681	142772	59	283832	266218	266582
240	4	285	329	474	140253	29	50359	32367	54568
210	24	508	536	683	138501	24	204883	184829	223028
212	9	140	148	692	138054	11	147235	133059	153719

213	2	600	679	897	137547	37	9409	6827	6858
227	49	770	825	925	137359	49	249398	222620	243496
230	51	998	1095	550	137350	51	181317	350033	228772
238	4	284	327	479	137133	30	22681	21599	21134
248	71	2087	2419	686	135859	76	204025	211539	281460
222	77	1736	2032	620	131900	77	245055	212042	231504
262	37	382	459	477	131434	37	255814	216945	306689
217	5	1063	1192	723	130888	42	11646	13871	15735
191	3	881	980	827	125960	50	10676	9532	8841
204	3	1016	1141	689	124470	40	20361	12883	17549
187	4	844	942	771	122796	51	2758	4226	3283
190	29	503	543	838	122462	29	157470	139979	172242
208	67	2422	2649	662	122106	67	237035	193549	215956
199	40	2080	2291	674	120810	70	114146	97163	117268
200	1	1681	1916	577	119383	57	1704	1905	1538
199	1	1678	1914	577	118836	57	3830	3617	3336
186	71	1969	2269	548	117958	71	192209	187354	194858
210	25	2148	2502	708	117816	54	99735	77550	152787
197	1	364	395	818	116563	23	6266	5208	6042
166	16	442	477	753	116274	16	113522	96933	130557
204	42	574	608	562	115420	42	199730	187276	219308
183	42	748	831	702	113739	42	133193	119657	155606
195	1	896	1036	560	112186	47	1729	1963	2083
171	40	1089	1192	701	111809	40	168012	162138	171408
183	1	715	814	503	111343	38	2310	2418	2239
193	1	896	1035	560	111109	47	6412	5606	6414
181	1	715	815	503	109933	38			
184	32	730	792	696	109638	32	183450	182357	202651
163	46	803	888	547	109330	46	148765	150767	167307
152	21	624	687	712	108701	21	184628	152775	187278
155	7	217	221	725	107026	9	76594	82975	81368
188	3	248	283	503	107025	18	24469	28893	32065
165	46	579	663	627	104778	46	153919	132141	152589
170	29	415	438	709	101428	29	163649	159805	187928
161	17	384	432	563	100796	17	179616	178364	225747
184	6	450	499	514	100721	23	41501	24979	40192
164	34	801	869	721	98472	34	190840	170999	190657
159	25	255	285	681	98399	25	130073	138177	165173
144	23	329	344	843	97637	23	115484	103558	135135
162	29	479	518	809	96463	29	218504	234880	219881
156	31	495	551	878	94826	31	173298	159145	164412
158	26	658	719	693	94326	26	153072	162073	160833
151	2	8924	9996	576	94110	82			
167	22	258	294	486	93659	23	226359	196863	246573
148	40	737	833	888	93478	40	139651	117034	120096
163	2	552	610	810	93331	28	10002	9500	9529
150	1	8924	10003	580	93273	81			
133	8	724	806	647	92709	32	6800	7759	6653
134	29	185	197	521	92601	29	169861	139017	183495
163	20	655	722	520	92601	31	63068	59067	62211

173	5	424	458	458	92392	24	13627	10857	11295
162	3	566	626	784	92021	29	21701	18484	18715
154	1	449	488	787	91116	25	6483	5784	7595
160	39	1082	1242	613	90828	39	186415	169057	181352
146	21	496	552	557	90717	21	127064	118483	128862
153	2	446	485	787	90166	26			
136	23	383	428	828	88647	23	134555	108502	136965
157	33	467	516	882	88541	33	125777	106029	149520
129	28	378	430	524	87893	28	177234	153137	177894
162	3	449	499	514	87690	20	12367	8894	9937
145	40	845	969	633	86535	40	150885	133989	153272
156	37	866	868	941	86001	37	135032	122090	127713
132	56	2866	3104	644	85537	56	152155	149578	160481
139	31	671	726	621	84928	31	131543	129360	127634
149	6	1024	1105	762	84189	41	19349	16608	18533
177	1	376	416	558	83929	26	14998	17033	17063
130	4	286	321	947	83427	20	66775	70961	59300
157	0	891	947	515	82604	20			
138	42	738	820	891	82419	42	186953	161363	184711
138	12	108	118	948	81696	15	117830	106625	130134
152	2	891	946	515	81437	20	5244	5864	6236
134	1	1484	1656	644	80599	51	1869	1637	1759
124	9	107	117	463	80412	9	87820	74375	73313
131	24	392	439	676	80333	24	87593	74405	99972
104	2	471	505	456	80315	17	12396	11023	13809
135	2	1464	1628	585	80171	52	6747	5576	6243
123	4	1339	1451	739	79235	41	15994	15421	14658
140	2	836	898	802	79079	37			
132	1	1439	1601	596	78930	51	4810	4127	4057
117	28	516	566	806	78916	28	86367	75562	90075
128	52	2054	2312	502	78803	52	134518	113680	135387
139	32	592	673	692	78432	32	212417	164491	192310
111	19	393	433	674	78190	19	113780	109752	113868
110	20	247	263	634	77668	20	99544	81470	118434
98	3	473	508	458	76323	18	6607	6009	7087
122	33	494	559	502	75968	33	154379	122082	173974
117	6	922	1025	673	75155	32	12057	11392	12246
140	21	327	345	907	74843	21	115477	114906	134916
127	5	1648	1795	715	74431	41	7872	6957	6746
136	55	5246	5934	597	74410	55	139579	148449	139341
129	25	398	448	834	74220	25	109740	105901	145816
112	1	1302	1415	750	73448	38			
126	26	420	455	815	73341	26	118661	111632	119310
132	15	172	195	539	73036	15	169270	180569	162067
127	2	704	822	555	72888	28	10703	11952	12676
120	11	645	705	540	72630	22	39562	40524	47419
106	2	610	679	733	72523	26	1199	1618	1570
108	1	193	201	828	72292	9	3165	3703	3617
108	1	170	175	747	72228	9			
131	5	424	468	911	72221	28	25115	22246	21874

89	29	1977	2289	454	71220	29	86275	130364	102692
112	3	3902	4335	550	71165	44	13206	10869	11144
113	10	176	187	813	70957	10	52897	49852	52673
104	22	505	569	785	70788	28	140957	114936	135233
116	45	2437	2653	498	70494	48	154893	138186	151672
109	1	4034	4490	538	70365	42	746	842	631
119	10	462	505	895	70162	23	63870	62436	87941
110	19	429	472	900	70036	19	159811	148256	159080
114	28	790	865	730	69925	28	80787	80209	85565
115	24	663	752	881	69588	24	134148	133292	123974
102	17	491	519	922	69128	17	75878	64751	82084
106	21	545	581	891	68002	21	83434	73740	77828
112	30	1088	1212	534	67898	30	107991	111479	120117
109	18	396	436	850	67023	18	108725	104970	122047
118	1	1537	1676	793	66950	37			
100	28	561	632	642	66503	28	88920	73325	96842
91	10	149	168	422	66495	10	48035	88099	89718
108	27	627	679	701	66249	27	95511	83675	93468
112	18	210	226	970	66143	18	132658	103504	155182
120	1	689	805	559	66066	27	972	978	1054
109	24	570	637	824	65653	24	101453	82303	95473
99	35	981	1113	595	65491	35	108684	101218	112528
114	9	463	504	909	65461	22	62770	57273	94487
116	1	421	471	904	65214	24			
101	20	521	565	777	63867	20	75261	71307	74636
102	1	814	900	623	63339	27	2973	2748	2707
98	28	493	552	578	61759	28	80521	55425	70097
87	6	165	179	1037	61725	7	59760	57492	59627
94	23	518	559	670	61466	23	79705	76818	84105
75	4	141	146	1014	59501	4	35900	33884	50871
96	42	3701	4116	502	58955	42	119651	105987	116584
79	15	842	833	964	58809	15	60800	46607	50469
97	22	544	595	828	58325	23	91961	92032	114347
78	8	306	332	846	58317	8	61803	78478	69241
91	27	628	697	947	57775	27	92471	70060	82197
87	25	372	405	696	57676	25	139527	121548	159243
95	27	633	706	744	57625	27	70187	62571	88328
94	1	241	268	881	57313	17			
112	8	449	505	492	57254	18	32882	32924	41255
106	41	1569	1804	870	57078	41	100477	100688	104832
94	26	844	943	639	57011	27	92832	84449	96011
100	43	2446	2748	623	56893	43	116311	97851	94671
100	24	272	299	553	56535	24	81480	70108	71491
91	1	1128	1248	652	56359	31	5709	4926	4520
76	11	134	149	661	55866	11	65955	63359	70349
94	17	198	214	876	55246	17	120532	100872	118793
93	14	106	116	948	54902	14	130838	125261	145657
93	26	684	783	706	54890	29	79840	68364	72617
95	22	512	580	671	54848	22	90174	84442	100095
82	29	514	561	762	54831	29	93695	89803	97336

96	21	254	274	873	54338	21	110143	93455	123668
99	12	166	196	963	54195	12	65844	65326	82151
88	2	1100	1187	812	54147	20	9738	10204	8076
86	19	324	338	848	54060	19	106304	99789	106346
91	14	400	446	705	53793	14	118567	117343	136311
89	14	354	385	680	53406	14	106043	97339	118829
91	21	407	471	586	52694	21	98680	81118	97377
81	12	169	190	797	52484	12	92949	91281	103089
83	24	443	499	559	52313	24	100877	90613	100407
76	26	964	1071	652	52260	26	72097	74156	70967
94	13	177	203	944	52206	13	111288	124901	126403
89	1	313	345	950	52181	22	3912	4027	3927
87	1	354	390	890	51865	22			
91	21	715	747	758	51819	21	81007	72042	74675
85	41	5515	6266	497	51810	41	86137	82116	87574
89	14	245	265	758	51793	16	60010	56568	67516
84	2	1990	2178	670	51679	20	2084	1749	2429
81	24	532	578	642	51483	24	66140	57041	65831
85	17	370	405	847	51303	17	51618	57342	68891
86	21	534	579	585	51031	21	83176	78363	83975
83	17	132	148	714	50686	17	67423	66552	84980
102	2	445	497	483	50547	9	11264	9595	9853
90	10	159	181	459	50536	10	96216	104127	127788
77	23	305	333	875	50311	23	91628	73403	84048
87	22	390	433	569	50222	22	115553	104940	116867
76	16	219	244	825	50213	16	86179	74510	86414
88	26	719	777	818	49864	26	87574	66074	78852
75	14	188	199	862	49855	14	70218	58700	84513
77	16	225	250	553	49787	16	69417	73870	78096
88	2	536	613	651	49646	32	8016	6742	7618
85	25	688	787	693	49589	26	51735	39018	54348
88	2	373	413	846	49519	17	9819	9610	10557
73	17	237	270	549	49378	17	70454	64879	79972
73	23	852	915	686	49059	23	58344	57537	56019
74	38	3472	3939	879	49029	38	44659	40450	46348
84	28	728	812	803	48415	28	83182	75741	87080
89	20	297	327	888	48323	20	133920	129592	135759
87	17	333	357	670	48279	17	89250	84263	93547
84	1	357	404	615	47170	16	3190	2581	2625
86	3	260	278	891	47148	14	21545	18905	18003
83	1	632	692	627	47045	16			
73	13	191	219	483	46996	13	72447	70883	92526
69	15	112	113	459	46949	15	48073	49234	50986
70	1	318	356	646	46918	13	10274	7215	11007
67	12	422	464	643	46699	12	57493	47917	53694
86	7	254	271	879	46551	16	27143	24981	25346
73	18	901	1007	406	46184	18	48613	59912	60971
73	13	468	528	684	45912	13	50670	54668	56868
82	20	390	426	835	45386	21	92367	73628	93827
73	26	986	1140	810	45249	26	93550	79745	83942

82	1	544	623	651	45176	31			
70	7	206	223	901	45140	7	63448	68590	66121
71	8	824	894	898	45113	27	17501	16469	15825
75	30	1332	1492	609	45107	30	76361	77537	72293
92	15	239	264	1026	44898	15	80716	80491	84193
69	23	483	535	557	44686	23	62323	55726	64448
74	12	2783	3173	520	44609	35	24966	22438	23804
80	2	377	418	831	44501	17	2604	2669	2577
79	21	609	679	661	44141	21	56041	61834	57634
61	14	550	615	822	44132	14	62000	53450	54869
68	17	381	436	637	43936	17	67318	45090	67504
77	6	220	246	434	43872	6	78750	78097	84940
66	12	93	108	958	43816	12	42377	52852	61336
78	3	213	234	536	43724	19	6456	4627	6573
74	1	213	236	469	43624	8	12792	10457	9948
79	14	448	505	492	43618	19	57332	49795	61253
69	15	443	501	834	43582	16	55318	51273	56188
68	3	325	357	493	43453	16	6472	7698	7888
68	10	266	282	844	43365	10	51270	43474	56827
65	1	317	355	619	43129	13	3814	2982	4040
76	34	4632	5150	511	43074	34	68298	61810	63740
56	3	315	342	698	42986	14	10861	9757	12488
73	1	213	237	464	42954	8	6095	4789	4618
57	11	184	202	851	42893	11	61099	55475	64063
63	12	211	232	743	42819	12	50014	47435	52411
81	16	302	329	966	42760	18	113291	102939	109041
65	19	604	658	658	42502	19	58045	46573	58481
63	17	505	552	506	42435	17	40692	36462	33736
69	22	754	868	728	42080	22	67916	57203	62646
65	3	892	960	935	41872	22	9098	9674	9169
58	15	409	433	837	41531	15	64439	52340	60633
59	14	437	474	625	41435	14	43003	40555	46700
77	23	363	395	909	41426	23	65353	57802	59780
68	13	398	416	854	41029	13	74785	73377	67678
64	20	758	849	778	40969	20	94882	88641	92129
62	20	1788	2005	535	40824	20	61716	56186	59885
63	33	5813	6743	571	40759	33	63640	56497	60966
62	16	171	196	478	40732	16	95274	78015	98216
67	16	469	507	900	40698	16	60407	58285	62821
61	15	729	842	531	40367	15	49166	46188	51653
67	22	850	935	543	40356	22	85241	83456	97202
73	8	353	417	534	40142	18	16445	11889	14748
73	19	332	353	791	40096	19	95641	75243	96287
70	19	1418	1520	633	39947	19	36229	31796	36107
58	12	563	640	788	39895	12	44634	41407	42997
72	17	689	782	637	39601	19	38068	29108	35420
61	1	291	321	487	39208	14	365	335	500
51	3	126	132	866	39155	3	22040	23254	20059
62	16	594	663	505	39139	16	55522	56131	51591
70	5	89	100	986	39081	5	76873	77506	66332

68	2	382	423	493	38918	18	15228	11233	10136
66	23	921	1033	739	38511	23	62410	53741	64913
70	18	291	321	904	38169	18	63398	58115	63598
72	28	795	911	491	38136	28	72520	52093	73806
85	7	119	137	828	38064	7	80419	70890	83417
70	13	196	218	596	37948	13	85568	64864	75276
56	15	1393	1517	662	37919	15	86857	77941	75156
53	15	280	298	890	37820	15	61032	45779	59838
48	11	740	785	711	37576	11	31164	28356	34732
67	17	498	541	519	37361	17	92933	82743	93325
60	19	463	511	796	37325	20	79570	62825	83297
60	10	146	167	497	37273	10	103624	78201	96364
53	10	156	171	681	37195	10	51570	48051	49429
67	4	662	733	505	37137	14			
62	32	3364	3797	806	37121	32	85014	78017	65638
53	10	176	191	529	37114	10	41333	41520	43136
68	5	353	415	520	37034	15	18602	32783	20893
62	17	1265	1414	520	36948	17	65318	62284	64277
66	17	401	460	892	36807	17	77108	70604	69528
51	7	153	154	624	36623	7	60906	44000	58441
63	3	503	538	890	36554	17	8928	10696	11447
51	15	468	525	787	36417	15	56877	50622	62387
60	9	184	191	895	36367	10	46792	35277	48593
66	17	561	608	624	36198	17	70403	66829	76927
67	23	1194	1317	554	36121	23	53383	47872	57380
40	1	160	169	431	35992	1	17340	17606	20822
63	20	512	579	717	35748	20	70279	58581	68570
52	9	185	212	931	35725	9	58239	57535	60820
46	12	347	383	665	35705	12	43266	38258	42022
56	16	173	195	640	35609	16	75636	65617	82505
50	14	500	559	639	35548	14	65913	63075	68981
45	12	518	583	658	35483	12	46048	40258	45359
64	2	487	522	882	35455	16	3418	2907	3471
57	17	504	568	756	35312	23	35546	30098	37587
51	21	1624	1799	544	35122	21	62983	60409	63410
58	22	1246	1397	637	35101	22	91898	79275	90993
59	4	2583	2952	723	35057	27	13720	13164	11661
56	13	1129	1227	458	34821	13	61392	50600	59107
63	17	606	661	667	34785	17	70560	66526	68493
57	1	1581	1781	620	34553	19	6361	4890	4739
59	23	966	1081	601	34428	23	72673	65448	72452
66	8	245	267	680	34320	12	33265	21713	33010
57	18	645	732	869	34217	18	69103	61119	68975
55	18	489	530	568	34132	18	82410	79260	83084
53	11	491	521	572	34065	11	50497	49090	46875
63	15	292	315	847	33980	15	76611	66878	91479
45	16	221	250	563	33968	16	41450	44861	50254
61	14	490	557	728	33921	14	98689	78306	110229
53	17	385	442	774	33920	17	87924	86135	82706
63	11	321	357	475	33672	12	39268	38907	46412

47	12	511	583	623	33657	12	35782	30863	34299
65	14	220	249	581	33427	14	93148	84553	115210
59	18	196	221	572	33085	18	52336	84703	67894
58	20	432	489	768	32993	20	101750	83090	104925
62	17	541	592	853	32956	17	67344	59145	59750
45	1	315	342	699	32936	12			
49	15	531	585	680	32668	15	44322	42180	42713
55	7	126	137	591	32493	7	58429	55178	68919
64	14	354	404	553	32437	16	59406	63053	67274
56	19	389	430	904	32311	19	70474	63774	68057
56	20	839	941	573	31707	20	68162	57445	66733
57	1	1656	1865	624	31638	19	7147	6084	7087
58	21	590	642	803	31593	21	69830	58741	66101
46	12	362	395	876	31578	12	65177	48442	61231
45	14	625	693	554	31574	15	32966	27411	32584
58	14	271	303	501	31504	14	72645	58513	72485
61	13	120	141	976	31457	13	68207	61296	75609
52	16	441	487	658	31445	16	54724	45244	59000
59	3	248	265	881	31371	11	11654	11095	9531
46	8	246	272	753	31308	8	18394	16449	15980
53	12	315	338	560	31060	12	31462	28561	33365
57	21	546	595	543	31038	21	84101	80830	89833
41	6	250	260	803	30931	6	21425	16131	19095
52	15	1034	1159	472	30866	15	43105	36189	42062
48	1	376	417	654	30864	13	3241	4148	3649
49	11	358	400	667	30826	11	49538	41018	44721
41	13	377	415	844	30765	13	43700	40875	45072
51	14	409	464	539	30760	14	85751	67131	83195
43	16	510	548	646	30455	16	24814	22356	23189
55	11	333	370	658	30407	11	64722	52559	65039
48	10	407	467	456	30309	10	56949	50034	56564
48	5	211	238	596	30224	8	24735	21593	25302
44	15	464	525	473	30215	15	52858	41852	43378
56	13	226	257	614	30086	13	63299	54028	79687
38	5	217	247	592	29689	5	37549	36256	39299
43	15	544	592	778	29606	15	59059	40450	52432
55	16	1140	1234	498	29591	16	75193	62891	67589
48	16	1199	1325	714	29527	16	47114	50993	46579
49	14	660	722	660	29502	14	41902	31677	35011
38	8	255	270	866	29497	8	31814	24516	26022
50	17	699	754	954	29456	17	45734	46285	44533
50	17	698	782	692	29401	17	60809	53127	65410
44	4	85	92	998	29379	4	19015	20185	21003
48	17	1083	1212	657	29306	17	37985	34259	34992
52	18	956	1047	520	29158	18	61965	57422	61701
45	13	227	250	692	29094	13	46546	43808	51722
69	1	454	508	497	29071	10	7331	6547	6481
41	6	230	250	734	29006	6	12676	15931	22670
50	12	130	146	564	28991	12	82007	66199	107059
47	13	339	367	857	28883	13	31592	26099	28118

48	7	139	144	507	28874	7	46344	45455	60735
47	15	1229	1422	869	28653	15	33606	31363	32434
41	12	1527	1720	594	28607	12	39057	40785	39608
52	16	667	767	740	28458	16	64574	61378	78834
44	10	552	601	766	28457	17	19456	17922	18916
49	18	509	572	596	28301	18	48771	39510	47902
60	10	169	189	1046	28226	10	55188	54019	82647
50	1	841	909	935	28198	18	1142	1085	943
45	1	381	423	662	28172	13	3535	2798	3785
53	6	207	244	970	28170	12	40450	34953	40000
39	4	367	390	848	28090	7	12160	12197	12999
45	7	209	238	607	28028	10	38061	26654	43797
46	16	1117	1223	478	27506	24	37389	30857	33064
37	9	198	215	525	27466	9	33769	24707	29599
43	4	131	134	935	27460	4	33096	33838	35288
49	16	275	305	632	27287	16	49652	42571	43361
55	9	207	246	961	27243	15	48018	40709	54446
46	19	836	927	554	27197	19	46543	46787	47365
49	29	4684	5343	635	27187	29	56912	51331	50319
42	8	261	284	690	27088	8	26732	24469	24721
39	14	1334	1454	472	27040	14	24613	18242	21437
45	1	194	222	491	27017	8	8075	6269	7904
45	16	685	783	766	26912	16	66522	49356	61134
43	8	312	359	605	26881	8	48934	40786	44262
32	3	755	829	529	26846	12	6491	6465	7043
53	3	488	532	530	26814	6	5620	4309	5119
45	14	386	426	550	26813	14	44080	41555	41197
53	13	198	220	771	26784	13	56623	52536	55906
53	6	82	92	992	26779	7	38663	36683	48641
42	1	243	272	482	26754	10			
42	4	285	326	533	26746	11	8771	6816	11225
42	1	254	284	472	26716	10	1562	1427	1570
41	16	381	422	508	26621	16	58965	50276	51116
50	15	289	323	863	26577	15	49424	38379	51778
47	13	404	455	599	26577	13	46300	43139	47065
42	12	580	635	903	26527	12	34874	32036	31246
42	9	194	218	841	26420	9	34843	37338	33026
53	5	116	126	957	26285	7	22309	20389	21082
53	10	134	165	980	26229	10	57333	52279	54169
42	14	349	383	796	26078	14	56991	54056	56625
39	8	446	470	621	26034	8	8392	8124	7660
44	15	265	303	580	26033	15	36999	31005	38939
48	11	486	533	869	26001	11	45511	42508	55447
45	11	229	257	596	25997	11	32950	26064	32960
50	14	431	489	1130	25979	14	63629	58714	70798
48	15	630	690	799	25920	15	51111	47161	48349
44	2	1131	1252	668	25899	15	4706	4915	4018
44	17	3644	4029	928	25873	17	22241	23249	25119
44	15	373	415	758	25855	15	67868	55131	59147
49	15	238	270	885	25819	15	59627	59958	59233

39	15	813	901	522	25733	15	29736	27446	28508
41	6	108	115	818	25714	6	29877	30783	30052
45	11	236	255	919	25629	11	54640	46864	57935
34	7	145	156	545	25591	7	33568	32601	32327
34	6	209	227	769	25440	6	9203	13345	15476
43	13	194	215	730	25372	13	68911	67024	77251
40	7	274	310	832	25259	7	50467	43092	51295
43	13	422	459	834	25249	14	50254	45522	48006
44	4	363	405	559	25227	17	5464	4388	5172
53	8	100	107	904	25088	8	71345	58229	76593
41	14	580	651	597	25048	14	74989	71410	74818
43	20	1550	1757	680	25044	20	60491	52812	55736
46	16	665	718	977	24955	16	55809	47468	47168
47	18	543	609	516	24953	18	71899	47998	73676
46	5	448	502	657	24878	14	11411	9685	9637
32	12	622	698	903	24822	12	20722	23973	20753
50	13	300	329	974	24803	15	52206	39503	42277
42	15	1931	2018	578	24785	15	41644	40161	40436
47	11	241	263	703	24741	11	59251	53212	54750
37	14	2837	3123	481	24692	14	26735	21657	26604
30	2	736	809	538	24647	11	3851	3026	3939
43	21	2651	2947	494	24647	21	53026	44485	49795
42	11	276	313	614	24584	11	24964	22107	25886
41	14	951	1100	686	24480	14	63895	62072	62022
35	11	1295	1465	599	24397	11	16804	16461	15823
40	11	410	467	527	24385	11	22259	19728	22489
41	2	288	326	559	24360	9	9438	7769	10284
41	1	1151	1266	677	24345	14			
38	4	340	373	652	24328	6	40192	37604	45109
42	15	403	455	841	24302	15	34336	32614	33026
39	11	541	609	711	24298	14	45410	34938	39530
33	13	583	644	715	24248	13	20584	18359	17858
37	10	252	238	974	24057	10	41656	36373	39726
44	1	848	961	660	24057	13	17496	16087	14374
37	14	409	464	534	23984	14	34378	29066	35296
37	14	668	757	586	23963	14	41228	34418	35021
37	11	153	176	467	23961	11	38214	48136	52834
38	7	95	106	983	23836	7	46819	43920	41792
43	2	169	187	677	23800	4	12070	14624	12080
33	1	832	904	802	23785	9	17824	17435	17456
44	7	126	132	1040	23694	8	25094	16287	19759
33	6	186	219	881	23649	6	30698	26576	27322
36	7	192	221	992	23620	7	23147	18779	23546
35	7	218	240	477	23384	7	58865	53954	54452
41	18	1384	1571	679	23205	18	44183	28693	46358
38	8	234	234	853	23131	8	40264	32789	36944
36	21	1953	2186	524	23122	21	29974	26363	27891
39	14	625	681	914	23019	14	57803	54507	57756
32	6	95	101	465	22920	6	23243	25767	20922
43	9	293	330	585	22759	9	54402	52535	55969

40	12	543	594	594	22746	12	41218	34494	43790
31	1	880	948	980	22705	11	3124	3472	3218
36	10	215	232	977	22696	10	25799	25661	27177
39	13	279	304	652	22588	13	38245	34708	36567
31	8	224	250	711	22555	8	63213	38371	66236
45	11	206	234	1071	22495	11	35428	31540	36226
39	9	2693	2892	611	22455	23	6418	4810	5295
35	7	161	170	531	22183	7	28376	24473	27871
42	10	148	171	720	22165	10	49895	43855	61817
36	2	194	216	652	22130	9	1870	1745	1515
36	14	507	577	742	21986	14	20299	16839	22150
41	16	663	728	840	21719	16	38326	36794	36925
38	19	1386	1553	667	21714	19	48454	39806	42463
39	15	667	746	705	21665	15	44844	41105	39946
39	13	346	376	916	21651	13	40106	34742	36052
33	9	192	219	640	21624	9	30135	27300	29711
34	9	209	237	597	21591	9	45426	56841	49299
37	10	354	389	806	21563	10	33877	31467	33174
33	12	801	920	807	21521	12	31276	27111	31516
35	9	327	359	475	21488	9	31575	28173	31751
41	11	353	406	869	21369	11	62272	57826	56849
40	19	1324	1461	739	21343	19	40215	31264	36850
35	7	109	121	948	21226	7	42169	32617	36963
34	12	2012	2252	825	21226	12	25484	19339	21217
36	14	562	627	829	21214	14	55964	48027	57919
40	5	99	109	964	21043	5	42911	39784	42477
39	1	861	977	651	21040	13			
42	14	320	364	703	21014	14	61318	55979	56232
35	14	429	468	777	21002	14	37893	34022	37653
33	12	398	435	679	20986	12	33282	28947	31863
30	1	101	103	677	20932	1	10420	10555	10109
36	9	335	367	573	20925	9	55459	48818	46100
39	2	437	490	640	20915	11	5809	5966	6259
32	13	813	919	687	20785	13	24055	23107	23940
34	6	132	144	446	20773	6	46089	30057	41577
31	4	159	166	703	20724	4	17618	19747	19770
34	13	159	180	976	20716	13	55825	39336	48926
36	10	157	181	979	20706	10	41358	35219	43208
32	3	121	127	850	20700	3	14914	12058	11405
32	8	211	216	850	20692	9	26862	21293	24131
37	1	146	162	620	20668	3	2173	2641	2639
37	17	1008	1154	660	20667	17	38733	33376	38374
31	7	87	98	422	20615	7	25118	20950	24474
41	14	429	474	613	20599	14	25751	24874	27888
34	15	415	446	860	20582	15	43801	34820	37439
33	3	166	189	413	20578	10	7543	6348	6419
39	14	316	356	547	20516	14	35847	29056	32119
34	11	445	482	681	20495	11	22589	19519	21900
31	8	482	530	771	20465	8	31839	25834	33798
33	13	616	683	602	20432	13	37571	37599	37698

32	3	326	377	646	20419	10	11897	16459	14026
36	10	510	580	587	20396	10	21152	20918	20422
30	7	235	250	595	20343	7	34635	28789	34750
36	1	370	413	580	20306	14			
31	9	323	371	574	20280	9	34162	31236	33520
36	10	419	475	913	20148	10	13743	11795	15443
34	15	535	614	524	20066	15	40750	31352	39069
35	11	214	245	781	20045	11	37217	36753	39425
29	8	836	922	777	19995	8	31428	28484	28421
33	11	274	300	506	19854	11	35511	32978	33213
34	11	759	812	692	19758	11	30364	25911	25798
37	14	1519	1664	683	19727	14	36838	32284	33609
28	11	328	364	705	19720	11	8473	8152	8583
27	2	351	404	780	19682	3	15217	16924	21558
32	5	166	167	935	19664	5	33123	27546	34601
30	1	323	378	618	19645	8	5905	5833	6107
38	10	430	457	717	19578	10	47271	47265	44625
25	5	182	202	500	19555	5	44744	40867	48686
35	15	925	1036	524	19502	15	43086	33635	43260
31	6	137	144	724	19491	6	36692	31996	36136
33	16	398	422	703	19444	16	38261	30719	32270
35	9	217	251	1122	19435	9	29603	23993	32188
32	2	922	1005	644	19435	16	5256	4793	4589
33	8	258	290	781	19412	8	49053	37442	44472
32	18	3384	3825	609	19323	18	39710	35701	38863
33	13	584	691	610	19304	13	38729	39231	42378
27	9	1060	1181	841	19199	9	15672	15791	15302
30	16	409	458	684	19190	17	44476	40688	46529
27	12	819	933	627	19125	12	29751	27609	33591
27	9	282	313	895	19101	9	9842	10872	12575
37	12	393	444	742	19077	12	24103	22541	27191
31	13	840	936	654	19037	13	19858	18371	18820
35	15	854	955	661	18985	15	39235	39137	39076
28	10	946	1061	525	18965	10	42005	31382	45340
27	3	339	380	610	18961	3	35282	35395	39307
33	12	308	346	618	18954	12	38471	33351	37775
29	10	428	477	534	18952	10	24295	20532	22202
34	8	233	252	890	18812	8	29743	27424	28277
20	8	621	691	491	18709	8	30824	21193	30777
31	8	432	476	573	18596	8	25605	19566	26850
26	5	702	782	803	18596	8	9554	7782	8043
38	1	803	905	857	18593	10	8442	7406	7879
26	1	846	954	487	18544	11			
33	7	168	184	863	18511	7	32038	26804	27838
33	9	240	265	736	18509	9	33093	26650	33472
26	11	1273	1339	737	18507	11	14858	11641	13739
34	14	1105	1275	571	18496	14	53608	44083	52597
32	6	151	176	917	18470	6	28877	32037	34979
26	1	833	940	488	18460	11			
28	9	1241	1354	731	18451	9	29578	32883	31394

33	7	257	305	835	18417	7	37099	30563	34799
32	15	1669	1763	625	18409	15	33942	29899	31613
28	5	209	216	660	18379	5	22184	25009	27578
29	5	153	156	919	18364	5	15491	19466	17464
29	7	692	738	445	18359	7	20633	19654	20916
37	1	800	905	862	18321	10			
27	1	1130	1235	974	18293	12			
34	9	441	480	821	18280	9	51524	37729	46509
28	2	844	918	802	18150	10	8959	5905	6716
29	10	530	583	662	18100	10	31509	24414	17670
32	11	421	456	671	18081	11	28180	22960	26560
33	4	189	220	942	18050	4	30214	31064	36198
41	10	83	94	954	18046	10	30917	33069	35888
25	11	417	463	559	18034	11	13254	13489	13098
32	11	434	486	564	18028	11	43367	40787	43684
27	7	267	293	573	17997	7	26150	22185	27108
24	6	413	467	793	17989	6	13758	14332	13816
32	14	679	774	587	17921	14	31105	32407	33678
27	10	1227	1357	702	17903	10	14497	12389	14591
27	12	911	988	928	17892	12	17096	15284	14963
26	11	2806	3097	526	17834	11	31127	29185	30338
34	12	452	503	887	17808	12	47464	39796	43557
25	7	340	374	699	17747	7	27377	23992	25790
33	13	1449	1602	692	17737	13	47047	41946	41708
29	17	3305	3705	848	17730	17	29055	28462	27987
28	4	270	299	827	17685	4	35603	34830	38389
32	11	519	575	587	17627	11	24364	21132	24381
25	9	570	608	957	17533	9	34865	27802	31339
32	15	594	672	881	17519	15	33726	36492	33714
32	7	368	398	870	17502	7	39061	41253	47641
20	3	191	199	720	17459	3	6142	7162	6578
29	7	320	357	892	17402	7	54348	53115	52290
26	2	1144	1250	977	17363	11	1632	1931	1622
32	13	1201	1310	780	17303	13	31059	24243	28963
25	5	90	100	517	17274	5	17618	17918	22233
26	1	127	124	649	17261	1	7983	8602	10884
26	10	311	349	788	17241	10	42571	37518	43453
27	8	347	383	470	17236	8	16501	13081	13169
35	7	354	386	863	17231	9	34949	53087	40900
27	15	549	586	534	17176	16	17081	4953	22031
29	7	219	238	809	17150	7	32208	26196	33039
29	12	304	340	629	17119	12	35051	30238	41123
27	1	152	174	399	17087	8	1818	2082	1852
28	14	533	572	747	17076	14	33995	27770	30105
30	10	392	432	684	17054	10	22253	19208	18282
29	11	416	468	572	17042	11	35177	29829	35819
27	15	972	1096	709	17024	15	46645	39451	42459
29	11	966	1080	793	17004	11	27515	24469	26462
23	5	116	128	434	16970	5	30406	22606	25446
26	11	294	331	718	16900	11	22286	23775	20015

33	6	114	127	870	16777	6	34745	37329	44035
28	9	249	281	844	16740	9	34414	30546	34493
27	11	1107	1206	441	16731	11	13419	10170	12212
29	14	516	560	679	16693	14	29452	24990	28273
30	12	884	965	859	16666	12	28717	29229	27925
32	7	547	615	560	16657	7	42835	40677	46230
24	8	512	559	785	16623	8	34927	31849	33658
31	7	628	712	587	16602	7	17769	14950	17451
28	8	206	228	530	16595	9	39361	43853	41988
28	1	579	594	750	16590	11	4315	4742	4024
30	13	718	787	967	16581	14	20395	17011	17532
29	13	1175	1294	724	16569	13	28208	24646	26656
28	12	2084	2273	892	16566	12	21862	22067	20242
24	10	661	736	766	16519	10	20990	20585	21006
32	8	228	259	554	16499	8	31270	26090	26578
29	10	789	866	442	16483	10	28622	26597	25431
31	2	107	118	948	16465	5			
28	8	446	491	813	16433	8	17332	14979	17862
27	10	315	351	627	16425	10	35237	29075	32579
33	3	312	339	511	16405	5	4689	7851	4983
27	9	573	623	868	16388	9	33832	30067	31430
28	7	174	201	847	16362	7	22221	27438	29167
36	9	332	386	923	16325	9	66396	44052	69543
22	4	138	159	591	16319	4	15355	12087	13463
25	7	262	296	1030	16290	7	31517	29644	27543
29	15	434	504	503	16265	15	11861	10402	11333
29	10	605	662	714	16263	10	35309	27245	30318
28	15	1965	2178	720	16252	15	22799	19469	23441
28	11	246	277	724	16206	11	33694	34748	34001
23	7	386	439	647	16156	7	29525	24353	29038
29	1	742	763	856	16153	11			
27	8	197	216	812	16136	8	34764	29569	30971
30	11	542	596	655	16128	11	35830	33009	37685
30	9	1804	2006	652	16074	9	18526	17668	17230
34	5	207	244	973	16046	11	15759	15059	15143
24	4	146	161	740	16025	4	5304	5525	5403
23	6	127	141	618	15984	6	12654	12489	15186
21	5	378	421	699	15966	6	20877	20153	19873
24	9	355	396	1054	15962	9	8806	8271	7478
23	8	391	442	885	15959	8	27017	23076	25208
26	11	2438	2662	644	15955	11	25819	21703	23871
26	12	593	650	785	15855	12	35297	34199	30720
28	12	881	971	563	15853	12	22039	23056	24302
21	6	217	234	505	15775	6	19819	19078	18606
28	13	945	1018	822	15666	13	32211	31268	30222
18	4	195	216	620	15661	4	9068	9429	10596
23	11	342	405	793	15619	11	17322	18264	18121
31	9	151	162	1035	15608	9	30736	23609	24007
27	10	217	243	824	15599	10	23804	21637	21810
28	15	1391	1566	730	15590	15	53886	47990	46200

23	8	995	1081	674	15584	8	12269	10412	11315
31	8	551	628	632	15584	8	29006	28693	27047
29	15	1866	1974	951	15581	15	44464	39189	42114
28	2	196	206	891	15577	8	3736	4068	3463
26	13	756	854	960	15539	13	24765	22678	23563
31	13	1066	1202	680	15516	13	34864	35619	35055
31	13	3432	3804	627	15511	13	31981	28239	26643
23	4	111	129	1021	15507	4	9843	8898	9523
27	3	395	447	628	15492	11	9753	11546	11410
28	15	1372	1470	736	15474	15	35929	30904	32989
33	7	350	383	885	15431	9	36974	45481	34508
28	1	193	215	652	15429	8		1805	
25	10	933	1043	734	15420	10	8711	9279	10295
30	2	385	420	1008	15416	12	5963	4386	4330
23	7	367	389	488	15408	7	44871	45835	44180
26	10	257	296	851	15371	10	38578	33907	41019
27	1	549	564	780	15356	11			
31	3	210	228	505	15331	9	19982	4391	18665
25	8	338	381	478	15222	8	23928	18147	21587
25	7	408	453	742	15193	7	24512	20852	28183
25	8	557	611	643	15160	8	17759	13354	16583
25	6	313	357	540	15142	6	25637	16450	24192
23	2	126	126	954	15123	2	7658	9062	8687
19	7	183	209	829	15102	7	9973	9656	10078
23	6	256	274	784	15098	6	24096	21631	20737
32	2	111	123	973	15075	4	6329	5489	5504
24	7	250	274	791	15012	7	23849	17262	20547
20	7	426	489	677	15009	7	12746	13285	11006
19	5	231	239	489	14987	5	14352	12449	11062
29	6	635	695	599	14928	8	6207	7308	7925
28	6	165	194	709	14923	6	31425	27026	34279
26	17	4327	4958	649	14897	17	23523	21946	22839
24	14	788	891	521	14856	14	21909	22074	24721
29	3	855	934	1004	14828	13	6081	4969	7369
31	6	404	438	761	14786	7	22580	17344	20776
23	9	373	419	511	14737	9	18977	17780	19102
24	5	223	250	477	14703	5	34623	23882	28621
15	2	490	528	821	14682	2	16363	21939	24752
22	11	289	310	888	14680	11	19625	17022	19743
22	8	457	522	860	14679	8	16036	16037	15091
25	7	398	443	660	14668	7	37909	37660	35764
24	8	585	660	976	14596	8	27620	23029	25963
21	9	383	402	596	14582	9	17231	14628	15477
22	9	796	868	727	14542	9	22824	22237	19200
24	10	371	408	769	14534	10	21768	17674	22563
23	7	784	853	585	14524	7	24342	19636	21374
25	4	245	267	657	14523	6			
16	5	380	412	494	14460	5	11352	8756	10602
18	5	257	297	680	14439	5	9252	9548	9341
25	11	688	801	580	14374	11	23966	19041	22695

21	6	401	442	758	14358	6	20614	18469	19193
27	7	219	232	467	14353	7	38129	25811	31819
24	10	694	770	848	14346	10	11294	10045	10267
25	11	1892	2035	674	14328	11	14967	14106	14328
22	7	254	286	708	14291	7	20852	21853	20106
22	10	418	480	560	14275	10	24128	20682	22843
27	9	437	476	534	14250	9	37980	28481	44719
28	8	293	323	887	14233	8	30074	27766	30895
24	2	137	146	470	14201	2	15716	13621	16335
30	8	152	170	558	14198	8	24232	22666	27000
25	7	362	385	758	14174	7	23304	22362	21961
16	4	104	111	484	14165	4	16505	12297	13556
29	3	59	67	1043	14148	3	36649	35851	25585
18	9	504	556	863	14109	9	13491	13215	13109
23	10	704	772	706	14093	10	41205	36562	31856
24	6	261	273	1078	14093	6	32510	27147	26994
25	11	541	608	892	14084	11	17633	14134	15249
23	11	725	812	680	14073	11	30469	30278	32326
20	3	616	686	853	14050	6	6772	4839	5861
23	3	149	159	494	14047	3	19596	20826	22333
25	4	130	147	515	14040	4	21849	23217	23615
25	6	72	84	718	13961	6	18154	15185	16912
25	9	233	257	655	13955	9	28411	24614	28274
24	12	811	889	793	13951	12	29138	30325	28308
30	4	109	122	991	13902	4	17002	15892	14493
24	1	272	313	654	13795	11	3385	2224	2301
24	16	5119	5783	552	13780	16	16828	17531	15968
26	8	557	595	657	13758	8	24511	21425	25331
27	0	206	216	906	13710	8			
22	16	1174	1331	599	13706	16	29154	25337	24485
25	10	672	729	637	13699	10	28927	24452	24646
25	2	1142	1283	796	13633	7	1910	1901	7698
22	8	376	427	706	13594	8	13199	11394	12246
17	6	322	344	749	13583	6	6713	6567	6545
21	9	583	634	829	13541	9	38244	32182	34901
22	9	206	231	665	13539	9	27851	22854	24131
21	5	445	494	903	13508	5	29077	20883	22204
24	9	321	369	778	13484	9	36794	31580	53092
25	5	240	271	919	13482	5	19847	18775	15922
20	1	127	124	832	13476	1	8590	12180	9018
23	7	468	537	576	13453	7	40705	31863	40971
20	7	300	331	906	13444	7	24905	29080	25019
26	10	363	418	1163	13394	10	22545	23157	24783
26	9	145	171	894	13392	9	33353	27671	40429
24	11	901	987	616	13389	11	34861	29578	36273
18	8	377	404	705	13370	8	24409	22000	24491
18	4	385	415	475	13368	4	12926	11236	12049
22	1	272	315	655	13315	11	2825	2964	3017
20	3	101	112	459	13278	3	13034	9787	11549
24	5	193	203	1032	13260	5	20207	15726	18161

25	13	2681	2951	668	13243	13	34941	31490	35753
24	7	437	498	674	13235	7	33414	26980	32638
22	7	130	152	988	13223	7	28181	21088	24277
27	8	200	227	815	13178	8	15704	16861	19063
23	10	550	608	585	13155	10	15205	12685	14068
17	2	1066	1164	676	13141	2	4685	5403	4708
23	9	579	666	583	13136	9	13926	14066	11318
22	10	418	470	596	13134	10	21645	18883	20938
26	16	1011	1130	520	13131	16	27023	18226	23382
20	10	216	242	601	13115	10	24873	27651	24454
23	10	680	757	625	13096	10	18465	16244	15304
26	5	174	193	483	13087	5	38181	33318	37704
22	10	601	658	816	13082	10	15486	13880	12479
28	7	615	707	472	13071	7	30173	27138	31433
26	9	156	173	996	13068	9	51090	45303	60428
20	7	329	370	715	13015	7	21504	16609	20411
24	5	157	175	536	12998	5	10817	9854	12017
21	10	1227	1390	746	12994	10	27227	27543	28030
22	4	275	310	572	12907	4	23218	20785	18892
28	12	349	381	922	12903	12	29594	24089	24944
21	6	237	270	569	12884	6	7696	5245	14612
30	4	78	88	950	12881	4	35613	37558	44471
22	9	415	459	635	12864	9	19438	15300	18113
26	13	546	596	657	12855	13	30715	26952	33109
28	7	112	127	938	12832	7	14832	17003	26939
24	10	330	380	897	12812	10	30232	25099	22004
19	8	310	355	568	12767	8	10857	10353	9640
24	10	443	480	837	12742	10	23878	19138	21262
15	6	112	115	428	12727	6	8464	10926	9740
21	3	64	71	976	12725	3	1365	764	4688
25	8	188	207	812	12619	8	16900	14541	17961
20	7	367	399	879	12597	7	9494	8269	8890
19	6	324	359	594	12585	6	16516	19172	16565
18	8	716	822	683	12551	8	9525	9352	9324
18	6	512	572	536	12463	6	23875	21283	22799
21	7	286	315	592	12462	7	33434	29829	31242
15	6	553	635	597	12457	6	10683	14428	14109
19	9	1507	1709	828	12451	9	12493	12377	11720
17	7	693	732	837	12425	7	13361	12269	11416
22	11	1268	1394	673	12411	13	15980	16027	16484
25	7	139	160	665	12411	9	27199	17344	19715
20	8	469	509	749	12398	8	20443	18139	19909
22	1	156	180	974	12344	6			
27	10	243	269	961	12342	10	26421	20135	24025
18	7	775	883	671	12339	7	21158	17818	22310
22	10	1218	1344	766	12315	10	21120	17640	18520
19	10	1260	1398	693	12313	10	26644	21417	23877
19	7	269	302	887	12310	7	25219	24293	21621
19	8	438	491	658	12301	8	9301	8427	9366
19	7	563	625	408	12289	7	25694	29652	24634

20	5	557	619	731	12282	5	14761	15356	13401
18	9	923	1020	681	12249	9	25701	26701	25637
22	10	394	438	623	12238	10	16124	14268	16692
20	11	911	1021	582	12234	11	13348	11677	12495
14	7	1054	1150	578	12226	7	4336	4824	4949
21	7	899	992	535	12223	7	26798	25599	29183
28	3	207	243	963	12213	9	13592	11200	13736
18	8	1212	1353	563	12210	8	9887	8568	10553
17	8	255	269	857	12190	8	11282	8293	7633
20	9	289	330	544	12169	9	14126	12832	13968
19	9	522	557	725	12160	9	13386	12642	13483
20	8	749	839	623	12157	8	10953	11019	11998
24	11	488	527	586	12135	11	48664	37457	42891
23	8	288	327	602	12115	8	39491	37057	44207
22	6	297	340	976	12084	6	30767	26744	31632
18	7	270	301	947	12072	7	14198	11465	12686
23	1	536	582	799	12053	8			
19	2	55	64	925	12044	2	10422	8989	9316
20	13	911	987	493	12020	13	23606	22690	24119
23	8	493	538	493	12017	8	29939	30360	33646
21	8	576	661	662	12014	8	19871	14297	18090
21	4	322	364	909	11986	4	34781	38310	33122
22	1	830	929	652	11983	10	3732	3029	3776
21	10	2096	2334	564	11908	10	28411	24682	24000
17	5	127	139	718	11898	5	25109	21150	20097
21	7	182	205	511	11892	7	17605	15954	17701
19	6	270	302	624	11891	6	10011	9707	9730
17	11	535	593	615	11880	11	18431	18191	20159
18	8	784	879	780	11876	8	2961	1999	2696
24	7	146	176	941	11870	7	21485	20029	21839
23	7	346	393	1052	11850	7	17045	15708	16390
18	6	319	342	803	11835	6	11004	8387	10522
21	5	253	271	436	11831	5	28949	15910	21575
13	4	321	318	914	11792	4	7201	6657	7475
23	8	311	352	535	11787	8	25556	18850	22871
16	3	104	116	392	11768	3	13487	9458	25961
25	1	1284	1454	585	11760	9	257	197	207
18	7	193	216	484	11759	7	13008	11986	10864
23	12	397	444	521	11758	12	39410	29586	40965
23	12	1005	1120	730	11750	12	28165	25019	25938
19	5	1274	1417	550	11744	5	23323	22238	22252
23	3	321	360	482	11738	5	6721	6206	7674
22	6	387	438	550	11733	6	13692	13645	15445
20	7	237	272	974	11731	7	19009	19974	20307
20	10	684	795	664	11702	10	22803	16586	20010
25	5	136	154	1127	11702	5	18228	21734	17795
21	9	393	457	600	11688	9	19107	14510	15778
20	10	449	514	534	11675	10	26332	23116	22980
16	3	171	191	873	11674	3	6258	6263	5369
21	8	286	311	600	11656	8	29350	18210	21499

22	8	424	465	816	11626	8	20606	15630	16259
19	7	524	556	479	11619	7	12354	13296	14733
25	10	178	205	1108	11614	10	24387	19327	25973
19	3	219	239	473	11603	3	19882	14989	18897
23	8	1289	1416	698	11588	9	9021	8793	10448
16	6	397	456	585	11536	6	23100	22256	22019
20	8	513	585	607	11527	8	23436	22598	24252
18	9	505	573	925	11518	9	12709	11887	12382
25	5	151	176	969	11511	5	19535	15232	15921
20	3	165	176	897	11506	3	19365	23904	24026
23	8	387	418	554	11504	8	33720	19636	27222
23	1	494	569	644	11500	9	1404	1431	1585
22	2	480	524	529	11499	4			
23	5	224	250	605	11482	5	31473	25796	33421
20	9	322	350	929	11473	9	23273	18550	23911
21	8	317	350	790	11431	8	34402	24267	29073
22	9	811	910	909	11402	9	22119	21150	25684
19	6	127	146	698	11389	7	12549	8208	15488
19	12	1242	1428	866	11354	12	28031	28565	30176
18	5	376	422	687	11338	5	11090	10326	10860
19	7	185	198	825	11338	7	18561	15590	15551
21	1	400	453	606	11321	9			
18	8	353	385	895	11307	8	17216	12735	16752
15	6	620	670	894	11273	6	9953	9898	9726
24	1	1284	1454	581	11247	9	1184	1085	1065
21	9	723	819	519	11233	9	35647	31079	41227
22	5	365	388	455	11222	5	18680	16549	18103
22	5	456	518	601	11217	5	30393	24153	30700
13	6	326	371	562	11212	6	17881	17179	15573
18	5	205	225	690	11205	5	19908	15690	16073
20	8	256	297	916	11205	8	18982	18923	19492
20	13	1638	1833	841	11191	13	33970	28057	33014
18	7	247	267	843	11157	7	11872	8402	9832
16	6	306	338	699	11137	6	8658	5155	7559
20	6	320	347	539	11128	6	32750	32343	33619
21	8	476	543	794	11123	8	12969	10450	13999
20	1	526	573	448	11100	9			
20	7	246	274	689	11089	7	27984	21853	24077
22	2	188	198	892	11026	9	2542	2022	1789
14	5	344	380	484	10970	5	17313	14422	16111
17	8	275	310	759	10954	8	16370	13647	17432
21	1	547	622	739	10948	9	1672	1489	1873
14	6	217	240	531	10943	6	11170	8842	10083
20	7	126	144	898	10909	8	8156	6865	8767
25	5	193	207	888	10896	5	22534	19795	25008
19	8	1325	1480	813	10885	8	7570	5870	6194
16	5	200	221	706	10884	5	8901	8240	7830
24	11	398	448	705	10876	11	21405	16196	19399
17	9	431	490	613	10859	9	20375	15913	17887
19	8	242	269	478	10853	8	19746	15555	20006

23	6	180	206	848	10846	6	26274	29062	29514
17	9	927	1056	619	10843	9	30296	26499	30351
22	11	804	900	778	10797	11	13260	10531	10003
20	8	275	301	730	10779	8	19779	16670	19660
19	7	296	309	1062	10762	7	13331	11704	12703
23	10	715	802	791	10743	10	31360	32883	29565
14	5	288	326	539	10738	5	23020	15945	19273
18	7	1272	1435	642	10737	7	23961	24667	22771
16	7	403	442	648	10735	7	17601	19639	18477
19	8	326	360	590	10724	8	17562	15798	18222
15	5	168	186	492	10712	5	4771	4519	3635
20	7	1233	1386	559	10707	7	27629	26338	27489
20	5	118	127	994	10695	5	35372	31269	35629
16	6	214	236	863	10690	6	19383	17514	18483
16	5	182	206	714	10685	5	13471	12642	13513
17	10	1888	2180	863	10669	10	25483	23820	23231
18	8	283	299	1039	10636	8	30876	26851	25972
20	4	152	173	560	10607	4	39052	28028	31287
22	7	415	468	1023	10605	7	21089	16192	24563
14	2	136	144	595	10584	2	16559	14984	17579
16	6	394	425	493	10580	6	13820	15742	14981
18	4	190	214	992	10558	4	10331	9072	9074
18	6	336	368	610	10554	6	21925	19995	35107
18	7	848	965	825	10544	7	14143	12585	12943
18	7	616	682	610	10528	7	22273	18706	22747
18	8	693	764	646	10527	8	25325	18645	21490
21	7	472	512	917	10518	7	13804	11404	12764
18	9	408	463	582	10506	9	20266	15574	18263
18	8	702	781	657	10473	8	21708	19769	19657
15	4	272	303	832	10465	4	8524	8229	8849
20	6	246	279	957	10457	6	31640	17528	21179
20	8	899	1003	664	10449	8	34787	26879	28271
22	8	253	277	501	10427	8	20258	21330	21045
18	7	232	263	503	10383	8	34902	28742	33306
19	11	614	687	552	10354	11	23483	17104	20959
17	5	261	288	728	10345	5	12580	8991	11404
17	6	446	505	660	10332	6	10333	9121	11179
16	6	394	434	585	10320	6	13260	14132	14408
15	8	1670	1796	454	10309	8	9563	10208	9468
19	8	397	434	481	10308	8	25594	22382	24657
17	9	910	1060	628	10275	9	22636	20901	20787
15	8	456	501	624	10260	8	21900	19762	19851
17	9	562	628	527	10219	9	17345	17142	17254
16	8	530	605	689	10213	8	15629	11495	11921
16	7	224	246	473	10188	7	15628	12691	15949
18	8	433	490	644	10186	8	28484	25481	28696
20	8	669	749	718	10186	8	28159	23642	26992
19	8	462	516	596	10155	8	14985	13401	15442
17	1	864	967	668	10146	10			
22	6	1788	1950	458	10142	6	12463	10455	9671

18	2	126	136	938	10133	3	5323	4769	4869
14	5	138	153	448	10118	5	9659	12159	11747
14	4	606	661	683	10093	4	11050	10689	9964
20	6	273	321	901	10089	6	33969	33052	39809
19	5	74	81	712	10077	5	30642	25503	31503
19	8	843	946	714	10053	8	11125	8745	10549
23	8	265	296	665	10047	8	53917	55072	47952
17	9	1182	1347	730	10039	9	12478	9234	12519
18	2	142	155	606	10022	2	26604	28852	34905
16	10	412	458	586	10020	10	19384	16791	16124
18	6	1133	1332	866	10018	6	19358	17374	18262
15	4	219	246	944	10003	4	5870	5781	6333
17	4	210	243	851	9999	4	12698	13055	14847
17	4	307	352	502	9939	4	9234	7219	7705
17	8	762	843	497	9926	8	15034	15496	15971
16	8	1080	1246	655	9885	8	10448	7361	9917
16	4	218	231	652	9878	4	16662	15360	17999
15	4	418	463	592	9858	4	7178	6432	6572
16	4	394	410	670	9849	4	10175	8210	8061
15	7	222	248	654	9841	7	27664	18484	27460
22	7	232	259	853	9833	7	28329	22881	27257
15	5	511	563	936	9811	5	11827	10336	11827
14	6	413	476	615	9808	6	13994	12095	13684
14	7	333	363	910	9799	7	12918	14883	13957
16	7	1111	1215	578	9795	7	15165	16279	15493
13	6	471	537	667	9770	6	21498	21228	25678
18	11	405	462	926	9737	11	24753	20685	19413
20	6	152	175	1045	9721	6	25426	22121	22384
17	6	386	442	618	9717	6	15416	14569	17604
16	7	352	405	711	9680	7	22040	18775	19126
14	7	390	428	498	9669	7	8354	6048	6441
13	5	407	444	441	9667	5	11477	9988	11300
19	5	133	138	812	9655	5	24957	24272	20350
14	5	225	251	482	9627	5	17103	15020	15626
15	7	662	746	680	9611	7	14745	14519	12940
19	0	229	237	901	9583	8			
13	7	413	454	486	9569	7	11582	9426	11057
22	7	100	114	1052	9569	7	15780	14068	14616
16	3	191	195	919	9545	3	13580	12579	14638
15	6	514	564	676	9544	6	19299	18680	20989
23	7	215	243	601	9519	7	23132	20344	21542
20	4	70	83	1005	9519	4	16383	13807	12995
15	7	186	198	730	9518	7	30633	24059	26725
18	6	660	730	828	9480	6	11536	10821	12632
14	4	213	237	639	9470	4	26133	21320	24695
19	8	554	631	497	9451	9	12025	10806	11049
15	6	162	179	728	9443	6	27604	21658	27835
15	3	665	741	731	9439	3	2097	2387	1862
14	7	951	1055	600	9394	8	15848	15820	15376
16	7	1048	1191	647	9386	7	21479	20784	23675

14	5	211	242	590	9382	5	10915	10094	14441
18	5	418	481	783	9337	5	14854	12859	15579
16	1	78	91	887	9331	1	15849	23503	20334
17	7	607	668	683	9330	7	16967	13907	14284
17	3	231	256	679	9298	3	26096	24034	22421
13	7	469	501	887	9275	7	13266	12902	13698
17	7	703	797	712	9271	7	16228	13032	14509
19	8	275	299	1032	9251	8	23570	26310	29965
19	7	1066	1117	755	9199	7	27032	23084	21187
16	9	1342	1481	591	9197	9	12833	8969	10036
14	6	321	361	511	9190	8	16193	12714	15203
11	3	453	518	512	9190	3	7082	5764	6728
15	5	1234	1386	613	9183	5	19868	19585	19361
15	7	437	493	925	9176	7	23842	21768	24027
13	4	162	184	455	9161	4	10761	7673	9903
14	7	513	546	780	9156	7	14697	13968	12913
15	4	392	437	421	9141	4	16326	13764	14838
13	5	364	396	554	9138	5	9055	8259	8169
15	10	1348	1487	680	9117	10	8719	7407	8628
16	2	142	162	558	9099	2	17056	15996	12741
15	4	417	477	643	9080	4	41054	25078	26107
16	2	245	267	840	9039	3	4836	4445	4926
15	7	1578	1694	952	9036	7	2056	2116	2279
15	5	192	217	992	9028	5	18915	20430	16830
16	7	203	234	530	9022	7	25933	16500	19182
16	7	832	903	522	9014	7	25413	26268	24442
16	6	299	342	677	9002	6	26420	18224	21232
16	9	1088	1217	474	8996	9	21102	17122	18555
15	10	560	641	903	8982	10	14024	14415	14971
21	5	110	133	728	8973	5	26388	17642	28992
20	6	236	271	1092	8970	6	11197	9300	13453
16	6	1116	1274	625	8935	6	18973	17108	15306
15	8	695	795	668	8931	8	26574	24541	28040
10	6	1199	1256	796	8928	6	3296	3383	2899
12	9	1198	1355	816	8909	9	7154	7748	6469
16	8	1277	1415	654	8877	8	16554	14832	14811
15	5	458	493	698	8868	5	14776	13631	14144
13	5	323	357	828	8864	5	1804	1962	2177
17	4	445	483	491	8860	4	19371	16035	17314
15	5	228	260	547	8849	5	9400	7452	8969
12	6	637	715	533	8846	6	9389	9023	9231
16	4	600	656	514	8834	4	18504	16649	15254
14	6	329	370	470	8828	6	14329	17769	18432
13	4	316	362	531	8794	4	15709	14601	16299
15	6	369	415	574	8772	6	30557	26929	32116
17	3	340	371	644	8772	5	6877	4532	7334
10	5	956	1070	609	8769	5	14619	17134	19387
15	8	1337	1464	634	8753	8	13796	12557	14899
14	6	225	245	750	8731	6	14541	14957	14186
12	6	711	788	721	8725	6	11519	10358	11350

15	5	189	219	488	8718	5	10761	8636	10107
18	9	655	744	854	8692	9	13920	12938	12359
17	4	195	219	540	8649	4	26345	22432	22824
16	6	735	837	630	8634	6	21665	20747	22575
15	10	1472	1640	734	8626	10	15229	13056	15034
16	6	287	314	503	8612	6	15718	10974	11262
16	7	782	880	644	8609	7	15359	13177	15229
16	3	271	305	668	8607	3	18095	14230	25407
12	5	273	302	580	8596	5	9595	10958	9903
13	4	163	180	580	8586	4	9350	8748	9222
15	5	733	814	498	8544	5	10032	9222	10091
12	4	182	204	481	8543	4	14550	13082	13784
12	6	567	639	699	8539	6	6137	5633	5908
12	6	428	483	505	8509	6	5626	3205	3390
15	7	1065	1205	620	8504	7	20235	17907	18196
15	5	387	422	533	8494	5	11021	8117	8801
16	8	1016	1113	580	8492	8	11395	9981	12381
17	5	124	136	843	8490	5	17417	19005	16124
11	7	368	404	840	8479	7	10096	8444	9313
14	11	3400	3864	806	8472	11	19595	14483	13453
13	3	130	148	999	8472	3	14719	11845	10489
10	6	345	371	587	8469	6	19777	19020	18484
13	8	476	490	1045	8468	8	3706	4181	3666
13	3	544	561	885	8453	6	1721	1730	1704
15	4	526	603	607	8452	4	20600	16017	15188
14	7	451	507	507	8435	7	11517	11843	12507
13	6	1196	1390	853	8363	6	11610	10286	13336
13	4	165	191	547	8361	4	11379	8242	9061
11	7	773	878	692	8345	7	12246	12434	11509
11	5	133	143	456	8337	5	2757	2336	2627
13	6	1330	1484	705	8337	6	14791	10999	10840
17	4	131	141	841	8332	4	16858	12306	17225
15	9	1276	1442	578	8325	9	24449	19495	21368
13	6	794	860	897	8313	6	17193	14614	13688
16	7	1050	1179	816	8296	7	19104	18886	17659
14	9	590	657	621	8278	9	18584	14844	18401
10	5	333	357	884	8274	5	9474	9431	9684
14	4	427	472	799	8272	4	4796	4230	3901
15	5	273	301	706	8261	5	18145	12322	17253
17	6	652	742	850	8260	6	15734	16213	15758
16	5	136	144	470	8250	5	15427	10576	18713
15	5	151	167	797	8246	5	26213	21579	25223
14	5	789	888	690	8197	5	9979	10096	10184
14	4	167	177	882	8188	4	15282	14697	14673
14	4	261	286	1080	8184	4	12816	11947	17053
10	3	182	207	658	8172	3	16105	10208	10947
12	4	184	211	736	8171	4	21509	17483	19563
16	10	1500	1683	475	8152	10	30100	29122	28419
12	6	581	624	554	8116	6	13689	11785	11893
15	7	1717	1939	505	8080	7	9947	10194	8853

13	4	1128	1286	1081	8078	4	10808	10160	8592
13	6	411	463	755	8069	6	10861	9530	9495
12	2	149	170	1051	8065	2	8479	7122	9035
12	3	519	574	503	8065	3	6126	7851	7132
12	5	150	171	413	7985	5	4506	6554	4458
14	7	2652	2888	465	7980	7	17102	15109	16632
15	6	278	318	623	7979	6	15692	11984	13624
15	7	318	358	684	7978	7	20312	17929	17503
11	5	1800	1983	851	7975	5	10178	10384	9476
19	2	56	66	913	7940	2	17644	22718	30933
15	7	890	1007	553	7933	7	24986	22833	23379
13	7	418	471	548	7919	7	5391	2447	4647
15	9	754	839	720	7913	9	18903	17108	17634
12	1	133	132	832	7910	1	10698	11458	9585
15	6	238	274	521	7894	6	20986	12534	15068
15	8	331	379	739	7875	8	16775	12244	17813
12	6	360	393	695	7868	6	25243	16757	19820
13	8	753	851	712	7853	8	4938	5087	4886
13	5	417	475	733	7846	5	10716	9895	12923
17	6	215	248	892	7842	6	20772	16228	18109
10	5	327	370	689	7841	5	13847	13405	18111
16	6	1138	1262	525	7836	6	14143	12599	12315
16	3	183	210	1005	7835	3	24288	19200	18612
11	4	119	132	586	7827	4	13065	9622	9472
14	7	481	545	541	7818	7	28220	25520	27296
14	9	707	780	526	7817	9	24177	21593	22880
12	4	269	295	679	7814	4	18627	17127	16612
11	6	276	289	768	7810	6	12128	11404	10693
15	5	490	551	625	7795	5	13134	10779	13114
12	4	362	400	597	7781	4	10450	10578	11043
12	4	281	314	505	7769	4	4351	2988	4130
14	6	616	692	517	7761	6	32569	27010	26565
12	4	747	818	762	7757	4	18967	18259	18938
10	5	914	1006	844	7749	5	2471	2633	2692
13	3	246	271	928	7729	3	23658	15880	14670
12	5	1636	1767	632	7723	5	16867	13368	15980
15	8	901	1013	615	7719	8	25400	23456	21611
13	4	272	285	780	7713	4	26830	23572	25709
12	7	1334	1476	715	7701	7	3537	3497	3617
14	4	1072	1192	793	7698	4	4410	4372	3990
14	7	524	585	681	7696	7	24745	25692	26174
14	5	312	357	642	7695	6	11876	7407	11551
10	3	216	223	493	7677	3	16002	15348	12752
16	6	412	462	549	7676	6	15851	14090	16156
15	5	284	316	853	7674	5	13814	14238	14128
15	7	483	540	695	7654	7	13656	12185	11989
13	3	215	246	661	7644	3	15875	14902	11721
14	3	987	1098	758	7621	3	3004	2897	2740
12	5	138	166	505	7604	5	12518	10311	9855
12	5	507	557	475	7598	5	3228	2893	2741

15	10	1194	1291	623	7596	10	33642	26385	28933
12	7	679	762	928	7581	7	8600	9563	8731
11	3	221	248	465	7581	3	9222	12602	12281
12	7	1457	1650	599	7565	7	11728	13286	12051
12	7	553	629	536	7553	7	11905	10413	12296
13	7	423	488	574	7552	7	10560	9315	9332
12	5	288	317	974	7547	5	10805	11324	9628
12	5	1102	1240	473	7546	5	4951	5534	6036
11	4	534	585	578	7540	4	6895	5697	7159
13	7	619	696	692	7538	7	14572	12852	14378
11	4	474	519	661	7502	4	5267	5110	5032
12	6	374	419	514	7494	6	10632	10482	9635
11	5	222	248	526	7469	5	15208	12872	12556
11	5	307	345	554	7449	5	11969	7255	13197
13	7	208	236	623	7448	7	6298	5368	6052
9	3	277	323	816	7442	3	12799	13788	12441
13	4	989	1089	578	7437	4	10423	9483	9343
13	8	1043	1167	903	7427	8	28044	24269	21344
13	5	191	201	775	7425	5	16835	20232	19325
10	4	534	598	718	7423	4	7645	8309	9280
13	4	475	500	529	7412	5	2919	2025	2152
13	5	404	452	648	7390	5	21285	21428	19547
12	7	434	475	670	7389	7	6654	6111	6811
10	5	421	466	815	7375	5	10242	10784	13867
11	5	909	1009	819	7372	5	11152	10809	9693
13	2	193	219	703	7357	2	8677	8093	6492
12	5	383	426	638	7343	6	9636	11239	9319
11	4	270	306	914	7333	4	7158	5776	5623
15	4	124	149	970	7313	4	23511	20173	30243
13	5	167	195	914	7311	5	16602	14272	15701
13	6	373	426	790	7306	6	9258	9055	11097
12	7	811	933	693	7295	7	8303	8221	7067
12	6	528	581	560	7294	6	14157	16127	13113
12	6	650	739	590	7292	6	8629	8021	8646
11	8	338	379	507	7280	8	3853	4516	4308
11	4	311	346	613	7278	4	2061	3111	2789
13	1	357	410	683	7272	3	4405	4873	4354
13	5	184	210	665	7254	5	13455	8452	10128
9	3	170	192	473	7248	3	7450	7901	10660
11	6	184	208	580	7237	6	18245	17822	18024
14	5	705	813	743	7204	7	5414	5622	5447
13	5	435	510	661	7185	5	8604	7868	8958
14	7	633	699	505	7174	7	17390	18299	16393
13	4	309	336	976	7169	4	15330	13812	17334
12	6	736	826	720	7163	6	15767	13919	15750
10	4	175	201	677	7128	4	9318	9301	8853
11	5	713	809	711	7125	5	14544	13723	18480
9	5	332	370	576	7104	5	752	1008	786
12	5	1527	1688	642	7101	5	21376	18056	19068
12	5	574	623	648	7081	5	19115	19478	19432

11	5	550	596	742	7073	5	11570	11530	12658
9	2	377	426	487	7063	2	4982	5754	5027
9	4	184	205	488	7059	4	7829	5247	6842
11	4	336	346	887	7055	4	17977	15378	18632
11	5	934	1055	775	7047	5	7767	7133	9511
11	5	305	350	503	7042	5	25953	17499	25014
10	5	381	424	547	7040	5	16214	14661	14409
13	7	3408	3899	482	7029	7	8841	7719	8658
13	5	846	937	664	7028	5	11177	9066	8720
11	4	307	333	469	7004	4	13083	13658	13274
13	6	326	365	891	6993	6	31562	27268	25919
11	2	174	200	527	6985	2	23474	12038	18227
12	8	712	802	652	6973	8	2826	3172	2751
10	4	1422	1583	952	6970	4	9281	9495	9037
9	5	133	139	506	6944	5	9523	3377	8151
12	6	493	563	793	6935	6	6478	5360	6074
13	5	506	556	775	6921	5	13215	11417	12617
13	8	389	454	648	6917	8	17595	13751	15701
13	4	167	183	960	6915	4	33546	27367	33309
13	3	908	1020	611	6914	3	9097	11020	11052
11	4	624	693	582	6867	4	11063	11170	11173
11	4	608	679	787	6857	4	11920	8955	10762
13	6	642	704	493	6842	6	2956	2380	2855
12	4	591	641	547	6831	4	14514	11877	13748
9	2	719	791	679	6827	2	3681	4012	4213
11	6	1296	1442	681	6820	6	5773	4777	4348
14	5	241	261	850	6820	5	15997	17170	16940
9	3	369	395	496	6817	3	9495	8239	8708
10	2	160	182	643	6815	2	10638	8378	9694
12	4	205	234	679	6805	4	6108	4513	7043
15	4	508	596	679	6803	4	8014	7068	7248
13	7	1138	1256	613	6800	7	11812	11158	11469
12	6	411	470	724	6791	6	3644	4002	4047
12	5	210	236	521	6785	6	3300	3280	3109
9	2	438	491	609	6785	2	451	470	519
11	4	191	223	573	6783	4	19873	17666	20268
12	4	685	790	644	6770	4	11193	8764	11673
9	5	262	297	840	6767	5	4201	3297	4173
12	7	373	397	591	6766	7	8527	7695	9726
12	5	331	365	526	6756	5	9606	7971	9392
8	3	1034	1159	752	6719	3	1159	1160	1018
11	5	636	687	834	6710	5	7183	5288	5450
12	2	202	212	670	6705	2	14354	16483	17812
11	5	306	350	897	6683	5	7028	5414	5644
10	3	418	467	888	6681	3	9939	7540	9852
13	3	210	224	478	6677	5	25994	3605	29659
13	5	577	652	674	6674	6	14829	11630	12309
9	2	153	176	837	6660	2	3206	3417	3330
13	9	516	562	951	6654	9	10248	8803	10766
10	4	285	315	515	6637	4	12106	11054	10906

12	3	184	214	458	6635	3	4018	6173	3819
12	4	245	273	881	6634	4	13498	15271	13748
12	6	487	533	618	6623	6	14160	12026	13181
12	3	112	123	888	6617	3	9371	7839	7983
10	2	231	262	615	6605	2	8970	9530	11631
12	4	582	647	508	6605	4	12032	10739	10361
12	5	226	254	832	6601	5	15847	11899	13478
10	6	320	357	843	6599	6	20065	21775	17597
14	6	467	536	731	6597	6	22754	15555	18454
16	7	361	409	519	6582	7	12440	7600	7402
11	5	221	247	935	6575	5	10920	9572	12910
15	5	303	342	888	6575	5	14692	11833	11475
11	3	180	204	680	6569	3	8928	6093	7349
14	3	162	188	1023	6565	3	12467	11569	11820
13	6	531	610	775	6562	7	10902	10833	9107
10	6	508	585	492	6556	6	13383	8804	12081
12	6	992	1091	629	6549	6	13014	11055	12963
9	5	198	221	668	6548	5	5228	6319	5355
11	4	383	447	784	6542	6	3682	3489	3376
12	6	750	837	500	6540	6	14596	12030	14325
12	5	547	614	730	6538	5	18981	16917	18123
8	2	597	662	834	6524	3	3358	3012	3328
12	6	512	522	977	6520	6	7106	5035	5666
11	3	497	576	892	6511	5	1906	1191	1221
11	6	633	718	623	6500	6	11500	10482	11575
11	2	175	199	687	6491	2	15613	14525	14850
10	4	234	271	654	6489	4	7337	6428	7268
8	4	646	662	1059	6483	4	3395	2977	2713
11	5	285	313	692	6475	5	9625	7305	8165
16	1	232	252	474	6474	1	13400	15559	18452
11	5	541	624	720	6459	8	7317	7752	7751
9	5	82	95	963	6456	5	1999	1361	1804
11	2	125	136	919	6447	2	14656	11677	12942
11	3	159	180	679	6438	3	13683	11521	12424
10	7	465	518	576	6435	7	9563	9755	9529
11	5	539	583	724	6433	5	7990	5902	7947
10	5	325	346	762	6425	5	14449	14753	15165
10	4	1794	1889	802	6400	4	5656	5918	6113
11	5	373	416	818	6388	5	11807	8860	10272
11	5	447	502	947	6385	5	14586	12285	14334
9	7	498	565	558	6375	7	5699	4337	5855
11	10	8614	9852	464	6353	10	6359	6240	5767
11	5	212	237	459	6337	5	3518	2633	3159
12	7	932	1019	651	6322	7	18632	16128	16310
10	5	565	621	701	6315	5	8956	7447	7004
12	7	609	695	742	6301	7	13636	12592	12478
12	6	409	449	717	6297	6	4251	3741	4089
10	3	439	494	623	6283	3	10682	8768	7560
9	5	304	351	799	6278	5			
9	2	358	381	679	6266	2	4821	4619	4314

8	4	636	714	847	6265	4	16648	11816	12312
12	5	293	311	774	6263	5	4136	4228	4860
11	6	310	346	954	6248	6	15072	13310	12520
11	7	2099	2306	834	6246	7	13833	11998	10952
11	2	592	697	555	6233	2	5802	4752	4744
15	7	625	702	563	6215	7	17959	12013	17349
9	6	751	848	477	6205	6			
11	8	5536	6031	609	6187	8	6131	7219	6667
9	3	140	149	1083	6175	3	10440	9144	11162
9	3	631	687	976	6173	3	11338	10070	10351
11	5	363	421	640	6165	5	15564	12568	13281
11	5	242	258	894	6146	5	21339	17710	18952
12	4	475	521	784	6132	4	25216	20718	21658
13	3	110	123	677	6128	3	13850	14582	14267
10	4	624	688	761	6098	4	3919	3403	3074
10	4	215	236	491	6088	4	14109	11008	11473
10	3	185	186	847	6083	3	9955	9528	9280
13	4	1739	1908	879	6082	4	7421	5852	6176
10	8	517	581	664	6064	8	3457	2946	2723
11	5	558	618	526	6046	5	12401	9333	11626
10	2	93	105	548	6040	2	19382	11382	15489
10	5	582	667	629	6039	5	28062	22387	23082
10	6	494	550	553	6039	6	10839	9889	9190
9	4	596	656	489	6036	4	7849	7849	7512
10	3	160	176	557	6029	3	9205	7404	9473
13	6	483	533	562	6028	6	31478	27409	30504
11	6	375	413	597	6025	6	10729	8660	9539
10	7	439	504	819	6020	7	25897	17756	22777
10	4	468	510	765	6018	4	8534	7031	8749
10	8	2190	2391	696	6015	8	11250	10397	10813
8	3	448	503	544	6012	3	9744	9422	9969
12	3	152	176	1083	5995	3	11210	9114	14339
9	5	592	635	913	5993	5	9282	7760	7662
10	2	243	271	1165	5984	2	16549	12902	12606
12	4	360	422	539	5982	4	9717	7891	8524
9	3	204	235	1083	5982	3	10306	8376	10950
11	6	2160	2398	481	5979	6	4644	4285	3452
9	5	264	284	463	5942	5	2381	1990	2245
9	6	631	692	657	5941	6	15359	14905	14313
10	5	944	1053	609	5931	5	8983	7562	7509
9	2	372	419	654	5915	2	6090	5559	5805
10	5	285	318	966	5875	5	6193	4668	5035
11	4	211	245	907	5861	4	18134	16141	16019
10	6	307	338	655	5860	6	21117	21121	21078
13	5	128	147	517	5855	5	25671	23128	24026
13	4	321	368	605	5855	4	6674	4797	5896
13	6	280	304	910	5855	6	10347	9516	10011
8	5	417	482	606	5851	5	9493	7496	8411
10	4	315	339	957	5847	4	17500	19747	17697
10	4	154	177	581	5832	4	26676	30710	13307

9	3	118	129	881	5819	3	11521	11683	11379
9	4	140	155	847	5813	4	11864	9220	9891
10	4	1798	1845	888	5806	4	2110	2239	2105
10	3	477	536	559	5802	3	16484	16443	15769
7	3	782	867	717	5795	3	4307	4246	4401
10	4	110	119	529	5790	4	9468	6766	7006
10	4	150	164	977	5787	4	7697	13675	9914
11	6	777	873	667	5773	6	8721	7236	8928
11	4	918	1014	501	5768	4	10187	12147	10603
7	3	207	232	736	5766	3	2749	2629	3142
8	3	309	355	554	5762	3	4308	3719	4330
9	5	496	559	681	5760	5	12761	12272	11537
9	4	220	231	439	5745	4	11953	10665	9432
11	4	348	397	494	5744	4	16829	16807	19248
12	3	388	423	496	5742	3	17781	18310	16269
9	5	631	701	637	5740	5	8826	7761	7837
10	4	294	330	643	5734	5	4235	3550	3701
8	2	228	238	976	5732	2	10335	8024	9659
13	3	127	141	785	5731	3	10712	9057	9630
11	6	690	794	664	5722	7			
11	5	520	584	644	5716	5	13630	12259	13827
9	3	292	320	442	5699	3	1651	1553	1081
8	6	750	809	510	5684	6	5006	6078	5246
11	5	319	354	977	5680	5	13710	10481	12319
13	4	282	311	695	5679	4	26838	20069	23030
10	4	457	500	720	5672	4	11849	10309	10260
11	4	228	247	599	5663	4	3195	3365	3566
9	3	504	577	854	5652	3	8057	8354	7855
10	3	395	447	705	5638	3	17706	17400	18385
9	7	943	1074	533	5623	7	9687	8621	10203
11	5	217	245	888	5621	5	12776	11462	11122
9	3	298	331	778	5617	3	19388	16831	17128
8	6	735	803	618	5611	6	3743	3471	3879
8	4	589	650	482	5611	4	14822	14000	13964
12	5	258	291	960	5608	5	12899	9389	10314
9	7	315	336	592	5596	7	15686	12495	14790
9	4	1149	1284	680	5583	4	10225	9362	10807
13	3	74	82	960	5580	3	16439	22734	19707
10	7	308	339	600	5573	7	10296	6518	9008
11	5	522	570	828	5569	5	17927	15645	14044
8	4	1373	1532	692	5568	4	5772	5430	5413
10	5	356	402	819	5561	5	13309	11033	10661
9	3	227	265	580	5560	3	8442	6504	6793
8	2	363	399	715	5560	2	1046	1132	1133
12	5	240	263	686	5541	5	18058	21217	19415
11	5	491	543	759	5540	5	10455	8596	8666
10	5	404	444	607	5524	5	8258	7374	7185
6	2	247	264	539	5518	2	5808	5453	6054
11	1	208	238	658	5503	4	5774	5371	5956
12	5	362	396	762	5500	5	11930	9278	10313

13	4	174	201	460	5493	4	16109	12012	12020
7	1	531	608	693	5489	1	1050	909	785
7	2	294	335	698	5484	3	3501	4017	3715
10	2	3331	3684	790	5477	3			
10	4	545	597	676	5470	4	14304	9977	13562
8	1	497	576	900	5465	3	8504	7560	5856
7	3	633	686	815	5460	3	4933	5862	5823
8	4	419	438	484	5452	4	264	269	339
11	8	393	431	777	5448	8	15062	10379	12850
9	3	250	293	435	5445	3	3567	2567	3541
10	4	216	251	664	5435	4	14216	14137	13849
10	5	336	385	610	5420	5	12957	11160	12666
10	5	376	415	805	5418	5	6852	6186	7582
11	6	511	575	690	5416	6	18458	12500	15111
9	3	221	246	520	5416	3	3641	2140	3671
12	7	532	599	566	5414	7	14546	12865	12264
9	6	460	524	803	5409	6	8716	6923	8393
10	3	153	166	906	5406	3	4147	4141	3890
9	5	528	581	686	5404	5	3164	3772	3612
6	3	262	303	558	5401	3	3193	2994	3117
12	2	95	109	594	5401	2	16759	13169	13294
11	7	860	985	941	5396	7	12543	11344	12356
11	5	451	480	753	5382	5	11883	10141	9946
9	2	262	268	415	5356	2	6279	6699	7865
6	4	1207	1338	494	5354	4	1192	951	1193
9	5	152	170	1172	5347	5	2874	2791	2858
9	4	1176	1337	702	5342	4	12510	11654	11223
10	5	221	237	906	5341	5	3751	2485	2984
11	4	208	237	496	5330	4	12815	7953	10032
12	3	148	169	564	5313	3	13832	8684	15320
10	5	124	136	718	5310	5	15001	19292	22760
9	7	799	885	658	5307	7	7628	7128	7658
8	3	325	371	758	5302	3	15393	12081	12121
9	4	1054	1204	481	5300	4	8568	8350	9996
6	2	1942	2001	860	5288	2	461	427	425
8	2	319	347	952	5286	2	12833	12628	15107
8	3	126	142	508	5278	3	13221	11119	13840
13	2	177	204	1155	5276	2	12444	10313	12275
12	4	359	420	644	5259	4	3824	3266	3325
8	5	400	444	923	5248	5	7922	7722	7065
7	4	742	865	555	5247	4	5937	5346	4908
8	4	1166	1297	541	5246	4	5092	4370	5212
8	4	1113	1211	928	5233	4	3125	2976	2754
12	5	463	523	614	5225	5	11463	9930	11105
8	3	153	168	496	5219	3	11947	7629	8044
8	3	527	578	576	5211	3	2915	2602	2621
8	4	149	154	931	5198	4	12070	6061	11414
9	5	726	811	679	5195	5	11945	10580	11776
7	1	321	363	538	5191	2			
11	5	461	512	673	5179	5	7528	7398	7345

11	6	317	345	529	5174	6	16562	14165	14521
10	3	177	212	1061	5173	3	9701	8403	10450
10	6	86	95	717	5170	6	15874	18373	20841
10	4	165	190	510	5170	4	9005	6443	7506
10	3	248	273	862	5168	3	16221	12167	12891
9	5	704	752	674	5167	5	8279	4920	8190
8	4	273	300	611	5164	4	2146	1843	2252
6	3	118	129	432	5158	3	950	1147	995
11	4	2162	2379	638	5148	4	7152	5110	6404
9	4	858	962	586	5148	4	10630	8893	9602
7	1	607	692	651	5146	4			
4	1	303	339	766	5140	1	3514	4112	3703
9	6	1334	1486	601	5131	6	12918	9762	11059
7	4	839	966	535	5123	4	7743	6282	6983
10	4	336	391	695	5119	4	7236	6610	9715
9	3	106	120	526	5119	3	30702	17337	23322
9	5	783	870	762	5115	5	5197	4902	5066
10	9	438	498	948	5112	9	2869	2632	3371
9	4	119	134	1032	5111	4	15375	12449	18363
8	4	554	615	581	5110	4	14917	9089	15191
8	4	326	339	500	5103	4	3306	2601	3345
9	2	123	143	1093	5091	2	14528	13625	13007
10	1	196	209	488	5086	2			
9	4	880	951	907	5075	4	2044	2417	2104
7	4	770	855	606	5073	4	18179	13471	15779
9	4	285	292	686	5069	4	8535	10334	11779
6	1	440	512	768	5066	1			
11	4	255	288	960	5058	4	11923	10498	11477
8	5	612	700	557	5057	5	2977	3137	3678
10	7	395	391	928	5041	7	3236	2235	2654
10	5	325	362	654	5038	5	16290	14600	13944
9	2	228	265	568	5032	2	4175	8855	5743
10	2	269	304	680	5031	2	15556	13654	12380
9	4	345	377	802	5027	4	5665	4970	5561
9	3	704	766	657	5016	3	4310	4187	4108
11	3	171	182	906	5013	3	14467	13951	15823
7	5	611	680	506	4999	5	12997	8275	9771
7	3	580	662	615	4967	3	13461	12984	13362
8	6	685	773	543	4960	6	3949	4577	3664
10	5	741	801	639	4960	5	2006	1560	1525
9	4	790	871	540	4958	4	6977	6712	6517
11	3	290	334	610	4956	3	6080	1576	1336
10	1	327	373	633	4954	3	2458	1374	1576
10	5	1409	1569	662	4948	5	15910	15864	14506
10	4	199	221	788	4946	4	6069	4120	5709
9	7	1750	1946	590	4941	7	10286	8851	8349
8	3	420	480	854	4937	3	8100	8054	10299
7	3	372	426	496	4936	3	6443	5755	7612
9	4	868	954	671	4934	4	8664	6643	7369
9	4	777	854	793	4933	4	2562	2256	2520

9	6	404	447	634	4929	6	27102	23854	26470
11	5	493	536	705	4926	5	20542	15831	14496
8	3	2816	3053	512	4923	3	11249	10938	10272
9	4	590	667	534	4918	4	13066	12901	13363
6	4	323	360	933	4917	4	9099	13672	10533
9	5	470	523	586	4914	5	9439	7488	8733
9	1	212	243	658	4911	4	4020	3291	3839
7	4	475	524	846	4908	4	6043	5904	5978
8	4	846	965	708	4904	4	5970	3614	4756
8	5	303	324	712	4901	5	9757	8461	9249
9	5	3383	3829	702	4898	5	6160	6134	5967
10	5	148	167	929	4893	5	16235	13557	13780
8	2	358	396	530	4892	2	10758	9057	7337
9	2	353	404	620	4889	4	9925	8184	8810
7	1	666	757	708	4888	4			
8	5	661	744	917	4886	5	10339	9239	9518
8	3	948	1088	527	4880	3	10409	8094	9069
7	4	182	205	885	4872	4	7057	6564	8114
8	4	251	276	530	4868	4	21208	15068	18003
8	1	392	417	1077	4843	1	310	362	333
10	4	364	405	652	4837	4	8479	5514	6507
9	3	303	342	576	4831	3	8612	7750	7718
7	5	286	325	581	4828	5	17753	11289	12784
10	3	176	192	955	4822	3	12896	13889	11631
8	4	349	384	667	4821	4	12432	11085	11447
11	2	218	244	559	4807	2	11536	13483	10893
8	4	2272	2546	574	4807	4	11976	10361	12046
7	4	210	244	615	4803	4	4682	4438	4771
6	2	1067	1199	498	4802	2	9611	8380	9224
8	4	583	639	450	4800	4	10009	8878	8666
9	5	5366	5911	600	4800	5	13083	11675	13240
6	2	487	525	739	4797	2	9556	9265	10150
6	2	791	882	1008	4797	2	1274	1458	1559
9	5	530	559	683	4793	5	2072	1989	1835
8	3	620	692	574	4782	3	2682	2047	2042
6	2	235	261	563	4776	2	3674	3470	3340
6	2	336	378	648	4775	2	3349	3937	2913
9	4	900	1040	772	4765	4	2285	2002	2935
8	2	212	235	500	4763	2	3977	3326	3708
10	3	208	239	878	4747	4	7287	6135	8549
9	5	1298	1434	477	4730	5	12328	11236	13562
8	5	1875	2058	549	4725	5	8316	8361	7991
8	4	306	336	664	4724	4	11047	12850	10617
9	1	353	380	470	4715	1	8343	8440	6189
9	7	412	451	846	4715	7	7937	5890	7260
8	4	502	564	635	4710	4	10248	8497	9450
7	3	279	308	510	4698	3	9299	8988	8990
11	5	409	467	577	4697	5	19517	15522	16077
8	4	869	942	876	4687	4	2909	2273	2387
8	4	521	591	750	4669	4	5170	4384	5010

8	1	3423	3789	837	4667	2	1794	1739	2282
7	3	286	324	834	4666	3	5856	5490	7092
8	6	477	535	706	4664	6	8513	7315	7450
9	6	391	444	756	4657	6	17899	13039	16142
6	2	233	256	465	4657	2	8000	8541	7471
11	5	124	146	1035	4654	5	10249	8011	9030
8	3	205	233	595	4642	3	10420	7154	7830
9	2	222	247	613	4636	2	12397	11164	16988
7	3	629	702	548	4624	4	11094	8576	9080
8	3	286	324	640	4622	3	7746	6809	7940
9	6	553	625	680	4616	6	3889	3743	4410
8	5	421	484	610	4610	5	2375	1918	2383
9	3	255	293	998	4593	3	11914	10815	9518
7	5	878	973	559	4590	5	1067	848	872
10	3	178	205	591	4587	3	14094	13127	18020
9	2	553	631	868	4576	4	5132	4856	4422
9	3	604	683	728	4573	3	8696	6784	6650
8	5	1343	1512	616	4564	5	11265	7477	11177
8	3	1131	1292	982	4560	3	1788	1800	1582
10	5	491	543	577	4559	5	13342	10450	12627
9	3	242	258	657	4558	3	8182	5560	5810
5	1	221	244	673	4549	1	11233	6603	7745
7	6	737	808	744	4544	6	6896	4778	4118
7	4	1158	1291	526	4541	4	10647	9793	9843
10	2	539	597	705	4539	3	4220	3481	4092
8	3	147	162	999	4534	3	4258	4178	4782
8	4	1228	1373	652	4530	4	12004	9398	10901
7	4	1138	1264	724	4528	4	5371	4691	4322
9	6	1642	1838	506	4519	6	5165	5712	3908
8	4	279	293	872	4514	4	7177	6120	5914
8	4	444	515	545	4511	4	9503	6716	8102
6	3	429	483	493	4502	3	1533	1374	1312
11	4	114	122	957	4499	4	7059	6667	10027
9	6	999	1134	857	4495	6	6709	7306	7132
7	3	275	292	829	4492	3	19563	10218	14321
10	6	796	879	473	4477	6	14007	13701	12464
9	2	206	236	693	4474	2	5597	5070	4878
8	4	494	546	865	4474	4	15550	11758	13350
6	5	438	498	680	4471	5			
9	4	796	874	582	4467	4	3891	3847	4799
9	4	408	467	547	4467	4	7814	5647	8396
7	4	323	384	470	4455	4	7324	5958	7982
6	2	339	373	747	4446	2	9894	9257	8645
10	5	359	400	775	4442	5	11463	8492	10078
7	2	397	429	785	4439	2	4165	3957	4476
8	4	713	809	552	4438	4	10798	9162	11159
8	4	321	347	958	4431	4	6183	5207	5495
11	3	143	163	677	4430	3	11460	9313	11015
7	4	729	827	497	4414	4	11026	9397	10215
9	5	448	508	728	4411	5	11462	9619	12299

9	5	552	616	866	4409	5	12956	9404	11192
8	4	155	169	712	4408	4	14043	7752	9742
6	3	836	905	510	4407	3	1937	2193	1881
9	4	387	427	769	4405	4	15415	19337	14032
8	5	1308	1434	679	4399	5	7883	9305	7824
11	4	1064	1192	497	4397	4	11615	13274	12031
9	5	65	74	1037	4386	5	10864	9989	10358
9	4	1474	1664	611	4385	4	14496	13069	14252
8	3	253	279	679	4383	3	4678	3358	4030
8	4	957	1097	843	4367	4	8554	9388	8444
5	3	365	420	624	4367	3	9121	7308	7475
8	3	189	212	655	4365	3	10406	9811	11365
8	2	331	376	891	4356	2	7713	8098	8869
8	2	89	102	774	4347	2	13974	8093	9539
8	2	115	129	482	4345	2	5791	5041	5840
7	3	585	652	606	4344	3	12474	10652	11176
10	5	1298	1406	578	4339	5	20539	17634	17530
7	4	417	471	655	4335	4	12713	10292	12364
9	4	199	225	692	4333	4	10127	8571	9067
8	3	674	749	721	4318	3	9316	9123	8913
8	3	265	298	620	4306	3	29430	29053	31401
9	6	904	998	654	4304	6	10961	9741	9921
7	2	423	454	718	4304	2	900	977	834
7	2	139	161	747	4302	2	6680	4220	8355
7	2	329	360	646	4289	2	7571	6166	6819
6	2	143	164	569	4286	3	1673	2878	2389
7	3	797	912	853	4285	3	8966	6425	7367
7	3	237	271	572	4283	3	9431	7410	9919
9	2	129	147	992	4283	2	6600	5274	6604
9	5	351	384	692	4282	5	8940	6811	7606
7	6	2338	2551	891	4276	6	859	648	898
7	2	155	178	573	4267	2	2328	1250	2217
8	6	946	1058	683	4253	6	6676	6728	5461
9	3	83	93	721	4251	3	2266	2024	1508
6	2	126	135	600	4235	2			
11	3	424	480	586	4235	3	13947	8496	11771
7	4	4015	4220	651	4233	4	6246	5262	5189
7	4	436	475	630	4232	4	1228	775	1201
7	2	546	600	701	4229	2	4450	3094	3468
10	6	184	206	831	4227	6	5147	4234	4346
7	4	1228	1381	649	4223	4	20167	18876	19603
9	5	432	500	648	4214	5	262	111	162
8	4	481	545	639	4205	4	12293	15606	10764
6	4	456	520	553	4202	4	9712	7420	7375
8	4	1720	1905	821	4199	4	10003	9673	8327
8	4	1241	1371	574	4196	4	7424	7462	7950
9	3	151	174	1070	4194	3	3986	3801	5075
5	1	2387	2691	739	4189	1	350	501	519
7	4	182	205	661	4187	4	17281	15922	16553
6	2	952	1090	665	4180	2	2293	1946	2156

6	4	445	493	870	4179	4	4443	4352	4406
8	2	280	304	676	4172	2	7164	6126	9827
7	4	443	509	531	4166	4	20625	14808	20096
7	3	2188	2452	592	4153	3	4324	3879	4030
6	3	260	283	686	4151	3	3340	2993	3807
9	3	1388	1516	644	4145	3	9825	8476	8213
9	4	1574	1741	925	4141	4	4031	3400	2950
6	3	1019	1137	505	4125	3	937	834	1068
7	4	1276	1413	765	4124	4	2685	2772	2519
8	5	998	1067	654	4111	5	8749	8087	8077
5	2	582	660	592	4107	2	4706	4808	4167
7	4	831	939	813	4106	4	2321	1862	2410
7	4	529	570	938	4103	4	4135	4456	3253
9	5	550	620	655	4103	5	7684	7037	6264
6	3	860	953	515	4102	3	7778	7742	7367
8	3	884	996	539	4094	3	11524	11315	9318
6	3	228	261	510	4081	3	8208	6672	6332
10	4	83	94	873	4076	4	4394	4136	4258
6	4	403	456	848	4075	4	6287	5170	6654
7	4	1153	1271	613	4070	4	3833	2946	3491
8	5	735	838	960	4069	5	9129	7281	7209
8	5	250	267	717	4069	5	8630	6603	10497
7	3	137	150	482	4069	3	8681	6393	6159
9	4	309	351	714	4058	4	8612	5532	8155
8	3	134	158	1095	4058	3	7675	6991	7072
8	1	193	210	510	4056	5			
8	3	373	415	857	4045	3	4020	3802	4357
8	2	259	290	505	4034	2	5478	3150	4956
10	3	90	93	859	4020	3	8765	9448	13876
7	3	1027	1192	788	4019	3	13865	12243	11753
8	4	428	471	547	4016	5	4831	4578	5335
7	4	400	457	602	4016	4	3699	2669	2498
6	1	133	150	730	4012	1	9837	9707	9080
7	4	1459	1670	714	4011	4	4169	3449	4278
9	5	919	1027	526	4011	5	11104	10052	9860
8	4	386	426	505	4009	4	7419	6138	6624
6	2	327	360	686	4000	2	7070	7449	6596
6	1	183	202	932	3996	1	7100	6472	7389
8	3	234	266	625	3989	3	10245	9959	8553
7	5	295	321	860	3986	5	20211	13771	15060
6	4	929	1034	529	3982	4	11313	8646	9660
8	3	327	366	714	3978	3	8624	6889	6925
8	1	215	224	712	3975	1	1344	1415	1356
6	3	666	774	539	3973	3	3457	3462	4228
6	3	315	349	525	3968	3	1911	1476	1795
6	2	129	126	873	3965	2	5085	4102	4718
6	4	307	330	903	3962	4	10809	11649	10454
5	3	383	419	488	3960	3	5394	4280	5512
6	3	694	792	609	3945	3	3705	3633	3397
10	5	264	302	1119	3937	5	12662	10328	13255

6	6	1929	2127	643	3936	6	14796	14038	12655
6	4	551	604	957	3936	4	7878	6091	7714
6	1	157	160	897	3935	1	6694	5367	4657
6	2	433	492	654	3920	2	7518	7616	7143
4	2	503	574	558	3919	2	3085	2184	2794
6	5	933	1023	572	3915	5	1394	1623	1581
7	4	875	974	717	3910	4	1595	1363	1229
8	2	469	502	703	3901	2	6599	7914	7722
8	5	708	805	696	3896	5	5914	5301	5680
8	2	165	177	790	3892	2	2096	1904	2110
7	2	471	519	806	3891	2	13052	16363	15216
8	3	200	228	539	3889	3	8710	7786	7325
9	6	1658	1905	698	3889	6	2616	2833	2106
5	3	222	253	614	3886	3	10584	13049	11047
8	3	665	735	712	3885	3	13633	12458	12091
6	2	134	150	933	3877	2	5306	3881	5469
7	4	151	172	1051	3867	4	21408	17223	21300
6	5	1088	1204	640	3865	5	8665	7640	8328
7	5	1065	1135	850	3864	5	2900	2292	2436
8	2	132	144	1024	3859	2	7557	6701	4605
9	4	150	170	705	3858	4	8580	6379	7370
6	2	318	345	799	3852	2			
7	4	394	446	481	3850	4	5887	4877	5222
8	5	631	709	783	3846	5	10825	9260	11079
7	5	505	554	662	3846	5	4991	4737	4763
8	1	186	198	859	3844	3			
9	3	322	357	647	3843	3	6846	4814	6120
9	5	381	432	526	3823	5	17258	15375	18442
7	5	1393	1584	708	3816	5	2043	1901	1973
8	3	546	613	765	3815	3	11764	8266	9338
6	3	1617	1780	448	3814	3	8000	8289	7127
8	3	1287	1483	709	3811	3	8618	8289	6938
6	2	105	115	456	3807	2	4139	2215	3000
8	4	517	563	505	3805	4	3984	3667	2545
6	4	241	275	797	3800	4	11587	11206	12372
6	4	358	411	633	3797	4	3448	2770	3193
6	3	839	931	667	3791	3	5049	4678	4210
6	2	300	331	667	3789	2	9830	6661	8410
9	4	375	422	662	3787	4	12918	10346	10779
7	3	290	323	683	3782	3	11220	10291	9064
6	4	875	968	787	3780	4	8337	6807	8194
5	4	296	305	907	3776	4			
8	3	519	554	680	3773	3	2608	1385	1889
7	4	690	794	715	3769	4	11268	9674	9339
6	4	235	254	550	3750	4	782	737	1857
7	3	128	137	615	3743	3	8257	6766	8141
7	3	175	192	911	3742	3	10432	6383	9232
9	4	251	284	526	3728	4	8431	7832	9134
5	2	122	127	743	3726	2	4886	4212	4627
8	4	1184	1249	594	3725	4	5468	4202	4218

9	4	2103	2379	778	3724	4	15557	12114	12279
8	3	2036	2266	624	3718	3	6240	4826	5987
5	2	641	713	567	3716	2	2044	1943	1916
7	3	301	337	454	3706	3	9778	9266	9914
8	3	415	453	587	3694	3	7237	5357	6928
7	4	471	533	648	3693	4	7828	7002	6458
8	4	136	160	1100	3686	4	17990	19904	22499
7	4	564	625	677	3676	4	1956	2057	2160
7	4	1235	1401	658	3655	4	6452	4973	6384
6	2	396	430	527	3654	2	6288	7217	4310
6	3	390	427	544	3650	3	6432	6248	5758
7	4	594	656	947	3636	5	10936	9857	9504
5	2	245	261	468	3633	2	7292	6468	7663
6	3	282	312	531	3630	3	5767	4318	4702
6	2	1024	1150	503	3628	2	6213	4506	4917
4	1	597	667	739	3627	1			
5	3	881	975	555	3626	3	5157	3843	3976
6	3	271	309	637	3620	3	4143	3467	3927
6	3	2631	2912	910	3617	3	3677	3201	3098
6	3	1334	1505	648	3611	3	1224	1029	1198
7	1	119	129	853	3595	3	5229	3303	4331
9	4	292	332	785	3585	4	8451	5734	8175
6	3	572	632	838	3578	3	9404	7116	7445
7	3	374	420	536	3578	3	20021	12062	11708
8	6	2603	2864	744	3573	6	5626	5479	5495
8	4	1309	1471	632	3572	4	6550	6337	6692
7	5	750	813	637	3563	5	7155	6521	6851
6	3	950	1066	574	3552	3	4171	3746	3707
7	1	488	520	550	3549	1	7503	6405	5405
8	2	95	107	844	3541	2	7544	7386	7017
6	5	422	466	548	3540	5	10994	7255	7989
8	4	465	514	793	3534	4	8481	6601	7652
6	3	375	446	524	3531	3	11266	6691	10802
7	4	186	213	906	3529	4	5599	5180	4747
7	4	716	801	829	3528	4	1759	2201	2099
5	3	262	287	587	3524	3	5206	4913	4483
6	3	454	504	799	3523	3	1010	732	1800
7	5	1227	1409	762	3510	5	10966	10060	9414
6	3	362	412	961	3505	3	12688	8400	12833
7	1	75	81	784	3502	1	8319	8798	7890
6	4	498	561	887	3492	4	4659	4339	4902
5	4	2992	3384	602	3488	4	4439	4764	3962
6	3	328	372	585	3488	3	4317	2995	4484
5	3	334	374	895	3487	3	5733	5824	4764
6	2	105	114	539	3485	2	368	431	325
5	3	211	237	903	3481	3	8184	9022	5919
8	5	527	591	660	3472	5	9317	8076	9667
7	4	195	228	1061	3469	4	5280	4461	5447
6	3	382	426	679	3468	3	6987	5328	5722
7	4	1264	1355	577	3466	4	4113	3872	4337

7	2	132	145	516	3465	2	8792	6211	7908
6	2	656	719	807	3450	2	4752	4644	4348
6	3	1121	1288	665	3441	3	3225	2760	2698
6	4	698	790	775	3435	4	4868	3864	4894
6	3	222	251	709	3433	3	6326	4276	5275
6	3	1182	1337	838	3416	3	4267	4086	3892
5	4	280	315	909	3410	4			
4	2	462	523	840	3401	2	7827	7206	6757
5	3	783	892	844	3400	3	9858	10201	8528
7	2	270	306	1039	3399	2	12475	10967	10367
6	4	232	264	477	3398	4	3023	2230	1775
7	6	538	620	715	3395	6	3961	3975	4559
8	4	299	338	618	3395	4	6963	6601	5259
5	3	1521	1656	781	3391	3	4607	3918	4337
6	4	648	719	715	3388	4	9529	7294	8778
6	4	446	503	520	3388	4	4588	3652	3842
6	3	823	870	812	3387	3	5767	5322	5241
6	3	390	427	623	3379	4	6695	2680	7552
7	3	70	79	597	3353	3	5597	3459	4938
7	3	945	1057	966	3352	3	3630	4064	4287
6	2	300	328	470	3351	2	12496	12083	12945
7	5	83	88	816	3351	5	11252	12551	22033
6	3	1887	2085	758	3350	4	1552	1247	1341
6	2	693	776	917	3349	2	9278	7815	7287
5	3	1123	1217	788	3348	3	1107	928	1008
6	4	323	367	692	3348	4	5018	4982	5328
6	3	217	246	983	3346	3	8336	4102	6883
4	3	180	201	569	3344	3	3301	3821	3159
5	2	502	567	519	3341	2	1278	1613	1257
5	3	518	597	891	3338	3	4825	4704	5458
5	2	135	141	970	3335	2	2269	1638	2116
9	3	169	193	794	3333	3	9188	8021	9166
6	4	233	263	790	3331	4	5775	4396	4073
6	5	2657	2931	630	3328	5	8479	6637	7327
5	3	878	1013	538	3328	3	903	925	1100
4	3	579	647	541	3314	3	2785	2397	2648
6	2	126	146	677	3310	2	4880	4737	6444
7	3	107	125	999	3300	3	10964	9428	9162
7	3	481	528	554	3299	3	4707	2288	3663
8	4	389	438	865	3293	4	10046	9030	7902
6	2	240	268	536	3287	2	5388	4625	3815
6	5	131	152	803	3287	5	23661	20026	19580
7	4	793	862	660	3281	4	837	908	1075
5	5	289	332	711	3275	5	2437	2437	2264
7	4	233	261	627	3275	4	9419	8245	8693
8	2	136	152	526	3272	2	3860	2872	4092
6	2	772	895	673	3269	2	9438	6899	8446
5	4	458	529	695	3269	4	4309	3805	3363
5	3	2065	2356	848	3258	3	1978	1849	1596
6	3	545	625	629	3256	3	5460	6649	4757

6	2	441	488	1033	3253	2	6788	6328	6424
5	3	462	492	966	3250	3	4537	4055	4125
5	4	254	289	870	3245	4	4869	3890	3989
5	2	348	370	831	3244	2	3479	3451	3410
6	3	220	243	866	3242	3	11664	8375	9555
6	3	139	160	724	3242	3	4749	5014	4796
7	3	478	539	569	3236	3	9250	6939	8485
7	3	424	486	547	3232	3	9635	9494	7939
7	4	1536	1708	543	3222	4	2604	2286	2187
7	2	317	348	585	3219	2	11837	7686	8744
6	3	647	724	709	3218	4	2777	1683	1930
4	3	286	323	648	3215	3	6842	6426	6638
5	2	499	556	632	3214	2			
6	3	880	974	898	3210	3	8841	5057	5220
7	2	204	242	1137	3210	2	20671	15611	21163
6	1	119	128	920	3209	3			
4	2	1130	1274	516	3208	2	2785	2754	2940
6	3	163	185	506	3204	3	3087	3089	3210
5	2	347	378	711	3199	2	1400	1548	1400
5	3	949	1036	825	3194	3	2874	2132	2336
5	3	994	1099	717	3193	3	3471	3585	2750
6	3	671	770	638	3187	3			
7	2	193	213	680	3180	2	8012	5715	8438
4	3	91	108	963	3176	3	6833	5987	5555
6	3	564	614	850	3172	3	4307	3792	4617
4	2	1314	1444	683	3172	2	463	468	405
5	2	189	220	1103	3167	2	2381	1923	2078
4	1	417	486	818	3163	1	5852	5852	5624
6	4	614	685	857	3163	4	8043	8323	8987
6	3	465	485	512	3158	3	3254	2897	2790
7	4	726	825	724	3158	4	11242	9478	9434
6	4	969	1084	900	3157	4	8941	8983	7282
5	4	364	397	904	3157	4	3153	2336	2822
5	2	352	388	566	3156	2	4630	3188	4364
5	4	473	520	774	3153	4	10943	9503	10991
6	4	265	306	592	3144	4	11609	11248	11751
6	4	997	1038	426	3141	4	9405	6671	8141
5	3	400	461	913	3133	3	646	542	609
5	4	1130	1251	828	3129	4	6555	6132	6453
4	1	204	242	1144	3128	1	5591	5019	4980
5	4	343	393	780	3125	4	9125	6571	7628
5	2	382	419	500	3124	2	5194	4363	4742
5	2	590	682	709	3121	2	7934	7476	6723
5	2	567	630	747	3113	2	2058	1776	2195
7	5	1293	1425	774	3112	5	5143	4778	4114
5	2	495	557	522	3110	2	7875	6481	7125
5	2	123	132	496	3103	2	11971	11268	10975
5	3	1506	1677	901	3098	3			
6	5	331	360	799	3093	5	8325	6247	7079
6	2	640	694	525	3090	2	10593	8323	9235

5	3	210	244	510	3089	3	2854	2575	2431
4	1	194	217	848	3084	1	1595	1652	1517
4	1	365	410	834	3084	1	748	643	696
5	3	143	161	1058	3083	3	7450	6331	5628
5	2	176	199	715	3079	2	4023	5627	3820
4	3	458	528	739	3077	3	2662	2729	2627
6	3	256	271	771	3069	3	4939	4302	5108
7	2	341	394	1124	3066	2	6175	6206	5725
6	4	209	239	954	3056	4	8277	8294	8044
6	2	643	677	769	3056	2	4465	3982	3592
4	3	2141	2446	591	3055	3	5355	4429	4170
4	1	124	137	600	3054	1			
4	1	127	126	942	3042	1	161	138	165
6	3	845	925	582	3039	3	9642	9725	9152
5	2	416	468	805	3036	2	712	619	754
6	2	179	202	692	3033	2	4016	3222	3179
4	1	662	742	512	3027	1	1623	1882	1696
4	3	464	512	611	3026	3	11180	10637	13106
5	5	376	431	517	3026	5	1865	1387	1716
5	3	720	811	582	3018	3	2501	2495	2064
5	2	346	373	595	3018	2	4391	3627	4032
7	1	405	440	827	3014	2	4415	4498	4122
7	1	478	525	788	3000	1	5079	4208	3044
7	2	429	473	784	2995	2	7789	7224	8020
6	4	682	766	559	2990	4	10476	8333	9160
5	3	539	597	574	2988	3	3288	2702	2563
4	1	147	174	491	2988	1	3621	4191	5478
6	2	531	615	838	2985	3	2516	2845	2189
6	4	381	430	706	2982	4	12608	7385	10011
6	4	1201	1401	774	2973	4	9951	7604	8865
5	3	318	356	482	2968	3	2009	1252	1717
5	1	224	253	615	2966	1	9253	7409	10080
6	2	882	986	796	2966	2	10234	8951	9086
5	2	406	467	721	2964	2	4902	4691	6092
5	3	215	247	519	2955	3	6452	5222	5516
5	2	318	374	737	2949	2	5403	3811	4671
5	3	332	372	699	2946	3			
5	3	321	354	784	2945	3	10896	9283	10078
6	3	204	235	686	2941	3	13639	8201	10187
6	2	125	135	601	2937	2	4086	1979	2812
6	3	419	483	572	2934	3	15063	13515	13826
6	1	203	225	660	2927	1	8563	7039	7123
5	2	2022	2288	693	2921	2	7675	6961	7946
3	1	506	574	614	2919	1	1492	1839	1441
5	5	511	565	684	2919	5	3146	3146	3017
4	4	425	484	695	2916	4	2468	1902	2119
5	2	887	1019	580	2915	2	11617	9208	10940
4	3	448	493	541	2910	3	763	779	959
6	1	103	119	920	2908	1	6276	5713	7242
6	3	757	804	628	2902	3	4768	3696	4283

6	1	541	624	743	2898	4	2642	2496	2303
5	3	275	308	605	2897	3	22419	16786	19613
4	2	122	123	888	2897	2	1493	1850	1395
5	1	296	332	916	2896	1	3720	3616	2872
4	1	219	246	554	2894	1	425	325	374
4	3	328	363	530	2892	3			
5	1	143	165	633	2887	2			
5	3	3711	4157	569	2886	3	4012	3099	3706
5	3	139	160	979	2882	3	2291	2472	2021
4	4	615	653	881	2878	4	15668	14177	14224
5	2	807	887	525	2878	2	2572	2666	2292
5	2	437	473	944	2877	2	3739	2809	2572
5	2	149	174	582	2868	2	1703	1518	1718
4	2	219	238	892	2867	2	4377	3081	3695
6	3	379	434	848	2859	3	7719	7288	10544
4	3	393	445	600	2853	3			
4	1	605	688	572	2852	1	3712	3753	4071
6	3	4866	5332	803	2848	3	9864	7774	8709
5	3	671	756	711	2844	3	9179	7820	9816
6	1	402	438	894	2843	1	8423	6636	7904
4	2	779	903	800	2840	2	702	968	820
6	3	468	518	634	2832	3	6426	4788	4838
5	3	1325	1482	695	2828	3	732	569	703
5	4	414	477	510	2828	4	7597	6703	6982
4	1	271	292	837	2827	1	6484	5500	5375
7	1	243	276	821	2826	2			
4	3	1136	1246	988	2820	3			
6	4	263	303	1078	2820	4	5856	6067	8447
4	3	530	561	699	2817	3	2693	2404	2233
4	3	1464	1599	473	2810	3			
6	4	381	436	756	2805	4	2480	1944	2653
5	4	288	324	625	2799	4	6718	5027	6132
6	4	634	720	595	2797	4	7269	6024	7072
7	3	518	593	806	2790	3	15093	11370	12184
5	2	153	173	1122	2788	2	20252	13298	19475
6	6	2224	2466	668	2787	6	11481	9903	12751
4	4	1308	1491	681	2783	4	5994	6440	6311
6	1	118	135	492	2778	1			
5	4	148	173	932	2774	4	1892	1825	2129
6	2	150	169	859	2774	2	5984	5465	6176
4	1	168	190	491	2772	1	2555	2251	2518
4	3	282	328	487	2772	3			
5	3	127	151	916	2767	3	20502	18882	14003
4	1	255	294	878	2767	1	5976	5786	5477
5	1	681	794	673	2766	2			
5	2	342	377	696	2761	2	3093	3161	3718
7	3	219	249	654	2757	4	8669	8285	7618
6	3	228	260	545	2756	3	3242	2797	2728
4	2	1793	2059	723	2752	2	2435	2231	2264
5	3	1747	1948	723	2750	3	4941	4195	5054

4	3	995	1080	989	2741	3	2064	2389	1974
4	2	961	1091	797	2740	2	2653	2423	2566
5	4	509	570	671	2735	4	2872	2737	2404
4	3	1184	1285	580	2719	3	8819	8582	7803
5	3	508	574	775	2716	3	2029	1614	2061
5	1	497	566	870	2715	3	4200	2971	3401
4	2	566	637	634	2715	2	3211	2064	2514
4	3	804	904	610	2713	3			
5	4	718	768	746	2711	4			
4	2	522	583	545	2708	2	6265	5814	6617
4	3	1175	1302	777	2704	3	7471	7611	6927
7	2	87	103	872	2704	2	9970	8242	9724
5	4	128	145	506	2702	4	11271	7166	9105
3	2	930	1015	629	2699	2	2617	2910	2374
4	4	472	542	870	2698	4	3037	2774	3207
5	2	2091	2293	727	2696	2			
5	3	129	139	906	2695	3	19297	16011	16964
5	4	840	961	547	2695	4	6178	5221	4977
3	1	161	176	494	2694	1	2812	2792	2397
5	2	140	153	667	2689	2	3904	2859	4134
4	3	572	629	853	2675	3	6463	5317	6610
5	5	2193	2428	470	2674	5	7538	6839	6085
4	2	1426	1603	559	2674	2	11558	10516	9906
5	2	282	312	623	2671	2	8446	6371	5964
4	4	1524	1708	739	2669	4	4503	4776	4934
6	2	691	758	530	2668	2	6546	9596	9951
5	3	659	730	901	2665	3	6932	2024	6449
4	2	191	214	714	2649	2	5111	3866	4374
3	1	127	127	939	2635	1	130	126	137
4	2	2032	2314	701	2633	2	4336	3224	4474
5	4	486	539	835	2629	4	5070	5355	5242
4	4	97	108	655	2628	4	9410	6697	7667
4	2	1289	1385	731	2628	2	5147	4655	4929
5	3	382	428	914	2620	3	3270	2311	2949
4	1	420	469	687	2611	1	2491	2241	2332
5	2	1504	1636	549	2611	2			
5	4	403	455	810	2610	4			
3	1	174	188	931	2609	1	6437	7096	5996
4	1	124	141	815	2604	1	6866	6572	5341
5	3	1036	1220	563	2599	3	7544	6099	6830
4	3	623	728	562	2599	3			
5	3	377	436	637	2596	3	6645	5270	5982
5	4	705	799	753	2594	4	1039	1374	1857
7	2	1441	1620	909	2590	2	7332	7095	7461
3	1	355	383	969	2588	1	773	755	660
5	4	443	490	777	2588	4	7587	6502	5743
5	1	478	530	529	2586	1	1135	1044	1031
6	4	116	128	969	2586	4	4347	3292	3611
6	3	531	605	648	2585	3	10541	9329	10510
4	2	167	180	652	2585	2			

5	3	378	414	442	2581	3	13537	10663	11716
6	1	184	209	909	2581	1	4699	4812	5759
5	4	382	434	753	2581	4	13441	8793	8645
6	1	583	641	825	2579	1	2425	3375	2724
5	2	537	607	549	2579	2	9732	8759	8740
4	3	1393	1534	552	2576	3	1600	1272	1247
4	2	623	697	799	2573	2	122	76	95
6	3	207	232	657	2563	3	5374	5334	4378
4	2	452	493	750	2559	2	10277	9664	8270
5	1	296	325	519	2557	1	1470	1116	1118
5	2	269	300	903	2551	2	16014	11522	13544
5	2	395	456	469	2540	2	4116	2518	3143
5	3	355	382	544	2537	3	6503	4519	5192
4	2	1061	1184	475	2534	2	3745	3956	3333
5	2	1085	1217	533	2525	2	3713	3376	3347
5	2	682	779	644	2520	2	12723	11065	12026
4	2	219	240	680	2519	2	2040	2425	2651
6	3	186	215	1029	2517	3	5864	4511	13818
4	4	1983	2259	535	2511	4	10825	9743	10531
5	3	356	400	614	2511	3	11545	12539	10572
5	1	174	190	963	2508	1	7572	7065	7446
4	3	983	1135	615	2508	3	3106	2628	2466
4	2	170	178	840	2507	2	7601	6758	8151
5	3	623	696	607	2507	3	2876	2506	2380
3	2	678	763	571	2506	2	8097	7922	7493
4	2	497	560	529	2506	2	2996	2319	2689
4	2	1096	1245	651	2504	2	8670	8806	8297
6	3	1518	1702	901	2504	3			
5	3	586	647	506	2500	3	1295	1616	1171
4	2	378	437	872	2497	2	9182	9609	7730
3	1	759	882	799	2494	1	2039	2321	2805
5	2	594	649	960	2493	2	653	566	810
4	1	383	430	640	2491	1	2916	3225	2487
3	1	317	355	628	2489	1	2056	2152	2026
5	1	95	107	652	2488	1	9926	6324	7977
5	2	173	199	554	2485	2	13182	10858	11150
5	2	336	378	548	2480	2	4661	3296	4332
5	3	441	487	684	2474	3	4294	3828	3977
4	3	636	718	684	2470	3			
5	2	89	100	514	2469	2	3225	2478	3028
4	3	1032	1090	964	2469	3	10311	9714	10336
6	3	151	173	907	2468	3	10510	8504	6124
5	5	951	1052	674	2465	5	10133	8498	8932
6	3	433	488	642	2461	3	6524	2920	4221
4	2	526	605	878	2460	2			
3	1	548	617	785	2456	1	1131	1170	1155
5	2	116	136	952	2455	2	5348	4194	5397
5	2	149	163	911	2452	2	6622	5429	7676
4	2	300	333	630	2451	2	3486	3041	3486
5	3	639	686	929	2446	3	6726	4731	6796

4	2	311	324	464	2438	2	1473	1577	1440
4	3	143	168	613	2432	3	1092	808	1075
4	4	3675	4199	571	2422	4	1360	1364	1152
4	2	949	1038	655	2421	2	2270	1565	1862
6	2	130	141	582	2420	2	8002	4796	5661
5	2	592	655	564	2419	2	4652	3815	3850
3	1	240	273	774	2414	1	6595	7961	9106
4	3	809	903	725	2413	3	4405	3616	3723
3	3	314	338	605	2413	3	6963	8044	5941
5	1	157	180	461	2409	1	2340	1584	2025
4	2	335	365	498	2396	2	2605	1304	1960
5	2	199	225	613	2395	2	1972	2556	2485
5	1	233	240	863	2394	3	2799	2052	1996
5	3	903	994	673	2385	3	4028	3481	3213
6	1	245	267	761	2384	2			
3	1	351	376	489	2382	1			
5	1	506	580	540	2378	1	3616	2804	3963
3	1	632	695	654	2374	2	1414	1520	1391
5	2	307	349	607	2373	2	2523	2234	2603
4	2	289	319	464	2367	2	515	347	569
3	2	130	151	456	2364	2	2179	2439	2725
3	2	562	632	662	2364	2			
4	2	765	852	822	2361	2	5165	4660	5199
3	1	160	185	705	2358	1	4243	3314	4562
5	2	551	616	809	2356	2	7981	7052	7327
5	3	182	208	957	2355	3	14524	11662	13163
4	2	618	687	477	2355	2	3188	2815	2926
4	2	524	577	581	2351	2	10437	8885	8613
4	3	417	479	854	2345	3	2648	2188	2240
4	4	1008	1155	534	2337	4	2817	2155	1746
4	2	310	335	667	2335	2	24088	18990	21651
3	1	207	221	543	2327	1	359	342	409
4	2	450	509	527	2327	2			
3	1	78	88	742	2322	1	10390	7923	8496
4	3	725	803	961	2321	3	3829	3504	3482
4	1	128	137	512	2314	1	2175	1926	2620
4	4	975	1057	952	2310	4	7237	6695	6081
4	2	422	475	683	2308	2	2859	2181	2183
4	2	538	597	841	2307	2			
3	2	133	153	620	2303	2	7274	5800	5157
4	2	272	305	632	2298	2			
4	1	241	272	450	2297	1	6008	5422	5848
3	2	279	316	955	2295	2	3742	2847	3296
5	3	1056	1204	647	2294	3	6316	5802	8453
3	2	385	441	844	2292	2			
4	3	441	493	649	2292	3	7289	5602	6188
4	3	463	528	632	2287	3			
4	2	481	525	938	2286	2	3793	2880	4210
3	3	1797	2066	945	2284	3	808	699	775
3	2	293	341	749	2284	2	6642	5531	5139

4	2	235	268	632	2278	2	3015	2846	2973
3	2	356	398	516	2277	2	1662	1758	1810
4	2	240	277	696	2276	2			
4	1	425	476	857	2274	1	7814	6101	7096
5	2	93	109	931	2273	2	8071	6386	7599
4	1	489	542	524	2272	1	4275	4234	3718
4	2	614	654	477	2272	2			
2	1	543	602	536	2267	1	2075	2075	1794
4	3	495	550	619	2266	3			
3	1	503	549	794	2265	1	614	705	673
5	3	173	199	785	2259	3	8105	4577	6122
3	2	154	171	630	2256	2	3165	3154	2662
5	2	314	340	923	2254	2	2044	1642	1701
3	2	1981	2137	557	2252	2	2119	2340	1908
4	4	752	859	555	2248	4	9328	7763	9253
4	4	511	574	629	2243	4	3835	3504	3899
4	4	610	669	926	2240	4			
4	2	1446	1574	404	2238	2	1385	1113	1136
4	2	664	716	628	2236	2	11387	9697	10795
4	3	262	294	662	2230	3	10995	7815	10079
4	1	851	947	869	2225	1	2177	1916	2138
4	2	537	611	759	2223	2	3016	1818	3211
4	2	702	790	794	2222	2	4833	4483	3781
4	3	445	511	557	2222	3	3208	3238	2935
5	3	2596	2953	621	2218	3	10163	9265	9331
5	1	51	58	847	2218	1	4875	2978	4661
3	2	840	926	793	2217	2	3010	2979	2888
3	2	1869	1919	947	2205	2	2441	2604	2160
3	2	683	769	643	2204	2			
4	2	266	292	881	2203	2	5082	4041	4084
5	4	437	523	964	2202	4	5822	4709	5617
4	3	1123	1217	938	2199	3	3814	3583	4222
5	4	2383	2784	882	2198	4	8445	7057	6551
4	2	305	343	698	2198	2	3361	2293	3320
3	2	798	907	660	2194	2			
4	2	386	400	1064	2190	2	17699	14671	18351
4	2	302	345	554	2182	2	7015	5391	6203
5	4	170	194	720	2180	4			
4	1	276	323	835	2180	1	1020	969	1074
4	2	655	735	592	2177	2	3150	2397	2657
4	3	1693	1925	568	2176	3	3688	2665	3033
3	1	656	730	863	2172	1			
3	1	457	512	749	2167	1	2449	2031	2822
4	2	431	491	667	2165	2	10937	6164	8598
4	2	3586	3920	516	2162	2	2087	1930	2135
4	1	149	171	1089	2160	1	2282	2455	2439
6	3	161	185	1052	2159	3	6089	5513	6812
3	1	85	95	635	2157	1	4012	3083	3612
4	2	413	467	465	2156	2	1707	1419	1548
4	1	168	197	885	2153	1	7139	6158	6704

3	1	847	933	923	2151	1			
4	3	1084	1201	807	2147	3	8872	8203	6754
3	2	785	876	609	2146	2	3180	2915	5362
5	1	287	316	521	2143	1	8837	9193	8143
4	1	1338	1516	847	2133	1	4968	5811	4882
3	3	547	593	679	2121	3			
3	1	175	191	677	2119	1	2481	2588	2246
4	2	126	144	790	2117	2	5658	4075	5452
5	2	105	118	742	2113	2	7453	6333	6294
3	2	228	253	515	2109	2	2640	3261	2833
4	2	542	598	506	2107	2	2989	2664	2812
5	1	292	297	884	2106	1	7966	3936	5265
2	1	99	105	947	2096	1	2959	2955	2744
3	2	564	588	862	2091	2	1847	1847	1767
3	1	567	634	587	2091	1	3533	3253	4174
4	3	2015	2198	611	2088	3	2465	2186	1919
4	1	398	439	686	2088	1			
4	4	727	813	587	2086	4	5591	4869	5101
2	1	135	141	888	2084	1			
5	2	311	358	986	2081	2	3937	3286	3858
3	2	395	434	938	2080	2	478	397	393
5	2	90	98	954	2080	2	9097	8110	9480
4	2	1260	1441	545	2078	2	3041	3193	2965
4	3	567	620	824	2076	3	3421	2932	2916
3	1	231	264	544	2076	1	2196	2019	2457
5	1	157	176	810	2070	1	5033	4337	4542
3	2	235	265	870	2070	2			
3	2	707	794	606	2067	2	6369	5717	5721
3	2	263	292	569	2064	2			
4	1	2324	2579	639	2062	1			
3	1	154	172	972	2061	1	3907	3544	4291
3	3	133	154	639	2061	3			
3	1	171	196	674	2061	1			
4	3	367	410	881	2058	3			
3	1	265	294	989	2057	1	6287	5572	5701
4	3	1345	1455	884	2055	3			
3	1	1036	1163	910	2053	1	1308	1006	849
3	2	305	348	531	2053	2	516	484	453
4	1	255	286	671	2052	1	5014	4569	4895
4	2	406	452	693	2051	2	13015	9489	9871
4	3	259	277	534	2050	3	12575	12159	9056
5	1	386	431	613	2048	1	6564	5070	5478
3	2	271	307	870	2044	2	2928	2782	2825
5	3	2577	2746	711	2043	3	1354	871	1113
4	2	329	370	486	2042	2	2829	2305	2119
3	2	1357	1527	746	2031	2	6284	7958	7595
3	2	393	454	642	2028	2			
5	3	524	606	806	2023	3	8136	9305	8138
4	2	877	965	667	2022	2	5501	4894	4228
5	4	2357	2625	545	2017	4	14575	10381	11156

3	2	354	406	761	2015	2			
5	1	86	100	548	2014	1	11291	6953	9626
3	1	149	165	1018	2014	1	1750	1914	1824
3	3	552	624	794	2011	3			
4	2	398	465	995	2009	2	14592	11441	11455
4	3	336	387	508	2001	3			
3	2	1004	1122	638	2000	2			
4	2	182	204	1030	1996	2	5336	4522	4613
3	1	185	214	497	1995	1	3613	3253	3909
3	3	766	848	897	1995	3			
3	2	3218	3473	560	1995	2			
5	1	229	259	621	1994	1	7989	6292	6449
4	2	1164	1331	586	1993	2	9533	7586	10027
3	2	503	568	519	1992	2	3385	2704	2976
5	5	1937	2198	863	1991	5	8776	7878	7423
4	2	192	214	834	1988	2	2584	2632	2527
3	2	327	375	554	1986	2	895	868	942
5	3	972	1095	524	1985	3	1885	1507	1765
3	2	1037	1121	701	1983	2			
4	2	302	339	768	1983	2	2992	2382	2228
4	1	219	247	574	1980	2	4050	3073	2926
3	2	157	172	901	1980	2	3616	3366	3555
5	2	123	137	848	1976	2	8411	9628	9481
5	1	765	847	870	1973	1	9029	5394	6620
3	3	904	1001	486	1972	3	5216	5291	5249
3	3	336	388	454	1972	3			
4	1	107	120	904	1971	1	4380	5445	7572
4	2	499	570	591	1971	2	8690	8181	8105
3	3	257	289	758	1971	3	3537	3164	3076
4	3	303	345	715	1971	3	6322	5255	5965
4	2	640	703	772	1964	2	1496	1019	1344
3	2	378	428	625	1963	2	5540	4763	6569
4	1	460	512	695	1962	1	2284	1956	1715
3	1	612	673	638	1957	1	2361	1342	2444
3	2	548	616	649	1954	2	3071	3986	2935
4	3	222	257	684	1953	3	2007	1813	1942
4	2	2785	3136	620	1953	2	4662	3801	4586
5	2	327	370	689	1953	2	4594	3423	4279
4	3	324	345	614	1950	3			
3	2	753	843	892	1945	2			
3	1	335	384	615	1944	1			
3	1	236	270	639	1940	1	3400	3809	4197
4	2	252	280	850	1939	2	2837	2078	2535
3	2	491	567	568	1938	2			
4	1	298	334	526	1937	1	7252	5500	6307
3	2	266	302	752	1937	2	2487	2273	2493
2	1	306	340	677	1936	1	2550	2471	2387
3	1	333	383	616	1935	1	715	847	711
3	1	341	374	545	1933	1	11680	10499	8986
4	2	578	653	609	1931	2	5736	4478	5307

2	1	167	185	758	1922	1	2038	2124	1979
3	1	583	637	702	1922	1	1934	1711	1562
5	3	1296	1480	524	1922	3	1787	1843	1933
3	2	404	459	539	1920	2	2566	2006	2108
5	2	126	134	991	1917	2	6594	3562	4851
4	3	264	301	829	1914	3	4135	3627	3396
2	1	1180	1312	909	1911	1	5342	5279	4485
2	1	1373	1478	634	1910	1			
3	2	752	821	749	1907	2			
4	1	535	615	769	1904	2			
3	2	149	164	620	1903	2	5720	7438	5307
4	2	239	271	640	1903	3	1777	2246	2270
2	1	2426	2758	733	1902	1	829	1090	936
3	1	753	849	635	1899	1	1418	1213	1322
2	1	478	554	869	1897	1	3362	3152	3240
5	1	470	537	594	1892	1	6578	6578	5546
3	1	233	273	761	1892	1			
3	3	1717	1942	604	1890	3			
4	4	333	337	1020	1888	4	1230	1048	994
3	2	1886	1842	689	1887	2			
3	1	196	217	960	1886	1	5000	4062	4861
3	1	149	148	928	1878	1	4552	4468	4364
4	2	829	923	517	1877	2	8992	8549	7909
3	3	1188	1335	540	1871	3			
3	1	399	437	974	1869	1	619	560	511
2	1	181	204	646	1868	1			
3	3	450	497	515	1864	3	3555	2972	3964
3	1	177	187	1076	1861	1	7531	7965	6279
3	3	435	484	747	1855	3	2432	2668	2860
3	1	264	298	895	1855	1	4882	3815	4255
3	1	559	638	512	1852	1	4348	3806	3862
5	1	84	102	844	1851	1	3058	2608	2540
3	3	389	434	562	1851	3			
3	1	595	660	829	1851	2			
4	3	476	532	714	1849	3	4573	3376	3592
4	2	522	564	706	1849	2	11779	10021	9615
4	2	674	749	667	1848	2	1248	1072	879
3	1	366	405	772	1842	1			
4	2	542	620	654	1840	2	2802	2496	3193
3	3	1013	1151	531	1839	3	13361	13852	11153
3	1	965	1055	651	1839	1	7241	7908	6534
4	1	553	609	873	1838	1	2915	2313	2937
5	2	785	853	821	1837	2	5216	4987	5463
3	2	515	568	548	1837	2			
4	2	514	573	614	1834	2	6228	6132	6360
3	1	327	377	571	1825	1	4483	4243	3952
5	1	1019	1105	844	1818	1	7185	6618	7064
2	1	181	206	526	1817	1	7366	4778	5815
2	1	158	182	746	1817	1	1456	1887	1685
3	2	414	475	470	1816	2			

2	1	1113	1212	895	1816	1	712	690	692
3	3	441	516	633	1816	3	3300	3291	3205
3	3	1718	1931	604	1813	3	5742	4019	4627
2	2	418	463	994	1813	2			
4	1	225	249	654	1812	1	5173	3302	4749
4	2	865	968	609	1811	2	2450	2072	2416
4	2	345	390	489	1811	2	5047	4763	4660
4	3	1354	1545	536	1810	3	6495	6119	6232
5	1	159	168	543	1810	1	3837	2163	2314
3	2	331	367	661	1805	2	974	691	877
3	2	4651	5184	539	1805	2	2670	2211	2255
3	2	268	291	676	1799	2	9823	8162	9373
4	2	367	403	668	1799	2	3391	3327	3282
3	2	896	1002	629	1799	2			
3	3	987	1078	689	1797	3			
3	2	212	238	522	1796	2			
4	3	148	171	514	1794	3	7136	5713	7631
4	1	557	614	705	1794	1	4797	4528	4774
3	1	75	84	976	1793	1	8165	8024	7328
3	1	217	248	731	1790	1	1816	2032	2014
3	2	520	584	860	1789	2	3329	3294	2843
4	2	212	234	635	1785	2			
3	1	195	214	1015	1782	1	2651	2153	3214
3	2	226	252	844	1782	2	4899	4913	3894
4	3	1249	1416	897	1781	3	8109	7011	6681
3	2	1354	1523	633	1779	2			
4	2	200	218	483	1778	2	4377	3810	3840
2	1	417	456	846	1778	1			
3	1	916	1037	618	1777	1	960	812	873
3	2	386	418	850	1776	2			
3	2	695	775	873	1776	2	5655	5227	4704
3	1	165	172	885	1775	1	2916	3955	3681
3	2	789	884	614	1774	2			
3	1	680	678	914	1772	1	3607	2807	2739
4	2	715	776	463	1772	2	2656	2615	2705
2	1	1026	1117	644	1771	1	520	524	452
3	1	653	721	677	1768	1	1111	777	935
4	2	1640	1839	743	1768	2	7670	6672	7219
3	3	289	280	868	1768	3	3499	3345	6681
3	2	1103	1183	926	1752	2	362	387	356
3	1	92	102	454	1750	1	2747	2667	2373
3	1	125	149	815	1749	1	7568	4248	4905
3	2	898	1023	941	1749	2	5184	4602	4550
4	3	1160	1314	664	1749	3	5040	4409	4312
3	1	390	444	698	1746	1	1541	1245	1395
4	2	431	502	549	1741	2	3978	3523	4430
3	2	2078	2363	725	1739	2	999	892	753
5	2	251	282	752	1739	2	2581	1343	1752
3	2	394	437	632	1737	2			
3	2	263	292	848	1735	2			

3	1	254	295	938	1735	1	5927	7119	6393
4	3	2079	2357	693	1735	3	2028	1684	1791
3	2	288	332	982	1734	2	2951	2399	2196
3	1	518	594	728	1733	1	3971	4797	4064
3	2	570	606	582	1733	2			
3	3	967	1084	686	1733	3	4575	4208	4253
4	2	580	642	615	1732	2	13492	11109	9243
3	3	1187	1295	715	1730	3	5120	4410	4068
3	1	92	105	721	1729	1	4555	4565	4677
4	1	1888	2099	865	1727	2			
4	1	804	871	983	1725	1	1785	1961	1309
4	2	103	114	837	1721	2	6008	4754	5069
3	1	199	228	553	1718	1	1134	1042	1221
3	2	944	1064	884	1715	2	3565	2382	3648
3	2	1589	1714	533	1712	2	862	830	974
4	3	182	205	868	1708	3			
3	1	914	1033	667	1707	1	1561	1169	1318
4	1	1267	1400	657	1706	2	5819	4750	5754
3	2	325	363	681	1705	2	3568	2452	2706
2	2	318	369	916	1703	2			
3	2	578	644	783	1699	2	9230	7512	6947
4	4	635	737	677	1698	4	2928	2216	2477
4	2	128	147	948	1695	2	5734	3753	4561
2	1	435	481	604	1694	1	2418	2920	2231
3	3	875	994	683	1694	3	3557	3134	3500
4	2	615	690	785	1691	2			
4	3	188	210	947	1691	3	11570	11147	11127
4	2	331	355	708	1691	2	5168	5104	5479
3	2	4316	4936	599	1685	2	323	230	274
2	1	269	307	743	1685	1			
3	2	397	426	740	1682	2	257	386	424
2	1	659	692	970	1678	1	1360	1440	1159
5	2	822	912	533	1678	2	11435	6969	8906
3	3	740	806	668	1674	3	1131	1166	1193
3	2	486	536	467	1674	2	3928	3946	4732
3	2	368	413	752	1672	2	5049	4132	5182
3	1	241	271	521	1668	1	4981	2415	3845
3	2	531	585	477	1667	2	3937	2973	3032
2	2	983	1093	862	1667	2			
3	2	522	595	856	1665	2			
3	2	77	84	725	1662	2			
4	2	401	456	633	1661	2	3984	2771	3750
3	1	457	511	467	1659	1			
3	1	538	591	731	1659	1	332	421	383
3	1	161	183	969	1657	1	3816	4728	4678
3	1	490	541	679	1656	1			
2	1	1335	1378	1030	1655	1	1473	1672	1498
2	1	149	157	750	1651	1	1996	1728	1773
3	2	203	232	780	1651	2	2306	2234	2218
2	2	1226	1395	684	1650	2	1559	1186	1244

3	2	552	630	460	1650	2			
3	3	654	718	676	1650	3	13039	9531	10126
4	3	1184	1331	486	1641	3	9264	7217	8151
3	2	212	236	740	1639	2	4250	5239	4423
4	2	213	243	680	1633	2	3492	2023	2551
3	2	598	657	533	1630	2	3459	3113	3795
3	1	392	438	699	1630	1	7619	6625	6397
4	1	132	146	857	1630	1			
2	1	329	380	885	1629	1	1389	1484	1603
4	2	141	158	596	1622	2	3326	2829	2910
2	1	159	180	1030	1621	1			
4	2	1179	1264	945	1621	2	1907	1499	1619
2	1	188	212	536	1619	1	2366	2847	2632
2	2	993	1111	696	1618	2			
3	2	1665	1811	514	1612	2			
3	2	138	160	486	1605	2	2433	2344	2195
3	1	191	217	558	1604	1	2439	2097	2753
4	3	448	522	618	1604	3	16399	13248	14395
3	1	127	143	952	1603	1	276	306	261
3	2	3324	3678	560	1601	2	2545	2157	2217
3	3	534	585	702	1601	3	6892	5882	7020
3	2	331	384	468	1597	2	3913	3567	4111
3	2	369	418	515	1592	2	3319	4439	3286
3	1	250	280	796	1590	1	4760	7173	6928
3	3	1865	2079	686	1588	3	1736	1854	1653
2	1	426	488	800	1586	1	1987	2499	2162
2	1	492	557	531	1582	1			
2	2	159	173	848	1582	2	2130	2545	2315
3	2	947	1089	628	1581	2	6477	6854	6514
2	2	218	249	560	1580	2			
3	1	1814	2023	625	1579	1	2482	2203	1757
3	2	2836	3071	684	1578	2			
3	1	392	437	837	1573	1			
3	3	553	630	621	1572	3			
3	2	110	128	602	1572	2	1808	1535	1375
2	2	210	229	652	1569	2	2225	2211	2798
2	1	377	412	879	1569	1			
4	2	129	143	474	1569	2	6485	5780	6096
3	3	2698	3019	588	1563	3			
3	2	450	471	633	1559	2	9630	9808	8943
3	2	508	569	945	1558	2	612	490	596
4	2	361	397	543	1554	2			
3	1	322	370	468	1548	1	1077	892	1112
3	2	189	215	894	1546	2			
2	2	211	244	549	1540	2	16021	8645	11371
2	2	255	300	778	1536	2			
3	2	753	822	679	1533	2			
2	1	169	187	690	1532	1	1120	1415	1264
4	2	1005	1121	807	1532	2	9250	7942	9911
3	2	2980	3334	686	1531	2	575	399	483

2	1	337	375	511	1531	1			
3	2	825	932	670	1530	2	5221	5109	5141
3	1	609	689	787	1526	1	4167	2756	5474
3	1	236	271	933	1525	1	6834	6815	6314
3	1	160	183	815	1525	1	7983	7150	7244
3	2	405	447	474	1522	2	2065	2258	1923
2	1	339	396	926	1522	1	4437	3993	3436
3	2	190	216	783	1521	2			
2	1	695	805	492	1514	1			
3	1	328	366	926	1513	1	3358	2743	2582
3	2	358	395	533	1512	2	3077	2755	2281
2	1	1074	1200	721	1511	1			
2	2	319	346	477	1508	2	394	372	335
3	1	124	135	482	1503	1	1592	1272	1376
2	1	513	580	775	1503	1	1700	1860	1641
2	1	272	302	611	1503	1	95	86	47
2	2	347	359	859	1501	2	1713	1792	1638
2	2	228	248	569	1500	2			
4	2	219	254	1001	1498	2	5823	5215	5555
4	1	204	231	778	1497	2	1103	1104	1047
3	3	296	331	482	1497	3	2917	2425	2708
3	1	289	329	799	1494	1	2694	2677	2591
3	2	86	102	1024	1493	2	5663	6446	7259
3	2	286	326	907	1493	2			
2	1	506	559	692	1492	1	556	557	598
3	2	482	524	769	1490	2	1616	1405	1440
4	2	219	253	988	1487	2	2573	1748	2258
3	3	1215	1351	553	1486	3	5182	4277	4884
3	3	2004	2233	643	1484	3	2983	2490	2429
2	1	872	984	545	1483	1			
3	2	422	479	671	1483	2	4370	3152	4022
4	2	178	203	846	1482	2	7481	7608	9066
3	2	667	740	514	1480	2	3034	2647	2725
3	1	171	199	658	1480	1	2026	1653	1246
2	1	419	474	544	1478	1			
3	1	254	295	873	1478	1	2800	1887	2227
3	2	327	371	686	1478	2	5059	4100	4021
3	2	411	461	487	1476	2			
3	2	140	164	574	1470	2			
4	3	1346	1510	600	1470	3			
3	1	156	169	512	1462	1	4097	3084	3257
3	2	282	309	942	1458	2			
3	2	103	115	944	1455	2	6592	2802	7257
3	1	539	612	969	1455	1	3163	2326	3081
2	2	277	300	894	1454	2	15352	12321	12206
3	2	140	157	446	1454	2	2312	1920	2178
3	1	452	493	868	1451	2	7992	5761	6095
2	1	117	137	960	1450	1			
3	2	551	632	559	1450	2	4751	3690	3326
2	1	91	96	974	1449	1	5089	4215	5204

3	2	251	284	568	1448	2			
3	1	777	862	834	1446	1			
2	2	537	634	625	1445	2			
3	1	361	401	638	1444	1	5256	3812	4426
2	2	325	374	545	1443	2			
3	3	1245	1412	482	1443	3	3791	3906	3798
3	1	256	293	911	1439	1	7228	5556	5708
3	1	449	515	911	1439	1	1581	1291	1391
2	2	1466	1595	613	1435	2			
3	2	596	672	517	1434	2	6314	5042	5490
4	2	332	366	658	1429	2	1993	1427	1319
2	2	2444	2789	696	1429	2	5320	4628	4616
3	1	289	308	681	1428	2			
2	2	751	854	664	1426	2			
3	2	3199	3563	709	1423	2			
3	2	1917	2075	727	1422	2			
3	2	513	587	521	1422	2	2467	2035	2754
2	1	557	629	508	1420	1			
2	1	462	524	774	1419	1			
2	1	380	419	479	1417	1	3000	2982	2853
2	2	759	849	742	1417	2			
3	2	516	579	925	1417	2			
3	1	283	302	681	1414	2	3183	3135	3238
3	1	548	576	515	1411	1	2208	2256	2380
3	2	2342	2649	634	1411	2	10918	9542	10401
2	2	756	849	667	1405	2			
3	2	898	985	690	1402	2	3073	2689	3417
2	1	486	537	684	1401	1	6232	4469	5486
3	2	589	664	573	1400	2			
3	1	116	131	646	1399	2	313	326	336
2	1	140	148	805	1398	1			
3	2	633	735	903	1393	2	9840	7920	7555
2	1	304	335	717	1393	1	5497	5074	4445
2	1	510	586	863	1392	1	679	654	610
2	2	1686	1865	651	1391	2	3455	2558	2524
3	2	298	305	958	1390	2			
2	1	520	602	747	1388	1	2163	1796	1758
3	2	204	233	484	1387	2	8595	6730	7732
2	1	2049	2211	446	1385	1	4563	3408	3599
3	1	550	590	591	1385	2			
2	2	390	435	909	1379	2			
2	2	249	279	907	1378	2			
3	3	483	546	736	1375	3			
2	1	178	201	590	1375	1			
2	1	191	221	693	1375	1			
2	1	1120	1268	948	1373	1	5596	5113	5569
4	2	157	177	827	1370	2			
3	2	552	613	995	1364	2	5379	4592	4685
2	1	456	497	803	1362	1			
2	1	988	1110	595	1362	1			

2	2	337	392	555	1360	2			
2	2	279	307	544	1358	2	2245	1181	1771
2	1	283	324	911	1358	1			
2	2	219	250	621	1358	2	2326	2239	2280
2	1	360	400	664	1357	1			
2	2	512	564	566	1354	2			
2	1	128	137	625	1351	1			
3	2	656	747	702	1347	2	7172	5468	5854
3	1	416	461	677	1344	1	5849	5264	5594
2	2	843	940	664	1342	2	2110	1832	2024
3	3	231	263	832	1342	3			
3	1	239	271	894	1341	1	6362	5663	5138
3	2	521	580	892	1341	2	4820	4072	4969
2	2	548	651	559	1340	2			
2	2	120	135	534	1336	2	7291	5222	5478
3	3	709	798	555	1336	3			
3	2	426	485	746	1333	2	3345	2778	3017
3	2	290	325	585	1333	2	4129	3093	3055
2	1	475	529	731	1333	1			
3	1	248	298	920	1331	1	4419	3550	3898
2	1	1049	1167	473	1331	1	1938	1805	1988
2	2	486	538	945	1330	2			
2	1	323	364	895	1328	1	5552	5632	4227
3	1	600	692	712	1326	1	3301	3125	3074
2	1	1381	1507	816	1324	1	2786	2338	3210
2	2	282	318	644	1323	2	1369	770	1284
3	2	561	633	780	1322	2	6827	3873	5346
3	2	2050	2310	693	1322	2	5897	5615	5090
2	2	229	262	879	1320	2	3270	2512	2756
3	1	285	320	784	1318	1	5230	5389	4579
3	1	406	445	455	1318	1	2982	1602	2353
3	3	276	308	572	1314	3	5566	5817	4534
2	2	900	1005	951	1314	2	4513	3152	4710
2	1	273	306	549	1313	1			
2	2	1141	1287	865	1309	2			
2	1	179	200	822	1309	1	1374	1120	1151
3	1	147	153	1042	1308	1	4646	4284	3242
2	1	1300	1456	765	1308	1			
2	2	207	234	508	1308	2	4536	3799	3472
2	2	670	748	670	1307	2	2987	3212	3201
2	2	244	272	655	1306	2	7982	7218	8102
2	1	595	668	620	1301	1	2943	2962	3159
3	2	201	228	699	1299	2			
3	1	593	628	794	1297	1	1709	1479	1247
2	1	487	572	988	1296	1	2367	1486	2069
3	2	462	500	730	1292	2	3912	3626	3537
2	1	1359	1481	945	1290	1			
2	1	123	136	857	1289	1	2635	2407	2759
2	1	2232	2416	756	1288	1	2421	1825	1921
2	2	230	263	885	1287	2	2082	2357	2187

2	2	201	234	952	1287	2	2578	2433	2453
3	2	1725	1879	671	1285	2			
3	1	219	245	469	1281	1	6620	3985	5419
2	2	501	542	847	1281	2			
2	1	233	264	692	1277	1			
2	1	353	390	696	1276	1	1578	1509	1585
2	2	1464	1616	724	1273	2			
2	1	1321	1463	818	1272	1			
2	2	296	329	610	1271	2			
3	3	706	782	621	1268	3			
1	1	210	203	524	1266	1			
3	1	726	806	721	1266	1	3650	2810	2574
2	1	118	133	512	1262	1	4060	3818	3592
2	1	297	343	859	1259	1	518	410	476
3	1	386	446	548	1258	1	2901	2922	2618
2	2	444	483	568	1257	2			
2	1	341	387	898	1254	1	776	623	680
2	2	619	677	740	1254	2	4186	3641	4371
3	1	156	178	536	1254	1	488	365	685
2	2	689	749	764	1252	2			
2	1	263	291	996	1251	1	7283	8211	7685
3	1	594	647	412	1251	1			
2	1	386	424	703	1249	1	3919	3372	3625
3	1	230	256	583	1249	1	2416	1560	1607
3	1	1944	2023	885	1248	1	874	682	808
3	2	575	626	639	1246	2	4795	2648	4247
2	1	1268	1421	866	1244	1			
2	1	207	222	819	1244	1			
2	2	1415	1619	655	1243	2	3022	2532	2486
2	1	488	542	740	1241	1	1265	773	930
2	1	887	1000	734	1240	1	6195	5193	4979
2	1	268	302	923	1239	1			
2	2	227	265	904	1238	2			
2	2	887	996	777	1238	2	614	452	606
2	2	507	568	595	1235	2	3232	2545	2730
2	1	385	420	501	1235	1	234	202	199
3	3	362	424	525	1233	3	4409	2834	3365
3	1	109	123	1013	1231	1	3577	4818	4369
2	2	843	951	644	1230	2	4165	2793	3525
2	1	574	639	970	1230	1	1877	2196	2282
2	2	875	1005	629	1229	2	5237	7028	5560
2	1	515	593	671	1228	1	3392	3818	3709
3	1	4627	5185	527	1228	1	3201	2346	2698
2	1	148	163	843	1227	1	3626	3198	2846
2	2	363	408	812	1226	2	1698	1550	1518
2	1	779	866	630	1220	1	1845	1743	1615
2	1	694	772	711	1219	1			
2	1	1226	1418	812	1218	1	1159	856	985
3	2	1195	1335	517	1217	2	5633	3992	4100
2	2	484	573	550	1214	2	2142	1647	2019

2	1	344	398	545	1213	1			
2	1	612	659	947	1210	1			
2	1	455	522	670	1206	1	935	1468	1305
2	2	241	282	478	1205	2	1787	1536	1796
2	2	882	1013	784	1205	2	5835	5835	5391
3	2	329	373	991	1204	2	5746	3973	4305
2	2	680	746	938	1204	2			
2	1	1326	1483	681	1202	1	426	394	395
3	1	64	70	999	1200	1	2916	2314	3011
2	1	521	585	838	1198	1			
2	1	506	549	750	1198	1	5162	5100	3998
3	1	286	320	856	1196	1	5429	4260	4637
2	2	2186	2302	517	1194	2	1016	1107	1027
2	1	459	530	564	1194	1			
3	2	362	410	787	1193	2	2503	2089	2076
2	1	267	304	911	1193	1	4139	3126	4397
2	1	189	206	876	1190	1	1446	1165	1005
3	1	1706	1816	914	1190	1	2990	2786	2860
2	1	232	266	800	1188	1	2263	1628	1709
2	1	437	498	856	1188	1	1269	780	1214
3	2	709	801	803	1186	2	5232	4987	4475
2	2	1052	1196	733	1182	2			
3	1	2619	2938	564	1179	1	3519	2432	3209
2	1	775	855	511	1179	1			
2	2	599	671	652	1179	2	5244	6497	8598
2	1	260	286	595	1178	1	1315	1161	1021
2	1	926	1033	862	1176	1	1800	2012	1456
2	1	102	108	502	1173	1			
3	1	523	585	529	1172	1	4674	4391	3423
2	2	885	1001	824	1172	2	5753	4882	4575
2	2	1747	1936	765	1171	2	6327	5351	4564
3	1	286	319	486	1170	1	5959	4404	5263
2	2	154	166	994	1170	2	11440	10725	11260
3	1	569	638	510	1169	1	6685	3836	4474
2	2	639	732	1092	1168	2	3857	2713	2209
2	2	126	139	1162	1167	2			
2	1	957	1059	731	1165	1	2261	1909	2298
2	2	768	855	573	1165	2			
2	1	525	571	521	1165	1	4023	4074	4856
2	1	552	608	756	1164	1	3444	3921	4296
3	1	221	253	541	1164	1	3315	2685	4160
2	1	240	278	562	1163	1			
1	1	424	482	878	1157	1			
3	2	236	271	505	1155	2	3923	3460	3414
3	1	73	86	841	1154	1	2970	2109	2254
2	2	750	841	670	1152	2	2945	2300	2598
3	1	517	568	516	1151	1			
2	1	1505	1657	948	1151	1			
2	1	468	522	718	1151	1	1323	1168	941
3	1	108	122	926	1150	1	2928	3013	2485

2	1	461	502	680	1146	1			
3	1	566	632	916	1143	1	1410	1240	1052
2	1	138	155	488	1143	1	4875	3075	3245
2	1	985	1077	796	1142	1			
2	1	343	381	764	1140	1	1472	1295	1721
3	2	2031	2268	639	1139	2	6343	5522	5546
2	2	584	640	907	1139	2			
2	1	294	339	928	1138	1	7663	8163	6080
2	1	483	517	746	1138	1			
2	1	1599	1736	819	1137	1			
2	2	211	235	639	1136	2	2945	2733	2606
2	2	1102	1220	611	1135	2	2712	2799	2118
2	1	304	350	554	1135	1	2842	2153	2344
3	1	76	84	977	1134	1	1947	1968	2412
2	2	1436	1600	676	1133	2			
2	2	889	998	619	1132	2			
2	1	484	545	571	1131	1			
1	1	116	135	803	1130	1			
2	2	458	514	553	1130	2			
2	1	410	451	796	1129	1			
3	1	132	142	505	1129	1	5289	2353	3276
2	2	1131	1281	755	1128	2			
2	1	408	461	736	1124	1	5749	4519	4943
2	1	1535	1676	619	1123	1	3103	4228	3813
2	1	301	341	664	1123	1	3481	3010	3836
2	2	337	380	857	1123	2	601	550	693
2	1	193	216	514	1122	1	6249	4192	4799
2	1	1461	1592	619	1122	1			
2	2	422	484	721	1121	2	13064	11692	10373
2	1	152	170	724	1118	2			
2	2	823	931	668	1116	2			
2	1	275	300	512	1116	1			
3	1	187	220	512	1114	1	4789	3958	3705
3	1	415	476	922	1113	1	5797	3977	3337
2	2	955	1073	547	1109	2			
2	1	862	937	781	1107	1	4306	3717	4093
2	1	463	509	727	1107	1	602	463	517
2	2	598	657	766	1107	2			
2	1	1121	1252	711	1106	1	2476	2589	2507
2	2	222	252	952	1105	2			
2	1	282	312	922	1104	1			
2	1	163	186	898	1104	1	1257	1409	1485
2	2	227	249	901	1101	2	3250	2338	2164
2	2	602	686	799	1098	2			
2	2	517	527	942	1098	2			
2	1	183	209	957	1090	1			
2	1	938	1032	572	1090	1			
2	1	891	1022	549	1087	1			
2	2	762	825	642	1086	2	2868	2130	2654
2	1	607	668	497	1085	1			

2	2	1663	1864	643	1085	2	8170	6833	7208
2	2	1715	1900	458	1085	2	1875	1603	1878
2	1	768	820	699	1084	1	364	310	324
3	1	1809	1981	865	1083	1	2894	3295	3501
2	1	208	236	824	1083	1			
3	1	308	341	973	1082	1	3137	2472	3766
2	2	1639	1837	721	1082	2	2452	2123	2312
3	1	251	271	640	1080	1	4610	3774	4222
2	1	643	722	802	1079	1			
3	1	342	388	920	1077	1			
2	1	566	633	887	1077	1			
3	1	76	91	985	1077	1	2301	2426	1817
2	1	158	180	910	1075	1	3332	3103	3318
2	2	1064	1184	506	1074	2			
2	1	186	212	718	1074	1			
3	1	364	417	824	1073	1	7295	4955	6587
2	2	370	406	535	1073	2			
2	1	322	371	815	1072	1	3977	3040	3748
2	1	330	372	876	1070	1	1929	1437	1592
2	1	328	373	494	1070	1			
3	1	371	421	701	1070	1	2546	1797	2669
2	2	528	589	746	1070	2			
2	2	575	646	635	1069	2	5526	3928	5285
2	1	1519	1690	821	1067	1			
2	1	803	908	769	1066	1	5247	3719	3770
1	1	513	572	596	1065	1			
3	1	222	246	679	1064	2			
3	1	1031	1203	884	1064	1	4504	3304	3812
2	1	389	429	482	1064	1			
2	1	682	777	640	1062	1	2658	2366	2287
3	1	658	730	778	1060	1	2297	2153	2567
2	1	1694	1909	717	1060	2			
2	1	111	125	580	1060	1			
3	1	1058	1177	633	1057	1			
2	1	1109	1250	768	1056	1	2683	2322	2431
1	1	122	139	750	1054	1			
3	1	253	285	642	1054	1	1828	1609	1717
2	2	283	288	742	1053	2	947	788	940
2	1	339	371	633	1052	1	4762	3394	4308
2	1	131	141	604	1050	2	1753	1150	3301
3	2	148	167	1023	1050	2	6310	4246	5078
2	2	1000	1119	496	1048	2			
2	2	613	670	541	1047	2			
2	2	619	666	534	1047	2	2330	2608	2374
2	1	337	380	802	1046	1			
2	1	104	116	544	1045	1	1451	1332	1015
2	1	123	133	472	1045	1			
2	1	926	1034	698	1045	1	5941	4729	5480
2	2	1406	1559	660	1044	2	2867	2939	3170
2	1	115	121	587	1044	1	1952	2294	2293

2	2	377	424	511	1043	2	944	897	899
3	1	363	413	501	1042	1	3341	2167	2660
2	1	1627	1849	771	1042	1			
2	1	344	395	731	1041	1			
2	2	1292	1399	670	1041	2	6416	6577	6377
2	1	791	870	607	1041	1	9396	6544	6328
2	2	346	372	558	1040	2			
2	1	395	436	655	1040	1			
2	1	498	542	525	1040	1			
3	2	691	822	583	1040	2	3982	3898	3864
3	1	100	113	668	1037	1	3382	3478	4094
2	1	288	331	944	1036	1			
2	1	274	319	848	1036	1	884	878	933
2	1	717	786	548	1036	1			
2	1	672	766	512	1035	1	5277	5909	4225
2	1	291	320	963	1034	1			
3	1	219	230	796	1034	1	2957	2719	2385
2	1	399	457	727	1033	1			
1	1	313	359	644	1033	1			
2	1	745	824	516	1032	1			
3	1	252	286	844	1031	1	4290	3566	2956
2	2	322	378	477	1030	2			
2	2	1000	1158	955	1030	2	3457	2893	3363
3	1	236	248	746	1029	1	1227	1159	1130
2	1	424	476	540	1024	1	2829	2417	2287
2	1	386	434	723	1023	1			
2	1	265	311	536	1022	1	1904	1833	2860
3	1	161	184	941	1022	1	3132	2779	2631
2	1	397	443	906	1019	1	1728	1478	1597
3	1	59	60	1030	1017	1	2861	2203	3883
2	2	392	443	524	1017	2	3853	4428	3407
2	1	631	694	470	1015	1			
2	2	91	105	815	1015	2	5215	4446	4567
3	2	237	266	503	1014	2	3258	2962	2477
3	1	117	130	982	1014	1	2736	1633	2219
3	1	208	229	793	1010	1			
3	1	420	462	803	1009	1	1725	1545	1754
2	1	97	110	948	1008	1			
3	1	1163	1303	549	1006	1	4434	3974	5327
3	1	727	810	942	1002	1	993	1127	1079
2	2	790	894	897	1002	2			
2	2	608	683	576	996	2	6246	5713	4155
2	1	532	589	687	994	1	2224	1555	2259
2	1	88	99	481	994	1	2688	1660	2565
2	1	513	557	534	993	1	3422	3024	4143
2	2	504	563	515	990	2	2355	2528	1968
2	1	359	400	549	989	1	3618	2836	3749
2	2	522	574	925	989	2			
2	1	74	86	955	985	1	943	732	909
1	1	360	405	505	984	1	1442	1800	1290

2	2	699	770	660	983	2			
2	2	532	599	724	978	2			
2	1	1056	1176	445	978	1	3303	3097	3115
2	1	294	330	756	978	1	3167	2256	2660
2	1	326	362	708	975	1	3141	2440	2559
2	1	1007	1138	828	974	1			
1	1	885	972	692	973	1			
2	2	737	861	655	971	2	3122	2900	2464
2	2	3615	3954	587	970	2	2083	1772	1832
1	1	432	510	486	969	1			
2	2	407	454	873	968	2			
2	1	174	200	727	967	1			
2	1	722	832	602	965	1	4415	4915	4975
1	1	300	338	655	965	1	1629	1435	1470
2	1	284	318	599	965	1			
2	2	3028	3369	555	963	2	7235	6786	5449
2	1	176	192	507	962	1	3156	3941	3626
2	2	391	428	681	961	2	3444	3832	2902
2	1	372	411	548	957	1			
2	2	856	984	555	957	2			
2	1	122	136	497	954	1	5437	4669	4356
2	2	521	601	865	954	2			
2	1	679	761	777	951	1			
2	2	705	778	689	951	2			
2	2	158	189	536	944	2	2329	1741	2329
2	2	219	249	955	943	2			
2	2	1270	1412	600	941	2			
2	1	1013	1155	762	941	1	7130	7165	6567
1	1	208	238	652	938	1	1175	1300	1154
2	1	154	174	892	938	1	4639	3537	3092
2	1	1201	1369	1010	937	1	2607	2659	2223
2	1	498	559	841	936	1	4485	3782	5308
1	1	426	482	715	928	1	3062	2403	3117
2	1	326	368	668	928	1			
2	2	1179	1333	703	926	2			
2	2	392	449	665	925	2	2894	2853	3123
1	1	389	413	812	924	1			
1	1	239	272	535	924	1	3510	3209	3230
2	1	974	1102	506	923	2			
1	1	163	186	500	921	1	2050	1828	2062
2	2	288	296	872	919	2	1121	1093	1240
1	1	122	127	743	918	1			
1	1	710	803	755	913	1			
2	1	595	683	709	913	1	7130	4003	5404
2	2	548	629	489	912	2	4127	3138	3435
1	1	164	171	572	911	1			
2	1	168	194	879	909	1	5756	4455	3627
1	1	904	1026	555	908	1	2415	2718	2632
2	2	809	908	695	907	2			
2	2	1408	1584	843	906	2	5285	5186	5009

2	2	412	474	572	906	2	7271	5357	6096
2	1	524	588	906	905	1			
2	2	2204	2496	805	905	2			
2	2	626	690	635	902	2	4114	3991	4546
2	2	1761	2021	594	902	2			
2	1	183	205	739	901	2			
2	1	727	817	516	900	1	475	271	336
2	2	698	805	876	900	2			
2	2	578	642	813	898	2	2009	2249	2180
2	2	414	452	514	897	2	14191	10690	11706
1	1	1000	1059	544	895	1			
2	2	381	425	740	894	2			
1	1	115	132	846	894	1			
1	1	866	978	701	893	1			
2	1	3416	3774	639	889	1	1152	981	906
1	1	1094	1176	725	888	1	3964	3455	3688
2	1	1325	1489	681	885	2			
1	1	354	414	514	884	1			
1	1	408	464	655	879	1			
1	1	993	1074	892	879	1	620	696	496
2	2	190	218	525	878	2	1147	1185	1490
2	1	391	433	942	878	1	4348	3362	3777
2	2	160	186	744	878	2	1743	1624	1687
1	1	358	399	756	874	1			
2	2	108	122	654	870	2			
1	1	574	624	585	870	1	1778	2667	2523
2	1	120	134	790	870	1			
1	1	426	476	822	869	1			
2	1	254	287	911	865	1	520	507	504
1	1	428	484	945	865	1	675	684	576
2	2	187	213	857	864	2	5763	5338	5112
1	1	67	76	777	863	1	2856	2407	2377
1	1	522	595	857	863	1			
1	1	502	554	900	858	1			
1	1	381	426	816	857	1			
2	1	1018	1114	681	857	1			
2	1	879	984	806	852	1			
2	1	104	125	1080	849	1			
1	1	308	347	600	849	1			
1	1	1604	1766	625	847	1			
2	1	711	773	658	847	1			
2	2	308	318	777	845	2			
2	1	476	521	825	845	1	1811	1788	1838
2	2	4792	5507	585	844	2	3033	2638	2664
2	1	742	860	725	844	1			
2	1	397	432	620	844	1			
2	1	503	558	648	844	1			
2	1	520	590	802	842	2	5408	6190	7339
2	1	201	228	774	840	1	6138	7064	6207
2	1	490	567	756	839	1			

2	2	665	773	507	838	2	7083	8552	7728
1	1	174	202	505	837	1	1432	1521	1490
2	1	387	438	629	836	1	3959	2226	3715
1	1	812	873	725	835	1			
2	1	79	90	944	834	1	1997	1707	2035
1	1	228	258	470	834	1	745	647	568
1	1	547	622	705	834	1	3696	3430	3190
1	1	1620	1732	957	834	1			
1	1	596	684	856	833	1	1239	1426	1156
2	1	152	177	712	830	1			
2	2	521	584	680	823	2	5553	3728	3959
1	1	366	418	654	823	1	1078	1254	1055
2	1	130	151	1114	822	1	1504	1088	1608
2	2	387	446	882	822	2	3667	3897	3762
1	1	873	938	859	820	1			
2	1	421	475	616	817	1			
1	1	174	185	529	817	1			
1	1	700	762	916	817	1			
2	1	495	563	802	815	1			
2	2	330	369	652	814	2			
1	1	481	534	701	813	1			
1	1	491	572	508	813	1			
1	1	690	793	768	811	1			
2	1	689	776	810	809	1			
2	1	262	293	709	808	1	3896	3341	3431
1	1	320	345	639	807	1	1615	2362	1818
2	1	512	582	812	806	2			
1	1	79	90	725	805	1			
2	1	554	636	914	803	1	3889	3745	4103
2	1	171	188	724	802	1	3200	2923	3339
2	1	719	798	482	802	1			
1	1	471	535	898	802	1	3520	2705	2997
2	1	931	1048	783	802	1			
1	1	86	93	925	801	1			
2	1	182	210	772	800	1	6723	3637	5633
2	1	385	434	674	800	1			
2	1	829	923	679	798	1	6756	5240	4868
1	1	649	724	577	797	1	2416	3145	2404
1	1	699	769	520	797	1			
1	1	288	321	739	797	1			
2	1	1053	1212	583	797	1	2608	1935	2442
1	1	200	229	491	794	1	2635	2359	2646
1	1	713	779	753	794	1			
1	1	155	179	837	794	1			
1	1	913	1024	692	793	1			
1	1	136	149	723	792	1			
2	1	184	210	486	792	1	10455	9371	10258
1	1	208	237	723	791	1			
1	1	200	230	1056	790	1	2632	2436	2486
1	1	1064	1208	668	790	1			

1	1	3914	4447	780	790	1			
2	1	360	397	533	790	1			
1	1	512	567	651	789	1	2731	3283	2698
1	1	358	409	674	788	1	1461	1434	1429
2	1	243	266	597	785	1	838	692	694
2	1	626	708	586	784	1	214	225	142
1	1	112	129	752	783	1			
1	1	467	486	679	782	1			
1	1	178	201	914	782	1	2421	1618	2478
1	1	390	367	922	781	1	8424	2646	8821
2	1	225	248	590	781	1	694	681	2328
2	1	349	392	634	781	1	4095	3627	4390
1	1	817	916	660	780	1			
1	1	677	754	914	779	1			
1	1	151	173	507	778	1			
1	1	194	227	541	777	1			
2	2	661	761	872	777	2	5785	4884	4898
1	1	262	295	724	776	1			
2	2	1529	1612	680	775	2			
2	1	229	260	621	774	1			
1	1	351	396	674	773	1			
1	1	997	1128	705	773	1			
2	1	788	889	712	772	1	1456	850	1252
1	1	428	484	850	771	1			
1	1	831	963	844	771	1			
1	1	322	355	564	770	1			
1	1	293	315	496	770	1			
1	1	179	192	960	770	1			
2	1	167	181	796	770	1	4127	3461	3657
1	1	442	510	535	769	1	1381	1287	1405
2	1	103	118	1007	769	1			
1	1	345	371	788	768	1			
2	1	99	103	1165	768	1	5985	2218	2447
1	1	428	457	797	767	1			
2	1	739	806	563	767	1			
1	1	2181	2493	746	764	1			
1	1	718	818	733	762	1			
1	1	663	745	681	760	1	335	335	311
2	1	214	238	527	760	1	3758	2678	3121
1	1	773	864	846	759	1			
1	1	712	795	605	758	1	3720	3000	3443
1	1	753	855	586	758	1			
2	2	610	680	825	757	2	3732	2868	3715
1	1	528	606	588	756	1			
2	1	591	663	737	756	1	2951	2176	2444
2	1	327	376	525	755	1			
2	1	813	951	630	755	1	2553	2133	2552
2	1	76	88	1115	754	1	4576	4036	3779
1	1	878	1023	715	754	1			
1	1	359	404	875	753	1	1484	1178	1215

2	2	510	571	800	752	2	4312	3984	3422
1	1	849	939	601	752	1	1630	1445	1511
1	1	517	593	818	751	1	2302	2916	3010
1	1	341	388	630	751	1			
1	1	431	456	876	751	1			
1	1	286	307	496	749	1			
1	1	205	215	1125	749	1			
1	1	521	596	560	747	1			
1	1	779	823	863	745	1			
2	1	1403	1523	796	744	1			
2	1	689	745	983	741	1			
2	1	438	483	821	740	1	2312	2562	2040
1	1	64	68	450	740	1	1516	1526	1412
2	1	183	209	882	739	1			
1	1	65	78	1054	738	1			
2	1	594	681	875	737	1			
1	1	1412	1541	586	735	1			
1	1	1733	1963	706	735	1			
2	1	332	395	491	735	1	4508	3925	3964
1	1	385	429	897	732	1	1148	1141	1034
2	1	81	85	964	730	1	4091	3476	3918
2	1	248	272	859	730	1	4575	4689	6242
1	1	694	779	689	730	1	2748	2024	2154
1	1	1103	1248	596	729	1			
2	1	488	553	807	728	1			
1	1	461	506	581	728	1			
2	1	496	576	660	727	1	4904	3528	4856
1	1	931	1046	788	727	1			
2	2	2049	2256	723	727	2			
2	1	140	159	851	727	1	5937	4014	5602
1	1	701	804	673	727	1	1814	1604	1532
1	1	1373	1548	524	726	1			
2	1	698	803	559	726	1			
1	1	549	606	599	725	1	105	106	130
1	1	355	380	563	721	1			
1	1	264	299	662	721	1			
1	1	367	399	690	720	1	1092	872	940
1	1	254	274	482	718	1			
1	1	582	662	497	717	1			
2	1	360	415	664	717	1			
1	1	439	489	673	717	1			
1	1	811	915	819	716	1			
1	1	335	400	671	715	1			
1	1	714	825	563	715	1			
2	1	363	405	553	715	1	2461	1916	2257
2	1	704	792	818	714	1	2378	2348	2210
2	1	430	496	819	713	1	3831	3043	3697
1	1	103	118	797	713	1			
1	1	337	377	819	711	1	2214	2031	1867
2	1	265	306	687	711	1	2606	2336	3027

2	1	330	368	802	711	1			
1	1	550	610	578	710	1	2221	2082	2272
1	1	323	364	588	710	1			
1	1	415	473	683	710	1			
1	1	243	285	432	709	1			
2	1	365	421	550	709	1	8515	5412	7289
1	1	184	198	797	709	1	2120	2775	2558
2	2	249	280	588	708	2	5506	4060	4261
1	1	201	228	597	707	1			
2	2	894	998	640	706	2			
1	1	673	743	815	705	1	2184	2111	1772
1	1	160	181	544	705	1			
2	1	82	92	894	703	1	1778	1610	3237
1	1	706	762	527	703	1			
2	2	2379	2644	720	703	2			
2	1	640	702	796	702	1			
2	1	453	503	492	702	1			
2	1	600	684	482	702	1			
2	1	260	276	1067	700	1	4710	4551	3397
1	1	1556	1685	610	700	1			
2	1	515	578	456	700	1	3307	1735	2202
1	1	567	624	712	699	1			
1	1	1333	1411	813	698	1			
1	1	215	248	541	697	1			
1	1	385	409	762	697	1			
1	1	974	1081	676	697	1			
1	1	2119	2370	690	696	1			
1	1	257	296	982	696	1			
1	1	105	112	1026	696	1			
2	1	2051	2302	580	696	1			
1	1	805	938	771	695	1			
1	1	145	165	481	695	1	4619	4599	4529
1	1	512	583	935	694	1			
2	1	2102	2375	562	694	1			
1	1	255	289	888	693	1			
1	1	104	125	1010	693	1			
1	1	606	664	727	692	1			
1	1	355	379	951	692	1	1602	1418	1759
1	1	327	360	914	692	1			
1	1	1804	1955	723	691	1	937	896	884
1	1	666	756	605	691	1	446	452	508
2	1	194	218	724	691	1	8529	7590	6960
2	2	2177	2332	832	691	2	445	437	497
1	1	302	338	488	690	1			
1	1	747	824	482	689	1			
1	1	537	610	674	689	1			
1	1	312	326	802	689	1			
1	1	423	459	530	688	1			
1	1	151	155	784	687	1			
1	1	270	309	917	687	1			

2	2	238	273	932	687	2			
1	1	646	726	524	687	1			
2	1	1140	1247	851	686	1	2294	1727	1889
2	1	300	339	510	686	1	3862	4142	4205
2	1	1697	1915	620	686	1			
2	1	779	869	524	686	1			
1	1	434	500	853	685	1			
1	1	257	287	822	685	1			
2	1	414	465	787	684	1			
2	1	130	155	625	684	1			
2	1	103	112	926	682	1			
2	1	685	750	512	682	1			
1	1	2269	2524	567	682	1			
1	1	1638	1868	1096	682	1			
1	1	550	618	619	681	1			
1	1	292	326	708	681	1			
1	1	338	372	687	681	1	1484	2107	1895
1	1	423	472	736	681	1			
1	1	390	447	859	681	1			
1	1	179	199	922	680	1	1531	1381	1386
1	1	1777	1751	876	680	1			
2	1	179	206	680	679	1			
1	1	5680	6106	809	678	1			
2	1	339	376	736	678	1	2170	3842	3859
1	1	1504	1670	840	677	1			
1	1	2962	3211	566	677	1			
1	1	1343	1489	665	677	1	2316	2636	2256
1	1	631	696	938	675	1			
1	1	343	390	787	675	1			
1	1	400	470	847	675	1			
2	1	609	695	674	674	1	3911	2983	2901
1	1	332	370	935	674	1	2765	3791	2511
2	1	132	147	878	674	1	4454	3467	3727
1	1	522	599	1112	671	1			
1	1	178	203	860	670	1			
1	1	1536	1628	809	670	1			
2	1	257	291	531	670	1	3888	1838	1852
2	1	140	154	734	670	1			
2	2	1108	1259	947	668	2	3537	2671	3721
1	1	101	98	941	667	1			
1	1	547	606	660	667	1			
2	1	435	498	854	666	1	5488	4197	4413
2	1	510	580	635	666	1	4204	3794	4251
1	1	150	171	483	665	1	3211	2731	2783
2	1	346	397	536	664	1	2629	2789	2897
1	1	236	265	860	664	1			
1	1	527	609	897	664	1			
1	1	973	1053	692	661	1			
1	1	536	616	800	659	1	2585	3629	2103
2	1	2374	2682	863	658	1	5543	3712	5028

2	1	464	508	847	658	1	1580	1297	1546
1	1	925	1030	809	658	1	3803	3684	3759
1	1	745	799	764	657	1			
1	1	113	135	809	657	1	2929	2783	2867
2	1	1914	2187	661	656	1			
2	1	4201	4613	929	656	1	2465	1966	2357
2	1	232	258	835	655	1	3126	2641	3092
2	1	112	127	967	653	1			
1	1	587	648	897	653	1			
1	1	813	941	750	652	1	6568	6355	5511
2	2	3930	4420	543	652	2	10228	6982	7336
1	1	355	410	478	651	1			
1	1	186	218	969	650	1	3700	3339	3695
1	1	264	290	493	648	1			
1	1	621	683	815	648	1			
1	1	1152	1328	737	645	1			
1	1	1546	1766	529	644	1	3969	3443	4521
1	1	616	626	815	642	1			
1	1	1124	1223	759	642	1	4568	3488	4329
1	1	1140	1303	668	641	1			
1	1	2140	2382	891	641	1	3494	2849	3080
1	1	121	140	538	638	1			
1	1	95	103	475	635	1			
1	1	1206	1402	705	634	1			
1	1	164	174	481	633	1			
1	1	125	131	909	631	1			
1	1	303	350	824	630	1			
1	1	452	490	470	630	1			
1	1	628	694	706	629	1			
1	1	451	522	888	629	1			
1	1	566	648	487	626	1			
1	1	364	407	847	625	1			
1	1	308	345	1178	623	1	5675	5108	4931
1	1	617	661	712	623	1			
1	1	1514	1742	629	622	1			
1	1	331	387	587	621	1			
1	1	187	203	736	621	1			
1	1	270	304	903	620	1	3955	4552	4649
1	1	225	256	534	620	1			
1	1	274	291	895	620	1			
1	1	266	283	577	620	1			
1	1	1172	1299	661	619	1	1248	858	1344
1	1	659	745	929	618	1	2025	1735	1592
1	1	629	720	711	617	1			
1	1	1394	1546	624	614	1			
1	1	146	169	725	613	1	1182	925	1053
1	1	478	536	554	613	1			
1	1	318	361	592	612	1	2021	1874	1819
1	1	883	977	680	612	1			
1	1	1118	1126	596	611	1	509	493	600

1	1	2878	3173	497	608	1			
1	1	270	303	898	607	1			
1	1	1205	1348	661	607	1			
1	1	518	585	587	607	1			
1	1	412	460	727	606	1			
1	1	465	521	574	606	1			
1	1	576	649	568	606	1			
1	1	4868	5401	506	605	1			
1	1	699	775	980	603	1			
1	1	294	339	578	603	1			
1	1	442	503	536	602	1			
1	1	485	547	597	602	1			
1	1	484	555	879	602	1			
1	1	412	464	840	600	1	1808	1743	2352
1	1	86	100	399	599	1			
1	1	2991	3386	706	599	1			
1	1	1270	1421	606	598	1			
1	1	1056	1195	810	597	1			
1	1	171	197	922	597	1			
1	1	2885	3204	807	596	1	2391	2495	2207
1	1	139	152	787	595	1			
1	1	326	367	568	594	1			
1	1	556	628	794	594	1	6791	4589	3870
1	1	315	347	728	591	1	2191	1680	1801
1	1	321	360	640	591	1	4083	3832	3361
1	1	250	285	660	590	1			
1	1	505	582	718	590	1			
1	1	318	360	638	590	1			
1	1	237	262	560	590	1	2368	2001	2284
1	1	313	361	856	589	1			
1	1	300	338	966	589	1	3524	4366	4397
1	1	107	119	954	589	1	1536	1545	1205
1	1	1195	1316	473	588	1			
1	1	295	338	753	588	1	6185	4044	4746
1	1	180	186	818	587	1			
1	1	556	582	671	587	1	2320	1873	2141
1	1	140	149	941	587	1	342	323	362
1	1	701	781	582	587	1			
1	1	235	266	742	586	1	2702	2626	2688
1	1	206	241	714	586	1			
1	1	1029	1144	696	585	1	1600	1244	1113
1	1	182	196	592	584	1			
1	1	118	140	903	582	1			
1	1	1062	1197	609	580	1			
1	1	272	304	644	580	1			
1	1	236	266	676	579	1	775	717	870
1	1	792	885	638	579	1			
1	1	387	424	788	579	1	2109	1664	2016
1	1	225	261	484	579	1			
1	1	415	474	702	578	1			

1	1	501	560	496	578	1			
1	1	947	1044	736	577	1			
1	1	227	261	796	577	1	1757	2125	2363
1	1	338	390	686	577	1	935	1053	1119
1	1	831	956	549	576	1			
1	1	574	658	923	576	1			
1	1	1212	1364	674	575	1			
1	1	516	553	777	574	1			
1	1	541	621	744	574	1			
1	1	848	956	895	574	1	898	1136	1001
1	1	372	425	980	573	1			
1	1	717	815	590	572	1			
1	1	311	346	813	572	1			
1	1	491	539	541	571	1			
1	1	691	764	541	570	1	1366	1366	1366
1	1	990	1073	911	570	1	1426	1245	1557
1	1	416	469	714	569	1	5324	4676	4145
1	1	812	902	774	569	1	6045	3988	4803
1	1	800	922	709	568	1			
1	1	485	534	534	567	1			
1	1	1288	1410	437	567	1	621	688	651
1	1	364	393	695	566	1			
1	1	1877	2128	582	565	1			
1	1	509	592	787	564	1			
1	1	739	849	699	563	1			
1	1	319	361	725	563	1	778	735	711
1	1	1223	1368	680	563	1			
1	1	649	719	687	562	1			
1	1	351	405	818	561	1	1384	1671	1385
1	1	155	173	829	561	1			
1	1	1170	1235	706	560	1			
1	1	631	696	554	560	1			
1	1	343	381	803	560	1			
1	1	292	336	643	560	1	1557	895	1102
1	1	185	213	960	560	1			
1	1	638	709	802	560	1			
1	1	143	159	995	560	1	2117	1799	2215
1	1	328	363	797	559	1			
1	1	85	99	635	559	1			
1	1	219	250	772	559	1			
1	1	308	353	497	558	1			
1	1	1135	1229	668	557	1			
1	1	2875	3215	873	557	1			
1	1	341	382	501	557	1			
1	1	1395	1555	796	557	1			
1	1	1360	1516	569	556	1			
1	1	599	696	541	556	1			
1	1	602	685	683	556	1			
1	1	337	400	753	556	1			
1	1	432	476	799	556	1			

1	1	491	550	568	555	1			
1	1	155	176	727	554	1			
1	1	246	283	892	554	1			
1	1	993	1131	583	553	1			
1	1	85	98	920	553	1			
1	1	549	624	829	553	1	1991	1663	1945
1	1	363	414	587	552	1	3139	3533	2746
1	1	1442	1544	813	552	1	232	306	221
1	1	387	441	693	551	1			
1	1	592	647	865	551	1			
1	1	232	242	917	551	1			
1	1	892	1028	673	549	1	4244	3974	3320
1	1	644	737	604	549	1			
1	1	1565	1798	654	549	1			
1	1	1163	1290	683	549	1	5041	4498	4510
1	1	418	473	534	549	1			
1	1	1861	2022	625	549	1	2091	1607	1518
1	1	1919	2133	812	548	1			
1	1	149	172	543	548	1	988	762	1276
1	1	642	691	784	548	1			
1	1	385	420	646	547	1			
1	1	411	448	670	547	1			
1	1	955	1055	862	547	1			
1	1	321	362	505	547	1			
1	1	1126	1262	862	547	1			
1	1	289	338	824	546	1			
1	1	1055	1225	742	546	1			
1	1	422	474	818	546	1			
1	1	1103	1224	541	545	1			
1	1	271	312	743	545	1			
1	1	492	538	527	545	1			
1	1	542	598	689	545	1			
1	1	880	972	554	544	1			
1	1	612	687	885	544	1			
1	1	334	388	891	543	1			
1	1	321	361	1002	543	1	2895	2275	2026
1	1	601	668	580	542	1	2161	1506	1828
1	1	842	960	623	542	1			
1	1	592	655	917	542	1			
1	1	280	321	665	542	1			
1	1	662	756	961	541	1			
1	1	905	1032	824	541	1	820	731	842
1	1	290	337	507	541	1	4577	3445	3822
1	1	1029	1158	708	541	1			
1	1	502	553	715	541	1			
1	1	395	448	901	540	1	2294	1700	2048
1	1	2843	3255	580	540	1	3733	3781	4359
1	1	476	531	907	540	1			
1	1	1251	1401	594	540	1	2296	1702	1577
1	1	338	377	702	539	1			

1	1	344	396	607	539	1			
1	1	1222	1325	898	539	1			
1	1	370	438	961	538	1	2248	2728	2582
1	1	839	969	879	538	1			
1	1	285	326	928	537	1			
1	1	602	700	533	537	1			
1	1	1740	1948	572	537	1			
1	1	447	512	577	537	1			
1	1	1574	1667	742	537	1			
1	1	290	336	851	537	1			
1	1	436	490	720	537	1	1375	1584	1249
1	1	425	456	731	537	1			
1	1	181	187	778	536	1	689	899	778
1	1	736	834	544	536	1			
1	1	392	437	954	535	1	8069	6266	8156
1	1	257	284	552	535	1			
1	1	373	411	974	535	1			
1	1	1508	1722	771	535	1			
1	1	459	533	827	535	1	2719	2513	2131
1	1	798	911	709	535	1			
1	1	933	1055	580	534	1			
1	1	291	316	506	534	1			
1	1	476	532	900	534	1			
1	1	610	674	812	534	1			
1	1	288	317	519	534	1	5608	5643	5332
1	1	148	165	521	534	1			
1	1	1151	1283	569	534	1			
1	1	900	1009	635	533	1			
1	1	751	831	481	533	1			
1	1	336	349	982	533	1			
1	1	273	311	681	533	1			
1	1	773	850	781	533	1			
1	1	400	458	469	533	1			
1	1	173	196	986	532	1			
1	1	297	329	1007	532	1	1255	1008	1026
1	1	671	766	616	532	1			
1	1	564	637	712	532	1			
1	1	624	710	810	531	1			
1	1	487	552	576	531	1	1941	1869	1713
1	1	419	496	769	531	1			
1	1	1519	1616	728	531	1			
1	1	662	769	781	530	1	3612	4771	4139
1	1	1378	1532	851	530	1			
1	1	801	891	825	530	1	715	615	496
1	1	75	88	654	530	1	5060	7331	4836
1	1	856	963	824	529	1			
1	1	1574	1747	592	529	1			
1	1	355	410	553	529	1			
1	1	657	720	734	529	1	5894	5562	5982
1	1	199	235	740	529	1			

1	1	244	285	758	529	1			
1	1	763	855	627	528	1			
1	1	997	1098	928	528	1			
1	1	495	554	615	528	1	5263	4350	4769
1	1	266	304	837	528	1			
1	1	826	943	655	528	1			
1	1	474	523	573	527	1			
1	1	2012	2275	661	527	1			
1	1	485	537	829	526	1			
1	1	350	378	827	526	1			
1	1	400	443	619	526	1			
1	1	1000	1141	911	526	1			
1	1	381	410	884	526	1			
1	1	548	626	512	525	1			
1	1	288	339	621	525	1			
1	1	635	734	563	525	1			
1	1	290	310	724	525	1			
1	1	489	537	684	525	1			
1	1	901	1010	897	525	1	2837	4508	4007
1	1	170	193	853	525	1			
1	1	1249	1382	488	524	1			
1	1	444	495	797	524	1			
1	1	434	504	652	524	1			
1	1	600	688	644	524	1			
1	1	649	743	516	524	1			
1	1	276	333	948	524	1			
1	1	300	328	553	524	1	5555	4921	5692
1	1	141	160	582	524	1			
1	1	1382	1604	657	524	1			
1	1	469	525	879	524	1	3788	3717	3712
1	1	534	596	706	523	1	1471	1061	1357
1	1	184	207	835	523	1	3291	2490	2923
1	1	257	285	955	523	1			
1	1	740	819	574	523	1			
1	1	464	500	706	522	1			
1	1	1726	1943	607	522	1	3187	2988	3301
1	1	420	463	909	522	1			
1	1	1284	1434	674	522	1			
1	1	2037	2239	633	522	1			
1	1	907	1027	876	522	1			
1	1	619	718	655	522	1	3891	3490	3941
1	1	1196	1300	774	522	1	1390	1667	1532
1	1	273	311	936	521	1	4290	3001	2841
1	1	490	544	625	521	1	3570	3183	4047
1	1	592	677	1037	521	1	291	351	284
1	1	565	650	910	521	1			
1	1	1933	2152	859	521	1			
1	1	860	970	667	520	1	3487	5973	3476
1	1	354	406	737	520	1			
1	1	513	558	548	520	1			

1	1	221	258	614	520	1			
1	1	308	340	652	519	1			
1	1	1600	1738	702	519	1			
1	1	479	535	957	519	1			
1	1	416	457	942	519	1			
1	1	613	702	728	519	1			
1	1	591	662	576	518	1			
1	1	1780	2049	512	518	1	4193	4357	4071
1	1	1125	1248	775	518	1	4503	2969	3987
1	1	296	327	939	517	1			
1	1	825	894	787	517	1	1458	1477	1594
1	1	5019	5750	605	517	1			
1	1	797	854	720	517	1			
1	1	673	765	633	517	1	1528	1523	1585
1	1	1377	1548	784	517	1			
1	1	274	300	539	517	1			
1	1	327	377	788	516	1			
1	1	3788	4288	692	516	1			
1	1	413	476	821	516	1			
1	1	188	208	473	516	1			
1	1	360	414	655	516	1			
1	1	3096	3547	506	515	1	1799	2470	2290
1	1	230	257	744	515	1			
1	1	707	789	941	515	1	1811	1770	1449
1	1	3451	3767	502	515	1	1279	1540	1785
1	1	267	304	983	515	1	1431	1327	1284
1	1	4882	5346	667	515	1			
1	1	395	447	538	515	1			
1	1	594	679	720	515	1			
1	1	178	202	526	515	1	6634	5560	9151
1	1	202	230	670	514	1	1906	1597	1885
1	1	97	116	501	514	1			
1	1	391	428	630	514	1			
1	1	4887	5392	547	514	1			
1	1	470	505	835	514	1			
1	1	3299	3774	580	514	1	3403	3329	3113
1	1	291	336	642	513	1			
1	1	939	1046	844	513	1			
1	1	196	218	992	513	1			
1	1	442	486	947	513	1			
1	1	471	532	547	513	1			
1	1	905	1058	574	512	1	6951	6777	6670
1	1	931	1033	761	512	1			
1	1	263	302	597	512	1	1466	1205	939
1	1	809	889	866	512	1			
1	1	339	381	753	512	1	1101	815	809
1	1	710	782	736	512	1			
1	1	887	999	640	512	1			
1	1	437	496	569	512	1	395	274	382
1	1	188	215	868	511	1			

1	1	306	343	563	511	1			
1	1	123	141	463	511	1			
1	1	214	235	573	511	1			
1	1	240	276	522	511	1			
1	1	208	228	516	510	1			
1	1	148	164	1010	510	1			
1	1	268	299	522	510	1			
1	1	588	674	571	510	1			
1	1	1109	1275	522	510	1	2297	1777	1883
1	1	284	323	840	510	1	7129	7391	7017
1	1	1900	2139	690	510	1			
1	1	787	885	552	510	1			
1	1	102	115	437	510	1			
1	1	381	439	625	510	1	2264	1516	1979
1	1	683	781	901	509	1			
1	1	160	170	591	509	1			
1	1	591	657	606	509	1			
1	1	750	848	514	508	1			
1	1	399	451	947	508	1	1910	1322	1559
1	1	460	530	903	508	1	3098	2812	3420
1	1	488	529	635	508	1			
1	1	168	180	512	508	1			
1	1	779	868	515	508	1			
1	1	1452	1637	834	508	1			
1	1	378	423	865	508	1			
1	1	499	571	819	507	1			
1	1	306	348	609	507	1	1436	1284	1474
1	1	244	280	576	507	1			
1	1	297	326	793	507	1			
1	1	852	935	947	507	1			
1	1	1008	1132	634	507	1			
1	1	3663	4012	856	507	1			
1	1	558	601	764	506	1	688	462	631
1	1	1257	1418	658	506	1			
1	1	1166	1348	654	506	1			
1	1	946	1072	687	506	1			
1	1	367	419	933	505	1			
1	1	195	218	657	505	1			
1	1	349	382	835	505	1			
1	1	978	1096	736	505	1			
1	1	334	377	733	505	1			
1	1	986	1089	813	505	1			
1	1	620	706	639	505	1			
1	1	604	678	743	505	1			
1	1	924	974	891	505	1			
1	1	393	428	884	505	1	1914	1904	2017
1	1	498	549	505	504	1			
1	1	524	586	928	504	1			
1	1	145	156	995	504	1			
1	1	306	353	810	504	1	3551	2404	4757

1	1	168	191	559	504	1			
1	1	227	223	1095	503	1	854	455	666
1	1	648	720	547	503	1	1782	1541	1856
1	1	215	248	533	503	1	3641	2345	2321
1	1	385	440	740	503	1			
1	1	201	214	657	503	1			
1	1	500	578	806	503	1			
1	1	405	459	783	503	1	2820	2963	2569
1	1	552	608	816	503	1	2962	1966	2646
1	1	696	804	637	503	1			
1	1	569	642	711	502	1	9379	8218	7814
1	1	531	612	644	501	1	7171	4575	5673
1	1	1537	1715	762	496	1			
1	1	239	276	838	496	1			
1	1	307	340	979	493	1	3208	2793	2831
1	1	902	1015	635	492	1	2190	1734	1413
1	1	390	434	529	492	1			
1	1	674	728	677	491	1	4192	3915	2847
1	1	3729	4126	604	490	1			
1	1	474	549	869	486	1			
1	1	214	243	498	482	1	4678	3184	2944
1	1	275	301	550	481	1	5903	2647	2996
1	1	363	407	520	481	1	2333	1893	2090
1	1	413	469	585	478	1			
1	1	3010	3356	856	478	1	4592	3709	4162
1	1	703	786	569	476	1			
1	1	1210	1348	699	474	1	4877	4027	4883
1	1	140	153	725	474	1			
1	1	819	916	646	473	1			
1	1	123	137	680	470	1	2339	1932	2176
1	1	777	859	660	470	1			
1	1	539	605	564	464	1			
1	1	321	377	939	461	1			
1	1	182	208	972	460	1			
1	1	540	603	680	459	1	2719	2605	2420
1	1	484	545	717	457	1	1664	1344	1555
1	1	1590	1789	796	455	1	3763	3574	3500
1	1	1208	1377	580	455	1			
1	1	1097	1259	628	455	1			
1	1	293	331	705	450	1	2345	1785	1905
1	1	385	440	505	448	1	4085	3373	3924
1	1	646	724	683	448	1			
1	1	203	226	605	446	1			
1	1	740	840	621	444	1			
1	1	929	1058	907	443	1			
1	1	200	228	920	443	1			
1	1	307	352	512	441	1			
1	1	204	230	474	441	1			
1	1	170	192	936	439	1			
1	1	137	155	493	437	1			

1	1	388	453	644	436	1			
1	1	1388	1545	825	436	1	2663	2185	2913
1	1	677	745	857	433	1			
1	1	453	492	737	431	1			
1	1	852	956	835	430	1			
1	1	327	383	740	430	1			
1	1	265	280	667	429	1	1506	2003	2089
1	1	609	675	553	429	1			
1	1	573	660	851	429	1			
1	1	281	318	942	424	1	3500	2307	3341
1	1	1280	1335	989	424	1			
1	1	469	535	807	423	1	7032	5257	4888
1	1	673	757	655	422	1	2847	2378	2803
1	1	1012	1145	737	422	1			
1	1	543	625	812	420	1	2361	2255	2505
1	1	240	281	831	419	1			
1	1	251	292	958	419	1			
1	1	758	863	639	416	1			
1	1	481	532	928	416	1	3494	2747	3255
1	1	449	494	803	415	1			
1	1	993	1130	803	414	1	2498	2135	2340
1	1	788	883	635	411	1	2704	2871	2812
1	1	394	451	933	411	1			
1	1	488	533	932	410	1			
1	1	724	813	497	409	1	3823	3750	3530
1	1	834	934	736	408	1	3276	2496	2659
1	1	721	842	809	405	1			
1	1	600	685	526	404	1	2550	3692	2551
1	1	1108	1282	807	404	1			
1	1	331	364	876	404	1			
1	1	610	675	856	404	1	2065	1632	1968
1	1	560	639	838	403	1			
1	1	764	866	635	403	1			
1	1	285	319	642	402	1	190	150	141
1	1	314	349	703	402	1	3380	2384	2422
1	1	277	307	847	400	1			
1	1	1804	2050	667	400	1			
1	1	293	322	840	400	1	3564	2887	4240
1	1	907	1027	865	399	1			
1	1	159	184	859	399	1			
1	1	604	703	703	398	1			
1	1	618	696	637	397	1	1385	1124	1245
1	1	236	260	906	393	1	7640	8831	9505
1	1	735	838	807	393	1			
1	1	318	343	616	392	1	6660	5278	5386
1	1	563	639	605	391	1	3637	2938	3334
1	1	375	434	588	389	1			
1	1	2002	2258	613	386	1			
1	1	126	148	914	386	1	2435	1421	2084
1	1	333	379	977	385	1			

1	1	1973	2164	911	383	1			
1	1	173	202	948	382	1			
1	1	123	143	564	381	1	2560	2006	2556
1	1	757	861	938	381	1			
1	1	650	742	643	380	1			
1	1	68	77	1178	380	1	3122	3200	3476
1	1	247	279	913	379	1			
1	1	351	390	684	378	1	2519	2068	2287
1	1	231	271	775	378	1			
1	1	3575	3976	517	378	1			
1	1	158	185	560	378	1	2545	2680	2757
1	1	931	1071	690	377	1	4132	4550	3349
1	1	230	259	837	377	1			
1	1	528	592	614	376	1			
1	1	183	216	524	376	1			
1	1	252	273	544	376	1	3501	2662	3240
1	1	802	911	884	376	1			
1	1	545	601	712	374	1			
1	1	210	229	919	374	1			
1	1	776	842	922	374	1			
1	1	648	727	689	373	1			
1	1	199	214	541	372	1			
1	1	503	576	714	372	1			
1	1	626	709	524	371	1			
1	1	182	206	491	370	1			
1	1	92	104	866	369	1	3915	3998	3830
1	1	1267	1429	717	369	1			
1	1	318	352	895	369	1			
1	1	443	492	667	369	1			
1	1	1034	1198	901	368	1			
1	1	1134	1269	807	368	1	5119	3639	3583
1	1	64	76	976	368	1			
1	1	1519	1744	522	367	1	2704	2233	2567
1	1	1028	1148	655	366	1			
1	1	568	646	639	366	1			
1	1	1081	1175	557	365	1			
1	1	820	898	923	365	1			
1	1	1025	1167	807	364	1			
1	1	553	636	932	364	1			
1	1	580	634	544	364	1	2703	1858	2165
1	1	588	683	727	364	1			
1	1	1510	1712	555	364	1	3424	2584	2638
1	1	765	869	613	364	1			
1	1	142	156	753	364	1			
1	1	209	233	822	363	1			
1	1	1244	1417	566	363	1			
1	1	978	1132	619	363	1	498	439	397
1	1	762	864	508	363	1			
1	1	2115	2369	954	363	1			
1	1	240	273	564	362	1			

1	1	1406	1567	674	362	1	2009	1571	1624
1	1	327	351	780	362	1			
1	1	517	601	596	362	1	3997	2609	3507
1	1	488	557	505	362	1			
1	1	1029	1173	664	361	1			
1	1	3417	3782	851	360	1			
1	1	458	518	812	359	1			
1	1	1975	2287	661	359	1	2733	2632	2490
1	1	565	642	597	359	1			
1	1	159	173	479	358	1			
1	1	190	219	615	358	1			
1	1	776	879	616	358	1			
1	1	362	406	665	358	1			
1	1	326	351	887	357	1	2033	1963	1690
1	1	399	446	660	357	1	2912	2376	2961
1	1	294	313	914	357	1			
1	1	645	732	834	357	1			
1	1	408	456	931	357	1			
1	1	1461	1647	731	355	1	8476	6751	10149
1	1	578	651	841	355	1			
1	1	209	240	900	354	1			
1	1	644	718	703	354	1			
1	1	218	249	857	354	1			
1	1	478	536	958	353	1			
1	1	457	485	632	353	1			
1	1	381	424	502	353	1			
1	1	387	456	712	352	1			
1	1	664	746	1037	352	1			
1	1	112	123	634	352	1	9916	4285	6568
1	1	818	885	947	351	1			
1	1	229	268	799	351	1			
1	1	286	315	479	351	1			
1	1	1204	1370	582	351	1			
1	1	484	553	596	351	1			
1	1	399	450	974	350	1			
1	1	505	563	709	350	1			
1	1	705	804	890	350	1			
1	1	540	614	868	350	1			
1	1	887	1022	690	350	1	3507	2503	3616
1	1	881	991	651	349	1			
1	1	1091	1240	756	349	1	1466	1248	1265
1	1	698	806	534	349	1			
1	1	845	965	574	349	1			
1	1	633	722	651	349	1	7973	7821	7531
1	1	301	342	742	348	1			
1	1	629	706	585	348	1	1717	1569	1858
1	1	504	582	882	348	1			
1	1	462	526	778	348	1			
1	1	210	247	818	348	1			
1	1	461	507	925	348	1			

1	1	480	546	525	348	1			
1	1	751	870	686	348	1	954	1089	1093
1	1	466	530	680	347	1	2671	4951	5022
1	1	1987	2263	596	347	1			
1	1	2129	2395	667	347	1			
1	1	636	713	979	347	1			
1	1	490	560	564	346	1			
1	1	889	1011	635	346	1	2782	1912	2229
1	1	542	581	910	346	1			
1	1	1091	1247	560	346	1			
1	1	842	965	824	346	1	4885	4276	5527
1	1	358	396	897	346	1			
1	1	516	587	677	346	1			
1	1	189	203	916	346	1	3991	3060	3668
1	1	226	243	655	346	1			
1	1	133	146	1052	346	1	2945	3259	3418
1	1	1165	1268	512	345	1			
1	1	964	1050	596	345	1	3941	2946	3722
1	1	1604	1774	894	345	1			
1	1	175	171	493	345	1			
1	1	182	202	629	345	1			
1	1	599	665	929	345	1			
1	1	1623	1777	793	345	1			
1	1	323	379	512	345	1			
1	1	157	170	474	345	1	1651	1275	1609
1	1	310	358	963	345	1			
1	1	290	309	702	344	1	3179	2748	3179
1	1	1470	1658	677	344	1			
1	1	107	117	555	344	1			
1	1	447	487	810	344	1			
1	1	442	498	774	343	1			
1	1	341	395	854	343	1			
1	1	433	482	655	343	1			
1	1	1654	1851	916	343	1	3678	3835	3552
1	1	213	247	945	343	1			
1	1	306	347	878	343	1			
1	1	1386	1592	619	343	1			
1	1	1687	1920	655	343	1			
1	1	306	348	764	342	1			
1	1	1065	1216	660	342	1			
1	1	412	464	932	342	1			
1	1	444	483	566	342	1			
1	1	533	610	756	342	1			
1	1	302	343	794	342	1			
1	1	4512	5074	730	342	1			
1	1	1546	1668	440	342	1			
1	1	94	105	522	342	1			
1	1	996	1131	873	341	1			
1	1	282	314	692	341	1	2498	1968	1524
1	1	347	395	1005	341	1			

1	1	666	760	868	341	1			
1	1	202	229	702	341	1	4168	4180	4387
1	1	451	494	701	341	1			
1	1	357	384	739	340	1	3123	2509	2695
1	1	1794	2051	628	340	1			
1	1	1111	1266	746	340	1	2949	2453	2372
1	1	649	749	797	340	1			
1	1	1154	1324	743	340	1			
1	1	244	281	554	340	1			
1	1	602	686	702	339	1	6420	6220	7114
1	1	487	532	835	339	1	2867	2832	2574
1	1	707	767	649	339	1			
1	1	1249	1345	699	339	1	3903	2903	3649
1	1	153	173	916	338	1	2074	1997	4841
1	1	219	240	917	338	1			
1	1	915	1060	563	338	1			
1	1	729	814	508	338	1	6655	4599	6847
1	1	271	308	884	338	1			
1	1	249	274	506	338	1			
1	1	1992	2156	911	337	1	7136	5953	8359
1	1	319	371	554	337	1			
1	1	3096	3509	727	337	1			
1	1	357	413	853	337	1	3150	2145	2520
1	1	497	553	800	337	1			
1	1	77	87	972	337	1	6129	4697	5565
1	1	814	890	674	337	1			
1	1	347	393	944	337	1	7993	5626	6830
1	1	504	581	840	336	1			
1	1	439	476	618	336	1			
1	1	250	286	591	336	1	8054	6893	8064
1	1	181	215	955	336	1	880	646	690
1	1	264	306	624	336	1			
1	1	300	331	573	336	1			
1	1	1192	1352	812	336	1			
1	1	2249	2492	882	336	1			
1	1	1248	1365	524	336	1			
1	1	725	786	834	336	1			
1	1	910	1013	701	335	1			
1	1	529	584	784	335	1			
1	1	361	400	916	335	1			
1	1	1667	1889	502	335	1			
1	1	225	251	616	335	1			
1	1	340	389	926	335	1			
1	1	241	271	907	335	1			
1	1	722	811	662	335	1			
1	1	209	236	724	335	1			
1	1	496	552	827	334	1			
1	1	306	348	955	334	1			
1	1	606	680	602	334	1	6425	5505	4541
1	1	2244	2415	638	334	1	1512	1559	1583

1	1	416	469	552	334	1			
1	1	546	605	627	334	1	3706	4616	4003
1	1	485	556	526	334	1	2046	2010	2183
1	1	399	432	582	334	1	3902	3433	3140
1	1	260	289	477	334	1	1845	1655	1851
1	1	132	151	654	334	1			
1	1	322	356	701	334	1			
1	1	976	1135	686	334	1			
1	1	1221	1309	629	334	1			
1	1	404	469	519	333	1			
1	1	1072	1183	560	333	1			
1	1	1183	1335	552	333	1			
1	1	1479	1658	724	333	1			
1	1	3930	4519	613	333	1			
1	1	708	824	733	333	1			
1	1	153	173	520	333	1			
1	1	629	708	640	332	1			
1	1	122	128	747	332	1	5104	4316	4432
1	1	1217	1374	677	332	1			
1	1	1037	1206	768	332	1			
1	1	699	807	644	332	1			
1	1	172	194	954	332	1			
1	1	549	621	750	332	1			
1	1	312	353	863	332	1			
1	1	330	362	520	331	1			
1	1	236	275	936	331	1			
1	1	1587	1791	618	331	1			
1	1	372	403	775	331	1			
1	1	419	481	892	331	1			
1	1	585	669	696	331	1	6282	7032	8113
1	1	194	220	679	331	1	6124	4251	6477
1	1	411	472	530	331	1			
1	1	2718	2983	869	331	1			
1	1	3031	3334	885	331	1			
1	1	495	542	683	331	1			
1	1	357	401	835	331	1			
1	1	135	153	670	331	1	3638	2013	3151
1	1	761	857	812	331	1			
1	1	252	279	891	330	1			
1	1	3190	3531	841	330	1			
1	1	410	459	791	330	1	3870	3233	3727
1	1	159	186	451	330	1	2616	1843	2114
1	1	323	362	920	330	1			
1	1	660	752	503	330	1			
1	1	579	636	600	330	1			
1	1	1097	1245	873	330	1			
1	1	420	439	869	330	1			
1	1	194	215	613	330	1			
1	1	495	551	652	329	1			
1	1	1343	1528	693	329	1			

1	1	237	272	695	329	1			
1	1	683	765	743	329	1			
1	1	3707	4164	664	329	1			
1	1	177	202	922	329	1	5597	5029	6068
1	1	159	178	1007	329	1			
1	1	1197	1335	535	329	1	6527	4177	6926
1	1	119	135	844	329	1			
1	1	121	135	796	329	1			
1	1	332	382	876	329	1			
1	1	609	672	827	328	1			
1	1	1159	1302	774	328	1			
1	1	88	106	652	328	1			
1	1	630	719	752	328	1	3245	3653	3883
1	1	1295	1446	846	328	1			
1	1	430	485	516	328	1	3530	3066	2981
1	1	414	448	585	328	1	3522	2858	2697
1	1	420	461	914	328	1			
1	1	1056	1175	547	328	1			
1	1	342	369	655	328	1	1659	1223	1457
1	1	682	786	569	327	1	3066	2169	2574
1	1	1082	1236	815	327	1	2884	2884	3333
1	1	368	423	846	327	1			
1	1	1373	1543	615	327	1			
1	1	440	490	742	327	1			
1	1	280	332	624	327	1			
1	1	291	338	725	327	1	3114	2685	2427
1	1	946	1050	699	327	1			
1	1	821	941	568	327	1	817	716	713
1	1	1155	1309	604	327	1			
1	1	2013	2243	562	327	1			
1	1	538	624	827	327	1			
1	1	404	467	931	326	1			
1	1	548	598	472	326	1	3991	3579	4203
1	1	121	139	496	326	1			
1	1	270	302	805	326	1	4712	1967	4051
1	1	329	354	904	326	1			
1	1	330	366	891	326	1			
1	1	381	420	875	326	1			
1	1	1666	1904	705	326	1			
1	1	279	309	769	326	1			
1	1	626	682	649	326	1			
1	1	1188	1277	906	325	1			
1	1	495	561	994	325	1			
1	1	1646	1827	911	325	1			
1	1	978	1116	709	325	1			
1	1	265	305	901	325	1	3436	3440	3617
1	1	1296	1388	876	325	1	3105	2803	2954
1	1	850	985	601	325	1			
1	1	454	510	599	325	1			
1	1	245	264	569	325	1			

1	1	155	174	806	325	1			
1	1	315	370	464	324	1			
1	1	798	889	894	324	1			
1	1	810	918	515	324	1			
1	1	809	920	633	324	1	1851	1670	1824
1	1	623	707	668	324	1			
1	1	909	1020	628	324	1	4461	4019	4109
1	1	117	135	554	324	1			
1	1	1463	1622	848	324	1			
1	1	721	774	755	324	1	3969	3949	3431
1	1	79	88	775	324	1			
1	1	252	289	821	324	1	2864	2178	2441
1	1	740	854	860	324	1			
1	1	1344	1470	765	324	1			
1	1	509	587	647	324	1			
1	1	1046	1166	591	324	1			
1	1	88	103	1013	324	1			

Abundance	Abundance	Abundance	Abundance	Abundance	Abundance	Abundance	Abundance	Abundance	Found in Sa
545752	659313	728575	755343	551934	639012	726091	825728	547322	High
25889	21609	21603	45880	17625	20903	25572	40084	18303	High
499475	523991	502362	508967	514589	538567	517676	513207	527939	High
276901	348577	364026	370382	319902	355283	349075	378859	319264	High
120948	186869	156582	168523	141484	200388	155844	185117	140895	High
605380	684741	667740	748880	625119	594098	568251	659178	534396	High
542968	688609	625730	722097	588938	606185	540685	634193	509726	High
228559	242095	189785	220561	218252	269047	212991	251104	252293	High
18626	21006	17812	20204	18321	7509	7463	7960	7750	High
347699	403814	389316	487695	393343	327179	329469	396707	321510	High
426794	538392	507574	579708	466122	529253	483424	557486	454706	High
607619	583955	550643	671003	557730	558857	519591	651796	526286	High
53074	42774	66898	64001	47770	61651	93451	79068	65948	High
4065	5852	5715	3950	3934	5902	5677	4072	4108	High
					342	301	299	225	Not Found
404743	584314	546096	556415	428273	506150	478447	495811	373131	High
23426	28350	34123	32904	30392	23618	31175	27022	26106	High
311979	320675	306629	326552	304446	262687	243084	261417	248756	High
58157	46971	44998	48496	40195	34893	33206	38065	29650	High
312356	362023	341327	347452	338481	342590	327015	362675	325868	High
	3400	3869	3978	2934	8006	10132	10286	7177	Not Found
3212									High
361031	367814	355342	418593	331684	376568	369943	433804	343274	High
25738	12786	10458	18149	11044	10040	9189	11341	8737	High
283635	188735	187498	236055	196026	276142	267565	354811	285987	High
1264	1948	1735	1903	1601	3251	2909	2920	2714	High
371036	377140	316247	409147	336290	356357	302759	372359	314618	High
211927	243546	241518	276356	226428	221876	227435	253396	213102	High
	3864	5515	4289	4581					Not Found
354327	356148	341838	405690	335153	359642	325403	374011	321817	High
273290	204360	187706	308088	256733	180043	165361	267632	242426	High
97785	79503	88167	73823	90531	115873	129931	107722	136909	High
9730	3116	3340	3365	2730	12207	11957	11853	9504	High
200841	178784	176359	195118	163807	198647	199538	216024	184940	High
140896	181756	191770	205578	159367	202000	200685	217320	171716	High
1289	3365	3029	4318	2764	299	202	323	227	High
274490	232245	233360	313783	223201	252854	257385	331092	244176	High
112119	117141	161304	124718	134931	124387	147651	139654	138451	High
220926	258929	266072	291247	255913	250495	251473	274427	241476	High
49895	59764	57600	53323	54339	39804	44022	36231	36826	High
12162	6379	2503	5510	2912	2088	915	1939	1148	High
203420	243956	243917	278467	226302	260332	247668	276364	234441	High
200933	241676	216126	264499	204367	173724	154806	193577	148134	High
78377	51474	53609	67138	49794	59392	69254	62780	64135	High
9190	18317	16732	15374	17796	20623	18463	15315	20173	High
242256	212009	188861	213435	178264	211511	197734	209207	176045	High
36969	37361	12940	34966	16429	52354	40004	56373	45403	High
197130	179392	168803	191742	177278	172025	157577	182612	163536	High
143065	152537	133818	150817	151919	132945	118477	149784	132171	High

7591	4186	3882	3444	3425	15597	11972	11543	12802	High
217718	231460	203641	234694	201878	226960	205265	223936	196846	High
157346	166532	302423	208475	143205	194041	361865	235076	169673	High
21160	21474	21008	19859	20670	20440	20183	21998	19148	High
296371	201905	209654	279177	295119	218459	224867	315374	312448	High
218601	227785	197268	212594	201926	244976	205071	235836	215832	High
261880	239369	209467	312801	255742	303988	266862	377958	325556	High
12273	24587	20741	24263	20272	17583	15536	17172	15097	High
8467	4973	5480	5030	4112	3219	4006	3650	3037	High
13322	3121	2810	3053	2328	12366	9205	11377	8839	High
2803	5633	3083	4753	3430	5380	4334	5014	3673	High
142763	145057	127031	159196	130331	211899	188997	220602	191042	High
171033	259498	194734	228935	179801	247737	201470	227653	176271	High
97941	138492	128493	143227	123628	131338	107525	133261	110446	High
1547	1665	1675	1523	1494	2004	2082	1901	1776	High
3154	4708	4507	3656	3667					High
174605	208519	202332	212034	186932	186958	176544	187842	165470	High
99058	66656	52026	107635	59078	49225	39822	69941	45664	High
5588	10711	9392	10731	8826	7502	6084	7046	6210	High
104941	137556	125598	169903	142075	160024	141216	205097	157996	High
186411	135502	127215	159087	129464	223873	208936	244534	208710	High
115144	168612	157101	189216	149978	211170	194855	225454	187898	High
1455	8541	8409	9388	6866	13817	13338	14513	10717	High
137516	159769	148605	161891	132960	155638	149671	152166	126663	High
1973	1300	1422	1415	1337	1300	1324	1299	1157	High
5886	6551	5818	6425	5595	9507	8636	8621	7896	High
	1192	1192	1284	1031	1014	1097	1142	946	Not Found
157177	182831	180249	209316	155540	166528	156038	180347	138136	High
137470	130920	125109	143983	115214	124148	118260	143874	110222	High
153783	124379	108744	131153	105363	101100	98139	106605	93124	High
78390	76372	84370	79841	83049	54934	60663	60732	57413	High
26919	29132	35718	36767	33473	9711	12537	11345	11723	High
150752	178521	156553	165952	169068	174513	142043	167449	164279	High
165347	155266	136945	166809	151073	153864	144478	177015	156066	High
161158	179776	168924	189332	155517	166461	153084	170054	141198	High
29699	24597	24354	35989	23832	44562	32801	40626	34085	High
153935	161577	138648	182759	129277	178391	155426	184549	142882	High
137706	129727	145106	154676	141080	166955	172483	192473	170795	High
110905	160244	148557	188759	151577	145884	129444	170699	136688	High
198870	148923	158223	173736	140574	137911	132934	144500	120843	High
156812	147973	150866	146571	142168	156058	140466	152727	142059	High
134169	144194	154053	153620	130000	168866	168122	176777	143099	High
	1462	1818	1934	1321	6652	6528	8426	5078	Not Found
212841	165391	141300	188687	152178	144042	121458	165191	133049	High
115972	177798	144926	159531	153567	141669	128183	131721	127618	High
8037	15064	14142	12021	13572	8806	7163	8337	7400	High
					3201	3328	3205	2419	Not Found
6588	17609	20658	18220	16598	34019	38192	33370	30591	High
174654	137165	111528	166379	141333	124778	107351	136860	123335	High
59761	112564	93767	115117	103030	82881	71853	83289	77318	High

10857	21152	16864	17110	16253	12412	10388	10429	10545	High
18973	15025	13574	13416	13342	11891	10442	10615	10356	High
5386	3488	3490	3920	2988	8141	6608	9409	7317	High
159472	219693	201211	210874	183990	178337	164002	181524	153550	High
115024	116784	114518	117231	106248	117526	108959	114391	106568	High
	6360	6220	7575	5603	3912	3196	4298	3270	Not Found
101008	129698	112443	123536	104495	122062	101300	126757	94727	High
112566	116620	116213	140098	111197	148265	120787	160165	126879	High
164242	173093	147080	165218	158789	164278	148152	156400	154058	High
9504					5143	4070	5487	4446	High
128930	206990	187723	210633	172675	175016	156803	185928	149639	High
135410	181881	168812	168182	180657	148822	137118	139570	147676	High
124773	150960	149529	161967	122380	154781	160085	162444	126992	High
125668	160862	163828	160287	159651	146999	141758	142089	142430	High
16250	33144	24048	33983	22951	18303	17325	17003	14601	High
13079	3354	3426	3467	2936	9370	9722	11222	8192	High
64144	57007	55048	43954	53055	78099	81811	70606	75734	High
									Not Found
156831	150940	130235	141318	123532	193080	176299	198426	164925	High
123056	89762	83327	93782	93916	113798	95280	133837	112791	High
6720	4110	4702	4607	4955	545	490	1008	436	High
2008	3076	2602	3449	2827	2143	1911	2027	2053	High
78827	92148	75109	76571	84153	74893	58799	63188	67695	High
81935	116491	104709	132316	113633	98551	85459	101100	92825	High
12746	12445	11564	14107	11869	23175	18324	24697	21780	High
5566	1900	1344	1538	1520	2017	1429	1624	1535	High
13249	15041	13038	13625	12125	4078	3316	4704	3210	High
	8548	7294	8379	7318	10393	7954	10271	8050	Not Found
3580									High
82179	122501	112127	119085	117365	128974	108916	128988	119524	High
115524	151138	132088	146725	131579	130416	109210	134580	115248	High
176960	124763	101804	121687	107954	214741	176245	198905	183446	High
98871	86191	88531	90821	76417	90899	89036	90778	80987	High
90837	127361	105965	139916	112064	115294	99885	127757	104088	High
6261	4066	4244	4717	3985	9448	9287	9758	9221	High
138911	142470	116803	161496	132305	97233	73622	106652	83980	High
10461	22116	23214	20219	20560	14544	13207	11214	13321	High
105854	155201	154156	182162	146279	116129	114702	136502	110155	High
6819	5024	3821	3802	4356	6809	7611	6986	6527	High
110490	119988	125487	121008	94307	149218	157003	147860	114255	High
98490	146337	135665	192194	127543	92714	81106	124664	78088	High
	4830	3498	3381	3795					Not Found
105426	160565	147198	157196	142272	132226	125703	139965	121988	High
155653	154589	155233	150370	139253	142495	153539	135739	132440	High
11362	9234	10141	9889	9527	13585	13305	14220	12564	High
36771	26141	24667	28976	22665	32657	28836	34307	26469	High
1241	818	1190	929	991	1557	2376	1895	1827	High
3760	1613	2056	2204	2244	3876	4486	5191	5223	High
	2884	3374	2846	3625	1761	2238	1889	2649	Not Found
23347	22860	19958	21256	22087	28919	26359	25067	26433	High

71460	92477	140258	111913	79306	69925	100711	83148	57978	High
9466	806	776	940	780	1866	1566	1711	1473	High
51229	41187	36741	37878	37687	54480	49182	47948	50174	High
123302	84752	73440	82013	75893	78645	71085	76190	67023	High
132070	83487	72627	84176	70205	135200	114545	141623	111342	High
711	980	1138	887	896					High
60977	67290	69954	91414	62594	58210	55712	71800	52483	High
153638	108455	97935	111568	98379	113452	98058	123241	99626	High
69273	142291	133112	142345	116552	114168	108495	121618	97532	High
108636	131740	127881	120258	107056	88763	81879	77303	69438	High
64881	90566	85204	98058	85305	78977	74792	82480	72831	High
71914	74927	71595	70807	66178	93616	77345	77446	78960	High
99950	52791	55004	53165	46378	90531	85440	92810	78489	High
94534	148086	147051	162774	131128	123708	124696	130572	109760	High
	4068	3883	3304	3353					Not Found
77147	93657	77863	94530	80824	103619	80704	104340	85900	High
62812	46184	76635	86468	56702	43823	78716	81059	52780	High
81338	124640	105968	117433	107558	82611	67775	70944	65907	High
113782	102524	99247	139599	95219	129891	95245	162913	105939	High
1039	683	589	689	653	750	829	909	779	High
85632	122452	105956	120806	109117	95828	79142	91231	83536	High
92906	105087	95091	105774	88643	106223	91684	103653	88278	High
58961	51135	45877	71554	46023	44921	39579	66528	41591	High
	2625	2175	3387	2119	2998	2488	4209	2445	Not Found
65044	123227	122215	118818	108377	87772	88948	86540	77525	High
2853					3428	3061	3021	3045	High
69371	106185	77657	96096	94460	131133	93019	109132	113591	High
59944	48245	47495	48568	47990	35830	38812	37958	34676	High
69909	96479	86041	92530	78933	83676	74853	83606	67607	High
42199	34805	32362	50388	36844	27543	26614	40850	32624	High
113519	114293	96785	107310	104242	112490	96268	104118	102324	High
45124	40054	31513	34989	29092	61324	46833	53100	45758	High
86242	129026	112375	143023	113635	108446	94373	123726	92613	High
48594	44416	53597	49955	35292	46138	56548	46140	34643	High
75375	77663	62336	71916	66412	80467	62792	70460	64004	High
124458	73424	58764	76362	61319	121981	107750	133668	107583	High
70115	72778	60620	102796	75301	82219	64084	104355	77718	High
	8147	6608	9163	8464	3166	2550	4046	3246	Not Found
31471	28131	29143	35860	28120	41630	41437	46482	38768	High
78959	93695	95314	95249	75814	84500	89571	89873	70330	High
71045	126093	121494	131118	100577	67150	63967	69457	52175	High
86054	138706	115695	111132	101141	128795	101670	102445	90162	High
79516	77482	65939	71459	70309	87918	80020	80978	86142	High
4130									High
66673	46100	49413	56725	51606	44883	49709	60385	50294	High
117570	119891	100963	112495	118139	117087	98076	112807	112789	High
143178	105337	103501	121959	111327	102060	95327	101496	104530	High
64764	69242	63441	72400	62596	65219	60138	67266	56192	High
87690	94094	82461	98393	83517	87010	84103	93865	85592	High
84632	67738	65553	77590	62292	81437	75321	88370	74460	High

99578	86263	72830	99746	76872	113020	98762	117259	106665	High
66842	66212	63372	78345	68528	44986	48528	56012	46256	High
9086	1308	1332	1161	1212	14517	16058	13714	13521	High
92370	95556	85397	96567	82325	103708	87666	98155	88230	High
111408	124135	117310	135671	108737	79975	80174	91684	74677	High
93228	102613	94036	110124	88118	67431	64372	70426	59401	High
79090	165786	146146	166385	136095	104260	83017	98979	81059	High
88660	91140	82338	96373	85406	71306	61649	70904	62874	High
90508	116134	104511	129063	105648	122238	95241	122198	101900	High
61220	49402	55296	51566	42795	33239	33743	33534	27728	High
98499	103822	123388	127679	100493	90396	102141	96948	86401	High
4166	18607	16602	16441	17278	1878	1874	1802	1886	High
	2249	2902	2205	2539	2436	3048	2397	2738	Not Found
76634	86990	77714	84706	81349	86162	76741	82429	81525	High
69814	74195	68274	72366	61809	99259	87304	96162	78282	High
66039	70434	72069	85941	82258	68111	65942	77801	76424	High
1691	3226	2767	3259	2569					High
56309	49989	46262	54060	47308	81594	69236	82545	70139	High
48749	58302	63348	69274	57259	56960	57792	67808	52382	High
78171	88421	80957	100027	84794	77180	71244	87128	71341	High
68950	74724	76098	83666	77349	76832	77071	96798	79034	High
10496	18687	15208	15613	16978	12700	10376	10471	10842	High
96518	72612	83404	104410	77782	98257	107517	126781	101662	High
80097	91216	80903	84280	84848	108395	84640	100690	96643	High
108216	74332	71532	76055	71731	91855	86591	90370	85732	High
80305	69256	67829	73783	72297	81033	75213	84631	83396	High
80608	144374	101187	122892	125168	102034	75688	92687	91450	High
61950	82869	67656	99925	72424	69115	56963	85138	62841	High
74872	82740	90490	97156	82754	72503	72525	80843	75923	High
6363	8721	8183	8149	6963	593	620	509	561	High
41801	60517	40498	50063	45601	97277	62888	83566	72824	High
10431	17236	21656	18502	19763	11538	14002	13478	12490	High
71720	81463	80541	93230	91122	48909	45340	53610	50071	High
56126	73118	65612	69503	68120	64112	54531	58474	57742	High
41886	52487	51054	55367	51648	58857	52366	60286	54046	High
60442	87345	81710	90329	65707	67990	69232	76551	55742	High
121301	109979	102544	114168	98018	100419	90244	107083	87904	High
82105	91494	87090	92613	79799	71207	68975	80220	65948	High
2512	4582	3984	3722	4253					High
20967	24964	23007	21735	23891	15584	12987	15720	14882	High
	446	409	331	443					Not Found
65686	74684	74423	97772	68867	60496	65052	79898	58623	High
49890	39905	34330	36416	36669	40456	37553	37240	40093	High
8778	9846	7195	10875	8103	10836	8326	10941	8933	High
46135	64872	53720	62861	51327	65743	54869	64135	52233	High
26801	18925	16342	16910	18437	9595	9753	10625	10677	High
48182	96296	106439	101183	85227	106852	115161	109723	92432	High
46573	32773	33043	31379	29521	78967	76424	81941	71689	High
74133	94648	77139	96736	77410	71441	63993	65899	63679	High
76683	79817	71223	77462	66493	76393	69018	71363	64514	High

					5091	3996	4508	3904	Not Found
63724	63575	69196	72514	63030	63814	66131	68169	62839	High
16982	14782	13820	12727	14261	7529	8132	7337	7189	High
63693	64376	52842	61400	45422	119104	111057	107735	91685	High
74368	78180	70466	73354	73823	91974	81248	100887	83939	High
59579	76771	64656	76754	70556	74276	64011	72741	69736	High
24522	25991	22598	26262	25197	20058	16789	18842	19067	High
2393	8505	4562	5985	5986	5047	3111	3937	3911	High
60393	66189	69429	64754	65322	44632	44924	42894	42408	High
48513	46433	41118	43246	37768	42010	40698	40982	36592	High
46614	67494	47116	67874	45631	65375	46665	69302	46278	High
77868	65699	74090	75936	72787	105106	112842	119875	109655	High
53563	35541	42128	49414	46376	47071	54626	60326	56026	High
4773	14376	9998	12155	12057	13316	10206	13161	10538	High
8747					15347	11163	10438	9869	High
51282	39363	34892	47907	34607	51631	44998	53648	44061	High
47154	59450	49932	56936	48533	63308	57237	61135	52809	High
7302	8851	10620	12116	10160	10388	15002	14394	13478	High
45593	41819	36343	41621	36122	44856	40100	43380	41544	High
3527									High
60330	75980	74347	74130	71043	61930	57699	57016	55314	High
10331	4690	4397	7561	3226	8478	9720	11756	7354	High
3931	6505	4678	4780	4029					High
54683	58690	55968	61501	55582	52515	48715	55278	46474	High
47168	66181	60919	67233	61206	45625	39761	45044	40745	High
81316	115156	106470	103325	86682	141020	132776	134847	103769	High
48071	71484	58974	71895	60315	63628	52484	64401	54374	High
37945	33081	28627	26804	30359	62579	53143	52754	57494	High
55954	70144	58952	69228	58584	71830	64687	70863	61158	High
9281	3351	2947	3627	3278	10123	9555	10188	9597	High
53382	33796	32060	32637	28338	62828	55611	56895	51121	High
37105	53678	45677	51067	44539	60218	51756	57498	50310	High
60367	66177	56249	58761	62257	53208	41378	42910	44678	High
68578	73514	69791	67862	63946	61287	60669	59477	54969	High
88573	63770	57174	64300	58142	77621	72377	75589	69834	High
58317	68988	67623	71975	69975	58240	58251	61689	58407	High
43181	81229	79490	82250	59144	89279	82321	85459	63838	High
90870	73970	55889	76733	69318	112278	85288	101505	102808	High
53987	65639	52243	64136	55367	64888	54256	59100	55254	High
48274	59898	57615	59096	60021	61330	58955	65441	62901	High
78816	94176	87879	99468	86336	72733	64443	74260	63672	High
12569	16292	11037	12862	12773	18410	14859	16050	12976	High
76294	90038	70427	87443	71598	78957	64805	78944	65389	High
32599	37860	32526	32455	33461	63765	55245	57580	54172	High
44934	42489	41814	38681	43961	60282	58190	55389	61921	High
32870	33685	27805	35648	30615	53239	41655	47819	44985	High
388					5392	5038	5821	4659	High
21218	22075	25455	22926	24185	20758	21337	19788	22624	High
49286	41041	35185	41608	35510	68666	60727	63489	57034	High
80560	69459	67842	59266	73025	55201	54946	45645	54053	High

14730					8039	7257	6843	9292	High
50545	69680	58406	64094	55029	83068	76458	80920	70794	High
57853	77689	77411	85627	73230	52833	51703	53481	49197	High
60789	78425	58028	76673	66915	71698	49964	67531	58592	High
75551	80062	73371	82864	76312	88270	82400	89323	84005	High
72864	81582	68778	79312	76547	68749	61692	64575	63422	High
69986	59866	57011	59302	49575	89021	84972	87611	75886	High
54489	88337	62511	76464	76048	55686	38549	47848	47413	High
26778	61709	45327	62309	47485	31540	27731	32767	26301	High
85700	70970	65203	76408	68400	84515	78022	91132	81398	High
66303	48922	45652	54146	42813	49814	47823	55816	44850	High
98233	69699	51670	68036	64909	52123	41413	51549	47817	High
46351	66273	59347	60119	59908	46678	38652	38682	41064	High
	9967	8442	7892	8187	8901	5951	7612	6872	Not Found
55319	62560	61043	44853	38652	80328	73911	62405	54345	High
36198	81455	81230	77724	70113	93594	89578	85030	78163	High
19673	3445	3715	4130	3473	8757	12671	8843	8357	High
54655	87980	86229	85947	75394	99186	95395	91439	84767	High
64499	66825	60639	60510	58484	52897	49890	53185	45576	High
53532	62369	49512	65134	57024	39810	32711	38622	36810	High
9473	1614	1493	1865	1339	12478	14561	16627	12797	High
50022	31061	27843	34171	27035	42304	36247	47054	36754	High
35650	43458	31603	44945	33632	49729	36572	50973	35692	High
63959	66886	59576	67855	59410	89874	87141	95763	82940	High
49513	55713	53748	56513	51287	50897	48440	57842	49043	High
21873	38039	39104	49872	49611	25042	26866	33546	32213	High
58625	63923	52385	60760	50822	62442	51357	59690	52095	High
54107	61334	59299	60855	57835	46959	48971	48722	46149	High
36670	23684	28275	28537	24033	40668	39015	44912	35345	High
67671	51199	47253	53652	46364	51710	44471	56609	46473	High
62107	46352	39521	47091	39978	29143	27186	31950	28317	High
42282	34365	28268	32870	31369	21572	19903	21129	21566	High
3025	5313	4297	4097	4144	9329	7679	7620	7199	High
36643	16589	13206	16574	16095	60489	51294	60029	62186	High
63253	35790	36941	36197	37249	58644	58764	58814	57680	High
80054	76581	66032	72744	64932	59635	46635	54805	49454	High
12680	15482	14238	13766	13925	13349	11631	10481	12415	High
50076	65841	53439	61422	53755	53114	40435	49786	41101	High
66476	71049	60191	61953	61091	51343	45801	47795	45544	High
6249	14214	9167	9818	14799	1551	1175	1311	1778	High
67484	84151	75879	78685	78600	65151	59893	65253	60502	High
26617	42129	26005	39228	34230	27078	18634	27662	24006	High
64166	86434	81963	88104	81830	49658	43714	46446	42422	High
81860	84315	68191	75090	76695	72357	66349	65715	70578	High
45359	49202	47962	42927	45347	45329	36666	41604	38158	High
72379	78872	70376	93218	70386	98845	86910	111754	88681	High
46461	41476	45245	49116	46289	35884	37250	42020	40105	High
78383	79524	67342	86259	67362	57335	48672	61466	48530	High
70932	65608	65672	59640	53180	69832	74172	65348	55838	High
37274	40119	38958	48531	37779	39558	36895	47303	36017	High

30802	35678	32099	34358	32538	47077	42373	45353	42485	High
86834	73311	76907	89860	72851	68882	63379	78388	66884	High
98983	44529	78446	63350	95502	43414	72930	61657	84927	High
90794	109491	84878	110098	96882	68788	54833	68738	61729	High
62064	59769	56195	57374	59990	56148	47987	51037	51117	High
					1818	1988	2431	1668	Not Found
39057	45281	46657	42720	38744	44833	43532	44136	39244	High
54449	68973	62234	82933	65227	66679	63186	75030	63654	High
49534	54082	50791	64704	45952	75705	74373	73622	59859	High
61911	65053	60005	66973	57570	53167	42961	57860	47493	High
57667	35935	32940	39438	30792	66683	57163	65511	54585	High
6355	5841	4324	5526	5013	6712	5365	6587	6005	High
53520	73050	67794	72303	61092	67027	59226	60817	54678	High
51156	52814	45389	55834	44949	31508	26969	35079	26215	High
28688	51526	43546	51054	44183	54394	40382	50578	45256	High
72151	67724	64637	71256	72783	69296	58130	78113	73092	High
71247	100211	88099	109932	107358	58710	51714	63861	63596	High
56394	69668	58881	78672	68532	68674	58752	72441	68909	High
11776	13309	14556	10185	13879	8007	7976	5955	8454	High
14165	40774	34032	34451	33809	27443	24922	26003	22185	High
30400	56626	45441	54642	51484	36515	28319	38360	32239	High
75885	68373	63227	75416	61702	103352	89227	102144	87295	High
20863	27761	21791	25760	27757	33163	26361	31713	32909	High
36141	53703	47472	52972	45573	42371	35028	40330	35843	High
3450	2213	2561	3967	2298	4563	5536	6121	4422	High
38885	53010	44148	52311	41456	49714	42544	48164	39368	High
38694	43388	37089	40379	37704	30834	26750	30412	26563	High
67595	53180	41705	51338	42411	59814	47298	58093	46756	High
21677	42904	40397	43103	37698	70676	61889	70392	61310	High
59535	56601	51516	63613	56322	54181	46604	58732	52678	High
54343	33924	26467	33102	32456	39770	30631	36014	36064	High
24524	26940	22461	28392	25517	16371	13124	16010	15308	High
44251	63323	54682	59593	54761	45606	37104	40037	39168	High
59831	42564	39153	44729	42362	54926	49761	69545	53207	High
34220	30218	30232	30173	27574	33235	31050	32909	29788	High
57276	89203	50233	65826	80264	75809	45720	58686	67971	High
65175	52330	43968	47295	43507	45595	34203	39432	37036	High
36513	44463	47771	42835	33273	36994	37341	35481	28156	High
29870	57107	41645	47356	41918	54441	40046	49027	38624	High
26499	37361	28921	31968	32255	30970	24909	25109	25646	High
40241	33298	33204	34206	29581	27234	25489	28057	24265	High
50447	60116	54934	62432	50662	44567	38797	45724	35702	High
20732	28280	28288	29783	28819	16892	16771	17815	17685	High
35067	37882	33722	34388	35149	61791	53978	55875	57344	High
57416	66853	62918	64716	61153	59032	52384	63599	54619	High
48000	43620	42224	46952	44019	46083	45153	48435	48317	High
7127									High
16297	14643	18154	24834	19046	15390	19537	26953	21482	High
89221	68052	56850	94470	72639	48158	40845	67796	51033	High
27346	47494	42507	47777	43331	44435	41155	41937	43185	High

52627	42886	42325	56723	47920	27756	28818	33134	32006	High
30237	31729	31326	29620	27386	25434	23494	23958	22307	High
34024	29541	29611	28453	24917	35955	37120	35310	30229	High
58711	57367	63356	76494	56715	56795	60137	73462	56112	High
17696	21578	21593	20530	17620	12983	12123	14019	11768	High
41239	65259	54954	66643	56655	55334	53128	54493	50753	High
57758	41814	43920	48091	45768	43615	48983	54237	47334	High
919	1530	1412	1373	1322					High
3132	4199	3256	4491	3762					High
33796	58817	53488	66088	52675	42439	35246	39082	34307	High
11249	14224	12690	10966	11624	20541	17898	18928	17506	High
37071	125	141	165	128	13288	8768	13339	10893	High
30707	31419	27381	27874	27644	18510	14654	15506	14538	High
31829	44415	32228	38378	42147	26497	20517	25376	24588	High
39269	45676	46591	50879	55363	35354	38165	39169	45608	High
43573	36805	29078	32456	31204	50531	41620	44681	44206	High
39240	66781	52744	66665	51989	36941	29510	36095	27810	High
42146	39452	34864	39419	34448	31799	26411	28428	25996	High
43547	50894	47231	47790	39541	31368	30519	29242	25073	High
26103	26534	22446	24928	23827	20142	16694	19760	18467	High
19391	27413	24194	25491	23338	42400	36996	39343	37456	High
6621	6896	5925	7359	6349	7018	6211	7778	6937	High
51892	35856	29966	34934	29904	43642	35851	40402	34728	High
35178	65807	56739	65267	48158	59779	60521	62100	44952	High
5591	1803	2340	2682	1902	2878	3371	3219	2602	High
4663	7908	5810	7424	7550	13212	9875	12619	13127	High
33478	49503	46623	46601	36669	45298	43033	42128	33877	High
56221	58119	49263	64413	55348	77076	65533	84339	76413	High
38108	44905	49201	58019	48944	42126	39534	58928	43424	High
	2395	2835	2393	2249					Not Found
6623	6379	10155	11565	8746	3416	3685	3791	3272	High
1391									High
47735	47333	44794	41024	44000	30934	27033	27377	28356	High
37239	69138	75735	82889	65945	30133	29164	36471	26444	High
41733	50030	43557	50688	41897	43506	38369	42708	37691	High
31773	15377	15338	12069	15286	23675	23159	21849	22523	High
45574	27306	29061	24924	34387	35708	37032	34429	45011	High
19527	17635	20085	17677	17760	11040	9696	10081	9855	High
55654	101918	95282	98642	102012	56634	53581	59864	58423	High
55955	39746	37895	38579	39795	36752	34246	37158	35795	High
7642	7150	7438	6154	6859	9762	9798	8574	8532	High
33073	35797	30552	39003	31650	36128	28375	34385	32991	High
41726	59343	54892	66445	52539	30512	27757	32630	26440	High
27972	72083	54195	72137	63868	67945	56245	67436	66904	High
60096	42418	37600	44569	37709	46739	42342	53324	43436	High
43400	46660	40025	48581	41627	45925	40853	43518	39700	High
4435					4144	3754	4143	3729	High
21318	39320	38090	41103	36905	26578	27261	25428	24880	High
56432	36258	25955	33840	29791	40265	28237	35150	31758	High
51605	40943	45104	40696	35269	50937	51919	50381	44539	High

25737	37384	34238	35916	33406	23958	21281	22666	20547	High
26893	36222	35586	31252	35383	29790	30476	29443	28961	High
52518	70380	60655	69097	68538	22134	20253	26052	21879	High
30420	24071	20100	20923	22286	36761	35346	34647	34309	High
13396	9805	11885	12400	11566	19159	22178	27589	23919	High
69579	46047	42212	46265	44663	50945	49404	50591	50585	High
44131	62063	43678	49036	52495	21877	17452	23788	18608	High
43032	42203	38260	41302	39319	67510	66168	64848	69058	High
4269	6817	5796	6078	5575	10615	9292	9384	8827	High
69103	41444	34389	41580	36488	35542	30681	38088	33819	High
67061	38199	32655	38777	32553	40831	33449	39708	32513	High
44816	26438	25795	25668	21971	44655	40238	43641	34650	High
48966	53556	47232	47148	47842	47659	41014	45056	43145	High
58514	66722	45165	61684	52006	70147	46729	66271	54259	High
8760	18566	16338	17455	15451	16540	18426	18046	16852	High
24310	31070	34961	32916	39985	8902	10025	9126	9790	High
31666	85880	76073	88152	57206	86790	74126	76189	56241	High
38612	43508	42319	39996	41135	27761	25730	24802	27012	High
53392	49554	38022	43332	41641	64907	54925	63527	58239	High
21852	36131	31081	36897	30457	17920	14986	17726	14740	High
3091	5511	5851	6431	5046	2219	2393	2436	1857	High
44646	39703	34722	40637	35493	28550	24981	26897	24113	High
23775	28002	24301	28642	26340	30202	22576	27469	25757	High
57124	53478	53413	52088	48517	44156	43618	40510	38152	High
15922	19597	20306	19746	18787	36214	34389	33395	32286	High
21288	28045	26159	27249	27618	28927	26127	30361	26396	High
8234	8917	6995	8801	7636	9822	7378	9682	8296	High
	2537	2570	2499	2265					Not Found
36354	18391	17913	20169	19460	40747	40026	43071	39420	High
32444	33050	28767	30830	30331	37823	34162	36631	34930	High
41263	27905	23484	25378	27555	20727	16487	19130	19510	High
16679	42496	36197	36297	32602	20955	17812	20194	15896	High
39295	38719	36804	38781	37876	38227	30495	34153	35842	High
12771	5021	4933	4278	3944	18506	16551	13826	12983	High
33688	37037	30728	33036	38525	52382	41339	46635	49915	High
31590	29623	26072	25268	23521	33199	28901	28540	26531	High
47464	25263	27562	31451	28450	14924	15878	16022	14357	High
40818	55080	51468	55244	50111	36776	41820	35334	33654	High
13799	4659	3825	4542	3662	7813	8524	7722	7769	High
16887	13503	11369	14566	10741	18016	14242	15709	15289	High
22343	60229	51386	51856	55829	50174	45301	44429	46767	High
31353	48590	36825	40628	46265	22009	21662	22192	24052	High
21902	24017	23533	27532	23643	31617	27017	34434	29125	High
56439	35246	31042	34696	34430	47743	42787	45877	46134	High
31606	53168	33845	56562	37305	57186	33972	59842	37841	High
37758	50564	43293	47743	49727	27464	24360	27277	26811	High
23885	18963	22547	21019	18552	42757	40888	42565	37216	High
43022	59132	57952	59209	42850	23906	22776	23931	18447	High
22583	35727	35132	29491	33649	16045	14446	12850	14810	High
45491	36387	35319	38475	32316	27662	24475	29928	22643	High

34643	31522	25077	33897	25161	42357	42636	54206	40612	High
2908	4981	4948	4879	4172	2322	2292	2565	2061	High
26159	25480	26574	29063	25225	25426	25891	29911	24612	High
34537	35335	33664	31598	32405	32510	29324	33640	30254	High
45710	10564	5976	9233	7110	29616	19590	33446	22771	High
34072	73355	70513	68478	66726	46793	43833	47001	43722	High
5017	21366	17984	19695	17294	9061	8596	9306	7737	High
24916	38920	38577	47196	41155	44360	50205	50957	47903	High
46831	71269	62291	77058	67955	52817	46782	56314	49495	High
1881	13560	11632	11261	12761	19808	17036	14782	17913	High
18606	39720	41772	44804	39213	43723	37527	48704	39082	High
33947	24623	23364	25894	22127	56426	48144	54324	45903	High
36005	48595	43085	44793	36943	31929	26402	27574	23387	High
38417	38841	39748	32454	33831	53181	49119	46808	43959	High
32596	36084	30582	33468	29948	44760	35278	35643	34179	High
28497	23385	21316	24585	22391	11551	12297	13792	13519	High
55867	34906	36072	36431	38130	26519	33877	29096	34762	High
33862	36159	32468	32802	32046	34429	32758	30535	33058	High
27330	16803	14311	17235	15238	28223	20201	23674	23655	High
29151	46727	41080	40035	42918	26668	22643	22723	23689	High
48360	50515	44306	49089	36990	63112	57962	60708	49699	High
33459	44059	31000	38642	35702	55115	41484	49793	45068	High
38059	32813	26966	32128	33511	24488	19560	24105	23126	High
18779	35530	29004	32845	26419	20563	16633	20060	15199	High
41413	43212	34705	39601	30401	69680	52603	65569	48399	High
36103	36302	34753	37047	30335	48704	48217	49816	43318	High
	3389	3452	3238	2350					Not Found
58248	43825	44954	44544	42956	46398	43679	52780	43843	High
33663	30332	28263	32109	26875	41482	39417	42046	37533	High
27600	24363	20890	23996	20563	18924	14879	20325	15488	High
9558	14731	15518	14266	13140	8570	8884	8485	7523	High
48397	24194	19935	22433	20877	43202	37610	37648	38376	High
5113	4359	3932	4425	3464	7047	7314	7664	5981	High
19641	31102	28747	30549	25584	31200	27869	28118	25332	High
39422	36851	25726	31463	31917	24455	15237	22357	19837	High
18315	18241	21326	19780	19443	14315	14997	15552	14748	High
51967	35469	26104	32935	36623	26231	18822	26684	24083	High
37426	24379	19221	23211	22732	30120	28723	24844	26909	High
14017	9860	8459	7228	9411	12247	9811	9454	12096	High
21754	31751	23737	27854	27598	26319	22271	25814	23984	High
2744									High
34135	38794	34390	38364	35354	41249	35039	41992	36770	High
23168	20064	23067	20830	22089	31282	26371	29733	29088	High
19935	37740	37159	39368	29419	38306	38534	42108	31317	High
35246	34822	24640	33808	27331	51880	36994	44409	41178	High
5612	8150	6637	7145	6268	11228	10686	10065	9009	High
29201	43417	39187	44095	39522	50655	47000	50341	43224	High
21970	15152	14831	15914	15277	38527	33873	37792	36766	High
25603	19290	17749	21316	15777	28415	23456	26798	23643	High
36032	42999	36888	40220	35712	38254	36939	36515	34653	High

9680	4388	5313	3740	3133	2332	3232	2903	1794	High
20191	16692	16376	17808	15653	25805	24347	24892	22827	High
28439	16557	14259	15476	15151	24009	20717	22133	22191	High
	712	715	732	575					Not Found
32203	50429	45789	49340	48270	34898	31513	35912	33511	High
13500	8366	8482	10310	8467	21614	17645	20861	19587	High
37479	23031	21047	24788	21997	37193	34061	39223	37569	High
35400	33453	31454	34857	32066	50348	46374	51332	45651	High
26565	21425	20550	19270	18137	39430	40000	35080	31600	High
31596	37584	31908	37629	34848	39781	33597	34737	34098	High
26619	25367	20163	20214	22245	42534	34923	35279	37294	High
32719	41567	33573	33477	35318	22666	19778	19455	20414	High
7870	23121	18316	20854	19397	18842	16829	16983	17062	High
16583	18115	19188	26853	20453	17020	18569	24170	16841	High
32462	23172	19791	26017	21266	59693	51061	61301	56997	High
4428	6325	6128	5879	3888	17034	17367	16186	10424	High
47073	46354	34736	37825	39191	39582	35600	38134	39015	High
42816	28314	26551	24214	26890	31340	28378	36221	28449	High
35178	45325	34075	43020	36067	41191	33333	42192	35790	High
31273	44500	37199	45592	38594	39831	33183	38149	33504	High
33881	53662	44590	48635	47435	33089	35004	35403	34249	High
27411	18025	19234	22790	18937	39389	35892	46152	36286	High
4394	2796	2692	2964	2307					High
46511	36460	29825	31486	32133	48366	37718	42899	46577	High
32702	33352	31249	33204	27445	26164	26190	31065	23797	High
38287	20305	22699	23667	21476	41199	39312	43142	39448	High
14700	18507	17872	18146	17589	21836	19849	20115	19739	High
41742	43555	38960	50853	41528	50865	45323	54971	47178	High
25717	25547	22862	27673	22352	36543	34233	38306	31632	High
9236	53744	54253	58640	49745	18610	19664	20839	18442	High
23560	39060	38312	47663	39345	34645	27628	35378	31210	High
20457	27583	28392	26810	26983	28840	28654	27733	29279	High
35486	66054	61166	69194	54756	32902	33121	36725	30424	High
33525	17625	12207	16259	12800	28030	25680	28911	23630	High
37953	18367	17363	23132	19714	17459	17721	20643	19521	High
35450	32983	28984	32232	31152	36421	32097	33829	34628	High
21189	20532	18357	19757	18789	19352	16361	17422	16122	High
23955	41763	37146	39502	32804	34087	27614	32200	25057	High
24944	10404	7350	10176	11046	27153	21702	26507	23941	High
21197	35279	26257	41012	27721	27968	27864	32975	26172	High
8777	16240	14187	13983	16037	10857	9386	8830	10467	High
6922	3652	3179	3190	2971	8847	7381	7745	7217	High
					5603	4706	5575	4104	Not Found
26859	36370	30407	32583	31012	28292	22779	24994	23520	High
29517	24877	19728	22631	22690	59764	46646	56732	51321	High
12897	17468	17613	16667	17906	21065	19490	24158	20523	High
43855	49552	42773	47757	40927	27979	22977	26924	23273	High
29738	56911	54330	58829	51781	36299	33233	39555	31114	High
					4308	3288	3849	3296	Not Found
26385	22197	20938	23405	19474	26115	24137	27139	23398	High

37958	23304	18305	22717	22655	17528	15368	18155	19151	High
28236	33965	30098	29688	27872	18758	18858	17660	15983	High
26945	27932	28983	31167	29585	24296	27666	31157	28927	High
17573	23620	26893	26897	26222	26191	32260	29111	30459	High
18449	22267	21408	24142	20149	17037	16005	16815	14741	High
	1423	1159	1220	1028	5264	4737	5022	4188	Not Found
	1984	2009	2100	1769	1514	1813	1369	1577	Not Found
46165	39694	31859	39203	36777	49746	41101	49508	48208	High
6387	3968	2272	3084	2880	3651	2107	2894	2924	High
24001	38159	44384	24314	35620	28631	32349	17749	25636	High
23417	22496	14285	18044	15562	51098	37960	45983	42462	High
27597	26368	24988	31128	21996	36578	35106	42469	32211	High
28799	61513	70534	62025	64429	51163	46734	47510	49198	High
13165	9606	9578	9717	8649	18340	16920	17727	17135	High
39228	35053	34526	38737	34319	52558	52931	56266	49842	High
23565	40470	40003	44422	38533	28401	25189	31276	25876	High
10149	12488	11368	12320	8363	27576	24456	26593	20148	High
26922	24381	26955	26477	21666	33466	34295	36033	28449	High
11933	18677	16511	17665	15339	26162	26276	27036	22386	High
15017	17098	15248	16786	14833	27406	23967	24336	22995	High
26573	32536	32877	29782	28656	32341	31024	31461	27819	High
37547	36503	29206	34043	28076	40167	31961	36411	30964	High
23055	25802	23289	26134	22371	30670	26834	30941	26248	High
38785	31881	29284	27392	27535	31675	26714	26603	26016	High
27159	38175	39891	39633	35835	36837	36565	36846	34057	High
34257	35732	33825	38502	33288	33953	29576	33982	29618	High
21691	33115	29294	33308	29227	42878	36692	43014	37241	High
31784	25185	22677	23937	24644	27246	26810	26861	27419	High
33242	31275	31849	33360	28448	26059	26559	29576	24328	High
42871	44282	46938	56424	50117	27337	28788	33240	29013	High
6068	3112	3669	3640	3122	3388	3899	3937	3631	High
46204	22393	22389	22907	18592	21890	22078	22946	18578	High
1626	1041	1045	945	1002	1841	2075	1734	1868	High
22615	33720	26386	27489	24566	45185	36017	36948	33476	High
23208	40564	34792	34529	42977	38618	35209	38187	41980	High
8550	8070	8664	11159	8205	9318	9961	12054	9059	High
37566	13809	10710	14867	11746	18083	16923	20119	16845	High
13590	49481	40811	49016	41605	25726	20217	24810	22114	High
30044	45578	64758	49843	38930	25527	35522	28171	20708	High
5320	19968	7535	24485	7782	24368	9357	27391	9215	High
27818	29765	23884	29737	25132	24926	17366	22454	19652	High
29688	33448	26449	34675	29064	26849	22649	29732	24468	High
1641	1545	1708	1451	1397					High
28699	27683	21729	24289	23240	38386	31935	37301	33142	High
17585	32077	28605	27025	25255	37819	35993	29884	31981	High
30939	30565	27348	31978	29408	28199	25386	28174	27623	High
37621	34389	28883	33355	27867	36458	33335	33248	31462	High
22219	14559	15437	14690	12707	26425	24885	25507	23437	High
26988	22477	14789	18923	19743	23996	16868	20638	20874	High
19823	32104	30028	26977	27306	26670	27092	22727	23631	High

33874	31803	35160	40193	32586	29849	40697	42026	35030	High
31566	31845	27771	35227	30473	42467	35634	40416	39023	High
11737	31777	23801	27969	28233	24590	19269	22255	20059	High
29633	27736	21795	27557	24622	33028	26255	31569	29404	High
27429	25683	27238	28152	24496	39598	41256	44447	38509	High
39646	20349	18436	21946	18056	50311	47137	51457	46302	High
31244	25029	24966	25105	23112	21555	18331	20725	17950	High
14494	37014	31706	35358	30953	31759	26529	30474	26165	High
33828	24777	23819	25878	18893	23585	23449	24766	20613	High
4145					1343	1431	1415	1205	High
18773	18395	19062	17794	18422	27720	26073	27902	25970	High
22441	25783	24778	25715	21269	24728	23952	23939	20234	High
19774	18175	17877	16388	15748	23642	23555	25499	20113	High
20363	21291	19410	19852	20143	32837	31465	31466	31611	High
28548	28284	27027	26808	28657	46814	37953	39584	44197	High
27894	32972	31307	33007	32890	49389	45808	46189	45879	High
									Not Found
14124	22572	20755	22227	19501	22854	19956	24664	18835	High
29034	32356	26782	29620	28192	17741	13513	15957	14891	High
5734					5826	4332	5438	5557	High
27768	22606	18214	20973	16903	38689	31774	36280	30969	High
20505	24999	30833	32246	23401	30790	32450	34526	26326	High
43378	62130	48727	75419	46643	44158	30002	49014	29401	High
11926	26572	17971	24784	20574	18793	13234	16630	13451	High
28143	29953	24767	24165	25781	37743	29177	31421	32122	High
9795	31656	23174	26433	23872	60383	42093	48873	47400	High
26369	26299	23390	25112	21419	32345	25220	29943	24638	High
20458	27642	26660	27127	24497	32634	29609	30952	27836	High
32265	38110	36240	39767	37191	30081	29143	31328	29534	High
24875	13991	13065	13866	12026	17738	17233	17378	15006	High
									High
30508	18049	15027	16193	16269	36562	29817	31342	32516	High
30667	24909	19747	24633	20032	44002	39162	49363	39610	High
19442	26884	28346	27495	30375	21235	20821	17739	21124	High
16607	21757	20048	21812	22890	19747	17982	18427	20061	High
6432	14915	14481	13300	15405	20972	18871	19366	21399	High
11922	19387	20055	26719	18986	5437	5498	7419	5454	High
18852	18981	19409	20328	17127	18939	19999	20654	17380	High
7717	4440	6231	5272	5196	8140	8313	7884	7433	High
21134	23616	21477	21798	19376	20198	20759	20869	18275	High
22918	16706	13542	13931	13688	26088	21838	22595	22193	High
31157	27762	23862	23252	23628	14748	13850	12916	12832	High
19800	23281	23030	23856	20881	33727	35037	35124	28576	High
19084	26986	24430	24075	24243	13944	10990	11454	11785	High
30686	21744	22682	22047	21400	19584	17996	17193	18827	High
8802	14307	15724	13903	14646	12672	12552	11618	11680	High
16234	10810	11240	12292	9653	13905	12373	14805	11371	High
25927	20503	16960	17083	17053	25421	20656	20068	20445	High
22730	27426	20979	26296	24900	33441	28029	32134	30985	High
44470	23155	22365	21463	18963	26209	22512	22865	20771	High

10690	15818	12839	14433	12449	21571	18592	20169	18091	High
28763	36327	32185	32038	32917	32892	31474	29122	29921	High
35562	26847	22670	22463	20817	28666	25814	25688	23225	High
3670	3600	3664	3421	3581	4284	3853	4072	4165	High
22886	30131	27955	32090	25720	26367	24292	27140	22630	High
32921	36897	38557	38138	35724	41202	38097	39880	37643	High
23603	39039	34175	34129	29901	31587	26354	26394	23881	High
9547	9360	8200	9440	9602	11101	9955	11065	10262	High
10073	17117	14611	16084	14066	15731	14324	15823	14323	High
27786	21328	16523	18330	15722	46446	39453	43775	36362	High
28784	23106	27746	23449	17753	19736	25957	18583	15659	High
	3274	2689	2840	2843	2690	2068	1997	2079	Not Found
7431	18746	19976	19618	15907	18054	19849	17393	15734	High
5058	5849	4201	4148	4802	6322	4641	4315	5159	High
44107	24683	25209	25764	25178	28364	25814	28322	26623	High
38231	17911	13264	22081	15241	25308	22432	32533	27242	High
	1951	2045	1833	1765	1520	1730	1725	1380	Not Found
4829	35239	6959	37505	8890	38925	7798	44607	9942	High
18573	39695	28129	35767	33333	15712	13760	15483	14339	High
23438	29454	25041	30894	26938	28856	21188	25343	25977	High
13490	22878	21566	23939	19892	20037	17703	18561	16981	High
23818	22316	15910	20746	21795	20669	15084	19336	19545	High
8706	11016	12448	11833	11847	7257	8115	7949	7426	High
11034	25390	20056	21356	24188	10348	9490	9186	11759	High
20338	12653	12357	12268	11184	15467	14331	14546	14280	High
5245	6686	5102	5773	5030	5070	3764	4442	3813	High
17881	35484	36436	34659	33187	21664	18715	20529	18156	High
13313	12412	12081	13484	12418	16123	15734	15852	16529	High
15462	26629	24139	25947	26125	21854	18769	19643	20768	High
6314	14464	13629	14930	13446	14190	16214	15558	13394	High
29874	18011	15976	19315	18031	33066	30360	37351	34748	High
19501	22027	19826	22794	17585	18684	18217	19730	16170	High
20396	26053	22849	24311	23248	32145	28411	30516	27730	High
5817	11288	8357	11591	10346	9491	8371	11111	9822	High
21372	24282	18347	21442	21594	34705	27833	32549	31977	High
18115	23816	22224	25077	20186	50130	46464	53031	47324	High
29369	24107	20509	21639	22115	25997	22889	23935	24443	High
17180	9629	12840	16024	10821	10846	13785	17905	11741	High
18478	19766	17623	18999	18851	19740	16846	18545	18648	High
12458	28579	23597	25322	18622	24334	22064	23063	16934	High
32926	28889	29390	28403	25029	32397	33701	31193	28285	High
24160	27887	22907	26317	23346	14466	12588	12547	12926	High
16652	26408	21901	24888	24449	17603	15448	16928	16256	High
19393	13513	14538	11569	11445	21679	20612	21003	19164	High
18588	18285	14180	18704	15990	22273	17976	21602	18011	High
18535	16577	14729	15132	14290	10305	10054	8839	9407	High
	22506	17960	20603	20309					Not Found
9715	12984	9317	12420	11205	3526	2848	3774	3250	High
9098	9056	10233	10002	9720	11661	12160	11846	11809	High
19728	29864	22655	26420	23928	20506	18780	20731	18975	High

17686	19925	16345	16742	16095	20564	16274	17750	16736	High
31535	23032	15112	18422	18422	22440	16174	20164	19740	High
8607	50695	42805	45293	37678	25290	23069	22980	19722	High
15471	34443	32455	33908	32926	24590	22250	24019	22999	High
20168	11728	15483	14252	13738	13023	14984	14870	14949	High
20907	34099	27004	35773	31082	21203	19827	20114	18881	High
35452	26791	19252	26892	23547	55422	39995	51132	47470	High
27921	30742	29486	34955	29590	45545	40668	45928	40177	High
15924	20876	19481	21758	20506	19287	16102	19254	19017	High
21017	15567	16233	22441	14424	24379	22956	27254	21425	High
19157	29471	28800	28578	25264	30115	29611	29132	26283	High
12835	10001	7841	8641	7554	7770	6118	6147	6050	High
35077	20059	21461	14721	20929	34819	33593	22315	35883	High
12083	20040	18505	21468	19086	11844	11579	11929	10787	High
27957	25124	20250	23652	18946	34549	29293	28334	25153	High
28728	39310	33831	32863	34930	18884	16081	16120	16069	High
15561	35415	26456	31753	30943	24809	16498	22109	20706	High
26830	18573	17489	17645	15901	11681	13845	15556	10929	High
6189	2429	1961	2242	2162	5247	3941	4437	4681	High
21499	19596	16763	18597	19485	31140	29409	31842	35302	High
23380	14411	13944	15285	15098	17544	19146	23748	21268	High
17287	20801	21621	23266	22795	29002	27273	28022	28306	High
25015	30127	22791	30631	26313	35836	28389	32914	32693	High
24566	17233	20087	15854	13003	24311	27642	22720	17468	High
15076	24134	24991	19124	21141	13945	16761	16213	13876	High
2408	4220	2846	3161	3369	4463	2844	3160	3298	High
12462	12589	13942	12420	9154	24125	30254	23680	18227	High
20219	21496	17787	23670	17689	40643	34104	43842	35480	High
									Not Found
26526	26461	24191	24139	22944	24401	21522	23009	20958	High
23966	31189	28997	28180	26505	40624	36080	34686	33947	High
1641	1364	910	5098	885					High
11533	23017	21047	21793	21263	31536	26632	29415	28257	High
5689	15674	14285	13833	13015	11548	11012	11144	9601	High
28552	22545	20083	22231	17789	11046	11066	11016	9229	High
25093	27145	24265	29819	28184	21032	16624	19117	18447	High
21644	29678	25568	27636	23991	36890	29685	31444	29705	High
27075	24504	18434	30339	16082	33655	25603	41509	24325	High
20204	18102	16454	14577	19289	22094	19759	16651	23356	High
9632	14499	16870	16015	14119	15367	17993	17849	14987	High
36407	29130	21188	31060	24403	23697	18396	25937	20915	High
23525	9473	12855	9379	9741	11080	13915	12009	11091	High
26161	31296	31661	32069	34424	32537	32349	33596	35817	High
35112	39402	28299	40553	40444	23641	18175	26566	24370	High
30895	14424	12208	15633	13089	20109	16657	21355	17991	High
22439	22087	20806	20941	20903	21791	20102	20358	19901	High
11486	21460	21122	21135	19809	20465	17306	19705	17938	High
2752									High
11116	16178	12955	16085	15079	7993	6799	7560	7205	High
18930	22126	17220	21541	20891	18427	14496	17275	18013	High

31295	19336	18757	19826	17793	25258	22672	24786	22320	High
31263	9360	7701	9090	8319	15140	13410	14346	13870	High
24082	24136	19880	22610	21896	24736	18886	20893	21373	High
16260	25006	23000	28554	23022	23156	20796	23879	20242	High
11834	23581	18397	22569	18573	28219	22826	23315	19891	High
4780	7116	7125	6966	6461	5259	5496	4588	4682	High
12010	25173	28931	20145	24216	30628	35984	23787	29554	High
17531	16026	15958	16561	14362	20429	16191	19596	17212	High
20786	50816	39715	49542	43093	21438	16921	20574	18327	High
28333	17100	20794	23747	20641	27297	33558	28197	31099	High
15240	20973	18973	19420	17689	10847	9024	9832	8776	High
39308	22432	19344	24773	22124	38783	34173	38377	39204	High
14508	44093	35914	40692	36662	26989	23565	22595	24629	High
24909	37164	31047	38836	29769	25583	24048	28677	20868	High
51333	38501	30662	47950	35378	29278	23580	35241	26489	High
18368	17064	15690	18294	17655	20557	15596	21976	19973	High
9937	7937	8496	9536	7948	11213	10035	13279	10123	High
22401	11498	10895	11902	8897	18224	16076	16771	14283	High
19699	27039	23929	25200	23657	49314	42896	42280	42197	High
23912	32279	28384	26383	25635	21453	15475	16345	15804	High
4954	14994	10946	28305	11770	3827	2946	9751	2757	High
35512	35399	42294	47065	38697	39622	33607	51489	33356	High
15412	23076	19177	25789	19075	16200	12625	16669	12871	High
26899	28579	21118	29359	23504	48335	39750	50656	40616	High
21635	18752	19461	33718	24707	24991	26917	37499	34110	High
24999	16953	14265	14869	14750	26507	21788	20449	21406	High
11222	17035	15630	12649	16469	19293	19452	17123	19651	High
20222	37382	27685	28861	30043	22085	16640	17273	17877	High
8539	9654	7541	8736	9715	16477	15385	16358	15939	High
904	4812	1946	9091	2431	10439	2084	14135	2856	High
15406	21144	19744	22857	20526	23571	21830	25155	22754	High
9278	14944	11914	12076	12637	12742	9578	11074	10693	High
16613	14968	15995	16252	16297	7383	9164	8297	8053	High
8268	30668	26586	30175	25515	6669	7124	7083	6003	High
21233	13702	13105	13109	12941	25584	23347	22681	23525	High
31042	27465	25416	27164	23722	13701	13789	16432	14485	High
11749	13756	15196	14265	12737	8359	9186	8860	8163	High
10844	25701	20274	22718	20166	19721	14333	16505	15397	High
11335	10070	10606	9473	9332	19758	17918	18296	17839	High
19111	21566	19276	23298	24031	15631	14959	17706	16887	High
21623	45383	32751	36981	37350	49101	32153	38847	39095	High
16103	10183	8449	9353	7801	24353	18857	20538	17829	High
					1446	1258	2879	1280	Not Found
21614	35592	30264	39107	30734	28033	28312	29857	26402	High
18878	9204	9098	10690	8450	15079	12504	15703	13252	High
16329	26340	22460	36250	21719	27368	25329	25196	23042	High
21744	9413	8270	7957	6737	20929	15608	14020	17598	High
18785	18629	16186	15450	14499	26562	22088	22095	19793	High
8884	34812	28127	32554	30570	15847	13291	13458	14041	High
28820	27647	34467	29941	33548	10177	13535	10066	12955	High

13266	13422	13516	12779	12805	11117	10439	9830	9563	High
19639	11189	11431	10269	8481	37631	40965	37749	29934	High
14563	29099	24631	27761	23769	12461	11891	13950	11374	High
10899	16424	13931	13663	12011	29129	24961	24940	23089	High
4019	6250	5390	6395	5105	9696	7935	9652	7406	High
22058	15941	14464	16704	13003	22934	21273	24151	20408	High
11196	16616	13658	16129	14111	15431	12607	15416	13386	High
7992	21487	19441	22382	17635	18515	16875	19291	15294	High
9888	15170	12299	14491	12708	15550	12903	12549	13383	High
13201	21088	18059	18323	18261	28712	24340	26547	24547	High
12395	22507	20203	22581	20032	17444	15258	18558	15481	High
9919	16818	17313	17824	14813	8168	8720	9955	7647	High
45958	35243	25490	29995	32370	32797	22927	27405	29667	High
38329	14550	15226	17322	15710	27434	24263	31288	26412	High
26151	21625	18573	23792	19080	17033	12710	18163	13921	High
11067	25398	23687	22744	21715	13317	12093	12902	11871	High
									Not Found
9714	15949	14193	13762	15470	9117	7650	7869	8658	High
22801	21593	19931	20777	19796	10088	9768	10670	9575	High
28413	25417	25190	30457	23540	20511	20343	22973	19092	High
14248	19933	15199	20170	14416	13437	8362	11768	8802	High
26315	17523	20234	17326	13823	13550	13840	13050	9503	High
3201	4022	3008	3533	3120	4621	3516	3887	3723	High
24583	17013	16415	15720	15865	22054	19630	19606	20503	High
22597	25074	19708	19530	22163	18577	14898	15793	16657	High
16593	21531	18333	23788	20282	23115	20404	23443	21899	High
8858	13818	12098	11213	11756	35723	31552	34999	29528	High
16959	22550	17620	22099	18431	17022	13269	18553	14891	High
2297	15988	15554	17126	14373	17749	14523	16310	14604	High
18997	25593	23589	27142	22977	27034	23287	25402	23614	High
16017	29432	23556	28911	26306	11670	10743	12122	10100	High
9595	13421	11039	12119	11790	19666	15327	16804	16434	High
16610	26857	17422	26333	16574	47235	28745	36292	28199	High
6175	3789	3986	3466	3200	7241	5924	6638	6069	High
23891	37145	27880	33358	36002	26985	21429	24741	25394	High
11932	15017	9971	23150	13448	14399	9169	18675	12855	High
185	276	213	191	205					High
12934	27423	23787	22444	25224	9627	8008	9633	8592	High
34078	21458	17912	20155	17836	40717	35735	41241	39699	High
21750	23101	19371	21716	18418	29057	23683	23575	22175	High
22603	31646	30052	28984	31221	18147	17328	16287	17511	High
6306	11468	11704	11763	11924	6854	6440	7172	6590	High
15109	16162	14045	18004	16537	20738	20023	22261	22516	High
20726	20530	16571	21737	18157	28027	24722	27373	25099	High
18862	22494	21022	21657	18630	14704	13381	14705	13291	High
18749	13392	13169	15195	13666	22973	24570	26263	22971	High
16540	39297	28404	29573	32197	14013	12624	12895	14009	High
23316	19294	17881	20949	18824	20186	22670	21375	20022	High
5321	6550	6920	6137	5758	7385	6912	6558	6458	High
18471	23995	17476	18850	16560	12929	9952	10447	8799	High

15942	36202	30797	30955	30780	27926	24210	24572	23186	High
12419	12873	12052	14286	12000	17555	15404	16960	14721	High
22617	22562	19599	31029	21514	30057	28300	35736	28815	High
13646	37845	24061	32695	23746	17845	13450	17104	11537	High
10005	14653	14270	15439	14503	21054	22923	21527	20210	High
20835	19462	18625	17127	16755	18731	17511	17268	16202	High
21350	2817	3010	3160	2851	18321	17485	18492	15917	High
12223	24813	27222	24995	24531	19009	16802	18929	17421	High
15806	29958	22468	27752	25916	17595	13893	15823	15021	High
21962	11491	13071	13522	12667	12805	14444	13773	12886	High
28879	36591	23297	28310	33449	34732	27537	34742	36245	High
1215	1257	1120	1375	1089	737	757	889	624	High
	11464	11885	8822	9089	6268	4197	3928	5087	Not Found
28506	15987	14051	17869	13413	10379	8356	10240	8608	High
19690	16997	14091	18586	14930	21079	16107	21084	17700	High
27935	23223	16143	19204	18132	34140	21631	25267	25337	High
22475	19426	18798	18819	19226	17272	15712	16936	15514	High
9267	12582	8734	16224	9231	8959	7965	12348	7945	High
23072	11925	10561	10947	9215	16500	16352	16778	13596	High
10034	8725	8539	9230	8425	18221	16161	20222	16747	High
15804	29324	22475	25069	25960	20491	17188	20071	18427	High
									High
13036	15647	15808	15158	13226	23876	17379	20554	18697	High
9928	5709	5801	6027	5212	16569	14851	15388	14142	High
830									High
29762	38661	35272	42144	36300	18685	16335	20811	17116	High
17078	11430	11879	11489	12246	12606	12512	12419	12038	High
30418	30022	24477	29330	30028	19890	16034	19947	20687	High
15362	7945	7058	7098	7689	7677	6537	7233	6841	High
16148	14021	10776	12400	11642	27565	21578	23281	22781	High
19441	18150	18950	18307	20041	6284	6439	6762	6402	High
26365	29235	23217	26116	22653	13007	11198	11062	9683	High
10546	18224	12532	12978	15539	17659	13326	14722	14893	High
7032	25826	20821	25276	23968	6568	5876	7233	5933	High
31143	37885	40177	40319	38201	25932	27423	27236	26249	High
12045	23834	20442	24885	23034	24561	20718	24791	21511	High
	1648	1679	1707	1351					Not Found
23796	18480	13984	13967	14803	19628	16300	15102	15718	High
1671	1888	2166	1562	1422	2132	2213	2006	1885	High
13630	12694	9470	11267	9498	15235	10936	13061	11191	High
13450	9980	7376	9288	8058	24112	19530	22692	20111	High
1452									High
8352	20780	16416	20639	15428	1348	990	890	959	High
8049	25313	19406	25139	23508	48999	35282	43978	43448	High
20515	31153	29315	32127	29365	32705	31249	30686	31703	High
5457	14809	14294	12892	11322	30938	25721	26907	21973	High
9500	10737	9550	9008	11536	7286	6533	6565	7300	High
17827	25758	22428	26483	24208	37656	31322	34374	34432	High
16758	29441	25667	25826	29628	35060	30947	30662	34176	High
16964	20085	16693	18680	16471	19075	17290	20051	16633	High

23194	27619	30272	32723	22962	23867	27073	31137	20881	High
29639	35006	29706	33926	32765	4358	3330	4568	3887	High
10107	41070	40014	37590	37738	28036	25060	26185	24452	High
21485	25341	23845	23628	27016	19594	17633	18289	20225	High
13117	10393	9742	10182	10678	27188	23989	24798	26351	High
24866	34441	33838	30166	27172	24229	26320	23274	21024	High
17541	9176	6293	7909	6991	12090	9429	10790	9981	High
22064	18531	17272	17771	14495	18576	17553	19626	15379	High
17380	4442	4089	4634	3809	13945	15890	19176	15391	High
16897	19937	18988	19279	18345	23604	22286	23853	23379	High
4154	5473	7297	5237	6804	16289	18600	14498	15915	High
22609	19350	17852	19120	15583	17973	18466	19073	15672	High
36393	16894	14981	14232	18196	29313	28542	26807	33813	High
14648	14145	13730	14713	11189	12564	14035	13366	10773	High
11910	12925	11879	15496	13269	32979	28795	36330	32192	High
20016	18232	17260	16170	14488	11092	12106	11669	9598	High
26948	8140	7197	6152	8181	22012	20136	19164	18638	High
32447	20981	16492	15940	16891	34884	25004	27362	28885	High
17244	21958	16217	22368	17778	14644	10973	16638	11704	High
14810	11578	10696	11133	10650	11035	10067	12847	9600	High
12868	19781	18631	22056	17460	15552	14534	19046	13822	High
8911	24791	27821	24370	23701	18445	18139	18176	16987	High
22931	9657	9602	11198	10592	16755	14206	17006	14272	High
9869	16950	16606	13432	11090	18197	19593	15090	12586	High
18740	13607	11967	15360	12231	30452	24652	26040	25261	High
18788	26714	18788	23934	20521	10593	7724	9982	8356	High
11754	30389	26716	29356	26579	26112	23259	25161	22525	High
15692	9665	7995	9093	8017	19497	14862	19177	15912	High
16788	24849	21467	21486	17475	18872	16902	17312	14282	High
8723	25843	25240	23764	25101	29608	28124	29450	29616	High
30358	15384	9877	12587	13095	33015	19269	23527	29318	High
23995	17899	14597	16359	13133	22323	15660	16034	15544	High
21965	28575	30391	27761	30028	12065	10939	10176	12368	High
27782	16439	13326	15215	12673	27664	22479	26435	21983	High
18614	25735	25400	26756	23970	33118	27217	32454	29079	High
9973	15591	13188	15537	14362	27822	20237	20904	24412	High
8438	29544	25239	24997	28531	23923	20091	20019	21837	High
13513	11895	12326	14018	12428	4235	4519	4566	4363	High
9376	6943	7548	6295	6340	9672	8888	8969	9023	High
23352	24306	21776	22239	21800	12358	9329	11363	10108	High
18103	16226	16835	20060	15808	3896	3927	4166	3309	High
18444	10869	10036	11233	9547	36265	32271	31090	31574	High
15502	9182	9668	9091	7872	15959	15392	16257	13791	High
10653	12489	9149	9380	8045	58221	36456	40492	35167	High
15445	24739	23829	29695	29179	18936	14454	19112	18267	High
26052	27793	21475	27382	23639	16083	9920	12588	12635	High
22988	20013	16040	20695	17469	7279	4688	7051	6316	High
12821	11194	9326	10439	9006	21248	19007	18653	17359	High
					2752	3409	3505	3442	Not Found
11313	11783	9686	9244	10479	12124	10421	9528	11100	High

5078	2364	3962	4205	3994	2869	5129	5915	4787	High
10479	2855	3376	3226	2484	10907	10857	10537	10103	High
8815	11189	10539	10725	8789	12571	12323	12043	10051	High
33743	26707	24873	28848	25734	23534	21357	24199	22627	High
30336	33480	24678	29562	31891	15491	12572	16079	15646	High
9114	16823	17262	14714	13805	15308	14190	14883	12997	High
55324	19192	15938	18253	21171	16712	13948	15544	17549	High
10334	20386	17175	23083	18353	23034	19287	25845	20338	High
23149	10691	10791	9302	8700	13691	18312	23525	13773	High
16657	9958	9939	9358	8801	12165	9828	9811	10225	High
15060	16116	16351	16180	13565	15057	14326	14298	12371	High
5684	6079	5552	5909	5400	6627	5410	5956	5154	High
15356	15801	17534	19779	21829	10565	10654	12467	12569	High
9958	18434	13876	15586	19874	14774	11157	11802	14701	High
13202	14086	15447	17358	12629	10259	11324	10743	9416	High
9189	8050	5956	7429	6791	6622	5061	6202	5523	High
16083	13653	12406	14076	13207	13113	12491	13451	12334	High
7477	11798	11201	10517	12950	9506	8831	8943	10194	High
8299	15813	13917	13831	13477	7066	5933	6633	6399	High
20365	15118	9900	15434	12602	33702	22433	33052	27237	High
26188	48471	44782	56064	50088	30978	30180	44923	33077	High
10371	12390	10880	12378	10960	4578	4320	3922	4249	High
12386	17362	15324	17463	14834	16309	14697	17163	14928	High
12555	14199	13240	13117	13301	9558	8726	8608	8690	High
15825	18881	19855	17822	19016	9183	9545	7875	9449	High
23891	5097	4441	7051	6087	17871	14351	20985	17620	High
22009	15809	12142	13737	13963	23362	17607	18117	19613	High
24926	26147	25121	24980	25856	18826	14161	14713	16733	High
14498	17792	15939	18820	15963	11097	10015	11844	10465	High
19386	13654	13874	13309	14265	23898	21684	21981	22564	High
6619	7008	6185	5552	5770	23618	22360	21019	21660	High
12151	5335	5193	5744	5498	7584	6427	8414	7496	High
23439	11495	8282	9740	9673	13994	12114	13534	13517	High
16300	13660	10829	12699	12216	3341	2985	4388	2971	High
12848	18060	14685	17722	14923	6860	6838	7494	6214	High
									Not Found
11153	6669	5424	6566	5319	9758	7918	8982	9053	High
14410	17812	15985	17507	16472	7715	6786	7685	7083	High
13043	9730	10476	11910	10576	14089	11006	14592	12531	High
15855	13003	12533	13541	10909	19220	19353	18015	17899	High
17905	29872	28506	29088	23468	29624	27636	27509	23052	High
15585	34794	26035	29308	29988	28220	22130	23846	24743	High
27002	14722	9626	12816	13097	30058	21441	24729	27202	High
9649	10577	9575	11015	8240	18582	17635	19490	14703	High
19239	29376	26070	26955	21926	18103	15555	17197	12752	High
9513	21102	18441	22169	16938	12099	9125	9614	10127	High
25049	11375	9690	10830	9095	11833	10831	9191	10558	High
2149	5815	5710	5098	5272	2613	3035	2173	2636	High
15868	12886	13264	13070	11406	21849	22792	23133	22254	High
20505	23404	23663	27732	22213	3518	3500	3738	3251	High

11388	20864	17997	18116	18150	8257	6494	6924	6210	High
12255	16226	13069	14583	13793	7758	7507	7937	7430	High
21140	22400	28572	25834	26602	23867	30301	25995	28422	High
15882	32778	26721	30870	32449	17864	15909	15814	16818	High
24437	32392	29699	28140	30019	17045	14647	14672	15337	High
11771	6193	5744	5668	5289	7999	7367	7862	7295	High
13113	9600	6782	8150	7185	18873	14678	16715	15054	High
23606	21210	20070	24891	19653	22731	23092	26704	21874	High
18183	15863	16408	13604	11609	30658	30423	26942	21017	High
8740	21581	15572	17741	14969	27448	21025	26251	20302	High
12858	8121	6392	7015	6512	3776	3695	4031	3448	High
6163	9206	8873	9030	8790	6577	5917	6970	5971	High
16774	20942	19473	19462	17420	13917	13842	12965	11736	High
19947	10044	8583	8763	8330	3784	4282	4558	3601	High
6468	19948	17652	16653	15877	15839	12915	14850	14348	High
12181	22536	19246	18604	18268	11742	8976	9651	9449	High
15096	11831	8909	10479	9700	16428	13379	12562	15500	High
7593	15092	13443	13447	12731	16171	12881	14502	12965	High
7466	8464	7391	8241	7899	12011	11017	10870	9833	High
19211	9903	10156	8968	11557	15316	14641	13120	17788	High
24638	27641	19076	21881	18169	30741	20347	21573	19744	High
6497	5852	5474	6364	8038	8545	7926	8825	12061	High
1905	1676	2107	2662	1815	15037	14021	15297	13298	High
18039	15046	15811	12609	14150	12421	13259	11137	11885	High
18389	39105	24037	29353	28175	8283	4109	6684	5691	High
21503	12550	12028	11468	9943	13204	11985	11620	10230	High
22123	30134	23466	25058	26700	16622	10292	11828	13074	High
16413	3747	2740	3020	2570	32697	23086	25765	23033	High
11218	24284	25568	24882	20128	8318	7939	7841	6076	High
20520	17954	14244	24816	15553	13181	9752	17768	11256	High
8756	20612	16476	22187	17074	20051	16334	22221	16790	High
15687	9441	7385	6956	7190	15384	12528	13985	12754	High
23043	9337	8743	11497	7474	18959	18708	20546	16660	High
2994	7456	8099	7660	7245	4505	5440	4873	4974	High
6593	9466	11141	10612	8475	12685	14429	13224	10678	High
13424	10888	10499	10424	9307	15117	10282	13192	11294	High
12804	20733	17789	18888	16517	15477	13392	14993	12481	High
1902	11057	9805	11016	10360	8384	8943	9978	9489	High
19662	15960	13628	15066	14974	19054	16194	16807	17585	High
6858	6679	6708	7954	6748	16034	12263	14543	13060	High
7450	16350	12435	15593	12696	6417	6210	6739	5599	High
15227	16763	14031	14178	13708	18189	15353	15361	14825	High
15559	21340	19251	21760	18863	10175	12674	12681	10738	High
11430	7636	7953	9101	6104	9723	8587	9659	7165	High
26439	14492	15814	16815	13876	13731	14002	16352	13464	High
5731	13111	8489	11591	9495	9413	5608	8220	7366	High
13744	7762	8373	10079	6582	6860	6203	7906	5748	High
12270	12002	11150	13128	9459	6872	6104	6981	5353	High
13380	12829	13029	12859	11994	10164	9447	9364	9612	High
10002	25386	22767	22421	20285	10185	10347	9832	8726	High

8689	20349	15888	19749	16701	17452	13044	16189	13512	High
11169	19120	17212	17581	14935	23771	21414	21377	19442	High
26754	26182	25003	22852	27717	15590	15455	14480	17031	High
19000	26776	25874	28031	25139	13276	13453	14036	12421	High
12099	18453	18113	18570	15270	9243	9386	8361	7922	High
11560	13270	10738	11024	12111	5016	4092	4366	4178	High
12714	16770	15653	15825	15027	16375	14789	16659	14364	High
16123	24502	19579	32333	22877	17363	14904	26812	15966	High
8687	16490	15913	17514	14160	4855	4314	5046	3783	High
8992	14184	11193	14452	12387	6467	6267	7681	6339	High
8985	6945	6038	7102	6214	6917	5759	7030	6600	High
13378	1867	1226	1498	1533	7155	5233	6570	6404	High
5521	6800	7047	6484	6341	3584	3861	3494	3462	High
5082	17285	15498	15769	16347	22776	13680	15828	20298	High
18308	5443	5288	5484	5128	13954	13072	14097	12719	High
7530	13841	11224	12267	10172	17207	13128	14193	12765	High
9840	19957	18292	18716	16020	16303	15122	16140	15095	High
14554	17371	17952	17010	15065	13804	13918	13345	12512	High
8947	19447	16628	19430	17453	15975	12255	15105	13270	High
12352	20546	13401	13998	12810	13489	8787	9069	7438	High
11377	8175	8070	7902	6881	5307	3933	4129	3991	High
16826	5943	6157	5858	4948	4561	4576	4317	3804	High
4326	7078	8347	6868	8387	4446	4971	4617	5325	High
1508					553	293	289	386	High
18250	16404	12109	12156	14272	7912	6010	6349	6794	High
11957	8521	8531	8980	8132	29770	28500	28080	27178	High
10602	9874	8603	11212	8750	10965	9190	10539	8764	High
10575	13035	11781	12683	11630	16644	13591	15937	17938	High
11088	2887	2803	2496	2430	9465	10682	10364	8976	High
2226	9722	8878	9231	8670	7497	6782	6853	6291	High
11402	4784	4673	4807	4389	15213	12490	13112	11903	High
16351	13109	10441	12028	12641	17914	13774	15997	16036	High
18643	5938	5904	6464	5294	9264	8589	8994	7677	High
14454	19541	17583	16596	16810	2163	2062	1681	1808	High
14634	13767	14220	12313	10673	17544	17610	16195	14119	High
15376	21551	15319	20566	16645	11604	8768	11682	9130	High
8608	9621	8151	9167	8629	5453	3775	4430	4110	High
3868	23029	18334	16558	16844	15712	11267	10783	10726	High
15251	13206	9933	11216	11516	26451	18318	23681	24281	High
11989	6714	6999	6577	4801	25131	31912	28594	21302	High
14693	19463	12361	16975	15923	21669	15127	23328	20215	High
27777	21518	21699	24040	25612	18611	17541	18881	21308	High
7915	13385	14598	12873	10533	22247	19587	19051	16486	High
15464	2978	3402	2880	3399	9950	8687	11473	11242	High
14105	9540	8931	11509	9360	7746	8497	9620	7865	High
9003	4174	2399	2534	2160	11992	7121	7957	6387	High
17693	21802	17457	21548	18040	26876	19054	22331	19802	High
28449	12995	12797	13934	12757	14066	11729	11274	11794	High
10679	12571	12093	14532	10664	7930	6822	7650	6491	High
9693	15405	11567	12277	13458	34023	26967	28996	28422	High

8711	8579	7601	6808	7122	4223	4196	3536	4130	High
8508	6352	7445	5365	4985	6932	6369	6136	5903	High
6809	4554	3739	4849	3673	1928	2009	2118	1758	High
6556	8907	11308	9313	9298	10616	13883	12588	11813	High
5545	12341	17230	12354	15847	27126	37704	28876	35430	High
17055	21032	15611	19732	19305	12912	12223	15247	14684	High
13689	27433	18935	21632	22763	26302	19639	20588	22503	High
20565	18912	15738	16377	19185	25500	20739	21746	25059	High
9069	8104	7536	7213	7178	7970	7134	7307	6701	High
29855	35738	44642	57969	53531	36771	41029	52670	51210	High
24639	13492	10156	11158	11297	19671	15839	17896	17154	High
2687	17771	14158	16785	11565	27421	16484	23578	16031	High
16817	13165	13453	13086	11867	13733	13248	12658	12773	High
10161	9300	9561	7521	8513	3800	3810	3956	3494	High
13220	14528	7335	11105	9146	28076	17369	22308	18504	High
14038	14256	11382	15853	12473	13194	9057	13982	11317	High
18216	11085	7936	11351	8953	8862	5749	6547	6348	High
4218	15692	17131	14134	13181	1856	2253	1820	1514	High
13390	13391	11125	11410	11915	12163	9873	11092	11064	High
15713	24297	18217	20843	18016	12578	10448	12021	10377	High
12369	11127	11875	16697	10817	4697	3813	4371	3799	High
12317	20617	15831	18182	17279	20977	17203	17483	17469	High
21281	31763	24818	24913	27806	21216	16209	17599	18173	High
10205	16055	12450	13550	13721	17227	13132	13548	13719	High
24802	20636	19389	21570	18376	14746	14321	15821	14212	High
19089	18233	17133	18428	16767	5535	5058	5630	4691	High
17538	11738	10491	10519	10997	10741	8691	9826	9228	High
11327	25337	20403	22505	21253	6156	5153	5210	4787	High
10995	14206	12528	15128	11805	19553	16485	20468	16038	High
10514	17548	17223	19432	16987	12232	12163	13560	12131	High
2564	3983	3083	5539	2745	9231	6361	11206	5001	High
26115	12426	10559	11362	10716	21144	17644	19473	18875	High
15986	14823	15190	14847	12869	8921	9150	8998	8065	High
2279	3711	3909	4186	3428	6700	6026	7185	5768	High
20056	8821	5689	5142	7972	12520	7947	7464	10407	High
11868	20981	17537	20374	16080	5335	4399	5260	4417	High
20130	9586	8495	7916	7015	24231	21644	20855	18831	High
21568	1445	1053	2200	1005	18465	15937	16294	14016	High
3455	8298	6950	7233	7281	3097	3754	3556	3069	High
3528	3544	4708	3316	3274	15014	15187	14219	11601	High
23293	15289	17645	17142	14952	15964	15411	13960	14238	High
7521	8680	4971	7123	4727	29878	16564	26571	19649	High
27669	16306	17243	12905	26493	6848	6634	5531	11595	High
14901	13804	12155	13957	13414	10300	10122	11944	10931	High
14528	17322	17054	16952	19498	11710	11753	11206	12856	High
12210	6381	7232	6237	5732	12908	11316	10695	11167	High
18083	23093	22856	22575	24568	22167	20686	18843	23474	High
2500	2687	2583	2367	2131	5091	4503	5375	4286	High
10418	20076	16744	17050	16951	15704	14770	13614	13589	High
3127	20879	15298	18926	18642	9717	7118	7975	8110	High

26571	20797	16320	17032	16158	14225	10108	10969	10371	High
8826	7441	7450	7907	6668	12501	11931	12735	11416	High
11541	12089	12446	12783	11990	21154	18354	17792	19557	High
11476	8949	7180	7798	8140	18988	19060	17854	17876	High
10820	18698	14114	17909	15411	13547	13005	14714	12359	High
10087	20078	15002	18009	15704	30318	21912	24587	27227	High
8820	18084	16288	15567	13817	13867	14729	12472	13752	High
4632	5522	5826	6102	4607	2113	3123	3326	2438	High
5655	4092	3486	4195	3267	5858	4785	6038	4820	High
12657	28815	26571	28484	24569	12710	10695	13465	10824	High
4599	6784	6640	7163	6748	9617	8442	9256	8036	High
9684	3886	3528	3461	3340	1837	1887	1830	1606	High
13399	3933	2898	3113	2941	8225	6053	6750	7064	High
8562	16486	10089	19384	12200	13097	9516	14442	9704	High
4482	23683	22050	22974	19428	18058	14183	16731	13782	High
11883	11130	10560	11764	10941	5444	6624	5693	5427	High
8592	16399	14080	12832	12895	11118	9895	9990	9290	High
23906	16734	16068	13165	15682	7108	6158	5996	6218	High
9812	6543	8293	8276	3691	12250	14168	13398	6949	High
7606	8542	7439	8634	7206	8410	8634	10679	8160	High
2113	5282	3036	3181	3697	14129	8959	8915	10726	High
18636	12675	13412	11242	11765	16145	13090	15915	13222	High
5805	12995	12734	12924	11090	11702	12185	11026	10408	High
9770	12416	10341	13897	10178	7213	10304	11200	8750	High
8825	9534	8672	9422	8488	11307	10943	10325	9798	High
6072	19360	18185	16358	14383	12195	10956	9606	8726	High
11281	15104	15141	12626	14892	16251	16050	13451	16560	High
5060	10534	7786	8163	7974	5605	4566	7008	4449	High
23419	16123	15314	16501	16968	14795	11995	28160	14553	High
14489	17360	13459	16065	14478	25575	22152	24567	24083	High
9123	15619	13467	15739	13186	14036	11917	14027	12058	High
7217	14359	15361	13838	12690	10781	10648	9537	8739	High
13009	8474	9858	7755	7564	15471	17462	17052	17533	High
7343	15546	12923	15358	12458	3143	2125	2863	2336	High
3128	19711	16823	18719	13292	1647	1375	1601	1105	High
2287	6879	7474	6833	6300	22210	19460	21284	19832	High
4145	4658	5224	3590	4708	3406	3924	3139	3284	High
11609	20999	17978	16978	19787	22830	20953	20587	24238	High
8795	8877	9673	11797	9712	2028	2148	2604	2010	High
14224	11615	11103	11529	9140	3694	3322	3465	2697	High
4938	3678	3157	2969	2877	9730	9737	9117	8136	High
7334	14106	13416	13731	12007	7498	7616	7452	6506	High
13310	11538	13804	12316	9363	21328	21761	21191	16132	High
11981	16076	13033	16015	11555	19101	18099	18186	14077	High
12772	6364	4911	6358	4842	6390	4803	5994	4986	High
8800	12160	11357	10389	11467	17315	18721	16590	17495	High
12072	9239	8458	11802	7768	18187	16716	18688	14963	High
823	22608	17986	20190	18755	14870	12176	14640	13278	High
16251	3202	2637	2741	2369	16476	12968	14228	11934	High
15922	6214	5866	6486	5170	7435	6388	6111	5661	High

11577	11203	11531	11936	11189	17778	16920	17327	16597	High
4441	10774	11056	11552	9188	7375	7405	7836	6323	High
6595	13522	9780	11661	11418	6215	5876	5899	5743	High
16127	10433	8318	8572	9855	9242	7386	7531	8132	High
6368	8906	7876	8768	6701	10262	10566	10513	8713	High
21911	16484	16419	17657	15800	19023	18088	19478	17587	High
13463	11115	10472	9357	9562	19683	17232	16169	16042	High
7788	21315	20255	20515	19688	9184	9523	9468	8549	High
7136	14809	12895	12504	9818	10901	8918	8500	6957	High
12826	11034	9828	8126	9580	5253	4902	6268	4708	High
27837	18669	15099	14481	15721	18380	16527	15671	16080	High
16486	27042	12619	19776	18070	6338	4556	5744	4823	High
2666	6190	6014	6331	6049	5317	4950	4691	4309	High
8706	3598	3834	3339	3332	12499	11621	10462	12130	High
3133	13783	5438	13627	5097	15909	11955	16072	9125	High
5212	24393	20377	18827	18060	26518	21425	20864	19257	High
10167	9915	8105	10387	7554	14367	12096	13351	10806	High
14452	14591	12283	13291	12993	11379	9861	10135	9817	High
32333	13709	14877	19472	17528	17692	15695	20150	19192	High
10698	9213	9020	9481	9719	16042	15281	16085	16830	High
9125	5625	4778	5378	4195	11773	12938	11114	9468	High
10389	21440	15492	19201	19355	10642	7030	9272	9877	High
2366	19384	21993	19858	19615	16978	17408	18223	16159	High
12063	9696	7685	8636	7963	5729	4573	4743	4579	High
3473	5993	8929	6893	7864	8214	8967	8881	7583	High
4091	4366	3666	3102	2949	7075	6350	5556	5537	High
15905	12650	11690	12588	11096	27655	22783	25208	22342	High
9040	3550	3653	3935	3617	1142	1314	1348	1363	High
8270	6066	4992	5258	4782	6402	5539	6077	5228	High
6512	12754	11045	14979	14048	7376	5698	7968	7686	High
6807	9599	8735	7724	9704	16006	13071	12842	14013	High
10516	13319	13857	13343	12852	10543	11377	11532	10517	High
3305	11685	9242	10801	9343	19427	15710	17416	15836	High
2455	5946	5513	5435	4411	15856	13957	15069	12288	High
504	12459	10656	10738	10448	15763	14417	14990	13868	High
19347	4988	5093	6208	4706	16503	14992	17092	14607	High
8342	12353	9322	13098	8741	14269	11601	14123	10256	High
3316	15681	11430	13975	14738	11149	8053	8192	8764	High
7347	18484	16375	20814	16391	10085	7732	11465	8576	High
8735	12189	9640	12372	11055	10482	7998	9840	8544	High
919	12532	9724	10564	8946	877	867	758	730	High
6247	20300	16731	16772	17528	8893	6964	6509	8161	High
17528	7152	7874	9051	8607	15500	16796	17608	16649	High
6495	8485	6397	7838	8582	8541	7961	8498	8295	High
8385	10698	9432	11676	9962	11435	11230	12801	10362	High
4513	11370	2093	12350	2661	10149	876	11741	1229	High
11766	7930	8088	7126	7013	7388	6000	5922	6245	High
2796	2901	2690	2645	2441	7130	7437	6711	6237	High
8956	9328	8587	9895	8044	10270	9396	9956	8503	High
11570	4397	4558	4377	4560	7121	6745	7268	6872	High

4284	10121	7405	7807	7722	28586	25641	24360	24175	High
11630	9784	10821	9941	8474	14662	16263	15432	12990	High
11607	21296	19311	22109	17986	9562	9276	9122	8045	High
9088	16390	12732	12548	13975	25416	22682	19923	25236	High
9183	8588	9304	8707	6775	4077	4354	4564	3936	High
9846	10329	9807	9624	10145	9845	10468	10009	10586	High
12993	6711	5230	7356	6501	12057	11043	11883	12348	High
14956	11070	8024	7383	7341	7893	6361	5519	5499	High
17996	9590	7477	8603	7770	25153	17227	19835	19304	High
8574	15192	8357	9944	10477	31098	16272	20141	20440	High
9205	17364	11350	15156	14993	22826	18111	21179	20823	High
12584	16931	15285	15162	15873	15875	14916	12883	14313	High
8080	3119	2465	2179	2903	20598	12799	16265	19783	High
9401	17455	16873	16341	14172	17106	16562	14896	12517	High
7203	20947	17944	17139	14315	18115	16132	14107	11846	High
12282	7873	6320	7548	7598	11719	8328	10789	10927	High
9325	13390	11999	11979	9727	4426	4061	3279	3071	High
4732	9581	9870	9953	8207	9294	8242	8196	7723	High
2899	1821	2136	1900	1524	1664	2073	1721	1473	High
12706	14139	12008	15406	12765	7915	6536	8352	6727	High
14312	13391	11765	13796	10476	13756	12044	13225	10193	High
2487	2263	1991	2276	1797	4465	4386	4259	3573	High
5658	3463	3758	2713	3357	11449	10452	9980	11217	High
1091	6712	7802	6956	5487	7384	6575	6628	5067	High
9984	15608	14031	15758	13729	6449	6411	7211	5640	High
15363	17045	16810	15076	17012	9514	9312	9009	10134	High
6036	6378	6081	6337	5985	8041	7486	8094	7127	High
3024	2152	2243	2281	2088	464	355	326	455	High
8195	5708	3888	4893	4137	18035	15785	17800	15199	High
13239	22608	26174	30058	22369	11338	14752	17455	12248	High
5702					4341	3245	3811	2748	High
1843	1560	1423	1784	1476	2374	2459	2730	2268	High
11345	12416	10926	11918	10403	6851	6007	5944	5381	High
11362	18528	16262	16469	15542	10231	8558	8225	8519	High
7494	16945	13252	13978	13160	16545	13930	14405	13911	High
5977	14092	10154	13455	10355	13024	9562	11856	9835	High
14164	15351	12499	14101	12674	10556	10390	10723	9919	High
5563	4407	4790	4877	4348	3081	3488	4026	3253	High
9481	11276	9734	11433	10563	11014	8039	7422	7764	High
11891	11652	9718	11112	9740	15405	15423	16472	13533	High
5348	3426	2722	2815	2619	6650	5428	6259	6030	High
5051	18507	15587	17037	12090	6258	4748	5688	4088	High
2763	10381	9458	10123	9331	13569	10501	12295	11086	High
14048	12488	9362	11540	9027	20634	18499	17945	15411	High
6961	20409	17498	17992	17220	11517	10343	10235	9806	High
11956	12256	10810	11034	9918	19576	19838	19827	18851	High
3706	3988	2799	4004	3057	9349	8529	10494	8216	High
10330	10192	8970	8148	9737	9420	7350	6059	9039	High
					9336	9207	9504	8341	High
4259	4958	4640	4218	4188	9882	9878	8791	8863	High

13662	711	695	604	526	13506	11374	13300	12339	High
4157	8490	8379	9390	7856	7012	6109	6405	6255	High
14309	6462	5457	5230	6859	25812	22682	21997	24760	High
8807	11328	10454	9156	8144	17308	16247	16614	12514	High
4909	14279	12288	11509	13832	12930	11083	10345	12905	High
14206	38402	27587	34952	29445	19518	11842	16595	13771	High
	7039	6721	7502	6030	9159	9275	9527	8559	Not Found
6570	5030	5806	5607	5970	5440	5774	5617	5553	High
9121	18073	15298	19012	15351	7700	6934	7877	6856	High
10756	380	329	470	428	19495	17445	15253	18916	High
12819	10011	7814	8752	7898	8927	6853	8081	7390	High
20195	11414	9337	9083	11290	13524	11180	12117	12749	High
19581	14477	12783	13896	12397	9092	7512	6868	6949	High
13960	15855	15825	18392	15310	6418	5772	9681	6425	High
3774	8945	7958	8526	7459	6091	5066	5408	5322	High
11993	18849	14049	14904	15718	8331	6536	9801	7178	High
9110	6212	6771	5707	5946	6463	6362	7082	7211	High
6330	6914	5526	5841	5596	7681	6032	6243	6768	High
2983	10578	9842	10861	10382	12060	10775	12200	11129	High
11280	10534	9078	10889	11044	11618	10063	10839	10844	High
15887	19837	13899	14838	17760	8802	7288	5925	8964	High
22166	1438	1438	1412	1239					High
8281	7542	7473	7899	5331	10509	8304	11740	7445	High
8015	23838	23762	23155	24373	22665	21901	20956	22075	High
8958	4934	3986	5361	4901	10877	8997	12402	11237	High
29536	15617	12647	15091	12908	11961	10004	11203	10357	High
9746	26447	21266	22055	23870	9300	5666	6502	7026	High
21707	8529	6190	6960	6161	7892	5991	6660	6003	High
7473	11955	10179	12171	10102	7754	6335	7707	6431	High
9546	12792	12657	11982	9901	3968	3473	3870	3467	High
9399	3073	3189	3639	2592	11906	10438	12949	10747	High
11564	15495	13473	19561	16896	18066	14351	21327	18072	High
8213	9057	6421	7352	6933	9488	8282	8195	8191	High
13427	6004	4260	4289	4579	30727	26769	25174	26089	High
7815	16728	14426	14905	14602	17433	14761	15910	14766	High
9093	14440	11904	15051	13006	20053	17136	20854	18445	High
4308	6418	6214	5674	5727	12089	12531	12041	12491	High
2260	19623	20743	19174	19649	2816	3290	3648	3333	High
11860	16656	15993	15721	13560	7642	7238	6812	6037	High
7407	11748	9370	9438	7416	10234	8176	8518	7038	High
5735	701	714	883	825	9288	8600	9002	8729	High
5224	12079	9729	11303	10207	4149	3062	3833	3349	High
13170	11674	11318	10049	8857	9316	7669	9026	6622	High
18596	14109	12000	11726	10927	10192	11850	12363	9622	High
20225	12635	11788	10064	10454	7136	7230	6228	6693	High
5812	28298	25993	28237	27642	26205	23976	26456	25070	High
9252	19213	15786	15718	17382	6295	6244	6345	6225	High
7035	14827	11989	11692	10753	13022	9668	11046	9570	High
14990	9073	9261	9990	7906	7568	7530	7938	6553	High
18423	12132	12482	5420	8218	11763	13882	6643	8936	High

10877	11836	12394	11082	10761	9603	11495	9993	9015	High
10019	15089	13627	12286	12842	7206	5494	6837	5840	High
1916	7573	6686	5590	6220	4251	4023	3329	3600	High
14763	14600	11737	13271	12041	11649	9764	10548	9964	High
3685	2822	2757	3020	2266	3757	3828	3537	2884	High
9182	8783	8979	7740	7083	22467	17204	16680	20488	High
7449	15593	14466	14271	9671	30694	37870	26099	22937	High
7322	3884	2783	4219	3588	12391	8849	10976	8904	High
10405	18987	16859	16798	15668	8545	9493	8485	8003	High
2775	6765	6733	7030	6062	4906	4374	4781	4309	High
3824	11157	11619	12453	10173	11957	8982	11247	9887	High
12092	9892	9188	9664	9322	6732	5639	5646	5275	High
10264	13467	10772	9400	10796	17752	14524	12853	14332	High
16757	7727	5233	7572	5988	13360	11033	13865	12156	High
16244	12566	11995	10648	10649	7409	7868	6713	6541	High
7057	5035	4443	4719	3990	11919	10464	10081	9675	High
3740	1833	1469	1336	1759					High
8462	6975	6529	6770	6500	5408	4242	5387	4901	High
9187	19817	15755	18472	17251	13808	11564	13075	11825	High
	8360	8355	9549	8184	6176	5834	6788	5156	High
10219	6570	5954	6416	5111	15387	14719	15324	12004	High
1573	4797	3913	3946	4043	3315	3180	2376	2888	High
5047	6835	5950	5768	6066	5291	5539	4704	5320	High
11881	14590	13795	12501	12856	4201	3522	3214	3720	High
23720	14871	12204	13109	13773	7443	5117	6113	6171	High
9015	21341	21183	21373	17940	19618	18461	18490	16248	High
2929	8563	8103	7636	7540	8592	7755	8404	6388	High
6304	6082	7205	6569	5824	11903	14873	13142	11485	High
15859	7403	7085	7351	6478	16177	15604	17624	14716	High
8999	11020	12371	11184	11473	9305	8693	7621	8336	High
11554	7732	6739	7723	7538	9087	9749	9480	9066	High
16362	12009	9876	9818	9812	9082	9049	7023	8069	High
3577	11940	11489	12054	11500	2330	2769	2292	2077	High
11830	15109	15261	15668	13789	4026	4263	3789	3445	High
10977	6549	5154	5440	6037	13475	11137	11942	12809	High
14136	6861	7379	8001	6707	2053	1443	1942	1812	High
8318	6254	5107	6498	4584	12681	10858	12536	9617	High
15309	16208	22056	16770	14623	11639	16398	15563	10679	High
10204	5948	5789	5812	8224	19706	13225	17817	18802	High
15090	17896	14997	13782	14653	9773	8452	7555	8305	High
5143	2986	2693	3155	2434	4314	3388	3335	3170	High
10927	18346	13623	13816	15386	8215	5347	6225	6770	High
7558	6029	4891	5560	5560	20397	17152	18281	20474	High
1042	1032	1062	1201	1007	2389	2749	2537	2597	High
16437	13378	16181	13967	12464	17965	14817	17538	14318	High
8366	8538	6550	7757	6747	10444	8278	7994	7565	High
7030	15904	15003	14742	14722	8851	8353	9008	7494	High
5349	2912	2619	2574	2469	2553	2185	2554	2117	High
5264	2892	2748	2990	2479	3366	3076	3302	2957	High
11500	10919	9468	10208	10832	10149	7858	8149	10719	High

13757	19923	16028	18632	18047	17333	13869	14879	15495	High
866	2628	2308	2415	2300	4425	3951	3661	3559	High
3259	3569	3464	3503	3087	2905	2478	2789	2560	High
	4264	3390	3277	7477	492	418	449	910	Not Found
11363	10827	7975	10106	8265	4653	4506	4406	4210	High
5536	9033	8094	6104	5914					High
4784	1036	1041	1237	930	1120	1082	1016	966	High
231	2050	2027	2247	1701	359	318	442	363	High
11709	14551	10889	14038	11731	5924	4855	5138	4997	High
2700	13123	10904	13528	11863	17090	14477	17302	15161	High
12260	12002	10483	12131	10612	18309	12202	18125	17617	High
11473	9717	8370	10040	8786	11063	7405	9417	8693	High
5675	15192	12716	14862	12150	9420	7947	9082	7531	High
12938	14748	10180	12026	11202	15642	10485	12758	12124	High
2571	9004	5730	10082	7207	11726	7982	12728	9608	High
11238	10623	8959	8738	8044	10580	10053	9393	8476	High
6448	20710	15768	20377	16461	14960	12211	13948	11999	High
3765	7742	6762	7737	7073	8607	7681	8400	7376	High
2756	8690	5890	6563	4976	14882	16114	15176	11029	High
2703	8025	7274	7513	6732	4654	3154	4700	3624	High
15114	14607	11485	11332	13357	16182	14503	14069	16696	High
10755	14632	12385	13770	12482	14516	14353	19078	15990	High
9283	9094	6704	7727	6715	14165	10395	11897	11238	High
6201	1981	1907	3259	2230	404	255	387	267	High
920	4336	4001	4436	3904	1579	1495	1565	1488	High
2941	18155	17518	18411	18359	1320	1512	1413	1517	High
9397	2556	2147	2654	1963	13529	12905	12608	10193	High
2270	7464	4258	5709	3564	7211	4568	5560	3539	High
9922	8126	4920	7160	7109	25295	16548	19908	20518	High
8982	14269	11482	18712	11395	12277	8514	15385	8431	High
20246	10859	13338	18480	14025	6366	6944	7722	7339	High
6493	10260	8889	10100	8581	11455	10655	11822	9837	High
13849	7870	6176	6266	6949	2441	2440	2144	2226	High
8058	15312	14698	13431	12538	14286	11146	12421	10987	High
392	864	753	726	708	2777	2959	2141	2428	High
10091	13577	13308	17139	10810	10772	9983	11178	8081	High
11731	8623	6921	8511	8041	13012	10389	13352	11612	High
10488	9703	6914	8686	7458	12386	7491	11204	8090	High
2909	14569	10647	12141	9458	11422	9584	9590	7881	High
7639	6289	5903	5070	5144	769	924	1253	820	High
5247	1960	1325	1322	1450	5960	4480	5398	4864	High
4070	2538	2554	2764	2295	2001	1660	1876	1615	High
2935	859	960	670	924	3008	2996	2580	2806	High
10310	11627	10714	12183	11114	14732	11944	13598	12439	High
9249	9828	8604	9577	8977	10186	9464	9561	8991	High
2463	4892	3757	4063	3444	2453	2293	1936	2032	High
6196	19477	8506	19827	8895	16597	8012	19715	8585	High
9049	3317	2527	2865	2402	6951	6968	7060	5794	High
	5884	5184	5474	4690					Not Found
6651	9910	8979	9039	7598	16807	14121	16571	14099	High

16148	9099	8558	8521	9035	10232	7299	8702	8954	High
7550	14969	13063	15662	11939	5300	4906	5861	4008	High
18670	9366	10022	10388	10418	11017	9495	8792	9450	High
6396	22528	18446	22199	20101	9251	7827	8661	7953	High
13052	15700	10812	13779	12406	2824	2294	2373	2434	High
5220	9746	7364	8061	7755	12916	10993	11099	10509	High
1940					8036	7983	8574	7788	High
901					7173	7420	7231	6158	High
5625	8355	6892	8105	7348	3894	2806	3632	2963	High
9004	10524	9530	9833	9093	11021	9108	11000	9875	High
	191	127	222	179	395	270	363	280	Not Found
2861					3629	4178	3959	2831	High
9348	15433	10117	11035	11638	6541	5494	5567	4747	High
6931	4018	3883	4655	3971	3027	3206	3018	2795	High
7478	13550	12133	16343	12596	29227	31059	35110	30178	High
23554	12912	7724	10004	11039	21942	11830	17905	17046	High
4713	4308	3950	3871	3552	2035	1834	1762	1843	High
2135	17054	13748	14568	12891	16473	13883	12602	11636	High
15245	19191	15094	24432	19119	9772	7407	11979	9742	High
11885	8969	5923	8446	6932	10160	6618	9306	7543	High
3160	5557	5488	5953	6076	5587	5092	6354	5819	High
13756	6310	5880	10054	5867	17416	13585	15769	14446	High
									Not Found
1833	10315	11917	10866	9039	10059	12238	10418	9290	High
10622	2386	3624	3263	2653	2311	4341	3850	3143	High
9509	11210	11558	11099	11304	22518	22557	22414	19628	High
									High
10356	21430	20127	22428	20330	9798	10252	10317	9364	High
3341	14222	11929	13023	13543	1433	1494	2056	1774	High
2776	976	783	889	852	12735	8101	9998	10879	High
14675	13428	11779	11796	11475	14901	19014	15201	18095	High
4423	3564	9189	4833	4084	4504	5563	6541	3482	High
12447	6943	6003	5576	6176	7168	5818	5800	6023	High
4524	4620	3539	4905	3796	3651	2670	3391	2812	High
4523	8285	7425	6763	8298	8193	7529	6598	7762	High
15796	12325	8465	11956	11588	15275	11247	15243	14989	High
8933	10575	9014	11060	8890	9699	8916	9605	8277	High
9600	4161	3454	4003	2427	2473	1983	2253	1546	High
3887	6187	5590	5894	4945	15711	16864	14405	13262	High
1477	10227	8488	9359	8408	9872	8600	8723	7849	High
6071	8625	7590	7742	6806	4171	4259	3542	3541	High
2696	25525	7839	7220	11246	24495	8916	8618	11587	High
1771	5175	3230	3769	3791	3055	1681	1911	2202	High
13932	5211	5524	4707	4593	10787	10954	9216	8684	High
5051	14598	10660	11316	12084	16806	12891	13314	14316	High
7971	2688	2572	2279	2291	10505	10505	9940	8915	High
8390	12044	10142	13476	11133	3686	1897	2356	2206	High
5716	4330	4254	4204	3898					High
6497	7222	6406	6118	5721	10233	9562	9021	8550	High
2077	19643	14467	16296	14812	9424	6167	7507	6484	High

22678	15414	14526	15704	13415	2734	2332	3061	2405	High
17321	15563	11897	12344	14990	27261	21549	20539	24949	High
9513	8993	8536	7757	7893	10418	10421	9473	8745	High
12362	6117	4703	4226	4588	12864	12980	13503	12980	High
8521	3322	3136	3262	2675	2499	3204	2834	2143	High
7330	5382	4075	4857	4522	7757	6852	6890	6135	High
3216	8097	7845	8513	6775					High
5137	1785	1930	2142	1844	1371	1287	1412	1190	High
4073	12468	10269	11101	9678	9601	9345	8969	7424	High
9389	9884	10411	10293	10496					High
5113	5733	4602	4892	4261	10006	8663	9136	7554	High
15223	14239	12782	11670	14785	6289	4730	5108	5548	High
7783	10346	9274	8369	7750	9808	8736	7417	7690	High
7386	1873	1365	1715	1057	7582	6509	6963	5623	High
	935	801	579	740	1015	1070	894	916	Not Found
8419	8532	8085	9526	7658	1131	1148	1297	1086	High
7833	4541	4164	4683	4030	10143	9651	9148	8599	High
6612	7918	6489	8260	6769	9137	8014	9901	7489	High
17466					13814	10486	11015	11704	High
314	949	1040	1053	919	1471	1693	1441	1604	High
6859	15603	10764	13463	13230	20120	14186	17452	17263	High
6992	12199	9941	11345	11511	12717	9926	12090	11953	High
12008	4666	4059	4774	4089	14539	9035	9884	10220	High
8798	12439	12582	10366	8613	6152	6754	5772	4606	High
11762	12304	9997	11564	12112	4981	3901	5134	4398	High
11933	7900	9059	7224	8639	11279	13157	10568	11382	High
9752	6734	4710	5702	4711	5640	4940	5936	4648	High
4027	9497	8143	8127	8071	4135	3754	4386	3532	High
9263	7134	6679	7330	6812	7064	6790	6995	6821	High
8417	7669	6859	7324	6872	8149	7758	7676	7451	High
10437	10649	10416	9026	8125	6501	6194	5885	4904	High
8660	5876	5342	5778	5473	1011	1110	1489	1091	High
1377	10316	9679	8853	9493	674	636	653	660	High
1732	1615	1179	1446	1341	8143	6931	6662	6720	High
2151	10678	7759	8544	8271	10693	8614	9788	8601	High
3282	4813	4181	4165	4061	2182	2182	2214	2125	High
2840	8221	7381	7948	6347	4509	4141	4609	3820	High
2112	15859	14452	16406	12779	12611	11350	12411	10390	High
3280	3159	2893	3058	2917	7078	6240	6578	6521	High
5636	9276	9396	9258	7353	8217	8593	8370	6275	High
11823	6460	5933	6253	6017	11950	10646	11904	11175	High
7379	6451	5721	6166	5514	4398	3973	3598	3408	High
9677	7762	5633	5982	5258	3291	3109	2845	2642	High
9681	5291	5266	3865	5662	9919	10278	7183	11370	High
6425	6363	4735	6391	5563	21080	16612	19643	17692	High
8135	9304	9539	10649	8629					High
8091	6747	6174	6779	5970	1848	1288	1395	1531	High
14609	2080	1657	1849	1610	7395	5481	6537	5756	High
2306	1930	1487	1827	1529	5626	4669	4931	5019	High
3971	13574	11052	12420	9097	9162	7708	7966	6284	High

1176									High
5194	3731	3784	3375	3153	9416	8964	10876	7977	High
7248					10531	9984	9283	9365	High
13748	10615	7776	10035	8275	10347	9183	10314	8078	High
7422	11015	12733	10594	11143	6316	5730	5909	5097	High
8819	4377	3853	4151	4271	4155	3520	4463	3481	High
7798	1341	922	1068	1085	5689	4037	4386	4379	High
11858	5631	4781	6644	5487	3812	3000	5707	3842	High
8576	3532	3724	3502	3195	3892	2698	2977	2866	High
5977	6053	5458	8081	4929	3244	2799	4245	2615	High
2555	16234	15274	15415	12217	11596	11279	12202	7924	High
1833	2635	2277	2789	2273	11603	10119	11990	9390	High
11059	7032	6779	6281	6531	13615	11963	12240	12617	High
746	18784	16011	15916	14229	10544	10271	10068	8602	High
12083	4747	3980	4248	3702	12258	12131	14553	11480	High
4452	6215	6327	6420	5522	4033	3427	3293	3537	High
7078	9094	6967	7464	8141	9050	7012	7094	7609	High
8842	9066	5000	7404	5720	1775	1465	1821	1342	High
1634	7609	7862	7190	6742	8053	8604	8137	7638	High
11919	19556	17606	19601	18202	14415	10358	11847	12312	High
6195	11534	8120	9607	8964	8069	5563	7343	6366	High
7910	5332	2993	3540	3605	11554	6611	7664	7819	High
4113					6977	4753	4344	4527	High
9315	12121	12525	11198	11640	8158	8496	7192	7419	High
3119	4112	3340	3562	2952	8758	7274	7979	7042	High
4248	7394	6681	6684	6814	6332	5965	5959	6123	High
9240	8118	6715	7727	6772	11902	10125	10825	9605	High
4503	8934	8796	9123	8318	9200	8029	8100	7832	High
4937	14354	14578	12177	12623	15701	15161	12603	12658	High
5976	1292	1034	1232	1047	7317	6420	6325	6587	High
7295	14992	13044	15225	14225	1707	1144	1427	1088	High
1177	3257	3551	4078	3131	6662	6734	6316	6735	High
7797	10645	11880	15580	12084	5985	5838	8244	7017	High
7007	9359	8345	9936	8441	6813	5057	6771	5472	High
11441	12003	5267	8381	6382	16898	7672	11857	9442	High
12741	13559	12454	12898	13032	7068	6209	6254	6727	High
5207	12818	10944	10317	11554	13012	12150	11724	13187	High
11195	19949	14792	16756	14002	5869	4395	4610	3841	High
	13337	10729	11388	9816	7285	6924	6796	5906	Not Found
3864					4186	3779	5336	3736	High
6565	7651	5883	8014	6220	7855	6231	8859	6915	High
6871	2839	2922	3025	3084	16499	13579	16454	16983	High
9566	5761	5578	4944	5737	3150	3776	2405	2930	High
9418	15232	12141	13763	13058	8802	7075	7990	7259	High
3681	6050	6188	6615	5846	3161	3358	3606	3117	High
9667	10709	9300	9605	8726	8106	5961	7249	6509	High
4293	7039	6312	6091	4936	14776	12016	12471	9832	High
10953	18974	15086	17311	17831	31166	23039	26490	28798	High
8494	15873	12271	16288	12754	1063	1324	1225	997	High
8707	5383	5379	6574	5031	8282	7874	9149	6964	High

9929	7959	6084	7100	6538	1544	1242	1476	1382	High
9876	16319	10891	13224	13020	390	354	412	302	High
1671	1321	1524	1141	1035	6447	7246	6501	6012	High
11723	8372	10654	7153	5521	11454	18625	11272	8789	High
7604	7533	5834	7446	5176	5071	4537	4923	4108	High
10588	10981	12279	11342	9422	17299	18814	18164	15170	High
8346	3210	1741	2297	2717	17075	10839	12163	14022	High
12310					11169	8124	8582	7921	High
4238	7311	5102	6476	6099	18432	15120	18257	16257	High
7669	4767	4506	4618	4751	9232	8697	8260	7310	High
7353	6921	5752	5497	5640	3875	3388	3215	3195	High
10269	4441	3980	4760	4283	5816	5244	5746	5500	High
6012	6099	6098	6627	4334	8739	9583	8245	6403	High
11689	4600	3716	5123	4383	5427	2693	3394	4270	High
5115	10388	8881	10897	9092	12472	10016	12071	10058	High
9389	3813	3463	4183	3269	4013	3372	3046	3158	High
16942	7729	6187	6866	6213	10646	8686	9398	9057	High
10928	25195	19135	22817	20798	14978	9588	12236	10498	High
7287	10966	8962	9350	8064	9143	8008	9387	6969	High
8111	3767	3723	3213	3388	12621	12768	14145	11435	High
33791	3160	3281	2949	2916	3103	3011	2842	2722	High
9067	6101	5856	5862	5334	8030	7677	8022	7163	High
703	5424	5009	4905	4309	5265	4814	4746	4099	High
6030	14871	7950	14241	10740	7604	4587	9300	6463	High
5853	5924	3832	5281	4562	9772	7527	9411	7839	High
2318	2398	3272	3018	2961	561	448	512	504	High
6686	11850	8531	10229	9110	5040	3683	4112	3815	High
7675	4624	3510	4958	3734	2117	1748	2036	1638	High
4886	8369	6791	8282	6921	9493	7424	9151	7714	High
6588	12363	9890	10533	9202	12482	10206	10521	10203	High
889	3742	3611	3792	3257	4275	3584	3829	3736	High
1672	3040	1880	3014	2206	9051	6809	8538	7013	High
5706					14981	15315	15102	13024	High
2055	3362	3353	2914	3559	4164	4144	3461	4114	High
	1899	2030	1977	1703	3715	3658	3818	2981	Not Found
10671	12017	6815	9620	8917	14464	7891	11302	10437	High
5468	5088	3910	3875	3826	756	947	916	799	High
1168	12795	11938	12147	13310	3090	2985	3521	2911	High
3070	2437	2555	2459	2166	8363	6829	6834	6243	High
4574	12327	9289	10626	10090	8235	6540	7484	5545	High
13682	2698	1845	2170	1687	14146	11329	12311	9553	High
176	6644	4997	7630	5969	13473	11522	12846	11560	High
18117	14986	15549	13316	17847	6669	6633	6871	6828	High
6808	8717	8715	7748	7185	3587	3376	3688	3277	High
8621	2169	1865	1903	1953	11446	10261	9412	9113	High
6577	6773	5664	8151	5745	12287	9244	11860	10322	High
3442	4427	4397	4922	4317	2645	2402	3225	2431	High
414	980	1109	1077	1087	893	1004	957	934	High
17109	6945	4827	6793	5530	8830	6182	9129	7778	High
1857	2977	3422	3861	3185	7717	5417	5687	5930	High

3375	2716	3194	2594	2156	7144	6457	5405	4420	High
7095	6586	4849	7001	5605	7469	6276	9254	7037	High
16755	993	809	986	702	12916	10176	13624	11350	High
3772	4285	4233	4336	3954	3175	2952	2999	2802	High
2912	3619	3390	3888	3071	6739	5611	7343	5374	High
7938	5445	5029	5103	4831	13464	11253	13011	11275	High
3052	9118	7508	7367	6724	6429	5324	4792	4607	High
768	6469	5613	6944	5742	9041	9142	10117	8118	High
2231	11100	10824	10034	9166	15824	17918	16126	14860	High
7470	3160	3273	3080	3018	3887	3038	3506	2970	High
3589	3749	4181	3378	3154	2465	2465	2087	2033	High
1849	18291	18642	18816	15761	1736	1501	1981	1610	High
3249	9229	8636	10112	8237	5011	5212	4551	3828	High
7088	6152	5983	5080	5826	9247	9117	7461	8900	High
6352	771	574	632	440	2523	2158	2591	1878	High
9691	11331	10773	9079	9533	11145	10874	9190	9737	High
6573	4854	3571	3816	4085	10539	9206	8615	8786	High
4815	12182	9017	9998	11243	6646	5612	5320	7578	High
5039	11889	9030	11138	7801	2888	2311	2578	1985	High
3160					7760	7757	7658	7709	High
7340	3578	3194	3283	3209	9943	8104	8662	9886	High
6429	8409	6317	8042	6749	6703	3280	6282	4189	High
6913	7695	6616	6422	6074	2277	1318	1339	1621	High
7289	4467	3356	3632	4161	10913	7107	10620	9862	High
6067	6989	6610	6562	6521	3019	3257	3107	3029	High
					6156	5378	6229	5937	Not Found
3095	7005	7449	8383	5254	5999	5472	5453	3972	High
5098	8802	4718	8447	8340	11209	5693	9558	10257	High
11968	3148	4367	5659	4752	9180	10668	15283	12886	High
10765	5873	5796	5035	4680	8445	7060	7062	6598	High
5040	12512	9677	11681	12157	7352	4332	6376	6581	High
2940	6445	6550	7726	7145	11554	10720	10813	9770	High
10183	5415	5824	5067	6150	3888	3840	3639	4328	High
3299	448	319	455	395	6133	5312	6411	4826	High
8736	2381	2047	1977	1850	12308	9589	10714	8727	High
6671	14741	13236	15053	14082	9045	8360	9742	9515	High
6275	1742	1668	2120	1608	9608	9623	10619	8576	High
6503	5559	5900	6242	5258	12643	11590	13726	11567	High
9725	13318	12949	11684	13767	9000	8355	8806	9938	High
13354	10470	6110	7432	5907	10768	9225	9518	8491	High
9385	1004	1162	954	773	11910	11120	10504	10108	High
7003	8516	8445	7990	8135	15849	13844	14771	13454	High
1124	4555	4489	3907	3449	4598	4775	4180	3646	High
3084	3549	3936	3828	3390	5689	6081	4326	4577	High
1471	3415	3608	3926	2905	17287	13958	15652	12991	High
4380	3296	2659	3105	3241	7747	6337	7392	7029	High
10181	8062	8667	7301	8171	4097	3148	3731	3426	High
5086	23451	16328	18567	20860	4874	3545	5123	4677	High
3417	10867	9835	9976	9372	1128	1049	961	919	High
12255	6844	5126	8099	6650	17509	16018	21723	18085	High

10961					6226	5877	5621	4689	High
6737	3965	3031	3549	3404	4839	4726	4247	4174	High
4957	9878	8685	8071	8027	10791	8766	7854	8391	High
5951	1853	1842	1813	1556	4467	4436	4062	3601	High
2929	1042	920	920	1044	732	815	697	663	High
1442	3039	2167	2600	2406	7296	7796	6510	6390	High
1089	11934	10407	10728	9933	1714	1424	1255	1202	High
8443	6311	7541	6575	6979	4727	6000	4186	5545	High
5292	5379	4836	5190	4755	13876	11544	10995	11035	High
1640	5095	4697	4668	4718	6844	6387	6687	6496	High
12769	2160	2293	2511	1886	6827	7614	6629	5943	High
6852	5121	4405	4719	4374	8454	6864	7754	6825	High
1961	18667	17186	16701	14481	20784	19442	20276	16978	High
9871	3658	5243	4031	3938					High
10807	17032	15399	15841	14137	8126	7377	7959	6775	High
4197	5093	3993	4692	4232	5897	4498	5620	4022	High
19288	9372	8413	8214	8434	7437	5485	8473	6963	High
7198	6241	6095	6408	5038	1530	1473	1591	1370	High
2179	1277	1213	1286	1071	12859	11250	11248	10836	High
7113	6920	5883	4217	6766	7475	6256	4474	6940	High
7551	6632	4828	4711	5994	4916	3670	3831	4224	High
	4974	3202	4272	3695	4867	3975	4185	3891	Not Found
5199	19041	13413	11053	16651	6273	6166	6392	6688	High
9058	6109	5818	5514	5694	4096	3362	3651	3541	High
4166	7716	6285	6295	6542	1598	1860	1530	1522	High
									Not Found
5667	6338	4477	5526	4956	9247	7015	8098	7727	High
16697	6025	5448	6069	5444	12604	11205	13124	12234	High
1846	1508	1142	1310	1220	2807	2357	2937	2141	High
9400	8983	6642	7813	7610	12472	7153	9800	9301	High
7665	6641	6776	5952	6171	8982	8072	6706	7515	High
6456	11329	9836	9354	8532	13980	13579	11864	10676	High
3503	4305	1909	2904	3525	10727	8116	10306	9311	High
3347	10746	8959	8274	9113	3639	2700	2071	2874	High
11238					3344	3285	3422	3031	High
2870	18860	14563	17108	15369	8009	6262	7693	6816	High
3818	8649	8011	7713	6705	14680	14745	13663	11639	High
7934	10208	7406	9038	8600	9432	6257	7994	7485	High
9978	8196	5915	6304	5849	16913	13490	13526	13352	High
8646	5124	4750	3897	3905	9127	8912	7185	7218	High
6421	3967	3169	2856	2889	7475	6658	5159	5373	High
	10794	9344	9868	9822	1744	1898	1843	1759	Not Found
1771	9802	6751	8219	7460	12396	9058	11362	9523	High
9046	13786	10734	10701	10313	10330	8443	7513	6584	High
832	3926	4346	5190	4563	9499	7593	8598	8012	High
7443	9947	7965	9441	8859	5887	4745	6077	5490	High
8507	7960	5358	6790	6449	4403	3732	4367	3679	High
7930	15285	17010	16937	16015	9804	11148	11130	11217	High
3346	4919	4760	3866	4361	1676	2003	1738	1574	High
4307	14725	11893	11213	12159	7327	7505	6805	6666	High

11564	8642	6012	7017	5853	12811	8353	10003	8442	High
4964	5539	4853	5497	4204	3011	2740	3096	2204	High
1628	3202	3658	2772	2548	489	471	470	398	High
7818	4938	3796	4131	4063	10012	8067	9135	8768	High
6422	3418	2267	3562	3230	7217	4582	8032	7159	High
6146	9631	9510	8303	7231	1763	1256	1308	1134	High
20071	12295	10721	12862	12338	5651	4728	5786	5324	High
1733	5780	4630	4429	4248	9744	7911	7397	6864	High
6009	8450	9080	8680	7962	1282	1160	1424	1274	High
5710	3740	4254	2482	3398	3097	2845	2474	2722	High
5562	10796	9431	9607	9005	2584	3299	3010	2653	High
8851	5306	3993	4894	3812	3649	2787	4151	3475	High
6684	6654	5356	6789	5676	117	82	98	60	High
4617	6738	5067	6114	5788	14136	10727	12234	11905	High
5084	12418	7686	9278	9735					High
	4616	5761	4570	3944	5135	6277	4782	4153	Not Found
3962	3195	2792	2848	2709	363	356	394	338	High
3485	10000	9271	9765	8448	5816	6121	6303	5197	High
2947	5183	4790	4812	4360	5029	4826	4448	4262	High
1016	5453	4958	4886	4216	1174	1070	1291	944	High
3146	5133	3205	4413	2940					High
6658	16860	13042	17417	15320	3591	2789	3362	3176	High
6709					11501	8800	9561	8769	High
16180	17250	13721	12718	15043	9708	5974	4830	7875	High
5102	3412	2888	2670	2637	14320	15280	12998	13060	High
5118	10263	9588	9605	7956	2728	2686	2485	2134	High
6483	4448	3674	4773	3995	515	427	620	495	High
3393	14847	12717	14222	12131	8130	8252	8330	7423	High
4933	2413	2015	1620	1512	6500	5608	4685	4413	High
5451	10868	10130	9865	7773	6107	5485	5434	4398	High
8125	14342	12897	12345	12564	13455	11730	12155	11649	High
5844	13048	10534	11722	8756	5932	5120	5612	4163	High
7476	293	187	312	289	8414	5203	7684	6229	High
4556	11254	11191	9849	9151	3642	3798	3289	3081	High
1604	11799	10321	11521	9177	12361	10791	11955	9216	High
4237	2755	2669	3145	2474	2520	2069	2436	2058	High
637	7782	6811	7996	4921	7417	6653	7147	4929	High
9290	6175	4775	5161	5187	3044	2451	2359	2469	High
8493	3786	3512	4183	2944	4218	3927	4167	3146	High
8545	2415	2685	1893	3114	7764	8541	7207	9439	High
4398	6772	5688	6576	5737	12611	14523	12600	11797	High
3636	8805	9503	9173	7237	1249	1138	1148	957	High
3462	12337	10061	10911	10173	7870	6048	7228	6202	High
4840	9714	7613	7109	7092	4573	3686	3404	3586	High
330					3646	5353	4952	4833	High
7316	6453	6616	4920	5570	2212	1670	1954	1721	High
7629	7837	6480	7100	5860	1733	1338	1656	1331	High
4626	9643	6287	8663	7036	15871	10580	14308	11002	High
5079	5302	3599	4295	3509	1361	1264	1235	1160	High
3103					4241	3844	3797	3322	High

7866	5173	3469	4373	4428	7154	5145	6422	6546	High
3919	4338	3989	3920	3601	3690	3332	3558	2412	High
2323	6270	5455	5332	4762	9118	8036	7869	6846	High
3584	3675	2242	2381	2422	4508	3890	4228	3626	High
5268	7243	6574	6945	6850	7272	4572	5709	5849	High
3315	4244	4068	3725	3409	7096	6856	6710	5703	High
	1733	1226	1513	1371	12300	7398	10180	10469	Not Found
6143	1740	1902	1629	1569	2334	2538	2101	1975	High
7583					4188	3829	4165	3692	High
10423	7158	6329	4980	6001	6694	5407	5580	5088	High
1833	2588	2223	2313	1968	664	723	784	640	High
3779	12113	9662	10033	10081	9986	8401	10040	8345	High
6387	10189	9903	8597	10161	14377	14313	12731	14635	High
3609	2374	2315	2433	2380	5417	4766	4948	4587	High
10313	8418	6071	6528	8898	8487	6177	6985	7353	High
4098	5669	4660	5085	4397	2518	2344	2614	2320	High
4901	7031	7291	6724	6362	7014	5987	6291	5818	High
2534	7186	3130	9605	2895	3109	2184	3269	1716	High
3403	4142	3650	3682	3253	16963	13795	14957	12752	High
3422	4511	3831	4082	3565	9658	7141	9930	7959	High
12614	6362	7377	7352	7112	3540	4005	3956	4328	High
13431	9107	9590	12569	8784	5463	5777	5444	4894	High
1144	6525	6615	5395	4910	6270	6035	5535	4937	High
6064	8463	7444	7044	5840	10387	8685	8488	6931	High
856	572	562	544	466	1104	1130	1168	1190	High
4838	8815	6482	7183	8113	10304	10058	9892	9893	High
4598	8208	4716	8074	5517	21392	16369	20129	15188	High
2851	8070	7407	7513	6485	3984	3572	3398	2851	High
1274					467	493	341	419	High
3639	4198	3945	4571	3081	8979	7822	8615	6479	High
1725	3467	2578	3592	2917	3424	2935	3271	3244	High
9212	13176	11686	12226	11873	5582	5304	6132	5108	High
3811	5559	5577	4597	3732	3370	3277	2817	2672	High
6047	5968	5218	5338	4514	2534	2424	2516	2074	High
937	6185	5158	4911	4728	529	382	480	411	High
2474					2583	2807	2763	2863	High
4886	6112	5054	6318	5204	2067	2070	1797	1978	High
6728	6342	5297	5196	3859	16139	19279	13617	11945	High
4888	7462	5695	5537	7057	3743	1704	2855	3880	High
8687	7224	6019	5745	6626	11139	8872	8865	9765	High
4207	14478	13381	11819	11376	8218	7635	6299	6516	High
22855	9148	9384	8514	9340	3795	2633	3110	3123	High
778	3039	3042	2891	2591	5009	4390	4613	3997	High
2221	15953	15410	17176	16215	1495	1034	1501	1436	High
8665	9872	8016	9159	8651	7212	5799	5781	5846	High
3552	2611	3108	3713	3346	4355	4297	5621	4938	High
8399	7410	5698	6468	6739	8233	6471	8140	7838	High
3530	10251	9485	8591	8412					High
1586	6281	5727	5570	4767	3312	2869	2813	2778	High
4710	5846	5664	4670	5019	4309	5279	3771	3943	High

5931	7672	7284	7048	6723	8388	7857	8457	7150	High
4032	6294	5225	4854	4966	8341	7884	7082	6737	High
4081					3688	2838	2481	2810	High
3351	170	181	150	127	3829	3423	3449	3342	High
8635	6877	5083	5469	4988	5383	3797	3933	3943	High
5416	13169	11783	10716	11492	5219	4445	4726	4725	High
7225	2302	2037	2010	2133	6279	4609	5785	4780	High
8342	15239	12618	12456	11623	12989	12458	10174	10819	High
2030	3126	2248	2301	2191	6465	5656	5212	4940	High
8862	17208	11860	13495	13397	12328	7973	9201	9221	High
2134	12036	9445	10693	10614	11851	10005	9652	10185	High
6549	11075	10596	10264	9984					High
	9008	8315	8624	7569	5759	5369	5700	4633	Not Found
6365	3131	1821	1809	2341	4878	3261	3460	3679	High
17655	16639	12710	16670	14383	12590	9443	11952	10266	High
					3742	3635	4137	4186	Not Found
2500					2756	2738	2863	2192	High
4018	6706	6395	5875	6541	8375	7291	7744	8181	High
1199	2966	3293	2671	2411	4383	4012	3656	3415	High
2023	2908	3091	3118	3003	6497	6165	6143	6104	High
2997	2898	2568	2564	2137	10152	8758	8597	8329	High
	7768	6610	6753	6418	10157	8275	8525	8549	High
9339	10707	7987	10596	11580	5845	3845	5585	6134	High
5555	5088	3893	4202	4087	3143	2403	2764	2581	High
3667	9785	9427	9786	10345	12676	11995	12163	12391	High
449	456	499	378	393	421	473	336	388	High
1793	2332	1780	2319	1709	5697	4339	5560	3995	High
5822					5712	5691	5369	5746	High
7192	3607	2771	3314	2764	6478	4877	7150	5098	High
2445	5298	4909	5510	4675	9804	7565	7910	7955	High
9609	15005	14248	14012	13192	4377	4158	4381	3578	High
7735	7077	7084	6156	6286	5326	5758	3871	4461	High
2197	12980	9822	11823	11433					High
3421	2835	2815	3638	2442	664	459	950	489	High
9799	5784	5776	5267	4919	2611	2832	2148	2154	High
10688					3801	3481	4332	3407	High
10305	6492	7061	5468	6903	3451	2992	4714	3266	High
432	589	608	552	428	2577	3193	2605	2281	High
4939					9167	8630	9231	7316	High
4599					615	499	684	561	High
7111	6072	3706	5671	4226	2772	1959	2517	2064	High
4249	3401	3068	3873	3078	5312	4026	4701	4364	High
6104	4134	4337	3661	3504	12147	11389	11326	8821	High
1869	8986	6589	9846	7677	6931	4604	6532	5625	High
3937	15169	13761	13835	13052	9974	10026	10276	8974	High
6176	8496	6920	7770	6775	5236	3557	4594	4018	High
12615	3738	2903	3460	4733	2159	2812	2271	2200	High
	5263	5735	5302	4382	3879	3653	3695	3395	Not Found
6690	3801	3321	3537	3236	7035	5753	5433	5728	High
7742	7253	5958	7327	5466	3406	2405	3297	2160	High

2156	7963	6393	6652	6231	6373	4955	5706	4839	High
1124	3534	4514	3266	2478	1120	1263	1146	971	High
792	941	864	958	1114	2680	2355	2475	2628	High
7219	6389	5356	5175	6247	12781	10673	11442	11704	High
5000	5105	6940	4374	7931	10680	11167	9183	11487	High
1894	4850	4586	4675	3483	5373	6657	5435	3898	High
4788	12174	10479	13214	11692					High
6608	9204	9069	8459	9214	8270	8312	8007	9002	High
8630	3494	2944	3480	3365	7074	7709	7782	7585	High
3796	7725	6136	6489	6394	6558	5187	5750	5292	High
3701					1356	1263	1265	1037	High
	3427	3696	3762	3900	3400	3131	3279	3997	Not Found
191	598	578	660	524					High
8061	6576	6977	6908	5688	5961	3413	4447	3742	High
590	2798	2233	2776	2173	4880	3803	4286	3581	High
3568	4381	3994	3952	3980	2541	2299	1939	1986	High
1521	1193	1186	1230	1066	2711	3029	2930	2518	High
10273					8080	7745	8510	7348	High
1547	9820	7963	8480	8929	4715	3180	3583	3382	High
2174	367	280	267	316	2292	2866	2783	2162	High
3849	4474	3737	4190	3941	3468	2699	3273	2945	High
3332	4129	5498	3360	3799					High
3932	3953	3331	2668	3073	10248	8853	6501	8156	High
7106	2833	2975	3480	3320	6790	6659	7308	6388	High
8174	8171	7446	7623	6344	9285	6192	7833	6585	High
2700	2566	2006	2038	1977	13837	10496	11154	10605	High
4584	4975	5368	7001	5774	6627	7744	9880	7985	High
2003	4681	4050	4227	3388					High
8372	13155	6617	9088	7689	5328	2451	3489	2913	High
7843	7451	7654	5737	5946	7984	6498	6441	5708	High
1612	2232	1370	1925	1674	13682	8863	11929	10285	High
6790	9594	8066	9910	7208	5556	4285	6322	4060	High
6386	8111	7702	7284	5156	7452	6219	6827	4581	High
4035	23940	23211	20582	15300	5378	5143	5122	4121	High
4680	9939	8377	8051	6755					High
3995	7808	5469	6572	5664	7171	5202	5860	5389	High
	5879	6372	6416	7788	5811	5779	6403	6961	Not Found
9695	4155	4051	4007	3658					High
10327	11857	8660	10869	9620	5148	2869	4375	3838	High
3004	10558	6208	8346	8804	9185	6580	8117	8140	High
11656	4497	4015	3875	3227	4632	3462	4603	3563	High
7892	8416	6772	7169	8240	7771	6258	6379	7268	High
5356	8235	7720	9079	6043	2777	2553	2887	2115	High
1370	1560	1754	1425	1204	821	1153	845	744	High
2802	5813	4790	5672	4412	4400	2900	4185	3003	High
2016	4524	4590	5091	4152					High
9121	13402	11066	12714	10418	5613	5004	5802	4469	High
735	5652	3305	5650	4277	4760	3376	4712	3962	High
7065	6087	5373	6201	6685	2559	2399	2391	3213	High
4161	4260	3670	4017	3701	6404	5030	5230	4917	High

2164									High
20052	8033	4457	6069	7396	5758	5103	5221	5057	High
1348	1402	1304	1248	1086	2288	2314	2188	2426	High
2420	2268	2089	1617	1370	2855	2514	2068	1921	High
325	299	270	266	248	244	162	141	157	High
	15583	13988	15394	13230					Not Found
	2159	2099	1761	1690	2457	2411	2330	2228	Not Found
3143	5285	4728	5668	4366	2667	2359	2296	2003	High
2154	9675	10308	9095	9007	6717	5337	5658	5520	High
15005	3679	2681	3519	3161					High
2146	3263	3568	3038	2993	2878	3247	2564	2630	High
2728	10345	8999	7728	8881	9892	8570	6918	8665	High
1513	10373	11250	11263	11283	5542	5650	5446	4823	High
3675	8817	6597	7268	7361	4283	3688	3496	3733	High
6613	7122	6883	9566	5973	6474	6764	8049	5805	High
	3107	2173	3041	2533	983	912	1322	913	Not Found
2934	2807	2772	3328	2269	2221	2054	2483	1713	High
7892	3871	2975	3423	3138	5985	4696	5919	4822	High
7827	9728	7706	8867	7419					High
6416	6962	5624	7155	5437	8723	6728	7809	6537	High
699	3060	3599	3079	2736	5261	3908	4162	3844	High
4404	7931	6365	7554	6488	8686	6087	7219	6248	High
573	6975	5859	7034	5646	1236	1101	1227	1028	High
5902	2189	1420	1902	1981	19463	16511	19265	16055	High
5613	11608	9802	9538	9895	2838	1940	2342	2235	High
	3457	3848	3573	3167					Not Found
	315	327	284	297	732	739	762	635	High
6848	4741	4198	4627	4615	5891	5298	7193	5503	High
2556	4420	3516	4270	3597	14028	7888	9480	8899	High
	3765	4045	3612	3118	3033	3206	2857	2703	Not Found
2146	6889	5556	7033	5995	11700	9316	10707	9595	High
6201	13688	10595	12246	11575	5254	3586	4707	4178	High
6284	3568	2914	3275	3568	7822	6736	6686	6670	High
9752	6854	5384	6017	4608	7868	6102	6767	5323	High
15494	3801	2465	3975	2899	12280	11428	17993	11435	High
9328	2169	1894	2180	1857	3427	2592	2578	2457	High
5182	5101	5493	5287	4578					High
									High
2179	578	401	592	575	7200	6103	8025	6821	High
5309	457	462	473	464	12876	12196	13258	11624	High
2078	3334	3139	2432	3708	7464	7116	5754	7961	High
	7565	7137	7870	6844	1148	1486	1987	1223	Not Found
19591	14949	12741	13550	13927					High
4605					4670	4357	6603	3657	High
	5650	4619	4895	4743					Not Found
3219	5548	5079	4002	4756	6369	5745	4625	5572	High
6040	1798	1950	2058	1734	9574	8629	8690	7414	High
2927	11131	9294	9303	9287	7932	5847	6483	6008	High
2016					6351	5600	4222	5336	High
4080	10600	9139	10438	8596	2280	2223	2324	2074	High

1987	1701	2540	1878	1705	3509	3345	2918	2758	High
1976	3409	2734	2753	2387	1727	1590	1668	1278	High
2609	10039	9753	10955	9159	2541	2102	2026	2149	High
7703	2593	2905	2614	2700	5066	5066	3959	4270	High
1808	5245	4491	4600	4225	997	947	1359	945	High
3315									High
2537	3334	2335	2738	2659	6739	5397	6303	5919	High
	3052	2452	2746	2513	6939	6763	6613	5827	Not Found
	8643	7251	7004	6407	1304	1222	1213	1044	Not Found
5269	1271	1146	1488	1025					High
7437	4466	4939	4018	3776	5927	6039	4959	5496	High
9971	8000	6515	7048	7465	6890	6171	6425	7114	High
9582	6824	6082	5550	6752					High
2414					6538	7041	6889	6439	High
2372	1265	1479	1301	1112	10410	8208	9993	8181	High
	2421	2243	2045	1921	7853	6901	6295	5958	Not Found
15994	7034	6083	6542	5716					High
4537	10367	9066	8736	8015	3623	2901	3463	2966	High
2660	2473	2681	2160	2611	2612	3058	2213	2690	High
3294	3183	2495	3425	2640	1970	2205	1998	1655	High
5206					2654	2395	2437	2119	High
5863	6745	5652	6290	5471	4598	3279	4553	3551	High
10120	7873	6017	6099	6918	2793	3871	2619	2561	High
5620	4182	3831	3744	3376	6022	4213	3955	3673	High
3762	4856	4848	4544	4142	2464	2479	2543	1982	High
8847	9610	11258	12198	10695	2036	3134	3062	2810	High
2230	6484	2133	6207	2277	12006	3930	10082	4407	High
3550	5269	6158	5150	4782	7916	9627	8111	6685	High
248	78	79	79	98	234	208	277	268	High
3144	9546	8215	9531	7748	3674	2804	3277	2702	High
4817	8408	7146	7030	7328	1412	1251	1375	1201	High
8591	3641	3243	3888	3348	3290	3256	2040	2356	High
4325	4499	4307	3675	3652	1054	895	1023	896	High
2534	6843	5870	6513	6936	2916	2004	2405	2247	High
1828	1474	1139	1415	1025	1330	1014	1255	857	High
	9227	8990	8318	8100	7233	7324	6558	6598	Not Found
	2275	1825	2455	1760	11445	10874	11396	9729	Not Found
5332	2370	1812	2009	1592					High
6262	3717	3751	2526	3433	3564	4315	3071	3843	High
6353	5364	4079	4833	4103	2018	1863	1838	1729	High
	22090	16043	21123	17038	1428	842	1364	1039	Not Found
5237	3920	2891	3179	3079					High
1224					10582	9939	10146	8683	High
5928	4101	3791	4544	3204	4454	3529	4459	3345	High
559	1013	1120	935	742	1236	1182	1102	965	High
6314	6288	4429	5197	5370	4508	2661	2509	2190	High
1141	3250	2918	2863	2922	6675	6223	5506	6637	High
3499	4759	4611	5384	5675	15150	11728	13156	13732	High
8337	11644	9475	9122	7678	3083	2651	3219	2239	High
	9441	6245	8591	7409	9207	5840	8648	7065	Not Found

11534	12164	8835	9139	10551					High
3502	1689	1475	3667	1537	3825	3900	5315	3065	High
7870					13590	8157	8140	6218	High
2102	2567	3485	2677	2210	2627	3869	3125	2259	High
7548	4944	4205	4463	3426	6680	6084	6977	5152	High
1157	3694	3555	3379	3090	2924	2965	2513	2684	High
72	1141	769	856	809	6718	6693	5289	5345	High
3958	4475	4082	3547	3019	2344	2738	2088	2023	High
9229	5293	4299	4632	4002	4211	3430	3474	3325	High
1025	2891	2340	2345	2466	2949	2600	2547	2428	High
12415					1166	1115	1105	1047	High
3218	6839	4320	4861	5129	5662	3212	4308	4079	High
4785	8057	6635	7050	6583	4403	2552	3253	3120	High
3376	5650	4921	5234	4929	3758	3575	4004	3811	High
3092	2656	2322	2414	2068	11797	10974	9867	9420	High
9255	2710	1521	1699	1667	18563	9863	11193	10985	High
2648	7117	7221	7060	7045	3718	4025	3184	3500	High
5434	4533	3634	7623	4018	2874	1249	2244	1891	High
8727					1869	2010	2564	1955	High
10535	3415	2712	3191	2659	6317	6120	5170	5375	High
7522	2918	2424	2594	2331	4345	3877	4139	4004	High
2382	2579	2363	2133	1913	2910	2611	2164	2633	High
7968	13448	12513	13757	13017					High
2106	6600	4663	4366	4811	12653	10095	9601	9805	High
7117	1241	995	1280	903					High
2290	3317	3131	3259	2909	8427	7519	7912	7070	High
7224	9287	9352	9873	8212					High
	4135	3510	3314	3593	5051	4499	4068	4460	Not Found
1259	3179	2986	3148	2772					High
9098	3622	2874	3078	2696	3567	3567	3247	3292	High
2313	1246	1614	1846	1469	1750	1927	2463	1956	High
790	6143	4976	5545	5858	5374	4373	4663	4770	High
2865	5362	5512	4759	5073	3741	4085	3255	3614	High
2177	1261	1396	1270	1239	2734	3058	2697	2732	High
7817	11257	7597	9450	9245	5739	3771	5134	4897	High
11177	11849	10184	10646	10400					High
4059	4760	3739	4901	4246					High
3494	3795	3253	3207	3066	10655	9113	9778	9319	High
					3844	4442	4349	4063	Not Found
2648	9544	5721	8472	7275	8909	5615	7281	7337	High
9037	4378	4404	3972	4258					High
7508	9457	7679	5573	6728					High
8695	2967	2801	2970	2775	3289	2975	3121	2643	High
3234	11863	6485	9021	6404	11030	4311	7737	4800	High
	6449	6258	6962	5689	5140	4988	5872	4664	Not Found
989	1194	1165	1085	994	1261	1300	1164	1023	High
5218	3578	3147	3387	3374	1830	1502	1871	1851	High
7407	1401	1219	1399	1287	13363	10987	14196	13804	High
3816	9091	8479	8171	9289	4470	3985	4290	4335	High
5618	3121	2130	2958	2500	10158	8575	9049	8834	High

1321	2693	3001	2973	2529	2039	2098	2332	1893	High
1005	4509	2781	3545	2910	2889	1285	2058	1660	High
1254	4794	3872	3995	3338	5529	5908	5485	4862	High
1640	2908	1818	2273	1981	1692	1128	1426	1269	High
6799	8367	5613	7166	6769	3580	2381	2805	3234	High
4124	5285	4529	4577	4605	1856	1482	1558	1490	High
6420	7314	8003	8588	6730	5728	5805	7027	5178	High
3249	2554	2716	2298	1990					High
6156									High
1679	1419	939	1077	918	3116	2223	2529	2063	High
1866					4244	2972	3051	3129	High
1885	3413	3606	3620	2817	5760	5033	4967	4103	High
2009					6361	4473	4063	4418	High
3138	2477	2464	2514	2630	4248	3856	3467	3519	High
	3175	3293	3257	3006					Not Found
									High
3520	11548	8996	11759	10263	4219	3156	3851	3851	High
1668									High
2181	4103	3795	4288	3603					High
344	4593	3281	4316	2612	4597	3873	4407	3395	High
2089	4655	4841	5831	4642					High
	5440	4281	4458	3927	6567	5363	6176	5311	Not Found
4215	4733	4201	4405	3195	5429	5090	5313	4204	High
4379	8838	7088	9893	9712					High
5797	8529	7511	8223	5961	2165	1896	2104	1534	High
11952					10280	7757	9286	8248	High
2767	6100	5122	5670	4922	2911	2711	2867	2350	High
7987					11175	9004	8259	8425	High
2138	3149	2076	2531	2306					High
2053	1790	1586	1822	1687	6624	4591	4768	5381	High
18431					8873	6975	7503	6771	High
397					292	264	203	228	High
	12703	9116	10593	10688	14081	8929	11686	11209	Not Found
9686	12459	9722	10774	12684					High
2332	4953	4860	4673	3582	5367	5475	5287	4100	High
2089	4430	2549	2259	2813	3385	2284	2876	2593	High
6297					3192	2230	2537	2645	High
2060	2358	1745	1710	1568	6941	4343	5502	4521	High
	9036	11004	7456	7560	9125	10557	7659	8089	Not Found
7529	2184	1970	1740	2424					High
	7778	4994	6988	5195	8939	5705	9333	6130	Not Found
5384	8772	7768	7885	7479	4816	4190	4417	4502	High
3160					6189	5070	5797	4905	High
6320	10404	9239	11214	8726	7418	6412	8354	6276	High
	9811	8854	8602	7379	1334	1049	1258	974	Not Found
5970					7571	6498	7667	6519	High
	6495	7362	6784	7575	3073	3942	3164	3473	Not Found
3918	1847	1532	2183	1867	4869	3932	4693	4453	High
530	2164	2365	1914	1496	3327	3102	3044	2467	High
4692					3297	2490	2308	2465	High

3024	2161	1903	1810	1896	11515	9499	10615	9785	High
1577	3264	3391	3039	2963	1706	1845	1843	1662	High
	11337	10181	9201	9889	5245	5363	4006	4278	Not Found
5843	12277	9202	10331	8980	5515	4745	4971	4226	High
7227	4481	3407	3686	3734	7688	7929	7787	9353	High
3494	3958	3893	3188	3070	6817	7000	5364	5136	High
	6944	5359	5781	5334	7091	5053	5642	5208	Not Found
1580					1893	2007	1610	1371	High
	2612	2500	2353	2479	1910	1563	1760	1501	Not Found
576	315	392	386	323	346	444	446	347	High
6726	10484	5381	8045	8807	4825	3396	3836	5575	High
2429	10052	7475	6626	8207					High
1854					4194	3993	3619	3793	High
1968	548	528	495	474					High
7805									High
3476	840	786	712	665	7492	7682	8367	6242	High
	15422	13808	15099	12679	577	470	451	429	Not Found
1255	3349	2816	2818	3006	4082	3899	3323	3516	High
9694	6430	5842	5635	5635	4192	2974	4044	2946	High
10040	5902	5312	5105	5304	7436	6028	6745	7439	High
1560	3384	2998	3231	2526					High
2308	3666	2224	3557	2726	6156	5176	6033	5164	High
3641	4410	4121	3891	3540	4990	4551	4444	3724	High
2809	8913	9367	8334	6540	4179	5050	3736	3387	High
8157	2669	2289	2065	2041	5861	5517	5451	4694	High
3540	751	1535	1868	1665	4440	2775	4733	3367	High
2862					1369	1267	1249	1284	High
2263					6230	5785	5522	5418	High
	2402	2387	2274	2051	1816	1828	1727	1569	Not Found
2967	1040	1124	879	757	6445	4809	4871	3955	High
6149	1079	808	846	929	6819	6108	6112	6430	High
3253					5722	4645	4953	4204	High
5751	4496	3525	4142	3248	2814	2208	2617	2112	High
1988	8916	7164	8525	5647	4357	3082	3926	2670	High
	2105	2399	2182	2127	6544	6604	6735	5647	Not Found
15985	8550	7859	9399	8996	4501	3993	4905	3497	High
5898	5514	4114	4920	4609	11606	9922	9935	10073	High
	5029	3701	5055	4645	2730	2061	2073	2444	Not Found
980	3353	2992	3237	2931	4217	4058	3982	3986	High
2214	4266	4000	4084	3481	3468	2866	2895	2493	High
2626	3287	2371	3024	2402	5875	5507	5845	5170	High
	286	265	231	245	401	341	364	369	High
1560	2429	1930	2777	1565	1640	1462	1880	1201	High
7277	5297	2697	4438	3570	5062	2190	4364	3236	High
2204	1058	813	1122	881	2344	2115	2301	2401	High
2743	3515	3760	4831	4024	1760	1877	1943	2218	High
6977	3726	3446	4396	4705	5199	5293	5988	6146	High
3378					6083	4773	5286	5235	High
1372	3854	3129	3081	2651	1283	1346	1101	1336	High
6189	948	508	731	640	2635	2520	2953	2460	High

					650	760	618	547	Not Found
7492	2164	1787	1948	1673	2492	2072	2004	1880	High
3053					5387	5690	5592	5365	High
8294	2105	1900	2003	2014	2616	2225	2297	2481	High
4232	8850	9465	8610	8122	5111	6011	5018	4630	High
	4796	2381	4244	2933	1152	977	900	867	Not Found
2332	4511	4373	4214	4050					High
5199	6121	4430	6029	5723	7860	5105	7895	7252	High
6933	6720	5638	5546	6434	3605	2850	2593	3126	High
2361					5387	3955	4464	4272	High
2399	3080	2608	2539	2430	1513	1180	1590	1096	High
5523	4351	1874	2595	2805	7602	3384	4324	4905	High
2886									High
1734	92	113	121	96	1761	1972	2569	1825	High
3710	2795	2685	3206	2746	2697	2512	2765	2429	High
1859	7557	6618	7157	5658	5845	5434	5111	4666	High
									High
4492	4205	2917	3219	3378	3817	3000	3511	3058	High
	781	688	852	838	946	837	1044	923	Not Found
2869	4335	3877	4114	3274	1984	1636	2360	1458	High
324	5606	4523	4475	4004					High
11571	3360	2964	3789	3149	5040	3849	5030	4264	High
2628	3061	3166	2885	2830	7765	7412	7600	6779	High
2724	4695	3333	4465	3820	6659	4596	6063	5243	High
1859	5586	5882	6246	4920					High
4953	9566	8080	8647	9351	18305	15955	15955	17634	High
	3791	5061	4345	3264	9449	10421	10337	7409	Not Found
4587	5185	4836	4837	3988					High
	4171	3516	3593	3570	2986	2652	3075	2532	Not Found
									Not Found
4483	1520	1436	1699	1650	1469	1233	1502	1602	High
					10103	7520	9488	8847	Not Found
	4418	5253	7858	6329	11020	14394	21406	18047	Not Found
	1541	1224	1286	1144	5378	4175	5081	3875	Not Found
5326	4803	4206	4355	4316	4013	3483	3482	3253	High
	4546	3216	3756	3035	6939	6154	6818	5690	Not Found
988	1341	962	872	932	1412	1082	986	1053	High
493					2719	2627	3195	2614	High
4171	3165	2947	2957	2740	1363	1215	1297	1113	High
12120	3848	2361	3005	3224	5141	3084	3291	4431	High
11325					3228	2652	2212	2417	High
5171	2395	2277	2164	2201	6937	5505	5619	5378	High
2447	8128	8122	7978	7312					High
831					3489	2699	2887	2580	High
1790	1069	998	871	767	1878	1620	1597	1424	High
6614	5533	6192	5630	5166	1273	1687	1042	1012	High
					9775	8749	8568	8314	Not Found
6653	4187	4071	5832	3385	3024	2874	4037	2913	High
5458									High
10049	8072	6274	7251	5967					High

	1324	1737	1696	1333	1208	2070	1927	1261	High
8900	4539	2066	3427	2473	12554	8016	9811	9534	High
1597	3601	3927	3330	2867	2498	2796	2440	2193	High
	8503	8098	7411	6609	2339	2738	2160	2067	Not Found
10464					1075	737	732	806	High
	1839	1536	1869	1333	6195	4382	5044	4866	High
	9665	9252	8730	7924	7009	7405	6676	6051	Not Found
5774	4870	3983	4063	4560					High
3329					4661	4284	5448	4719	High
					3826	3144	2976	2680	Not Found
	1426	1380	1202	1339	1494	1460	1265	1249	Not Found
7049	3464	2814	3028	3292	9196	7948	8390	8430	High
7167	2161	1916	2099	1688	2925	2639	2813	2329	High
2488	2194	1933	2052	1829					High
6875	2414	1990	1966	1872	2066	1978	1758	1725	High
2265	8743	5270	6917	4927	3844	4134	4000	3741	High
802	781	783	916	744	7093	4205	4397	5598	High
1339	7745	7031	6530	5952	2473	1995	2617	1873	High
	4079	3598	3323	3215	3041	2282	2743	2461	Not Found
2996	5859	4882	4806	5733	3923	2913	2835	3858	High
2734	3751	2902	2986	2391	3322	2654	2472	2261	High
3253					1025	1067	1111	1090	High
9050	3708	4857	4567	4241	7256	8170	8263	7908	High
7506	8890	5179	6149	7042	4489	2676	3513	4038	High
5118					12616	9112	9564	8510	High
	6608	6595	7046	6530					Not Found
5303	1980	3973	4622	3407	1913	3424	3985	3133	High
7924	4369	2603	4166	2949	3257	1795	3071	2257	High
2981	4357	2946	4047	3551					High
5163	2534	2022	2405	2083	2276	1998	2149	2031	High
1111	2949	2200	2390	2301	1084	772	1063	886	High
4507					8093	7120	8853	6033	High
1781	566	594	454	513					High
1821	2804	1887	2552	2162	2902	1707	2500	1983	High
2541	2023	2714	1937	1588	1849	1942	2186	1643	High
1736	1758	1098	1497	1233	1625	1276	1520	1682	High
3547	3879	3099	3488	2953					High
3613	7810	6298	6808	6406					High
	6036	5436	4842	5166	3941	3646	4418	3335	Not Found
	3670	3334	3518	3306	4491	4102	3945	3995	Not Found
									High
4782	2591	3031	3365	3670	2585	2942	3359	3630	High
2118					359	262	314	318	High
					9111	7181	9094	7840	Not Found
7205	4450	2954	4740	4284	3672	2548	3075	3725	High
2135	4925	2883	3627	3141	3232	2098	2219	2111	High
2217	1853	1737	2043	2209					High
676	994	1037	782	939	1063	1157	912	1006	High
12115					9654	9012	9597	8785	High
4415					3499	3035	2957	2865	High

1816	1792	2063	1906	1602					High
1686					8716	6408	6869	6733	High
1510	3155	2889	2606	2687	1646	1637	1726	1309	High
1884	8101	6591	7235	6750					High
5636	9553	6053	7216	8093	10180	6640	7927	8591	High
3640					9979	9894	10237	8436	High
4099									High
	1083	1396	1340	1160	1671	1943	1917	1454	Not Found
	3863	3059	3275	2952	873	624	626	561	Not Found
	1903	1884	2109	1569					Not Found
9277	4147	4971	3880	6776					High
1625	2800	2071	2871	2249	2457	2163	2740	2354	High
883	1027	1266	1102	1031					High
1108	2181	1633	1929	1550	2220	1734	2030	1840	High
2687					2009	1632	1876	1524	High
5753	2389	2377	2381	2074	6844	6803	6016	6366	High
	3272	2685	3126	2383	6252	5749	5829	4773	Not Found
	8431	6788	6735	6250	3859	4885	4012	3987	Not Found
1073	8455	7134	6507	6994	2942	2499	2071	2586	High
	806	688	636	758	5669	5099	5160	4824	Not Found
3594	1474	1064	1468	1145					High
3844	1592	1366	1650	1346	3739	3432	3191	3844	High
7342	3178	2529	2086	2301	2898	2583	2050	2432	High
	3670	2962	3051	2842	1388	1177	1309	1103	Not Found
492	2819	2749	3226	2892	1403	1169	1131	1089	High
	1699	1561	2262	1848	1863	1613	2193	1907	Not Found
3067	6600	6404	6720	5694					High
4658					3010	3397	3313	2304	High
2159					4140	4140	4864	3924	High
3747	5368	4196	4836	3742	6259	4809	5483	4406	High
3789	6803	5696	5450	5272					High
2886	5899	4759	4812	5867	7159	6115	6219	7612	High
	2557	1889	2156	1889	8203	7856	7098	7065	Not Found
	2917	2498	2928	2389					Not Found
3785					4987	3598	4053	3827	High
10071	5654	4042	3739	4323	5588	3917	3667	4152	High
1030	1630	1504	1687	1616	5299	4428	4536	4339	High
	8807	8440	7821	6787	3351	3145	2768	2357	Not Found
2241	2280	1892	2608	1804	4934	3886	4975	4078	High
10558	1278	1384	1211	1031					High
5612	6384	6515	6988	4690	5145	5034	5349	4098	High
1919	2657	2167	2850	1886	5964	4382	5580	3900	High
5352	7189	6682	7080	7112	9446	8299	9002	8833	High
	1428	3492	3385	1979	3147	3029	3174	2873	Not Found
5535	4019	3478	3542	3353	4412	3868	3916	3818	High
3716	1036	1072	1337	999	3314	3583	3649	3108	High
6676	7607	7449	7537	7561	3358	3379	3525	3352	High
4436					3868	3359	3331	3150	High
1459					1018	1210	1171	945	High
	4560	5275	5167	4186	2287	1933	2261	2222	Not Found

614	689	637	768	666					High
3087	2304	2204	2332	2058	3032	2591	3315	2886	High
4330					237	270	262	193	High
					1904	1881	1741	1582	High
3179	1562	1484	1527	1400	5001	3481	4657	3298	High
2013	3156	2504	3220	2297	2503	2067	2496	1802	High
5015	3029	2916	2964	3253	8658	7058	8561	8138	High
5628	2889	2441	2417	2314					High
2965	4235	2920	3552	3685	4892	3514	3907	4522	High
815					4434	4011	4138	3406	High
2198	3705	2775	2654	2797	4036	3993	3643	3208	High
7665	4836	4655	5046	3912					High
3093	2273	2322	2042	2280	851	679	910	673	High
	2299	2372	2256	2022	4462	4580	5632	5123	Not Found
	8484	6446	6387	6883					Not Found
	3696	3176	3962	3433	1726	1530	1622	1583	Not Found
6183	10498	8622	10506	8279					High
4095	2971	3113	3691	2949	10945	11147	12272	10352	High
6980					5548	5827	5343	4875	High
1632	3068	2140	2345	2862	1612	1804	1876	1590	High
3902	3789	3681	3208	3899					High
	22216	27303	21714	24193	1462	1257	1393	1393	Not Found
2473	3647	3132	4440	3656	12353	10800	16422	12808	High
3392	3302	2645	2319	1869					High
7305	1100	1238	1279	1130	4271	3654	3654	4031	High
	5625	5024	4450	3967	5011	4179	3935	3370	Not Found
4480	10216	8720	8441	10205	5305	4710	3898	5586	High
					1624	2290	1921	1921	Not Found
687	2123	1940	2067	1832					High
					12908	11584	13939	13354	Not Found
4682	3869	2854	3010	3059					High
2779	1559	1893	1968	1448	2596	3992	3310	2641	High
	5031	4409	3993	3712	4176	3805	3416	3110	Not Found
2919	2794	2615	2409	2440	2576	2291	2209	2289	High
2542	2805	2430	2761	2564	7251	7003	6654	6718	High
436					646	745	693	548	High
934					935	881	714	764	High
6341	2536	2207	2472	2028	2359	2141	2370	1893	High
3084	4908	4052	4738	4516					High
318									High
2217	1778	1974	1889	1786	1557	1890	1796	1604	High
5212	6306	3972	3945	4690	7675	4663	4857	5845	High
4279	3641	2926	2852	2680					High
3889	8229	7020	7614	5867					High
1290	2441	1958	2303	1869	2086	1614	1953	1663	High
3441	6119	5551	7459	5350	10400	9810	13048	9863	High
804	2165	1946	1738	1576	2588	2226	2527	1889	High
1592	14513	8173	10011	9738	10042	6037	7002	6913	High
	3862	4067	3717	3431	5423	5775	5568	4892	Not Found
	6621	7060	6163	5912	1407	1278	1338	1140	Not Found

5101	8716	9131	8240	6799					High
1440	6548	5810	6271	5340	2759	2125	2252	1961	High
2196	2814	2182	2524	1860	3200	2096	2019	2239	High
3442	3574	4451	3925	3293	3489	4631	3880	3087	High
	3599	3304	3548	3235	1997	1571	1814	1520	Not Found
3791					7132	7523	6944	5518	High
10993	4510	2720	2496	3517	4892	2956	2677	3655	High
4345	5853	5855	5344	4770					High
5960	2799	2494	2178	3343	3434	3552	3365	4757	High
	2731	3353	2396	2524					Not Found
1284	2164	2221	1820	1764	4816	3928	4209	4180	High
4270	6400	4899	5665	5205	1121	1107	1120	1246	High
1020	2797	2310	2584	2367	2923	2387	2658	2408	High
2601	1384	1260	1247	1044	7119	4816	8158	5020	High
803	889	755	1031	669	1457	1475	1474	1241	High
	10036	7469	7973	7720	7256	5464	6285	5509	Not Found
1047	662	528	627	442	3524	2722	3491	2660	High
5366									High
2833	1403	1072	1202	1134	4464	3031	3323	3574	High
	3442	3145	3529	3255	3507	3665	3525	3053	Not Found
8676	4848	4245	4275	5076					High
2258	6347	6178	5887	6103	3620	3743	3961	4086	High
4153	15966	9045	15174	14059	5982	3628	4338	4186	High
1983	2638	3571	2589	2390					High
2626					3071	2527	2716	2298	High
					1057	1156	1078	978	High
7885					15997	13950	14968	10770	High
4866	2376	2390	2142	2332	4220	3969	3442	3961	High
254	3598	2858	2741	3151	267	234	285	258	High
	647	505	657	513	965	583	647	782	Not Found
437					1580	1274	1319	1170	High
1552					1551	1844	1439	1760	High
8771	5626	3314	3519	4168	1903	934	2171	1656	High
1038	3904	3082	3359	2917					High
4280	4769	4739	5683	5115					High
3556	4897	3815	5064	3571	677	608	692	577	High
3635	6277	3042	4955	4251	4189	2370	3681	3312	High
2670	2040	1964	1925	1751	2795	2492	2627	2381	High
	2369	2306	2385	1966	1214	1273	1489	1208	Not Found
	6824	6290	6777	5874	6389	6485	6464	5967	Not Found
	5712	2563	5066	2621	10666	2621	13348	3341	Not Found
3099	4929	3659	4844	3890	5981	4654	5866	5154	High
									High
292	909	1260	910	1080	991	1384	934	1150	High
3090	3509	5552	4192	3327	2852	4080	3476	2581	High
	2375	1877	2236	1748	9259	7658	8870	7258	Not Found
1531									High
1521					2816	2428	2339	2339	High
1674	5247	4616	4481	3313					High
1102	2713	2671	2264	2194					High

	4553	3588	5122	4349	10653	8055	11190	9694	Not Found
10782					4084	4406	5192	3515	High
7336					6792	4952	5315	5354	High
4576					5757	4383	4865	4439	High
2678	9787	6320	7568	7615	3423	2125	2549	2608	High
3604					582	837	685	621	High
6316	4359	3829	3761	3667	5649	4977	5072	5010	High
	8490	7063	8330	6853	5219	5011	5604	4716	Not Found
1336					1358	1795	1714	1300	High
2681	4065	3247	3477	3069	8638	6367	7284	7400	High
	306	305	290	279	229	161	195	226	Not Found
1479	4725	5235	4230	4161	1527	1244	1232	1139	High
2159	1983	2345	2188	1821					High
	470	618	675	531	219	262	267	215	Not Found
	1952	1384	1682	1558	3010	2549	2716	2716	Not Found
2463					5681	4430	5324	5310	High
2208	1089	972	1296	1098	1162	1022	1569	1298	High
12670					4044	2819	3423	3187	High
245	238	291	260	236	179	188	170	147	High
2161	6171	5544	5528	5165					High
5903					3077	2699	3150	2857	High
3463	1781	1477	2012	1429	3570	3661	4185	3624	High
1594	1219	1032		1022					High
6395	4280	6978	6575	6331	4093	6714	6284	6051	High
1392	6461	4433	4553	3849					High
1981					1667	2066	1908	1712	High
	3543	3649	7600	3417					Not Found
1857					1091	1094	1343	1203	High
6545	4150	4699	4158	4532					High
	3322	3094	3226	2871	4232	5031	4252	4319	Not Found
1814	2272	1950	1800	1712	2047	1995	1515	1520	High
	2872	2530	2546	2280	5578	5170	4848	4252	Not Found
					3718	3742	8559	3256	Not Found
	6114	5858	6501	4992	3469	2716	2390	2667	Not Found
1545	1837	1412	1595	1576	1533	1277	1247	1306	High
2048					3216	3027	3256	2819	High
	747	822	761	809	803	741	707	684	Not Found
6263					2806	2487	2569	2655	High
	1703	1970	1707	1470	3061	1920	2147	1849	Not Found
8167					3498	2951	2316	2203	High
535	6055	4350	4886	4303	5900	4402	4891	4568	High
	12440	9733	10477	10999	3104	2146	2545	2693	Not Found
932	1322	1022	1113	1176	2856	2288	2260	2231	High
	5447	4124	3936	4338					Not Found
11299									High
	993	839	1062	1113	1949	1738	1794	1577	Not Found
									Not Found
1186					628	825	714	832	High
7448					2836	2137	2905	2141	High
387	4185	3304	3379	2815					High

	4805	5601	4245	4192	3521	3332	3018	3138	Not Found
4304					6015	5211	5170	5596	High
3773	3707	2451	4746	3355	3368	2408	4243	2963	High
6273	3917	3965	3521	3547	3412	3409	2841	2841	High
6924					4880	3920	4236	4426	High
2226	9623	7718	8915	8161					High
3313					6604	6281	5265	5043	High
	5252	4420	4262	3696	3103	2586	2392	2136	Not Found
	1294	1366	1277	1201	1892	1924	1582	1657	Not Found
2936	3522	2830	2848	3226	3773	2768	3028	3318	High
2211	4520	4209	4329	3749	6310	5658	5345	5209	High
	2120	2034	2245	1881	1540	1482	1485	1288	Not Found
328					4411	4108	4435	3548	High
2189					3987	3049	3687	4747	High
1375					1456	1761	1575	1334	High
90	459	226	298	250					High
1411									High
	6655	6456	6059	5708					Not Found
4831	3558	3116	2855	2683	4148	3374	3514	2992	High
912	1210	1054	1012	963	1076	985	965	817	High
2736					6934	4914	5082	4797	High
2373	2081	2094	2046	1824	2640	2508	2660	2231	High
7357	3243	1903	2009	2475	3304	1997	2202	2453	High
	5410	4770	5219	4993	1369	1558	1456	1437	Not Found
493	746	959	861	729					High
1093	4179	4065	3804	3524					High
1888	2161	1765	1993	1619	4515	3372	3975	3489	High
4570					7279	5464	4322	4524	High
2194									High
	4677	3696	3794	3673	2915	2470	2445	2272	Not Found
3917					9618	9303	8507	8638	High
7987	4801	4621	5767	4717	3296	3152	3500	2672	High
2360	6458	5013	5428	5040					High
1601									High
	6038	5153	5603	4595	5115	4671	4831	4255	Not Found
2722	2766	1800	2290	2628	1798	1209	1388	1505	High
4047	5555	4276	4465	4030	4817	3764	4198	3556	High
	9660	7724	7518	7250	4035	2836	3585	3330	Not Found
	1704	1561	1937	1662	6195	3886	4685	4407	Not Found
	5736	5724	5715	5628	4088	3832	3839	3674	Not Found
3196	6113	4252	4897	5249	6005	4330	4905	5291	High
	3985	3090	4411	4179	6745	6726	7692	7030	Not Found
2433	5666	2089	4500	1973					High
2712	3013	2502	2894	2717	3326	2778	3138	2885	High
12348									High
1971					3548	2825	3168	2803	High
5308					6140	4386	4348	3904	High
	3694	3121	3591	3801	4813	4093	4701	5147	Not Found
3918					5086	3950	3770	4181	High
4629	4141	4011	3879	4025					High

	5800	5582	4716	6643	2329	1930	1899	2628	Not Found
	2261	2716	1944	1825					Not Found
	10003	7629	9818	8116					Not Found
4038	4789	3549	4011	4006	4660	3499	3914	3613	High
	2423	2045	2773	2338	2657	1901	1979	2180	Not Found
3548					10702	9326	9282	8386	High
6123	3617	2813	2832	3329	5463	4873	4265	4238	High
1345	2499	1752	1728	1998	2973	2337	2354	2475	High
					3724	3515	3594	3062	Not Found
5627									High
1399	8083	6268	6128	5509					High
3910									High
					3015	1374	3548	1455	Not Found
	3455	3313	3460	2780					Not Found
									Not Found
	2600	2149	1709	1825	5127	4324	4251	3874	Not Found
2272	2193	2132	2087	1870	1761	1405	1857	1492	High
	4051	4103	3229	4327	3204	3431	2698	3747	Not Found
	7698	7881	7545	7524					Not Found
2568					2543	2593	2593	2379	High
	4948	4285	4387	3617					Not Found
	2695	2403	2667	1962	2984	2521	3155	2055	High
3025									High
2096	2570	2926	2890	2554	2497	2999	2496	2257	High
9063	3870	3034	3497	3012					High
	2968	2488	2215	2396	1360	1591	2014	1286	Not Found
2639	1849	1775	2619	1750					High
5116					4636	3264	3940	3670	High
	4800	3996	3972	3899	6487	5804	5950	5370	Not Found
314									High
	3865	3192	3670	3710	6663	5472	6440	6871	Not Found
7913	2350	1834	1896	2008					High
4373	4511	4648	3824	3488					High
548	779	664	611	635					High
2445	935	1368	1399	1049					High
					7450	3733	3680	4454	High
1703	1287	1017	1118	1064					High
6769					4617	3933	4870	4442	High
3362	4640	3753	3880	3736					High
	3137	3000	3489	3100					Not Found
	11596	13912	15153	16105					Not Found
	3961	4971	4290	4061					Not Found
	2659	2688	2465	2481	3409	3094	2705	2977	Not Found
	12816	11779	9629	11237					Not Found
	1897	1612	2254	1633					Not Found
4743	2481	2186	2260	2102					High
	6420	4736	5990	4433	4834	2514	4153	2777	Not Found
5088	2769	2884	2785	2715	5117	4348	4309	4421	High
	2048	1722	1768	1598	4558	4444	4370	4496	Not Found
	2626	1863	2050	2267	3677	2353	2680	2768	Not Found

	3999	4653	4464	5726	1817	1818	1709	1464	Not Found
1611					4178	3710	3785	4161	High
	2929	3427	3169	2805					Not Found
2036	2921	2333	2418	2437					High
	4832	3809	3805	4095					Not Found
	11676	5312	11104	5540					Not Found
	1839	3826	2954	2880	2359	2841	2655	2213	Not Found
5354	2564	1810	2476	2614					High
5720	1442	1269	1440	1517					High
1974	1388	1208	1268	1162					High
	11177	9736	10563	9691					High
5667	6820	6070	5338	6216	3273	2356	3153	2357	High
3617	5082	4430	4656	3736					High
					3031	2889	3040	2912	Not Found
4665	10308	6012	7363	6271					High
	11294	8910	10613	9778	1532	1346	1143	1284	Not Found
2467	8951	8206	8639	7848					High
3115	3211	2494	2278	2588	4348	4588	3382	4540	High
	8730	7530	6077	5355	7065	6163	5324	4386	Not Found
3514	4201	3277	3639	3339	5146	3955	4407	4193	High
1709					2346	2157	2082	2082	High
	4506	4778	4431	3772					Not Found
4227					2440	2426	2003	1780	High
2960	2751	2455	2441	2218	3190	2934	2941	2626	High
2268					2793	2619	3428	2366	High
990	2296	1673	2238	1816					High
5538	3564	2787	2123	2639	6814	3342	5152	5225	High
4501					1658	1419	1346	1153	High
2791	2940	2980	2472	2537					High
3509	5228	5656	4566	3765	3353	2965	2945	2245	High
1817					3458	2213	3070	2106	High
5236	13017	17428	13931	16141	3263	3324	3414	3247	High
3899					1616	1636	1600	1236	High
	7165	8073	6811	6259					Not Found
	12635	14139	12315	10875					Not Found
1173					707	578	505	605	High
4504	5210	4790	3531	5016	3768	3508	2603	3531	High
	2288	1804	1942	1870	1947	1605	1818	1483	Not Found
3894					1087	1195	1088	1221	High
3026					938	1192	1318	932	High
8215									High
2817					3154	3174	3193	2879	High
	4675	3569	3847	4383	9152	6953	7840	8725	Not Found
1427	6394	4974	4969	4720					High
1923					2825	1659	2497	2379	High
2683	762	628	712	581	772	620	809	584	High
	791	916	912	801	718	944	883	740	Not Found
2525					4893	4348	4594	4396	High
1862					759	675	695	641	High
1857	6164	4500	5041	4652					High

2221	2690	2146	2209	2114					High
	5132	4738	4092	3607	604	525	602	461	Not Found
4972	6896	4014	5257	4602	4489	2767	3519	3057	High
					5480	5038	5240	4492	Not Found
	1958	2104	1975	1856	1746	1865	1753	1619	Not Found
1420					1232	1240	1263	1047	High
	6617	5212	5106	5106	3562	3763	3743	3389	Not Found
	1519	1729	1528	1552	1485	1715	1464	1694	Not Found
	6472	5355	4704	5285					Not Found
	5235	4364	4383	4075	3521	2609	2730	2750	Not Found
					689	641	720	698	Not Found
2434	8461	7005	5664	5259					High
4626					6023	6324	5970	7401	High
372	844	726	674	622					High
2537	2805	2784	2396	2322	2968	2968	2810	2555	High
	3662	3653	3886	3365					Not Found
563	470	390	419	361					High
3810					5491	4888	5397	4700	High
444	5594	3433	5111	4439					High
	4208	3235	3602	3601					Not Found
6629									High
									High
3316					3980	3141	3521	3049	High
1715	1735	1316	1260	1306	3387	2084	2157	2530	High
681	2315	1915	2398	1988	395	344	344	302	High
2982	5090	3654	4332	2947	3970	2248	3447	2472	High
	2790	2156	2521	1886	2604	2035	2310	1936	Not Found
	1955	1906	1985	1480					Not Found
2701	1185	1148	1187	920					High
860					711	397	465	447	High
4885					6427	6169	6582	5699	High
	4458	4027	3805	3733	3392	2801	2624	2803	Not Found
					9010	6295	7723	7420	Not Found
514					3911	3691	3863	2970	High
2404					2245	2400	2161	2069	High
218					198	237	191	193	High
3487	2091	1673	1663	1674	4836	4381	4555	3658	High
4904	3717	4659	4141	5020	2697	3395	3175	3412	High
2585									High
1913	1191	1408	1222	1070					High
5354									High
3064	3992	4688	3805	3619					High
2037					3596	2409	3018	2380	High
3456	3528	2931	2762	2988					High
1353	1260	1346	1736	1292					High
1605					2521	2136	2119	1811	High
	1333	1024	1217	951	3610	2754	3970	2910	Not Found
979					7875	6467	7385	6542	High
3946	207	158	171	143	593	556	603	481	High
1805	4797	4682	4199	4624					High

	5944	5880	4683	5044	5789	5578	4493	5113	Not Found
	1418	1245	1109	1195	814	893	773	735	Not Found
937	654	1040	959	706					High
1576					4799	4048	4217	4164	High
5576									High
3666	7522	5325	5855	4888					High
									Not Found
396					462	443	412	376	High
2551	2467	1825	2555	2140	1198	925	1225	1040	High
	4047	3205	3282	3240	2062	1722	1944	1746	Not Found
3296	7637	6310	6306	4784					High
4695	8367	7691	8817	7976	8684	7792	8525	8159	High
965					3215	3627	3606	2777	High
	2736	2052	3261	2154	2394	2136	3057	1936	Not Found
1583					6579	4843	4724	4306	High
3413					3720	2860	3728	3025	High
1040									High
2333	4487	3910	3896	3604					High
1663					2754	2588	2425	2364	High
811	1990	1408	1692	1318					High
4256					8227	8048	7815	7071	High
	4460	5161	4465	4483	4565	5296	4067	3300	Not Found
2652	2611	1816	2464	1908	2940	2006	2644	2059	High
	4135	3690	3388	4743	1797	1635	1569	2162	Not Found
5869	3042	2484	2788	2250					High
1207					3003	2522	2121	2493	High
1761					1720	1839	1317	1499	High
	3138	2126	2229	2097	2833	2079	1958	1801	Not Found
3785	4296	3711	3041	3460	3918	3680	2869	3423	High
4662					650	498	559	479	High
5920					3471	2812	3164	2956	High
4930	5149	3891	4770	4211	6496	4965	5885	5743	High
10665									High
3689	6330	3457	4099	3296	5417	3098	3438	2792	High
2375					2302	2316	2196	1950	High
					947	1157	836	999	Not Found
1845					3719	3696	3923	3311	High
	1218	1320	1169	1147	1416	1211	1295	1161	Not Found
3773					4316	4217	5215	3956	High
3144	3879	4244	4696	3499					High
3167	4211	3416	5229	3910	4055	3231	4858	3850	High
									Not Found
	1166	1181	1319	972					Not Found
3645	4518	3779	3605	3676	4576	3792	3667	3644	High
2254	2633	1922	2028	2115	2607	1809	2021	2183	High
2354					2215	2572	3670	1959	High
									High
	2404	2116	2715	2091	3299	2738	3375	2858	Not Found
946	975	660	829	824					High
2681	2717	2973	2513	2758	2290	2367	1955	2129	High

									High
1414	1352	1150	1174	1313	1604	1275	1356	1388	High
4441					3531	2205	3037	3114	High
	4844	2923	3494	3700	5049	2805	3584	3841	Not Found
1348	620	586	786	571					High
5278					4482	3252	2995	3239	High
	7859	6239	5844	5288	8114	6650	6556	6753	Not Found
5928	9197	8873	6993	6993					High
	1006	2520	2240	1419	1720	5784	5221	2802	Not Found
	1017	1190	1216	1003	1075	1250	1419	1139	Not Found
2552					1303	1177	1438	1186	High
2123					5527	4850	4171	4336	High
2669					3219	2485	2694	2914	High
2057	1654	1789	1646	1904					High
	785	876	877	607	1063	1024	1000	897	Not Found
	737	691	623	545	1854	1494	1551	1475	Not Found
	5505	5126	5137	5471	3869	3938	3532	3773	Not Found
	1516	1788	1795	1389					Not Found
					4566	3944	3861	3077	Not Found
									High
4385	5975	2966	3771	5231	6050	2920	3970	5218	High
					9078	8587	8998	6996	Not Found
4725					4257	3397	4247	3437	High
4124	3067	4190	3807	4196					High
2769					9300	7767	9648	7663	High
435	2597	2049	2122	1704					High
6533					4812	3136	3593	5048	High
	4209	4108	4343	3239					Not Found
11371									High
					3648	3557	3557	2726	Not Found
	3953	3351	3702	3377	1223	1392	1321	992	Not Found
	7436	5850	7213	5984					Not Found
4147	4728	3980	3952	4058	4732	4110	3956	4169	High
3611	5408	3651	3154	3478	5890	4175	3420	3916	High
	1069	792	758	758	2752	2588	2431	2099	Not Found
3467	3595	3358	3059	3224					High
462	282	251	168	236					High
					2497	2755	2059	2170	Not Found
2124					2679	2495	2688	2355	High
					6887	6558	5682	5763	Not Found
	10635	9465	8612	12509					Not Found
1279	970	1153	1038	1013					High
1793					394	288	412	340	High
	12676	11037	9858	9019					Not Found
					8384	7897	6571	7387	Not Found
	3637	4171	3065	2639	5853	7339	5078	4142	Not Found
	2617	3159	2872	3126	3249	3738	3397	3504	Not Found
					4931	4433	4134	4055	Not Found
2064	3151	2790	3449	2815					High
	387	297	312	286	494	367	372	327	Not Found

6898									High
1505	902	868	1055	838					High
289	342	299	274	300					High
2485	2565	3099	2939	2197	2393	2844	2871	2038	High
	3128	2024	2202	2202	2062	1431	1601	1697	Not Found
2796	5028	4275	5948	4483					High
1855					1668	1355	1453	1146	High
3564					1250	1026	1147	990	High
	4779	5275	3735	4751	5232	5740	3958	5121	Not Found
	6355	4968	5726	4568	6618	4774	5924	4749	Not Found
									Not Found
1894	1422	1375	1156	1186	3167	3105	2254	2293	High
2856	3510	3187	3340	3065					High
	3597	3297	2556	3032	11359	8638	10145	9703	Not Found
	5623	5518	5356	3731	7167	6750	6480	4821	Not Found
6091	6613	4471	5993	5524	3701	2575	3420	3343	High
	1779	1513	1585	1468	6619	3405	4251	5024	Not Found
3542	2944	2048	2717	2406					High
1375					1725	1356	1458	1291	High
	2746	2336	2352	2261	1408	1076	1216	1079	Not Found
2084	2521	1717	2761	2342	1585	1244	1748	1448	High
					2591	2227	3256	2093	Not Found
3812									High
	2283	1961	2825	1941	2382	1998	2558	1804	Not Found
3771	4352	3846	3731	3792					High
	1764	2208	2088	1755					Not Found
					1779	1681	1293	1367	Not Found
3339	4823	3874	4100	3770	4283	2957	3661	2924	High
	2159	1878	1810	1601	3019	2536	2764	2408	Not Found
1815	3247	2916	3040	2618					High
1963	1624	1650	1953	1576	2485	2342	2520	2170	High
					1441	1214	1317	1115	Not Found
	3280	2933	3482	2982	3165	2839	3267	2614	Not Found
									High
1991					2288	1705	1784	1613	High
	3076	3021	3065	2669					Not Found
1717	1812	1481	1632	1632	1659	1440	1492	1455	High
739	1075	831	971	996					High
3816	4992	3549	4503	3819					High
1350									High
4725					6703	5471	4652	4877	High
	4358	3644	4052	3583					Not Found
	8435	8135	7378	6820					Not Found
2124									High
	909	596	732	770	935	678	782	777	Not Found
1205									High
	526	561	440	664	702	632	514	1164	Not Found
4970	5584	4772	5633	5097					High
2286	4479	4300	3728	3849					High
1775	1246	1418	1469	1099					High

754	499	398	541	406					High
2573	2720	1879	2164	2138	2946	2447	2876	2498	High
	3742	2846	3521	2871	5326	3866	5358	4065	Not Found
									Not Found
5600									High
6758									High
	1705	1766	1884	1466					Not Found
	2839	2575	2455	2672	5503	4366	4260	4179	Not Found
	5841	4026	3481	3862	9227	5959	5651	6036	Not Found
5274	209	160	225	203	3192	3192	3622	4251	High
4149	1395	1343	2120	1541					High
	3147	3084	2772	2737	2272	2318	2056	1865	Not Found
768	1349	1242	1221	1088					High
	4468	4283	5224	4446					Not Found
4544					7334	6747	5733	5999	High
	3174	3254	3766	2436	3908	3545	4245	2939	Not Found
2777	2422	2195	1943	2312	2664	2338	2217	2564	High
	1270	1482	1168	913	3819	5290	3270	2755	Not Found
					729	773	694	681	Not Found
									Not Found
3094	5488	4382	3992	3845	4720	3752	3377	3313	High
	480	363	435	363	4431	3675	3956	3833	Not Found
2932									High
1018	1276	1377	1441	1248	1579	1762	1389	1297	High
1916	2230	2165	1869	1628					High
	4497	3419	3402	3178	5155	3986	3950	3601	Not Found
1983					1469	1359	1955	1443	High
3025	3368	3257	2680	3667	4085	3661	3085	4041	High
1331					587	518	510	556	High
2585	2747	2273	4047	2447	2373	1926	3740	2292	High
3830	2197	1715	2115	1840					High
	3868	2343	3020	2770	4190	2308	3214	2881	Not Found
5258	3759	2243	3238	2732					High
2342	2458	2371	1875	2003	6072	4647	4505	4392	High
2315	6338	3764	5593	5459	6183	4149	4889	5291	High
									Not Found
1340	1298	1401	1268	1150					High
	4569	3614	5867	4469	3692	2601	4529	3347	Not Found
3722	2288	2076	2721	1975	2859	2562	3052	2385	High
1045	1281	1547	1438	1297	786	690	750	646	High
					2858	2585	2184	2364	Not Found
5375	2918	2473	2457	1962					High
1648	3204	2197	3220	2533					High
2256					2751	1712	2713	2315	High
2509					2258	1919	2376	1701	High
1724					5223	5077	5969	3780	High
2946					6868	5289	6868	5887	High
	245	272	213	222	6934	6356	6430	5936	Not Found
675	1971	1675	2106	1515					High
1420									High

	10042	8828	10592	9725					Not Found
	10815	10815	9734	10114	3410	2523	3181	3149	Not Found
2508	2570	2482	2666	2053					High
2520					3120	2372	2740	2374	High
2892					5296	3925	4110	5000	High
	2800	2442	2037	2205					Not Found
					3473	2837	2503	2569	Not Found
2683	2004	1634	1616	1606					High
1797					2379	2050	1954	2113	High
					1030	836	990	928	Not Found
					6510	3263	6012	3520	Not Found
	4940	4171	4035	4547	4958	4406	3804	4827	Not Found
3948					4996	5028	4898	3929	High
1368									High
	3295	3322	2705	2148	2309	2244	2133	1476	Not Found
6251									High
3387					1735	1575	2063	1309	High
4525					5118	3959	4067	5047	High
	5063	3440	4034	3891	3742	2726	3508	3422	Not Found
	6276	5465	5978	4706					Not Found
4450	5239	4353	3924	4514					High
	1766	1120	1422	1282	1276	897	875	842	Not Found
					3609	2923	3122	3500	Not Found
	4841	3485	4326	3965	1001	734	910	735	Not Found
2088					4941	3309	4710	4433	High
					1196	1366	1077	1078	Not Found
	4214	2823	3638	2968	6762	5652	5112	4830	Not Found
5387									High
983									High
3619	5679	4488	3920	5001					High
2181					2128	2069	1566	1684	High
4123									High
2568									High
	453	316	420	369					Not Found
	3985	4006	3022	3005					Not Found
2393	3556	4011	3160	3305					High
	1429	2016	1806	1613					Not Found
3409									High
					2189	2075	1559	1688	Not Found
1951									High
1063	363	183	278	302					High
					5066	4768	4398	4079	Not Found
					1644	1206	1593	1168	Not Found
4810					5518	3344	4368	4039	High
2897	4527	4640	4274	3833					High
	2888	1736	3297	1255					Not Found
4480					5973	4870	3766	4441	High
2071									High
									Not Found
4467									High

6573	3952	2629	2890	3155					High
	6106	5241	6675	4601	5110	4526	5397	4198	Not Found
					5728	4523	5023	4198	Not Found
4815	5119	5324	4241	5382					High
					6420	5345	5892	4748	Not Found
									Not Found
343	442	261	343	367					High
	9657	9007	7255	8734	3657	2625	3184	2824	Not Found
1817					3893	2347	3039	3335	High
9819									High
	805	759	578	654					Not Found
	1708	2106	1874	1459					High
					1645	1522	1542	1308	Not Found
	2011	2188	1937	2055					Not Found
867	1704	1443	1430	1328					High
3267									High
	6183	4505	4126	4319					Not Found
	419	547	603	550					Not Found
	1233	1542	1279	1096					Not Found
595									High
1370					3961	3394	3489	2923	High
3466					4691	3647	4163	3585	High
1661					3440	2786	2622	2525	High
	180	149	145	203					Not Found
	2277	2864	2647	2126					High
1859									High
	6549	4664	4760	5126	9743	6443	6156	6487	Not Found
					2207	2064	1906	1737	Not Found
491	1406	1299	1307	1125					High
555									High
4953									High
2252									High
	5217	4654	4626	4557					Not Found
	2348	2423	2480	2147					Not Found
	1136	1725	1417	1143					Not Found
					4715	4461	5515	3907	Not Found
	2697	1902	2112	1750	3433	3412	3607	2464	Not Found
	2457	1996	2693	2214	3142	2534	3402	2792	Not Found
	2154	2397	2044	2090					Not Found
	2741	2707	2644	2744					Not Found
	3814	3339	3149	3014	1874	1792	1933	1557	Not Found
	3328	2949	3049	3288	2869	2734	2464	2880	Not Found
1224	2112	2043	2272	1416					High
2444					4961	3709	2912	3274	High
									Not Found
	2309	1998	2185	1752	3022	2109	2878	1983	Not Found
	4018	3994	3647	2597	4195	4315	3821	2802	Not Found
4928									High
5018					7890	7050	6205	5059	High
	4411	3895	3842	3223	6875	6380	6118	5366	Not Found

7688									High
1441									High
3100	4361	2025	3589	3016					High
	2194	2182	2250	2101					Not Found
1772	3977	3458	3855	3668					High
591									High
2945									High
					1773	2097	1753	1870	Not Found
1165									High
					9339	7694	7284	6475	Not Found
4147					1683	1464	1701	1532	High
941									High
1179					1551	1081	1201	1231	High
3498	7183	5342	5818	6440					High
	1127	1049	1128	962					Not Found
	5215	5575	5443	5727	2588	2735	2577	2752	Not Found
									Not Found
					2512	2434	2495	2235	Not Found
	3107	2839	2912	2672	2971	2660	2864	2613	Not Found
					4940	3308	4443	3883	Not Found
									Not Found
	778	860	746	697					Not Found
									High
	2402	1999	2117	1966	2140	1948	2038	1892	Not Found
3725					3681	3017	3325	3448	High
1844									High
	3756	4442	5068	3271					Not Found
					1491	1324	3005	1226	Not Found
3052					2372	2260	2508	2172	High
2667	1723	1547	1714	1484					High
	2111	1885	2133	1900	3207	2710	3231	2913	Not Found
2401									High
	4709	4618	3888	3832					Not Found
					1408	1301	1265	1182	Not Found
4205	6298	3482	5135	3743					High
	3318	4977	3913	3412	3806	4370	4080	3456	Not Found
4900	3459	2666	2339	2464					High
2218									High
	1070	901	894	922					Not Found
									Not Found
1982					3343	2376	3092	2433	High
3220									High
	1511	2720	1313	2250					Not Found
					4312	3732	3937	3456	Not Found
	1852	2138	2078	1929					Not Found
	4583	4069	3439	2978					Not Found
11461	5453	4251	6496	4118					High
	2455	3713	2509	2212					Not Found
2507									High
	1449	1836	1781	1351					Not Found

	2516	2863	2765	2432					Not Found
	2352	1978	2231	2137	2007	1715	2121	1867	Not Found
1988									High
1506									High
694	1413	1117	1062	1126					High
205					157	205	137	163	High
									Not Found
	1315	2064	1174	1467					Not Found
1991									High
3937									High
613	656	677	1518	710					High
3523					4262	3824	4397	3684	High
	1731	1534	1058	1902					Not Found
					3388	3785	3020	2982	Not Found
									Not Found
	2891	2899	2037	2735					Not Found
5225									High
					3354	2920	2938	2924	Not Found
					3495	3365	3750	3392	Not Found
	6657	4195	5110	6132	6891	4691	5339	6033	Not Found
	986	1135	1033	1017					Not Found
					1887	2253	1930	1635	Not Found
1061	3156	2780	2959	2742					High
					663	459	561	481	Not Found
					2378	2173	2377	1928	Not Found
					1228	1220	1128	1209	Not Found
									High
	359	422	328	422					Not Found
3457	3844	3113	3203	3123					High
1011									High
	2735	3268	3172	2648	3004	3554	3736	3048	Not Found
					1191	1081	1123	1069	Not Found
2090					4713	2210	2213	2080	High
					1681	1727	1369	1642	Not Found
	3982	3759	3699	3455	4228	4202	3481	3415	Not Found
									Not Found
					738	682	827	607	Not Found
237									High
2814					3508	2644	2693	2562	High
	3019	2649	2628	2353					Not Found
2900									High
	3473	2701	2872	2630					Not Found
3280									High
					1103	989	1167	920	Not Found
2263	3092	2135	2501	2371					High
	1722	1280	1750	1209	2133	1647	2300	1537	Not Found
1808									High
4190	3641	3034	2872	3328					High
					4235	3850	3288	2832	Not Found
1185									High

3736					7348	6091	5794	5573	High
1445									High
2306									High
	5441	5189	4638	4244					Not Found
	2785	4372	2751	2538					Not Found
									High
					1928	2379	2234	1893	Not Found
	2899	2395	2593	2100					Not Found
	1011	1020	1099	901					Not Found
	2005	1759	2117	1727	3510	3093	3218	2912	Not Found
									Not Found
1537					2440	2704	2162	1729	High
1413									High
	1499	1115	1454	1072	1774	1273	1760	1336	Not Found
	1322	955	888	1108					Not Found
	2860	2956	2881	1925	9155	10009	8982	6494	Not Found
	2670	4276	3750	2790					Not Found
					1080	1641	1182	1309	Not Found
3868					5019	4035	4099	4047	High
1093									High
3619	4639	4504	4783	4844					High
4621	4301	4489	5909	4622					High
2049									High
					2757	4450	2644	2358	Not Found
									Not Found
					471	498	390	492	Not Found
4197	5242	3392	4797	4127					High
	2633	2752	2616	2216					Not Found
	5498	6743	6088	5676	2068	1651	1890	1558	Not Found
5178					4277	2738	3437	3475	High
1327									High
					608	551	707	574	Not Found
									High
92									High
									Not Found
					3043	2987	3137	2747	Not Found
725									High
									Not Found
					2702	3980	3247	3247	Not Found
									High
									High
					1210	1123	1207	920	Not Found
					3639	3844	3939	3585	Not Found
	1393	1612	1672	1134					Not Found
2270					3508	2629	2649	3365	High
1779					3973	3851	3640	3057	High
3098	4677	3769	4508	3925					High
					1633	2491	2073	2273	Not Found
1572									High
2026	2595	2400	3043	2307					High

	1761	1447	1592	1534	1928	2003	1893	2022	Not Found
2044									High
	2304	2719	2904	2171					Not Found
	1558	1591	1429	1385					Not Found
					2699	1459	2250	2011	Not Found
5177					9405	5797	7522	5995	High
2767									High
4059					3530	2830	3415	3125	High
	1405	927	1422	958					Not Found
	5707	4223	4737	4506	9556	7631	7744	7398	Not Found
1812									High
	2410	3661	2694	2468					Not Found
1581	3194	2493	6873	2742					High
	2685	3357	2641	2366					Not Found
	1679	1290	1620	1312	6146	7422	6923	5447	Not Found
	3055	2923	2417	2528	3546	3442	3131	3193	Not Found
	3153	1875	3602	2116	4734	3231	5690	3037	Not Found
									Not Found
3863									High
	1237	1152	1168	975					Not Found
2374	3247	1780	2055	2294					High
					3356	3063	3117	2965	Not Found
									Not Found
									Not Found
									Not Found
					352	294	302	302	Not Found
									Not Found
	6155	6310	5975	5718					Not Found
									Not Found
									High
					1852	2330	2376	1811	Not Found
4388									High
					3484	2290	2799	3062	Not Found
	2657	2809	2040	1917	3584	4443	2498	2450	Not Found
					1358	1195	1544	1047	Not Found
	3840	3098	4116	3202					Not Found
									Not Found
1355									High
									Not Found
740									High
402									High
6662					6819	5737	5298	5102	High
480					2340	1911	2107	1820	High
	1896	2476	2339	1556					Not Found
	1728	1457	1809	1320					Not Found
	3147	4220	3506	2727					Not Found
					1708	2419	1856	1810	Not Found
									Not Found
									High
	1251	1301	1444	1084					Not Found

									High
									Not Found
1463	2401	1885	2327	1528					High
3543	2926	3275	3164	2896					High
	5595	3957	4816	3982	3797	2462	3044	2636	Not Found
									High
	2526	2377	2862	1981					Not Found
					1453	1578	2182	1464	Not Found
									Not Found
									High
	3491	2701	2784	2747	4526	3460	3239	2820	Not Found
	1981	1753	1692	1519	3418	2660	2477	2480	Not Found
									Not Found
	2229	2299	2178	2346					Not Found
	3690	3449	2753	3728					Not Found
									Not Found
1538									High
									High
					1186	1172	1019	1062	Not Found
1207									High
									Not Found
	1875	1414	2197	1320	2393	1732	2681	1767	Not Found
	1018	1210	1184	927					Not Found
3295					1931	3499	3561	3016	High
									Not Found
	2319	2810	2314	1939					Not Found
2033									High
					309	252	223	260	Not Found
	6983	8212	7350	6403					Not Found
					4524	2674	3785	3446	Not Found
3516					3798	2946	2806	3245	High
2338									High
3704					3541	2768	3425	2854	High
					1378	1088	1711	1193	Not Found
	2536	2332	2657	2111					Not Found
	514	419	429	474					Not Found
1663	3869	1937	1883	1748					High
	3353	4610	3540	3955	3130	4119	3412	3596	Not Found
3073									High
					184	178	126	234	Not Found
					941	836	693	882	Not Found
4198					2568	1828	2116	1942	High
3967					3608	3217	3577	3289	High
2833									High
2537					2823	3427	3794	3207	High
	436	344	422	389					Not Found
					3667	4220	4485	3887	Not Found
	1619	1640	2064	1521					Not Found
2278									High
4061	4270	2921	3825	3319					High

1198					1494	1078	1268	1029	High
3296									High
					2031	1739	1956	1806	Not Found
3276									High
	15400	14158	12230	10846	15271	14606	12575	11339	Not Found
1745	3656	2839	3434	2412					High
3054	2586	2212	2362	2545					High
	4664	3595	4261	3609	3584	2740	3077	2820	Not Found
									Not Found
6341									High
7430									High
					2161	2195	2625	2152	Not Found
3226									High
	3493	4042	4014	3016					Not Found
					3776	4010	4519	3630	Not Found
	986	918	761	659					Not Found
3795									High
					458	395	439	531	Not Found
4162									High
	3018	3569	3253	2873					Not Found
2474									High
	3730	723	3479	795					Not Found
	1979	1321	1321	1242					Not Found
					550	626	547	485	Not Found
	1322	1451	1407	1283					Not Found
					2273	2403	1987	2000	Not Found
					2939	2574	2729	2390	Not Found
	4852	3676	3826	4026					Not Found
					4957	4237	5217	4486	Not Found
	2303	2316	2474	1954					Not Found
					2301	2416	2200	2080	Not Found
	4099	4416	3301	2910					Not Found
5148									High
					1435	1732	1600	1432	Not Found
	1144	899	968	801					Not Found
					542	572	565	537	Not Found
	4424	3994	3736	3505					Not Found
3177									High
					888	768	761	807	Not Found
	1131	989	1022	791					Not Found
	3415	1353	1325	1256					Not Found
924									High
1214									High
					1508	1204	1386	1220	Not Found
					2471	2264	2172	1913	Not Found
955									High
	4610	3553	3474	3025					Not Found
1612									High
	1796	1544	1512	1234					Not Found
547									High

					1011	1137	988	964	Not Found
	661	744	798	630					Not Found
					6901	7006	6924	5739	Not Found
	2641	2476	2550	2793					Not Found
					4796	4488	5282	4088	Not Found
					5629	7321	4972	5525	Not Found
	3062	3120	3873	2652					Not Found
	1379	1119	1068	1015					Not Found
	1817	2251	2091	1825					Not Found
					1825	2105	1546	1787	Not Found
	6517	7009	7625	7304					Not Found
	2053	1454	2368	1616					Not Found
	4807	5320	5733	3764					Not Found
1484									High
	2100	1597	1804	1605					Not Found
					2874	2400	3036	2314	Not Found
	1715	1370	1361	1272					Not Found
					3880	2953	4554	3553	Not Found
	9367	8506	9026	8020					Not Found
1909									High
					4266	3900	3895	3541	Not Found
					2122	3428	3581	2984	Not Found
4048									High
1847									High
3299									High
	5001	3904	4062	3818					Not Found
					4451	4414	4871	4663	Not Found
	1601	1300	1491	1415					Not Found
1618									High
	3273	3436	3599	2548					Not Found
3070									High
1503									High
					2980	2769	2643	3092	Not Found
4409									High
					1044	1123	1094	993	Not Found
1923									High
287									High
					2074	1795	1452	1528	Not Found
2341									High
									Not Found
1178									High
					2409	2214	2567	2070	Not Found
	6266	5190	5117	5883					Not Found
	3209	3024	2814	2384					Not Found
					2003	1688	2149	1638	Not Found
667									High
	1089	989	1094	991					Not Found
1698									High
					4580	3366	4071	3724	Not Found
					1182	1182	1290	914	Not Found

	3545	3230	2555	2691					Not Found
	1754	1900	1836	1705					Not Found
1497									High
984									High
					1273	1442	1036	1180	Not Found
					1863	1656	1810	1380	Not Found
					6894	6298	6670	6317	Not Found
					1579	1329	1311	1534	Not Found
					1732	1136	1758	1279	Not Found
870									High
					3905	3360	3429	3494	Not Found
	3290	3704	3522	2814					Not Found
									Not Found
									Not Found
1473									High
1088									High
4131									High
4333									High
	2946	2456	3000	2265					Not Found
	2479	1980	2261	1745					Not Found
653									High
	2097	1701	2226	1795					Not Found
	2492	2094	2401	2056					Not Found
					3414	2420	2430	2128	Not Found
					622	675	581	578	Not Found
581									High
	3377	4333	5690	3379					Not Found
					7590	6703	5922	5923	Not Found
1169									High
									Not Found
	687	737	656	568					Not Found
	3079	3002	2893	2955					Not Found
	935	1207	1070	1326					Not Found
966									High
					4779	3953	4362	4149	Not Found
	2307	2168	2775	1895					Not Found
1887									High
	5899	5165	5399	4499					Not Found
	563	454	523	486					Not Found
					2812	2563	2149	1995	Not Found
					4890	6882	4941	5277	Not Found
	844	730	1029	719					Not Found
	7793	8040	7268	6438					Not Found
	777	582	739	603					Not Found
					4790	4248	4249	3790	Not Found
					1036	972	898	782	Not Found
									Not Found
									Not Found
									Not Found
					4063	3224	3951	3843	Not Found

									Not Found
									High
	6463	5325	4269	5633					Not Found
	1483	1390	1484	1049					Not Found
					2773	3106	2626	3111	Not Found
1524									High
2535									High
236									High
					3726	3112	3692	2949	Not Found
					1734	1756	1687	1464	Not Found
					1901	3087	2132	2426	Not Found
2975									High
									Not Found
					2389	3798	3174	3057	Not Found
3517									High
					1931	2028	1722	1628	Not Found
1528									High
					2689	2739	2652	2309	Not Found
802									High
	687	752	760	706					Not Found
	5131	4237	4984	4371					Not Found
									Not Found
	2115	2049	2315	1881					Not Found
	4114	3986	4657	3731					Not Found
									Not Found
	2461	2116	3721	2335					Not Found
	3391	3158	3409	2519					Not Found
					3642	3392	3696	3167	Not Found
	4531	5328	4536	4128					Not Found
					5486	4934	6135	4772	Not Found
	1584	1047	1258	1171					Not Found
	2245	1920	2013	1963					Not Found
	2870	3137	2820	2749					Not Found
									High
	2849	3078	2420	2274					Not Found
2178									High
2057									High
									Not Found
					4692	2996	3924	3959	Not Found
	4382	2584	2914	2772					Not Found
					4786	5175	4401	4375	Not Found
626									High
3754									High
									Not Found
					7801	5005	5146	4560	Not Found
1706									High
3753									High
									Not Found
1519									High
					2579	2480	2804	2498	Not Found

					3944	3039	3626	3022	Not Found
	1080	1037	857	939					Not Found
1908									High
					649	845	710	531	Not Found
	2396	2676	2703	2327					Not Found
									Not Found
									Not Found
									Not Found
					2982	3777	3116	2654	Not Found
					5050	4058	3776	3926	Not Found
1195									High
	2034	2392	2356	2008					Not Found
735									High
					3753	3552	3315	2872	Not Found
6738									High
									Not Found
					677	1054	842	1013	Not Found
					4247	4086	3342	3742	Not Found
2750									High
					1438	1291	1348	1298	Not Found
									Not Found
	5067	4860	6750	4903					Not Found
	6626	6520	6330	6715					Not Found
									Not Found
4783									High
					3975	2039	3404	2832	Not Found
					2771	2516	2548	2207	Not Found
									Not Found
					2634	2584	2873	2496	Not Found
									High
									Not Found
									Not Found
	4273	3880	4402	4194					Not Found
									Not Found
1073									High
					3917	3518	5495	3167	Not Found
					2635	3691	3146	2387	Not Found
					3156	3021	3647	3239	Not Found
1649									High
	1811	1909	1598	1537					Not Found
	7022	8170	8178	6280					Not Found
3820									High
	1025	651	880	747					Not Found
579									High
5918									High
	5314	4522	3822	3658					Not Found
	1288	1256	1183	1089					Not Found
	5441	4973	4174	5038					Not Found
4242									High
					2253	3665	2209	2950	Not Found

	794	779	887	753					Not Found
									Not Found
									Not Found
4084									High
									Not Found
					2996	2420	2491	2186	Not Found
	923	716	770	641					Not Found
									Not Found
					3880	4098	3980	4522	Not Found
	4048	3288	3741	3288					Not Found
					1444	1444	2887	1533	Not Found
	1224	1393	1359	1171					Not Found
					6455	6102	5750	6181	Not Found
	2323	2091	4384	2080					Not Found
	4857	5437	5500	4338					Not Found
					1795	1467	1533	1366	Not Found
					3145	2988	3433	2633	Not Found
	6121	6294	5274	5208					Not Found
2774									High
									Not Found
	4482	3524	3583	3606					Not Found
									Not Found
	598	468	490	494					Not Found
									Not Found
									Not Found
					2734	2785	2924	2547	Not Found
4929									High
									Not Found
									Not Found
3647									High
911									High
2369									High
									High
									Not Found
					2595	2048	2552	1960	Not Found
2738									High
	3722	4799	3888	3451					Not Found
					4765	4326	4920	4089	Not Found
	8528	6739	8549	6422					Not Found
	2589	3141	2785	2222					Not Found
3481									High
1356									High
2808									High
2996									High
275									High
					3736	4911	3883	3887	Not Found
									Not Found
4462									High
	3306	2963	3910	3335					Not Found
					1730	1485	1643	1391	Not Found

					2923	2246	2739	2125	Not Found
	8473	6454	6542	6629					Not Found
					1806	1702	1690	1314	Not Found
					1420	1828	1387	1214	Not Found
	3512	3359	3497	2864					Not Found
	4991	2435	2813	3426					Not Found
	1087	1524	1253	1390					Not Found
3692									High
3367									High
					1645	1409	1358	1478	Not Found
1240									High
	4056	4176	4616	3434					Not Found
	4556	4151	3725	3626					Not Found
1258									High
	1990	2052	1840	1477					Not Found
					2199	2185	2021	1947	Not Found
	3236	3247	2832	2583					Not Found
									Not Found
									Not Found
					6370	5831	6264	6500	Not Found
					1514	1464	1578	1351	Not Found
2192									High
					3880	2995	3177	3097	Not Found
1201									High
1321									High
1286									High
									Not Found
									Not Found
									Not Found
5519									High
1660									High
									Not Found
									Not Found
									Not Found
									Not Found
2484									High
	10101	5583	4697	5243					Not Found
									Not Found
					2593	3193	4182	2594	Not Found
									Not Found
	1410	1033	1273	1325					Not Found
6036									High
					3750	7509	3631	3608	Not Found
1166									High
	2042	1715	1866	1799					Not Found
886									High
	501	485	315	415					Not Found
									Not Found
327									High
					4678	3737	3939	3558	Not Found

									Not Found
									Not Found
					1584	1189	1807	1683	Not Found
					1840	1764	1886	2123	Not Found
					2613	2050	2541	2316	Not Found
									Not Found
	2588	2754	2715	2506					Not Found
									Not Found
1723									High
6477									High
	5819	4248	4868	3992					Not Found
	7259	6562	6016	6808					Not Found
					2788	3490	2843	2300	Not Found
2356									High
									Not Found
									Not Found
									Not Found
	4205	3917	4321	3544					Not Found
1328									High
3072									High
									High
									Not Found
									Not Found
									Not Found
					1080	1004	974	830	Not Found
	2770	2473	2184	1953					Not Found
1578									High
					2936	3583	2749	3116	Not Found
	5948	1603	4548	1700					Not Found
	5548	4730	5207	4612					Not Found
					5655	5450	5028	5827	Not Found
					1757	1411	2911	1894	Not Found
474									High
									Not Found
									Not Found
									Not Found
									Not Found
									Not Found
					5215	3022	4077	4087	Not Found
	2916	2879	3435	2693					Not Found
									Not Found
	4375	4365	4114	4062					Not Found
	1521	1473	1579	1286					Not Found
									Not Found
					2432	2265	2508	1951	Not Found
1593									High
					2693	2676	3309	3071	Not Found
	2094	2191	2533	1738					Not Found
									Not Found
2752									High

									Not Found
594									High
1374									High
3148									High
									Not Found
	1660	1522	1461	1434					Not Found
	3323	2544	2585	2769					Not Found
2212									High
1832									High
	5136	6421	4724	4578					Not Found
6880									High
5195									High
					4738	3620	3753	3343	Not Found
					1283	1069	958	934	Not Found
2624									High
1550									High
	5426	4664	4542	4243					Not Found
3366									High
	2327	1673	2065	1892					Not Found
	5448	6567	5075	3908					Not Found
3629									High
2881									High
1696									High
	3652	2949	2882	3065					Not Found
3818									High
	1350	1089	1275	939					Not Found
4157									High
	3029	3713	3723	3260					Not Found
	2077	1807	2164	1582					Not Found
2352									High
	3867	2972	3476	3063					Not Found
	1459	1247	1525	1315					Not Found
	750	901	975	850					Not Found
					3683	2270	2935	2619	Not Found
2395									High
1261									High
2971									High
	7055	5826	6885	5203					Not Found
									Not Found
1632									High
3721									High
									Not Found
	3317	2108	3007	2566					Not Found
									Not Found
					6371	4934	4612	4335	Not Found
	798	994	796	950					Not Found
	11655	8333	8038	8056					Not Found
					1067	872	1087	805	Not Found
					5112	4676	4662	3505	Not Found
					1807	625	1827	757	Not Found

	7173	5899	6172	6853					Not Found
1597									High
					4050	3307	3629	3187	Not Found
					3569	2979	3152	2684	Not Found
	4654	3545	3597	2986					Not Found
	2906	2207	2937	2709					Not Found
1389									High
					3648	2846	2845	2415	Not Found
	1742	1439	1630	1257					Not Found
2258									High
	3784	4033	3110	3539					Not Found
6270									High
2136									High
	2024	1477	1569	1446					Not Found
1613									High
					1339	1627	1183	1475	Not Found
	4376	3403	3429	3370					Not Found
	1567	1262	1429	1068					Not Found
3481									High
					2810	2491	2410	2556	Not Found
2110									High
2675									High
	324	284	251	297					Not Found
					3371	1951	2331	1932	Not Found
3400									High
2440									High
					6201	5641	5491	6291	Not Found
2161									High
					4505	3810	3818	3750	Not Found
					2944	2298	2846	2305	Not Found
1640									High
	1167	793	926	775					Not Found
	3788	3161	4563	3529					Not Found
149									High
2361									High
	5309	4428	3676	3881					Not Found
	5516	4746	5119	3756					Not Found
3584									High
					3020	2625	2655	2556	Not Found
	3596	3122	2660	3231					Not Found
									Not Found
1121									High
9681									High
					3656	3064	2539	3082	Not Found
6175									High
2714									High
	2409	2187	2194	1900					Not Found
	389	452	328	371					Not Found
1884									High
	3509	2664	2954	2711					Not Found

	7491	8904	7980	6845					Not Found
	6647	4351	5115	4145					Not Found
2157									High
					5917	5004	5121	5063	Not Found
	6984	5126	5115	4590					Not Found
3110									High
									Not Found
2086									High
					3897	3435	3237	3634	Not Found
	4224	3478	2854	3222					Not Found
1935									High
3807									High
									High
					1772	2073	1740	1511	Not Found
									Not Found
3082									High
									Not Found
					927	995	998	772	Not Found
									Not Found
									Not Found
									High
									Not Found
					5048	4815	4328	3525	Not Found
									Not Found
	4855	3204	4750	3675					Not Found
3806									High
					1479	1396	1465	1398	Not Found
	4524	3513	4386	3523					Not Found
					2136	1607	1822	1447	Not Found
	4443	4998	3948	3732					Not Found
3468									High
	2951	2211	2590	2954					Not Found
2061									High
	3330	2949	2937	2793					Not Found
	2590	2238	2908	1819					Not Found
					2166	1605	1637	1946	Not Found
					3247	3185	3023	2437	Not Found
					4482	2873	5461	3946	Not Found
	2237	1681	2121	1889					Not Found
2039									High
					13158	5937	11000	10040	Not Found
2509									High
	2824	2350	7322	2737					Not Found
	4409	4572	6077	3666					Not Found
					4424	3601	4017	3330	Not Found
	1694	1402	1549	1341					Not Found
379									High
									Not Found
									Not Found
					3152	2299	2822	2290	Not Found

1795									High
					3755	3232	3869	3847	Not Found
3016									High
									Not Found
					4397	3234	3798	2984	Not Found
					3413	2170	2731	3202	Not Found
					4983	2801	3868	3024	Not Found
2138									High
	3200	2549	2598	2188					Not Found
	515	291	557	469					Not Found
	3666	2908	3675	2629					Not Found
	1034	1118	1647	1781					Not Found
	3443	2827	3154	3179					Not Found
2056									High
2676									High
									Not Found
	5297	4633	4563	3930					Not Found
									High
7682									High
	2468	2187	2487	2198					Not Found
					3523	3225	3158	3284	Not Found
	2129	3575	2553	3049					Not Found
					3107	3333	3285	3068	Not Found
					5556	4096	4265	4684	Not Found
	4728	3934	4266	3869					Not Found
	6270	5732	4367	5429					Not Found
					3156	3018	5953	2995	Not Found
	4463	4014	3921	3570					Not Found
6148									High
									Not Found
					5668	4069	4571	4404	Not Found
	4030	1856	2938	2925					Not Found
	3451	3886	3192	3002					Not Found
					5843	4702	3941	5119	Not Found
	3060	3522	2786	3953					Not Found
	3353	8156	5258	2954					Not Found
									Not Found
					4581	4294	3821	3888	Not Found
2729									High
									Not Found
1763									High
									Not Found
					5616	5899	4580	4112	Not Found
5917									High
									Not Found
1602									High
	3187	4149	3262	3719					Not Found
									Not Found
					4017	3209	3603	3520	Not Found
					3429	2863	5054	4023	Not Found

									Not Found
1434									High
4375									High
									Not Found
									Not Found
	1189	1354	1199	1056					Not Found
	2758	2561	3121	2809					Not Found
2198									High
					1963	1277	1749	1767	Not Found
	3013	2703	2899	2313					Not Found
4132									High
									High
					6523	6240	6014	5150	Not Found
3333									High
	2138	1906	1985	1732					Not Found
3380									High
									High
3069									High
					7633	6449	8148	5949	Not Found
	4323	3240	3209	3930					Not Found
									High
	5749	4583	3979	6388					Not Found
	4214	4747	3714	3434					Not Found
									Not Found
1191									High
	1387	1077	1327	1080					Not Found
3570									High
	7696	6992	6619	6837					Not Found
					8040	6343	5907	5115	Not Found
	2320	2115	2298	2073					Not Found
					3550	3299	3221	3615	Not Found
					4031	2415	3557	2558	Not Found
					2808	1545	1748	1752	Not Found
2929									High
	6017	3204	3723	4586					Not Found
					3289	3889	3725	2852	Not Found
					1099	1347	1263	866	Not Found
	2119	2732	2491	1901					Not Found
									High
									Not Found
									Not Found
									Not Found
									Not Found
									High
	3171	2935	2904	2618					Not Found
	4589	4239	4877	3681					Not Found
									High
									Not Found
1904									High
	5414	4169	4107	4004					Not Found

	3393	4246	3969	3377					Not Found
4332									High
					1982	2255	5504	1521	Not Found
2651									High
	4139	3131	3578	3282					Not Found
2550									High
									High
	4490	7922	6384	5399					Not Found
	3625	3332	3546	3174					Not Found
4790									High
2559									High
					2344	2208	2747	2404	Not Found
2785									High
2136									High
					3437	3570	3839	3165	Not Found
	3166	2963	3515	2720					Not Found
5823									High
					1451	865	1516	939	Not Found
	6090	4458	5062	4484					Not Found
6576									High
	6241	3435	4023	4161					Not Found
					6336	5284	5630	5422	Not Found
2199									High
					6302	5616	4511	6846	Not Found
5501									High
	6856	5878	5974	5614					Not Found
6743									High
	6291	4867	5166	4163					Not Found
									Not Found
7455									High
703									High
					4847	3915	4091	3911	Not Found
					2233	2927	2749	2219	Not Found
					4978	4104	4870	4265	Not Found
	5565	3490	4588	4027					Not Found
					6383	5583	4537	5382	Not Found
					3416	2909	2875	3085	Not Found
									Not Found
					6148	4957	5861	4991	Not Found
									Not Found
					2432	2181	2491	3074	Not Found
					5624	2802	4508	3850	Not Found
					3879	2804	3281	3165	Not Found
	2916	3705	4391	3068					Not Found
	2696	2867	3193	2459					Not Found
									Not Found
					3996	2941	3874	3170	Not Found
	7974	6268	6485	6502					Not Found
3888									High
1341									High

									Not Found
3827									High
1383									High
3227									High
2169									High
									Not Found
									High
									High
					2364	3293	2746	2228	Not Found
	4216	3580	3711	3487					Not Found
	4507	3071	3495	4109					Not Found
									Not Found
					3986	3348	4009	3228	Not Found
					1612	1003	1249	967	Not Found
					6467	4847	5761	4988	Not Found
	4335	3208	3873	3939					Not Found
									Not Found
4525									High
	1878	1760	2422	2972					Not Found
									Not Found
					4268	3491	5227	4412	Not Found
									High
					1183	1422	1512	1512	Not Found
					5282	2788	5041	4238	Not Found
					2657	1469	1769	2079	Not Found
					8562	5303	9891	6130	Not Found
					2879	1964	2634	2565	Not Found
					2502	1883	2237	2001	Not Found
									High
6947									High
5083									High
					2349	2021	2164	1967	Not Found
	3990	5119	4918	3368					Not Found
	3612	3883	5527	3443					Not Found
					2531	1468	2375	1656	Not Found
					4666	3768	4432	3988	Not Found
2420									High
									High
					4363	3806	3429	3429	Not Found
									Not Found
3101									High
1963									High
	676	731	622	688					Not Found
	1396	1650	1597	1474					Not Found
	4670	5786	4894	5402					Not Found
	6807	4637	6325	4399					Not Found
									Not Found
					2231	1703	2459	1961	Not Found
					1744	1297	1644	1476	Not Found
									High

					1703	1719	1540	1825	Not Found
	5831	5287	6175	4528					Not Found
									High
4950									High
									Not Found
3658									High
					2330	1573	1898	1565	Not Found
					3591	2838	3006	2560	Not Found
									High
	2508	2069	2655	2105					Not Found
	2126	1981	2277	1762					Not Found
					2508	2557	2320	1718	Not Found
3418									High
					4704	4379	4008	3387	Not Found
2852									High
3264									High
					3710	6189	3887	3310	Not Found
	2978	3240	3794	2704					Not Found
1307									High
2261									High
2535									High
	7899	6877	6981	7042					Not Found
	1930	2019	2284	1675					Not Found
	1093	794	1073	736					Not Found
									Not Found
2444									High
	1237	1029	1028	1026					Not Found
637									High
	2682	2295	2503	2196					Not Found
									Not Found
					2131	2196	3008	2297	Not Found
									Not Found
3236									High
					10064	5996	11107	8780	Not Found
2411									High
									Not Found
	4952	4766	5428	5181					Not Found
									High
	2677	2879	3348	2447					Not Found
									Not Found
									Not Found
	4225	3159	4053	2901					Not Found
	4025	4026	7011	3333					Not Found
	3585	3466	4323	3362					Not Found
	3369	3708	3361	2427					Not Found
3483									High
2997									High
					5857	6363	7492	6769	Not Found
					5576	5471	5695	5303	Not Found
					3241	2663	3380	2728	Not Found

	3500	3303	3510	2858					Not Found
	4708	4358	4035	3533					Not Found
									High
	4138	3880	4138	3407					Not Found
1624									High
	2913	2981	3904	3159					Not Found
3500									High
					3362	2212	2519	2396	Not Found
					2916	2553	2810	2301	Not Found
3244									High
					1964	1555	1619	1809	Not Found
2595									High
					2905	2171	9989	3282	Not Found
									Not Found
									High
					1914	1334	1508	1271	Not Found
	3351	2224	2069	2351					Not Found

Found in Sa	Found in Sa	Found in Sa	Found in Sa	Found in Sa	Found in Sa	Found in Sa	Found in Sa	Found in Sa	Found in Sa
High	High	High	High	High	High	High	High	High	High
High	High	High	High	High	High	High	High	High	High
High	High	High	High	High	High	High	High	High	High
High	High	High	High	High	High	High	High	High	High
High	High	High	High	High	High	High	High	High	High
High	High	High	High	High	High	High	High	High	High
High	High	High	High	High	High	High	High	High	High
High	High	High	High	High	High	High	High	High	High
High	High	High	High	High	High	High	High	High	High
High	High	High	High	High	High	High	High	High	High
High	High	High	High	High	High	High	High	High	High
High	High	High	High	High	High	High	High	High	High
High	High	High	High	High	High	High	High	High	High
High	High	High	High	High	High	High	High	High	High
Not Found	Not Found	Not Found	Not Found	Not Found	Not Found	Not Found	High	High	High
High	High	High	High	High	High	High	High	High	High
High	High	High	High	High	High	High	High	High	High
High	High	High	High	High	High	High	High	High	High
High	High	High	High	High	High	High	High	High	High
High	High	High	High	High	High	High	High	High	High
Not Found	Not Found	Not Found	High	High	High	High	High	High	High
High	High	High	Not Found	Not Found	Not Found	Not Found	Not Found	Not Found	Not Found
High	High	High	High	High	High	High	High	High	High
High	High	High	High	High	High	High	High	High	High
High	High	High	High	High	High	High	High	High	High
High	High	High	High	High	High	High	High	High	High
High	High	High	High	High	High	High	High	High	High
High	High	High	High	High	High	High	High	High	High
Not Found	Not Found	Not Found	High	High	High	High	Not Found	Not Found	Not Found
High	High	High	High	High	High	High	High	High	High
High	High	High	High	High	High	High	High	High	High
High	High	High	High	High	High	High	High	High	High
High	High	High	High	High	High	High	High	High	High
High	High	High	High	High	High	High	High	High	High
High	High	High	High	High	High	High	High	High	High
High	High	High	High	High	High	High	High	High	High
High	High	High	High	High	High	High	High	High	High
High	High	High	High	High	High	High	High	High	High
High	High	High	High	High	High	High	High	High	High
High	High	High	High	High	High	High	High	High	High
High	High	High	High	High	High	High	High	High	High
High	High	High	High	High	High	High	High	High	High
High	High	High	High	High	High	High	High	High	High
High	High	High	High	High	High	High	High	High	High
High	High	High	High	High	High	High	High	High	High
High	High	High	High	High	High	High	High	High	High
High	High	High	High	High	High	High	High	High	High
High	High	High	High	High	High	High	High	High	High
High	High	High	High	High	High	High	High	High	High

High	High	High	High	High	High	High	High	High	High
High	High	High	High	High	High	High	High	High	High
High	High	High	High	High	High	High	High	High	High
High	High	High	High	High	High	High	High	High	High
High	High	High	High	High	High	High	High	High	High
High	High	High	High	High	High	High	High	High	High
High	High	High	High	High	High	High	High	High	High
High	High	High	High	High	High	High	High	High	High
High	High	High	High	High	High	High	High	High	High
High	High	High	High	High	High	High	High	High	High
High	High	High	High	High	High	High	High	High	High
High	High	High	High	High	High	High	High	High	High
High	High	High	High	High	High	High	High	High	High
High	High	High	High	High	High	High	High	High	High
High	High	High	High	High	High	High	High	High	High
High	High	High	High	High	High	High	Not Found	Not Found	Not Found
High	High	High	High	High	High	High	High	High	High
High	High	High	High	High	High	High	High	High	High
High	High	High	High	High	High	High	High	High	High
High	High	High	High	High	High	High	High	High	High
High	High	High	High	High	High	High	High	High	High
High	High	High	High	High	High	High	High	High	High
High	High	High	High	High	High	High	High	High	High
High	High	High	High	High	High	High	High	High	High
High	High	High	High	High	High	High	High	High	High
High	High	High	High	High	High	High	High	High	High
Not Found	Not Found	Not Found	High	High	High	High	High	High	High
High	High	High	High	High	High	High	High	High	High
High	High	High	High	High	High	High	High	High	High
High	High	High	High	High	High	High	High	High	High
High	High	High	High	High	High	High	High	High	High
High	High	High	High	High	High	High	High	High	High
High	High	High	High	High	High	High	High	High	High
High	High	High	High	High	High	High	High	High	High
High	High	High	High	High	High	High	High	High	High
High	High	High	High	High	High	High	High	High	High
High	High	High	High	High	High	High	High	High	High
High	High	High	High	High	High	High	High	High	High
High	High	High	High	High	High	High	High	High	High
High	High	High	High	High	High	High	High	High	High
High	High	High	High	High	High	High	High	High	High
High	High	High	High	High	High	High	High	High	High
Not Found	Not Found	Not Found	High	High	High	High	High	High	High
High	High	High	High	High	High	High	High	High	High
High	High	High	High	High	High	High	High	High	High
High	High	High	High	High	High	High	High	High	High
Not Found	Not Found	Not Found	Not Found	Not Found	Not Found	Not Found	High	High	High
High	High	High	High	High	High	High	High	High	High
High	High	High	High	High	High	High	High	High	High
High	High	High	High	High	High	High	High	High	High

High	High	High	High	High	High	High	High	High	High
High	High	High	High	High	High	High	High	High	High
High	High	High	High	High	High	High	High	High	High
High	High	High	High	High	High	High	High	High	High
High	High	High	High	High	High	High	High	High	High
Not Found	Not Found	Not Found	High	High	High	High	High	High	High
High	High	High	High	High	High	High	High	High	High
High	High	High	High	High	High	High	High	High	High
High	High	High	High	High	High	High	High	High	High
High	High	High	Not Found	Not Found	Not Found	Not Found	High	High	High
High	High	High	High	High	High	High	High	High	High
High	High	High	High	High	High	High	High	High	High
High	High	High	High	High	High	High	High	High	High
High	High	High	High	High	High	High	High	High	High
High	High	High	High	High	High	High	High	High	High
High	High	High	High	High	High	High	High	High	High
High	High	High	High	High	High	High	High	High	High
Not Found	Not Found	Not Found	Not Found	Not Found	Not Found	Not Found	Not Found	Not Found	Not Found
High	High	High	High	High	High	High	High	High	High
High	High	High	High	High	High	High	High	High	High
High	High	High	High	High	High	High	High	High	High
High	High	High	High	High	High	High	High	High	High
High	High	High	High	High	High	High	High	High	High
High	High	High	High	High	High	High	High	High	High
High	High	High	High	High	High	High	High	High	High
High	High	High	High	High	High	High	High	High	High
High	High	High	High	High	High	High	High	High	High
Not Found	Not Found	Not Found	High	High	High	High	High	High	High
High	High	High	Not Found	Not Found	Not Found	Not Found	Not Found	Not Found	Not Found
High	High	High	High	High	High	High	High	High	High
High	High	High	High	High	High	High	High	High	High
High	High	High	High	High	High	High	High	High	High
High	High	High	High	High	High	High	High	High	High
High	High	High	High	High	High	High	High	High	High
High	High	High	High	High	High	High	High	High	High
High	High	High	High	High	High	High	High	High	High
High	High	High	High	High	High	High	High	High	High
High	High	High	High	High	High	High	High	High	High
High	High	High	High	High	High	High	High	High	High
High	High	High	High	High	High	High	High	High	High
High	High	High	High	High	High	High	High	High	High
Not Found	Not Found	Not Found	High	High	High	High	Not Found	Not Found	Not Found
High	High	High	High	High	High	High	High	High	High
High	High	High	High	High	High	High	High	High	High
High	High	High	High	High	High	High	High	High	High
High	High	High	High	High	High	High	High	High	High
High	High	High	High	High	High	High	High	High	High
High	High	High	High	High	High	High	High	High	High
Not Found	Not Found	Not Found	High	High	High	High	High	High	High
High	High	High	High	High	High	High	High	High	High

High	High	High	High	High	High	High	High	High	High
High	High	High	High	High	High	High	High	High	High
High	High	High	High	High	High	High	High	High	High
High	High	High	High	High	High	High	High	High	High
High	High	High	High	High	High	High	High	High	High
High	High	High	High	High	High	High	Not Found	Not Found	Not Found
High	High	High	High	High	High	High	High	High	High
High	High	High	High	High	High	High	High	High	High
High	High	High	High	High	High	High	High	High	High
High	High	High	High	High	High	High	High	High	High
High	High	High	High	High	High	High	High	High	High
High	High	High	High	High	High	High	High	High	High
High	High	High	High	High	High	High	High	High	High
High	High	High	High	High	High	High	High	High	High
Not Found	Not Found	Not Found	High	High	High	High	Not Found	Not Found	Not Found
High	High	High	High	High	High	High	High	High	High
High	High	High	High	High	High	High	High	High	High
High	High	High	High	High	High	High	High	High	High
High	High	High	High	High	High	High	High	High	High
High	High	High	High	High	High	High	High	High	High
High	High	High	High	High	High	High	High	High	High
High	High	High	High	High	High	High	High	High	High
High	High	High	High	High	High	High	High	High	High
Not Found	Not Found	Not Found	High	High	High	High	High	High	High
High	High	High	High	High	High	High	High	High	High
High	High	High	Not Found	Not Found	Not Found	Not Found	High	High	High
High	High	High	High	High	High	High	High	High	High
High	High	High	High	High	High	High	High	High	High
High	High	High	High	High	High	High	High	High	High
High	High	High	High	High	High	High	High	High	High
High	High	High	High	High	High	High	High	High	High
High	High	High	High	High	High	High	High	High	High
High	High	High	High	High	High	High	High	High	High
High	High	High	High	High	High	High	High	High	High
High	High	High	High	High	High	High	High	High	High
High	High	High	High	High	High	High	High	High	High
High	High	High	High	High	High	High	High	High	High
Not Found	Not Found	Not Found	High	High	High	High	High	High	High
High	High	High	High	High	High	High	High	High	High
High	High	High	High	High	High	High	High	High	High
High	High	High	High	High	High	High	High	High	High
High	High	High	High	High	High	High	High	High	High
High	High	High	High	High	High	High	High	High	High
High	High	High	Not Found	Not Found	Not Found	Not Found	Not Found	Not Found	Not Found
High	High	High	High	High	High	High	High	High	High
High	High	High	High	High	High	High	High	High	High
High	High	High	High	High	High	High	High	High	High
High	High	High	High	High	High	High	High	High	High
High	High	High	High	High	High	High	High	High	High
High	High	High	High	High	High	High	High	High	High

High	High	High	High	High	High	High	High	High	High
High	High	High	High	High	High	High	High	High	High
High	High	High	High	High	High	High	High	High	High
High	High	High	High	High	High	High	High	High	High
High	High	High	High	High	High	High	High	High	High
High	High	High	High	High	High	High	High	High	High
High	High	High	High	High	High	High	High	High	High
High	High	High	High	High	High	High	High	High	High
High	High	High	High	High	High	High	High	High	High
High	High	High	High	High	High	High	High	High	High
High	High	High	High	High	High	High	High	High	High
High	High	High	High	High	High	High	High	High	High
Not Found	Not Found	Not Found	High	High	High	High	High	High	High
High	High	High	High	High	High	High	High	High	High
High	High	High	High	High	High	High	High	High	High
High	High	High	High	High	High	High	High	High	High
High	High	High	High	High	High	High	Not Found	Not Found	Not Found
High	High	High	High	High	High	High	High	High	High
High	High	High	High	High	High	High	High	High	High
High	High	High	High	High	High	High	High	High	High
High	High	High	High	High	High	High	High	High	High
High	High	High	High	High	High	High	High	High	High
High	High	High	High	High	High	High	High	High	High
High	High	High	High	High	High	High	High	High	High
High	High	High	High	High	High	High	High	High	High
High	High	High	High	High	High	High	High	High	High
High	High	High	High	High	High	High	High	High	High
High	High	High	High	High	High	High	High	High	High
High	High	High	High	High	High	High	High	High	High
High	High	High	High	High	High	High	High	High	High
High	High	High	High	High	High	High	High	High	High
High	High	High	High	High	High	High	High	High	High
High	High	High	High	High	High	High	High	High	High
High	High	High	High	High	High	High	High	High	High
High	High	High	High	High	High	High	High	High	High
High	High	High	High	High	High	High	High	High	High
High	High	High	High	High	High	High	High	High	High
High	High	High	High	High	High	High	High	High	High
High	High	High	High	High	High	High	Not Found	Not Found	Not Found
High	High	High	High	High	High	High	High	High	High
Not Found	Not Found	Not Found	High	High	High	High	Not Found	Not Found	Not Found
High	High	High	High	High	High	High	High	High	High
High	High	High	High	High	High	High	High	High	High
High	High	High	High	High	High	High	High	High	High
High	High	High	High	High	High	High	High	High	High
High	High	High	High	High	High	High	High	High	High
High	High	High	High	High	High	High	High	High	High
High	High	High	High	High	High	High	High	High	High
High	High	High	High	High	High	High	High	High	High
High	High	High	High	High	High	High	High	High	High

Not Found	Not Found	Not Found	Not Found	Not Found	Not Found	Not Found	High	High	High
High	High	High	High	High	High	High	High	High	High
High	High	High	High	High	High	High	High	High	High
High	High	High	High	High	High	High	High	High	High
High	High	High	High	High	High	High	High	High	High
High	High	High	High	High	High	High	High	High	High
High	High	High	High	High	High	High	High	High	High
High	High	High	High	High	High	High	High	High	High
High	High	High	High	High	High	High	High	High	High
High	High	High	High	High	High	High	High	High	High
High	High	High	High	High	High	High	High	High	High
High	High	High	High	High	High	High	High	High	High
High	High	High	High	High	High	High	High	High	High
High	High	High	High	High	High	High	High	High	High
High	High	High	Not Found	Not Found	Not Found	Not Found	High	High	High
High	High	High	High	High	High	High	High	High	High
High	High	High	High	High	High	High	High	High	High
High	High	High	High	High	High	High	High	High	High
High	High	High	High	High	High	High	High	High	High
High	High	High	Not Found	Not Found	Not Found	Not Found	Not Found	Not Found	Not Found
High	High	High	High	High	High	High	High	High	High
High	High	High	High	High	High	High	High	High	High
High	High	High	High	High	High	High	Not Found	Not Found	Not Found
High	High	High	High	High	High	High	High	High	High
High	High	High	High	High	High	High	High	High	High
High	High	High	High	High	High	High	High	High	High
High	High	High	High	High	High	High	High	High	High
High	High	High	High	High	High	High	High	High	High
High	High	High	High	High	High	High	High	High	High
High	High	High	High	High	High	High	High	High	High
High	High	High	High	High	High	High	High	High	High
High	High	High	High	High	High	High	High	High	High
High	High	High	High	High	High	High	High	High	High
High	High	High	High	High	High	High	High	High	High
High	High	High	High	High	High	High	High	High	High
High	High	High	High	High	High	High	High	High	High
High	High	High	High	High	High	High	High	High	High
High	High	High	High	High	High	High	High	High	High
High	High	High	High	High	High	High	High	High	High
High	High	High	High	High	High	High	High	High	High
High	High	High	High	High	High	High	High	High	High
High	High	High	High	High	High	High	High	High	High
High	High	High	High	High	High	High	High	High	High
High	High	High	High	High	High	High	High	High	High
High	High	High	High	High	High	High	High	High	High
High	High	High	High	High	High	High	High	High	High
High	High	High	Not Found	Not Found	Not Found	Not Found	High	High	High
High	High	High	High	High	High	High	High	High	High
High	High	High	High	High	High	High	High	High	High
High	High	High	High	High	High	High	High	High	High

High	High	High	Not Found	Not Found	Not Found	Not Found	High	High	High
High	High	High	High	High	High	High	High	High	High
High	High	High	High	High	High	High	High	High	High
High	High	High	High	High	High	High	High	High	High
High	High	High	High	High	High	High	High	High	High
High	High	High	High	High	High	High	High	High	High
High	High	High	High	High	High	High	High	High	High
High	High	High	High	High	High	High	High	High	High
High	High	High	High	High	High	High	High	High	High
High	High	High	High	High	High	High	High	High	High
High	High	High	High	High	High	High	High	High	High
High	High	High	High	High	High	High	High	High	High
High	High	High	High	High	High	High	High	High	High
Not Found	Not Found	Not Found	High	High	High	High	High	High	High
High	High	High	High	High	High	High	High	High	High
High	High	High	High	High	High	High	High	High	High
High	High	High	High	High	High	High	High	High	High
High	High	High	High	High	High	High	High	High	High
High	High	High	High	High	High	High	High	High	High
High	High	High	High	High	High	High	High	High	High
High	High	High	High	High	High	High	High	High	High
High	High	High	High	High	High	High	High	High	High
High	High	High	High	High	High	High	High	High	High
High	High	High	High	High	High	High	High	High	High
High	High	High	High	High	High	High	High	High	High
High	High	High	High	High	High	High	High	High	High
High	High	High	High	High	High	High	High	High	High
High	High	High	High	High	High	High	High	High	High
High	High	High	High	High	High	High	High	High	High
High	High	High	High	High	High	High	High	High	High
High	High	High	High	High	High	High	High	High	High
High	High	High	High	High	High	High	High	High	High
High	High	High	High	High	High	High	High	High	High
High	High	High	High	High	High	High	High	High	High
High	High	High	High	High	High	High	High	High	High
High	High	High	High	High	High	High	High	High	High
High	High	High	High	High	High	High	High	High	High
High	High	High	High	High	High	High	High	High	High
High	High	High	High	High	High	High	High	High	High
High	High	High	High	High	High	High	High	High	High
High	High	High	High	High	High	High	High	High	High
High	High	High	High	High	High	High	High	High	High
High	High	High	High	High	High	High	High	High	High
High	High	High	High	High	High	High	High	High	High
High	High	High	High	High	High	High	High	High	High
High	High	High	High	High	High	High	High	High	High
High	High	High	High	High	High	High	High	High	High
High	High	High	High	High	High	High	High	High	High
High	High	High	High	High	High	High	High	High	High
High	High	High	High	High	High	High	High	High	High

High	High	High	High	High	High	High	High	High	High
High	High	High	High	High	High	High	High	High	High
High	High	High	High	High	High	High	High	High	High
High	High	High	High	High	High	High	High	High	High
High	High	High	High	High	High	High	High	High	High
Not Found	Not Found	Not Found	Not Found	Not Found	Not Found	Not Found	High	High	High
High	High	High	High	High	High	High	High	High	High
High	High	High	High	High	High	High	High	High	High
High	High	High	High	High	High	High	High	High	High
High	High	High	High	High	High	High	High	High	High
High	High	High	High	High	High	High	High	High	High
High	High	High	High	High	High	High	High	High	High
High	High	High	High	High	High	High	High	High	High
High	High	High	High	High	High	High	High	High	High
High	High	High	High	High	High	High	High	High	High
High	High	High	High	High	High	High	High	High	High
High	High	High	High	High	High	High	High	High	High
High	High	High	High	High	High	High	High	High	High
High	High	High	High	High	High	High	High	High	High
High	High	High	High	High	High	High	High	High	High
High	High	High	High	High	High	High	High	High	High
High	High	High	High	High	High	High	High	High	High
High	High	High	High	High	High	High	High	High	High
High	High	High	High	High	High	High	High	High	High
High	High	High	High	High	High	High	High	High	High
High	High	High	High	High	High	High	High	High	High
High	High	High	High	High	High	High	High	High	High
High	High	High	High	High	High	High	High	High	High
High	High	High	High	High	High	High	High	High	High
High	High	High	High	High	High	High	High	High	High
High	High	High	High	High	High	High	High	High	High
High	High	High	High	High	High	High	High	High	High
High	High	High	High	High	High	High	High	High	High
High	High	High	High	High	High	High	High	High	High
High	High	High	High	High	High	High	High	High	High
High	High	High	High	High	High	High	High	High	High
High	High	High	High	High	High	High	High	High	High
High	High	High	High	High	High	High	High	High	High
High	High	High	High	High	High	High	High	High	High
High	High	High	High	High	High	High	High	High	High
High	High	High	High	High	High	High	High	High	High
High	High	High	High	High	High	High	High	High	High
High	High	High	High	High	High	High	High	High	High
High	High	High	High	High	High	High	High	High	High
High	High	High	High	High	High	High	High	High	High
High	High	High	High	High	High	High	High	High	High
High	High	High	Not Found	Not Found	Not Found	Not Found	Not Found	Not Found	Not Found
High	High	High	High	High	High	High	High	High	High
High	High	High	High	High	High	High	High	High	High
High	High	High	High	High	High	High	High	High	High

High	High	High	High	High	High	High	High	High	High
High	High	High	High	High	High	High	High	High	High
High	High	High	High	High	High	High	High	High	High
High	High	High	High	High	High	High	High	High	High
High	High	High	High	High	High	High	High	High	High
High	High	High	High	High	High	High	High	High	High
High	High	High	High	High	High	High	High	High	High
High	High	High	High	High	High	High	Not Found	Not Found	Not Found
High	High	High	High	High	High	High	Not Found	Not Found	Not Found
High	High	High	High	High	High	High	High	High	High
High	High	High	High	High	High	High	High	High	High
High	High	High	High	High	High	High	High	High	High
High	High	High	High	High	High	High	High	High	High
High	High	High	High	High	High	High	High	High	High
High	High	High	High	High	High	High	High	High	High
High	High	High	High	High	High	High	High	High	High
High	High	High	High	High	High	High	High	High	High
High	High	High	High	High	High	High	High	High	High
High	High	High	High	High	High	High	High	High	High
High	High	High	High	High	High	High	High	High	High
High	High	High	High	High	High	High	High	High	High
High	High	High	High	High	High	High	High	High	High
High	High	High	High	High	High	High	High	High	High
High	High	High	High	High	High	High	High	High	High
High	High	High	High	High	High	High	High	High	High
High	High	High	High	High	High	High	High	High	High
High	High	High	High	High	High	High	High	High	High
High	High	High	High	High	High	High	High	High	High
High	High	High	High	High	High	High	High	High	High
Not Found	Not Found	Not Found	High	High	High	High	Not Found	Not Found	Not Found
High	High	High	High	High	High	High	High	High	High
High	High	High	Not Found	Not Found	Not Found	Not Found	Not Found	Not Found	Not Found
High	High	High	High	High	High	High	High	High	High
High	High	High	High	High	High	High	High	High	High
High	High	High	High	High	High	High	High	High	High
High	High	High	High	High	High	High	High	High	High
High	High	High	High	High	High	High	High	High	High
High	High	High	High	High	High	High	High	High	High
High	High	High	High	High	High	High	High	High	High
High	High	High	High	High	High	High	High	High	High
High	High	High	High	High	High	High	High	High	High
High	High	High	High	High	High	High	High	High	High
High	High	High	High	High	High	High	High	High	High
High	High	High	High	High	High	High	High	High	High
High	High	High	High	High	High	High	High	High	High
High	High	High	High	High	High	High	High	High	High
High	High	High	Not Found	Not Found	Not Found	Not Found	High	High	High
High	High	High	High	High	High	High	High	High	High
High	High	High	High	High	High	High	High	High	High
High	High	High	High	High	High	High	High	High	High

High	High	High	High	High	High	High	High	High	High
High	High	High	High	High	High	High	High	High	High
High	High	High	High	High	High	High	High	High	High
High	High	High	High	High	High	High	High	High	High
High	High	High	High	High	High	High	High	High	High
High	High	High	High	High	High	High	High	High	High
High	High	High	High	High	High	High	High	High	High
High	High	High	High	High	High	High	High	High	High
High	High	High	High	High	High	High	High	High	High
High	High	High	High	High	High	High	High	High	High
High	High	High	High	High	High	High	High	High	High
High	High	High	High	High	High	High	High	High	High
High	High	High	High	High	High	High	High	High	High
High	High	High	High	High	High	High	High	High	High
High	High	High	High	High	High	High	High	High	High
High	High	High	High	High	High	High	High	High	High
High	High	High	High	High	High	High	High	High	High
High	High	High	High	High	High	High	High	High	High
High	High	High	High	High	High	High	High	High	High
High	High	High	High	High	High	High	High	High	High
High	High	High	High	High	High	High	High	High	High
High	High	High	High	High	High	High	High	High	High
High	High	High	High	High	High	High	High	High	High
High	High	High	High	High	High	High	High	High	High
High	High	High	High	High	High	High	High	High	High
High	High	High	High	High	High	High	High	High	High
High	High	High	High	High	High	High	High	High	High
Not Found	Not Found	Not Found	High	High	High	High	Not Found	Not Found	Not Found
High	High	High	High	High	High	High	High	High	High
High	High	High	High	High	High	High	High	High	High
High	High	High	High	High	High	High	High	High	High
High	High	High	High	High	High	High	High	High	High
High	High	High	High	High	High	High	High	High	High
High	High	High	High	High	High	High	High	High	High
High	High	High	High	High	High	High	High	High	High
High	High	High	High	High	High	High	High	High	High
High	High	High	High	High	High	High	High	High	High
High	High	High	High	High	High	High	High	High	High
High	High	High	High	High	High	High	High	High	High
High	High	High	High	High	High	High	High	High	High
High	High	High	High	High	High	High	High	High	High
High	High	High	High	High	High	High	High	High	High
High	High	High	High	High	High	High	High	High	High
High	High	High	High	High	High	High	High	High	High
High	High	High	High	High	High	High	High	High	High
High	High	High	High	High	High	High	High	High	High
High	High	High	High	High	High	High	High	High	High
High	High	High	High	High	High	High	High	High	High
High	High	High	High	High	High	High	High	High	High
High	High	High	High	High	High	High	High	High	High

High	High	High	High	High	High	High	High	High	High
High	High	High	High	High	High	High	High	High	High
High	High	High	High	High	High	High	High	High	High
High	High	High	High	High	High	High	High	High	High
High	High	High	High	High	High	High	High	High	High
High	High	High	High	High	High	High	High	High	High
High	High	High	High	High	High	High	High	High	High
High	High	High	High	High	High	High	High	High	High
High	High	High	High	High	High	High	High	High	High
High	High	High	High	High	High	High	High	High	High
High	High	High	High	High	High	High	High	High	High
High	High	High	High	High	High	High	High	High	High
High	High	High	High	High	High	High	High	High	High
High	High	High	High	High	High	High	High	High	High
High	High	High	High	High	High	High	High	High	High
High	High	High	High	High	High	High	High	High	High
High	High	High	High	High	High	High	High	High	High
High	High	High	High	High	High	High	High	High	High
High	High	High	High	High	High	High	High	High	High
High	High	High	High	High	High	High	High	High	High
High	High	High	High	High	High	High	High	High	High
High	High	High	High	High	High	High	High	High	High
High	High	High	High	High	High	High	High	High	High
High	High	High	High	High	High	High	High	High	High
High	High	High	High	High	High	High	High	High	High
High	High	High	High	High	High	High	High	High	High
Not Found	Not Found	Not Found	High	High	High	High	Not Found	Not Found	Not Found
High	High	High	High	High	High	High	High	High	High
High	High	High	High	High	High	High	High	High	High
High	High	High	High	High	High	High	High	High	High
High	High	High	High	High	High	High	High	High	High
High	High	High	High	High	High	High	High	High	High
High	High	High	High	High	High	High	High	High	High
High	High	High	High	High	High	High	High	High	High
High	High	High	High	High	High	High	High	High	High
High	High	High	High	High	High	High	High	High	High
High	High	High	High	High	High	High	High	High	High
High	High	High	High	High	High	High	High	High	High
High	High	High	High	High	High	High	High	High	High
High	High	High	High	High	High	High	High	High	High
High	High	High	Not Found	Not Found	Not Found	Not Found	Not Found	Not Found	Not Found
High	High	High	High	High	High	High	High	High	High
High	High	High	High	High	High	High	High	High	High
High	High	High	High	High	High	High	High	High	High
High	High	High	High	High	High	High	High	High	High
High	High	High	High	High	High	High	High	High	High
High	High	High	High	High	High	High	High	High	High
High	High	High	High	High	High	High	High	High	High
High	High	High	High	High	High	High	High	High	High
High	High	High	High	High	High	High	High	High	High

High	High	High	High	High	High	High	High	High	High
High	High	High	High	High	High	High	High	High	High
High	High	High	High	High	High	High	High	High	High
Not Found	Not Found	Not Found	High	High	High	High	Not Found	Not Found	Not Found
High	High	High	High	High	High	High	High	High	High
High	High	High	High	High	High	High	High	High	High
High	High	High	High	High	High	High	High	High	High
High	High	High	High	High	High	High	High	High	High
High	High	High	High	High	High	High	High	High	High
High	High	High	High	High	High	High	High	High	High
High	High	High	High	High	High	High	High	High	High
High	High	High	High	High	High	High	High	High	High
High	High	High	High	High	High	High	High	High	High
High	High	High	High	High	High	High	High	High	High
High	High	High	High	High	High	High	High	High	High
High	High	High	High	High	High	High	High	High	High
High	High	High	High	High	High	High	High	High	High
High	High	High	High	High	High	High	High	High	High
High	High	High	High	High	High	High	High	High	High
High	High	High	High	High	High	High	High	High	High
High	High	High	High	High	High	High	High	High	High
High	High	High	High	High	High	High	High	High	High
High	High	High	High	High	High	High	Not Found	Not Found	Not Found
High	High	High	High	High	High	High	High	High	High
High	High	High	High	High	High	High	High	High	High
High	High	High	High	High	High	High	High	High	High
High	High	High	High	High	High	High	High	High	High
High	High	High	High	High	High	High	High	High	High
High	High	High	High	High	High	High	High	High	High
High	High	High	High	High	High	High	High	High	High
High	High	High	High	High	High	High	High	High	High
High	High	High	High	High	High	High	High	High	High
High	High	High	High	High	High	High	High	High	High
High	High	High	High	High	High	High	High	High	High
High	High	High	High	High	High	High	High	High	High
High	High	High	High	High	High	High	High	High	High
High	High	High	High	High	High	High	High	High	High
High	High	High	High	High	High	High	High	High	High
High	High	High	High	High	High	High	High	High	High
High	High	High	High	High	High	High	High	High	High
High	High	High	High	High	High	High	High	High	High
High	High	High	High	High	High	High	High	High	High
Not Found	Not Found	Not Found	Not Found	Not Found	Not Found	Not Found	High	High	High
High	High	High	High	High	High	High	High	High	High
High	High	High	High	High	High	High	High	High	High
High	High	High	High	High	High	High	High	High	High
High	High	High	High	High	High	High	High	High	High
High	High	High	High	High	High	High	High	High	High
Not Found	Not Found	Not Found	Not Found	Not Found	Not Found	Not Found	High	High	High
High	High	High	High	High	High	High	High	High	High

High	High	High	High	High	High	High	High	High	High
High	High	High	High	High	High	High	High	High	High
High	High	High	High	High	High	High	High	High	High
High	High	High	High	High	High	High	High	High	High
High	High	High	High	High	High	High	High	High	High
Not Found	Not Found	Not Found	High	High	High	High	High	High	High
Not Found	Not Found	Not Found	High	High	High	High	High	High	High
High	High	High	High	High	High	High	High	High	High
High	High	High	High	High	High	High	High	High	High
High	High	High	High	High	High	High	High	High	High
High	High	High	High	High	High	High	High	High	High
High	High	High	High	High	High	High	High	High	High
High	High	High	High	High	High	High	High	High	High
High	High	High	High	High	High	High	High	High	High
High	High	High	High	High	High	High	High	High	High
High	High	High	High	High	High	High	High	High	High
High	High	High	High	High	High	High	High	High	High
High	High	High	High	High	High	High	High	High	High
High	High	High	High	High	High	High	High	High	High
High	High	High	High	High	High	High	High	High	High
High	High	High	High	High	High	High	High	High	High
High	High	High	High	High	High	High	High	High	High
High	High	High	High	High	High	High	High	High	High
High	High	High	High	High	High	High	High	High	High
High	High	High	High	High	High	High	High	High	High
High	High	High	High	High	High	High	High	High	High
High	High	High	High	High	High	High	High	High	High
High	High	High	High	High	High	High	High	High	High
High	High	High	High	High	High	High	High	High	High
High	High	High	High	High	High	High	High	High	High
High	High	High	High	High	High	High	High	High	High
High	High	High	High	High	High	High	High	High	High
High	High	High	High	High	High	High	High	High	High
High	High	High	High	High	High	High	High	High	High
High	High	High	High	High	High	High	High	High	High
High	High	High	High	High	High	High	High	High	High
High	High	High	High	High	High	High	High	High	High
High	High	High	High	High	High	High	High	High	High
High	High	High	High	High	High	High	High	High	High
High	High	High	High	High	High	High	High	High	High
High	High	High	High	High	High	High	High	High	High
High	High	High	High	High	High	High	High	High	High
High	High	High	High	High	High	High	Not Found	Not Found	Not Found
High	High	High	High	High	High	High	High	High	High
High	High	High	High	High	High	High	High	High	High
High	High	High	High	High	High	High	High	High	High
High	High	High	High	High	High	High	High	High	High
High	High	High	High	High	High	High	High	High	High
High	High	High	High	High	High	High	High	High	High
High	High	High	High	High	High	High	High	High	High

High	High	High	High	High	High	High	High	High	High
High	High	High	High	High	High	High	High	High	High
High	High	High	High	High	High	High	High	High	High
High	High	High	High	High	High	High	High	High	High
High	High	High	High	High	High	High	High	High	High
High	High	High	High	High	High	High	High	High	High
High	High	High	High	High	High	High	High	High	High
High	High	High	High	High	High	High	High	High	High
High	High	High	High	High	High	High	High	High	High
High	High	High	Not Found	Not Found	Not Found	Not Found	High	High	High
High	High	High	High	High	High	High	High	High	High
High	High	High	High	High	High	High	High	High	High
High	High	High	High	High	High	High	High	High	High
High	High	High	High	High	High	High	High	High	High
High	High	High	High	High	High	High	High	High	High
High	High	High	High	High	High	High	High	High	High
Not Found	Not Found	Not Found	Not Found	Not Found	Not Found	Not Found	Not Found	Not Found	Not Found
High	High	High	High	High	High	High	High	High	High
High	High	High	High	High	High	High	High	High	High
High	High	High	High	High	High	High	High	High	High
High	High	High	High	High	High	High	High	High	High
High	High	High	High	High	High	High	High	High	High
High	High	High	High	High	High	High	High	High	High
High	High	High	High	High	High	High	High	High	High
High	High	High	High	High	High	High	High	High	High
High	High	High	High	High	High	High	High	High	High
High	High	High	High	High	High	High	High	High	High
High	High	High	High	High	High	High	High	High	High
High	High	High	High	High	High	High	High	High	High
High	High	High	High	High	High	High	High	High	High
High	High	High	High	High	High	High	High	High	High
High	High	High	High	High	High	High	High	High	High
High	High	High	High	High	High	High	High	High	High
High	High	High	High	High	High	High	High	High	High
High	High	High	High	High	High	High	High	High	High
High	High	High	High	High	High	High	High	High	High
High	High	High	High	High	High	High	High	High	High
High	High	High	High	High	High	High	High	High	High
High	High	High	High	High	High	High	High	High	High
High	High	High	High	High	High	High	High	High	High
High	High	High	High	High	High	High	High	High	High
High	High	High	High	High	High	High	High	High	High
High	High	High	High	High	High	High	High	High	High
High	High	High	High	High	High	High	High	High	High
High	High	High	High	High	High	High	High	High	High
High	High	High	High	High	High	High	High	High	High
High	High	High	High	High	High	High	High	High	High
High	High	High	High	High	High	High	High	High	High
High	High	High	High	High	High	High	High	High	High
High	High	High	High	High	High	High	High	High	High

High	High	High	High	High	High	High	High	High	High
High	High	High	High	High	High	High	High	High	High
High	High	High	High	High	High	High	High	High	High
High	High	High	High	High	High	High	High	High	High
High	High	High	High	High	High	High	High	High	High
High	High	High	High	High	High	High	High	High	High
High	High	High	High	High	High	High	High	High	High
High	High	High	High	High	High	High	High	High	High
High	High	High	High	High	High	High	High	High	High
High	High	High	High	High	High	High	High	High	High
High	High	High	High	High	High	High	High	High	High
High	Not Found	Not Found	High	High	High	High	High	High	High
High	High	High	High	High	High	High	High	High	High
High	High	High	High	High	High	High	High	High	High
High	High	High	High	High	High	High	High	High	High
High	High	High	High	High	High	High	High	High	High
Not Found	Not Found	Not Found	High	High	High	High	High	High	High
High	High	High	High	High	High	High	High	High	High
High	High	High	High	High	High	High	High	High	High
High	High	High	High	High	High	High	High	High	High
High	High	High	High	High	High	High	High	High	High
High	High	High	High	High	High	High	High	High	High
High	High	High	High	High	High	High	High	High	High
High	High	High	High	High	High	High	High	High	High
High	High	High	High	High	High	High	High	High	High
High	High	High	High	High	High	High	High	High	High
High	High	High	High	High	High	High	High	High	High
High	High	High	High	High	High	High	High	High	High
High	High	High	High	High	High	High	High	High	High
High	High	High	High	High	High	High	High	High	High
High	High	High	High	High	High	High	High	High	High
High	High	High	High	High	High	High	High	High	High
High	High	High	High	High	High	High	High	High	High
High	High	High	High	High	High	High	High	High	High
High	High	High	High	High	High	High	High	High	High
High	High	High	High	High	High	High	High	High	High
High	High	High	High	High	High	High	High	High	High
High	High	High	High	High	High	High	High	High	High
High	High	High	High	High	High	High	High	High	High
High	High	High	High	High	High	High	High	High	High
High	High	High	High	High	High	High	High	High	High
High	High	High	High	High	High	High	High	High	High
High	High	High	High	High	High	High	High	High	High
High	High	High	High	High	High	High	High	High	High
High	High	High	High	High	High	High	High	High	High
High	High	High	High	High	High	High	High	High	High
Not Found	Not Found	Not Found	High	High	High	High	Not Found	Not Found	Not Found
High	High	High	High	High	High	High	High	High	High
High	High	High	High	High	High	High	High	High	High
High	High	High	High	High	High	High	High	High	High

High	High	High	High	High	High	High	High	High	High
High	High	High	High	High	High	High	High	High	High
High	High	High	High	High	High	High	High	High	High
High	High	High	High	High	High	High	High	High	High
High	High	High	High	High	High	High	High	High	High
High	High	High	High	High	High	High	High	High	High
High	High	High	High	High	High	High	High	High	High
High	High	High	High	High	High	High	High	High	High
High	High	High	High	High	High	High	High	High	High
High	High	High	High	High	High	High	High	High	High
High	High	High	High	High	High	High	High	High	High
High	High	High	High	High	High	High	High	High	High
High	High	High	High	High	High	High	High	High	High
High	High	High	High	High	High	High	High	High	High
High	High	High	High	High	High	High	High	High	High
High	High	High	High	High	High	High	High	High	High
High	High	High	High	High	High	High	High	High	High
High	High	High	High	High	High	High	High	High	High
High	High	High	High	High	High	High	High	High	High
High	High	High	High	High	High	High	High	High	High
High	High	High	High	High	High	High	High	High	High
High	High	High	High	High	High	High	High	High	High
High	High	High	High	High	High	High	High	High	High
High	High	High	High	High	High	High	High	High	High
High	High	High	High	High	High	High	High	High	High
High	High	High	High	High	High	High	High	High	High
High	High	High	High	High	High	High	High	High	High
High	High	High	High	High	High	High	High	High	High
Not Found	Not Found	Not Found	Not Found	Not Found	Not Found	Not Found	Not Found	Not Found	Not Found
High	High	High	High	High	High	High	High	High	High
High	High	High	High	High	High	High	High	High	High
High	High	High	High	High	High	High	Not Found	Not Found	Not Found
High	High	High	High	High	High	High	High	High	High
High	High	High	High	High	High	High	High	High	High
High	High	High	High	High	High	High	High	High	High
High	High	High	High	High	High	High	High	High	High
High	High	High	High	High	High	High	High	High	High
High	High	High	High	High	High	High	High	High	High
High	High	High	High	High	High	High	High	High	High
High	High	High	High	High	High	High	High	High	High
High	High	High	High	High	High	High	High	High	High
High	High	High	High	High	High	High	High	High	High
High	High	High	High	High	High	High	High	High	High
High	High	High	High	High	High	High	High	High	High
High	High	High	High	High	High	High	High	High	High
High	High	High	High	High	High	High	High	High	High
High	High	High	High	High	High	High	High	High	High
High	High	High	Not Found	Not Found	Not Found	Not Found	Not Found	Not Found	Not Found
High	High	High	High	High	High	High	High	High	High
High	High	High	High	High	High	High	High	High	High

High	High	High	High	High	High	High	High	High	High
High	High	High	High	High	High	High	High	High	High
High	High	High	High	High	High	High	High	High	High
High	High	High	High	High	High	High	High	High	High
High	High	High	High	High	High	High	High	High	High
High	High	High	High	High	High	High	High	High	High
High	High	High	High	High	High	High	High	High	High
High	High	High	High	High	High	High	High	High	High
High	High	High	High	High	High	High	High	High	High
High	High	High	High	High	High	High	High	High	High
High	High	High	High	High	High	High	High	High	High
High	High	High	High	High	High	High	High	High	High
High	High	High	High	High	High	High	High	High	High
High	High	High	High	High	High	High	High	High	High
High	High	High	High	High	High	High	High	High	High
High	High	High	High	High	High	High	High	High	High
High	High	High	High	High	High	High	High	High	High
High	High	High	High	High	High	High	High	High	High
High	High	High	High	High	High	High	High	High	High
High	High	High	High	High	High	High	High	High	High
High	High	High	High	High	High	High	High	High	High
High	High	High	High	High	High	High	High	High	High
High	High	High	High	High	High	High	High	High	High
High	High	High	High	High	High	High	High	High	High
High	High	High	High	High	High	High	High	High	High
High	High	High	High	High	High	High	High	High	High
High	High	High	High	High	High	High	High	High	High
High	High	High	High	High	High	High	High	High	High
High	High	High	High	High	High	High	High	High	High
High	High	High	High	High	High	High	High	High	High
High	High	High	High	High	High	High	High	High	High
High	High	High	High	High	High	High	High	High	High
High	High	High	High	High	High	High	High	High	High
High	High	High	High	High	High	High	High	High	High
High	High	High	High	High	High	High	High	High	High
High	High	High	High	High	High	High	High	High	High
High	High	High	High	High	High	High	High	High	High
High	High	High	High	High	High	High	High	High	High
High	High	High	High	High	High	High	High	High	High
High	High	High	High	High	High	High	High	High	High
High	High	High	High	High	High	High	High	High	High
High	High	High	High	High	High	High	High	High	High
Not Found	Not Found	Not Found	Not Found	Not Found	Not Found	Not Found	High	High	High
High	High	High	High	High	High	High	High	High	High
High	High	High	High	High	High	High	High	High	High
High	High	High	High	High	High	High	High	High	High
High	High	High	High	High	High	High	High	High	High
High	High	High	High	High	High	High	High	High	High
High	High	High	High	High	High	High	High	High	High
High	High	High	High	High	High	High	High	High	High

High	High	High	High	High	High	High	High	High	High
High	High	High	High	High	High	High	High	High	High
High	High	High	High	High	High	High	High	High	High
High	High	High	High	High	High	High	High	High	High
High	High	High	High	High	High	High	High	High	High
High	High	High	High	High	High	High	High	High	High
High	High	High	High	High	High	High	High	High	High
High	High	High	High	High	High	High	High	High	High
High	High	High	High	High	High	High	High	High	High
High	High	High	High	High	High	High	High	High	High
High	High	High	High	High	High	High	High	High	High
High	High	High	High	High	High	High	High	High	High
High	High	High	High	High	High	High	High	High	High
High	High	High	High	High	High	High	High	High	High
High	High	High	High	High	High	High	High	High	High
High	High	High	High	High	High	High	High	High	High
Not Found	Not Found	Not Found	Not Found	Not Found	Not Found	Not Found	Not Found	Not Found	Not Found
High	High	High	High	High	High	High	High	High	High
High	High	High	High	High	High	High	High	High	High
High	High	High	High	High	High	High	High	High	High
High	High	High	High	High	High	High	High	High	High
High	High	High	High	High	High	High	High	High	High
High	High	High	High	High	High	High	High	High	High
High	High	High	High	High	High	High	High	High	High
High	High	High	High	High	High	High	High	High	High
High	High	High	High	High	High	High	High	High	High
High	High	High	High	High	High	High	High	High	High
High	High	High	High	High	High	High	High	High	High
High	High	High	High	High	High	High	High	High	High
High	High	High	High	High	High	High	High	High	High
High	High	High	High	High	High	High	High	High	High
High	High	High	High	High	High	High	High	High	High
High	High	High	High	High	High	High	High	High	High
High	High	High	High	High	High	High	High	High	High
High	High	High	High	High	High	High	High	High	High
High	High	High	High	High	High	High	High	High	High
High	High	High	High	High	High	High	Not Found	Not Found	Not Found
High	High	High	High	High	High	High	High	High	High
High	High	High	High	High	High	High	High	High	High
High	High	High	High	High	High	High	High	High	High
High	High	High	High	High	High	High	High	High	High
High	High	High	High	High	High	High	High	High	High
High	High	High	High	High	High	High	High	High	High
High	High	High	High	High	High	High	High	High	High
High	High	High	High	High	High	High	High	High	High
High	High	High	High	High	High	High	High	High	High
High	High	High	High	High	High	High	High	High	High
High	High	High	High	High	High	High	High	High	High
High	High	High	High	High	High	High	High	High	High
High	High	High	High	High	High	High	High	High	High

High	High	High	High	High	High	High	High	High	High
High	High	High	High	High	High	High	High	High	High
High	High	High	High	High	High	High	High	High	High
High	High	High	High	High	High	High	High	High	High
High	High	High	High	High	High	High	High	High	High
High	High	High	High	High	High	High	High	High	High
High	High	High	High	High	High	High	High	High	High
High	High	High	High	High	High	High	High	High	High
High	High	High	High	High	High	High	High	High	High
High	High	High	High	High	High	High	High	High	High
High	High	High	High	High	High	High	High	High	High
High	High	High	High	High	High	High	High	High	High
Not Found	Not Found	Not Found	High	High	High	High	High	High	High
High	High	High	High	High	High	High	High	High	High
High	High	High	High	High	High	High	High	High	High
High	High	High	High	High	High	High	High	High	High
High	High	High	High	High	High	High	High	High	High
High	High	High	High	High	High	High	High	High	High
High	High	High	High	High	High	High	High	High	High
High	High	High	High	High	High	High	High	High	High
High	High	High	High	High	High	High	High	High	High
High	High	High	High	High	High	High	High	High	High
High	High	High	High	High	High	High	High	High	High
High	High	High	High	High	High	High	High	High	High
High	High	High	Not Found	Not Found	Not Found	Not Found	Not Found	Not Found	Not Found
High	High	High	High	High	High	High	High	High	High
High	High	High	High	High	High	High	High	High	High
High	High	High	High	High	High	High	High	High	High
High	High	High	High	High	High	High	High	High	High
High	High	High	High	High	High	High	High	High	High
High	High	High	High	High	High	High	High	High	High
High	High	High	High	High	High	High	High	High	High
High	High	High	High	High	High	High	High	High	High
High	High	High	High	High	High	High	High	High	High
High	High	High	High	High	High	High	High	High	High
High	High	High	High	High	High	High	High	High	High
Not Found	Not Found	Not Found	High	High	High	High	Not Found	Not Found	Not Found
High	High	High	High	High	High	High	High	High	High
High	High	High	High	High	High	High	High	High	High
High	High	High	High	High	High	High	High	High	High
High	High	High	High	High	High	High	High	High	High
High	High	High	Not Found	Not Found	Not Found	Not Found	Not Found	Not Found	Not Found
High	High	High	High	High	High	High	High	High	High
High	High	High	High	High	High	High	High	High	High
High	High	High	High	High	High	High	High	High	High
High	High	High	High	High	High	High	High	High	High
High	High	High	High	High	High	High	High	High	High
High	High	High	High	High	High	High	High	High	High
High	High	High	High	High	High	High	High	High	High
High	High	High	High	High	High	High	High	High	High

High	High	High	High	High	High	High	High	High	High
High	High	High	High	High	High	High	High	High	High
High	High	High	High	High	High	High	High	High	High
High	High	High	High	High	High	High	High	High	High
High	High	High	High	High	High	High	High	High	High
High	High	High	High	High	High	High	High	High	High
High	High	High	High	High	High	High	High	High	High
High	High	High	High	High	High	High	High	High	High
High	High	High	High	High	High	High	High	High	High
High	High	High	High	High	High	High	High	High	High
High	High	High	High	High	High	High	High	High	High
High	High	High	High	High	High	High	High	High	High
High	High	High	High	High	High	High	High	High	High
High	High	High	High	High	High	High	High	High	High
High	High	High	High	High	High	High	High	High	High
High	High	High	High	High	High	High	High	High	High
High	High	High	High	High	High	High	High	High	High
High	High	High	High	High	High	High	High	High	High
High	High	High	High	High	High	High	High	High	High
High	High	High	High	High	High	High	High	High	High
High	High	High	High	High	High	High	High	High	High
High	High	High	High	High	High	High	High	High	High
High	High	High	High	High	High	High	High	High	High
High	High	High	High	High	High	High	High	High	High
High	High	High	High	High	High	High	High	High	High
High	High	High	High	High	High	High	High	High	High
High	High	High	High	High	High	High	High	High	High
High	High	High	High	High	High	High	High	High	High
High	High	High	High	High	High	High	High	High	High
High	High	High	High	High	High	High	High	High	High
High	High	High	High	High	High	High	High	High	High
High	High	High	High	High	High	High	High	High	High
High	High	High	High	High	High	High	High	High	High
High	High	High	High	High	High	High	High	High	High
High	High	High	High	High	High	High	High	High	High
High	High	High	High	High	High	High	High	High	High
High	High	High	High	High	High	High	High	High	High
High	High	High	High	High	High	High	High	High	High
High	High	High	High	High	High	High	High	High	High
High	High	High	High	High	High	High	High	High	High
High	High	High	High	High	High	High	High	High	High
High	High	High	High	High	High	High	High	High	High
High	High	High	High	High	High	High	High	High	High
High	High	High	High	High	High	High	High	High	High
High	High	High	High	High	High	High	High	High	High
High	High	High	High	High	High	High	High	High	High
High	High	High	High	High	High	High	High	High	High
High	High	High	High	High	High	High	High	High	High
Not Found	Not Found	Not Found	Not Found	Not Found	Not Found	Not Found	High	High	High
High	High	High	High	High	High	High	High	High	High

High	High	High	High	High	High	High	High	High	High
High	High	High	High	High	High	High	High	High	High
High	High	High	High	High	High	High	High	High	High
High	High	High	High	High	High	High	High	High	High
High	High	High	High	High	High	High	High	High	High
High	High	High	High	High	High	High	High	High	High
High	High	High	High	High	High	High	High	High	High
High	High	High	High	High	High	High	High	High	High
High	High	High	High	High	High	High	High	High	High
High	High	High	High	High	High	High	High	High	High
High	High	High	High	High	High	High	High	High	High
High	High	High	High	High	High	High	High	High	High
High	High	High	High	High	High	High	High	High	High
High	High	High	High	High	High	High	High	High	High
High	High	High	High	High	High	High	High	High	High
High	High	High	High	High	High	High	High	High	High
High	High	High	High	High	High	High	High	High	High
High	High	High	High	High	High	High	High	High	High
High	High	High	High	High	High	High	High	High	High
High	High	High	High	High	High	High	High	High	High
High	High	High	High	High	High	High	High	High	High
High	High	High	High	High	High	High	High	High	High
High	High	High	High	High	High	High	High	High	High
High	High	High	High	High	High	High	High	High	High
High	High	High	High	High	High	High	High	High	High
High	High	High	High	High	High	High	High	High	High
High	High	High	High	High	High	High	High	High	High
High	High	High	High	High	High	High	High	High	High
High	High	High	High	High	High	High	High	High	High
High	High	High	High	High	High	High	High	High	High
High	High	High	High	High	High	High	High	High	High
High	High	High	High	High	High	High	High	High	High
High	High	High	High	High	High	High	High	High	High
High	High	High	High	High	High	High	High	High	High
High	High	High	High	High	High	High	High	High	High
Not Found	Not Found	Not Found	Not Found	Not Found	Not Found	Not Found	Not Found	Not Found	Not Found
High	High	High	High	High	High	High	High	High	High
High	High	High	High	High	High	High	High	High	High
High	High	High	High	High	High	High	High	High	High
High	High	High	High	High	High	High	High	High	High
High	High	High	High	High	High	High	High	High	High
High	High	High	High	High	High	High	High	High	High
High	High	High	High	High	High	High	High	High	High
High	High	High	High	High	High	High	High	High	High
High	High	High	High	High	High	High	High	High	High
High	High	High	High	High	High	High	High	High	High
High	High	High	High	High	High	High	High	High	High
High	High	High	High	High	High	High	High	High	High
High	High	High	High	High	High	High	High	High	High
High	High	High	High	High	High	High	High	High	High

High	High	High	High	High	High	High	High	High	High
High	High	High	High	High	High	High	High	High	High
High	High	High	High	High	High	High	High	High	High
High	High	High	High	High	High	High	High	High	High
High	High	High	High	High	High	High	High	High	High
High	High	High	High	High	High	High	High	High	High
High	High	High	High	High	High	High	High	High	High
High	High	High	High	High	High	High	High	High	High
High	High	High	High	High	High	High	High	High	High
High	High	High	High	High	High	High	High	High	High
High	High	High	High	High	High	High	High	High	High
High	High	High	High	High	High	High	High	High	High
High	High	High	High	High	High	High	High	High	High
High	High	High	High	High	High	High	High	High	High
High	High	High	High	High	High	High	High	High	High
High	High	High	High	High	High	High	High	High	High
High	High	High	High	High	High	High	High	High	High
High	High	High	High	High	High	High	High	High	High
High	High	High	High	High	High	High	High	High	High
High	High	High	High	High	High	High	High	High	High
High	High	High	High	High	High	High	High	High	High
High	High	High	High	High	High	High	High	High	High
High	High	High	High	High	High	High	High	High	High
High	High	High	High	High	High	High	High	High	High
High	High	High	High	High	High	High	High	High	High
High	High	High	High	High	High	High	High	High	High
High	High	High	High	High	High	High	High	High	High
High	High	High	High	High	High	High	High	High	High
High	High	High	High	High	High	High	High	High	High
High	High	High	High	High	High	High	High	High	High
High	High	High	High	High	High	High	High	High	High
High	High	High	High	High	High	High	High	High	High
High	High	High	High	High	High	High	High	High	High
High	High	High	High	High	High	High	High	High	High
High	High	High	High	High	High	High	High	High	High
High	High	High	High	High	High	High	High	High	High
High	High	High	High	High	High	High	High	High	High
High	High	High	High	High	High	High	High	High	High
High	High	High	High	High	High	High	High	High	High
High	High	High	High	High	High	High	High	High	High
High	High	High	High	High	High	High	High	High	High
High	High	High	High	High	High	High	High	High	High
High	High	High	High	High	High	High	High	High	High
High	High	High	High	High	High	High	High	High	High
High	High	High	High	High	High	High	High	High	High
High	High	High	High	High	High	High	High	High	High
High	High	High	High	High	High	High	High	High	High
High	High	High	High	High	High	High	High	High	High
High	High	High	High	High	High	High	High	High	High
High	High	High	High	High	High	High	High	High	High

High	High	High	High	High	High	High	High	High	High
High	High	High	High	High	High	High	High	High	High
High	High	High	High	High	High	High	High	High	High
High	High	High	High	High	High	High	High	High	High
High	High	High	High	High	High	High	High	High	High
High	High	High	High	High	High	High	High	High	High
High	High	High	High	High	High	High	High	High	High
High	High	High	High	High	High	High	High	High	High
High	High	High	High	High	High	High	High	High	High
High	High	High	High	High	High	High	High	High	High
High	High	High	High	High	High	High	High	High	High
High	High	High	High	High	High	High	High	High	High
High	High	High	High	High	High	High	High	High	High
High	High	High	High	High	High	High	High	High	High
High	High	High	High	High	High	High	High	High	High
High	High	High	High	High	High	High	High	High	High
High	High	High	High	High	High	High	High	High	High
High	High	High	High	High	High	High	High	High	High
High	High	High	High	High	High	High	High	High	High
High	High	High	High	High	High	High	High	High	High
High	High	High	High	High	High	High	High	High	High
High	High	High	High	High	High	High	High	High	High
High	High	High	High	High	High	High	High	High	High
High	High	High	Not Found	Not Found	Not Found	Not Found	High	High	High
High	High	High	High	High	High	High	High	High	High
High	High	High	High	High	High	High	High	High	High
High	High	High	High	High	High	High	High	High	High
High	High	High	High	High	High	High	High	High	High
High	High	High	High	High	High	High	High	High	High
High	High	High	High	High	High	High	High	High	High
High	High	High	High	High	High	High	High	High	High
High	High	High	High	High	High	High	High	High	High
High	High	High	High	High	High	High	High	High	High
High	High	High	High	High	High	High	High	High	High
High	High	High	High	High	High	High	High	High	High
High	High	High	High	High	High	High	High	High	High
High	High	High	High	High	High	High	High	High	High
High	High	High	High	High	High	High	High	High	High
High	High	High	High	High	High	High	High	High	High
High	High	High	High	High	High	High	High	High	High
High	High	High	High	High	High	High	High	High	High
High	High	High	High	High	High	High	High	High	High
High	High	High	High	High	High	High	High	High	High
High	High	High	High	High	High	High	High	High	High
High	High	High	High	High	High	High	High	High	High
High	High	High	High	High	High	High	High	High	High
High	High	High	High	High	High	High	High	High	High
High	High	High	High	High	High	High	High	High	High
High	High	High	High	High	High	High	High	High	High
High	High	High	High	High	High	High	High	High	High

High	High	High	High	High	High	High	High	High	High
High	High	High	High	High	High	High	High	High	High
High	High	High	High	High	High	High	High	High	High
High	High	High	High	High	High	High	High	High	High
High	High	High	High	High	High	High	High	High	High
High	High	High	High	High	High	High	High	High	High
High	High	High	High	High	High	High	High	High	High
High	High	High	High	High	High	High	High	High	High
High	High	High	High	High	High	High	High	High	High
High	High	High	High	High	High	High	High	High	High
High	High	High	High	High	High	High	High	High	High
High	High	High	High	High	High	High	High	High	High
High	High	High	High	High	High	High	High	High	High
High	High	High	High	High	High	High	High	High	High
High	High	High	High	High	High	High	High	High	High
High	High	High	High	High	High	High	High	High	High
High	High	High	High	High	High	High	High	High	High
High	High	High	High	High	High	High	High	High	High
High	High	High	High	High	High	High	High	High	High
High	High	High	High	High	High	High	High	High	High
High	High	High	High	High	High	High	High	High	High
High	High	High	High	High	High	High	High	High	High
High	High	High	High	High	High	High	High	High	High
High	High	High	High	High	High	High	High	High	High
High	High	High	High	High	High	High	High	High	High
High	High	High	High	High	High	High	High	High	High
High	High	High	High	High	High	High	High	High	High
High	High	High	High	High	High	High	High	High	High
High	High	High	High	High	High	High	High	High	High
High	High	High	High	High	High	High	High	High	High
High	High	High	High	High	High	High	High	High	High
High	High	High	High	High	High	High	High	High	High
High	High	High	High	High	High	High	High	High	High
High	High	High	High	High	High	High	High	High	High
High	High	High	High	High	High	High	High	High	High
High	High	High	High	High	High	High	High	High	High
High	High	High	High	High	High	High	High	High	High
High	High	High	High	High	High	High	High	High	High
High	High	High	High	High	High	High	High	High	High
High	High	High	High	High	High	High	High	High	High
High	High	High	High	High	High	High	High	High	High
High	High	High	High	High	High	High	High	High	High
High	High	High	High	High	High	High	High	High	High
High	High	High	High	High	High	High	High	High	High
High	High	High	High	High	High	High	High	High	High
High	High	High	High	High	High	High	High	High	High
High	High	High	High	High	High	High	High	High	High
High	High	High	High	High	High	High	High	High	High
High	High	High	High	High	High	High	High	High	High
High	High	High	High	High	High	High	High	High	High

High	High	High	High	High	High	High	High	High	High
High	High	High	High	High	High	High	High	High	High
High	High	High	High	High	High	High	High	High	High
High	High	High	High	High	High	High	High	High	High
High	High	High	High	High	High	High	High	High	High
High	High	High	High	High	High	High	High	High	High
High	High	High	High	High	High	High	High	High	High
High	High	High	High	High	High	High	High	High	High
High	High	High	High	High	High	High	High	High	High
High	High	High	High	High	High	High	High	High	High
High	High	High	High	High	High	High	High	High	High
High	High	High	High	High	High	High	High	High	High
High	High	High	High	High	High	High	High	High	High
High	High	High	High	High	High	High	High	High	High
High	High	High	High	High	High	High	High	High	High
High	High	High	High	High	High	High	High	High	High
High	High	High	High	High	High	High	High	High	High
High	High	High	High	High	High	High	High	High	High
High	High	High	High	High	High	High	High	High	High
High	High	High	High	High	High	High	High	High	High
High	High	High	High	High	High	High	High	High	High
High	High	High	High	High	High	High	High	High	High
High	High	High	High	High	High	High	High	High	High
High	High	High	High	High	High	High	High	High	High
High	High	High	High	High	High	High	High	High	High
High	High	High	High	High	High	High	High	High	High
High	High	High	High	High	High	High	High	High	High
High	High	High	High	High	High	High	High	High	High
High	High	High	High	High	High	High	High	High	High
High	High	High	High	High	High	High	High	High	High
High	High	High	High	High	High	High	High	High	High
High	High	High	High	High	High	High	High	High	High
High	High	High	High	High	High	High	High	High	High
High	High	High	High	High	High	High	High	High	High
High	High	High	High	High	High	High	High	High	High
High	High	High	High	High	High	High	High	High	High
High	High	High	High	High	High	High	High	High	High
High	High	High	High	High	High	High	High	High	High
High	High	High	High	High	High	High	High	High	High
High	High	High	High	High	High	High	High	High	High
High	High	High	High	High	High	High	High	High	High
High	High	High	High	High	High	High	High	High	High
High	High	High	High	High	High	High	High	High	High
High	High	High	High	High	High	High	High	High	High
High	High	High	High	High	High	High	High	High	High
High	High	High	High	High	High	High	High	High	High
High	High	High	High	High	High	High	High	High	High
High	High	High	High	High	High	High	High	High	High
High	High	High	High	High	High	High	High	High	High
High	High	High	High	High	High	High	High	High	High

High	High	High	High	High	High	High	High	High	High
High	High	High	High	High	High	High	High	High	High
High	High	High	High	High	High	High	High	High	High
High	High	High	High	High	High	High	High	High	High
High	High	High	High	High	High	High	High	High	High
High	High	High	High	High	High	High	High	High	High
High	High	High	High	High	High	High	High	High	High
High	High	High	High	High	High	High	High	High	High
High	High	High	High	High	High	High	High	High	High
High	High	High	High	High	High	High	High	High	High
High	High	High	High	High	High	High	High	High	High
High	High	High	High	High	High	High	High	High	High
High	High	High	High	High	High	High	High	High	High
High	High	High	High	High	High	High	High	High	High
High	High	High	High	High	High	High	High	High	High
High	High	High	High	High	High	High	High	High	High
High	High	High	High	High	High	High	High	High	High
High	High	High	High	High	High	High	High	High	High
High	High	High	High	High	High	High	High	High	High
High	High	High	High	High	High	High	High	High	High
High	High	High	High	High	High	High	High	High	High
High	High	High	High	High	High	High	High	High	High
High	High	High	High	High	High	High	High	High	High
High	High	High	High	High	High	High	High	High	High
High	High	High	High	High	High	High	High	High	High
High	High	High	High	High	High	High	High	High	High
High	High	High	High	High	High	High	High	High	High
High	High	High	High	High	High	High	High	High	High
High	High	High	High	High	High	High	High	High	High
High	High	High	High	High	High	High	High	High	High
High	High	High	High	High	High	High	High	High	High
High	High	High	High	High	High	High	High	High	High
High	High	High	High	High	High	High	High	High	High
High	High	High	High	High	High	High	High	High	High
High	High	High	High	High	High	High	High	High	High
High	High	High	High	High	High	High	High	High	High
High	High	High	High	High	High	High	High	High	High
High	High	High	High	High	High	High	High	High	High
High	High	High	High	High	High	High	High	High	High
High	High	High	High	High	High	High	High	High	High
High	High	High	High	High	High	High	High	High	High
High	High	High	High	High	High	High	High	High	High
High	High	High	High	High	High	High	High	High	High
High	High	High	High	High	High	High	High	High	High
High	High	High	High	High	High	High	High	High	High
High	High	High	High	High	High	High	High	High	High
High	High	High	High	High	High	High	High	High	High
High	High	High	High	High	High	High	High	High	High
High	High	High	High	High	High	High	High	High	High
High	High	High	High	High	High	High	High	High	High

High	High	High	High	High	High	High	High	High	High
High	High	High	High	High	High	High	High	High	High
High	High	High	High	High	High	High	High	High	High
High	High	High	High	High	High	High	High	High	High
High	High	High	High	High	High	High	High	High	High
High	High	High	High	High	High	High	High	High	High
High	High	High	High	High	High	High	High	High	High
High	High	High	High	High	High	High	High	High	High
High	High	High	High	High	High	High	High	High	High
High	High	High	High	High	High	High	High	High	High
High	High	High	High	High	High	High	High	High	High
High	High	High	High	High	High	High	High	High	High
High	High	High	High	High	High	High	High	High	High
High	High	High	High	High	High	High	High	High	High
High	High	High	High	High	High	High	High	High	High
High	High	High	High	High	High	High	High	High	High
High	High	High	High	High	High	High	High	High	High
High	High	High	High	High	High	High	High	High	High
High	High	High	High	High	High	High	High	High	High
High	High	High	High	High	High	High	High	High	High
High	High	High	High	High	High	High	High	High	High
High	High	High	High	High	High	High	High	High	High
High	High	High	High	High	High	High	High	High	High
High	High	High	High	High	High	High	High	High	High
High	High	High	High	High	High	High	High	High	High
High	High	High	High	High	High	High	High	High	High
High	High	High	High	High	High	High	High	High	High
High	High	High	High	High	High	High	High	High	High
High	High	High	High	High	High	High	High	High	High
High	High	High	High	High	High	High	High	High	High
High	High	High	Not Found	Not Found	Not Found	Not Found	High	High	High
High	High	High	High	High	High	High	High	High	High
High	High	High	High	High	High	High	High	High	High
High	High	High	High	High	High	High	High	High	High
High	High	High	High	High	High	High	High	High	High
High	High	High	High	High	High	High	High	High	High
High	High	High	High	High	High	High	High	High	High
High	High	High	High	High	High	High	High	High	High
High	High	High	High	High	High	High	High	High	High
High	High	High	High	High	High	High	High	High	High
High	High	High	High	High	High	High	High	High	High
High	High	High	High	High	High	High	High	High	High
High	High	High	High	High	High	High	High	High	High
High	High	High	High	High	High	High	High	High	High
High	High	High	High	High	High	High	High	High	High
High	High	High	High	High	High	High	High	High	High
High	High	High	High	High	High	High	High	High	High
High	High	High	High	High	High	High	High	High	High
High	High	High	High	High	High	High	High	High	High
High	High	High	High	High	High	High	High	High	High

High	High	High	High	High	High	High	High	High	High
High	High	High	High	High	High	High	High	High	High
High	High	High	High	High	High	High	High	High	High
High	High	High	High	High	High	High	High	High	High
High	High	High	High	High	High	High	High	High	High
High	High	High	High	High	High	High	High	High	High
Not Found	Not Found	Not Found	High	High	High	High	High	High	High
High	High	High	High	High	High	High	High	High	High
High	High	High	High	High	High	High	High	High	High
High	High	High	High	High	High	High	High	High	High
High	High	High	High	High	High	High	High	High	High
High	High	High	High	High	High	High	High	High	High
High	High	High	High	High	High	High	High	High	High
High	High	High	High	High	High	High	High	High	High
High	High	High	High	High	High	High	High	High	High
High	High	High	High	High	High	High	High	High	High
High	High	High	High	High	High	High	High	High	High
High	High	High	High	High	High	High	High	High	High
High	High	High	High	High	High	High	High	High	High
High	High	High	High	High	High	High	High	High	High
High	High	High	High	High	High	High	High	High	High
High	High	High	High	High	High	High	High	High	High
High	High	High	High	High	High	High	High	High	High
High	High	High	High	High	High	High	High	High	High
High	High	High	High	High	High	High	High	High	High
High	High	High	High	High	High	High	High	High	High
High	High	High	High	High	High	High	High	High	High
High	High	High	High	High	High	High	High	High	High
High	High	High	High	High	High	High	High	High	High
High	High	High	High	High	High	High	High	High	High
High	High	High	High	High	High	High	High	High	High
High	High	High	High	High	High	High	High	High	High
High	High	High	High	High	High	High	High	High	High
High	High	High	High	High	High	High	High	High	High
High	High	High	High	High	High	High	High	High	High
High	High	High	High	High	High	High	High	High	High
High	High	High	High	High	High	High	High	High	High
High	High	High	High	High	High	High	High	High	High
High	High	High	High	High	High	High	High	High	High
High	High	High	High	High	High	High	High	High	High
High	High	High	High	High	High	High	High	High	High
High	High	High	High	High	High	High	High	High	High
High	High	High	High	High	High	High	High	High	High
High	High	High	High	High	High	High	High	High	High
High	High	High	High	High	High	High	High	High	High
High	High	High	High	High	High	High	High	High	High
High	High	High	High	High	High	High	High	High	High
High	High	High	High	High	High	High	High	High	High
High	High	High	High	High	High	High	High	High	High
High	High	High	High	High	High	High	High	High	High

High	High	High	High	High	High	High	High	High	High
High	High	High	High	High	High	High	High	High	High
High	High	High	High	High	High	High	High	High	High
High	High	High	High	High	High	High	High	High	High
High	High	High	High	High	High	High	High	High	High
High	High	High	High	High	High	High	High	High	High
High	High	High	High	High	High	High	High	High	High
High	High	High	High	High	High	High	High	High	High
High	High	High	High	High	High	High	High	High	High
High	High	High	High	High	High	High	High	High	High
High	High	High	High	High	High	High	High	High	High
High	High	High	High	High	High	High	High	High	High
High	High	High	High	High	High	High	High	High	High
High	High	High	High	High	High	High	High	High	High
High	High	High	High	High	High	High	High	High	High
High	High	High	High	High	High	High	High	High	High
High	High	High	High	High	High	High	High	High	High
High	High	High	High	High	High	High	High	High	High
High	High	High	High	High	High	High	High	High	High
High	High	High	High	High	High	High	High	High	High
High	High	High	High	High	High	High	High	High	High
High	High	High	High	High	High	High	High	High	High
High	High	High	High	High	High	High	High	High	High
High	High	High	High	High	High	High	High	High	High
High	High	High	High	High	High	High	High	High	High
High	High	High	High	High	High	High	High	High	High
High	High	High	High	High	High	High	High	High	High
High	High	High	High	High	High	High	High	High	High
High	High	High	High	High	High	High	High	High	High
High	High	High	High	High	High	High	High	High	High
High	High	High	High	High	High	High	High	High	High
High	High	High	High	High	High	High	High	High	High
High	High	High	High	High	High	High	High	High	High
High	High	High	High	High	High	High	High	High	High
High	High	High	High	High	High	High	High	High	High
High	High	High	High	High	High	High	High	High	High
High	High	High	High	High	High	High	High	High	High
High	High	High	High	High	High	High	High	High	High
High	High	High	High	High	High	High	High	High	High
High	High	High	High	High	High	High	High	High	High
High	High	High	High	High	High	High	High	High	High
High	High	High	High	High	High	High	High	High	High
High	High	High	High	High	High	High	High	High	High
High	High	High	High	High	High	High	High	High	High
High	High	High	High	High	High	High	High	High	High
High	High	High	High	High	High	High	High	High	High
High	High	High	High	High	High	High	High	High	High
High	High	High	High	High	High	High	High	High	High
High	High	High	High	High	High	High	High	High	High
High	High	High	High	High	High	High	High	High	High

High	High	High	High	High	High	High	High	High	High
High	High	High	High	High	High	High	High	High	High
High	High	High	High	High	High	High	High	High	High
Not Found	Not Found	Not Found	High	High	High	High	High	High	High
High	High	High	High	High	High	High	High	High	High
High	High	High	High	High	High	High	Not Found	Not Found	Not Found
High	High	High	High	High	High	High	High	High	High
High	High	High	High	High	High	High	High	High	High
High	High	High	High	High	High	High	High	High	High
High	High	High	High	High	High	High	High	High	High
High	High	High	High	High	High	High	High	High	High
High	High	High	High	High	High	High	High	High	High
High	High	High	High	High	High	High	High	High	High
High	High	High	High	High	High	High	High	High	High
High	High	High	High	High	High	High	High	High	High
High	High	High	High	High	High	High	High	High	High
High	High	High	High	High	High	High	High	High	High
High	High	High	High	High	High	High	High	High	High
High	High	High	High	High	High	High	High	High	High
High	High	High	High	High	High	High	High	High	High
High	High	High	High	High	High	High	High	High	High
High	High	High	High	High	High	High	High	High	High
High	High	High	High	High	High	High	High	High	High
High	High	High	High	High	High	High	High	High	High
High	High	High	High	High	High	High	High	High	High
High	High	High	High	High	High	High	High	High	High
High	High	High	High	High	High	High	High	High	High
High	High	High	High	High	High	High	High	High	High
High	High	High	High	High	High	High	High	High	High
High	High	High	High	High	High	High	High	High	High
High	High	High	High	High	High	High	High	High	High
High	High	High	High	High	High	High	High	High	High
High	High	High	High	High	High	High	High	High	High
High	High	High	High	High	High	High	High	High	High
High	High	High	High	High	High	High	High	High	High
High	High	High	High	High	High	High	High	High	High
High	High	High	High	High	High	High	High	High	High
High	High	High	High	High	High	High	High	High	High
High	High	High	High	High	High	High	High	High	High
High	High	High	High	High	High	High	High	High	High
High	High	High	High	High	High	High	High	High	High
High	High	High	High	High	High	High	High	High	High
High	High	High	High	High	High	High	High	High	High
High	High	High	High	High	High	High	High	High	High
High	High	High	High	High	High	High	High	High	High
High	High	High	High	High	High	High	High	High	High
High	High	High	High	High	High	High	High	High	High
High	High	High	High	High	High	High	High	High	High
Not Found	Not Found	Not Found	High	High	High	High	Not Found	Not Found	Not Found
High	High	High	High	High	High	High	High	High	High

High	High	High	High	High	High	High	High	High	High
High	High	High	High	High	High	High	High	High	High
High	High	High	High	High	High	High	High	High	High
High	High	High	High	High	High	High	High	High	High
High	High	High	High	High	High	High	High	High	High
High	High	High	High	High	High	High	High	High	High
High	High	High	High	High	High	High	High	High	High
High	High	High	Not Found	Not Found	Not Found	Not Found	High	High	High
High	High	High	High	High	High	High	High	High	High
High	High	High	High	High	High	High	High	High	High
Not Found	Not Found	Not Found	High	High	High	High	High	High	High
High	High	High	Not Found	Not Found	Not Found	Not Found	High	High	High
High	High	High	High	High	High	High	High	High	High
High	High	High	High	High	High	High	High	High	High
High	High	High	High	High	High	High	High	High	High
High	High	High	High	High	High	High	High	High	High
High	High	High	High	High	High	High	High	High	High
High	High	High	High	High	High	High	High	High	High
High	High	High	High	High	High	High	High	High	High
High	High	High	High	High	High	High	High	High	High
High	High	High	High	High	High	High	High	High	High
High	High	High	High	High	High	High	High	High	High
Not Found	Not Found	Not Found	Not Found	Not Found	Not Found	Not Found	High	High	High
High	High	High	High	High	High	High	High	High	High
High	High	High	High	High	High	High	High	High	High
High	High	High	High	High	High	High	High	High	High
High	High	High	High	High	High	High	High	High	High
High	High	High	High	High	High	High	High	High	High
High	High	High	High	High	High	High	High	High	High
High	High	High	High	High	High	High	High	High	High
High	High	High	High	High	High	High	High	High	High
High	High	High	High	High	High	High	High	High	High
High	High	High	High	High	High	High	High	High	High
High	High	High	High	High	High	High	High	High	High
High	High	High	High	High	High	High	High	High	High
High	High	High	High	High	High	High	High	High	High
High	High	High	High	High	High	High	High	High	High
High	High	High	High	High	High	High	High	High	High
High	High	High	High	High	High	High	High	High	High
High	High	High	High	High	High	High	High	High	High
High	High	High	High	High	High	High	High	High	High
High	High	High	High	High	High	High	High	High	High
High	High	High	High	High	High	High	High	High	High
High	High	High	High	High	High	High	High	High	High
High	High	High	High	High	High	High	High	High	High
High	High	High	High	High	High	High	High	High	High
High	High	High	High	High	High	High	High	High	High
High	High	High	High	High	High	High	Not Found	Not Found	Not Found
High	High	High	High	High	High	High	High	High	High
High	High	High	High	High	High	High	High	High	High

High	High	High	High	High	High	High	High	High	High
High	High	High	High	High	High	High	High	High	High
High	High	High	High	High	High	High	High	High	High
High	High	High	High	High	High	High	High	High	High
High	High	High	High	High	High	High	High	High	High
High	High	High	High	High	High	High	High	High	High
High	High	High	High	High	High	High	Not Found	Not Found	Not Found
High	High	High	High	High	High	High	High	High	High
High	High	High	High	High	High	High	High	High	High
High	High	High	High	High	High	High	Not Found	Not Found	Not Found
High	High	High	High	High	High	High	High	High	High
High	High	High	High	High	High	High	High	High	High
High	High	High	High	High	High	High	High	High	High
High	High	High	High	High	High	High	High	High	High
Not Found	Not Found	Not Found	High	High	High	High	High	High	High
High	High	High	High	High	High	High	High	High	High
High	High	High	High	High	High	High	High	High	High
High	High	High	High	High	High	High	High	High	High
High	High	High	Not Found	Not Found	Not Found	Not Found	High	High	High
High	High	High	High	High	High	High	High	High	High
High	High	High	High	High	High	High	High	High	High
High	High	High	High	High	High	High	High	High	High
High	High	High	High	High	High	High	High	High	High
High	High	High	High	High	High	High	High	High	High
High	High	High	High	High	High	High	High	High	High
High	High	High	High	High	High	High	High	High	High
High	High	High	High	High	High	High	High	High	High
High	High	High	High	High	High	High	High	High	High
High	High	High	High	High	High	High	High	High	High
High	High	High	High	High	High	High	High	High	High
High	High	High	High	High	High	High	High	High	High
High	High	High	High	High	High	High	High	High	High
High	High	High	High	High	High	High	High	High	High
High	High	High	High	High	High	High	High	High	High
High	High	High	High	High	High	High	High	High	High
High	High	High	High	High	High	High	High	High	High
High	High	High	High	High	High	High	High	High	High
High	High	High	High	High	High	High	High	High	High
High	High	High	High	High	High	High	High	High	High
High	High	High	High	High	High	High	High	High	High
High	High	High	High	High	High	High	High	High	High
High	High	High	High	High	High	High	High	High	High
High	High	High	High	High	High	High	High	High	High
High	High	High	High	High	High	High	High	High	High
High	High	High	High	High	High	High	High	High	High
High	High	High	High	High	High	High	Not Found	Not Found	Not Found
High	High	High	High	High	High	High	High	High	High
High	High	High	High	High	High	High	High	High	High
High	High	High	High	High	High	High	High	High	High
High	High	High	High	High	High	High	High	High	High

High	High	High	Not Found	Not Found	Not Found	Not Found	Not Found	Not Found	Not Found
High	High	High	High	High	High	High	High	High	High
High	High	High	Not Found	Not Found	Not Found	Not Found	High	High	High
High	High	High	High	High	High	High	High	High	High
High	High	High	High	High	High	High	High	High	High
High	High	High	High	High	High	High	High	High	High
High	High	High	High	High	High	High	High	High	High
High	High	High	High	High	High	High	High	High	High
High	High	High	High	High	High	High	High	High	High
High	High	High	High	High	High	High	High	High	High
High	High	High	High	High	High	High	High	High	High
High	High	High	High	High	High	High	High	High	High
High	High	High	High	High	High	High	High	High	High
High	High	High	High	High	High	High	High	High	High
High	High	High	High	High	High	High	High	High	High
High	High	High	High	High	High	High	High	High	High
High	High	High	High	High	High	High	High	High	High
High	High	High	High	High	High	High	High	High	High
High	High	High	High	High	High	High	High	High	High
High	High	High	High	High	High	High	High	High	High
High	High	High	High	High	High	High	High	High	High
High	High	High	High	High	High	High	High	High	High
High	High	High	Not Found	Not Found	Not Found	Not Found	High	High	High
High	High	High	High	High	High	High	High	High	High
High	High	High	High	High	High	High	High	High	High
High	High	High	High	High	High	High	High	High	High
High	High	High	High	High	High	High	High	High	High
High	High	High	High	High	High	High	High	High	High
High	High	High	High	High	High	High	High	High	High
High	High	High	High	High	High	High	High	High	High
High	High	High	High	High	High	High	High	High	High
High	High	High	High	High	High	High	High	High	High
High	High	High	High	High	High	High	High	High	High
High	High	High	High	High	High	High	High	High	High
High	High	High	High	High	High	High	High	High	High
High	High	High	High	High	High	High	High	High	High
High	High	High	High	High	High	High	High	High	High
High	High	High	High	High	High	High	High	High	High
Not Found	Not Found	Not Found	High	High	High	High	High	High	High
High	High	High	High	High	High	High	High	High	High
High	High	High	High	High	High	High	High	High	High
High	High	High	High	High	High	High	High	High	High
High	High	High	High	High	High	High	High	High	High
High	High	High	High	High	High	High	High	High	High
High	High	High	High	High	High	High	High	High	High
High	High	High	High	High	High	High	High	High	High
High	High	High	High	High	High	High	High	High	High
High	High	High	High	High	High	High	High	High	High
High	High	High	High	High	High	High	High	High	High
High	High	High	High	High	High	High	High	High	High

High	High	High	High	High	High	High	High	High	High
High	High	High	High	High	High	High	High	High	High
High	High	High	High	High	High	High	High	High	High
High	High	High	High	High	High	High	High	High	High
High	High	High	High	High	High	High	High	High	High
High	High	High	High	High	High	High	High	High	High
High	High	High	High	High	High	High	High	High	High
High	High	High	Not Found	Not Found	Not Found	Not Found	High	High	High
High	High	High	High	High	High	High	High	High	High
High	High	High	High	High	High	High	High	High	High
High	High	High	High	High	High	High	High	High	High
High	High	High	High	High	High	High	High	High	High
High	High	High	High	High	High	High	High	High	High
High	High	High	High	High	High	High	High	High	High
High	High	High	High	High	High	High	High	High	High
High	High	High	High	High	High	High	High	High	High
High	High	High	High	High	High	High	High	High	High
High	High	High	High	High	High	High	High	High	High
High	High	High	High	High	High	High	High	High	High
High	High	High	High	High	High	High	High	High	High
High	High	High	High	High	High	High	High	High	High
High	High	High	High	High	High	High	High	High	High
High	High	High	High	High	High	High	High	High	High
High	High	High	High	High	High	High	High	High	High
High	High	High	High	High	High	High	High	High	High
High	High	High	High	High	High	High	High	High	High
High	High	High	High	High	High	High	High	High	High
High	High	High	High	High	High	High	High	High	High
High	High	High	High	High	High	High	High	High	High
High	High	High	High	High	High	High	High	High	High
High	High	High	High	High	High	High	High	High	High
High	High	High	High	High	High	High	High	High	High
High	High	High	Not Found	Not Found	Not Found	Not Found	High	High	High
High	High	High	High	High	High	High	High	High	High
Not Found	Not Found	Not Found	High	High	High	High	High	High	High
High	High	High	High	High	High	High	High	High	High
High	High	High	High	High	High	High	High	High	High
High	High	High	High	High	High	High	High	High	High
High	High	High	High	High	High	High	High	High	High
High	High	High	High	High	High	High	High	High	High
High	High	High	High	High	High	High	High	High	High
High	High	High	High	High	High	High	High	High	High
High	High	High	High	High	High	High	High	High	High
High	High	High	High	High	High	High	High	High	High
High	High	High	High	High	High	High	High	High	High
High	High	High	High	High	High	High	High	High	High
High	High	High	High	High	High	High	High	High	High
High	High	High	High	High	High	High	High	High	High
High	High	High	High	High	High	High	High	High	High
High	High	High	High	High	High	High	High	High	High

High	High	High	High	High	High	High	High	High	High
High	High	High	High	High	High	High	High	High	High
High	High	High	High	High	High	High	High	High	High
High	High	High	High	High	High	High	High	High	High
High	High	High	High	High	High	High	High	High	High
High	High	High	High	High	High	High	High	High	High
High	High	High	High	High	High	High	High	High	High
High	High	High	High	High	High	High	High	High	High
High	High	High	High	High	High	High	High	High	High
High	High	High	High	High	High	High	High	High	High
High	High	High	High	High	High	High	High	High	High
High	High	High	High	High	High	High	High	High	High
High	High	High	High	High	High	High	High	High	High
High	High	High	High	High	High	High	High	High	High
High	High	High	High	High	High	High	High	High	High
High	High	High	High	High	High	High	High	High	High
High	High	High	High	High	High	High	High	High	High
High	High	High	High	High	High	High	High	High	High
High	High	High	High	High	High	High	High	High	High
High	High	High	High	High	High	High	High	High	High
High	High	High	High	High	High	High	High	High	High
High	High	High	High	High	High	High	High	High	High
High	High	High	High	High	High	High	High	High	High
High	High	High	High	High	High	High	High	High	High
High	High	High	High	High	High	High	High	High	High
Not Found	Not Found	Not Found	Not Found	Not Found	Not Found	Not Found	High	High	High
High	High	High	High	High	High	High	High	High	High
High	High	High	High	High	High	High	High	High	High
High	High	High	High	High	High	High	High	High	High
High	High	High	High	High	High	High	High	High	High
High	High	High	High	High	High	High	High	High	High
High	High	High	High	High	High	High	High	High	High
High	High	High	High	High	High	High	High	High	High
High	High	High	High	High	High	High	High	High	High
High	High	High	High	High	High	High	High	High	High
High	High	High	High	High	High	High	High	High	High
High	High	High	High	High	High	High	High	High	High
High	High	High	High	High	High	High	High	High	High
High	High	High	High	High	High	High	High	High	High
High	High	High	High	High	High	High	High	High	High
High	High	High	High	High	High	High	High	High	High
High	High	High	High	High	High	High	High	High	High
High	High	High	High	High	High	High	High	High	High
High	High	High	High	High	High	High	High	High	High
High	High	High	High	High	High	High	High	High	High
High	High	High	High	High	High	High	High	High	High
High	High	High	High	High	High	High	High	High	High
High	High	High	High	High	High	High	High	High	High
High	High	High	High	High	High	High	High	High	High
High	High	High	High	High	High	High	High	High	High

High	High	High	Not Found	Not Found	Not Found	Not Found	High	High	High
High	High	High	High	High	High	High	High	High	High
High	High	High	High	High	High	High	High	High	High
High	High	High	High	High	High	High	High	High	High
High	High	High	High	High	High	High	High	High	High
High	High	High	High	High	High	High	High	High	High
High	High	High	High	High	High	High	High	High	High
High	High	High	High	High	High	High	High	High	High
High	High	High	High	High	High	High	High	High	High
High	High	High	High	High	High	High	High	High	High
High	High	High	High	High	High	High	High	High	High
High	High	High	High	High	High	High	High	High	High
High	High	High	High	High	High	High	High	High	High
High	High	High	High	High	High	High	Not Found	Not Found	Not Found
High	High	High	High	High	High	High	High	High	High
High	High	High	High	High	High	High	High	High	High
High	High	High	High	High	High	High	High	High	High
High	High	High	High	High	High	High	High	High	High
High	High	High	High	High	High	High	High	High	High
High	High	High	High	High	High	High	High	High	High
High	High	High	High	High	High	High	High	High	High
Not Found	Not Found	Not Found	High	High	High	High	High	High	High
High	High	High	High	High	High	High	High	High	High
High	High	High	High	High	High	High	High	High	High
High	High	High	High	High	High	High	High	High	High
Not Found	Not Found	Not Found	Not Found	Not Found	Not Found	Not Found	Not Found	Not Found	Not Found
High	High	High	High	High	High	High	High	High	High
High	High	High	High	High	High	High	High	High	High
High	High	High	High	High	High	High	High	High	High
High	High	High	High	High	High	High	High	High	High
High	High	High	High	High	High	High	High	High	High
High	High	High	High	High	High	High	High	High	High
High	High	High	High	High	High	High	High	High	High
High	High	High	High	High	High	High	High	High	High
High	High	High	Not Found	Not Found	Not Found	Not Found	High	High	High
High	High	High	High	High	High	High	High	High	High
High	High	High	High	High	High	High	High	High	High
High	High	High	High	High	High	High	High	High	High
High	High	High	High	High	High	High	High	High	High
High	High	High	High	High	High	High	High	High	High
High	High	High	High	High	High	High	High	High	High
Not Found	Not Found	Not Found	High	High	High	High	High	High	High
High	High	High	High	High	High	High	High	High	High
High	High	High	High	High	High	High	High	High	High
High	High	High	High	High	High	High	High	High	High
High	High	High	High	High	High	High	High	High	High
High	High	High	High	High	High	High	High	High	High
High	High	High	High	High	High	High	High	High	High
High	High	High	High	High	High	High	High	High	High
High	High	High	High	High	High	High	High	High	High

High	High	High	High	High	High	High	High	High	High
High	High	High	High	High	High	High	High	High	High
High	High	High	High	High	High	High	High	High	High
High	High	High	High	High	High	High	High	High	High
High	High	High	High	High	High	High	High	High	High
High	High	High	High	High	High	High	High	High	High
High	High	High	High	High	High	High	High	High	High
High	High	High	High	High	High	High	High	High	High
High	High	High	High	High	High	High	High	High	High
High	High	High	High	High	High	High	High	High	High
High	High	High	High	High	High	High	High	High	High
High	High	High	High	High	High	High	High	High	High
High	High	High	High	High	High	High	High	High	High
High	High	High	High	High	High	High	High	High	High
High	High	High	High	High	High	High	Not Found	Not Found	Not Found
Not Found	Not Found	Not Found	High	High	High	High	High	High	High
High	High	High	High	High	High	High	High	High	High
High	High	High	High	High	High	High	High	High	High
High	High	High	High	High	High	High	High	High	High
High	High	High	High	High	High	High	High	High	High
High	High	High	High	High	High	High	Not Found	Not Found	Not Found
High	High	High	High	High	High	High	High	High	High
High	High	High	Not Found	Not Found	Not Found	Not Found	High	High	High
High	High	High	High	High	High	High	High	High	High
High	High	High	High	High	High	High	High	High	High
High	High	High	High	High	High	High	High	High	High
High	High	High	High	High	High	High	High	High	High
High	High	High	High	High	High	High	High	High	High
High	High	High	High	High	High	High	High	High	High
High	High	High	High	High	High	High	High	High	High
High	High	High	High	High	High	High	High	High	High
High	High	High	High	High	High	High	High	High	High
High	High	High	High	High	High	High	High	High	High
High	High	High	High	High	High	High	High	High	High
High	High	High	High	High	High	High	High	High	High
High	High	High	High	High	High	High	High	High	High
High	High	High	High	High	High	High	High	High	High
High	High	High	High	High	High	High	High	High	High
High	High	High	High	High	High	High	High	High	High
High	High	High	High	High	High	High	High	High	High
High	High	High	High	High	High	High	High	High	High
High	High	High	High	High	High	High	High	High	High
High	High	High	High	High	High	High	High	High	High
High	High	High	High	High	High	High	High	High	High
High	High	High	Not Found	Not Found	Not Found	Not Found	High	High	High
High	High	High	High	High	High	High	High	High	High
High	High	High	High	High	High	High	High	High	High
High	High	High	High	High	High	High	High	High	High
High	High	High	High	High	High	High	High	High	High
High	High	High	High	High	High	High	High	High	High

High	High	High	High	High	High	High	High	High	High
High	High	High	High	High	High	High	High	High	High
High	High	High	High	High	High	High	High	High	High
High	High	High	High	High	High	High	High	High	High
High	High	High	High	High	High	High	High	High	High
High	High	High	High	High	High	High	High	High	High
Not Found	Not Found	Not Found	High	High	High	High	High	High	High
High	High	High	High	High	High	High	High	High	High
High	High	High	Not Found	Not Found	Not Found	Not Found	High	High	High
High	High	High	High	High	High	High	High	High	High
High	High	High	High	High	High	High	High	High	High
High	High	High	High	High	High	High	High	High	High
High	High	High	High	High	High	High	High	High	High
High	High	High	High	High	High	High	High	High	High
High	High	High	High	High	High	High	High	High	High
High	High	High	High	High	High	High	High	High	High
High	High	High	High	High	High	High	High	High	High
High	High	High	High	High	High	High	High	High	High
High	High	High	High	High	High	High	High	High	High
High	High	High	High	High	High	High	High	High	High
High	High	High	High	High	High	High	High	High	High
High	High	High	High	High	High	High	High	High	High
High	High	High	High	High	High	High	High	High	High
High	High	High	High	High	High	High	High	High	High
High	High	High	High	High	High	High	High	High	High
High	High	High	High	High	High	High	High	High	High
High	High	High	High	High	High	High	High	High	High
High	High	High	High	High	High	High	High	High	High
High	High	High	High	High	High	High	High	High	High
High	High	High	High	High	High	High	High	High	High
High	High	High	High	High	High	High	High	High	High
High	High	High	High	High	High	High	High	High	High
High	High	High	High	High	High	High	High	High	High
High	High	High	High	High	High	High	High	High	High
High	High	High	High	High	High	High	High	High	High
High	High	High	Not Found	Not Found	Not Found	Not Found	High	High	High
High	High	High	High	High	High	High	High	High	High
High	High	High	High	High	High	High	High	High	High
High	High	High	High	High	High	High	High	High	High
High	High	High	High	High	High	High	High	High	High
High	High	High	High	High	High	High	High	High	High
High	High	High	High	High	High	High	High	High	High
High	High	High	High	High	High	High	High	High	High
High	High	High	High	High	High	High	High	High	High
High	High	High	High	High	High	High	High	High	High
High	High	High	High	High	High	High	High	High	High
High	High	High	High	High	High	High	High	High	High
High	High	High	High	High	High	High	Not Found	Not Found	Not Found
High	High	High	High	High	High	High	High	High	High
High	High	High	High	High	High	High	High	High	High

High	High	High	High	High	High	High	High	High	High
High	High	High	High	High	High	High	High	High	High
High	High	High	High	High	High	High	High	High	High
High	High	High	High	High	High	High	High	High	High
High	High	High	High	High	High	High	High	High	High
High	High	High	High	High	High	High	High	High	High
High	High	High	High	High	High	High	High	High	High
High	High	High	High	High	High	High	High	High	High
High	High	High	High	High	High	High	High	High	High
High	High	High	High	High	High	High	High	High	High
High	High	High	High	High	High	High	High	High	High
High	High	High	High	High	High	High	Not Found	Not Found	Not Found
Not Found	Not Found	Not Found	High	High	High	High	High	High	High
High	High	High	High	High	High	High	High	High	High
High	High	High	High	High	High	High	High	High	High
Not Found	Not Found	Not Found	Not Found	Not Found	Not Found	Not Found	High	High	High
High	High	High	Not Found	Not Found	Not Found	Not Found	High	High	High
High	High	High	High	High	High	High	High	High	High
High	High	High	High	High	High	High	High	High	High
High	High	High	High	High	High	High	High	High	High
High	High	High	High	High	High	High	High	High	High
High	High	High	High	High	High	High	High	High	High
High	High	High	High	High	High	High	High	High	High
High	High	High	High	High	High	High	High	High	High
High	High	High	High	High	High	High	High	High	High
High	High	High	High	High	High	High	High	High	High
High	High	High	High	High	High	High	High	High	High
High	High	High	Not Found	Not Found	Not Found	Not Found	High	High	High
High	High	High	High	High	High	High	High	High	High
High	High	High	High	High	High	High	High	High	High
High	High	High	High	High	High	High	High	High	High
High	High	High	High	High	High	High	High	High	High
High	High	High	High	High	High	High	Not Found	Not Found	Not Found
High	High	High	High	High	High	High	High	High	High
High	High	High	High	High	High	High	High	High	High
High	High	High	Not Found	Not Found	Not Found	Not Found	High	High	High
High	High	High	High	High	High	High	High	High	High
High	High	High	High	High	High	High	High	High	High
High	High	High	Not Found	Not Found	Not Found	Not Found	High	High	High
High	High	High	Not Found	Not Found	Not Found	Not Found	High	High	High
High	High	High	High	High	High	High	High	High	High
High	High	High	High	High	High	High	High	High	High
High	High	High	High	High	High	High	High	High	High
High	High	High	High	High	High	High	High	High	High
High	High	High	High	High	High	High	High	High	High
High	High	High	High	High	High	High	High	High	High
High	High	High	High	High	High	High	High	High	High
Not Found	Not Found	Not Found	High	High	High	High	High	High	High
High	High	High	High	High	High	High	High	High	High
High	High	High	High	High	High	High	High	High	High

High	High	High	High	High	High	High	High	High	High
High	High	High	High	High	High	High	High	High	High
High	High	High	High	High	High	High	High	High	High
High	High	High	High	High	High	High	High	High	High
High	High	High	High	High	High	High	High	High	High
High	High	High	High	High	High	High	High	High	High
High	High	High	High	High	High	High	Not Found	Not Found	Not Found
High	High	High	High	High	High	High	High	High	High
High	High	High	High	High	High	High	High	High	High
High	High	High	High	High	High	High	High	High	High
High	High	High	Not Found	Not Found	Not Found	Not Found	High	High	High
Not Found	Not Found	Not Found	High	High	High	High	High	High	High
High	High	High	High	High	High	High	Not Found	Not Found	Not Found
High	High	High	High	High	High	High	High	High	High
High	High	High	High	High	High	High	High	High	High
High	High	High	High	High	High	High	High	High	High
High	High	High	High	High	High	High	High	High	High
High	High	High	Not Found	Not Found	Not Found	Not Found	High	High	High
High	High	High	High	High	High	High	High	High	High
High	High	High	High	High	High	High	High	High	High
High	High	High	High	High	High	High	High	High	High
High	High	High	High	High	High	High	Not Found	Not Found	Not Found
High	High	High	High	High	High	High	High	High	High
High	High	High	High	High	High	High	High	High	High
High	High	High	High	High	High	High	High	High	High
High	High	High	High	High	High	High	High	High	High
High	High	High	High	High	High	High	High	High	High
High	High	High	High	High	High	High	Not Found	Not Found	Not Found
High	High	High	High	High	High	High	High	High	High
High	High	High	High	High	High	High	High	High	High
High	High	High	High	High	High	High	High	High	High
High	High	High	High	High	High	High	High	High	High
High	High	High	High	High	High	High	High	High	High
High	High	High	High	High	High	High	High	High	High
High	High	High	High	High	High	High	Not Found	Not Found	Not Found
High	High	High	High	High	High	High	High	High	High
Not Found	Not Found	Not Found	High	High	High	High	High	High	High
High	High	High	High	High	High	High	Not Found	Not Found	Not Found
High	High	High	High	High	High	High	High	High	High
High	High	High	High	High	High	High	High	High	High
High	High	High	High	High	High	High	High	High	High
High	High	High	High	High	High	High	High	High	High
High	High	High	High	High	High	High	High	High	High
High	High	High	High	High	High	High	High	High	High
High	High	High	High	High	High	High	High	High	High
High	High	High	High	High	High	High	Not Found	Not Found	Not Found
High	High	High	High	High	High	High	High	High	High
High	High	High	High	High	High	High	High	High	High
High	High	High	High	High	High	High	High	High	High
High	High	High	High	High	High	High	High	High	High

High	High	High	Not Found	Not Found	Not Found	Not Found	Not Found	Not Found	Not Found
High	High	High	High	High	High	High	High	High	High
High	High	High	High	High	High	High	High	High	High
High	High	High	High	High	High	High	High	High	High
High	High	High	High	High	High	High	High	High	High
Not Found	Not Found	Not Found	High	High	High	High	Not Found	Not Found	Not Found
Not Found	Not Found	Not Found	High	High	High	High	High	High	High
High	High	High	High	High	High	High	High	High	High
High	High	High	High	High	High	High	High	High	High
High	High	High	High	High	High	High	Not Found	Not Found	Not Found
High	High	High	High	High	High	High	High	High	High
High	High	High	High	High	High	High	High	High	High
High	High	High	High	High	High	High	High	High	High
High	High	High	High	High	High	High	High	High	High
High	High	High	High	High	High	High	High	High	High
Not Found	Not Found	Not Found	High	High	High	High	High	High	High
High	High	High	High	High	High	High	High	High	High
High	High	High	High	High	High	High	High	High	High
High	High	High	High	High	High	High	Not Found	Not Found	Not Found
High	High	High	High	High	High	High	High	High	High
High	High	High	High	High	High	High	High	High	High
High	High	High	High	High	High	High	High	High	High
High	High	High	High	High	High	High	High	High	High
High	High	High	High	High	High	High	High	High	High
High	High	High	High	High	High	High	High	High	High
Not Found	Not Found	Not Found	High	High	High	High	Not Found	Not Found	Not Found
High	High	High	High	High	High	High	High	High	High
High	High	High	High	High	High	High	High	High	High
High	High	High	High	High	High	High	High	High	High
Not Found	Not Found	Not Found	High	High	High	High	High	High	High
High	High	High	High	High	High	High	High	High	High
High	High	High	High	High	High	High	High	High	High
High	High	High	High	High	High	High	High	High	High
High	High	High	High	High	High	High	High	High	High
High	High	High	High	High	High	High	High	High	High
High	High	High	High	High	High	High	High	High	High
High	High	High	High	High	High	High	Not Found	Not Found	Not Found
High	High	High	High	High	High	High	High	High	High
High	High	High	High	High	High	High	High	High	High
High	High	High	High	High	High	High	High	High	High
High	High	High	High	High	High	High	High	High	High
Not Found	Not Found	Not Found	High	High	High	High	High	High	High
High	High	High	High	High	High	High	Not Found	Not Found	Not Found
High	High	High	Not Found	Not Found	Not Found	Not Found	High	High	High
Not Found	Not Found	Not Found	High	High	High	High	Not Found	Not Found	Not Found
High	High	High	High	High	High	High	High	High	High
High	High	High	High	High	High	High	High	High	High
High	High	High	High	High	High	High	High	High	High
High	High	High	Not Found	Not Found	Not Found	Not Found	High	High	High
High	High	High	High	High	High	High	High	High	High

High	High	High	High	High	High	High	High	High	High
High	High	High	High	High	High	High	High	High	High
High	High	High	High	High	High	High	High	High	High
High	High	High	High	High	High	High	High	High	High
High	High	High	High	High	High	High	High	High	High
High	High	High	Not Found	Not Found	Not Found	Not Found	Not Found	Not Found	Not Found
High	High	High	High	High	High	High	High	High	High
Not Found	Not Found	Not Found	High	High	High	High	High	High	High
Not Found	Not Found	Not Found	High	High	High	High	High	High	High
High	High	High	High	High	High	High	Not Found	Not Found	Not Found
High	High	High	High	High	High	High	High	High	High
High	High	High	High	High	High	High	High	High	High
High	High	High	High	High	High	High	Not Found	Not Found	Not Found
High	High	High	Not Found	Not Found	Not Found	Not Found	High	High	High
High	High	High	High	High	High	High	High	High	High
Not Found	Not Found	Not Found	High	High	High	High	High	High	High
High	High	High	High	High	High	High	Not Found	Not Found	Not Found
High	High	High	High	High	High	High	High	High	High
High	High	High	High	High	High	High	High	High	High
High	High	High	High	High	High	High	High	High	High
High	High	High	Not Found	Not Found	Not Found	Not Found	High	High	High
High	High	High	High	High	High	High	High	High	High
High	High	High	High	High	High	High	High	High	High
High	High	High	High	High	High	High	High	High	High
High	High	High	High	High	High	High	High	High	High
High	High	High	High	High	High	High	High	High	High
High	High	High	High	High	High	High	High	High	High
High	High	High	High	High	High	High	High	High	High
High	High	High	High	High	High	High	High	High	High
High	High	High	High	High	High	High	High	High	High
High	High	High	High	High	High	High	High	High	High
High	High	High	High	High	High	High	High	High	High
High	High	High	High	High	High	High	High	High	High
High	High	High	High	High	High	High	High	High	High
High	High	High	High	High	High	High	High	High	High
Not Found	Not Found	Not Found	High	High	High	High	High	High	High
Not Found	Not Found	Not Found	High	High	High	High	High	High	High
High	High	High	High	High	High	High	Not Found	Not Found	Not Found
High	High	High	High	High	High	High	High	High	High
High	High	High	High	High	High	High	High	High	High
Not Found	Not Found	Not Found	High	High	High	High	High	High	High
High	High	High	High	High	High	High	Not Found	Not Found	Not Found
High	High	High	Not Found	Not Found	Not Found	Not Found	High	High	High
High	High	High	High	High	High	High	High	High	High
High	High	High	High	High	High	High	High	High	High
High	High	High	High	High	High	High	High	High	High
High	High	High	High	High	High	High	High	High	High
High	High	High	High	High	High	High	High	High	High
High	High	High	High	High	High	High	High	High	High
Not Found	Not Found	Not Found	High	High	High	High	High	High	High

High	High	High	High	High	High	High	Not Found	Not Found	Not Found
High	High	High	High	High	High	High	High	High	High
High	High	High	Not Found	Not Found	Not Found	Not Found	High	High	High
High	High	High	High	High	High	High	High	High	High
High	High	High	High	High	High	High	High	High	High
High	High	High	High	High	High	High	High	High	High
High	High	High	High	High	High	High	High	High	High
High	High	High	High	High	High	High	High	High	High
High	High	High	High	High	High	High	High	High	High
High	High	High	High	High	High	High	High	High	High
High	High	High	Not Found	Not Found	Not Found	Not Found	High	High	High
High	High	High	High	High	High	High	High	High	High
High	High	High	High	High	High	High	High	High	High
High	High	High	High	High	High	High	High	High	High
High	High	High	High	High	High	High	High	High	High
High	High	High	High	High	High	High	High	High	High
High	High	High	High	High	High	High	High	High	High
High	High	High	High	High	High	High	High	High	High
High	High	High	Not Found	Not Found	Not Found	Not Found	High	High	High
High	High	High	High	High	High	High	High	High	High
High	High	High	High	High	High	High	High	High	High
High	High	High	High	High	High	High	High	High	High
High	High	High	High	High	High	High	Not Found	Not Found	Not Found
High	High	High	High	High	High	High	High	High	High
High	High	High	High	High	High	High	Not Found	Not Found	Not Found
High	High	High	High	High	High	High	High	High	High
High	High	High	High	High	High	High	Not Found	Not Found	Not Found
Not Found	Not Found	Not Found	High	High	High	High	High	High	High
High	High	High	High	High	High	High	High	High	High
High	High	High	High	High	High	High	High	High	High
High	High	High	High	High	High	High	High	High	High
High	High	High	High	High	High	High	High	High	High
High	High	High	High	High	High	High	High	High	High
High	High	High	High	High	High	High	High	High	High
High	High	High	High	High	High	High	High	High	High
High	High	High	High	High	High	High	Not Found	Not Found	Not Found
High	High	High	High	High	High	High	Not Found	Not Found	Not Found
High	High	High	High	High	High	High	High	High	High
Not Found	Not Found	Not Found	High	High	High	High	High	High	High
High	High	High	High	High	High	High	High	High	High
High	High	High	High	High	High	High	Not Found	Not Found	Not Found
High	High	High	High	High	High	High	Not Found	Not Found	Not Found
High	High	High	High	High	High	High	High	High	High
High	High	High	High	High	High	High	High	High	High
Not Found	Not Found	Not Found	High	High	High	High	High	High	High
High	High	High	High	High	High	High	High	High	High
High	High	High	High	High	High	High	High	High	High
High	High	High	High	High	High	High	High	High	High
High	High	High	High	High	High	High	High	High	High
High	High	High	High	High	High	High	High	High	High

High	High	High	High	High	High	High	High	High	High
High	High	High	High	High	High	High	High	High	High
High	High	High	High	High	High	High	High	High	High
High	High	High	High	High	High	High	High	High	High
High	High	High	High	High	High	High	High	High	High
High	High	High	High	High	High	High	High	High	High
High	High	High	High	High	High	High	High	High	High
High	High	High	High	High	High	High	Not Found	Not Found	Not Found
High	High	High	Not Found	Not Found	Not Found	Not Found	Not Found	Not Found	Not Found
High	High	High	High	High	High	High	High	High	High
High	High	High	Not Found	Not Found	Not Found	Not Found	High	High	High
High	High	High	High	High	High	High	High	High	High
High	High	High	Not Found	Not Found	Not Found	Not Found	High	High	High
High	High	High	High	High	High	High	High	High	High
Not Found	Not Found	Not Found	High	High	High	High	Not Found	Not Found	Not Found
High	High	High	Not Found	Not Found	Not Found	Not Found	High	High	High
High	High	High	High	High	High	High	High	High	High
High	High	High	Not Found	Not Found	Not Found	Not Found	Not Found	Not Found	Not Found
High	High	High	High	High	High	High	High	High	High
High	High	High	High	High	High	High	High	High	High
High	High	High	High	High	High	High	Not Found	Not Found	Not Found
Not Found	Not Found	Not Found	High	High	High	High	High	High	High
High	High	High	High	High	High	High	High	High	High
High	High	High	High	High	High	High	Not Found	Not Found	Not Found
High	High	High	High	High	High	High	High	High	High
High	High	High	Not Found	Not Found	Not Found	Not Found	High	High	High
High	High	High	High	High	High	High	High	High	High
High	High	High	Not Found	Not Found	Not Found	Not Found	High	High	High
High	High	High	High	High	High	High	Not Found	Not Found	Not Found
High	High	High	High	High	High	High	High	High	High
High	High	High	Not Found	Not Found	Not Found	Not Found	High	High	High
High	High	High	Not Found	Not Found	Not Found	Not Found	High	High	High
Not Found	Not Found	Not Found	High	High	High	High	High	High	High
High	High	High	High	High	High	High	Not Found	Not Found	Not Found
High	High	High	High	High	High	High	High	High	High
High	High	High	High	High	High	High	High	High	High
High	High	High	Not Found	Not Found	Not Found	Not Found	High	High	High
High	High	High	High	High	High	High	High	High	High
Not Found	Not Found	Not Found	High	High	High	High	High	High	High
High	High	High	High	High	High	High	Not Found	Not Found	Not Found
Not Found	Not Found	Not Found	High	High	High	High	High	High	High
High	High	High	High	High	High	High	High	High	High
High	High	High	Not Found	Not Found	Not Found	Not Found	High	High	High
High	High	High	High	High	High	High	High	High	High
Not Found	Not Found	Not Found	High	High	High	High	High	High	High
High	High	High	Not Found	Not Found	Not Found	Not Found	High	High	High
Not Found	Not Found	Not Found	High	High	High	High	High	High	High
High	High	High	High	High	High	High	High	High	High
High	High	High	High	High	High	High	High	High	High
High	High	High	Not Found	Not Found	Not Found	Not Found	High	High	High

High	High	High	High	High	High	High	High	High	High
High	High	High	High	High	High	High	High	High	High
Not Found	Not Found	Not Found	High	High	High	High	High	High	High
High	High	High	High	High	High	High	High	High	High
High	High	High	High	High	High	High	High	High	High
High	High	High	High	High	High	High	High	High	High
Not Found	Not Found	Not Found	High	High	High	High	High	High	High
High	High	High	Not Found	Not Found	Not Found	Not Found	High	High	High
Not Found	Not Found	Not Found	High	High	High	High	High	High	High
High	High	High	High	High	High	High	High	High	High
High	High	High	High	High	High	High	High	High	High
High	High	High	High	High	High	High	Not Found	Not Found	Not Found
High	High	High	Not Found	Not Found	Not Found	Not Found	High	High	High
High	High	High	High	High	High	High	Not Found	Not Found	Not Found
High	High	High	Not Found	Not Found	Not Found	Not Found	Not Found	Not Found	Not Found
High	High	High	High	High	High	High	High	High	High
Not Found	Not Found	Not Found	High	High	High	High	High	High	High
High	High	High	High	High	High	High	High	High	High
High	High	High	High	High	High	High	High	High	High
High	High	High	High	High	High	High	High	High	High
High	High	High	High	High	High	High	Not Found	Not Found	Not Found
High	High	High	High	High	High	High	High	High	High
High	High	High	High	High	High	High	High	High	High
High	High	High	High	High	High	High	High	High	High
High	High	High	High	High	High	High	High	High	High
High	High	High	High	High	High	High	High	High	High
High	High	High	Not Found	Not Found	Not Found	Not Found	High	High	High
High	High	High	Not Found	Not Found	Not Found	Not Found	High	High	High
Not Found	Not Found	Not Found	High	High	High	High	High	High	High
High	High	High	High	High	High	High	High	High	High
High	High	High	High	High	High	High	High	High	High
High	High	High	Not Found	Not Found	Not Found	Not Found	High	High	High
High	High	High	High	High	High	High	High	High	High
High	High	High	High	High	High	High	High	High	High
Not Found	Not Found	Not Found	High	High	High	High	High	High	High
High	High	High	High	High	High	High	High	High	High
High	High	High	High	High	High	High	High	High	High
Not Found	Not Found	Not Found	High	High	High	High	High	High	High
High	High	High	High	High	High	High	High	High	High
High	High	High	High	High	High	High	High	High	High
High	High	High	High	High	High	High	High	High	High
High	High	High	High	High	High	High	High	High	High
High	High	High	High	High	High	High	High	High	High
High	High	High	High	High	High	High	High	High	High
High	High	High	High	High	High	High	High	High	High
High	High	High	High	High	High	High	High	High	High
High	High	High	High	High	High	High	High	High	High
High	High	High	Not Found	Not Found	Not Found	Not Found	High	High	High
High	High	High	High	High	High	High	High	High	High
High	High	High	High	High	High	High	High	High	High

Not Found	Not Found	Not Found	High	High	High	High	High	High	High
High	High	High	High	High	High	High	High	High	High
High	High	High	Not Found	Not Found	Not Found	Not Found	High	High	High
High	High	High	High	High	High	High	High	High	High
High	High	High	High	High	High	High	High	High	High
Not Found	Not Found	Not Found	High	High	High	High	High	High	High
High	High	High	High	High	High	High	Not Found	Not Found	Not Found
High	High	High	High	High	High	High	High	High	High
High	High	High	High	High	High	High	High	High	High
High	High	High	Not Found	Not Found	Not Found	Not Found	High	High	High
High	High	High	High	High	High	High	High	High	High
High	High	High	High	High	High	High	High	High	High
High	High	High	Not Found	Not Found	Not Found	Not Found	Not Found	Not Found	Not Found
High	High	High	High	High	High	High	High	High	High
High	High	High	High	High	High	High	High	High	High
High	High	High	High	High	High	High	High	High	High
High	High	High	High	High	High	High	High	High	High
High	High	High	High	High	High	High	High	High	High
Not Found	Not Found	Not Found	High	High	High	High	High	High	High
High	High	High	High	High	High	High	High	High	High
High	High	High	High	High	High	High	Not Found	Not Found	Not Found
High	High	High	High	High	High	High	High	High	High
High	High	High	High	High	High	High	High	High	High
High	High	High	High	High	High	High	High	High	High
High	High	High	High	High	High	High	Not Found	Not Found	Not Found
High	High	High	High	High	High	High	High	High	High
Not Found	Not Found	Not Found	High	High	High	High	High	High	High
High	High	High	High	High	High	High	Not Found	Not Found	Not Found
Not Found	Not Found	Not Found	High	High	High	High	High	High	High
Not Found	Not Found	Not Found	Not Found	Not Found	Not Found	Not Found	Not Found	Not Found	Not Found
High	High	High	High	High	High	High	High	High	High
Not Found	Not Found	Not Found	Not Found	Not Found	Not Found	Not Found	High	High	High
Not Found	Not Found	Not Found	High	High	High	High	High	High	High
Not Found	Not Found	Not Found	High	High	High	High	High	High	High
High	High	High	High	High	High	High	High	High	High
Not Found	Not Found	Not Found	High	High	High	High	High	High	High
High	High	High	High	High	High	High	High	High	High
High	High	High	Not Found	Not Found	Not Found	Not Found	High	High	High
High	High	High	High	High	High	High	High	High	High
High	High	High	High	High	High	High	High	High	High
High	High	High	High	High	High	High	High	High	High
High	High	High	High	High	High	High	High	High	High
High	High	High	High	High	High	High	Not Found	Not Found	Not Found
High	High	High	High	High	High	High	High	High	High
High	High	High	High	High	High	High	High	High	High
High	High	High	High	High	High	High	High	High	High
Not Found	Not Found	Not Found	Not Found	Not Found	Not Found	Not Found	High	High	High
High	High	High	High	High	High	High	High	High	High
High	High	High	High	High	High	High	High	High	High
High	High	High	High	High	High	High	Not Found	Not Found	Not Found

High	High	High	High	High	High	High	High	High	High
High	High	High	High	High	High	High	High	High	High
High	High	High	High	High	High	High	High	High	High
Not Found	Not Found	Not Found	High	High	High	High	High	High	High
High	High	High	Not Found	Not Found	Not Found	Not Found	High	High	High
High	High	High	High	High	High	High	High	High	High
Not Found	Not Found	Not Found	High	High	High	High	High	High	High
High	High	High	High	High	High	High	Not Found	Not Found	Not Found
High	High	High	Not Found	Not Found	Not Found	Not Found	High	High	High
Not Found	Not Found	Not Found	Not Found	Not Found	Not Found	Not Found	High	High	High
Not Found	Not Found	Not Found	High	High	High	High	High	High	High
High	High	High	High	High	High	High	High	High	High
High	High	High	High	High	High	High	High	High	High
High	High	High	High	High	High	High	Not Found	Not Found	Not Found
High	High	High	High	High	High	High	High	High	High
High	High	High	High	High	High	High	High	High	High
High	High	High	High	High	High	High	High	High	High
High	High	High	High	High	High	High	High	High	High
Not Found	Not Found	Not Found	High	High	High	High	High	High	High
High	High	High	High	High	High	High	High	High	High
High	High	High	High	High	High	High	High	High	High
High	High	High	Not Found	Not Found	Not Found	Not Found	High	High	High
High	High	High	High	High	High	High	High	High	High
High	High	High	High	High	High	High	High	High	High
High	High	High	Not Found	Not Found	Not Found	Not Found	High	High	High
Not Found	Not Found	Not Found	High	High	High	High	Not Found	Not Found	Not Found
High	High	High	High	High	High	High	High	High	High
High	High	High	High	High	High	High	High	High	High
High	High	High	High	High	High	High	Not Found	Not Found	Not Found
High	High	High	High	High	High	High	High	High	High
High	High	High	High	High	High	High	High	High	High
High	High	High	Not Found	Not Found	Not Found	Not Found	High	High	High
High	High	High	High	High	High	High	Not Found	Not Found	Not Found
High	High	High	High	High	High	High	High	High	High
High	High	High	High	High	High	High	High	High	High
High	High	High	High	High	High	High	High	High	High
High	High	High	High	High	High	High	Not Found	Not Found	Not Found
High	High	High	High	High	High	High	Not Found	Not Found	Not Found
Not Found	Not Found	Not Found	High	High	High	High	High	High	High
Not Found	Not Found	Not Found	High	High	High	High	High	High	High
High	High	High	High	High	High	High	High	High	High
High	High	High	High	High	High	High	High	High	High
High	High	High	High	High	High	High	High	High	High
Not Found	Not Found	Not Found	Not Found	Not Found	Not Found	Not Found	High	High	High
High	High	High	High	High	High	High	High	High	High
High	High	High	High	High	High	High	High	High	High
High	High	High	High	High	High	High	Not Found	Not Found	Not Found
High	High	High	High	High	High	High	High	High	High
High	High	High	Not Found	Not Found	Not Found	Not Found	High	High	High
High	High	High	Not Found	Not Found	Not Found	Not Found	High	High	High

High	High	High	High	High	High	High	Not Found	Not Found	Not Found
High	High	High	Not Found	Not Found	Not Found	Not Found	High	High	High
High	High	High	High	High	High	High	High	High	High
High	High	High	High	High	High	High	Not Found	Not Found	Not Found
High	High	High	High	High	High	High	High	High	High
High	High	High	Not Found	Not Found	Not Found	Not Found	High	High	High
High	High	High	Not Found	Not Found	Not Found	Not Found	Not Found	Not Found	Not Found
Not Found	Not Found	Not Found	High	High	High	High	High	High	High
Not Found	Not Found	Not Found	High	High	High	High	High	High	High
Not Found	Not Found	Not Found	High	High	High	High	Not Found	Not Found	Not Found
High	High	High	High	High	High	High	Not Found	Not Found	Not Found
High	High	High	High	High	High	High	High	High	High
High	High	High	High	High	High	High	Not Found	Not Found	Not Found
High	High	High	High	High	High	High	High	High	High
High	High	High	Not Found	Not Found	Not Found	Not Found	High	High	High
High	High	High	High	High	High	High	High	High	High
Not Found	Not Found	Not Found	High	High	High	High	High	High	High
Not Found	Not Found	Not Found	High	High	High	High	High	High	High
High	High	High	High	High	High	High	High	High	High
Not Found	Not Found	Not Found	High	High	High	High	High	High	High
High	High	High	High	High	High	High	Not Found	Not Found	Not Found
High	High	High	High	High	High	High	High	High	High
High	High	High	High	High	High	High	High	High	High
Not Found	Not Found	Not Found	High	High	High	High	High	High	High
High	High	High	High	High	High	High	High	High	High
Not Found	Not Found	Not Found	High	High	High	High	High	High	High
High	High	High	High	High	High	High	Not Found	Not Found	Not Found
High	High	High	Not Found	Not Found	Not Found	Not Found	High	High	High
High	High	High	Not Found	Not Found	Not Found	Not Found	High	High	High
High	High	High	High	High	High	High	High	High	High
High	High	High	High	High	High	High	Not Found	Not Found	Not Found
High	High	High	High	High	High	High	High	High	High
Not Found	Not Found	Not Found	High	High	High	High	High	High	High
Not Found	Not Found	Not Found	High	High	High	High	Not Found	Not Found	Not Found
High	High	High	Not Found	Not Found	Not Found	Not Found	High	High	High
High	High	High	High	High	High	High	High	High	High
High	High	High	High	High	High	High	High	High	High
Not Found	Not Found	Not Found	High	High	High	High	High	High	High
High	High	High	High	High	High	High	High	High	High
High	High	High	High	High	High	High	Not Found	Not Found	Not Found
High	High	High	High	High	High	High	High	High	High
High	High	High	High	High	High	High	High	High	High
High	High	High	High	High	High	High	High	High	High
Not Found	Not Found	Not Found	High	High	High	High	High	High	High
High	High	High	High	High	High	High	High	High	High
High	High	High	High	High	High	High	High	High	High
High	High	High	High	High	High	High	High	High	High
High	High	High	Not Found	Not Found	Not Found	Not Found	High	High	High
High	High	High	Not Found	Not Found	Not Found	Not Found	High	High	High
Not Found	Not Found	Not Found	High	High	High	High	High	High	High

High	High	High	High	High	High	High	Not Found	Not Found	Not Found
High	High	High	High	High	High	High	High	High	High
High	High	High	Not Found	Not Found	Not Found	Not Found	High	High	High
High	High	High	Not Found	Not Found	Not Found	Not Found	High	High	High
High	High	High	High	High	High	High	High	High	High
High	High	High	High	High	High	High	High	High	High
High	High	High	High	High	High	High	High	High	High
High	High	High	High	High	High	High	Not Found	Not Found	Not Found
High	High	High	High	High	High	High	High	High	High
High	High	High	Not Found	Not Found	Not Found	Not Found	High	High	High
High	High	High	High	High	High	High	High	High	High
High	High	High	High	High	High	High	Not Found	Not Found	Not Found
High	High	High	High	High	High	High	High	High	High
Not Found	Not Found	Not Found	High	High	High	High	High	High	High
Not Found	Not Found	Not Found	High	High	High	High	Not Found	Not Found	Not Found
Not Found	Not Found	Not Found	High	High	High	High	High	High	High
High	High	High	High	High	High	High	Not Found	Not Found	Not Found
High	High	High	High	High	High	High	High	High	High
High	High	High	Not Found	Not Found	Not Found	Not Found	High	High	High
High	High	High	High	High	High	High	High	High	High
High	High	High	High	High	High	High	Not Found	Not Found	Not Found
Not Found	Not Found	Not Found	High	High	High	High	High	High	High
High	High	High	High	High	High	High	High	High	High
High	High	High	High	High	High	High	Not Found	Not Found	Not Found
High	High	High	High	High	High	High	High	High	High
Not Found	Not Found	Not Found	High	High	High	High	High	High	High
High	High	High	High	High	High	High	High	High	High
Not Found	Not Found	Not Found	Not Found	Not Found	Not Found	Not Found	High	High	High
High	High	High	High	High	High	High	Not Found	Not Found	Not Found
Not Found	Not Found	Not Found	Not Found	Not Found	Not Found	Not Found	High	High	High
High	High	High	High	High	High	High	Not Found	Not Found	Not Found
High	High	High	High	High	High	High	High	High	High
Not Found	Not Found	Not Found	High	High	High	High	High	High	High
High	High	High	High	High	High	High	High	High	High
High	High	High	High	High	High	High	High	High	High
High	High	High	Not Found	Not Found	Not Found	Not Found	High	High	High
High	High	High	Not Found	Not Found	Not Found	Not Found	High	High	High
High	High	High	High	High	High	High	High	High	High
High	High	High	High	High	High	High	Not Found	Not Found	Not Found
High	High	High	High	High	High	High	High	High	High
High	High	High	High	High	High	High	High	High	High
High	High	High	High	High	High	High	High	High	High
High	High	High	High	High	High	High	Not Found	Not Found	Not Found
High	High	High	High	High	High	High	Not Found	Not Found	Not Found
High	High	High	High	High	High	High	High	High	High
High	High	High	High	High	High	High	High	High	High
High	High	High	High	High	High	High	High	High	High
High	High	High	High	High	High	High	High	High	High
Not Found	Not Found	Not Found	High	High	High	High	High	High	High
Not Found	Not Found	Not Found	High	High	High	High	High	High	High

High	High	High	High	High	High	High	Not Found	Not Found	Not Found
High	High	High	High	High	High	High	High	High	High
High	High	High	High	High	High	High	High	High	High
High	High	High	High	High	High	High	High	High	High
Not Found	Not Found	Not Found	High	High	High	High	High	High	High
High	High	High	Not Found	Not Found	Not Found	Not Found	High	High	High
High	High	High	High	High	High	High	High	High	High
High	High	High	High	High	High	High	Not Found	Not Found	Not Found
High	High	High	High	High	High	High	High	High	High
Not Found	Not Found	Not Found	High	High	High	High	Not Found	Not Found	Not Found
High	High	High	High	High	High	High	High	High	High
High	High	High	High	High	High	High	High	High	High
High	High	High	High	High	High	High	High	High	High
High	High	High	High	High	High	High	High	High	High
High	High	High	High	High	High	High	High	High	High
Not Found	Not Found	Not Found	High	High	High	High	High	High	High
High	High	High	High	High	High	High	High	High	High
High	High	High	Not Found	Not Found	Not Found	Not Found	Not Found	Not Found	Not Found
High	High	High	High	High	High	High	High	High	High
Not Found	Not Found	Not Found	High	High	High	High	High	High	High
High	High	High	High	High	High	High	Not Found	Not Found	Not Found
High	High	High	High	High	High	High	High	High	High
High	High	High	High	High	High	High	High	High	High
High	High	High	High	High	High	High	Not Found	Not Found	Not Found
High	High	High	Not Found	Not Found	Not Found	Not Found	High	High	High
High	High	High	High	High	High	High	High	High	High
High	High	High	Not Found	Not Found	Not Found	Not Found	High	High	High
High	High	High	High	High	High	High	High	High	High
High	High	High	High	High	High	High	High	High	High
Not Found	Not Found	Not Found	High	High	High	High	High	High	High
High	High	High	Not Found	Not Found	Not Found	Not Found	High	High	High
High	High	High	Not Found	Not Found	Not Found	Not Found	High	High	High
High	High	High	High	High	High	High	High	High	High
High	High	High	High	High	High	High	Not Found	Not Found	Not Found
High	High	High	High	High	High	High	High	High	High
High	High	High	High	High	High	High	High	High	High
High	High	High	High	High	High	High	High	High	High
High	High	High	High	High	High	High	High	High	High
Not Found	Not Found	Not Found	High	High	High	High	High	High	High
Not Found	Not Found	Not Found	High	High	High	High	High	High	High
Not Found	Not Found	Not Found	High	High	High	High	High	High	High
High	High	High	High	High	High	High	High	High	High
High	High	High	High	High	High	High	High	High	High
High	High	High	High	High	High	High	High	High	High
High	High	High	High	High	High	High	High	High	High
Not Found	Not Found	Not Found	High	High	High	High	High	High	High
High	High	High	Not Found	Not Found	Not Found	Not Found	Not Found	Not Found	Not Found
High	High	High	Not Found	Not Found	Not Found	Not Found	High	High	High
High	High	High	High	High	High	High	Not Found	Not Found	Not Found
High	High	High	High	High	High	High	Not Found	Not Found	Not Found

Not Found	Not Found	Not Found	High	High	High	High	High	High	High
High	High	High	Not Found	Not Found	Not Found	Not Found	High	High	High
High	High	High	Not Found	Not Found	Not Found	Not Found	High	High	High
High	High	High	Not Found	Not Found	Not Found	Not Found	High	High	High
High	High	High	High	High	High	High	High	High	High
High	High	High	Not Found	Not Found	Not Found	Not Found	High	High	High
High	High	High	High	High	High	High	High	High	High
Not Found	Not Found	Not Found	High	High	High	High	High	High	High
High	High	High	Not Found	Not Found	Not Found	Not Found	High	High	High
High	High	High	High	High	High	High	High	High	High
Not Found	Not Found	Not Found	High	High	High	High	High	High	High
High	High	High	High	High	High	High	High	High	High
High	High	High	High	High	High	High	Not Found	Not Found	Not Found
Not Found	Not Found	Not Found	High	High	High	High	High	High	High
Not Found	Not Found	Not Found	High	High	High	High	High	High	High
High	High	High	Not Found	Not Found	Not Found	Not Found	High	High	High
High	High	High	High	High	High	High	High	High	High
High	High	High	Not Found	Not Found	Not Found	Not Found	High	High	High
High	High	High	High	High	High	High	High	High	High
High	High	High	High	High	High	High	Not Found	Not Found	Not Found
High	High	High	Not Found	Not Found	Not Found	Not Found	High	High	High
High	High	High	High	High	High	High	High	High	High
High	High	High	High	High	Not Found	High	Not Found	Not Found	Not Found
High	High	High	High	High	High	High	High	High	High
High	High	High	High	High	High	High	Not Found	Not Found	Not Found
High	High	High	Not Found	Not Found	Not Found	Not Found	High	High	High
Not Found	Not Found	Not Found	High	High	High	High	Not Found	Not Found	Not Found
High	High	High	Not Found	Not Found	Not Found	Not Found	High	High	High
High	High	High	High	High	High	High	Not Found	Not Found	Not Found
Not Found	Not Found	Not Found	High	High	High	High	High	High	High
High	High	High	High	High	High	High	High	High	High
Not Found	Not Found	Not Found	High	High	High	High	High	High	High
Not Found	Not Found	Not Found	Not Found	Not Found	Not Found	Not Found	High	High	High
Not Found	Not Found	Not Found	High	High	High	High	High	High	High
High	High	High	High	High	High	High	High	High	High
High	High	High	Not Found	Not Found	Not Found	Not Found	High	High	High
Not Found	Not Found	Not Found	High	High	High	High	High	High	High
High	High	High	Not Found	Not Found	Not Found	Not Found	High	High	High
Not Found	Not Found	Not Found	High	High	High	High	High	High	High
High	High	High	Not Found	Not Found	Not Found	Not Found	High	High	High
High	High	High	High	High	High	High	High	High	High
Not Found	Not Found	Not Found	High	High	High	High	High	High	High
High	High	High	High	High	High	High	High	High	High
Not Found	Not Found	Not Found	High	High	High	High	High	High	High
High	High	High	Not Found	Not Found	Not Found	Not Found	Not Found	Not Found	Not Found
Not Found	Not Found	Not Found	High	High	High	High	High	High	High
Not Found	Not Found	Not Found	High	High	High	High	Not Found	Not Found	Not Found
High	High	High	Not Found	Not Found	Not Found	Not Found	High	High	High
High	High	High	Not Found	Not Found	Not Found	Not Found	High	High	High
High	High	High	High	High	High	High	Not Found	Not Found	Not Found

Not Found	Not Found	Not Found	High	High	High	High	High	High	High
High	High	High	Not Found	Not Found	Not Found	Not Found	High	High	High
High	High	High	High	High	High	High	High	High	High
High	High	High	High	High	High	High	High	High	High
High	High	High	Not Found	Not Found	Not Found	Not Found	High	High	High
High	High	High	High	High	High	High	Not Found	Not Found	Not Found
High	High	High	Not Found	Not Found	Not Found	Not Found	High	High	High
Not Found	Not Found	Not Found	High	High	High	High	High	High	High
Not Found	Not Found	Not Found	High	High	High	High	High	High	High
High	High	High	High	High	High	High	High	High	High
High	High	High	High	High	High	High	High	High	High
Not Found	Not Found	Not Found	High	High	High	High	High	High	High
High	High	High	Not Found	Not Found	Not Found	Not Found	High	High	High
High	High	High	Not Found	Not Found	Not Found	Not Found	High	High	High
High	High	High	Not Found	Not Found	Not Found	Not Found	High	High	High
High	High	High	High	High	High	High	Not Found	Not Found	Not Found
High	High	High	Not Found	Not Found	Not Found	Not Found	High	High	High
Not Found	Not Found	Not Found	High	High	High	High	Not Found	Not Found	Not Found
High	High	High	High	High	High	High	High	High	High
High	High	High	High	High	High	High	High	High	High
High	High	High	Not Found	Not Found	Not Found	Not Found	High	High	High
High	High	High	High	High	High	High	High	High	High
High	High	High	High	High	High	High	High	High	High
Not Found	Not Found	Not Found	High	High	High	High	High	High	High
High	High	High	High	High	High	High	Not Found	Not Found	Not Found
High	High	High	High	High	High	High	Not Found	Not Found	Not Found
High	High	High	High	High	High	High	High	High	High
High	High	High	Not Found	Not Found	Not Found	Not Found	High	High	High
High	High	High	Not Found	Not Found	Not Found	Not Found	Not Found	Not Found	Not Found
Not Found	Not Found	Not Found	High	High	High	High	High	High	High
High	High	High	Not Found	Not Found	Not Found	Not Found	High	High	High
High	High	High	High	High	High	High	High	High	High
High	High	High	High	High	High	High	Not Found	Not Found	Not Found
High	High	High	Not Found	Not Found	Not Found	Not Found	Not Found	Not Found	Not Found
Not Found	Not Found	Not Found	High	High	High	High	High	High	High
High	High	High	High	High	High	High	High	High	High
High	High	High	High	High	High	High	High	High	High
Not Found	Not Found	Not Found	High	High	High	High	High	High	High
Not Found	Not Found	Not Found	High	High	High	High	High	High	High
Not Found	Not Found	Not Found	High	High	High	High	High	High	High
High	High	High	High	High	High	High	High	High	High
Not Found	Not Found	Not Found	High	High	High	High	High	High	High
High	High	High	High	High	High	High	Not Found	Not Found	Not Found
High	High	High	High	High	High	High	High	High	High
High	High	High	Not Found	Not Found	Not Found	Not Found	Not Found	Not Found	Not Found
High	High	High	Not Found	Not Found	Not Found	Not Found	High	High	High
High	High	High	Not Found	Not Found	Not Found	Not Found	High	High	High
Not Found	Not Found	Not Found	High	High	High	High	High	High	High
High	High	High	Not Found	Not Found	Not Found	Not Found	High	High	High
High	High	High	High	High	High	High	Not Found	Not Found	Not Found

Not Found	Not Found	Not Found	High	High	High	High	High	High	High
Not Found	Not Found	Not Found	High	High	High	High	Not Found	Not Found	Not Found
Not Found	Not Found	Not Found	High	High	High	High	Not Found	Not Found	Not Found
High	High	High	High	High	High	High	High	High	High
Not Found	Not Found	Not Found	High	High	High	High	High	High	High
High	High	High	Not Found	Not Found	Not Found	Not Found	High	High	High
High	High	High	High	High	High	High	High	High	High
High	High	High	High	High	High	High	High	High	High
Not Found	Not Found	Not Found	Not Found	Not Found	Not Found	Not Found	High	High	High
High	High	High	Not Found	Not Found	Not Found	Not Found	High	High	High
High	High	High	High	High	High	High	Not Found	Not Found	Not Found
High	High	High	Not Found	Not Found	Not Found	Not Found	Not Found	Not Found	Not Found
Not Found	Not Found	Not Found	Not Found	Not Found	Not Found	Not Found	High	High	High
Not Found	Not Found	Not Found	High	High	High	High	Not Found	Not Found	Not Found
Not Found	Not Found	Not Found	Not Found	Not Found	Not Found	Not Found	Not Found	Not Found	Not Found
Not Found	Not Found	Not Found	High	High	High	High	High	High	High
High	High	High	High	High	High	High	High	High	High
Not Found	Not Found	Not Found	High	High	High	High	High	High	High
Not Found	Not Found	Not Found	High	High	High	High	Not Found	Not Found	Not Found
High	High	High	Not Found	Not Found	Not Found	Not Found	High	High	High
Not Found	Not Found	Not Found	High	High	High	High	Not Found	Not Found	Not Found
High	High	High	High	High	High	High	High	High	High
High	High	High	Not Found	Not Found	Not Found	Not Found	Not Found	Not Found	Not Found
High	High	High	High	High	High	High	High	High	High
High	High	High	High	High	High	High	Not Found	Not Found	Not Found
Not Found	Not Found	Not Found	High	High	High	High	High	High	High
High	High	High	High	High	High	High	Not Found	Not Found	Not Found
High	High	High	Not Found	Not Found	Not Found	Not Found	High	High	High
Not Found	Not Found	Not Found	High	High	High	High	High	High	High
High	High	High	Not Found	Not Found	Not Found	Not Found	Not Found	Not Found	Not Found
Not Found	Not Found	Not Found	High	High	High	High	High	High	High
High	High	High	High	High	High	High	Not Found	Not Found	Not Found
High	High	High	High	High	High	High	Not Found	Not Found	Not Found
High	High	High	High	High	High	High	Not Found	Not Found	Not Found
High	High	High	High	High	High	High	Not Found	Not Found	Not Found
High	High	High	Not Found	Not Found	Not Found	Not Found	High	High	High
High	High	High	High	High	High	High	Not Found	Not Found	Not Found
High	High	High	Not Found	Not Found	Not Found	Not Found	High	High	High
High	High	High	High	High	High	High	Not Found	Not Found	Not Found
Not Found	Not Found	Not Found	High	High	High	High	Not Found	Not Found	Not Found
Not Found	Not Found	Not Found	High	High	High	High	Not Found	Not Found	Not Found
Not Found	Not Found	Not Found	High	High	High	High	Not Found	Not Found	Not Found
Not Found	Not Found	Not Found	High	High	High	High	High	High	High
Not Found	Not Found	Not Found	High	High	High	High	Not Found	Not Found	Not Found
Not Found	Not Found	Not Found	High	High	High	High	Not Found	Not Found	Not Found
High	High	High	High	High	High	High	Not Found	Not Found	Not Found
Not Found	Not Found	Not Found	High	High	High	High	High	High	High
High	High	High	High	High	High	High	High	High	High
Not Found	Not Found	Not Found	High	High	High	High	High	High	High
Not Found	Not Found	Not Found	High	High	High	High	High	High	High

Not Found	Not Found	Not Found	High	High	High	High	High	High	High
High	High	High	Not Found	Not Found	Not Found	Not Found	High	High	High
Not Found	Not Found	Not Found	High	High	High	High	Not Found	Not Found	Not Found
High	High	High	High	High	High	High	Not Found	Not Found	Not Found
Not Found	Not Found	Not Found	High	High	High	High	Not Found	Not Found	Not Found
Not Found	Not Found	Not Found	High	High	High	High	Not Found	Not Found	Not Found
Not Found	Not Found	Not Found	High	High	High	High	High	High	High
High	High	High	High	High	High	High	Not Found	Not Found	Not Found
High	High	High	High	High	High	High	Not Found	Not Found	Not Found
High	High	High	High	High	High	High	Not Found	Not Found	Not Found
High	High	High	High	High	High	High	Not Found	Not Found	Not Found
High	High	High	High	High	High	High	High	High	High
High	High	High	High	High	High	High	Not Found	Not Found	Not Found
Not Found	Not Found	Not Found	Not Found	Not Found	Not Found	Not Found	High	High	High
High	High	High	High	High	High	High	Not Found	Not Found	Not Found
Not Found	Not Found	Not Found	High	High	High	High	High	High	High
High	High	High	High	High	High	High	Not Found	Not Found	Not Found
High	High	High	High	High	High	High	High	High	High
Not Found	Not Found	Not Found	High	High	High	High	High	High	High
High	High	High	High	High	High	High	High	High	High
High	High	High	Not Found	Not Found	Not Found	Not Found	High	High	High
Not Found	Not Found	Not Found	High	High	High	High	Not Found	Not Found	Not Found
High	High	High	Not Found	Not Found	Not Found	Not Found	High	High	High
High	High	High	High	High	High	High	High	High	High
High	High	High	Not Found	Not Found	Not Found	Not Found	High	High	High
High	High	High	High	High	High	High	Not Found	Not Found	Not Found
High	High	High	High	High	High	High	High	High	High
High	High	High	Not Found	Not Found	Not Found	Not Found	High	High	High
High	High	High	High	High	High	High	Not Found	Not Found	Not Found
High	High	High	High	High	High	High	High	High	High
High	High	High	Not Found	Not Found	Not Found	Not Found	High	High	High
High	High	High	High	High	High	High	High	High	High
High	High	High	Not Found	Not Found	Not Found	Not Found	High	High	High
Not Found	Not Found	Not Found	High	High	High	High	Not Found	Not Found	Not Found
Not Found	Not Found	Not Found	High	High	High	High	Not Found	Not Found	Not Found
High	High	High	Not Found	Not Found	Not Found	Not Found	High	High	High
High	High	High	High	High	High	High	High	High	High
Not Found	Not Found	Not Found	High	High	High	High	High	High	High
High	High	High	Not Found	Not Found	Not Found	Not Found	High	High	High
High	High	High	Not Found	Not Found	Not Found	Not Found	High	High	High
High	High	High	Not Found	Not Found	Not Found	Not Found	Not Found	Not Found	Not Found
High	High	High	Not Found	Not Found	Not Found	Not Found	High	High	High
Not Found	Not Found	Not Found	High	High	High	High	High	High	High
High	High	High	High	High	High	High	Not Found	Not Found	Not Found
High	High	High	Not Found	Not Found	Not Found	Not Found	High	High	High
High	High	High	High	High	High	High	High	High	High
Not Found	Not Found	Not Found	High	High	High	High	High	High	High
High	High	High	Not Found	Not Found	Not Found	Not Found	High	High	High
High	High	High	Not Found	Not Found	Not Found	Not Found	High	High	High
High	High	High	High	High	High	High	Not Found	Not Found	Not Found

High	High	High	High	High	High	High	Not Found	Not Found	Not Found
Not Found	Not Found	Not Found	High	High	High	High	High	High	High
High	High	High	High	High	High	High	High	High	High
Not Found	Not Found	Not Found	Not Found	Not Found	Not Found	Not Found	High	High	High
Not Found	Not Found	Not Found	High	High	High	High	High	High	High
High	High	High	Not Found	Not Found	Not Found	Not Found	High	High	High
Not Found	Not Found	Not Found	High	High	High	High	High	High	High
Not Found	Not Found	Not Found	High	High	High	High	High	High	High
Not Found	Not Found	Not Found	High	High	High	High	Not Found	Not Found	Not Found
Not Found	Not Found	Not Found	High	High	High	High	High	High	High
Not Found	Not Found	Not Found	Not Found	Not Found	Not Found	Not Found	High	High	High
High	High	High	High	High	High	High	High	High	High
High	High	High	Not Found	Not Found	Not Found	Not Found	High	High	High
High	High	High	High	High	High	High	Not Found	Not Found	Not Found
High	High	High	High	High	High	High	High	High	High
Not Found	Not Found	Not Found	High	High	High	High	Not Found	Not Found	Not Found
High	High	High	High	High	High	High	Not Found	Not Found	Not Found
High	High	High	Not Found	Not Found	Not Found	Not Found	High	High	High
High	High	High	High	High	High	High	Not Found	Not Found	Not Found
Not Found	Not Found	Not Found	High	High	High	High	Not Found	Not Found	Not Found
High	High	High	Not Found	Not Found	Not Found	Not Found	Not Found	Not Found	Not Found
High	High	High	High	High	High	High	High	High	High
High	High	High	Not Found	Not Found	Not Found	Not Found	High	High	High
High	High	High	High	High	High	High	High	High	High
High	High	High	High	High	High	High	High	High	High
High	High	High	High	High	High	High	High	High	High
Not Found	Not Found	Not Found	High	High	High	High	High	High	High
Not Found	Not Found	Not Found	High	High	High	High	Not Found	Not Found	Not Found
High	High	High	High	High	High	High	Not Found	Not Found	Not Found
High	High	High	Not Found	Not Found	Not Found	Not Found	High	High	High
High	High	High	Not Found	Not Found	Not Found	Not Found	High	High	High
Not Found	Not Found	Not Found	High	High	High	High	High	High	High
Not Found	Not Found	Not Found	Not Found	Not Found	Not Found	Not Found	High	High	High
High	High	High	Not Found	Not Found	Not Found	Not Found	High	High	High
High	High	High	Not Found	Not Found	Not Found	Not Found	High	High	High
High	High	High	Not Found	Not Found	Not Found	Not Found	High	High	High
High	High	High	High	High	High	High	High	High	High
High	High	High	High	High	High	High	High	High	High
High	High	High	Not Found	Not Found	Not Found	Not Found	Not Found	Not Found	Not Found
High	High	High	High	High	High	High	Not Found	Not Found	Not Found
High	High	High	Not Found	Not Found	Not Found	Not Found	High	High	High
High	High	High	High	High	High	High	Not Found	Not Found	Not Found
High	High	High	Not Found	Not Found	Not Found	Not Found	High	High	High
High	High	High	High	High	High	High	Not Found	Not Found	Not Found
High	High	High	High	High	High	High	Not Found	Not Found	Not Found
High	High	High	Not Found	Not Found	Not Found	Not Found	High	High	High
Not Found	Not Found	Not Found	High	High	High	High	High	High	High
High	High	High	Not Found	Not Found	Not Found	Not Found	High	High	High
High	High	High	High	High	High	High	High	High	High
High	High	High	High	High	High	High	Not Found	Not Found	Not Found

Not Found	Not Found	Not Found	High	High	High	High	High	High	High
Not Found	Not Found	Not Found	High	High	High	High	High	High	High
High	High	High	High	High	High	High	Not Found	Not Found	Not Found
High	High	High	Not Found	Not Found	Not Found	Not Found	High	High	High
High	High	High	Not Found	Not Found	Not Found	Not Found	Not Found	Not Found	Not Found
High	High	High	High	High	High	High	Not Found	Not Found	Not Found
Not Found	Not Found	Not Found	Not Found	Not Found	Not Found	Not Found	Not Found	Not Found	Not Found
High	High	High	Not Found	Not Found	Not Found	Not Found	High	High	High
High	High	High	High	High	High	High	High	High	High
Not Found	Not Found	Not Found	High	High	High	High	High	High	High
High	High	High	High	High	High	High	Not Found	Not Found	Not Found
High	High	High	High	High	High	High	High	High	High
High	High	High	Not Found	Not Found	Not Found	Not Found	High	High	High
Not Found	Not Found	Not Found	High	High	High	High	High	High	High
High	High	High	Not Found	Not Found	Not Found	Not Found	High	High	High
High	High	High	Not Found	Not Found	Not Found	Not Found	High	High	High
High	High	High	Not Found	Not Found	Not Found	Not Found	Not Found	Not Found	Not Found
High	High	High	High	High	High	High	Not Found	Not Found	Not Found
High	High	High	Not Found	Not Found	Not Found	Not Found	High	High	High
High	High	High	High	High	High	High	Not Found	Not Found	Not Found
High	High	High	Not Found	Not Found	Not Found	Not Found	High	High	High
Not Found	Not Found	Not Found	High	High	High	High	High	High	High
High	High	High	High	High	High	High	High	High	High
Not Found	Not Found	Not Found	High	High	High	High	High	High	High
High	High	High	High	High	High	High	Not Found	Not Found	Not Found
High	High	High	Not Found	Not Found	Not Found	Not Found	High	High	High
High	High	High	Not Found	Not Found	Not Found	Not Found	High	High	High
Not Found	Not Found	Not Found	High	High	High	High	High	High	High
High	High	High	High	High	High	High	High	High	High
High	High	High	Not Found	Not Found	Not Found	Not Found	High	High	High
High	High	High	Not Found	Not Found	Not Found	Not Found	High	High	High
High	High	High	High	High	High	High	High	High	High
High	High	High	Not Found	Not Found	Not Found	Not Found	Not Found	Not Found	Not Found
High	High	High	High	High	High	High	High	High	High
High	High	High	Not Found	Not Found	Not Found	Not Found	High	High	High
Not Found	Not Found	Not Found	High	High	High	High	High	High	High
High	High	High	Not Found	Not Found	Not Found	Not Found	High	High	High
Not Found	Not Found	Not Found	High	High	High	High	High	High	High
High	High	High	Not Found	Not Found	Not Found	Not Found	High	High	High
High	High	High	High	High	High	High	Not Found	Not Found	Not Found
High	High	High	High	High	High	High	High	High	High
Not Found	Not Found	Not Found	Not Found	Not Found	Not Found	Not Found	Not Found	Not Found	Not Found
Not Found	Not Found	Not Found	High	High	High	High	Not Found	Not Found	Not Found
High	High	High	High	High	High	High	High	High	High
High	High	High	High	High	High	High	High	High	High
High	High	High	Not Found	Not Found	Not Found	Not Found	High	High	High
High	High	High	Not Found	Not Found	Not Found	Not Found	High	High	High
Not Found	Not Found	Not Found	High	High	High	High	High	High	High
High	High	High	High	High	High	High	Not Found	Not Found	Not Found
High	High	High	High	High	High	High	High	High	High

High	High	High	Not Found	Not Found	Not Found	Not Found	Not Found	Not Found	Not Found
High	High	High	High	High	High	High	High	High	High
High	High	High	Not Found	Not Found	Not Found	Not Found	High	High	High
Not Found	Not Found	Not Found	High	High	High	High	High	High	High
High	High	High	High	High	High	High	Not Found	Not Found	Not Found
High	High	High	Not Found	Not Found	Not Found	Not Found	High	High	High
Not Found	Not Found	Not Found	High	High	High	High	High	High	High
High	High	High	High	High	High	High	Not Found	Not Found	Not Found
Not Found	Not Found	Not Found	High	High	High	High	High	High	High
Not Found	Not Found	Not Found	High	High	High	High	High	High	High
High	High	High	Not Found	Not Found	Not Found	Not Found	High	High	High
High	High	High	Not Found	Not Found	Not Found	Not Found	High	High	High
High	High	High	Not Found	Not Found	Not Found	Not Found	High	High	High
High	High	High	High	High	High	High	Not Found	Not Found	Not Found
Not Found	Not Found	Not Found	High	High	High	High	High	High	High
Not Found	Not Found	Not Found	High	High	High	High	High	High	High
Not Found	Not Found	Not Found	High	High	High	High	High	High	High
Not Found	Not Found	Not Found	High	High	High	High	Not Found	Not Found	Not Found
Not Found	Not Found	Not Found	High	High	High	High	High	High	High
High	High	High	High	High	High	High	Not Found	Not Found	Not Found
High	High	High	High	High	High	High	High	High	High
Not Found	Not Found	Not Found	Not Found	Not Found	Not Found	Not Found	High	High	High
High	High	High	Not Found	Not Found	Not Found	Not Found	High	High	High
High	High	High	High	High	High	High	Not Found	Not Found	Not Found
High	High	High	Not Found	Not Found	Not Found	Not Found	High	High	High
High	High	High	High	High	High	High	Not Found	Not Found	Not Found
High	High	High	Not Found	Not Found	Not Found	Not Found	High	High	High
Not Found	Not Found	Not Found	High	High	High	High	Not Found	Not Found	Not Found
High	High	High	Not Found	Not Found	Not Found	Not Found	Not Found	Not Found	Not Found
Not Found	Not Found	Not Found	Not Found	Not Found	Not Found	Not Found	High	High	High
Not Found	Not Found	Not Found	High	High	High	High	High	High	High
Not Found	Not Found	Not Found	High	High	High	High	Not Found	Not Found	Not Found
High	High	High	High	High	High	High	High	High	High
High	High	High	High	High	High	High	High	High	High
Not Found	Not Found	Not Found	High	High	High	High	High	High	High
High	High	High	High	High	High	High	Not Found	Not Found	Not Found
High	High	High	High	High	High	High	Not Found	Not Found	Not Found
Not Found	Not Found	Not Found	Not Found	Not Found	Not Found	Not Found	High	High	High
High	High	High	Not Found	Not Found	Not Found	Not Found	High	High	High
Not Found	Not Found	Not Found	Not Found	Not Found	Not Found	Not Found	High	High	High
Not Found	Not Found	Not Found	High	High	High	High	Not Found	Not Found	Not Found
High	High	High	High	High	High	High	Not Found	Not Found	Not Found
High	High	High	Not Found	Not Found	Not Found	Not Found	High	High	High
Not Found	Not Found	Not Found	High	High	High	High	Not Found	Not Found	Not Found
Not Found	Not Found	Not Found	Not Found	Not Found	Not Found	Not Found	High	High	High
Not Found	Not Found	Not Found	High	High	High	High	High	High	High
Not Found	Not Found	Not Found	High	High	High	High	High	High	High
Not Found	Not Found	Not Found	Not Found	Not Found	Not Found	Not Found	High	High	High
High	High	High	High	High	High	High	Not Found	Not Found	Not Found
Not Found	Not Found	Not Found	High	High	High	High	High	High	High

High	High	High	Not Found	Not Found	Not Found	Not Found	High	High	High
High	High	High	High	High	High	High	Not Found	Not Found	Not Found
High	High	High	High	High	High	High	Not Found	Not Found	Not Found
High	High	High	High	High	High	High	High	High	High
Not Found	Not Found	Not Found	High	High	High	High	High	High	High
High	High	High	High	High	High	High	Not Found	Not Found	Not Found
High	High	High	Not Found	Not Found	Not Found	Not Found	High	High	High
High	High	High	Not Found	Not Found	Not Found	Not Found	High	High	High
Not Found	Not Found	Not Found	High	High	High	High	High	High	High
Not Found	Not Found	Not Found	High	High	High	High	High	High	High
Not Found	Not Found	Not Found	Not Found	Not Found	Not Found	Not Found	Not Found	Not Found	Not Found
High	High	High	High	High	High	High	High	High	High
High	High	High	High	High	High	High	Not Found	Not Found	Not Found
Not Found	Not Found	Not Found	High	High	High	High	High	High	High
Not Found	Not Found	Not Found	High	High	High	High	High	High	High
High	High	High	High	High	High	High	High	High	High
Not Found	Not Found	Not Found	High	High	High	High	High	High	High
High	High	High	High	High	High	High	Not Found	Not Found	Not Found
High	High	High	Not Found	Not Found	Not Found	Not Found	High	High	High
Not Found	Not Found	Not Found	High	High	High	High	High	High	High
High	High	High	High	High	High	High	High	High	High
Not Found	Not Found	Not Found	Not Found	Not Found	Not Found	Not Found	High	High	High
High	High	High	Not Found	Not Found	Not Found	Not Found	Not Found	Not Found	Not Found
Not Found	Not Found	Not Found	High	High	High	High	High	High	High
High	High	High	High	High	High	High	Not Found	Not Found	Not Found
Not Found	Not Found	Not Found	High	High	High	High	Not Found	Not Found	Not Found
Not Found	Not Found	Not Found	Not Found	Not Found	Not Found	Not Found	High	High	High
High	High	High	High	High	High	High	High	High	High
Not Found	Not Found	Not Found	High	High	High	High	High	High	High
High	High	High	High	High	High	High	Not Found	Not Found	Not Found
High	High	High	High	High	High	High	High	High	High
Not Found	Not Found	Not Found	Not Found	Not Found	Not Found	Not Found	High	High	High
Not Found	Not Found	Not Found	High	High	High	High	High	High	High
High	High	High	High	High	High	High	High	High	High
High	High	High	Not Found	Not Found	Not Found	Not Found	High	High	High
Not Found	Not Found	Not Found	High	High	High	High	Not Found	Not Found	Not Found
High	High	High	High	High	High	High	High	High	High
High	High	High	High	High	High	High	Not Found	Not Found	Not Found
High	High	High	High	High	High	High	Not Found	Not Found	Not Found
High	High	High	Not Found	Not Found	Not Found	Not Found	Not Found	Not Found	Not Found
High	High	High	Not Found	Not Found	Not Found	Not Found	High	High	High
Not Found	Not Found	Not Found	High	High	High	High	Not Found	Not Found	Not Found
Not Found	Not Found	Not Found	High	High	High	High	Not Found	Not Found	Not Found
High	High	High	Not Found	Not Found	Not Found	Not Found	Not Found	Not Found	Not Found
Not Found	Not Found	Not Found	High	High	High	High	High	High	High
High	High	High	Not Found	Not Found	Not Found	Not Found	Not Found	Not Found	Not Found
Not Found	Not Found	Not Found	High	High	High	High	High	High	High
High	High	High	High	High	High	High	Not Found	Not Found	Not Found
High	High	High	High	High	High	High	Not Found	Not Found	Not Found
High	High	High	High	High	High	High	Not Found	Not Found	Not Found

High	High	High	High	High	High	High	Not Found	Not Found	Not Found
High	High	High	High	High	High	High	High	High	High
Not Found	Not Found	Not Found	High	High	High	High	High	High	High
Not Found	Not Found	Not Found	Not Found	Not Found	Not Found	Not Found	Not Found	Not Found	Not Found
High	High	High	Not Found	Not Found	Not Found	Not Found	Not Found	Not Found	Not Found
High	High	High	Not Found	Not Found	Not Found	Not Found	Not Found	Not Found	Not Found
Not Found	Not Found	Not Found	High	High	High	High	Not Found	Not Found	Not Found
Not Found	Not Found	Not Found	High	High	High	High	High	High	High
Not Found	Not Found	Not Found	High	High	High	High	High	High	High
High	High	High	High	High	High	High	High	High	High
High	High	High	High	High	High	High	Not Found	Not Found	Not Found
Not Found	Not Found	Not Found	High	High	High	High	High	High	High
High	High	High	High	High	High	High	Not Found	Not Found	Not Found
Not Found	Not Found	Not Found	High	High	High	High	Not Found	Not Found	Not Found
High	High	High	Not Found	Not Found	Not Found	Not Found	High	High	High
Not Found	Not Found	Not Found	High	High	High	High	High	High	High
High	High	High	High	High	High	High	High	High	High
Not Found	Not Found	Not Found	High	High	High	High	High	High	High
Not Found	Not Found	Not Found	Not Found	Not Found	Not Found	Not Found	High	High	High
Not Found	Not Found	Not Found	Not Found	Not Found	Not Found	Not Found	Not Found	Not Found	Not Found
High	High	High	High	High	High	High	High	High	High
Not Found	Not Found	Not Found	High	High	High	High	High	High	High
High	High	High	Not Found	Not Found	Not Found	Not Found	Not Found	Not Found	Not Found
High	High	High	High	High	High	High	High	High	High
High	High	High	High	High	High	High	Not Found	Not Found	Not Found
Not Found	Not Found	Not Found	High	High	High	High	High	High	High
High	High	High	Not Found	Not Found	Not Found	Not Found	High	High	High
High	High	High	High	High	High	High	High	High	High
High	High	High	Not Found	Not Found	Not Found	Not Found	High	High	High
High	High	High	High	High	High	High	High	High	High
High	High	High	High	High	High	High	Not Found	Not Found	Not Found
Not Found	Not Found	Not Found	High	High	High	High	High	High	High
High	High	High	High	High	High	High	Not Found	Not Found	Not Found
High	High	High	High	High	High	High	High	High	High
High	High	High	High	High	High	High	High	High	High
Not Found	Not Found	Not Found	Not Found	Not Found	Not Found	Not Found	Not Found	Not Found	Not Found
High	High	High	High	High	High	High	Not Found	Not Found	Not Found
Not Found	Not Found	Not Found	High	High	High	High	High	High	High
High	High	High	High	High	High	High	High	High	High
High	High	High	High	High	High	High	High	High	High
Not Found	Not Found	Not Found	Not Found	Not Found	Not Found	Not Found	High	High	High
High	High	High	High	High	High	High	Not Found	Not Found	Not Found
High	High	High	High	High	High	High	Not Found	Not Found	Not Found
High	High	High	Not Found	Not Found	Not Found	Not Found	High	High	High
High	High	High	Not Found	Not Found	Not Found	Not Found	High	High	High
High	High	High	Not Found	Not Found	Not Found	Not Found	High	High	High
High	High	High	Not Found	Not Found	Not Found	Not Found	High	High	High
Not Found	Not Found	Not Found	High	High	High	High	High	High	High
High	High	High	High	High	High	High	Not Found	Not Found	Not Found
High	High	High	Not Found	Not Found	Not Found	Not Found	Not Found	Not Found	Not Found

Not Found	Not Found	Not Found	High	High	High	High	Not Found	Not Found	Not Found
Not Found	Not Found	Not Found	High	High	High	High	High	High	High
High	High	High	High	High	High	High	Not Found	Not Found	Not Found
High	High	High	Not Found	Not Found	Not Found	Not Found	High	High	High
High	High	High	Not Found	Not Found	Not Found	Not Found	High	High	High
Not Found	Not Found	Not Found	High	High	High	High	High	High	High
Not Found	Not Found	Not Found	Not Found	Not Found	Not Found	Not Found	High	High	High
High	High	High	High	High	High	High	Not Found	Not Found	Not Found
High	High	High	Not Found	Not Found	Not Found	Not Found	High	High	High
Not Found	Not Found	Not Found	Not Found	Not Found	Not Found	Not Found	High	High	High
Not Found	Not Found	Not Found	Not Found	Not Found	Not Found	Not Found	High	High	High
Not Found	Not Found	Not Found	High	High	High	High	High	High	High
High	High	High	Not Found	Not Found	Not Found	Not Found	High	High	High
High	High	High	Not Found	Not Found	Not Found	Not Found	Not Found	Not Found	Not Found
Not Found	Not Found	Not Found	High	High	High	High	High	High	High
High	High	High	Not Found	Not Found	Not Found	Not Found	Not Found	Not Found	Not Found
High	High	High	Not Found	Not Found	Not Found	Not Found	High	High	High
High	High	High	Not Found	Not Found	Not Found	Not Found	High	High	High
Not Found	Not Found	Not Found	High	High	High	High	High	High	High
Not Found	Not Found	Not Found	High	High	High	High	Not Found	Not Found	Not Found
High	High	High	High	High	High	High	Not Found	Not Found	Not Found
Not Found	Not Found	Not Found	High	High	High	High	High	High	High
Not Found	Not Found	Not Found	High	High	High	High	High	High	High
Not Found	Not Found	Not Found	High	High	High	High	High	High	High
High	High	High	Not Found	Not Found	Not Found	Not Found	High	High	High
Not Found	Not Found	Not Found	High	High	High	High	High	High	High
Not Found	Not Found	Not Found	High	High	High	High	High	High	High
High	High	High	Not Found	Not Found	Not Found	Not Found	Not Found	Not Found	Not Found
High	High	High	Not Found	Not Found	Not Found	Not Found	Not Found	Not Found	Not Found
High	High	High	High	High	High	High	Not Found	Not Found	Not Found
High	High	High	Not Found	Not Found	Not Found	Not Found	High	High	High
High	High	High	Not Found	Not Found	Not Found	Not Found	Not Found	Not Found	Not Found
High	High	High	Not Found	Not Found	Not Found	Not Found	Not Found	Not Found	Not Found
Not Found	Not Found	Not Found	High	High	High	High	Not Found	Not Found	Not Found
Not Found	Not Found	Not Found	High	High	High	High	High	High	High
High	High	High	High	High	High	High	Not Found	Not Found	Not Found
Not Found	Not Found	Not Found	High	High	High	High	Not Found	Not Found	Not Found
High	High	High	Not Found	Not Found	Not Found	Not Found	Not Found	Not Found	Not Found
Not Found	Not Found	Not Found	Not Found	Not Found	Not Found	Not Found	High	High	High
High	High	High	Not Found	Not Found	Not Found	Not Found	Not Found	Not Found	Not Found
High	High	High	High	High	High	High	Not Found	Not Found	Not Found
Not Found	Not Found	Not Found	Not Found	Not Found	Not Found	Not Found	High	High	High
Not Found	Not Found	Not Found	Not Found	Not Found	Not Found	Not Found	High	High	High
High	High	High	Not Found	Not Found	Not Found	Not Found	High	High	High
High	High	High	High	High	High	High	Not Found	Not Found	Not Found
Not Found	Not Found	Not Found	High	High	High	High	Not Found	Not Found	Not Found
High	High	High	Not Found	Not Found	Not Found	Not Found	High	High	High
High	High	High	Not Found	Not Found	Not Found	Not Found	Not Found	Not Found	Not Found
Not Found	Not Found	Not Found	Not Found	Not Found	Not Found	Not Found	Not Found	Not Found	Not Found
High	High	High	Not Found	Not Found	Not Found	Not Found	Not Found	Not Found	Not Found

High	High	High	High	High	High	High	Not Found	Not Found	Not Found
Not Found	Not Found	Not Found	High	High	High	High	High	High	High
Not Found	Not Found	Not Found	Not Found	Not Found	Not Found	Not Found	High	High	High
High	High	High	High	High	High	High	Not Found	Not Found	Not Found
Not Found	Not Found	Not Found	Not Found	Not Found	Not Found	Not Found	High	High	High
Not Found	Not Found	Not Found	Not Found	Not Found	Not Found	Not Found	Not Found	Not Found	Not Found
High	High	High	High	High	High	High	Not Found	Not Found	Not Found
Not Found	Not Found	Not Found	High	High	High	High	High	High	High
High	High	High	Not Found	Not Found	Not Found	Not Found	High	High	High
High	High	High	Not Found	Not Found	Not Found	Not Found	Not Found	Not Found	Not Found
Not Found	Not Found	Not Found	High	High	High	High	Not Found	Not Found	Not Found
High	High	High	High	High	High	High	Not Found	Not Found	Not Found
Not Found	Not Found	Not Found	Not Found	Not Found	Not Found	Not Found	High	High	High
Not Found	Not Found	Not Found	High	High	High	High	Not Found	Not Found	Not Found
High	High	High	High	High	High	High	Not Found	Not Found	Not Found
High	High	High	Not Found	Not Found	Not Found	Not Found	Not Found	Not Found	Not Found
Not Found	Not Found	Not Found	High	High	High	High	Not Found	Not Found	Not Found
Not Found	Not Found	Not Found	High	High	High	High	Not Found	Not Found	Not Found
Not Found	Not Found	Not Found	High	High	High	High	Not Found	Not Found	Not Found
High	High	High	Not Found	Not Found	Not Found	Not Found	Not Found	Not Found	Not Found
High	High	High	Not Found	Not Found	Not Found	Not Found	High	High	High
High	High	High	Not Found	Not Found	Not Found	Not Found	High	High	High
High	High	High	Not Found	Not Found	Not Found	Not Found	High	High	High
Not Found	Not Found	Not Found	High	High	High	High	Not Found	Not Found	Not Found
High	High	High	High	High	High	High	Not Found	Not Found	Not Found
High	High	High	Not Found	Not Found	Not Found	Not Found	Not Found	Not Found	Not Found
Not Found	Not Found	Not Found	High	High	High	High	High	High	High
Not Found	Not Found	Not Found	Not Found	Not Found	Not Found	Not Found	High	High	High
High	High	High	High	High	High	High	Not Found	Not Found	Not Found
High	High	High	Not Found	Not Found	Not Found	Not Found	Not Found	Not Found	Not Found
High	High	High	Not Found	Not Found	Not Found	Not Found	Not Found	Not Found	Not Found
High	High	High	Not Found	Not Found	Not Found	Not Found	Not Found	Not Found	Not Found
Not Found	Not Found	Not Found	High	High	High	High	Not Found	Not Found	Not Found
Not Found	Not Found	Not Found	High	High	High	High	Not Found	Not Found	Not Found
Not Found	Not Found	Not Found	High	High	High	High	Not Found	Not Found	Not Found
Not Found	Not Found	Not Found	Not Found	Not Found	Not Found	Not Found	High	High	High
Not Found	Not Found	Not Found	High	High	High	High	High	High	High
Not Found	Not Found	Not Found	High	High	High	High	High	High	High
Not Found	Not Found	Not Found	High	High	High	High	Not Found	Not Found	Not Found
Not Found	Not Found	Not Found	High	High	High	High	Not Found	Not Found	Not Found
Not Found	Not Found	Not Found	High	High	High	High	High	High	High
Not Found	Not Found	Not Found	High	High	High	High	High	High	High
High	High	High	High	High	High	High	Not Found	Not Found	Not Found
High	High	High	Not Found	Not Found	Not Found	Not Found	High	High	High
Not Found	Not Found	Not Found	Not Found	Not Found	Not Found	Not Found	Not Found	Not Found	Not Found
Not Found	Not Found	Not Found	High	High	High	High	High	High	High
Not Found	Not Found	Not Found	High	High	High	High	High	High	High
High	High	High	Not Found	Not Found	Not Found	Not Found	Not Found	Not Found	Not Found
High	High	High	Not Found	Not Found	Not Found	Not Found	High	High	High
Not Found	Not Found	Not Found	High	High	High	High	High	High	High

High	High	High	Not Found	Not Found	Not Found	Not Found	Not Found	Not Found	Not Found
High	High	High	Not Found	Not Found	Not Found	Not Found	Not Found	Not Found	Not Found
High	High	High	High	High	High	High	Not Found	Not Found	Not Found
Not Found	Not Found	Not Found	High	High	High	High	Not Found	Not Found	Not Found
High	High	High	High	High	High	High	Not Found	Not Found	Not Found
High	High	High	Not Found	Not Found	Not Found	Not Found	Not Found	Not Found	Not Found
High	High	High	Not Found	Not Found	Not Found	Not Found	Not Found	Not Found	Not Found
Not Found	Not Found	Not Found	Not Found	Not Found	Not Found	Not Found	High	High	High
High	High	High	Not Found	Not Found	Not Found	Not Found	Not Found	Not Found	Not Found
Not Found	Not Found	Not Found	Not Found	Not Found	Not Found	Not Found	High	High	High
High	High	High	Not Found	Not Found	Not Found	Not Found	High	High	High
High	High	High	Not Found	Not Found	Not Found	Not Found	Not Found	Not Found	Not Found
High	High	High	Not Found	Not Found	Not Found	Not Found	High	High	High
High	High	High	High	High	High	High	Not Found	Not Found	Not Found
Not Found	Not Found	Not Found	High	High	High	High	Not Found	Not Found	Not Found
Not Found	Not Found	Not Found	High	High	High	High	High	High	High
Not Found	Not Found	Not Found	Not Found	Not Found	Not Found	Not Found	Not Found	Not Found	Not Found
Not Found	Not Found	Not Found	Not Found	Not Found	Not Found	Not Found	High	High	High
Not Found	Not Found	Not Found	High	High	High	High	High	High	High
Not Found	Not Found	Not Found	Not Found	Not Found	Not Found	Not Found	High	High	High
Not Found	Not Found	Not Found	Not Found	Not Found	Not Found	Not Found	Not Found	Not Found	Not Found
Not Found	Not Found	Not Found	High	High	High	High	Not Found	Not Found	Not Found
High	High	High	Not Found	Not Found	Not Found	Not Found	Not Found	Not Found	Not Found
Not Found	Not Found	Not Found	High	High	High	High	High	High	High
High	High	High	Not Found	Not Found	Not Found	Not Found	High	High	High
High	High	High	Not Found	Not Found	Not Found	Not Found	Not Found	Not Found	Not Found
Not Found	Not Found	Not Found	High	High	High	High	Not Found	Not Found	Not Found
Not Found	Not Found	Not Found	Not Found	Not Found	Not Found	Not Found	High	High	High
High	High	High	Not Found	Not Found	Not Found	Not Found	High	High	High
High	High	High	High	High	High	High	Not Found	Not Found	Not Found
Not Found	Not Found	Not Found	High	High	High	High	High	High	High
High	High	High	Not Found	Not Found	Not Found	Not Found	Not Found	Not Found	Not Found
Not Found	Not Found	Not Found	High	High	High	High	Not Found	Not Found	Not Found
Not Found	Not Found	Not Found	Not Found	Not Found	Not Found	Not Found	High	High	High
High	High	High	High	High	High	High	Not Found	Not Found	Not Found
Not Found	Not Found	Not Found	High	High	High	High	High	High	High
High	High	High	High	High	High	High	Not Found	Not Found	Not Found
High	High	High	Not Found	Not Found	Not Found	Not Found	Not Found	Not Found	Not Found
Not Found	Not Found	Not Found	High	High	High	High	Not Found	Not Found	Not Found
Not Found	Not Found	Not Found	Not Found	Not Found	Not Found	Not Found	Not Found	Not Found	Not Found
High	High	High	Not Found	Not Found	Not Found	Not Found	High	High	High
High	High	High	Not Found	Not Found	Not Found	Not Found	Not Found	Not Found	Not Found
Not Found	Not Found	Not Found	High	High	High	High	Not Found	Not Found	Not Found
Not Found	Not Found	Not Found	Not Found	Not Found	Not Found	Not Found	High	High	High
Not Found	Not Found	Not Found	High	High	High	High	Not Found	Not Found	Not Found
Not Found	Not Found	Not Found	High	High	High	High	Not Found	Not Found	Not Found
High	High	High	High	High	High	High	Not Found	Not Found	Not Found
Not Found	Not Found	Not Found	High	High	High	High	Not Found	Not Found	Not Found
High	High	High	Not Found	Not Found	Not Found	Not Found	Not Found	Not Found	Not Found
Not Found	Not Found	Not Found	High	High	High	High	Not Found	Not Found	Not Found

Not Found	Not Found	Not Found	High	High	High	High	Not Found	Not Found	Not Found
Not Found	Not Found	Not Found	High	High	High	High	High	High	High
High	High	High	Not Found	Not Found	Not Found	Not Found	Not Found	Not Found	Not Found
High	High	High	Not Found	Not Found	Not Found	Not Found	Not Found	Not Found	Not Found
High	High	High	High	High	High	High	Not Found	Not Found	Not Found
High	High	High	Not Found	Not Found	Not Found	Not Found	High	High	High
Not Found	Not Found	Not Found	Not Found	Not Found	Not Found	Not Found	Not Found	Not Found	Not Found
Not Found	Not Found	Not Found	High	High	High	High	Not Found	Not Found	Not Found
High	High	High	Not Found	Not Found	Not Found	Not Found	Not Found	Not Found	Not Found
High	High	High	Not Found	Not Found	Not Found	Not Found	Not Found	Not Found	Not Found
High	High	High	High	High	High	High	Not Found	Not Found	Not Found
High	High	High	Not Found	Not Found	Not Found	Not Found	High	High	High
Not Found	Not Found	Not Found	High	High	High	High	Not Found	Not Found	Not Found
Not Found	Not Found	Not Found	Not Found	Not Found	Not Found	Not Found	High	High	High
Not Found	Not Found	Not Found	Not Found	Not Found	Not Found	Not Found	Not Found	Not Found	Not Found
Not Found	Not Found	Not Found	High	High	High	High	Not Found	Not Found	Not Found
High	High	High	Not Found	Not Found	Not Found	Not Found	Not Found	Not Found	Not Found
Not Found	Not Found	Not Found	Not Found	Not Found	Not Found	Not Found	High	High	High
Not Found	Not Found	Not Found	High	High	High	High	High	High	High
Not Found	Not Found	Not Found	High	High	High	High	High	High	High
Not Found	Not Found	Not Found	High	High	High	High	Not Found	Not Found	Not Found
Not Found	Not Found	Not Found	Not Found	Not Found	Not Found	Not Found	High	High	High
High	High	High	High	High	High	High	Not Found	Not Found	Not Found
Not Found	Not Found	Not Found	Not Found	Not Found	Not Found	Not Found	High	High	High
Not Found	Not Found	Not Found	Not Found	Not Found	Not Found	Not Found	High	High	High
Not Found	Not Found	Not Found	Not Found	Not Found	Not Found	Not Found	High	High	High
High	High	High	Not Found	Not Found	Not Found	Not Found	Not Found	Not Found	Not Found
Not Found	Not Found	Not Found	High	High	High	High	Not Found	Not Found	Not Found
High	High	High	High	High	High	High	Not Found	Not Found	Not Found
High	High	High	Not Found	Not Found	Not Found	Not Found	Not Found	Not Found	Not Found
Not Found	Not Found	Not Found	High	High	High	High	High	High	High
Not Found	Not Found	Not Found	Not Found	Not Found	Not Found	Not Found	High	High	High
High	High	High	Not Found	Not Found	Not Found	Not Found	High	High	High
Not Found	Not Found	Not Found	Not Found	Not Found	Not Found	Not Found	High	High	High
Not Found	Not Found	Not Found	High	High	High	High	High	High	High
Not Found	Not Found	Not Found	Not Found	Not Found	Not Found	Not Found	Not Found	Not Found	Not Found
Not Found	Not Found	Not Found	Not Found	Not Found	Not Found	Not Found	High	High	High
High	High	High	Not Found	Not Found	Not Found	Not Found	Not Found	Not Found	Not Found
High	High	High	Not Found	Not Found	Not Found	Not Found	High	High	High
Not Found	Not Found	Not Found	High	High	High	High	Not Found	Not Found	Not Found
High	High	High	Not Found	Not Found	Not Found	Not Found	Not Found	Not Found	Not Found
Not Found	Not Found	Not Found	High	High	High	High	Not Found	Not Found	Not Found
High	High	High	Not Found	Not Found	Not Found	Not Found	Not Found	Not Found	Not Found
Not Found	Not Found	Not Found	Not Found	Not Found	Not Found	Not Found	High	High	High
High	High	High	High	High	High	High	Not Found	Not Found	Not Found
Not Found	Not Found	Not Found	High	High	High	High	High	High	High
High	High	High	Not Found	Not Found	Not Found	Not Found	Not Found	Not Found	Not Found
High	High	High	High	High	High	High	Not Found	Not Found	Not Found
Not Found	Not Found	Not Found	Not Found	Not Found	Not Found	Not Found	High	High	High
High	High	High	Not Found	Not Found	Not Found	Not Found	Not Found	Not Found	Not Found

High	High	High	Not Found	Not Found	Not Found	Not Found	High	High	High
High	High	High	Not Found	Not Found	Not Found	Not Found	Not Found	Not Found	Not Found
High	High	High	Not Found	Not Found	Not Found	Not Found	Not Found	Not Found	Not Found
Not Found	Not Found	Not Found	High	High	High	High	Not Found	Not Found	Not Found
Not Found	Not Found	Not Found	High	High	High	High	Not Found	Not Found	Not Found
High	High	High	Not Found	Not Found	Not Found	Not Found	Not Found	Not Found	Not Found
Not Found	Not Found	Not Found	Not Found	Not Found	Not Found	Not Found	High	High	High
Not Found	Not Found	Not Found	High	High	High	High	Not Found	Not Found	Not Found
Not Found	Not Found	Not Found	High	High	High	High	Not Found	Not Found	Not Found
Not Found	Not Found	Not Found	High	High	High	High	High	High	High
Not Found	Not Found	Not Found	High	High	High	High	High	High	High
High	High	High	Not Found	Not Found	Not Found	Not Found	High	High	High
High	High	High	Not Found	Not Found	Not Found	Not Found	Not Found	Not Found	Not Found
Not Found	Not Found	Not Found	High	High	High	High	High	High	High
Not Found	Not Found	Not Found	High	High	High	High	Not Found	Not Found	Not Found
Not Found	Not Found	Not Found	High	High	High	High	High	High	High
Not Found	Not Found	Not Found	High	High	High	High	Not Found	Not Found	Not Found
Not Found	Not Found	Not Found	Not Found	Not Found	Not Found	Not Found	High	High	High
High	High	High	Not Found	Not Found	Not Found	Not Found	High	High	High
High	High	High	Not Found	Not Found	Not Found	Not Found	Not Found	Not Found	Not Found
High	High	High	High	High	High	High	Not Found	Not Found	Not Found
High	High	High	High	High	High	High	Not Found	Not Found	Not Found
High	High	High	Not Found	Not Found	Not Found	Not Found	Not Found	Not Found	Not Found
Not Found	Not Found	Not Found	Not Found	Not Found	Not Found	Not Found	High	High	High
Not Found	Not Found	Not Found	High	High	High	High	High	High	High
Not Found	Not Found	Not Found	Not Found	Not Found	Not Found	Not Found	High	High	High
High	High	High	High	High	High	High	Not Found	Not Found	Not Found
Not Found	Not Found	Not Found	High	High	High	High	Not Found	Not Found	Not Found
Not Found	Not Found	Not Found	High	High	High	High	High	High	High
High	High	High	Not Found	Not Found	Not Found	Not Found	High	High	High
High	High	High	Not Found	Not Found	Not Found	Not Found	Not Found	Not Found	Not Found
Not Found	Not Found	Not Found	Not Found	Not Found	Not Found	Not Found	High	High	High
High	High	High	Not Found	Not Found	Not Found	Not Found	High	High	High
High	High	High	Not Found	Not Found	Not Found	Not Found	Not Found	Not Found	Not Found
Not Found	Not Found	Not Found	Not Found	Not Found	Not Found	Not Found	High	High	High
Not Found	Not Found	Not Found	Not Found	Not Found	Not Found	Not Found	High	High	High
High	High	High	Not Found	Not Found	Not Found	Not Found	Not Found	Not Found	Not Found
Not Found	Not Found	Not Found	Not Found	Not Found	Not Found	Not Found	Not Found	Not Found	Not Found
Not Found	Not Found	Not Found	Not Found	Not Found	Not Found	Not Found	High	High	High
High	High	High	High	High	High	High	Not Found	Not Found	Not Found
High	High	High	Not Found	Not Found	Not Found	Not Found	Not Found	Not Found	Not Found
Not Found	Not Found	Not Found	Not Found	Not Found	Not Found	Not Found	High	High	High
Not Found	Not Found	Not Found	Not Found	Not Found	Not Found	Not Found	High	High	High
Not Found	Not Found	Not Found	High	High	High	High	Not Found	Not Found	Not Found
High	High	High	Not Found	Not Found	Not Found	Not Found	High	High	High
High	High	High	Not Found	Not Found	Not Found	Not Found	High	High	High
High	High	High	High	High	High	High	Not Found	Not Found	Not Found
Not Found	Not Found	Not Found	Not Found	Not Found	Not Found	Not Found	High	High	High
High	High	High	Not Found	Not Found	Not Found	Not Found	Not Found	Not Found	Not Found
High	High	High	High	High	High	High	Not Found	Not Found	Not Found

Not Found	Not Found	Not Found	High	High	High	High	High	High	High
High	High	High	Not Found	Not Found	Not Found	Not Found	Not Found	Not Found	Not Found
Not Found	Not Found	Not Found	High	High	High	High	Not Found	Not Found	Not Found
Not Found	Not Found	Not Found	High	High	High	High	Not Found	Not Found	Not Found
Not Found	Not Found	Not Found	Not Found	Not Found	Not Found	Not Found	High	High	High
High	High	High	Not Found	Not Found	Not Found	Not Found	High	High	High
High	High	High	Not Found	Not Found	Not Found	Not Found	Not Found	Not Found	Not Found
High	High	High	Not Found	Not Found	Not Found	Not Found	High	High	High
Not Found	Not Found	Not Found	High	High	High	High	Not Found	Not Found	Not Found
Not Found	Not Found	Not Found	High	High	High	High	High	High	High
High	High	High	Not Found	Not Found	Not Found	Not Found	Not Found	Not Found	Not Found
Not Found	Not Found	Not Found	High	High	High	High	Not Found	Not Found	Not Found
High	High	High	High	High	High	High	Not Found	Not Found	Not Found
Not Found	Not Found	Not Found	High	High	High	High	Not Found	Not Found	Not Found
Not Found	Not Found	Not Found	High	High	High	High	High	High	High
Not Found	Not Found	Not Found	High	High	High	High	High	High	High
Not Found	Not Found	Not Found	High	High	High	High	High	High	High
Not Found	Not Found	Not Found	Not Found	Not Found	Not Found	Not Found	Not Found	Not Found	Not Found
High	High	High	Not Found	Not Found	Not Found	Not Found	Not Found	Not Found	Not Found
Not Found	Not Found	Not Found	High	High	High	High	Not Found	Not Found	Not Found
High	High	High	High	High	High	High	Not Found	Not Found	Not Found
Not Found	Not Found	Not Found	Not Found	Not Found	Not Found	Not Found	High	High	High
Not Found	Not Found	Not Found	Not Found	Not Found	Not Found	Not Found	Not Found	Not Found	Not Found
Not Found	Not Found	Not Found	Not Found	Not Found	Not Found	Not Found	Not Found	Not Found	Not Found
Not Found	Not Found	Not Found	High	High	High	High	Not Found	Not Found	Not Found
Not Found	Not Found	Not Found	Not Found	Not Found	Not Found	Not Found	High	High	High
Not Found	Not Found	Not Found	Not Found	Not Found	Not Found	Not Found	Not Found	Not Found	Not Found
Not Found	Not Found	Not Found	High	High	High	High	Not Found	Not Found	Not Found
Not Found	Not Found	Not Found	Not Found	Not Found	Not Found	Not Found	Not Found	Not Found	Not Found
High	High	High	High	High	High	High	Not Found	Not Found	Not Found
Not Found	Not Found	Not Found	Not Found	Not Found	Not Found	Not Found	High	High	High
High	High	High	Not Found	Not Found	Not Found	Not Found	Not Found	Not Found	Not Found
Not Found	Not Found	Not Found	Not Found	Not Found	Not Found	Not Found	High	High	High
Not Found	Not Found	Not Found	High	High	High	High	High	High	High
Not Found	Not Found	Not Found	Not Found	Not Found	Not Found	Not Found	High	High	High
Not Found	Not Found	Not Found	High	High	High	High	Not Found	Not Found	Not Found
Not Found	Not Found	Not Found	Not Found	Not Found	Not Found	Not Found	Not Found	Not Found	Not Found
High	High	High	Not Found	Not Found	Not Found	Not Found	Not Found	Not Found	Not Found
Not Found	Not Found	Not Found	Not Found	Not Found	Not Found	Not Found	Not Found	Not Found	Not Found
High	High	High	Not Found	Not Found	Not Found	Not Found	Not Found	Not Found	Not Found
High	High	High	Not Found	Not Found	Not Found	Not Found	Not Found	Not Found	Not Found
High	High	High	Not Found	Not Found	Not Found	Not Found	High	High	High
High	High	High	Not Found	Not Found	Not Found	Not Found	High	High	High
Not Found	Not Found	Not Found	High	High	High	High	Not Found	Not Found	Not Found
Not Found	Not Found	Not Found	High	High	High	High	Not Found	Not Found	Not Found
Not Found	Not Found	Not Found	High	High	High	High	Not Found	Not Found	Not Found
Not Found	Not Found	Not Found	Not Found	Not Found	Not Found	Not Found	High	High	High
Not Found	Not Found	Not Found	Not Found	Not Found	Not Found	Not Found	Not Found	Not Found	Not Found
High	High	High	Not Found	Not Found	Not Found	Not Found	Not Found	Not Found	Not Found
Not Found	Not Found	Not Found	High	High	High	High	Not Found	Not Found	Not Found

High	High	High	High	High	High	High	Not Found	Not Found	Not Found
Not Found	Not Found	Not Found	Not Found	Not Found	Not Found	Not Found	Not Found	Not Found	Not Found
High	High	High	High	High	High	High	Not Found	Not Found	Not Found
High	High	High	High	High	High	High	Not Found	Not Found	Not Found
Not Found	Not Found	Not Found	High	High	High	High	High	High	High
High	High	High	Not Found	Not Found	Not Found	Not Found	High	High	High
Not Found	Not Found	Not Found	High	High	High	High	Not Found	Not Found	Not Found
Not Found	Not Found	Not Found	Not Found	Not Found	Not Found	Not Found	High	High	High
Not Found	Not Found	Not Found	High	High	High	High	Not Found	Not Found	Not Found
High	High	High	Not Found	Not Found	Not Found	Not Found	High	High	High
Not Found	Not Found	Not Found	High	High	High	High	High	High	High
Not Found	Not Found	Not Found	High	High	High	High	High	High	High
Not Found	Not Found	Not Found	Not Found	Not Found	Not Found	Not Found	High	High	High
Not Found	Not Found	Not Found	High	High	High	High	Not Found	Not Found	Not Found
Not Found	Not Found	Not Found	High	High	High	High	Not Found	Not Found	Not Found
Not Found	Not Found	Not Found	Not Found	Not Found	Not Found	Not Found	Not Found	Not Found	Not Found
High	High	High	Not Found	Not Found	Not Found	Not Found	Not Found	Not Found	Not Found
High	High	High	Not Found	Not Found	Not Found	Not Found	Not Found	Not Found	Not Found
Not Found	Not Found	Not Found	Not Found	Not Found	Not Found	Not Found	High	High	High
High	High	High	Not Found	Not Found	Not Found	Not Found	Not Found	Not Found	Not Found
Not Found	Not Found	Not Found	Not Found	Not Found	Not Found	Not Found	Not Found	Not Found	Not Found
Not Found	Not Found	Not Found	High	High	High	High	High	High	High
Not Found	Not Found	Not Found	High	High	High	High	Not Found	Not Found	Not Found
High	High	High	Not Found	Not Found	Not Found	Not Found	High	High	High
Not Found	Not Found	Not Found	Not Found	Not Found	Not Found	Not Found	Not Found	Not Found	Not Found
Not Found	Not Found	Not Found	High	High	High	High	Not Found	Not Found	Not Found
High	High	High	Not Found	Not Found	Not Found	Not Found	Not Found	Not Found	Not Found
Not Found	Not Found	Not Found	Not Found	Not Found	Not Found	Not Found	High	High	High
Not Found	Not Found	Not Found	High	High	High	High	Not Found	Not Found	Not Found
Not Found	Not Found	Not Found	Not Found	Not Found	Not Found	Not Found	High	High	High
High	High	High	Not Found	Not Found	Not Found	Not Found	High	High	High
High	High	High	Not Found	Not Found	Not Found	Not Found	Not Found	Not Found	Not Found
High	High	High	Not Found	Not Found	Not Found	Not Found	High	High	High
Not Found	Not Found	Not Found	Not Found	Not Found	Not Found	Not Found	High	High	High
Not Found	Not Found	Not Found	High	High	High	High	Not Found	Not Found	Not Found
Not Found	Not Found	Not Found	High	High	High	High	Not Found	Not Found	Not Found
High	High	High	High	High	High	High	Not Found	Not Found	Not Found
Not Found	Not Found	Not Found	High	High	High	High	High	High	High
High	High	High	Not Found	Not Found	Not Found	Not Found	High	High	High
Not Found	Not Found	Not Found	Not Found	Not Found	Not Found	Not Found	High	High	High
Not Found	Not Found	Not Found	Not Found	Not Found	Not Found	Not Found	High	High	High
High	High	High	Not Found	Not Found	Not Found	Not Found	High	High	High
High	High	High	Not Found	Not Found	Not Found	Not Found	High	High	High
High	High	High	Not Found	Not Found	Not Found	Not Found	Not Found	Not Found	Not Found
High	High	High	Not Found	Not Found	Not Found	Not Found	High	High	High
Not Found	Not Found	Not Found	High	High	High	High	Not Found	Not Found	Not Found
Not Found	Not Found	Not Found	Not Found	Not Found	Not Found	Not Found	High	High	High
Not Found	Not Found	Not Found	High	High	High	High	Not Found	Not Found	Not Found
High	High	High	Not Found	Not Found	Not Found	Not Found	Not Found	Not Found	Not Found
High	High	High	High	High	High	High	Not Found	Not Found	Not Found

High	High	High	Not Found	Not Found	Not Found	Not Found	High	High	High
High	High	High	Not Found	Not Found	Not Found	Not Found	Not Found	Not Found	Not Found
Not Found	Not Found	Not Found	Not Found	Not Found	Not Found	Not Found	High	High	High
High	High	High	Not Found	Not Found	Not Found	Not Found	Not Found	Not Found	Not Found
Not Found	Not Found	Not Found	High	High	High	High	High	High	High
High	High	High	High	High	High	High	Not Found	Not Found	Not Found
High	High	High	High	High	High	High	Not Found	Not Found	Not Found
Not Found	Not Found	Not Found	High	High	High	High	High	High	High
Not Found	Not Found	Not Found	Not Found	Not Found	Not Found	Not Found	High	High	High
High	High	High	Not Found	Not Found	Not Found	Not Found	Not Found	Not Found	Not Found
High	High	High	Not Found	Not Found	Not Found	Not Found	Not Found	Not Found	Not Found
Not Found	Not Found	Not Found	Not Found	Not Found	Not Found	Not Found	High	High	High
High	High	High	Not Found	Not Found	Not Found	Not Found	Not Found	Not Found	Not Found
Not Found	Not Found	Not Found	High	High	High	High	Not Found	Not Found	Not Found
Not Found	Not Found	Not Found	Not Found	Not Found	Not Found	Not Found	High	High	High
Not Found	Not Found	Not Found	High	High	High	High	Not Found	Not Found	Not Found
High	High	High	Not Found	Not Found	Not Found	Not Found	Not Found	Not Found	Not Found
Not Found	Not Found	Not Found	Not Found	Not Found	Not Found	Not Found	High	High	High
High	High	High	Not Found	Not Found	Not Found	Not Found	Not Found	Not Found	Not Found
Not Found	Not Found	Not Found	High	High	High	High	Not Found	Not Found	Not Found
High	High	High	Not Found	Not Found	Not Found	Not Found	Not Found	Not Found	Not Found
Not Found	Not Found	Not Found	High	High	High	High	Not Found	Not Found	Not Found
Not Found	Not Found	Not Found	High	High	High	High	Not Found	Not Found	Not Found
Not Found	Not Found	Not Found	Not Found	Not Found	Not Found	Not Found	High	High	High
Not Found	Not Found	Not Found	High	High	High	High	Not Found	Not Found	Not Found
Not Found	Not Found	Not Found	Not Found	Not Found	Not Found	Not Found	High	High	High
Not Found	Not Found	Not Found	Not Found	Not Found	Not Found	Not Found	High	High	High
Not Found	Not Found	Not Found	High	High	High	High	Not Found	Not Found	Not Found
Not Found	Not Found	Not Found	Not Found	Not Found	Not Found	Not Found	High	High	High
Not Found	Not Found	Not Found	High	High	High	High	Not Found	Not Found	Not Found
Not Found	Not Found	Not Found	Not Found	Not Found	Not Found	Not Found	High	High	High
Not Found	Not Found	Not Found	High	High	High	High	Not Found	Not Found	Not Found
High	High	High	Not Found	Not Found	Not Found	Not Found	Not Found	Not Found	Not Found
Not Found	Not Found	Not Found	Not Found	Not Found	Not Found	Not Found	High	High	High
Not Found	Not Found	Not Found	High	High	High	High	Not Found	Not Found	Not Found
Not Found	Not Found	Not Found	Not Found	Not Found	Not Found	Not Found	High	High	High
Not Found	Not Found	Not Found	High	High	High	High	Not Found	Not Found	Not Found
High	High	High	Not Found	Not Found	Not Found	Not Found	Not Found	Not Found	Not Found
Not Found	Not Found	Not Found	Not Found	Not Found	Not Found	Not Found	High	High	High
Not Found	Not Found	Not Found	High	High	High	High	Not Found	Not Found	Not Found
Not Found	Not Found	Not Found	High	High	High	High	Not Found	Not Found	Not Found
High	High	High	Not Found	Not Found	Not Found	Not Found	Not Found	Not Found	Not Found
High	High	High	Not Found	Not Found	Not Found	Not Found	Not Found	Not Found	Not Found
Not Found	Not Found	Not Found	Not Found	Not Found	Not Found	Not Found	High	High	High
Not Found	Not Found	Not Found	Not Found	Not Found	Not Found	Not Found	High	High	High
High	High	High	Not Found	Not Found	Not Found	Not Found	Not Found	Not Found	Not Found
Not Found	Not Found	Not Found	High	High	High	High	Not Found	Not Found	Not Found
High	High	High	Not Found	Not Found	Not Found	Not Found	Not Found	Not Found	Not Found
Not Found	Not Found	Not Found	High	High	High	High	Not Found	Not Found	Not Found
High	High	High	Not Found	Not Found	Not Found	Not Found	Not Found	Not Found	Not Found

Not Found	Not Found	Not Found	Not Found	Not Found	Not Found	Not Found	High	High	High
Not Found	Not Found	Not Found	High	High	High	High	Not Found	Not Found	Not Found
Not Found	Not Found	Not Found	Not Found	Not Found	Not Found	Not Found	High	High	High
Not Found	Not Found	Not Found	High	High	High	High	Not Found	Not Found	Not Found
Not Found	Not Found	Not Found	Not Found	Not Found	Not Found	Not Found	High	High	High
Not Found	Not Found	Not Found	Not Found	Not Found	Not Found	Not Found	High	High	High
Not Found	Not Found	Not Found	High	High	High	High	Not Found	Not Found	Not Found
Not Found	Not Found	Not Found	High	High	High	High	Not Found	Not Found	Not Found
Not Found	Not Found	Not Found	High	High	High	High	Not Found	Not Found	Not Found
Not Found	Not Found	Not Found	Not Found	Not Found	Not Found	Not Found	High	High	High
Not Found	Not Found	Not Found	High	High	High	High	Not Found	Not Found	Not Found
Not Found	Not Found	Not Found	High	High	High	High	Not Found	Not Found	Not Found
Not Found	Not Found	Not Found	High	High	High	High	Not Found	Not Found	Not Found
High	High	High	Not Found	Not Found	Not Found	Not Found	Not Found	Not Found	Not Found
Not Found	Not Found	Not Found	High	High	High	High	Not Found	Not Found	Not Found
Not Found	Not Found	Not Found	Not Found	Not Found	Not Found	Not Found	High	High	High
Not Found	Not Found	Not Found	High	High	High	High	Not Found	Not Found	Not Found
Not Found	Not Found	Not Found	Not Found	Not Found	Not Found	Not Found	High	High	High
Not Found	Not Found	Not Found	High	High	High	High	Not Found	Not Found	Not Found
High	High	High	Not Found	Not Found	Not Found	Not Found	Not Found	Not Found	Not Found
Not Found	Not Found	Not Found	Not Found	Not Found	Not Found	Not Found	High	High	High
Not Found	Not Found	Not Found	Not Found	Not Found	Not Found	Not Found	High	High	High
High	High	High	Not Found	Not Found	Not Found	Not Found	Not Found	Not Found	Not Found
High	High	High	Not Found	Not Found	Not Found	Not Found	Not Found	Not Found	Not Found
High	High	High	Not Found	Not Found	Not Found	Not Found	Not Found	Not Found	Not Found
Not Found	Not Found	Not Found	High	High	High	High	Not Found	Not Found	Not Found
Not Found	Not Found	Not Found	Not Found	Not Found	Not Found	Not Found	High	High	High
Not Found	Not Found	Not Found	High	High	High	High	Not Found	Not Found	Not Found
High	High	High	Not Found	Not Found	Not Found	Not Found	Not Found	Not Found	Not Found
Not Found	Not Found	Not Found	High	High	High	High	Not Found	Not Found	Not Found
High	High	High	Not Found	Not Found	Not Found	Not Found	Not Found	Not Found	Not Found
High	High	High	Not Found	Not Found	Not Found	Not Found	Not Found	Not Found	Not Found
Not Found	Not Found	Not Found	Not Found	Not Found	Not Found	Not Found	High	High	High
High	High	High	Not Found	Not Found	Not Found	Not Found	Not Found	Not Found	Not Found
Not Found	Not Found	Not Found	Not Found	Not Found	Not Found	Not Found	High	High	High
High	High	High	Not Found	Not Found	Not Found	Not Found	Not Found	Not Found	Not Found
High	High	High	Not Found	Not Found	Not Found	Not Found	Not Found	Not Found	Not Found
Not Found	Not Found	Not Found	Not Found	Not Found	Not Found	Not Found	High	High	High
High	High	High	Not Found	Not Found	Not Found	Not Found	Not Found	Not Found	Not Found
Not Found	Not Found	Not Found	Not Found	Not Found	Not Found	Not Found	Not Found	Not Found	Not Found
High	High	High	Not Found	Not Found	Not Found	Not Found	Not Found	Not Found	Not Found
Not Found	Not Found	Not Found	Not Found	Not Found	Not Found	Not Found	High	High	High
Not Found	Not Found	Not Found	High	High	High	High	Not Found	Not Found	Not Found
Not Found	Not Found	Not Found	High	High	High	High	Not Found	Not Found	Not Found
Not Found	Not Found	Not Found	Not Found	Not Found	Not Found	Not Found	High	High	High
High	High	High	Not Found	Not Found	Not Found	Not Found	Not Found	Not Found	Not Found
Not Found	Not Found	Not Found	High	High	High	High	Not Found	Not Found	Not Found
High	High	High	Not Found	Not Found	Not Found	Not Found	Not Found	Not Found	Not Found
Not Found	Not Found	Not Found	Not Found	Not Found	Not Found	Not Found	High	High	High
Not Found	Not Found	Not Found	Not Found	Not Found	Not Found	Not Found	High	High	High

Not Found	Not Found	Not Found	High	High	High	High	Not Found	Not Found	Not Found
Not Found	Not Found	Not Found	High	High	High	High	Not Found	Not Found	Not Found
High	High	High	Not Found	Not Found	Not Found	Not Found	Not Found	Not Found	Not Found
High	High	High	Not Found	Not Found	Not Found	Not Found	Not Found	Not Found	Not Found
Not Found	Not Found	Not Found	Not Found	Not Found	Not Found	Not Found	High	High	High
Not Found	Not Found	Not Found	Not Found	Not Found	Not Found	Not Found	High	High	High
Not Found	Not Found	Not Found	Not Found	Not Found	Not Found	Not Found	High	High	High
Not Found	Not Found	Not Found	Not Found	Not Found	Not Found	Not Found	High	High	High
Not Found	Not Found	Not Found	Not Found	Not Found	Not Found	Not Found	High	High	High
High	High	High	Not Found	Not Found	Not Found	Not Found	Not Found	Not Found	Not Found
Not Found	Not Found	Not Found	Not Found	Not Found	Not Found	Not Found	High	High	High
Not Found	Not Found	Not Found	High	High	High	High	Not Found	Not Found	Not Found
Not Found	Not Found	Not Found	High	High	High	High	Not Found	Not Found	Not Found
Not Found	Not Found	Not Found	Not Found	Not Found	Not Found	Not Found	Not Found	Not Found	Not Found
High	High	High	Not Found	Not Found	Not Found	Not Found	Not Found	Not Found	Not Found
High	High	High	Not Found	Not Found	Not Found	Not Found	Not Found	Not Found	Not Found
High	High	High	Not Found	Not Found	Not Found	Not Found	Not Found	Not Found	Not Found
High	High	High	Not Found	Not Found	Not Found	Not Found	Not Found	Not Found	Not Found
Not Found	Not Found	Not Found	High	High	High	High	Not Found	Not Found	Not Found
Not Found	Not Found	Not Found	High	High	High	High	Not Found	Not Found	Not Found
High	High	High	Not Found	Not Found	Not Found	Not Found	Not Found	Not Found	Not Found
Not Found	Not Found	Not Found	High	High	High	High	Not Found	Not Found	Not Found
Not Found	Not Found	Not Found	High	High	High	High	Not Found	Not Found	Not Found
Not Found	Not Found	Not Found	Not Found	Not Found	Not Found	Not Found	High	High	High
Not Found	Not Found	Not Found	Not Found	Not Found	Not Found	Not Found	High	High	High
High	High	High	Not Found	Not Found	Not Found	Not Found	Not Found	Not Found	Not Found
Not Found	Not Found	Not Found	High	High	High	High	Not Found	Not Found	Not Found
Not Found	Not Found	Not Found	Not Found	Not Found	Not Found	Not Found	High	High	High
High	High	High	Not Found	Not Found	Not Found	Not Found	Not Found	Not Found	Not Found
Not Found	Not Found	Not Found	Not Found	Not Found	Not Found	Not Found	Not Found	Not Found	Not Found
Not Found	Not Found	Not Found	High	High	High	High	Not Found	Not Found	Not Found
Not Found	Not Found	Not Found	High	High	High	High	Not Found	Not Found	Not Found
Not Found	Not Found	Not Found	High	High	High	High	Not Found	Not Found	Not Found
High	High	High	Not Found	Not Found	Not Found	Not Found	Not Found	Not Found	Not Found
Not Found	Not Found	Not Found	Not Found	Not Found	Not Found	Not Found	High	High	High
Not Found	Not Found	Not Found	High	High	High	High	Not Found	Not Found	Not Found
High	High	High	Not Found	Not Found	Not Found	Not Found	Not Found	Not Found	Not Found
Not Found	Not Found	Not Found	High	High	High	High	Not Found	Not Found	Not Found
Not Found	Not Found	Not Found	High	High	High	High	Not Found	Not Found	Not Found
Not Found	Not Found	Not Found	Not Found	Not Found	Not Found	Not Found	High	High	High
Not Found	Not Found	Not Found	Not Found	Not Found	Not Found	Not Found	High	High	High
Not Found	Not Found	Not Found	High	High	High	High	Not Found	Not Found	Not Found
Not Found	Not Found	Not Found	High	High	High	High	Not Found	Not Found	Not Found
Not Found	Not Found	Not Found	High	High	High	High	Not Found	Not Found	Not Found
Not Found	Not Found	Not Found	Not Found	Not Found	Not Found	Not Found	High	High	High
Not Found	Not Found	Not Found	Not Found	Not Found	Not Found	Not Found	High	High	High
Not Found	Not Found	Not Found	Not Found	Not Found	Not Found	Not Found	Not Found	Not Found	Not Found
Not Found	Not Found	Not Found	Not Found	Not Found	Not Found	Not Found	Not Found	Not Found	Not Found
Not Found	Not Found	Not Found	Not Found	Not Found	Not Found	Not Found	Not Found	Not Found	Not Found
Not Found	Not Found	Not Found	Not Found	Not Found	Not Found	Not Found	High	High	High

Not Found	Not Found	Not Found	Not Found	Not Found	Not Found	Not Found	Not Found	Not Found	Not Found
High	High	High	Not Found	Not Found	Not Found	Not Found	Not Found	Not Found	Not Found
Not Found	Not Found	Not Found	High	High	High	High	Not Found	Not Found	Not Found
Not Found	Not Found	Not Found	High	High	High	High	Not Found	Not Found	Not Found
Not Found	Not Found	Not Found	Not Found	Not Found	Not Found	Not Found	High	High	High
High	High	High	Not Found	Not Found	Not Found	Not Found	Not Found	Not Found	Not Found
High	High	High	Not Found	Not Found	Not Found	Not Found	Not Found	Not Found	Not Found
High	High	High	Not Found	Not Found	Not Found	Not Found	Not Found	Not Found	Not Found
Not Found	Not Found	Not Found	Not Found	Not Found	Not Found	Not Found	High	High	High
Not Found	Not Found	Not Found	Not Found	Not Found	Not Found	Not Found	High	High	High
Not Found	Not Found	Not Found	Not Found	Not Found	Not Found	Not Found	High	High	High
High	High	High	Not Found	Not Found	Not Found	Not Found	Not Found	Not Found	Not Found
Not Found	Not Found	Not Found	Not Found	Not Found	Not Found	Not Found	Not Found	Not Found	Not Found
Not Found	Not Found	Not Found	Not Found	Not Found	Not Found	Not Found	High	High	High
High	High	High	Not Found	Not Found	Not Found	Not Found	Not Found	Not Found	Not Found
Not Found	Not Found	Not Found	Not Found	Not Found	Not Found	Not Found	High	High	High
High	High	High	Not Found	Not Found	Not Found	Not Found	Not Found	Not Found	Not Found
Not Found	Not Found	Not Found	Not Found	Not Found	Not Found	Not Found	High	High	High
High	High	High	Not Found	Not Found	Not Found	Not Found	Not Found	Not Found	Not Found
Not Found	Not Found	Not Found	High	High	High	High	Not Found	Not Found	Not Found
Not Found	Not Found	Not Found	High	High	High	High	Not Found	Not Found	Not Found
Not Found	Not Found	Not Found	Not Found	Not Found	Not Found	Not Found	Not Found	Not Found	Not Found
Not Found	Not Found	Not Found	High	High	High	High	Not Found	Not Found	Not Found
Not Found	Not Found	Not Found	High	High	High	High	Not Found	Not Found	Not Found
Not Found	Not Found	Not Found	Not Found	Not Found	Not Found	Not Found	Not Found	Not Found	Not Found
Not Found	Not Found	Not Found	High	High	High	High	Not Found	Not Found	Not Found
Not Found	Not Found	Not Found	High	High	High	High	Not Found	Not Found	Not Found
Not Found	Not Found	Not Found	Not Found	Not Found	Not Found	Not Found	High	High	High
Not Found	Not Found	Not Found	High	High	High	High	Not Found	Not Found	Not Found
Not Found	Not Found	Not Found	Not Found	Not Found	Not Found	Not Found	High	High	High
Not Found	Not Found	Not Found	High	High	High	High	Not Found	Not Found	Not Found
Not Found	Not Found	Not Found	High	High	High	High	Not Found	Not Found	Not Found
Not Found	Not Found	Not Found	High	High	High	High	Not Found	Not Found	Not Found
High	High	High	Not Found	Not Found	Not Found	Not Found	Not Found	Not Found	Not Found
Not Found	Not Found	Not Found	High	High	High	High	Not Found	Not Found	Not Found
High	High	High	Not Found	Not Found	Not Found	Not Found	Not Found	Not Found	Not Found
High	High	High	Not Found	Not Found	Not Found	Not Found	Not Found	Not Found	Not Found
Not Found	Not Found	Not Found	High	High	High	High	Not Found	Not Found	Not Found
Not Found	Not Found	Not Found	Not Found	Not Found	Not Found	Not Found	High	High	High
Not Found	Not Found	Not Found	High	High	High	High	Not Found	Not Found	Not Found
Not Found	Not Found	Not Found	Not Found	Not Found	Not Found	Not Found	High	High	High
High	High	High	Not Found	Not Found	Not Found	Not Found	Not Found	Not Found	Not Found
High	High	High	Not Found	Not Found	Not Found	Not Found	Not Found	Not Found	Not Found
Not Found	Not Found	Not Found	High	High	High	High	Not Found	Not Found	Not Found
Not Found	Not Found	Not Found	Not Found	Not Found	Not Found	Not Found	High	High	High
High	High	High	Not Found	Not Found	Not Found	Not Found	Not Found	Not Found	Not Found
High	High	High	Not Found	Not Found	Not Found	Not Found	Not Found	Not Found	Not Found
Not Found	Not Found	Not Found	Not Found	Not Found	Not Found	Not Found	Not Found	Not Found	Not Found
High	High	High	Not Found	Not Found	Not Found	Not Found	Not Found	Not Found	Not Found
Not Found	Not Found	Not Found	Not Found	Not Found	Not Found	Not Found	High	High	High

Not Found	Not Found	Not Found	Not Found	Not Found	Not Found	Not Found	High	High	High
Not Found	Not Found	Not Found	High	High	High	High	Not Found	Not Found	Not Found
High	High	High	Not Found	Not Found	Not Found	Not Found	Not Found	Not Found	Not Found
Not Found	Not Found	Not Found	Not Found	Not Found	Not Found	Not Found	High	High	High
Not Found	Not Found	Not Found	High	High	High	High	Not Found	Not Found	Not Found
Not Found	Not Found	Not Found	Not Found	Not Found	Not Found	Not Found	Not Found	Not Found	Not Found
Not Found	Not Found	Not Found	Not Found	Not Found	Not Found	Not Found	Not Found	Not Found	Not Found
Not Found	Not Found	Not Found	Not Found	Not Found	Not Found	Not Found	Not Found	Not Found	Not Found
Not Found	Not Found	Not Found	Not Found	Not Found	Not Found	Not Found	High	High	High
Not Found	Not Found	Not Found	Not Found	Not Found	Not Found	Not Found	High	High	High
High	High	High	Not Found	Not Found	Not Found	Not Found	Not Found	Not Found	Not Found
Not Found	Not Found	Not Found	High	High	High	High	Not Found	Not Found	Not Found
High	High	High	Not Found	Not Found	Not Found	Not Found	Not Found	Not Found	Not Found
Not Found	Not Found	Not Found	Not Found	Not Found	Not Found	Not Found	High	High	High
High	High	High	Not Found	Not Found	Not Found	Not Found	Not Found	Not Found	Not Found
Not Found	Not Found	Not Found	Not Found	Not Found	Not Found	Not Found	Not Found	Not Found	Not Found
Not Found	Not Found	Not Found	Not Found	Not Found	Not Found	Not Found	High	High	High
Not Found	Not Found	Not Found	Not Found	Not Found	Not Found	Not Found	High	High	High
High	High	High	Not Found	Not Found	Not Found	Not Found	Not Found	Not Found	Not Found
Not Found	Not Found	Not Found	Not Found	Not Found	Not Found	Not Found	High	High	High
Not Found	Not Found	Not Found	Not Found	Not Found	Not Found	Not Found	High	High	High
Not Found	Not Found	Not Found	High	High	High	High	Not Found	Not Found	Not Found
Not Found	Not Found	Not Found	High	High	High	High	Not Found	Not Found	Not Found
Not Found	Not Found	Not Found	Not Found	Not Found	Not Found	Not Found	Not Found	Not Found	Not Found
High	High	High	Not Found	Not Found	Not Found	Not Found	Not Found	Not Found	Not Found
Not Found	Not Found	Not Found	Not Found	Not Found	Not Found	Not Found	High	High	High
Not Found	Not Found	Not Found	Not Found	Not Found	Not Found	Not Found	High	High	High
Not Found	Not Found	Not Found	High	High	High	High	Not Found	Not Found	Not Found
Not Found	Not Found	Not Found	Not Found	Not Found	Not Found	Not Found	High	High	High
High	High	High	Not Found	Not Found	Not Found	Not Found	Not Found	Not Found	Not Found
Not Found	Not Found	Not Found	Not Found	Not Found	Not Found	Not Found	Not Found	Not Found	Not Found
Not Found	Not Found	Not Found	Not Found	Not Found	Not Found	Not Found	Not Found	Not Found	Not Found
Not Found	Not Found	Not Found	High	High	High	High	Not Found	Not Found	Not Found
Not Found	Not Found	Not Found	Not Found	Not Found	Not Found	Not Found	Not Found	Not Found	Not Found
High	High	High	Not Found	Not Found	Not Found	Not Found	Not Found	Not Found	Not Found
Not Found	Not Found	Not Found	Not Found	Not Found	Not Found	Not Found	High	High	High
Not Found	Not Found	Not Found	Not Found	Not Found	Not Found	Not Found	High	High	High
Not Found	Not Found	Not Found	Not Found	Not Found	Not Found	Not Found	High	High	High
High	High	High	Not Found	Not Found	Not Found	Not Found	Not Found	Not Found	Not Found
Not Found	Not Found	Not Found	High	High	High	High	Not Found	Not Found	Not Found
Not Found	Not Found	Not Found	High	High	High	High	Not Found	Not Found	Not Found
High	High	High	Not Found	Not Found	Not Found	Not Found	Not Found	Not Found	Not Found
Not Found	Not Found	Not Found	High	High	High	High	Not Found	Not Found	Not Found
High	High	High	Not Found	Not Found	Not Found	Not Found	Not Found	Not Found	Not Found
High	High	High	Not Found	Not Found	Not Found	Not Found	Not Found	Not Found	Not Found
Not Found	Not Found	Not Found	High	High	High	High	Not Found	Not Found	Not Found
Not Found	Not Found	Not Found	High	High	High	High	Not Found	Not Found	Not Found
Not Found	Not Found	Not Found	High	High	High	High	Not Found	Not Found	Not Found
High	High	High	Not Found	Not Found	Not Found	Not Found	Not Found	Not Found	Not Found
Not Found	Not Found	Not Found	Not Found	Not Found	Not Found	Not Found	High	High	High

Not Found	Not Found	Not Found	High	High	High	High	Not Found	Not Found	Not Found
Not Found	Not Found	Not Found	Not Found	Not Found	Not Found	Not Found	High	High	High
Not Found	Not Found	Not Found	Not Found	Not Found	Not Found	Not Found	Not Found	Not Found	Not Found
High	High	High	Not Found	Not Found	Not Found	Not Found	Not Found	Not Found	Not Found
Not Found	Not Found	Not Found	Not Found	Not Found	Not Found	Not Found	Not Found	Not Found	Not Found
Not Found	Not Found	Not Found	Not Found	Not Found	Not Found	Not Found	High	High	High
Not Found	Not Found	Not Found	High	High	High	High	Not Found	Not Found	Not Found
Not Found	Not Found	Not Found	Not Found	Not Found	Not Found	Not Found	Not Found	Not Found	Not Found
Not Found	Not Found	Not Found	Not Found	Not Found	Not Found	Not Found	High	High	High
Not Found	Not Found	Not Found	High	High	High	High	Not Found	Not Found	Not Found
Not Found	Not Found	Not Found	Not Found	Not Found	Not Found	Not Found	High	High	High
Not Found	Not Found	Not Found	High	High	High	High	Not Found	Not Found	Not Found
Not Found	Not Found	Not Found	Not Found	Not Found	Not Found	Not Found	High	High	High
Not Found	Not Found	Not Found	High	High	High	High	Not Found	Not Found	Not Found
Not Found	Not Found	Not Found	High	High	High	High	Not Found	Not Found	Not Found
Not Found	Not Found	Not Found	Not Found	Not Found	Not Found	Not Found	High	High	High
Not Found	Not Found	Not Found	Not Found	Not Found	Not Found	Not Found	High	High	High
Not Found	Not Found	Not Found	High	High	High	High	Not Found	Not Found	Not Found
High	High	High	Not Found	Not Found	Not Found	Not Found	Not Found	Not Found	Not Found
Not Found	Not Found	Not Found	Not Found	Not Found	Not Found	Not Found	Not Found	Not Found	Not Found
Not Found	Not Found	Not Found	High	High	High	High	Not Found	Not Found	Not Found
Not Found	Not Found	Not Found	Not Found	Not Found	Not Found	Not Found	Not Found	Not Found	Not Found
Not Found	Not Found	Not Found	High	High	High	High	Not Found	Not Found	Not Found
Not Found	Not Found	Not Found	High	High	High	High	Not Found	Not Found	Not Found
Not Found	Not Found	Not Found	Not Found	Not Found	Not Found	Not Found	Not Found	Not Found	Not Found
Not Found	Not Found	Not Found	Not Found	Not Found	Not Found	Not Found	High	High	High
High	High	High	Not Found	Not Found	Not Found	Not Found	Not Found	Not Found	Not Found
Not Found	Not Found	Not Found	High	High	High	High	Not Found	Not Found	Not Found
Not Found	Not Found	Not Found	Not Found	Not Found	Not Found	Not Found	Not Found	Not Found	Not Found
High	High	High	Not Found	Not Found	Not Found	Not Found	Not Found	Not Found	Not Found
High	High	High	Not Found	Not Found	Not Found	Not Found	Not Found	Not Found	Not Found
High	High	High	Not Found	Not Found	Not Found	Not Found	Not Found	Not Found	Not Found
High	High	High	Not Found	Not Found	Not Found	Not Found	Not Found	Not Found	Not Found
Not Found	Not Found	Not Found	Not Found	Not Found	Not Found	Not Found	High	High	High
Not Found	Not Found	Not Found	Not Found	Not Found	Not Found	Not Found	High	High	High
High	High	High	Not Found	Not Found	Not Found	Not Found	Not Found	Not Found	Not Found
Not Found	Not Found	Not Found	High	High	High	High	Not Found	Not Found	Not Found
Not Found	Not Found	Not Found	Not Found	Not Found	Not Found	Not Found	High	High	High
Not Found	Not Found	Not Found	High	High	High	High	Not Found	Not Found	Not Found
Not Found	Not Found	Not Found	High	High	High	High	Not Found	Not Found	Not Found
High	High	High	Not Found	Not Found	Not Found	Not Found	Not Found	Not Found	Not Found
High	High	High	Not Found	Not Found	Not Found	Not Found	Not Found	Not Found	Not Found
High	High	High	Not Found	Not Found	Not Found	Not Found	Not Found	Not Found	Not Found
High	High	High	Not Found	Not Found	Not Found	Not Found	Not Found	Not Found	Not Found
High	High	High	Not Found	Not Found	Not Found	Not Found	Not Found	Not Found	Not Found
Not Found	Not Found	Not Found	Not Found	Not Found	Not Found	Not Found	High	High	High
Not Found	Not Found	Not Found	Not Found	Not Found	Not Found	Not Found	Not Found	Not Found	Not Found
High	High	High	Not Found	Not Found	Not Found	Not Found	Not Found	Not Found	Not Found
Not Found	Not Found	Not Found	High	High	High	High	Not Found	Not Found	Not Found
Not Found	Not Found	Not Found	Not Found	Not Found	Not Found	Not Found	High	High	High

Not Found	Not Found	Not Found	Not Found	Not Found	Not Found	Not Found	High	High	High
Not Found	Not Found	Not Found	High	High	High	High	Not Found	Not Found	Not Found
Not Found	Not Found	Not Found	Not Found	Not Found	Not Found	Not Found	High	High	High
Not Found	Not Found	Not Found	Not Found	Not Found	Not Found	Not Found	High	High	High
Not Found	Not Found	Not Found	High	High	High	High	Not Found	Not Found	Not Found
Not Found	Not Found	Not Found	High	High	High	High	Not Found	Not Found	Not Found
Not Found	Not Found	Not Found	High	High	High	High	Not Found	Not Found	Not Found
High	High	High	Not Found	Not Found	Not Found	Not Found	Not Found	Not Found	Not Found
High	High	High	Not Found	Not Found	Not Found	Not Found	Not Found	Not Found	Not Found
Not Found	Not Found	Not Found	Not Found	Not Found	Not Found	Not Found	High	High	High
High	High	High	Not Found	Not Found	Not Found	Not Found	Not Found	Not Found	Not Found
Not Found	Not Found	Not Found	High	High	High	High	Not Found	Not Found	Not Found
Not Found	Not Found	Not Found	High	High	High	High	Not Found	Not Found	Not Found
High	High	High	Not Found	Not Found	Not Found	Not Found	Not Found	Not Found	Not Found
Not Found	Not Found	Not Found	High	High	High	High	Not Found	Not Found	Not Found
Not Found	Not Found	Not Found	Not Found	Not Found	Not Found	Not Found	High	High	High
Not Found	Not Found	Not Found	High	High	High	High	Not Found	Not Found	Not Found
Not Found	Not Found	Not Found	Not Found	Not Found	Not Found	Not Found	Not Found	Not Found	Not Found
Not Found	Not Found	Not Found	Not Found	Not Found	Not Found	Not Found	High	High	High
Not Found	Not Found	Not Found	Not Found	Not Found	Not Found	Not Found	High	High	High
Not Found	Not Found	Not Found	Not Found	Not Found	Not Found	Not Found	High	High	High
High	High	High	Not Found	Not Found	Not Found	Not Found	Not Found	Not Found	Not Found
Not Found	Not Found	Not Found	Not Found	Not Found	Not Found	Not Found	High	High	High
High	High	High	Not Found	Not Found	Not Found	Not Found	Not Found	Not Found	Not Found
High	High	High	Not Found	Not Found	Not Found	Not Found	Not Found	Not Found	Not Found
High	High	High	Not Found	Not Found	Not Found	Not Found	Not Found	Not Found	Not Found
Not Found	Not Found	Not Found	Not Found	Not Found	Not Found	Not Found	Not Found	Not Found	Not Found
Not Found	Not Found	Not Found	Not Found	Not Found	Not Found	Not Found	Not Found	Not Found	Not Found
Not Found	Not Found	Not Found	High	High	High	High	Not Found	Not Found	Not Found
High	High	High	Not Found	Not Found	Not Found	Not Found	Not Found	Not Found	Not Found
High	High	High	Not Found	Not Found	Not Found	Not Found	Not Found	Not Found	Not Found
Not Found	Not Found	Not Found	Not Found	Not Found	Not Found	Not Found	Not Found	Not Found	Not Found
Not Found	Not Found	Not Found	Not Found	Not Found	Not Found	Not Found	Not Found	Not Found	Not Found
Not Found	Not Found	Not Found	Not Found	Not Found	Not Found	Not Found	Not Found	Not Found	Not Found
Not Found	Not Found	Not Found	Not Found	Not Found	Not Found	Not Found	Not Found	Not Found	Not Found
High	High	High	Not Found	Not Found	Not Found	Not Found	Not Found	Not Found	Not Found
Not Found	Not Found	Not Found	High	High	High	High	Not Found	Not Found	Not Found
Not Found	Not Found	Not Found	Not Found	Not Found	Not Found	Not Found	Not Found	Not Found	Not Found
Not Found	Not Found	Not Found	Not Found	Not Found	Not Found	Not Found	High	High	High
Not Found	Not Found	Not Found	High	High	High	High	Not Found	Not Found	Not Found
Not Found	Not Found	Not Found	High	High	High	High	Not Found	Not Found	Not Found
High	High	High	Not Found	Not Found	Not Found	Not Found	Not Found	Not Found	Not Found
Not Found	Not Found	Not Found	Not Found	Not Found	Not Found	Not Found	High	High	High
High	High	High	Not Found	Not Found	Not Found	Not Found	Not Found	Not Found	Not Found
Not Found	Not Found	Not Found	High	High	High	High	Not Found	Not Found	Not Found
High	High	High	Not Found	Not Found	Not Found	Not Found	Not Found	Not Found	Not Found
Not Found	Not Found	Not Found	High	High	High	High	Not Found	Not Found	Not Found
Not Found	Not Found	Not Found	Not Found	Not Found	Not Found	Not Found	Not Found	Not Found	Not Found
High	High	High	Not Found	Not Found	Not Found	Not Found	Not Found	Not Found	Not Found
Not Found	Not Found	Not Found	Not Found	Not Found	Not Found	Not Found	High	High	High

Not Found	Not Found	Not Found	Not Found	Not Found	Not Found	Not Found	Not Found	Not Found	Not Found
Not Found	Not Found	Not Found	Not Found	Not Found	Not Found	Not Found	Not Found	Not Found	Not Found
Not Found	Not Found	Not Found	Not Found	Not Found	Not Found	Not Found	High	High	High
Not Found	Not Found	Not Found	Not Found	Not Found	Not Found	Not Found	High	High	High
Not Found	Not Found	Not Found	Not Found	Not Found	Not Found	Not Found	High	High	High
Not Found	Not Found	Not Found	High	High	High	High	Not Found	Not Found	Not Found
Not Found	Not Found	Not Found	High	High	High	High	Not Found	Not Found	Not Found
Not Found	Not Found	Not Found	Not Found	Not Found	Not Found	Not Found	Not Found	Not Found	Not Found
High	High	High	Not Found	Not Found	Not Found	Not Found	Not Found	Not Found	Not Found
High	High	High	Not Found	Not Found	Not Found	Not Found	Not Found	Not Found	Not Found
Not Found	Not Found	Not Found	High	High	High	High	Not Found	Not Found	Not Found
Not Found	Not Found	Not Found	High	High	High	High	Not Found	Not Found	Not Found
Not Found	Not Found	Not Found	Not Found	Not Found	Not Found	Not Found	High	High	High
High	High	High	Not Found	Not Found	Not Found	Not Found	Not Found	Not Found	Not Found
Not Found	Not Found	Not Found	Not Found	Not Found	Not Found	Not Found	Not Found	Not Found	Not Found
Not Found	Not Found	Not Found	Not Found	Not Found	Not Found	Not Found	Not Found	Not Found	Not Found
Not Found	Not Found	Not Found	Not Found	Not Found	Not Found	Not Found	Not Found	Not Found	Not Found
Not Found	Not Found	Not Found	High	High	High	High	Not Found	Not Found	Not Found
High	High	High	Not Found	Not Found	Not Found	Not Found	Not Found	Not Found	Not Found
High	High	High	Not Found	Not Found	Not Found	Not Found	Not Found	Not Found	Not Found
High	High	High	Not Found	Not Found	Not Found	Not Found	Not Found	Not Found	Not Found
Not Found	Not Found	Not Found	Not Found	Not Found	Not Found	Not Found	Not Found	Not Found	Not Found
Not Found	Not Found	Not Found	Not Found	Not Found	Not Found	Not Found	Not Found	Not Found	Not Found
Not Found	Not Found	Not Found	Not Found	Not Found	Not Found	Not Found	Not Found	Not Found	Not Found
Not Found	Not Found	Not Found	Not Found	Not Found	Not Found	Not Found	High	High	High
Not Found	Not Found	Not Found	High	High	High	High	Not Found	Not Found	Not Found
High	High	High	Not Found	Not Found	Not Found	Not Found	Not Found	Not Found	Not Found
Not Found	Not Found	Not Found	Not Found	Not Found	Not Found	Not Found	High	High	High
Not Found	Not Found	Not Found	High	High	High	High	Not Found	Not Found	Not Found
Not Found	Not Found	Not Found	High	High	High	High	Not Found	Not Found	Not Found
Not Found	Not Found	Not Found	Not Found	Not Found	Not Found	Not Found	High	High	High
Not Found	Not Found	Not Found	Not Found	Not Found	Not Found	Not Found	High	High	High
High	High	High	Not Found	Not Found	Not Found	Not Found	Not Found	Not Found	Not Found
Not Found	Not Found	Not Found	High	High	High	High	Not Found	Not Found	Not Found
Not Found	Not Found	Not Found	Not Found	Not Found	Not Found	Not Found	Not Found	Not Found	Not Found
Not Found	Not Found	Not Found	Not Found	Not Found	Not Found	Not Found	Not Found	Not Found	Not Found
Not Found	Not Found	Not Found	Not Found	Not Found	Not Found	Not Found	Not Found	Not Found	Not Found
Not Found	Not Found	Not Found	Not Found	Not Found	Not Found	Not Found	High	High	High
Not Found	Not Found	Not Found	Not Found	Not Found	Not Found	Not Found	High	High	High
Not Found	Not Found	Not Found	High	High	High	High	Not Found	Not Found	Not Found
Not Found	Not Found	Not Found	Not Found	Not Found	Not Found	Not Found	Not Found	Not Found	Not Found
Not Found	Not Found	Not Found	High	High	High	High	Not Found	Not Found	Not Found
Not Found	Not Found	Not Found	High	High	High	High	Not Found	Not Found	Not Found
Not Found	Not Found	Not Found	Not Found	Not Found	Not Found	Not Found	Not Found	Not Found	Not Found
Not Found	Not Found	Not Found	Not Found	Not Found	Not Found	Not Found	High	High	High
High	High	High	Not Found	Not Found	Not Found	Not Found	Not Found	Not Found	Not Found
Not Found	Not Found	Not Found	Not Found	Not Found	Not Found	Not Found	High	High	High
Not Found	Not Found	Not Found	High	High	High	High	Not Found	Not Found	Not Found
Not Found	Not Found	Not Found	Not Found	Not Found	Not Found	Not Found	Not Found	Not Found	Not Found
High	High	High	Not Found	Not Found	Not Found	Not Found	Not Found	Not Found	Not Found

Not Found	Not Found	Not Found	High	High	High	High	Not Found	Not Found	Not Found
High	High	High	Not Found	Not Found	Not Found	Not Found	Not Found	Not Found	Not Found
High	High	High	Not Found	Not Found	Not Found	Not Found	Not Found	Not Found	Not Found
High	High	High	Not Found	Not Found	Not Found	Not Found	Not Found	Not Found	Not Found
Not Found	Not Found	Not Found	Not Found	Not Found	Not Found	Not Found	Not Found	Not Found	Not Found
Not Found	Not Found	Not Found	High	High	High	High	Not Found	Not Found	Not Found
Not Found	Not Found	Not Found	High	High	High	High	Not Found	Not Found	Not Found
High	High	High	Not Found	Not Found	Not Found	Not Found	Not Found	Not Found	Not Found
High	High	High	Not Found	Not Found	Not Found	Not Found	Not Found	Not Found	Not Found
Not Found	Not Found	Not Found	High	High	High	High	Not Found	Not Found	Not Found
High	High	High	Not Found	Not Found	Not Found	Not Found	Not Found	Not Found	Not Found
High	High	High	Not Found	Not Found	Not Found	Not Found	Not Found	Not Found	Not Found
Not Found	Not Found	Not Found	Not Found	Not Found	Not Found	Not Found	High	High	High
Not Found	Not Found	Not Found	Not Found	Not Found	Not Found	Not Found	High	High	High
High	High	High	Not Found	Not Found	Not Found	Not Found	Not Found	Not Found	Not Found
High	High	High	Not Found	Not Found	Not Found	Not Found	Not Found	Not Found	Not Found
Not Found	Not Found	Not Found	High	High	High	High	Not Found	Not Found	Not Found
High	High	High	Not Found	Not Found	Not Found	Not Found	Not Found	Not Found	Not Found
Not Found	Not Found	Not Found	High	High	High	High	Not Found	Not Found	Not Found
Not Found	Not Found	Not Found	High	High	High	High	Not Found	Not Found	Not Found
High	High	High	Not Found	Not Found	Not Found	Not Found	Not Found	Not Found	Not Found
High	High	High	Not Found	Not Found	Not Found	Not Found	Not Found	Not Found	Not Found
High	High	High	Not Found	Not Found	Not Found	Not Found	Not Found	Not Found	Not Found
Not Found	Not Found	Not Found	High	High	High	High	Not Found	Not Found	Not Found
High	High	High	Not Found	Not Found	Not Found	Not Found	Not Found	Not Found	Not Found
Not Found	Not Found	Not Found	High	High	High	High	Not Found	Not Found	Not Found
High	High	High	Not Found	Not Found	Not Found	Not Found	Not Found	Not Found	Not Found
Not Found	Not Found	Not Found	High	High	High	High	Not Found	Not Found	Not Found
Not Found	Not Found	Not Found	High	High	High	High	Not Found	Not Found	Not Found
High	High	High	Not Found	Not Found	Not Found	Not Found	Not Found	Not Found	Not Found
Not Found	Not Found	Not Found	High	High	High	High	Not Found	Not Found	Not Found
Not Found	Not Found	Not Found	High	High	High	High	Not Found	Not Found	Not Found
Not Found	Not Found	Not Found	High	High	High	High	Not Found	Not Found	Not Found
Not Found	Not Found	Not Found	Not Found	Not Found	Not Found	Not Found	High	High	High
High	High	High	Not Found	Not Found	Not Found	Not Found	Not Found	Not Found	Not Found
High	High	High	Not Found	Not Found	Not Found	Not Found	Not Found	Not Found	Not Found
High	High	High	Not Found	Not Found	Not Found	Not Found	Not Found	Not Found	Not Found
Not Found	Not Found	Not Found	High	High	High	High	Not Found	Not Found	Not Found
Not Found	Not Found	Not Found	High	High	High	High	Not Found	Not Found	Not Found
High	High	High	Not Found	Not Found	Not Found	Not Found	Not Found	Not Found	Not Found
High	High	High	Not Found	Not Found	Not Found	Not Found	Not Found	Not Found	Not Found
Not Found	Not Found	Not Found	Not Found	Not Found	Not Found	Not Found	High	High	High
Not Found	Not Found	Not Found	High	High	High	High	Not Found	Not Found	Not Found
Not Found	Not Found	Not Found	Not Found	Not Found	Not Found	Not Found	High	High	High
Not Found	Not Found	Not Found	Not Found	Not Found	Not Found	Not Found	High	High	High
Not Found	Not Found	Not Found	High	High	High	High	Not Found	Not Found	Not Found
Not Found	Not Found	Not Found	High	High	High	High	Not Found	Not Found	Not Found
Not Found	Not Found	Not Found	Not Found	Not Found	Not Found	Not Found	High	High	High
Not Found	Not Found	Not Found	Not Found	Not Found	Not Found	Not Found	High	High	High
Not Found	Not Found	Not Found	Not Found	Not Found	Not Found	Not Found	High	High	High

Not Found	Not Found	Not Found	High	High	High	High	Not Found	Not Found	Not Found
High	High	High	Not Found	Not Found	Not Found	Not Found	Not Found	Not Found	Not Found
Not Found	Not Found	Not Found	Not Found	Not Found	Not Found	Not Found	High	High	High
Not Found	Not Found	Not Found	Not Found	Not Found	Not Found	Not Found	High	High	High
Not Found	Not Found	Not Found	High	High	High	High	Not Found	Not Found	Not Found
Not Found	Not Found	Not Found	High	High	High	High	Not Found	Not Found	Not Found
High	High	High	Not Found	Not Found	Not Found	Not Found	Not Found	Not Found	Not Found
Not Found	Not Found	Not Found	Not Found	Not Found	Not Found	Not Found	High	High	High
Not Found	Not Found	Not Found	High	High	High	High	Not Found	Not Found	Not Found
High	High	High	Not Found	Not Found	Not Found	Not Found	Not Found	Not Found	Not Found
Not Found	Not Found	Not Found	High	High	High	High	Not Found	Not Found	Not Found
High	High	High	Not Found	Not Found	Not Found	Not Found	Not Found	Not Found	Not Found
High	High	High	Not Found	Not Found	Not Found	Not Found	Not Found	Not Found	Not Found
Not Found	Not Found	Not Found	High	High	High	High	Not Found	Not Found	Not Found
High	High	High	Not Found	Not Found	Not Found	Not Found	Not Found	Not Found	Not Found
Not Found	Not Found	Not Found	Not Found	Not Found	Not Found	Not Found	High	High	High
Not Found	Not Found	Not Found	High	High	High	High	Not Found	Not Found	Not Found
Not Found	Not Found	Not Found	High	High	High	High	Not Found	Not Found	Not Found
High	High	High	Not Found	Not Found	Not Found	Not Found	Not Found	Not Found	Not Found
Not Found	Not Found	Not Found	Not Found	Not Found	Not Found	Not Found	High	High	High
High	High	High	Not Found	Not Found	Not Found	Not Found	Not Found	Not Found	Not Found
High	High	High	Not Found	Not Found	Not Found	Not Found	Not Found	Not Found	Not Found
Not Found	Not Found	Not Found	High	High	High	High	Not Found	Not Found	Not Found
Not Found	Not Found	Not Found	Not Found	Not Found	Not Found	Not Found	High	High	High
High	High	High	Not Found	Not Found	Not Found	Not Found	Not Found	Not Found	Not Found
High	High	High	Not Found	Not Found	Not Found	Not Found	Not Found	Not Found	Not Found
Not Found	Not Found	Not Found	Not Found	Not Found	Not Found	Not Found	High	High	High
High	High	High	Not Found	Not Found	Not Found	Not Found	Not Found	Not Found	Not Found
Not Found	Not Found	Not Found	Not Found	Not Found	Not Found	Not Found	High	High	High
Not Found	Not Found	Not Found	Not Found	Not Found	Not Found	Not Found	High	High	High
High	High	High	Not Found	Not Found	Not Found	Not Found	Not Found	Not Found	Not Found
Not Found	Not Found	Not Found	High	High	High	High	Not Found	Not Found	Not Found
Not Found	Not Found	Not Found	High	High	High	High	Not Found	Not Found	Not Found
High	High	High	Not Found	Not Found	Not Found	Not Found	Not Found	Not Found	Not Found
High	High	High	Not Found	Not Found	Not Found	Not Found	Not Found	Not Found	Not Found
Not Found	Not Found	Not Found	High	High	High	High	Not Found	Not Found	Not Found
Not Found	Not Found	Not Found	High	High	High	High	Not Found	Not Found	Not Found
High	High	High	Not Found	Not Found	Not Found	Not Found	Not Found	Not Found	Not Found
Not Found	Not Found	Not Found	Not Found	Not Found	Not Found	Not Found	High	High	High
Not Found	Not Found	Not Found	High	High	High	High	Not Found	Not Found	Not Found
Not Found	Not Found	Not Found	Not Found	Not Found	Not Found	Not Found	High	High	High
High	High	High	Not Found	Not Found	Not Found	Not Found	Not Found	Not Found	Not Found
High	High	High	Not Found	Not Found	Not Found	Not Found	Not Found	Not Found	Not Found
Not Found	Not Found	Not Found	Not Found	Not Found	Not Found	Not Found	High	High	High
High	High	High	Not Found	Not Found	Not Found	Not Found	Not Found	Not Found	Not Found
High	High	High	Not Found	Not Found	Not Found	Not Found	Not Found	Not Found	Not Found
Not Found	Not Found	Not Found	High	High	High	High	Not Found	Not Found	Not Found
Not Found	Not Found	Not Found	High	High	High	High	Not Found	Not Found	Not Found
High	High	High	Not Found	Not Found	Not Found	Not Found	Not Found	Not Found	Not Found
Not Found	Not Found	Not Found	High	High	High	High	Not Found	Not Found	Not Found

Not Found	Not Found	Not Found	High	High	High	High	Not Found	Not Found	Not Found
Not Found	Not Found	Not Found	High	High	High	High	Not Found	Not Found	Not Found
High	High	High	Not Found	Not Found	Not Found	Not Found	Not Found	Not Found	Not Found
Not Found	Not Found	Not Found	Not Found	Not Found	Not Found	Not Found	High	High	High
Not Found	Not Found	Not Found	High	High	High	High	Not Found	Not Found	Not Found
High	High	High	Not Found	Not Found	Not Found	Not Found	Not Found	Not Found	Not Found
Not Found	Not Found	Not Found	High	High	High	High	Not Found	Not Found	Not Found
High	High	High	Not Found	Not Found	Not Found	Not Found	Not Found	Not Found	Not Found
Not Found	Not Found	Not Found	Not Found	Not Found	Not Found	Not Found	High	High	High
Not Found	Not Found	Not Found	High	High	High	High	Not Found	Not Found	Not Found
High	High	High	Not Found	Not Found	Not Found	Not Found	Not Found	Not Found	Not Found
High	High	High	Not Found	Not Found	Not Found	Not Found	Not Found	Not Found	Not Found
High	High	High	Not Found	Not Found	Not Found	Not Found	Not Found	Not Found	Not Found
Not Found	Not Found	Not Found	Not Found	Not Found	Not Found	Not Found	High	High	High
Not Found	Not Found	Not Found	Not Found	Not Found	Not Found	Not Found	Not Found	Not Found	Not Found
High	High	High	Not Found	Not Found	Not Found	Not Found	Not Found	Not Found	Not Found
Not Found	Not Found	Not Found	High	High	High	High	Not Found	Not Found	Not Found
Not Found	Not Found	Not Found	Not Found	Not Found	Not Found	Not Found	High	High	High
Not Found	Not Found	Not Found	High	High	High	High	Not Found	Not Found	Not Found
Not Found	Not Found	Not Found	High	High	High	High	Not Found	Not Found	Not Found
High	High	High	Not Found	Not Found	Not Found	Not Found	Not Found	Not Found	Not Found
Not Found	Not Found	Not Found	Not Found	Not Found	Not Found	Not Found	Not Found	Not Found	Not Found
Not Found	Not Found	Not Found	Not Found	Not Found	Not Found	Not Found	High	High	High
Not Found	Not Found	Not Found	High	High	High	High	Not Found	Not Found	Not Found
Not Found	Not Found	Not Found	High	High	High	High	Not Found	Not Found	Not Found
High	High	High	Not Found	Not Found	Not Found	Not Found	Not Found	Not Found	Not Found
Not Found	Not Found	Not Found	Not Found	Not Found	Not Found	Not Found	High	High	High
Not Found	Not Found	Not Found	High	High	High	High	Not Found	Not Found	Not Found
Not Found	Not Found	Not Found	Not Found	Not Found	Not Found	Not Found	High	High	High
Not Found	Not Found	Not Found	High	High	High	High	Not Found	Not Found	Not Found
High	High	High	Not Found	Not Found	Not Found	Not Found	Not Found	Not Found	Not Found
Not Found	Not Found	Not Found	High	High	High	High	Not Found	Not Found	Not Found
High	High	High	Not Found	Not Found	Not Found	Not Found	Not Found	Not Found	Not Found
Not Found	Not Found	Not Found	High	High	High	High	Not Found	Not Found	Not Found
Not Found	Not Found	Not Found	High	High	High	High	Not Found	Not Found	Not Found
Not Found	Not Found	Not Found	Not Found	Not Found	Not Found	Not Found	High	High	High
Not Found	Not Found	Not Found	Not Found	Not Found	Not Found	Not Found	High	High	High
Not Found	Not Found	Not Found	Not Found	Not Found	Not Found	Not Found	High	High	High
Not Found	Not Found	Not Found	High	High	High	High	Not Found	Not Found	Not Found
High	High	High	Not Found	Not Found	Not Found	Not Found	Not Found	Not Found	Not Found
Not Found	Not Found	Not Found	Not Found	Not Found	Not Found	Not Found	High	High	High
High	High	High	Not Found	Not Found	Not Found	Not Found	Not Found	Not Found	Not Found
Not Found	Not Found	Not Found	High	High	High	High	Not Found	Not Found	Not Found
Not Found	Not Found	Not Found	High	High	High	High	Not Found	Not Found	Not Found
Not Found	Not Found	Not Found	Not Found	Not Found	Not Found	Not Found	High	High	High
Not Found	Not Found	Not Found	High	High	High	High	Not Found	Not Found	Not Found
High	High	High	Not Found	Not Found	Not Found	Not Found	Not Found	Not Found	Not Found
Not Found	Not Found	Not Found	High	High	High	High	Not Found	Not Found	Not Found
Not Found	Not Found	Not Found	Not Found	Not Found	Not Found	Not Found	High	High	High
Not Found	Not Found	Not Found	Not Found	Not Found	Not Found	Not Found	High	High	High

High	High	High	Not Found	Not Found	Not Found	Not Found	Not Found	Not Found	Not Found
Not Found	Not Found	Not Found	Not Found	Not Found	Not Found	Not Found	High	High	High
High	High	High	Not Found	Not Found	Not Found	Not Found	Not Found	Not Found	Not Found
Not Found	Not Found	Not Found	Not Found	Not Found	Not Found	Not Found	High	High	High
Not Found	Not Found	Not Found	Not Found	Not Found	Not Found	Not Found	High	High	High
Not Found	Not Found	Not Found	Not Found	Not Found	Not Found	Not Found	High	High	High
Not Found	Not Found	Not Found	Not Found	Not Found	Not Found	Not Found	High	High	High
High	High	High	Not Found	Not Found	Not Found	Not Found	Not Found	Not Found	Not Found
Not Found	Not Found	Not Found	High	High	High	High	Not Found	Not Found	Not Found
Not Found	Not Found	Not Found	High	High	High	High	Not Found	Not Found	Not Found
Not Found	Not Found	Not Found	High	High	High	High	Not Found	Not Found	Not Found
Not Found	Not Found	Not Found	High	High	High	High	Not Found	Not Found	Not Found
Not Found	Not Found	Not Found	High	High	High	High	Not Found	Not Found	Not Found
High	High	High	Not Found	Not Found	Not Found	Not Found	Not Found	Not Found	Not Found
High	High	High	Not Found	Not Found	Not Found	Not Found	Not Found	Not Found	Not Found
Not Found	Not Found	Not Found	Not Found	Not Found	Not Found	Not Found	Not Found	Not Found	Not Found
Not Found	Not Found	Not Found	High	High	High	High	Not Found	Not Found	Not Found
High	High	High	Not Found	Not Found	Not Found	Not Found	Not Found	Not Found	Not Found
High	High	High	Not Found	Not Found	Not Found	Not Found	Not Found	Not Found	Not Found
Not Found	Not Found	Not Found	High	High	High	High	Not Found	Not Found	Not Found
Not Found	Not Found	Not Found	Not Found	Not Found	Not Found	Not Found	High	High	High
Not Found	Not Found	Not Found	High	High	High	High	Not Found	Not Found	Not Found
Not Found	Not Found	Not Found	Not Found	Not Found	Not Found	Not Found	High	High	High
Not Found	Not Found	Not Found	Not Found	Not Found	Not Found	Not Found	High	High	High
Not Found	Not Found	Not Found	High	High	High	High	Not Found	Not Found	Not Found
Not Found	Not Found	Not Found	High	High	High	High	Not Found	Not Found	Not Found
Not Found	Not Found	Not Found	Not Found	Not Found	Not Found	Not Found	High	High	High
Not Found	Not Found	Not Found	High	High	High	High	Not Found	Not Found	Not Found
High	High	High	Not Found	Not Found	Not Found	Not Found	Not Found	Not Found	Not Found
Not Found	Not Found	Not Found	High	High	High	High	Not Found	Not Found	Not Found
Not Found	Not Found	Not Found	Not Found	Not Found	Not Found	Not Found	High	High	High
Not Found	Not Found	Not Found	High	High	High	High	Not Found	Not Found	Not Found
Not Found	Not Found	Not Found	High	High	High	High	Not Found	Not Found	Not Found
Not Found	Not Found	Not Found	Not Found	Not Found	Not Found	Not Found	High	High	High
Not Found	Not Found	Not Found	High	High	High	High	Not Found	Not Found	Not Found
Not Found	Not Found	Not Found	High	High	High	High	Not Found	Not Found	Not Found
Not Found	Not Found	Not Found	Not Found	Not Found	Not Found	Not Found	Not Found	Not Found	Not Found
Not Found	Not Found	Not Found	Not Found	Not Found	Not Found	Not Found	High	High	High
High	High	High	Not Found	Not Found	Not Found	Not Found	Not Found	Not Found	Not Found
Not Found	Not Found	Not Found	High	High	High	High	Not Found	Not Found	Not Found
High	High	High	Not Found	Not Found	Not Found	Not Found	Not Found	Not Found	Not Found
Not Found	Not Found	Not Found	Not Found	Not Found	Not Found	Not Found	Not Found	Not Found	Not Found
Not Found	Not Found	Not Found	Not Found	Not Found	Not Found	Not Found	High	High	High
High	High	High	Not Found	Not Found	Not Found	Not Found	Not Found	Not Found	Not Found
Not Found	Not Found	Not Found	Not Found	Not Found	Not Found	Not Found	High	High	High
High	High	High	Not Found	Not Found	Not Found	Not Found	Not Found	Not Found	Not Found
Not Found	Not Found	Not Found	High	High	High	High	Not Found	Not Found	Not Found
Not Found	Not Found	Not Found	Not Found	Not Found	Not Found	Not Found	High	High	High
Not Found	Not Found	Not Found	Not Found	Not Found	Not Found	Not Found	High	High	High
Not Found	Not Found	Not Found	Not Found	Not Found	Not Found	Not Found	High	High	High

Not Found	Not Found	Not Found	Not Found	Not Found	Not Found	Not Found	Not Found	Not Found	Not Found
High	High	High	Not Found	Not Found	Not Found	Not Found	Not Found	Not Found	Not Found
High	High	High	Not Found	Not Found	Not Found	Not Found	Not Found	Not Found	Not Found
Not Found	Not Found	Not Found	High	High	High	High	Not Found	Not Found	Not Found
Not Found	Not Found	Not Found	Not Found	Not Found	Not Found	Not Found	Not Found	Not Found	Not Found
Not Found	Not Found	Not Found	High	High	High	High	Not Found	Not Found	Not Found
Not Found	Not Found	Not Found	High	High	High	High	Not Found	Not Found	Not Found
High	High	High	Not Found	Not Found	Not Found	Not Found	Not Found	Not Found	Not Found
Not Found	Not Found	Not Found	Not Found	Not Found	Not Found	Not Found	High	High	High
Not Found	Not Found	Not Found	High	High	High	High	Not Found	Not Found	Not Found
High	High	High	Not Found	Not Found	Not Found	Not Found	Not Found	Not Found	Not Found
High	High	High	Not Found	Not Found	Not Found	Not Found	Not Found	Not Found	Not Found
Not Found	Not Found	Not Found	Not Found	Not Found	Not Found	Not Found	High	High	High
High	High	High	Not Found	Not Found	Not Found	Not Found	Not Found	Not Found	Not Found
Not Found	Not Found	Not Found	High	High	High	High	Not Found	Not Found	Not Found
High	High	High	Not Found	Not Found	Not Found	Not Found	Not Found	Not Found	Not Found
High	High	High	Not Found	Not Found	Not Found	Not Found	Not Found	Not Found	Not Found
High	High	High	Not Found	Not Found	Not Found	Not Found	Not Found	Not Found	Not Found
Not Found	Not Found	Not Found	Not Found	Not Found	Not Found	Not Found	High	High	High
Not Found	Not Found	Not Found	High	High	High	High	Not Found	Not Found	Not Found
High	High	High	Not Found	Not Found	Not Found	Not Found	Not Found	Not Found	Not Found
Not Found	Not Found	Not Found	High	High	High	High	Not Found	Not Found	Not Found
Not Found	Not Found	Not Found	High	High	High	High	Not Found	Not Found	Not Found
Not Found	Not Found	Not Found	Not Found	Not Found	Not Found	Not Found	High	High	High
High	High	High	Not Found	Not Found	Not Found	Not Found	Not Found	Not Found	Not Found
Not Found	Not Found	Not Found	High	High	High	High	Not Found	Not Found	Not Found
High	High	High	Not Found	Not Found	Not Found	Not Found	Not Found	Not Found	Not Found
Not Found	Not Found	Not Found	High	High	High	High	Not Found	Not Found	Not Found
Not Found	Not Found	Not Found	Not Found	Not Found	Not Found	Not Found	High	High	High
Not Found	Not Found	Not Found	High	High	High	High	Not Found	Not Found	Not Found
Not Found	Not Found	Not Found	Not Found	Not Found	Not Found	Not Found	High	High	High
Not Found	Not Found	Not Found	Not Found	Not Found	Not Found	Not Found	High	High	High
Not Found	Not Found	Not Found	Not Found	Not Found	Not Found	Not Found	High	High	High
High	High	High	Not Found	Not Found	Not Found	Not Found	Not Found	Not Found	Not Found
Not Found	Not Found	Not Found	High	High	High	High	Not Found	Not Found	Not Found
Not Found	Not Found	Not Found	Not Found	Not Found	Not Found	Not Found	High	High	High
Not Found	Not Found	Not Found	Not Found	Not Found	Not Found	Not Found	High	High	High
Not Found	Not Found	Not Found	High	High	High	High	Not Found	Not Found	Not Found
High	High	High	Not Found	Not Found	Not Found	Not Found	Not Found	Not Found	Not Found
Not Found	Not Found	Not Found	High	High	High	High	Not Found	Not Found	Not Found
Not Found	Not Found	Not Found	High	High	High	High	Not Found	Not Found	Not Found
Not Found	Not Found	Not Found	Not Found	Not Found	Not Found	Not Found	Not Found	Not Found	Not Found
Not Found	Not Found	Not Found	Not Found	Not Found	Not Found	Not Found	Not Found	Not Found	Not Found
High	High	High	Not Found	Not Found	Not Found	Not Found	Not Found	Not Found	Not Found
Not Found	Not Found	Not Found	High	High	High	High	Not Found	Not Found	Not Found
Not Found	Not Found	Not Found	High	High	High	High	Not Found	Not Found	Not Found
High	High	High	Not Found	Not Found	Not Found	Not Found	Not Found	Not Found	Not Found
Not Found	Not Found	Not Found	Not Found	Not Found	Not Found	Not Found	High	High	High
High	High	High	Not Found	Not Found	Not Found	Not Found	Not Found	Not Found	Not Found
Not Found	Not Found	Not Found	High	High	High	High	Not Found	Not Found	Not Found

Not Found	Not Found	Not Found	High	High	High	High	Not Found	Not Found	Not Found
High	High	High	Not Found	Not Found	Not Found	Not Found	Not Found	Not Found	Not Found
Not Found	Not Found	Not Found	Not Found	Not Found	Not Found	Not Found	High	High	High
High	High	High	Not Found	Not Found	Not Found	Not Found	Not Found	Not Found	Not Found
Not Found	Not Found	Not Found	High	High	High	High	Not Found	Not Found	Not Found
High	High	High	Not Found	Not Found	Not Found	Not Found	Not Found	Not Found	Not Found
High	High	High	Not Found	Not Found	Not Found	Not Found	Not Found	Not Found	Not Found
Not Found	Not Found	Not Found	High	High	High	High	Not Found	Not Found	Not Found
Not Found	Not Found	Not Found	High	High	High	High	Not Found	Not Found	Not Found
High	High	High	Not Found	Not Found	Not Found	Not Found	Not Found	Not Found	Not Found
High	High	High	Not Found	Not Found	Not Found	Not Found	Not Found	Not Found	Not Found
Not Found	Not Found	Not Found	Not Found	Not Found	Not Found	Not Found	High	High	High
High	High	High	Not Found	Not Found	Not Found	Not Found	Not Found	Not Found	Not Found
High	High	High	Not Found	Not Found	Not Found	Not Found	Not Found	Not Found	Not Found
Not Found	Not Found	Not Found	Not Found	Not Found	Not Found	Not Found	High	High	High
Not Found	Not Found	Not Found	High	High	High	High	Not Found	Not Found	Not Found
High	High	High	Not Found	Not Found	Not Found	Not Found	Not Found	Not Found	Not Found
Not Found	Not Found	Not Found	Not Found	Not Found	Not Found	Not Found	High	High	High
Not Found	Not Found	Not Found	High	High	High	High	Not Found	Not Found	Not Found
High	High	High	Not Found	Not Found	Not Found	Not Found	Not Found	Not Found	Not Found
Not Found	Not Found	Not Found	High	High	High	High	Not Found	Not Found	Not Found
Not Found	Not Found	Not Found	Not Found	Not Found	Not Found	Not Found	High	High	High
High	High	High	Not Found	Not Found	Not Found	Not Found	Not Found	Not Found	Not Found
Not Found	Not Found	Not Found	Not Found	Not Found	Not Found	Not Found	High	High	High
High	High	High	Not Found	Not Found	Not Found	Not Found	Not Found	Not Found	Not Found
Not Found	Not Found	Not Found	High	High	High	High	Not Found	Not Found	Not Found
High	High	High	Not Found	Not Found	Not Found	Not Found	Not Found	Not Found	Not Found
Not Found	Not Found	Not Found	High	High	High	High	Not Found	Not Found	Not Found
Not Found	Not Found	Not Found	Not Found	Not Found	Not Found	Not Found	High	High	High
High	High	High	Not Found	Not Found	Not Found	Not Found	Not Found	Not Found	Not Found
High	High	High	Not Found	Not Found	Not Found	Not Found	Not Found	Not Found	Not Found
Not Found	Not Found	Not Found	Not Found	Not Found	Not Found	Not Found	High	High	High
Not Found	Not Found	Not Found	Not Found	Not Found	Not Found	Not Found	High	High	High
Not Found	Not Found	Not Found	Not Found	Not Found	Not Found	Not Found	High	High	High
Not Found	Not Found	Not Found	High	High	High	High	Not Found	Not Found	Not Found
Not Found	Not Found	Not Found	Not Found	Not Found	Not Found	Not Found	High	High	High
Not Found	Not Found	Not Found	Not Found	Not Found	Not Found	Not Found	High	High	High
Not Found	Not Found	Not Found	Not Found	Not Found	Not Found	Not Found	High	High	High
Not Found	Not Found	Not Found	Not Found	Not Found	Not Found	Not Found	High	High	High
Not Found	Not Found	Not Found	Not Found	Not Found	Not Found	Not Found	Not Found	Not Found	Not Found
Not Found	Not Found	Not Found	Not Found	Not Found	Not Found	Not Found	High	High	High
Not Found	Not Found	Not Found	Not Found	Not Found	Not Found	Not Found	High	High	High
Not Found	Not Found	Not Found	Not Found	Not Found	Not Found	Not Found	High	High	High
Not Found	Not Found	Not Found	High	High	High	High	Not Found	Not Found	Not Found
Not Found	Not Found	Not Found	High	High	High	High	Not Found	Not Found	Not Found
Not Found	Not Found	Not Found	High	High	High	High	Not Found	Not Found	Not Found
Not Found	Not Found	Not Found	Not Found	Not Found	Not Found	Not Found	High	High	High
Not Found	Not Found	Not Found	High	High	High	High	Not Found	Not Found	Not Found
High	High	High	Not Found	Not Found	Not Found	Not Found	Not Found	Not Found	Not Found
High	High	High	Not Found	Not Found	Not Found	Not Found	Not Found	Not Found	Not Found

Not Found	Not Found	Not Found	Not Found	Not Found	Not Found	Not Found	Not Found	Not Found	Not Found
High	High	High	Not Found	Not Found	Not Found	Not Found	Not Found	Not Found	Not Found
High	High	High	Not Found	Not Found	Not Found	Not Found	Not Found	Not Found	Not Found
High	High	High	Not Found	Not Found	Not Found	Not Found	Not Found	Not Found	Not Found
High	High	High	Not Found	Not Found	Not Found	Not Found	Not Found	Not Found	Not Found
Not Found	Not Found	Not Found	Not Found	Not Found	Not Found	Not Found	Not Found	Not Found	Not Found
High	High	High	Not Found	Not Found	Not Found	Not Found	Not Found	Not Found	Not Found
High	High	High	Not Found	Not Found	Not Found	Not Found	Not Found	Not Found	Not Found
Not Found	Not Found	Not Found	Not Found	Not Found	Not Found	Not Found	High	High	High
Not Found	Not Found	Not Found	High	High	High	High	Not Found	Not Found	Not Found
Not Found	Not Found	Not Found	High	High	High	High	Not Found	Not Found	Not Found
Not Found	Not Found	Not Found	Not Found	Not Found	Not Found	Not Found	Not Found	Not Found	Not Found
Not Found	Not Found	Not Found	Not Found	Not Found	Not Found	Not Found	High	High	High
Not Found	Not Found	Not Found	Not Found	Not Found	Not Found	Not Found	High	High	High
Not Found	Not Found	Not Found	Not Found	Not Found	Not Found	Not Found	High	High	High
Not Found	Not Found	Not Found	High	High	High	High	Not Found	Not Found	Not Found
Not Found	Not Found	Not Found	Not Found	Not Found	Not Found	Not Found	High	High	High
High	High	High	Not Found	Not Found	Not Found	Not Found	Not Found	Not Found	Not Found
Not Found	Not Found	Not Found	High	High	High	High	Not Found	Not Found	Not Found
Not Found	Not Found	Not Found	Not Found	Not Found	Not Found	Not Found	Not Found	Not Found	Not Found
Not Found	Not Found	Not Found	Not Found	Not Found	Not Found	Not Found	High	High	High
High	High	High	Not Found	Not Found	Not Found	Not Found	Not Found	Not Found	Not Found
Not Found	Not Found	Not Found	Not Found	Not Found	Not Found	Not Found	High	High	High
Not Found	Not Found	Not Found	Not Found	Not Found	Not Found	Not Found	High	High	High
Not Found	Not Found	Not Found	Not Found	Not Found	Not Found	Not Found	High	High	High
Not Found	Not Found	Not Found	Not Found	Not Found	Not Found	Not Found	High	High	High
Not Found	Not Found	Not Found	Not Found	Not Found	Not Found	Not Found	High	High	High
Not Found	Not Found	Not Found	Not Found	Not Found	Not Found	Not Found	High	High	High
High	High	High	Not Found	Not Found	Not Found	Not Found	Not Found	Not Found	Not Found
High	High	High	Not Found	Not Found	Not Found	Not Found	Not Found	Not Found	Not Found
High	High	High	Not Found	Not Found	Not Found	Not Found	Not Found	Not Found	Not Found
Not Found	Not Found	Not Found	Not Found	Not Found	Not Found	Not Found	High	High	High
Not Found	Not Found	Not Found	High	High	High	High	Not Found	Not Found	Not Found
Not Found	Not Found	Not Found	High	High	High	High	Not Found	Not Found	Not Found
Not Found	Not Found	Not Found	Not Found	Not Found	Not Found	Not Found	High	High	High
Not Found	Not Found	Not Found	Not Found	Not Found	Not Found	Not Found	High	High	High
High	High	High	Not Found	Not Found	Not Found	Not Found	Not Found	Not Found	Not Found
High	High	High	Not Found	Not Found	Not Found	Not Found	Not Found	Not Found	Not Found
Not Found	Not Found	Not Found	Not Found	Not Found	Not Found	Not Found	High	High	High
Not Found	Not Found	Not Found	High	High	High	High	Not Found	Not Found	Not Found
High	High	High	Not Found	Not Found	Not Found	Not Found	Not Found	Not Found	Not Found
High	High	High	Not Found	Not Found	Not Found	Not Found	Not Found	Not Found	Not Found
Not Found	Not Found	Not Found	High	High	High	High	Not Found	Not Found	Not Found
Not Found	Not Found	Not Found	High	High	High	High	Not Found	Not Found	Not Found
Not Found	Not Found	Not Found	High	High	High	High	Not Found	Not Found	Not Found
Not Found	Not Found	Not Found	High	High	High	High	Not Found	Not Found	Not Found
Not Found	Not Found	Not Found	Not Found	Not Found	Not Found	Not Found	Not Found	Not Found	Not Found
Not Found	Not Found	Not Found	Not Found	Not Found	Not Found	Not Found	High	High	High
Not Found	Not Found	Not Found	Not Found	Not Found	Not Found	Not Found	High	High	High
High	High	High	Not Found	Not Found	Not Found	Not Found	Not Found	Not Found	Not Found

Not Found	Not Found	Not Found	Not Found	Not Found	Not Found	Not Found	High	High	High
Not Found	Not Found	Not Found	High	High	High	High	Not Found	Not Found	Not Found
High	High	High	Not Found	Not Found	Not Found	Not Found	Not Found	Not Found	Not Found
High	High	High	Not Found	Not Found	Not Found	Not Found	Not Found	Not Found	Not Found
Not Found	Not Found	Not Found	High	High	High	High	Not Found	Not Found	Not Found
High	High	High	Not Found	Not Found	Not Found	Not Found	Not Found	Not Found	Not Found
Not Found	Not Found	Not Found	Not Found	Not Found	Not Found	Not Found	High	High	High
Not Found	Not Found	Not Found	Not Found	Not Found	Not Found	Not Found	High	High	High
High	High	High	Not Found	Not Found	Not Found	Not Found	Not Found	Not Found	Not Found
Not Found	Not Found	Not Found	High	High	High	High	Not Found	Not Found	Not Found
Not Found	Not Found	Not Found	High	High	High	High	Not Found	Not Found	Not Found
Not Found	Not Found	Not Found	Not Found	Not Found	Not Found	Not Found	High	High	High
High	High	High	Not Found	Not Found	Not Found	Not Found	Not Found	Not Found	Not Found
Not Found	Not Found	Not Found	Not Found	Not Found	Not Found	Not Found	High	High	High
High	High	High	Not Found	Not Found	Not Found	Not Found	Not Found	Not Found	Not Found
High	High	High	Not Found	Not Found	Not Found	Not Found	Not Found	Not Found	Not Found
Not Found	Not Found	Not Found	Not Found	Not Found	Not Found	Not Found	High	High	High
Not Found	Not Found	Not Found	High	High	High	High	Not Found	Not Found	Not Found
High	High	High	Not Found	Not Found	Not Found	Not Found	Not Found	Not Found	Not Found
High	High	High	Not Found	Not Found	Not Found	Not Found	Not Found	Not Found	Not Found
High	High	High	Not Found	Not Found	Not Found	Not Found	Not Found	Not Found	Not Found
Not Found	Not Found	Not Found	High	High	High	High	Not Found	Not Found	Not Found
Not Found	Not Found	Not Found	High	High	High	High	Not Found	Not Found	Not Found
Not Found	Not Found	Not Found	High	High	High	High	Not Found	Not Found	Not Found
Not Found	Not Found	Not Found	High	High	High	High	Not Found	Not Found	Not Found
High	High	High	Not Found	Not Found	Not Found	Not Found	Not Found	Not Found	Not Found
Not Found	Not Found	Not Found	High	High	High	High	Not Found	Not Found	Not Found
High	High	High	Not Found	Not Found	Not Found	Not Found	Not Found	Not Found	Not Found
Not Found	Not Found	Not Found	High	High	High	High	Not Found	Not Found	Not Found
Not Found	Not Found	Not Found	High	High	High	High	Not Found	Not Found	Not Found
Not Found	Not Found	Not Found	Not Found	Not Found	Not Found	Not Found	High	High	High
Not Found	Not Found	Not Found	Not Found	Not Found	Not Found	Not Found	Not Found	Not Found	Not Found
High	High	High	Not Found	Not Found	Not Found	Not Found	Not Found	Not Found	Not Found
Not Found	Not Found	Not Found	Not Found	Not Found	Not Found	Not Found	High	High	High
High	High	High	Not Found	Not Found	Not Found	Not Found	Not Found	Not Found	Not Found
Not Found	Not Found	Not Found	Not Found	Not Found	Not Found	Not Found	Not Found	Not Found	Not Found
Not Found	Not Found	Not Found	High	High	High	High	Not Found	Not Found	Not Found
High	High	High	Not Found	Not Found	Not Found	Not Found	Not Found	Not Found	Not Found
Not Found	Not Found	Not Found	High	High	High	High	Not Found	Not Found	Not Found
Not Found	Not Found	Not Found	Not Found	Not Found	Not Found	Not Found	High	High	High
Not Found	Not Found	Not Found	High	High	High	High	Not Found	Not Found	Not Found
Not Found	Not Found	Not Found	High	High	High	High	Not Found	Not Found	Not Found
Not Found	Not Found	Not Found	High	High	High	High	Not Found	Not Found	Not Found
Not Found	Not Found	Not Found	High	High	High	High	Not Found	Not Found	Not Found
Not Found	Not Found	Not Found	High	High	High	High	Not Found	Not Found	Not Found
High	High	High	Not Found	Not Found	Not Found	Not Found	Not Found	Not Found	Not Found
High	High	High	Not Found	Not Found	Not Found	Not Found	Not Found	Not Found	Not Found
Not Found	Not Found	Not Found	Not Found	Not Found	Not Found	Not Found	High	High	High
Not Found	Not Found	Not Found	Not Found	Not Found	Not Found	Not Found	High	High	High
Not Found	Not Found	Not Found	Not Found	Not Found	Not Found	Not Found	High	High	High

Not Found	Not Found	Not Found	High	High	High	High	Not Found	Not Found	Not Found
Not Found	Not Found	Not Found	High	High	High	High	Not Found	Not Found	Not Found
High	High	High	Not Found	Not Found	Not Found	Not Found	Not Found	Not Found	Not Found
Not Found	Not Found	Not Found	High	High	High	High	Not Found	Not Found	Not Found
High	High	High	Not Found	Not Found	Not Found	Not Found	Not Found	Not Found	Not Found
Not Found	Not Found	Not Found	High	High	High	High	Not Found	Not Found	Not Found
High	High	High	Not Found	Not Found	Not Found	Not Found	Not Found	Not Found	Not Found
Not Found	Not Found	Not Found	Not Found	Not Found	Not Found	Not Found	High	High	High
Not Found	Not Found	Not Found	Not Found	Not Found	Not Found	Not Found	High	High	High
High	High	High	Not Found	Not Found	Not Found	Not Found	Not Found	Not Found	Not Found
Not Found	Not Found	Not Found	Not Found	Not Found	Not Found	Not Found	High	High	High
High	High	High	Not Found	Not Found	Not Found	Not Found	Not Found	Not Found	Not Found
Not Found	Not Found	Not Found	Not Found	Not Found	Not Found	Not Found	High	High	High
Not Found	Not Found	Not Found	High	High	High	High	Not Found	Not Found	Not Found
High	High	High	Not Found	Not Found	Not Found	Not Found	Not Found	Not Found	Not Found
Not Found	Not Found	Not Found	Not Found	Not Found	Not Found	Not Found	High	High	High
Not Found	Not Found	Not Found	High	High	High	High	Not Found	Not Found	Not Found

Found in Sample: [S12] F3: 117, Sample
High
High
High
High
High
High
High
High
High
High
High
High
High
High
High
High
High
High
High
High
High
Not Found
High
High
High
High
High
High
Not Found
High
High
High
High
High
High
High
High
High
High
High
High
High
High
High
High
High
High
High
High

High
High
High
High
High
High
High
High
High
High
High
High
High
High
High
Not Found
High
High
High
High
High
High
High
High
High
High
High
High
High
High
High
High
High
High
High
High
High
High
High
High
High
High
High
High
High
High
High
High
High
High

High
High
High
High
High
High
High
High
High
High
High
High
High
High
High
High
High
Not Found
High
High
High
High
High
High
High
High
High
High
Not Found
High
High
High
High
High
High
High
High
High
High
High
High
Not Found
High
High
High
High
High
High
High
High

High
High
High
High
High
Not Found
High
High
High
High
High
High
High
High
Not Found
High
High
High
High
High
High
High
High
High
High
High
High
High
High
High
High
High
High
High
High
High
High
High
High
High
High
High
High
Not Found
High
High
High
High
High
High

High
High
High
High
High
High
High
High
High
High
High
High
High
High
High
High
Not Found
High
High
High
High
High
High
High
High
High
High
High
High
High
High
High
High
High
High
High
High
High
Not Found
High
Not Found
High
High
High
High
High
High
High
High
High

High
High
High
High
High
High
High
High
High
High
High
High
High
High
High
High
High
High
High
Not Found
High
High
Not Found
High
High
High
High
High
High
High
High
High
High
High
High
High
High
High
High
High
High
High
High
High
High
High
High
High
High
High

High
High
High
High
High
High
High
High
High
High
High
High
High
High
High
High
High
High
High
High
High
High
High
High
High
High
High
High
High
High
High
High
High
High
High
High
High
High
High
High
High
High
High
High
High
High
High
High
High
High

High
High
High
High
High
High
High
High
High
High
High
High
High
High
High
High
High
High
High
High
High
High
High
High
High
High
High
High
High
High
High
High
High
High
High
High
High
High
High
High
High
High
High
High
High
High
Not Found
High
High
High

High
High
High
High
High
High
High
Not Found
Not Found
High
High
High
High
High
High
High
High
High
High
High
High
High
High
High
High
High
High
High
High
Not Found
High
Not Found
High
High
High
High
High
High
High
High
High
High
High
High
High
High
High
High
High
High

High
High
High
High
High
High
High
High
High
High
High
High
High
High
High
High
High
High
High
High
High
High
High
High
High
High
High
Not Found
High
High
High
High
High
High
High
High
High
High
High
High
High
High
High
High
High
High
High
High
High
High

High
High
High
High
High
High
High
High
High
High
High
High
High
High
High
High
High
High
High
High
High
High
High
High
High
High
Not Found
High
High
High
High
High
High
High
High
High
High
High
High
High
Not Found
High
High
High
High
High
High
High
High
High

High
High
High
Not Found
High
High
High
High
High
High
High
High
High
High
High
High
High
High
High
High
High
High
Not Found
High
High
High
High
High
High
High
High
High
High
High
High
High
High
High
High
High
High
High
High
High
High
High
High
High
High
High

High
High
High
High
High
High
High
High
High
High
High
High
High
High
High
High
High
High
High
High
High
High
High
High
High
High
High
High
High
High
High
High
High
High
High
High
High
High
High
High
High
High
Not Found
High
High
High
High
High
High
High

High
High
High
High
High
High
High
High
High
High
High
High
High
High
High
High
Not Found
High
High
High
High
High
High
High
High
High
High
High
High
High
High
High
High
High
High
High
High
High
High
High
High
High
High
High
High
High
High
High
High
High

High
High
High
High
High
High
High
High
High
High
High
High
High
High
High
High
High
High
High
High
High
High
High
High
High
High
High
High
High
High
High
High
High
High
High
High
High
High
High
High
High
High
High
High
High
High
Not Found
High
High
High

High
High
High
High
High
High
High
High
High
High
High
High
High
High
High
High
High
High
High
High
High
High
High
High
High
High
High
High
Not Found
High
High
Not Found
High
High
High
High
High
High
High
High
High
High
High
High
High
High
High
Not Found
High
High

High
High
High
High
High
High
High
High
High
High
High
High
High
High
High
High
High
High
High
High
High
High
High
High
High
High
High
High
High
High
High
High
High
High
High
High
High
High
High
High
High
High
High
High
High
High
High
High
High
High

High
High
High
High
High
High
High
High
High
High
High
High
High
High
High
High
Not Found
High
High
High
High
High
High
High
High
High
High
High
High
High
High
High
High
High
High
High
Not Found
High
High
High
High
High
High
High
High
High
High
High
High
High

High
High
High
High
High
High
High
High
High
High
High
High
High
High
High
High
High
High
High
High
High
High
High
High
Not Found
High
High
High
High
High
High
High
High
High
High
High
Not Found
High
High
High
High
Not Found
High
High
High
High
High
High
High
High

High
High
High
High
High
High
High
High
High
High
High
High
High
High
High
High
High
High
High
High
High
High
High
High
High
High
High
High
High
High
High
High
High
High
High
High
High
High
High
High
High
High
High
High
High
High
High
High
High
High

High
High
High
High
High
High
High
High
High
High
High
High
High
High
High
High
High
High
High
High
High
High
High
High
High
High
High
High
High
High
High
High
High
High
High
Not Found
High
High
High
High
High
High
High
High
High
High
High
High
High
High

High
High
High
High
High
High
High
High
High
High
High
High
High
High
High
High
High
High
High
High
High
High
High
High
High
High
High
High
High
High
High
High
High
High
High
High
High
High
High
High
High
High
High
High
High
High
High
High
High
High

High
High
High
High
High
High
High
High
High
High
High
High
High
High
High
High
High
High
High
High
High
High
High
High
High
High
High
High
High
High
High
High
High
High
High
High
High
High
High
High
High
High
High
High
High
High
High
High
High
High

High
High
High
High
High
High
High
High
High
High
High
High
High
High
High
High
High
High
High
High
High
High
High
High
High
High
High
High
High
High
High
High
High
High
High
High
High
High
High
High
High
High
High
High
High
High
High
High
High
High

High
High
High
High
High
High
High
High
High
High
High
High
High
High
High
High
High
High
High
High
High
High
High
High
High
High
High
High
High
High
High
High
High
High
High
High
High
High
High
High
High
High
High
High
High
High
High
High
High
High

High
High
High
High
High
High
High
High
High
High
High
High
High
High
High
High
High
High
High
High
High
High
High
High
High
High
High
High
High
High
High
High
High
High
High
High
High
High
High
High
High
High
High
High
High
High
High
High
High
High

High
High
High
High
High
High
High
High
High
High
High
High
High
High
High
High
High
High
High
High
High
High
High
High
High
High
High
High
High
High
High
High
High
High
High
High
High
High
High
High
High
High
High
High
High
High
High
High
High
High

High
High
High
High
High
High
High
High
High
High
High
High
High
High
High
High
High
High
High
High
High
High
High
High
High
High
High
High
High
High
High
High
High
High
High
High
High
High
High
High
High
High
High
High
High
High
High
High
High
High

High
High
High
High
High
High
High
High
High
High
High
High
High
High
High
High
High
High
High
High
High
High
High
High
High
High
High
High
High
High
High
High
High
High
High
High
High
High
High
High
High
High
High
High
High
High
High
High
High
High

High
High
High
High
High
Not Found
High
High
High
High
High
High
High
High
High
High
High
High
High
High
High
High
High
High
High
High
High
High
High
High
High
High
High
High
High
High
High
High
High
High
High
High
High
High
High
High
High
High
Not Found
High

High
High
High
High
High
High
High
High
High
High
High
High
High
High
High
High
High
High
High
High
High
High
High
High
High
High
High
High
High
High
High
High
High
High
High
High
High
High
High
High
High
High
High
High
High
High
High
Not Found
High
High

High
High
High
High
High
High
Not Found
High
High
Not Found
High
High
High
High
High
High
High
High
High
High
High
High
High
High
High
High
High
High
High
High
High
High
High
High
High
High
High
High
High
High
High
High
High
High
High
Not Found
High
High
High
High

Not Found
High
High
High
High
High
High
High
High
High
High
High
High
High
High
High
High
High
High
High
High
High
High
High
High
High
High
High
High
High
High
High
High
High
High
High
High
High
High
High
High
High
High
High
High
High
High
High
High
High

High
High
High
High
High
High
High
High
High
High
High
High
High
High
High
High
High
High
High
High
High
High
High
High
High
High
High
High
High
High
High
High
High
High
High
High
High
High
High
High
High
High
High
High
High
High
High
High
High
High

High
High
High
High
High
High
High
High
High
High
High
High
High
High
High
High
High
High
High
High
High
High
High
High
High
High
High
High
High
High
High
High
High
High
High
High
High
High
High
High
High
High
High
High
High
High
High
High
High
High

High
High
High
High
High
High
High
High
High
High
High
High
High
Not Found
High
High
High
High
High
High
High
High
High
High
High
Not Found
High
High
High
High
High
High
High
High
High
High
High
High
High
High
High
High
High
High
High
High
High
High
High
High

High
High
High
High
High
High
High
High
High
High
High
High
High
High
Not Found
High
High
High
High
High
Not Found
High
High
High
High
High
High
High
High
High
High
High
High
High
High
High
High
High
High
High
High
High
High
High
High
High
High
High
High
High

High
High
High
High
High
High
High
High
High
High
High
High
High
High
High
High
High
High
High
High
High
High
High
High
High
High
High
High
High
High
High
High
High
High
High
High
High
High
High
High
High
High
High
High
High
High
High
Not Found
High
High

High
High
High
High
High
High
High
High
High
High
High
Not Found
High
High
High
High
High
High
High
High
High
High
High
High
High
High
High
High
High
High
High
High
Not Found
High
High
High
High
High
High
High
High
High
High
High
High
High
High
High
High
High

High
High
High
High
High
High
Not Found
High
High
High
High
High
Not Found
High
High
High
High
High
High
High
High
Not Found
High
High
High
High
High
Not Found
High
High
High
High
High
High
Not Found
High
High
Not Found
High
High
High
High
High
High
High
Not Found
High
High
High
High

Not Found
High
High
High
High
Not Found
High
High
High
Not Found
High
High
High
High
High
High
High
High
Not Found
High
High
High
High
High
High
Not Found
High
High
High
High
High
High
High
High
High
High
Not Found
High
High
High
High
High
Not Found
High
Not Found
High
High
High
High
High

High
High
High
High
High
Not Found
High
High
High
Not Found
High
High
Not Found
High
High
High
Not Found
High
High
High
High
High
High
High
High
High
High
High
High
High
High
High
High
High
High
High
High
Not Found
High
High
High
Not Found
High
High
High
High
High
High
High
High

Not Found
High
High
High
High
High
High
High
High
High
High
High
High
High
High
High
High
High
High
High
High
High
Not Found
High
Not Found
High
Not Found
High
High
High
High
High
High
High
High
Not Found
Not Found
High
High
High
Not Found
Not Found
High
High
High
High
High
High
High
High

High
High
High
High
High
High
High
Not Found
Not Found
High
High
High
High
High
Not Found
High
High
Not Found
High
High
Not Found
High
High
Not Found
High
High
High
High
Not Found
High
High
High
High
Not Found
High
High
High
High
High
Not Found
High
High
High
High
High
High
High
High
High
High

High
High
High
High
High
High
High
High
High
High
High
Not Found
High
Not Found
Not Found
High
High
High
High
High
Not Found
High
High
High
High
High
High
High
High
High
High
High
High
High
High
High
High
High
High
High
High
High
High
High
High
High
High
High
High
High

High
High
High
High
High
High
Not Found
High
High
High
High
High
Not Found
High
High
High
High
High
High
High
Not Found
High
High
High
Not Found
High
High
Not Found
High
Not Found
High
High
High
High
High
High
High
High
High
High
High
High
Not Found
High
High
High
High
High
High
Not Found

High
High
High
High
High
High
High
Not Found
High
High
High
High
High
Not Found
High
High
High
High
High
High
High
High
High
High
High
Not Found
High
High
Not Found
High
High
High
Not Found
High
High
High
Not Found
Not Found
High
High
High
High
High
High
High
High
Not Found
High
High
High

Not Found
High
High
Not Found
High
High
Not Found
High
High
Not Found
Not Found
High
Not Found
High
High
High
High
High
High
High
Not Found
High
High
High
High
High
Not Found
High
High
High
Not Found
High
High
Not Found
High
High
High
High
High
Not Found
High
High
High
High
High
High
High
High
High
High

Not Found
High
High
High
High
High
High
Not Found
High
High
High
Not Found
High
High
Not Found
High
Not Found
High
High
High
Not Found
High
High
Not Found
High
High
High
High
Not Found
High
Not Found
High
High
High
High
High
High
High
Not Found
High
High
High
Not Found
Not Found
High
High
High
High
High
High

Not Found
High
High
High
High
High
High
Not Found
High
Not Found
High
High
High
High
High
High
High
Not Found
High
High
Not Found
High
High
Not Found
High
High
High
High
High
High
High
High
High
Not Found
High
High
High
High
High
High
High
High
High
High
High
High
Not Found
High
Not Found
Not Found

High
High
High
High
High
High
High
High
High
High
High
High
Not Found
High
High
High
High
High
High
Not Found
High
High
Not Found
High
Not Found
High
Not Found
High
Not Found
High
High
High
High
High
High
High
High
High
High
High
High
High
High
High
Not Found
High
Not Found
High
High
Not Found

High
High
High
High
High
Not Found
High
High
High
High
High
High
High
High
High
Not Found
High
Not Found
High
High
High
High
High
High
Not Found
Not Found
High
High
Not Found
High
High
High
Not Found
Not Found
High
High
High
High
High
High
High
High
Not Found
High
Not Found
High
High
High
High
Not Found

High
Not Found
Not Found
High
High
High
High
High
High
High
Not Found
Not Found
High
Not Found
Not Found
High
High
High
Not Found
High
Not Found
High
Not Found
High
Not Found
High
Not Found
High
High
Not Found
High
Not Found
Not Found
Not Found
Not Found
High
Not Found
High
Not Found
Not Found
Not Found
Not Found
High
Not Found
Not Found
Not Found
High
High
High
High

High
High
Not Found
Not Found
Not Found
Not Found
High
Not Found
Not Found
Not Found
Not Found
High
Not Found
High
Not Found
High
Not Found
High
High
High
High
Not Found
High
High
High
Not Found
High
High
Not Found
High
High
High
High
Not Found
Not Found
High
High
High
High
High
Not Found
High
High
Not Found
High
High
High
High
High
Not Found

Not Found
High
High
High
High
High
High
High
Not Found
High
High
High
High
Not Found
High
Not Found
Not Found
High
Not Found
Not Found
Not Found
High
High
High
High
High
High
Not Found
Not Found
High
High
High
High
High
High
High
High
High
Not Found
Not Found
High
Not Found
High
Not Found
Not Found
High
High
High
High
Not Found

High
High
Not Found
High
Not Found
Not Found
Not Found
High
High
High
Not Found
High
High
High
High
High
Not Found
Not Found
High
Not Found
High
High
High
High
Not Found
High
High
High
High
High
High
High
Not Found
High
High
High
High
High
High
Not Found
High
Not Found
Not Found
High
High
High
High
High
Not Found
High

Not Found
High
High
High
Not Found
High
High
Not Found
High
High
High
High
High
Not Found
High
High
High
Not Found
High
Not Found
High
High
High
Not Found
High
Not Found
High
Not Found
Not Found
High
High
Not Found
High
High
High
Not Found
Not Found
High
High
High
Not Found
Not Found
High
Not Found
High
High
High
High
Not Found
High

High
Not Found
Not Found
High
High
Not Found
High
High
High
High
Not Found
High
Not Found
High
High
High
High
Not Found
High
High
High
High
Not Found
High
Not Found
Not Found
High
High
High
Not Found
High
High
High
High
High
Not Found
High
Not Found
Not Found
Not Found
High
Not Found
Not Found
Not Found
High
Not Found
High
Not Found
Not Found
Not Found

Not Found
High
High
Not Found
Not Found
Not Found
Not Found
High
High
High
Not Found
High
Not Found
Not Found
High
High
High
High
High
Not Found
High
High
Not Found
High
Not Found
High
High
High
High
High
Not Found
High
Not Found
High
High
Not Found
Not Found
High
High
High
High
Not Found
Not Found
High
High
High
High
High
Not Found
Not Found

Not Found
High
Not Found
High
High
High
High
Not Found
High
High
High
High
High
Not Found
High
Not Found
High
High
High
Not Found
Not Found
High
High
High
High
High
High
Not Found
Not Found
Not Found
High
Not Found
Not Found
Not Found
High
Not Found
Not Found
Not Found
High
Not Found
Not Found
High
High
High
Not Found
Not Found
High
Not Found
Not Found
Not Found

Not Found
High
High
Not Found
High
Not Found
Not Found
High
High
Not Found
Not Found
Not Found
High
Not Found
Not Found
Not Found
Not Found
Not Found
Not Found
Not Found
High
High
High
Not Found
Not Found
Not Found
High
High
Not Found
Not Found
Not Found
Not Found
Not Found
Not Found
Not Found
High
High
High
Not Found
Not Found
High
High
Not Found
High
Not Found
High
High
Not Found
High
High

Not Found
Not Found
Not Found
Not Found
Not Found
Not Found
Not Found
High
Not Found
High
High
Not Found
High
Not Found
Not Found
High
Not Found
High
High
High
Not Found
Not Found
Not Found
High
High
Not Found
Not Found
High
High
Not Found
High
Not Found
Not Found
High
Not Found
High
Not Found
Not Found
Not Found
Not Found
High
Not Found
Not Found
High
Not Found
Not Found
Not Found
Not Found
Not Found
Not Found

Not Found
High
Not Found
Not Found
Not Found
High
Not Found
Not Found
Not Found
Not Found
Not Found
High
Not Found
High
Not Found
Not Found
Not Found
High
High
High
Not Found
High
Not Found
High
High
High
Not Found
Not Found
Not Found
Not Found
High
High
High
High
High
Not Found
High
Not Found
High
Not Found
Not Found
Not Found
Not Found
High
Not Found
High
Not Found
Not Found
High
Not Found

High
Not Found
Not Found
Not Found
Not Found
Not Found
High
Not Found
Not Found
High
High
High
Not Found
High
Not Found
High
Not Found
High
High
Not Found
Not Found
Not Found
Not Found
High
High
High
Not Found
Not Found
High
High
Not Found
High
High
Not Found
High
High
Not Found
Not Found
High
Not Found
Not Found
High
High
Not Found
High
High
Not Found
High
Not Found
Not Found

High
Not Found
Not Found
Not Found
High
High
Not Found
High
Not Found
High
Not Found
Not Found
Not Found
Not Found
High
High
High
Not Found
Not Found
Not Found
Not Found
High
Not Found
Not Found
Not Found
High
Not Found
Not Found
Not Found
Not Found
High
Not Found
High
High
High
Not Found
Not Found
Not Found
Not Found
Not Found
Not Found
High
High
Not Found
Not Found
Not Found
High
Not Found
Not Found
Not Found

Not Found
Not Found
Not Found
Not Found
High
High
Not Found
High
Not Found
High
High
High
High
Not Found
Not Found
Not Found
Not Found
Not Found
High
Not Found
Not Found
High
Not Found
High
Not Found
Not Found
Not Found
High
Not Found
High
High
Not Found
High
High
Not Found
Not Found
Not Found
High
High
High
High
High
High
Not Found
High
Not Found
High
Not Found
Not Found
Not Found

High
Not Found
High
Not Found
High
Not Found
Not Found
High
High
Not Found
Not Found
High
Not Found
Not Found
High
Not Found
Not Found
High
Not Found
Not Found
Not Found
Not Found
Not Found
High
Not Found
High
High
Not Found
High
Not Found
High
Not Found
Not Found
High
Not Found
High
Not Found
Not Found
High
Not Found
Not Found
Not Found
Not Found
High
High
Not Found
Not Found
Not Found
Not Found
Not Found

High
Not Found
High
Not Found
High
High
Not Found
Not Found
Not Found
High
Not Found
Not Found
Not Found
Not Found
Not Found
High
Not Found
High
Not Found
Not Found
High
High
Not Found
Not Found
Not Found
Not Found
High
Not Found
Not Found
Not Found
Not Found
Not Found
High
Not Found
High
Not Found
Not Found
High
Not Found
Not Found
Not Found
High
Not Found
Not Found
High
Not Found
Not Found
Not Found
High
High

Not Found
Not Found
Not Found
Not Found
High
High
High
High
High
Not Found
High
Not Found
Not Found
Not Found
Not Found
Not Found
Not Found
Not Found
Not Found
Not Found
Not Found
Not Found
Not Found
High
High
Not Found
Not Found
High
Not Found
Not Found
Not Found
Not Found
Not Found
Not Found
High
Not Found
Not Found
Not Found
Not Found
High
High
Not Found
Not Found
Not Found
High
High
Not Found
Not Found
Not Found
High

Not Found
Not Found
Not Found
Not Found
High
Not Found
Not Found
Not Found
High
High
High
Not Found
Not Found
High
Not Found
High
Not Found
High
Not Found
Not Found
Not Found
Not Found
Not Found
Not Found
Not Found
Not Found
Not Found
High
Not Found
High
Not Found
Not Found
Not Found
Not Found
Not Found
Not Found
Not Found
Not Found
High
Not Found
High
Not Found
Not Found
Not Found
High
Not Found
Not Found
Not Found
Not Found
High

High
Not Found
Not Found
High
Not Found
Not Found
Not Found
Not Found
High
High
Not Found
Not Found
Not Found
High
Not Found
Not Found
High
High
Not Found
High
High
Not Found
Not Found
Not Found
Not Found
High
High
Not Found
High
Not Found
Not Found
Not Found
Not Found
Not Found
Not Found
High
High
High
Not Found
Not Found
Not Found
Not Found
Not Found
Not Found
Not Found
Not Found
Not Found
Not Found
Not Found
High

Not Found
High
Not Found
Not Found
Not Found
High
Not Found
Not Found
High
Not Found
High
Not Found
High
Not Found
Not Found
High
High
Not Found
Not Found
Not Found
Not Found
Not Found
Not Found
Not Found
Not Found
High
Not Found
Not Found
Not Found
Not Found
Not Found
Not Found
Not Found
High
High
Not Found
Not Found
High
Not Found
Not Found
Not Found
Not Found
Not Found
Not Found
Not Found
High
Not Found
Not Found
Not Found
High

High
Not Found
High
High
Not Found
Not Found
Not Found
Not Found
Not Found
High
Not Found
Not Found
Not Found
Not Found
Not Found
High
Not Found
Not Found
High
High
High
Not Found
High
Not Found
Not Found
Not Found
Not Found
Not Found
Not Found
Not Found
Not Found
Not Found
Not Found
Not Found
Not Found
Not Found
Not Found
Not Found
High
Not Found
Not Found
Not Found
High
Not Found
Not Found
Not Found
Not Found
Not Found
Not Found
High

Not Found
Not Found
High
High
High
Not Found
Not Found
Not Found
Not Found
Not Found
Not Found
Not Found
High
Not Found
Not Found
Not Found
Not Found
Not Found
Not Found
Not Found
Not Found
Not Found
Not Found
Not Found
High
Not Found
Not Found
High
Not Found
Not Found
High
High
Not Found
Not Found
Not Found
Not Found
Not Found
High
High
Not Found
Not Found
Not Found
Not Found
Not Found
High
Not Found
High
Not Found
Not Found
Not Found

Not Found
Not Found
Not Found
Not Found
Not Found
Not Found
Not Found
Not Found
Not Found
Not Found
Not Found
Not Found
High
High
Not Found
Not Found
Not Found
Not Found
Not Found
Not Found
Not Found
Not Found
Not Found
Not Found
Not Found
Not Found
Not Found
Not Found
Not Found
Not Found
Not Found
Not Found
Not Found
High
Not Found
Not Found
Not Found
Not Found
Not Found
Not Found
Not Found
High
Not Found
High
High
Not Found
Not Found
High
High
High

Not Found
Not Found
High
High
Not Found
Not Found
Not Found
High
Not Found
Not Found
Not Found
Not Found
Not Found
Not Found
Not Found
High
Not Found
Not Found
Not Found
High
Not Found
Not Found
Not Found
High
Not Found
Not Found
High
Not Found
High
High
Not Found
Not Found
Not Found
Not Found
Not Found
Not Found
Not Found
Not Found
High
Not Found
High
Not Found
Not Found
High
Not Found
Not Found
Not Found
Not Found
Not Found
Not Found

Not Found
Not Found
Not Found
High
Not Found
Not Found
Not Found
Not Found
High
Not Found
Not Found
Not Found
Not Found
High
Not Found
Not Found
Not Found
High
Not Found
Not Found
Not Found
Not Found
High
Not Found
Not Found
Not Found
High
Not Found
High
Not Found
Not Found
Not Found
Not Found
Not Found
Not Found
High
High
High
Not Found
Not Found
High
Not Found
Not Found
Not Found
High
Not Found
Not Found
Not Found
High
High

Not Found
High
Not Found
High
High
High
High
Not Found
Not Found
Not Found
Not Found
Not Found
Not Found
Not Found
Not Found
Not Found
Not Found
Not Found
Not Found
Not Found
High
Not Found
High
High
Not Found
Not Found
High
Not Found
Not Found
Not Found
High
Not Found
Not Found
High
Not Found
Not Found
Not Found
High
Not Found
Not Found
Not Found
Not Found
High
Not Found
High
Not Found
Not Found
High
High
High

Not Found
Not Found
Not Found
Not Found
Not Found
Not Found
Not Found
Not Found
High
Not Found
Not Found
Not Found
High
Not Found
Not Found
Not Found
Not Found
Not Found
High
Not Found
Not Found
Not Found
Not Found
High
Not Found
Not Found
Not Found
Not Found
High
Not Found
High
High
High
Not Found
Not Found
High
High
Not Found
Not Found
Not Found
Not Found
Not Found
Not Found
Not Found
Not Found
Not Found
Not Found
High
Not Found
Not Found

Not Found
Not Found
High
Not Found
Not Found
Not Found
Not Found
Not Found
Not Found
Not Found
Not Found
High
Not Found
Not Found
High
Not Found
Not Found
High
Not Found
Not Found
Not Found
High
Not Found
High
Not Found
Not Found
Not Found
Not Found
High
Not Found
Not Found
High
High
High
Not Found
High
High
High
High
Not Found
High
High
High
Not Found
Not Found
Not Found
High
Not Found
Not Found
Not Found

Not Found
Not Found
Not Found
Not Found
Not Found
Not Found
Not Found
Not Found
High
Not Found
Not Found
Not Found
High
High
High
Not Found
High
Not Found
Not Found
Not Found
High
Not Found
High
High
High
High
High
High
Not Found
Not Found
Not Found
High
Not Found
Not Found
High
High
Not Found
Not Found
High
Not Found
Not Found
Not Found
Not Found
Not Found
Not Found
Not Found
Not Found
High
High
Not Found

High
Not Found
Not Found
Not Found
Not Found
Not Found
High
High
Not Found
Not Found
Not Found
High
Not Found
High
Not Found
Not Found
High
Not Found
Not Found
Not Found
Not Found
Not Found
Not Found
Not Found
Not Found
Not Found
Not Found
Not Found
Not Found
Not Found
High
Not Found
Not Found
High
Not Found
Not Found
Not Found
Not Found
Not Found
High
Not Found
Not Found
Not Found
Not Found
Not Found
Not Found
Not Found
High
High
High

Not Found
Not Found
Not Found
Not Found
Not Found
Not Found
Not Found
High
High
Not Found
High
Not Found
High
Not Found
Not Found
High
Not Found
