# Supplementary material for: Comprehensive Quantitative Proteome Analysis of Aedes aegypti Identifies Proteins and Pathways Involved in Wolbachia pipientis and Zika Virus Interference Phenomenon
Source: Front Physiol. 2021 Feb 25;12:642237. doi: 10.3389/fphys.2021.642237 (PMC7947915; doi:10.3389/fphys.2021.642237)
Supplement: Supplementary file 3 [file Data_Sheet_3.PDF]

Protein
AAEL026217-PA
AAEL005656-PB myosin heavy chain, nonmuscle or smooth muscle
AAEL009955-PB
AAEL001928-PA Act1: Actin-1
AAEL012062-PX Na+/K+ ATPase alpha subunit
AAEL002565-PD titin
AAEL010975-PB paramyosin, long form
AAEL012897-PA aconitase, mitochondrial
AAEL009847-PB microtubule-associated protein
AAEL002851-PA Tubulin beta chain
AAEL005422-PA pyrroline-5-carboxylate dehydrogenase
AAEL006138-PA
AAEL002759-PD tropomyosin invertebrate
AAEL011504-PA pupal cuticle protein, putative
AAEL006582-PH calcium-transporting ATPase sarcoplasmic/endoplasmic reticulum type
AAEL002761-PAM tropomyosin invertebrate
AAEL006126-PA
AAEL001194-PA FAS1: fatty acid synthase
AAEL004297-PI ATP-citrate synthase
AAEL012552-PA NADH-ubiquinone oxidoreductase
AAEL004988-PA Pgk: Phosphoglycerate kinase
AAEL006834-PA glutamate semialdehyde dehydrogenase
AAEL005798-PA ATP synthase subunit beta vacuolar
AAEL013535-PA phosrestin ii (arrestin a) (arrestin 1)
AAEL015314-PM cAMP-dependent protein kinase type ii regulatory subunit
AAEL024235-PG
AAEL004423-PA mitochondrial F0 ATP synthase D chain, putative
AAEL024583-PB
AAEL017301-PA Elongation factor 1-alpha
AAEL012326-PA calmodulin
AAEL010823-PA ATP synthase delta chain
AAEL008542-PF kinesin heavy chain subunit
AAEL017096-PA Elongation factor 1-alpha
AAEL017293-PA
AAEL008773-PA laminin A chain, putative
AAEL015458-PA Tf1: transferrin
AAEL000596-PB myosin
AAEL013279-PB peptidyl-prolyl cis-trans isomerase (cyclophilin)
AAEL011981-PA glutamate decarboxylase
AAEL013952-PD prohibitin
AAEL010143-PA isocitrate dehydrogenase
AAEL005997-PA allergen, putative
AAEL012207-PF myosin light chain 1,
AAEL001677-PA
AAEL008844-PA calcium-binding protein, putative
AAEL004088-PH aldo-keto reductase
AAEL011758-PA peptidyl-prolyl cis-trans isomerase f, ppif
AAEL014600-PA 4-hydroxyphenylpyruvate dioxygenase
AAEL001082-PA

AAEL004559-PJ synaptosomal associated protein
AAEL012827-PA endoplasmin
AAEL001683-PA
AAEL001473-PC dynamin-associated protein
AAEL007383-PE secreted ferritin G subunit precursor, putative
AAEL005407-PD annexin x
AAEL008789-PA apolipophorin-III, putative
AAEL006946-PA chaperonin
AAEL011090-PA complement component
AAEL009389-PA Transaldolase
AAEL023497-PA Protein-L-isoaspartate O-methyltransferase
AAEL004745-PA pupal cuticle protein, putative
AAEL019951-PG
AAEL000934-PI clathrin light chain
AAEL004041-PD flotillin-2
AAEL013338-PA lethal(2)essential for life protein, l2efl
AAEL010840-PB
AAEL001851-PB
AAEL004176-PB microtubule binding protein, putative
AAEL001794-PB macroglobulin/complement
AAEL019778-PA
AAEL022352-PA
AAEL001946-PA four and a half lim domains
AAEL017263-PA
AAEL006271-PD CUSOD2: copper-zinc (Cu-Zn) superoxide dismutase
AAEL002185-PA cuticle protein, putative
AAEL005946-PA NADH-ubiquinone oxidoreductase subunit B14.5b
AAEL006948-PB tomosyn
AAEL006027-PB lipase
AAEL012206-PB microtubule-associated protein tau
AAEL000762-PB LRIM19: leucine-rich immune protein (Coil-less)
AAEL013138-PA
AAEL003888-PD ubiquitin
AAEL014416-PC pupal cuticle protein 78E, putative
AAEL025999-PA 40S ribosomal protein S17
AAEL009257-PA
AAEL017212-PA
AAEL015424-PA adult cuticle protein, putative
AAEL009604-PG Receptor expression-enhancing protein
AAEL018211-PB
AAEL010168-PA RpS2: 40S ribosomal protein S2
AAEL012865-PA
AAEL004148-PA heat shock protein 70 (hsp70)-interacting protein
AAEL019767-PA
AAEL019793-PB
AAEL011982-PC cortactin
AAEL001864-PA eukaryotic translation initiation factor 4E binding protein (4EBP)
AAEL019718-PA
AAEL000028-PA CLIPB34: Clip-Domain Serine Protease family B.
AAEL007010-PA CYP6AG4: cytochrome P450

AAEL013808-PF fascin
AAEL006406-PG Putative 14.5 kDa secreted protein
AAEL009398-PA Pep12p, putative
AAEL018664-PA COX2: cytochrome c oxidase subunit II
AAEL000090-PB secretory carrier-associated membrane protein (scamp)
AAEL006719-PA AMY1: Alpha-amylase I Precursor (EC 3.2.1.1)(1,4-alpha-D-glucan glucanohydrolase)
AAEL006677-PA phospholipase a-2-activating protein
AAEL003954-PA juvenile hormone-inducible protein, putative
AAEL001402-PA LRIM10B: leucine-rich immune protein (Short)
AAEL003431-PA proteasome subunit beta type 7,10
AAEL005793-PA AMP dependent ligase
AAEL009653-PA RpS30: 40S ribosomal protein S30
AAEL008422-PD
AAEL009314-PA adenylate cyclase
AAEL002245-PA
AAEL001077-PA CLIPB45: Clip-Domain Serine Protease family B. Protease homologue.
AAEL004146-PA CRY1: cryptochrome 1
AAEL003104-PC tripartite motif protein trim2,3
AAEL006124-PA Mitochondrial import inner membrane translocase subunit TIM50
AAEL006464-PA
AAEL009642-PA cathepsin b
AAEL013119-PA charged multivesicular body protein
AAEL014372-PA juvenile hormone-inducible protein, putative
AAEL008764-PA cuticle protein, putative
AAEL010139-PA serine protease, putative
AAEL014961-PA gdp mannose-4,6-dehydratase
AAEL009863-PD sodium/dicarboxylate cotransporter, putative
AAEL000556-PA CTL25: C-Type Lectin (CTL25)
AAEL010491-PB FKBP12: fk506-binding protein
AAEL003188-PA sphingosine phosphate lyase
AAEL027103-PA
AAEL006002-PA
AAEL005469-PB
AAEL006971-PA
AAEL020992-PE
AAEL009974-PA ras-related protein Rab-8A, putative
AAEL009212-PG lola
AAEL011070-PA CTLGA3: C-Type Lectin (CTL) - galactose binding.
AAEL018353-PB
AAEL008250-PC
AAEL008490-PC NADH dehydrogenase, putative
AAEL026670-PA
AAEL002135-PA tubulin-specific chaperone b (tubulin folding cofactor b)
AAEL002350-PA
AAEL011388-PA Vacuolar-sorting protein SNF8
AAEL002317-PE InR: Insulin-like receptor Precursor (MIR)(EC 2.7.10.1)
AAEL010059-PA bacterial-type ABC transport ATP-binding subunit? or RNAse l inhibitor
AAEL022886-PA
AAEL015430-PB CLIPB19: Clip-Domain Serine Protease family B. Protease homologue.
AAEL011433-PA small nuclear ribonucleoprotein sm d3

AAEL019654-PB
AAEL015180-PB smooth muscle caldesmon, putative
AAEL003059-PB
AAEL010408-PD GPRGBB2: GPCR GABA B Family
AAEL006451-PB
AAEL022232-PA
AAEL014325-PA proteasome regulatory subunits
AAEL009833-PA mRpL46: mitochondrial ribosomal protein, L46, putative
AAEL021103-PA
AAEL011234-PC reticulon/nogo receptor
AAEL010681-PA sodium/chloride dependent neurotransmitter transporter
AAEL008458-PA
AAEL007805-PA
AAEL002591-PA OBP13: odorant binding protein OBP13
AAEL019579-PA
AAEL007821-PA signalosome, subunit 2, CSN8, putative
AAEL007420-PB SRPN25: Serine Protease Inhibitor (serpin) homologue - unlikely to be inhibitory
AAEL019517-PA
AAEL002915-PA
AAEL009589-PA
AAEL009375-PK plekhh1
AAEL009951-PA dimeric dihydrodiol dehydrogenase
AAEL014511-PE
AAEL029038-PA CECA: cecropin
AAEL002601-PA CLIPA1: Clip-Domain Serine Protease family A. Protease homologue.
AAEL024070-PA Putative small nuclear ribonucleoprotein snrnp smf
AAEL000937-PA
AAEL000330-PA
AAEL005944-PA mRpS23: mitochondrial ribosomal protein, S23, putative
AAEL018131-PB
AAEL003991-PH alcohol dehydrogenase
AAEL020192-PA
AAEL021230-PA
AAEL009026-PD ubiquitin-conjugating enzyme m
AAEL004300-PB
AAEL001950-PB
AAEL023091-PA
AAEL013989-PA protein translocation complex beta subunit, putative
AAEL004406-PD
AAEL012841-PA
AAEL011216-PG Phosphatidylinositol-4,5-bisphosphate 4-phosphatase
AAEL002102-PB
AAEL018117-PB
AAEL010791-PA Autophagy-specific protein, putative
AAEL014516-PA metalloproteinase, putative
AAEL011890-PC
AAEL008557-PA

(-Log ANOVA p-value)	Significant pairs (Up-regulated_down-regulated)
4,271777813	115_117;116_117;115_114;116_114
2,514001451	116_117;116_114;116_115
2,195629072	116_114;116_115
1,800850488	115_114
7,11879894	117_116;114_116;115_116;114_117;115_117;115_114
1,806231463	116_117;115_117
1,314499523	115_114
4,240561485	117_114;115_114;116_114;116_117;116_115
1,77714195	116_115
1,552090751	116_114
2,184255666	116_114
4,517435874	117_114;116_114;117_115;116_115
2,121497626	116_114;116_117
1,607203142	117_114
4,911628212	116_114;115_114;116_117;115_117;115_116
1,677982154	117_114;115_114
4,511369267	115_114;116_114;117_114;116_115;117_115
1,811428505	116_117;114_117
2,526180238	116_117;115_117;115_114
1,66727909	116_117;116_114
3,077106946	117_114;116_114;117_115;116_115
1,316789414	116_117
1,549407725	115_114
2,17733154	116_117;116_115
2,098280165	117_115;117_114
2,840685255	116_117;115_117;116_114;115_114
1,402250531	115_114
2,16614374	115_117;115_114
1,855121353	116_114;116_117
6,629410461	117_114;116_114;115_114;116_117;115_117
2,406253156	116_115;116_114;116_117
2,621489163	116_117;116_114;116_115
1,635565462	116_114;116_115
2,296841727	116_114;116_115
1,916965629	117_115;117_114
2,50568231	116_115;116_114
2,481800641	116_117;115_117;115_114
4,651840282	116_115;117_115;117_114;117_116
3,261398284	117_114;116_114;116_115
2,15301324	117_114;115_114;115_116
1,974182672	116_114
1,313495456	116_114
1,319537308	116_114
1,609275972	116_115
2,040394666	116_114;116_117
2,504453412	114_115;116_115
2,772874519	116_114;117_114;115_114
5,467053042	114_115;116_115;114_117;116_117;116_114
1,829540732	117_114;116_114

1,686140462	114_117;114_116
1,859306212	114_115;116_115
2,403005778	116_115;116_117
1,404560111	116_114
1,89879228	116_114;117_114
3,750898348	117_114;115_114;116_114;116_117;116_115
6,190366803	116_114;115_114;117_114;115_116;117_116;117_115
1,282782467	115_114
2,669480529	116_115;117_115;117_114
1,98264351	117_115;116_115;116_114
2,775892202	116_114;117_114
2,586973455	115_114;117_114;116_114
4,789914023	117_114;115_114;116_114;116_117;116_115
1,919802572	116_115
1,321402995	115_117
1,487090987	117_114;117_116
2,229411597	117_115;116_115
4,012423993	117_115;114_115;116_115;116_117
2,027657273	116_115
1,757699803	116_115
2,545595403	115_114;117_114;116_114
1,719161308	117_116;117_114
1,870893776	116_114
2,835314331	114_115;116_115;116_117
1,612285952	116_114;117_114
2,210230165	116_114
3,524279291	117_114;115_114;116_114
1,630732328	115_117
2,512978955	116_114;115_114
3,461529529	117_116;114_116;117_115;114_115
2,533734366	114_115;116_115;117_115
2,190112627	117_115;117_114
1,499108348	116_117;116_115
1,95874832	117_116
1,533198334	117_115
1,735940859	116_114
4,19347108	114_115;117_115;116_115;116_114;116_117
2,324346555	116_115;116_117;116_114
2,983369006	115_114;116_114;115_117;116_117
4,186777407	115_116;114_116;117_116;117_115
1,566481125	116_114
1,317062128	116_115
1,394760066	116_115
2,164853975	114_116;117_116
1,761699228	117_114;115_114
1,798033943	117_115
1,306963121	115_114
1,510324956	115_114
1,708280762	117_115;117_116
1,700443182	114_116;115_116

1,388013685	114_115
1,957996691	116_115;116_117
1,979171968	115_117;115_114
2,547584077	115_114;115_117
2,674457264	114_116;115_116
1,919418688	114_116;114_117
1,334478579	116_114
2,79570589	116_115;116_117;116_114
2,43294121	116_115;117_115;114_115
1,308314374	116_115
5,868868376	114_116;117_116;115_116;115_114;115_117
6,088741815	115_116;114_116;117_116;117_115;117_114
1,806945766	117_115;114_115
1,930417494	115_117;115_114;115_116
3,537852527	116_117;114_117;116_115;114_115
1,565423585	117_115
1,391490707	115_116
3,162779729	117_116;115_116;115_114
2,7915452	115_114;117_114;115_116;117_116
1,348559818	117_114
2,135923633	117_114;117_116;117_115
2,331529326	117_116;117_115
2,942325794	116_114;116_117;116_115
3,385118481	117_116;115_116;117_114;115_114
2,963764022	117_115;116_115;114_115
2,125006815	114_117;114_116;114_115
3,502744434	115_114;115_117;115_116
2,692047592	117_116;115_116;114_116
1,445125001	114_115
3,331676114	117_116;115_116;115_114;115_117
1,821833076	114_115;114_116;114_117
2,106633723	115_117;115_116
2,324487859	114_116
1,967613183	116_114;115_114
1,369902298	115_116
1,757153294	114_117;114_116
1,512195091	114_115;116_115
2,201901256	116_114;117_114
1,570541993	115_114
2,137421729	117_114;116_114;117_115;116_115
2,234786037	114_116;114_115
5,34600802	116_114;115_114;116_117;115_117;115_116
1,71317976	114_115
2,070802008	115_114;115_117
6,982654776	116_114;117_114;115_114;115_116
2,088114025	114_116;115_116
3,507872757	114_115;117_115;116_115;116_114;116_117
2,240673165	115_114;115_117;115_116
2,153234226	114_115
1,310683895	117_116

2,186666781 117_114;116_114;115_114
2,511924586 117_114;115_114;117_116;115_116
1,594979945 115_116
1,416228099 114_117
1,791040044 114_116;117_116
3,004180084 114_116;117_116;115_116;115_114
1,833896368 116_115;116_117;116_114
1,348750399 116_115
2,295144608 117_116;115_116;115_114
2,780134195 117_116;114_116;115_116
2,743872823 114_117;114_115;114_116
3,128904889 117_115;114_115;117_116;114_116
3,307690677 116_115;117_115;114_115;117_116;114_116
3,101064427 114_115;117_115;114_116;117_116
3,390568158 116_114;115_114;117_114;115_116;117_116
2,05489339 114_116;117_116
3,948336385 117_116;115_116;114_116;114_117;114_115
2,852828682 116_114;115_114;116_117;115_117
1,982345339 116_117;114_117;116_115;114_115
1,809032341 114_117;114_115;114_116
1,648776888 116_117;116_115
1,395167766 114_115
1,422163284 117_116
4,563490639 117_115;116_115;116_114;116_117
2,487458073 117_115;116_115;114_115;114_117;114_116
2,10243834 117_115;114_115;116_115
1,954272132 117_116
1,700894246 115_116;117_116;114_116
1,850199634 117_116;115_116;114_116
2,234278377 117_115;114_115;114_116
2,241277965 116_117;114_117;115_117
2,866821279 117_115;116_115;114_115
2,059537005 117_116;117_115;117_114
3,465385818 117_115;116_115;114_115;116_117;114_117
1,683474263 115_114
1,355021154 115_116
2,133515319 114_115;117_115;117_116
2,973482988 114_117;114_116;114_115
1,397828331 114_115
1,589217982 115_117
3,386553816 115_114;117_114;116_114;116_115;116_117
2,230950028 117_115;114_115;116_115
3,946110261 116_115;117_115;114_115;117_116;114_116;114_117
3,986947222 117_114;116_114;115_114
3,17627519 114_117;114_116;114_115
2,288170865 117_114;115_114;115_116
2,838859624 114_117;115_117;114_116;115_116
