# Supplementary material for: Comprehensive Quantitative Proteome Analysis of Aedes aegypti Identifies Proteins and Pathways Involved in Wolbachia pipientis and Zika Virus Interference Phenomenon
Source: Front Physiol. 2021 Feb 25;12:642237. doi: 10.3389/fphys.2021.642237 (PMC7947915; doi:10.3389/fphys.2021.642237)
Supplement: Supplementary file 4 [file Data_Sheet_4.PDF]

Up regulated				
ID	Name	P-value	Benjamini	Bonferroni
GO:00067!	phosphate-containing compound metabolic proces	0.000559	0.058999	0.116889
GO:00067!	phosphorus metabolic process	0.000583	0.058999	0.121842
GO:00171	drug metabolic process	0.000847	0.058999	0.176997
GO:00442	small molecule metabolic process	0.002083	0.072132	0.435254
GO:00061	citrate metabolic process	0.002216	0.072132	0.463097
GO:00723	tricarboxylic acid metabolic process	0.002413	0.072132	0.504229
GO:00091	purine ribonucleotide metabolic process	0.003775	0.072132	0.788942
GO:00061	purine nucleotide metabolic process	0.003879	0.072132	0.810685
GO:00169	antibiotic metabolic process	0.00427	0.072132	0.892361
GO:00092	ribonucleotide metabolic process	0.004313	0.072132	0.901315
GO:00197	carboxylic acid metabolic process	0.004893	0.072132	1
GO:00434	oxoacid metabolic process	0.004969	0.072132	1
GO:00196	ribose phosphate metabolic process	0.005016	0.072132	1
GO:00060	organic acid metabolic process	0.005125	0.072132	1
GO:00163	phosphorylation	0.005453	0.072132	1
GO:00725	purine-containing compound metabolic process	0.005522	0.072132	1
GO:00070	cytoskeletal anchoring at plasma membrane	0.006121	0.075251	1
GO:00196	organophosphate metabolic process	0.00706	0.081971	1
GO:00453	cellular respiration	0.008674	0.08596	1
GO:00159	energy derivation by oxidation of organic compoun	0.010986	0.08596	1
GO:00067	nucleoside phosphate metabolic process	0.011259	0.08596	1
GO:00091	nucleotide metabolic process	0.011259	0.08596	1
GO:00460	ATP metabolic process	0.011395	0.08596	1
GO:00065	proline biosynthetic process	0.012205	0.08596	1
GO:00516	maintenance of location in cell	0.012205	0.08596	1
GO:00551	L-proline biosynthetic process	0.012205	0.08596	1
GO:00197	calcium-mediated signaling	0.012205	0.08596	1
GO:00325	maintenance of protein location in cell	0.012205	0.08596	1
GO:00091	purine nucleoside triphosphate metabolic process	0.01354	0.08596	1
GO:00092	purine ribonucleoside triphosphate metabolic proc	0.01354	0.08596	1
GO:00091	ribonucleoside triphosphate metabolic process	0.014443	0.08596	1
GO:00716	acyl-CoA biosynthetic process	0.015234	0.08596	1
GO:00451	maintenance of protein location	0.015234	0.08596	1
GO:00353	thioester biosynthetic process	0.015234	0.08596	1
GO:00060	acetyl-CoA biosynthetic process	0.015234	0.08596	1
GO:00199	second-messenger-mediated signaling	0.015234	0.08596	1
GO:00091	nucleoside triphosphate metabolic process	0.015372	0.08596	1
GO:00091	purine ribonucleoside monophosphate metabolic p	0.017304	0.08596	1
GO:00091	purine nucleoside monophosphate metabolic proc	0.017304	0.08596	1
GO:00551	oxidation-reduction process	0.017776	0.08596	1
GO:00550	nucleobase-containing small molecule metabolic p	0.017903	0.08596	1
GO:00065	proline metabolic process	0.018254	0.08596	1
GO:00091	ribonucleoside monophosphate metabolic process	0.019857	0.08596	1
GO:00091	purine ribonucleotide biosynthetic process	0.02092	0.08596	1
GO:00197	antibacterial humoral response	0.021264	0.08596	1
GO:00468	phosphatidylinositol dephosphorylation	0.021264	0.08596	1
GO:00197	antimicrobial humoral response	0.021264	0.08596	1
GO:00512	maintenance of location	0.021264	0.08596	1
GO:00061	purine nucleotide biosynthetic process	0.02146	0.08596	1
GO:00091	nucleoside monophosphate metabolic process	0.022558	0.08596	1

GO:007252	purine-containing compound biosynthetic process	0.023679	0.08596	1
GO:000926	ribonucleotide biosynthetic process	0.023679	0.08596	1
GO:004639	ribose phosphate biosynthetic process	0.023679	0.08596	1
GO:003403	purine nucleoside bisphosphate biosynthetic proce	0.024266	0.08596	1
GO:004683	phospholipid dephosphorylation	0.024266	0.08596	1
GO:000608	acetyl-CoA metabolic process	0.024266	0.08596	1
GO:003386	nucleoside bisphosphate biosynthetic process	0.024266	0.08596	1
GO:000695	humoral immune response	0.024266	0.08596	1
GO:003403	ribonucleoside bisphosphate biosynthetic process	0.024266	0.08596	1
GO:000908	glutamine family amino acid biosynthetic process	0.027259	0.091889	1
GO:003538	thioester metabolic process	0.027259	0.091889	1
GO:000663	acyl-CoA metabolic process	0.027259	0.091889	1
GO:005170	response to other organism	0.033218	0.102096	1
GO:000960	response to biotic stimulus	0.033218	0.102096	1
GO:009854	defense response to other organism	0.033218	0.102096	1
GO:000961	response to bacterium	0.033218	0.102096	1
GO:004274	defense response to bacterium	0.033218	0.102096	1
GO:004320	response to external biotic stimulus	0.033218	0.102096	1
GO:000907	aromatic amino acid family metabolic process	0.036184	0.109073	1
GO:000646	protein phosphorylation	0.038118	0.109073	1
GO:000916	nucleotide biosynthetic process	0.038811	0.109073	1
GO:190129	nucleoside phosphate biosynthetic process	0.038811	0.109073	1
GO:003403	purine nucleoside bisphosphate metabolic process	0.039141	0.109073	1
GO:003387	ribonucleoside bisphosphate metabolic process	0.039141	0.109073	1
GO:003386	nucleoside bisphosphate metabolic process	0.039141	0.109073	1
GO:000609	generation of precursor metabolites and energy	0.045959	0.126386	1
GO:000662	lipid metabolic process	0.049467	0.134267	1

Down regulated

ID	Name	P-value	Benjamini	Bonferroni
GO:001038	COP9 signalosome assembly	0.005582	0.323225	0.586063
GO:000033	protein deneddylation	0.008361	0.323225	0.877906
GO:001953	protein metabolic process	0.009235	0.323225	0.969674
GO:000038	spliceosomal snRNP assembly	0.024881	0.443185	1
GO:000703	vacuolar transport	0.035745	0.443185	1
GO:190156	organonitrogen compound metabolic process	0.040162	0.443185	1
GO:000650	proteolysis	0.044855	0.443185	1
