# Supplementary material for: Comprehensive Quantitative Proteome Analysis of Aedes aegypti Identifies Proteins and Pathways Involved in Wolbachia pipientis and Zika Virus Interference Phenomenon
Source: Front Physiol. 2021 Feb 25;12:642237. doi: 10.3389/fphys.2021.642237 (PMC7947915; doi:10.3389/fphys.2021.642237)
Supplement: Supplementary file 5 [file Data_Sheet_5.PDF]

Up regulated				
ID	Name	P-value	Benjamini	Bonferroni
GO:00068€	lipid transport	0.000345	0.039955	0.070793
GO:00108ï	lipid localization	0.00039	0.039955	0.079909
GO:00193€	pyridine nucleotide metabolic process	0.002509	0.119231	0.514413
GO:00464¢	nicotinamide nucleotide metabolic process	0.002509	0.119231	0.514413
GO:00725Z	pyridine-containing compound metabolic p	0.002908	0.119231	0.596155
GO:00067З	oxidoreduction coenzyme metabolic proces	0.00427	0.14588	0.875283
GO:20004ï	regulation of cAMP-dependent protein kinas	0.00863	0.235486	1
GO:00196€	glyceraldehyde-3-phosphate metabolic proc	0.017188	0.235486	1
GO:00060¢	pentose-phosphate shunt	0.017188	0.235486	1
GO:00511!	glucose 6-phosphate metabolic process	0.017188	0.235486	1
GO:00067З	coenzyme metabolic process	0.017233	0.235486	1
GO:00197З	antibacterial humoral response	0.020024	0.235486	1
GO:00197З	antimicrobial humoral response	0.020024	0.235486	1
GO:19015€	organonitrogen compound metabolic proce	0.020045	0.235486	1
GO:00069!	humoral immune response	0.022853	0.235486	1
GO:00067З	NADP metabolic process	0.022853	0.235486	1
GO:00003€	spliceosomal snRNP assembly	0.025674	0.235486	1
GO:00517(	response to other organism	0.031292	0.235486	1
GO:00096ᴉ	response to bacterium	0.031292	0.235486	1
GO:00985⁄	defense response to other organism	0.031292	0.235486	1
GO:00427⁄	defense response to bacterium	0.031292	0.235486	1
GO:00432(	response to external biotic stimulus	0.031292	0.235486	1
GO:00096(	response to biotic stimulus	0.031292	0.235486	1
GO:00195З	protein metabolic process	0.032515	0.235486	1
GO:00428€	pyruvate biosynthetic process	0.034089	0.235486	1
GO:00067!	ATP generation from ADP	0.034089	0.235486	1
GO:00060¢	glycolytic process	0.034089	0.235486	1
GO:00060€	cellular aldehyde metabolic process	0.036878	0.235486	1
GO:00070З	vacuolar transport	0.036878	0.235486	1
GO:00511€	cofactor metabolic process	0.037888	0.235486	1
GO:00330З	macromolecule localization	0.037924	0.235486	1
GO:00460З	ADP metabolic process	0.03966	0.235486	1
GO:00717(	organic substance transport	0.040165	0.235486	1
GO:00091€	ribonucleoside diphosphate metabolic proc	0.042434	0.235486	1
GO:00091З	purine nucleoside diphosphate metabolic pı	0.042434	0.235486	1
GO:00091ï	purine ribonucleoside diphosphate metabol	0.042434	0.235486	1
GO:00196¢	ribose phosphate metabolic process	0.042502	0.235486	1
GO:00061€	nucleoside diphosphate phosphorylation	0.0452	0.237589	1
GO:00469З	nucleotide phosphorylation	0.0452	0.237589	1
GO:00060¢	pyruvate metabolic process	0.047958	0.239791	1
GO:00160!	carbohydrate catabolic process	0.047958	0.239791	1

Down regulated				
ID	Name	P-value	Benjamini	Bonferroni
GO:00068ᴉ	transport	1.47E-05	0.001101	0.002497
GO:00512З	establishment of localization	1.60E-05	0.001101	0.002712
GO:00511ï	localization	1.94E-05	0.001101	0.003304
GO:00068ᴉ	cation transport	0.000868	0.036891	0.147565
GO:00550€	transmembrane transport	0.00111	0.037738	0.188689
GO:00300(	metal ion transport	0.002897	0.065283	0.492533

GO:001567	monovalent inorganic cation transport	0.003149	0.065283	0.535283
GO:001619	vesicle-mediated transport	0.00362	0.065283	0.61547
GO:009866	inorganic cation transmembrane transport	0.003763	0.065283	0.639659
GO:000701	cytoskeletal anchoring at plasma membrane	0.004323	0.065283	0.734836
GO:000642	cysteinyl-tRNA aminoacylation	0.004323	0.065283	0.734836
GO:009865	cation transmembrane transport	0.004855	0.065283	0.825367
GO:000681	ion transport	0.005212	0.065283	0.886065
GO:009866	inorganic ion transmembrane transport	0.005376	0.065283	0.913957
GO:005165	maintenance of location in cell	0.008627	0.083245	1
GO:003250	maintenance of protein location in cell	0.008627	0.083245	1
GO:001972	calcium-mediated signaling	0.008627	0.083245	1
GO:007161	acyl-CoA biosynthetic process	0.010773	0.083245	1
GO:001993	second-messenger-mediated signaling	0.010773	0.083245	1
GO:004518	maintenance of protein location	0.010773	0.083245	1
GO:003538	thioester biosynthetic process	0.010773	0.083245	1
GO:000608	acetyl-CoA biosynthetic process	0.010773	0.083245	1
GO:001975	carboxylic acid metabolic process	0.012464	0.087802	1
GO:004343	oxoacid metabolic process	0.012612	0.087802	1
GO:000608	organic acid metabolic process	0.012912	0.087802	1
GO:005164	cellular localization	0.014312	0.088519	1
GO:005123	maintenance of location	0.015051	0.088519	1
GO:003422	ion transmembrane transport	0.016483	0.088519	1
GO:003386	nucleoside bisphosphate biosynthetic proce	0.017183	0.088519	1
GO:006102	membrane fusion	0.017183	0.088519	1
GO:000608	acetyl-CoA metabolic process	0.017183	0.088519	1
GO:003403	purine nucleoside bisphosphate biosyntheti	0.017183	0.088519	1
GO:003403	ribonucleoside bisphosphate biosynthetic p	0.017183	0.088519	1
GO:000663	acyl-CoA metabolic process	0.019311	0.093796	1
GO:003538	thioester metabolic process	0.019311	0.093796	1
GO:003387	ribonucleoside bisphosphate metabolic pro	0.027778	0.124272	1
GO:003403	purine nucleoside bisphosphate metabolic p	0.027778	0.124272	1
GO:003386	nucleoside bisphosphate metabolic process	0.027778	0.124272	1
GO:003461	cellular protein localization	0.041277	0.177553	1
GO:007072	cellular macromolecule localization	0.041777	0.177553	1
GO:006500	regulation of biological quality	0.04329	0.179393	1
GO:004427	sulfur compound biosynthetic process	0.046575	0.179393	1
GO:007058	calcium ion transmembrane transport	0.046575	0.179393	1
GO:000681	calcium ion transport	0.048642	0.179393	1
GO:000610	citrate metabolic process	0.048642	0.179393	1
GO:000689	endocytosis	0.048642	0.179393	1
