# Supplementary material for: Comprehensive Quantitative Proteome Analysis of Aedes aegypti Identifies Proteins and Pathways Involved in Wolbachia pipientis and Zika Virus Interference Phenomenon
Source: Front Physiol. 2021 Feb 25;12:642237. doi: 10.3389/fphys.2021.642237 (PMC7947915; doi:10.3389/fphys.2021.642237)
Supplement: Supplementary file 6 [file Data_Sheet_6.PDF]

Up regulated			
ID	Name	P-value	Benjamini
GO:000609	generation of precursor metabolites and energy	1.44E-05	0.0047389
GO:004428	small molecule metabolic process	4.03E-05	0.0053581
GO:004533	cellular respiration	6.53E-05	0.0053581
GO:000686	lipid transport	7.73E-05	0.0053581
GO:001087	lipid localization	9.09E-05	0.0053581
GO:001598	energy derivation by oxidation of organic compounds	0.000106	0.0053581
GO:004603	ATP metabolic process	0.000114	0.0053581
GO:000914	purine nucleoside triphosphate metabolic process	0.000163	0.0057683
GO:000920	purine ribonucleoside triphosphate metabolic process	0.000163	0.0057683
GO:000919	ribonucleoside triphosphate metabolic process	0.000186	0.0057683
GO:001969	ribose phosphate metabolic process	0.000203	0.0057683
GO:000914	nucleoside triphosphate metabolic process	0.000211	0.0057683
GO:000912	purine nucleoside monophosphate metabolic process	0.000269	0.0062985
GO:000916	purine ribonucleoside monophosphate metabolic process	0.000269	0.0062985
GO:000916	ribonucleoside monophosphate metabolic process	0.000356	0.0077868
GO:000912	nucleoside monophosphate metabolic process	0.000462	0.0094710
GO:001975	carboxylic acid metabolic process	0.000707	0.0122933
GO:004343	oxoacid metabolic process	0.000723	0.0122933
GO:000608	organic acid metabolic process	0.000757	0.0122933
GO:000911	nucleotide metabolic process	0.000787	0.0122933
GO:000675	nucleoside phosphate metabolic process	0.000787	0.0122933
GO:001714	drug metabolic process	0.001074	0.0160072
GO:005507	iron ion homeostasis	0.001198	0.0166146
GO:001963	organophosphate metabolic process	0.001216	0.0166146
GO:003303	macromolecule localization	0.001399	0.0182283
GO:000915	purine ribonucleotide metabolic process	0.001448	0.0182283
GO:000616	purine nucleotide metabolic process	0.001501	0.0182283
GO:005507	transition metal ion homeostasis	0.001689	0.0188538
GO:005508	nucleobase-containing small molecule metabolic process	0.001707	0.0188538
GO:000925	ribonucleotide metabolic process	0.001724	0.0188538
GO:007252	purine-containing compound metabolic process	0.002384	0.0249417
GO:004423	cellular metabolic process	0.002433	0.0249417
GO:004277	ATP synthesis coupled electron transport	0.003636	0.0361381
GO:000611	oxidative phosphorylation	0.004028	0.0377436
GO:005506	metal ion homeostasis	0.004028	0.0377436
GO:002290	electron transport chain	0.00432	0.0387338
GO:001013	proline catabolic process to glutamate	0.004778	0.0387338
GO:000609	tricarboxylic acid cycle	0.004867	0.0387338
GO:002290	respiratory electron transport chain	0.004867	0.0387338
GO:000681	transport	0.005098	0.0387338
GO:005511	oxidation-reduction process	0.005262	0.0387338
GO:005080	ion homeostasis	0.005314	0.0387338
GO:000610	citrate metabolic process	0.005314	0.0387338
GO:009877	inorganic ion homeostasis	0.005314	0.0387338
GO:005508	cation homeostasis	0.005314	0.0387338
GO:005123	establishment of localization	0.005468	0.0389891
GO:007235	tricarboxylic acid metabolic process	0.00578	0.0394935
GO:000641	translational elongation	0.00578	0.0394935
GO:000906	aerobic respiration	0.006263	0.0419236
GO:005117	localization	0.006462	0.0423930

GO:004649	nicotinamide nucleotide metabolic process	0.006764	0.0426668
GO:001936	pyridine nucleotide metabolic process	0.006764	0.0426668
GO:004887	chemical homeostasis	0.007283	0.0450732
GO:007252	pyridine-containing compound metabolic process	0.00782	0.0474966
GO:000675	ATP biosynthetic process	0.008373	0.0494569
GO:007170	organic substance transport	0.008444	0.0494569
GO:003251	late endosome to vacuole transport via multivesicular body sorting pat	0.009533	0.0512603
GO:004532	late endosome to vacuole transport	0.009533	0.0512603
GO:004332	protein transport to vacuole involved in ubiquitin-dependent protein c	0.009533	0.0512603
GO:000701	cytoskeletal anchoring at plasma membrane	0.009533	0.0512603
GO:000656	proline catabolic process	0.009533	0.0512603
GO:001699	antibiotic metabolic process	0.010136	0.0519493
GO:000920	purine ribonucleoside triphosphate biosynthetic process	0.010136	0.0519493
GO:000914	purine nucleoside triphosphate biosynthetic process	0.010136	0.0519493
GO:000920	ribonucleoside triphosphate biosynthetic process	0.011395	0.0557860
GO:000914	nucleoside triphosphate biosynthetic process	0.011395	0.0557860
GO:000673	oxidoreduction coenzyme metabolic process	0.011395	0.0557860
GO:000998	cellular process	0.012279	0.0592280
GO:000906	glutamine family amino acid catabolic process	0.014266	0.0659061
GO:004316	ubiquitin-dependent protein catabolic process via the multivesicular b	0.014266	0.0659061
GO:000653	glutamate metabolic process	0.014266	0.0659061
GO:000681	cation transport	0.015856	0.0722341
GO:000912	purine nucleoside monophosphate biosynthetic process	0.01707	0.0756596
GO:000916	purine ribonucleoside monophosphate biosynthetic process	0.01707	0.0756596
GO:003250	maintenance of protein location in cell	0.018977	0.0787912
GO:007266	establishment of protein localization to vacuole	0.018977	0.0787912
GO:007266	protein localization to vacuole	0.018977	0.0787912
GO:005165	maintenance of location in cell	0.018977	0.0787912
GO:001972	calcium-mediated signaling	0.018977	0.0787912
GO:000681	ion transport	0.020678	0.0844362
GO:190156	organonitrogen compound metabolic process	0.021089	0.0844362
GO:000915	ribonucleoside monophosphate biosynthetic process	0.021109	0.0844362
GO:000912	nucleoside monophosphate biosynthetic process	0.022826	0.0892234
GO:004424	cellular catabolic process	0.023209	0.0892234
GO:003250	endosome transport via multivesicular body sorting pathway	0.023666	0.0892234
GO:001993	second-messenger-mediated signaling	0.023666	0.0892234
GO:004518	maintenance of protein location	0.023666	0.0892234
GO:005115	glucose 6-phosphate metabolic process	0.028333	0.0929315
GO:007145	cellular response to superoxide	0.028333	0.0929315
GO:007145	cellular response to oxygen radical	0.028333	0.0929315
GO:007198	multivesicular body sorting pathway	0.028333	0.0929315
GO:000030	response to reactive oxygen species	0.028333	0.0929315
GO:001968	glyceraldehyde-3-phosphate metabolic process	0.028333	0.0929315
GO:003461	cellular response to reactive oxygen species	0.028333	0.0929315
GO:003201	regulation of ARF protein signal transduction	0.028333	0.0929315
GO:000609	pentose-phosphate shunt	0.028333	0.0929315
GO:000656	proline metabolic process	0.028333	0.0929315
GO:000030	response to superoxide	0.028333	0.0929315
GO:001943	removal of superoxide radicals	0.028333	0.0929315
GO:000030	response to oxygen radical	0.028333	0.0929315
GO:190260	proton transmembrane transport	0.029265	0.0950401
GO:000679	phosphate-containing compound metabolic process	0.031571	0.0957611

GO:190113	carbohydrate derivative metabolic process	0.031783	0.0957611
GO:190157	organic substance catabolic process	0.031954	0.0957611
GO:000679	phosphorus metabolic process	0.032508	0.0957611
GO:004685	phosphatidylinositol dephosphorylation	0.032978	0.0957611
GO:001973	antimicrobial humoral response	0.032978	0.0957611
GO:001973	antibacterial humoral response	0.032978	0.0957611
GO:004364	dicarboxylic acid metabolic process	0.032978	0.0957611
GO:005123	maintenance of location	0.032978	0.0957611
GO:003459	cellular response to oxidative stress	0.032978	0.0957611
GO:000682	iron ion transport	0.032978	0.0957611
GO:009866	inorganic cation transmembrane transport	0.032991	0.0957611
GO:190156	organonitrogen compound catabolic process	0.034139	0.0982248
GO:006500	regulation of biological quality	0.0371	0.1036393
GO:000687	cellular iron ion homeostasis	0.037601	0.1036393
GO:000673	NADP metabolic process	0.037601	0.1036393
GO:004683	phospholipid dephosphorylation	0.037601	0.1036393
GO:000695	humoral immune response	0.037601	0.1036393
GO:004423	primary metabolic process	0.040826	0.1107389
GO:009865	cation transmembrane transport	0.041461	0.1107389
GO:000905	catabolic process	0.041939	0.1107389
GO:000612	mitochondrial electron transport, NADH to ubiquinone	0.042202	0.1107389
GO:004691	cellular transition metal ion homeostasis	0.042202	0.1107389
GO:001003	response to inorganic substance	0.042202	0.1107389
GO:007170	organic substance metabolic process	0.042991	0.1119122
GO:000673	coenzyme metabolic process	0.044	0.1136380
GO:009866	inorganic ion transmembrane transport	0.045397	0.1163296
GO:000915	purine ribonucleotide biosynthetic process	0.047452	0.1197675
GO:000616	purine nucleotide biosynthetic process	0.048624	0.1197675
GO:004259	homeostatic process	0.049807	0.1197675
GO:000651	ubiquitin-dependent protein catabolic process	0.049807	0.1197675

Down Regulated

ID	Name	P-value	Benjamini
GO:000926	response to pH	0.002614	0.0605637
GO:007146	cellular response to alkaline pH	0.002614	0.0605637
GO:005129	protein heterooligomerization	0.002614	0.0605637
GO:007146	cellular response to pH	0.002614	0.0605637
GO:005129	protein heterotetramerization	0.002614	0.0605637
GO:001044	response to alkaline pH	0.002614	0.0605637
GO:003286	cellular response to insulin stimulus	0.005222	0.0639645
GO:003286	response to insulin	0.005222	0.0639645
GO:000828	insulin receptor signaling pathway	0.005222	0.0639645
GO:004677	protein autophosphorylation	0.005222	0.0639645
GO:001038	COP9 signalosome assembly	0.005222	0.0639645
GO:000033	protein deneddylation	0.007823	0.0639645
GO:190165	response to peptide	0.007823	0.0639645
GO:001967	GDP-mannose metabolic process	0.007823	0.0639645
GO:007137	cellular response to peptide hormone stimulus	0.007823	0.0639645
GO:190165	cellular response to peptide	0.007823	0.0639645
GO:004343	response to peptide hormone	0.007823	0.0639645
GO:007121	cellular response to abiotic stimulus	0.010418	0.0724017
GO:005126	protein tetramerization	0.010418	0.0724017

GO:01040(	cellular response to environmental stimulus	0.010418	0.0724017
GO:007141	cellular response to organonitrogen compound	0.013005	0.0860838
GO:00195Ξ	protein metabolic process	0.017275	0.1091448
GO:19016Ç	cellular response to nitrogen compound	0.018162	0.1097604
GO:00092Ż	nucleotide-sugar metabolic process	0.02073	0.1200624
GO:00071€	transmembrane receptor protein tyrosine kinase signaling pathway	0.025848	0.1437124
GO:00102Ź	response to organonitrogen compound	0.028397	0.1518123
GO:19016Ç	response to nitrogen compound	0.033475	0.1723353
GO:00442€	cellular protein metabolic process	0.038516	0.1846698
GO:19017(	cellular response to oxygen-containing compound	0.038528	0.1846698
GO:19017(	response to oxygen-containing compound	0.041045	0.1901760
GO:00512Ŝ	protein complex oligomerization	0.04606	0.2065271

Bonferroni
0.00473890005384
0.0132328593713
0.0214248141808
0.0253669967639
0.0298125626848
0.0347991586463
0.0375073364312
0.0533937855641
0.0533937855641
0.0609368864357
0.0666758204254
0.0692207722645
0.0881791678409
0.0881791678409
0.116802219911
0.151536718726
0.231755107369
0.237154957767
0.248247428917
0.258160030814
0.258160030814
0.352159912419
0.393071524632
0.398751037029
0.458942392647
0.474901489338
0.492165551187
0.554044671508
0.559934989826
0.565614507865
0.781984433582
0.798136251065
1.0
1.0
1.0
1.0
1.0
1.0
1.0
1.0
1.0
1.0
1.0
1.0
1.0
1.0
1.0
1.0
1.0
1.0

1.0
1.0
1.0
1.0
1.0
1.0
1.0
1.0
1.0
1.0
1.0
1.0
1.0
1.0
1.0
1.0
1.0
1.0
1.0
1.0
1.0
1.0
1.0
1.0
1.0
1.0
1.0
1.0
1.0
1.0
1.0
1.0
1.0
1.0
1.0
1.0
1.0
1.0
1.0
1.0
1.0
1.0
1.0
1.0
1.0
1.0
1.0
1.0
1.0
1.0
1.0
1.0

1.0
1.0
1.0
1.0
1.0
1.0
1.0
1.0
1.0
1.0
1.0
1.0
1.0
1.0
1.0
1.0
1.0
1.0
1.0
1.0
1.0
1.0
1.0
1.0
1.0
1.0
1.0
1.0
1.0
1.0

Bonferroni
0.363382313168
0.363382313168
0.363382313168
0.363382313168
0.363382313168
0.363382313168
0.725847325148
0.725847325148
0.725847325148
0.725847325148
0.725847325148
1.0
1.0
1.0
1.0
1.0
1.0
1.0
1.0

1.0
1.0
1.0
1.0
1.0
1.0
1.0
1.0
1.0
1.0
1.0
1.0
