# Supplementary material for: Comprehensive Quantitative Proteome Analysis of Aedes aegypti Identifies Proteins and Pathways Involved in Wolbachia pipientis and Zika Virus Interference Phenomenon
Source: Front Physiol. 2021 Feb 25;12:642237. doi: 10.3389/fphys.2021.642237 (PMC7947915; doi:10.3389/fphys.2021.642237)
Supplement: Supplementary file 7 [file Data_Sheet_7.PDF]

up regulated				
ID	Name	P-value	Benjamini	Bonferroni
GO:001967	GDP-mannose metabolic process	0.008361	0.55681	0.702325
GO:000922	nucleotide-sugar metabolic process	0.022146	0.55681	1
GO:000038	spliceosomal snRNP assembly	0.024881	0.55681	1
GO:000907	aromatic amino acid family metabolic process	0.03304	0.55681	1
GO:005511	oxidation-reduction process	0.041939	0.55681	1

down regulated				
ID	Name	P-value	Benjamini	Bonferroni
GO:000681	cation transport	2.10E-05	0.001909	0.005993
GO:001567	monovalent inorganic cation transport	2.35E-05	0.001909	0.006712
GO:000681	transport	2.81E-05	0.001909	0.008037
GO:005123	establishment of localization	3.12E-05	0.001909	0.008921
GO:009866	inorganic cation transmembrane transport	3.35E-05	0.001909	0.009595
GO:005117	localization	4.00E-05	0.001909	0.011452
GO:009865	cation transmembrane transport	5.59E-05	0.002284	0.015985
GO:009866	inorganic ion transmembrane transport	6.85E-05	0.00245	0.0196
GO:190260	proton transmembrane transport	8.84E-05	0.002809	0.025285
GO:000681	ion transport	0.000453	0.012959	0.129592
GO:003422	ion transmembrane transport	0.000637	0.016559	0.182145
GO:003303	macromolecule localization	0.000735	0.017526	0.210309
GO:000915	purine ribonucleotide metabolic process	0.00092	0.019491	0.263192
GO:000616	purine nucleotide metabolic process	0.000954	0.019491	0.272875
GO:000925	ribonucleotide metabolic process	0.001098	0.020942	0.314131
GO:000609	generation of precursor metabolites and energy	0.001257	0.022474	0.359589
GO:001969	ribose phosphate metabolic process	0.001343	0.022586	0.383963
GO:004603	ATP metabolic process	0.00144	0.022873	0.411716
GO:007252	purine-containing compound metabolic process	0.001525	0.022955	0.436143
GO:000914	purine nucleoside triphosphate metabolic process	0.001868	0.025444	0.534333
GO:000920	purine ribonucleoside triphosphate metabolic proc	0.001868	0.025444	0.534333
GO:000919	ribonucleoside triphosphate metabolic process	0.00206	0.026777	0.589092
GO:000914	nucleoside triphosphate metabolic process	0.002263	0.02814	0.647226
GO:004428	small molecule metabolic process	0.002497	0.028663	0.714025
GO:005508	transmembrane transport	0.002517	0.028663	0.719964
GO:000916	purine ribonucleoside monophosphate metabolic p	0.002706	0.028663	0.7739
GO:000912	purine nucleoside monophosphate metabolic proce	0.002706	0.028663	0.7739
GO:002290	electron transport chain	0.003071	0.031367	0.878276
GO:000916	ribonucleoside monophosphate metabolic process	0.00333	0.032843	0.952441
GO:001714	drug metabolic process	0.003696	0.03366	1
GO:000911	nucleotide metabolic process	0.003916	0.03366	1
GO:000675	nucleoside phosphate metabolic process	0.003916	0.03366	1
GO:000912	nucleoside monophosphate metabolic process	0.004036	0.03366	1
GO:001963	organophosphate metabolic process	0.004096	0.03366	1
GO:000610	citrate metabolic process	0.0042	0.03366	1
GO:007178	endoplasmic reticulum tubular network organizatic	0.004237	0.03366	1
GO:007235	tricarboxylic acid metabolic process	0.00457	0.035324	1
GO:007170	organic substance transport	0.005066	0.038129	1
GO:005508	nucleobase-containing small molecule metabolic pr	0.007217	0.052576	1
GO:001699	antibiotic metabolic process	0.008038	0.052576	1

GO:000642	cysteinyl-tRNA aminoacylation	0.008456	0.052576	1
GO:004594	negative regulation of translational initiation	0.008456	0.052576	1
GO:004332	protein transport to vacuole involved in ubiquitin-d	0.008456	0.052576	1
GO:003251	late endosome to vacuole transport via multivesicul	0.008456	0.052576	1
GO:000701	cytoskeletal anchoring at plasma membrane	0.008456	0.052576	1
GO:004532	late endosome to vacuole transport	0.008456	0.052576	1
GO:000810	protein localization	0.010687	0.065031	1
GO:004316	ubiquitin-dependent protein catabolic process via t	0.012658	0.075421	1
GO:001975	carboxylic acid metabolic process	0.015316	0.083455	1
GO:004343	oxoacid metabolic process	0.015542	0.083455	1
GO:000608	organic acid metabolic process	0.016	0.083455	1
GO:004533	cellular respiration	0.016154	0.083455	1
GO:007266	establishment of protein localization to vacuole	0.016842	0.083455	1
GO:007266	protein localization to vacuole	0.016842	0.083455	1
GO:001972	calcium-mediated signaling	0.016842	0.083455	1
GO:003250	maintenance of protein location in cell	0.016842	0.083455	1
GO:005165	maintenance of location in cell	0.016842	0.083455	1
GO:000679	phosphate-containing compound metabolic proces	0.0173	0.083455	1
GO:000686	lipid transport	0.017512	0.083455	1
GO:000645	protein folding	0.017512	0.083455	1
GO:000679	phosphorus metabolic process	0.017846	0.083455	1
GO:001087	lipid localization	0.018916	0.083455	1
GO:003000	metal ion transport	0.018983	0.083455	1
GO:001598	energy derivation by oxidation of organic compoun	0.020367	0.083455	1
GO:007161	acyl-CoA biosynthetic process	0.02101	0.083455	1
GO:004518	maintenance of protein location	0.02101	0.083455	1
GO:000608	acetyl-CoA biosynthetic process	0.02101	0.083455	1
GO:001993	second-messenger-mediated signaling	0.02101	0.083455	1
GO:003250	endosome transport via multivesicular body sorting	0.02101	0.083455	1
GO:003538	thioester biosynthetic process	0.02101	0.083455	1
GO:000702	endoplasmic reticulum organization	0.02101	0.083455	1
GO:000644	regulation of translational initiation	0.02101	0.083455	1
GO:001619	vesicle-mediated transport	0.023331	0.091407	1
GO:007198	multivesicular body sorting pathway	0.025159	0.095857	1
GO:003201	regulation of ARF protein signal transduction	0.025159	0.095857	1
GO:003461	cellular protein localization	0.025472	0.095857	1
GO:007072	cellular macromolecule localization	0.025913	0.096249	1
GO:000662	lipid metabolic process	0.027319	0.100171	1
GO:001714	negative regulation of translation	0.029292	0.102164	1
GO:004685	phosphatidylinositol dephosphorylation	0.029292	0.102164	1
GO:003424	negative regulation of cellular amide metabolic proc	0.029292	0.102164	1
GO:005123	maintenance of location	0.029292	0.102164	1
GO:004683	phospholipid dephosphorylation	0.033407	0.108574	1
GO:003403	purine nucleoside bisphosphate biosynthetic proces	0.033407	0.108574	1
GO:003403	ribonucleoside bisphosphate biosynthetic process	0.033407	0.108574	1
GO:003386	nucleoside bisphosphate biosynthetic process	0.033407	0.108574	1
GO:006102	membrane fusion	0.033407	0.108574	1
GO:000608	acetyl-CoA metabolic process	0.033407	0.108574	1
GO:000998	cellular process	0.036848	0.116593	1
GO:003538	thioester metabolic process	0.037506	0.116593	1
GO:000663	acyl-CoA metabolic process	0.037506	0.116593	1
GO:000612	mitochondrial electron transport, NADH to ubiquin	0.037506	0.116593	1

GO:00091[illegible]	purine ribonucleotide biosynthetic process	0.03817	0.117382	1
GO:00061[illegible]	purine nucleotide biosynthetic process	0.039126	0.119043	1
GO:00463[illegible]	ribose phosphate biosynthetic process	0.043041	0.126906	1
GO:00725[illegible]	purine-containing compound biosynthetic process	0.043041	0.126906	1
GO:00092[illegible]	ribonucleotide biosynthetic process	0.043041	0.126906	1
GO:00150[illegible]	protein transport	0.044494	0.129851	1
GO:00158[illegible]	peptide transport	0.045679	0.131963	1
GO:00428[illegible]	amide transport	0.046278	0.132355	1
GO:00451[illegible]	establishment of protein localization	0.047487	0.134467	1
